# Large-Scale Differential Proteome Analysis in *Plasmodium falciparum* under Drug Treatment

**DOI:** 10.1371/journal.pone.0004098

**Published:** 2008-12-31

**Authors:** Judith Helena Prieto, Sasa Koncarevic, Sung Kyu Park, John Yates, Katja Becker

**Affiliations:** 1 Department of Cell Biology, The Scripps Research Institute, La Jolla, California, United States of America; 2 Interdisciplinary Research Center, Justus-Liebig-University, Giessen, Germany; The Research Institute for Children at Children's Hospital New Orleans, United States of America

## Abstract

Proteome studies contribute markedly to our understanding of parasite biology, host-parasite interactions, and mechanisms of drug action. For most antimalarial drugs neither mode of action nor mechanisms of resistance development are fully elucidated although this would be important prerequisites for successfully developing urgently required novel antimalarials. Here, we establish a large-scale quantitative proteomic approach to examine protein expression changes in trophozoite stages of the malarial parasite *Plasmodium falciparum* following chloroquine and artemisinin treatment. For this purpose SIL (stable isotope labeling) using ^14^N-isoleucine and ^13^C_6_,^15^N_1_-isoleucine was optimized to obtain 99% atomic percent enrichment. Proteome fractionation with anion exchange chromatography was used to reduce sample complexity and increase quantitative coverage of protein expression. Tryptic peptides of subfractions were subjected to SCX/RP separation, measured by LC-MS/MS and quantified using the novel software tool Census. In drug treated parasites, we identified a total number of 1,253 proteins, thus increasing the overall number of proteins identified in the trophozoite stage by 30%. A relative quantification was obtained for more than 800 proteins. Under artemisinin and chloroquine treatment 41 and 38 proteins respectively were upregulated (>1.5) whereas 14 and 8 proteins were down-regulated (<0.5). Apart from specifically regulated proteins we also identified sets of proteins which were regulated as a general response to drug treatment. The proteomic data was confirmed by Western blotting. The methodology described here allows for the efficient large-scale differential proteome analysis of *P. falciparum* to study the response to drug treatment or environmental changes. Only 100 µg of protein is required for the analysis suggesting that the method can also be transferred to other apicomplexan parasites.

## Introduction

The parasitic protozoon *Plasmodium falciparum* is responsible for approximately 500 million cases of malaria and one million deaths from malaria each year. Recent anti-mosquito measures and new artemisinin-containing treatments prompted calls for global malaria eradication. Novel drugs, vaccines, and insecticides, as well as deeper insights into parasite biology, human immunity, and vector behavior are essential to support these efforts [1]. Proteome studies contribute markedly to our understanding of parasite biology, host-parasite interactions, and mechanisms of drug action [2]. Respective analyses identifying proteins of different stages of malarial parasites have been carried out in our and other laboratories [3], [4]. Mass spectrometric (MS) methods like the Multidimensional Protein Identification Technology (MudPIT) were developed to enable large scale identification of proteins. In a typical MudPIT analysis an unfractionated protein mixture is digested to peptides, separated by biphasic liquid chromatography (SCX-RP-LC), and analyzed online by tandem mass spectrometry. Such approaches can include either *in vitro* or *in vivo* isotope tagging of amino acids which enables pair-wise comparison of protein expression patterns [5], [6]. Resulting data provide important insights into molecular mechanisms in cells including stress response and mechanisms of drug action and resistance.

Stable isotope labeling with heavy amino acids is a well established technique for protein labeling. It uses metabolic labeling, where the respective isotope-labeled amino acid is translationally inserted into proteins and no chemical labeling and purification steps after labeling are needed. Although stable isotope labeling of *Plasmodium* is complicated by the required use of red blood cell cultures, the application of SIL was developed for *P. falciparum* by Nirmalan *et al.* in 2004 in combination with 2DE [7]. Due to resembling physicochemical properties the isotope labeled proteins or peptides display similar separation characteristics in electrophoresis or chromatography. In liquid-chromatography separation prior to MS the labeled and unlabeled peptides co-elute from the chromatographic column. In the MS they can be resolved, measured, and compared in the same scans. The amino acid best suited for labeling proteins of malaria parasites is isoleucine [4]. It is not synthesized but taken up efficiently by *Plasmodium* and gives–^13^C_6_^15^N_1_ minus ^12^C_6_^14^N_1_–a spectral separation of 7.017159 Da. Of all *P. falciparum* proteins more than 99% have isoleucine containing peptides and thus can be theoretically covered in this approach (plasmodb.org).

In the present study we aimed at developing a large-scale quantitative proteomic approach in malarial parasites as an efficient method for studying cellular response to *e.g.* drug pressure or environmental changes. For this purpose we employed isoleucine-based SILAC in combination with proteome fractionation via anion exchange chromatography, SCX/RP for peptide separation, LC-MS/MS analysis, and quantification using the novel software tool Census. This strategy represents a general approach that can be used to study the mechanism of action for drug treatment of pathogens.

## Results

Within the framework of this study we established the first large scale comparative proteomics analysis for *Plasmodium falciparum*. To optimize labeling efficiency with ^13^C_6_^15^N_1_ isoleucine three complete cycles of growth (72 hours) were carried out leading to an average labeling efficiency of 99%. The use of high mass accuracy instruments and new software tools maximized the number of protein identifications, while at the same time enabling quantitative comparisons.

### Protein Identification

The measurement of tryptic digests of *P. falciparum* trophozoite stages by MudPIT led to the identification of 1,253 parasite proteins (or 6,318 peptides) in total. The numbers of identifications and regulated proteins for the drug-treated samples are shown in Table 1. The supplementary Table S1 contains all identifications from trophozoite extracts produced in this study. The data obtained allowed for a significant enhancement of protein identification numbers for this parasite stage compared to previous studies [3], [4].

**Table 1:** Total number of proteins identified in trophozoite stages of *Plasmodium falciparum* after drug treatment. Furthermore the numbers of regulated proteins are given.

	Chloroquine	Artemisinin
Total Protein Identifications with Isoleucine	1211	1165
Overlap with Control	889	814
Upregulated (>1.5)	41	38
Down Regulated (<0.5)	14	8

**Figure 1:**
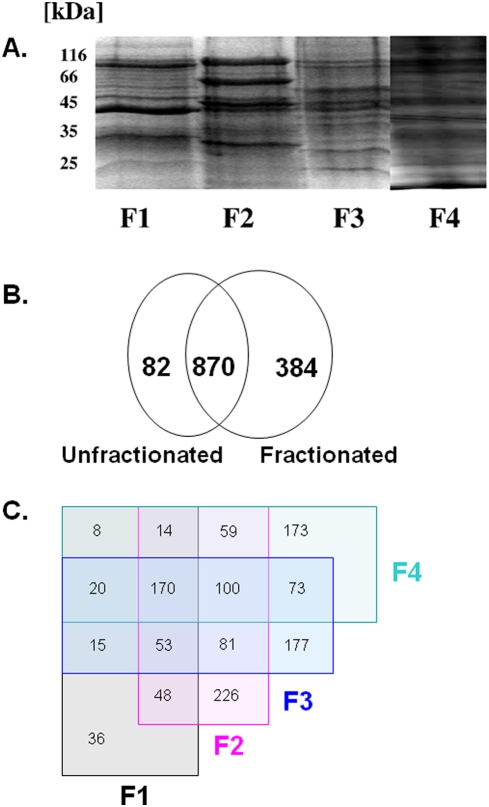
Quantification improvement by fractionation of cell extracts. A: Fractionation of *P. falciparum* trophozoite extracts by centrifugation and low-strength anion exchange chromatography. F1 to F3 are fractions derived by separation of the 100.000 g supernatant on low-strength anion exchanger chromatography. F1 (flowthrough); F2 (200 mM NaCl eluate); F3 (500 mM eluate); F4 (100.000 g pellet). The band pattern shows a fractionation into different subproteomes. B: Venn diagram comparing numbers of protein identifications of a previous MudPIT study by Florens *et al.* [3] and our current study, where prefractionation was applied. C: Venn diagram summarizing numbers of protein identifications per fraction and overlaps with other fractions.

This improvement was largely supported by a prefractionation step on the protein level. Fractionation of the soluble protein extract was performed on weak anion exchanger chromatographic columns to obtain three different protein fractions. These fractions plus the insoluble 100.000 *g* protein pellet constitute the four protein fractions used in our MS experiments. SDS-PAGE of the obtained fractions indicates that separation into subfractions was successful as the protein band patterns of the fractions differ considerably (Figure 1a).

To define the potential benefit of our fractionation procedure, we compared ID numbers of MudPIT runs of the unfractionated soluble sample to the fractionated soluble sample (three fractions). Through fractionation the overall number of IDs increased by about 30% as well as the quantification of all peptides identified. Figure 1b summarizes the number of protein identifications in non-fractionated and fractionated samples. Of all *Plasmodium* predicted proteins, all except one contain at least one isoleucine and thus could theoretically be quantified by an approach using labeled isoleucine.

In order to assess the benefit of the presented experimental setup, we submitted the mass spectrometric datasets of a previous study conducted in our lab [3] and the present study to the same search and filtering parameters for a comparable amount of MudPIT runs. We detected an increase in the number of protein identifications of 34% (data not shown). Thus, our study adds 384 proteins to the erythrocytic stage that had not been identified previously [3], [4], which represents a further step towards the completion of the *Plasmodium falciparum* proteome in the erythrocytic stages. The increase of identifications through fractionation is displayed in the Venn diagram (Figure 1c). Proteins that are exclusively found in the respective fractions are shown.

**Figure 2:**
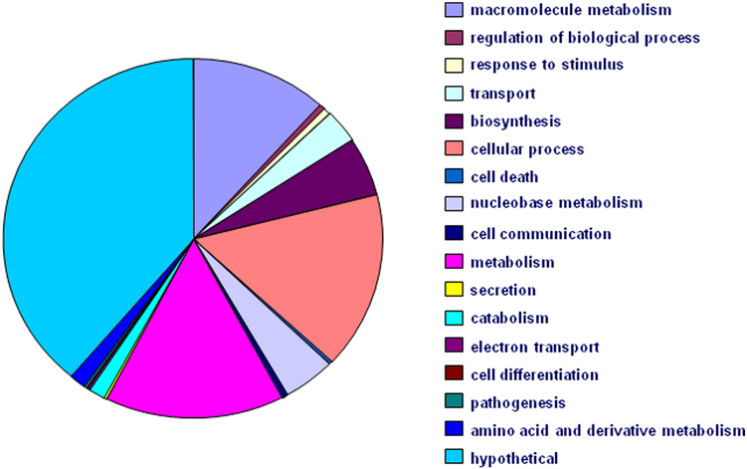
Functional classification of identified proteins. Identified proteins were classified using go annotations downloaded from PlasmoDB (www.plasmodb.org) and simplifying it with go-slim. The classification shows a widespread distribution of the identified proteins in metabolism.

The distribution of the functional profiles of the identified proteins is summarized in Figure 2. Large parts of the measured proteome contribute to important cellular functions including energy, amino acid, and nucleotide metabolism but also cell communication, transport, and biosynthesis. As deducible from a ranked list of all *P. falciparum* genes on PlasmoDB, many of these identified proteins represent important antimalarial drug targets. The opportunity to study these proteins quantitatively and differentially in a large scale approach will support future drug development strategies. In accordance with the genome annotations, a considerable percentage of the identified proteins are of unknown function. The methodology described here will facilitate more detailed studies.

### Relative quantification of isoleucine containing proteins

**Figure 3:**
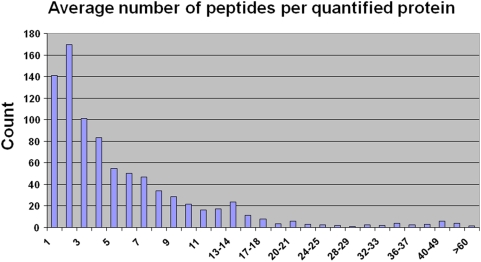
Number of peptides per protein used for quantification. Number of identified peptides with at least one isoleucine in their sequence per protein that have been quantified using Census against their incidence.

Relative quantification of isoleucine containing peptides was performed using the software tool Census [8]. We were able to quantify an average of 75% of all identified isoleucine-containing peptides for each dataset. In the chloroquine treated cells we relatively quantified 889 proteins, in the artemisinin treated cells 814. Figure 3 displays the count of peptides per quantified protein, showing that for quantification of 83% of the proteins at least two peptides were used. The average number of measured peptides per identified protein ranged from ∼5.8 to ∼6.2 in our experiments.

**Figure 4:**
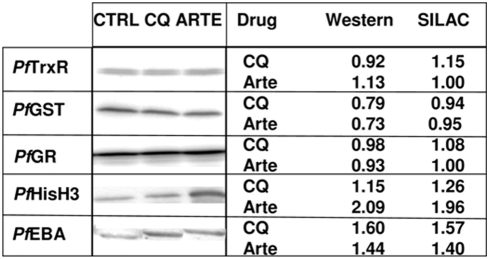
Validation of SILAC data by immunoblotting. The following polyclonal antibodies were used: rabbit-anti-*Pf*TrxR (thioredoxin reductase), rabbit-anti-*Pf*GST (glutathione S-transferase), rabbit-anti-*Pf*GR (glutathione reductase) (available in the Becker Lab), rabbit-anti-Histone H3 (Abcam, Cambridge, UK), rabbit-anti-*Pf*EBA (Erythrocyte Binding Antigen) (MRA-2 by MR4) at appropriate dilutions 1∶1,000–5,000 and peroxidase-conjugated anti-rabbit antibody (Dianova, Hamburg, Germany, anti-IgG, 1∶50,000–100,000). Visualization was performed by enhanced chemiluminescence (ECL, SuperSignal West Femto Maximum Sensitivity Substrate, Pierce Biotechnology, Rockford, IL, USA). Densitometric quantification was performed using the Quantity One software (BioRad) by quantitating band volumina (trace intensity×mm^2^) of each sample and comparing treated and control band volumina.

A validation of the quantification data was performed for five proteins for which antibodies were available. The immunoblots for the selected proteins are shown in Figure 4. The parasite proteins thioredoxin reductase (TrxR), glutathione reductase (GR), and thioredoxin (Trx) displayed no significant regulation in the mass spectrometry experiments (Table S1), which was confirmed in the immunoblots. For EBA-75 (erythrocyte binding antigen) we saw an upregulation by a factor of 1.6 in the CQ and 1.44 in the artemisinin SIL quantification as well as 1.57 and 1.40 in the immunoblot validation, respectively. Also histone H3, which was quantified in the SIL-MS analysis with 7 (CQ) and 8 (Arte) peptides as upregulated by 1.26 (CQ) and 1.96 (Arte), showed a similar regulation in the immunoblots with values of 1.15 (CQ) and 2.06 (Arte). These experiments prove that the quantifications determined by SIL deliver essentially the same results as the immunoblots. Thus our SIL approach is capable of presenting valuable protein profiling data for more than 800 proteins per comparison.

### Regulation of proteins under drug pressure

Our treatment regimen was to expose the cells in the early trophozoite stage to double IC_50_–values of the respective drug for twelve hours. An analysis of cell viability after certain time points showed that treated cells were fully viable and proved full reproductive abilities after treatment (see Methods for details). This information is important to verify that the observed proteome changes represent a specific drug response rather than changes induced by cell death.

The SIL quantification data show that a fraction of proteins was significantly regulated after treatment with chloroquine or artemisinin under chosen conditions (Table S1 and Table 2). When using a value of 0.5 for down regulation and of 1.5 for upregulation, we observed the following numbers. From 889 quantified proteins in chloroquine treated cells 41 were upregulated and 14 down regulated. In artemisinin treated cells, we observed 38 upregulated and 8 down regulated proteins from 804 quantified. These numbers, of course, vary with different set-offs for regulation factors used. Here we chose stringent factors that produce a manageable number of proteins.

**Table 2:** Regulated proteins in artemisinin and in chloroquine treated cells.

Protein ID	Description	RATIO Arte	Peptides Arte	RATIO CQ	Peptides CQ
MAL13P1.307	hypothetical protein	1.11	2	3.40	3
MAL13P1.92	40S ribosomal protein S15	0.72	1	1.78	2
PF07_0073	seryl-tRNA synthetase	0.97	10	1.51	7
PF11_0348	hypothetical protein	1.06	1	1.86	2
PF13_0198	reticulocyte binding protein 2 homolog a	1.24	1	1.65	1
PFC0135c	conserved protein	0.28	5	5.11	5
PFC0365w	conserved protein	1.02	3	1.55	4
PFC0730w	conserved protein	0.65	2	1.61	2
PFF0335c	hypothetical protein	0.99	3	1.61	2
PFF0615c	*P. falciparum* membrane protein pf12 precursor	0.74	2	1.66	2
PFI0235w	replication factor A-related protein	1.14	3	1.50	3
PFI0290c	beta subunit of coatomer complex	1.02	6	1.51	6
PFI1175c	RNA-binding protein	1.21	1	1.52	1
PFL0580w	DNA replication licensing factor mcm5	1.26	3	5.76	4
PFL1420w	macrophage migration inhibitory factor homolog	1.22	3	1.74	2
PFL1620w	asparagine/aspartate rich protein	1.28	2	1.65	3
MAL13P1.343	proteasome regulatory subunit	1.63	2	0.94	1
MAL8P1.127	hypothetical protein	1.52	3	0.96	5
PF07_0040	lysophospholipase-like protein	1.80	3	1.28	4
PF08_0121	peptidyl-prolyl cis-trans isomerase precursor	1.90	3	1.11	1
PF10_0043	ribosomal protein L13	2.02	1	0.83	8
PF10_0111	20S proteasome beta subunit	2.95	4	1.02	4
PF11_0053	*Pf*SNF2L	1.53	8	1.12	11
PF11_0384	hypothetical protein	1.60	1	1.29	1
PF13_0199	hypothetical protein	9.62	1	0.81	1
PF14_0242	arginine n-methyltransferase	2.09	2	0.89	2
PFB0835c	hypothetical protein	3.40	2	0.84	1
PFC0720w	hypothetical protein	1.50	4	1.11	2
PFC0915w	ATP-dependent RNA helicase	1.69	1	0.98	2
PFE0915c	proteasome subunit beta type 1	1.69	4	1.12	4
PFE1250w	long-chain fatty acid CoA ligase	3.11	2	0.84	3
PFF0445w	hypothetical protein	2.52	2	0.89	3
PFF0885w	60S ribosomal protein L27a	1.82	8	1.05	6
PFL2390c	hypothetical protein	1.86	3	1.17	3
PFL2465c	thymidylate kinase	4.88	2	1.05	2
MAL7P1.38	regulator of chromosome condensation protein	1.49	4	2.28	4
PF11_0061	histone H4	2.34	8	2.56	12
PF11_0142	hypothetical protein, conserved	2.94	2	1.37	3
PF11_0424	hypothetical protein	1.45	1	1.56	1
PF13_0102	DNAJ-like Sec63 homologue	1.34	5	1.43	6
PF13_0224	60S ribosomal subunit protein L18	1.84	4	1.45	4
PF13_0252	nucleoside transporter 1	1.47	2	1.49	2
PF14_0215	hypothetical protein	8.46	1	1.59	3
PF14_0231	ribosomal protein L7a	1.31	5	1.48	4
PF14_0567	hypothetical protein	1.60	7	1.68	6
PFC0262c	hypothetical protein, conserved	31.43	1	26.16	1
PFC0535w	60S ribosomal protein L26	1.86	1	1.33	1
PFC0900w	T-complex protein 1 epsilon subunit	1.31	6	1.55	2
PFC0945w	protein kinase	1.58	1	1.45	1
PFD0385w	hypothetical protein	1.61	3	2.15	2
PFD1035w	steroid dehydrogenase kik-i	1.94	1	2.56	3
PFD1070w	eukaryotic initiation factor	1.57	2	1.37	2
PFE0340c	hypothetical protein, conserved	1.35	3	1.33	3
PFE1150w	multidrug resistance protein	1.75	8	1.66	13
PFF0510w	histone H3	1.96	8	1.33	2
PFF0920c	hypothetical protein	3.07	1	3.00	1
PFI0975c	hypothetical protein	2.75	2	3.18	1
PFL0300c	phosphoesterase	1.89	2	1.69	2
PFL0720w	hypothetical protein	1.45	1	1.38	1
PFL1500w	Rab2 GTPase	1.92	1	1.96	1
PFL1550w	lipoamide dehydrogenase	1.40	4	1.43	2
MAL8P1.82	hypothetical protein	1.09	2	0.35	1
PF10_0031	hypothetical protein	0.91	6	0.18	2
PF14_0241	basic transcription factor 3b	0.93	2	0.01	1
PFC0390w	hypothetical protein, conserved	0.84	2	0.41	2
PFL1705w	hypothetical protein	0.86	6	0.36	1
MAL7P1.81	eukaryotic translation initiation factor 3 subunit	0.22	5	0.94	2
PF08_0132	glutamate dehydrogenase	0.23	2	1.32	2
PF11_0258	co-chaperone GrpE	0.39	2	1.11	2
PF13_0072	hypothetical protein	0.15	1	0.82	1
PFC0135c	conserved protein	0.28	5	5.11	5
PF07_0101	hypothetical protein	0.45	2	0.62	2
PF10_0170	hypothetical protein	0.67	2	0.53	2
PF11_0396	Protein phosphatase 2C	0.73	8	0.59	3
PF11_0433	hypothetical protein	0.63	3	0.59	1
PF13_0011	*Plasmodium falciparum* gamete antigen 27/25	0.46	3	0.24	2
PF14_0341	glucose-6-phosphate isomerase	0.73	6	0.69	7
PF14_0706	hypothetical protein	0.65	3	0.67	4
PFB0120w	hypothetical protein	0.58	1	0.56	2
PFB0640c	hypothetical protein	0.69	3	0.65	3
PFB0680w	hypothetical protein	0.56	8	0.70	7
PFB0830w	Ribosomal protein S26e	0.10	1	0.06	1
PFC1025w	F49C12.11-like protein	0.71	3	0.65	2
PFD0800c	hypothetical protein	0.70	1	0.66	1
PFD1130w	hypothetical protein	0.72	1	0.68	1
PFE1350c	ubiquitin-conjugating enzyme	0.72	1	0.66	3
PFF0860c	histone H2a	0.73	5	0.74	4
PFF0880c	hypothetical protein	0.70	5	0.67	7
PFL1155w	GTP cyclohydrolase I	0.66	3	0.38	3
PFL1340c	hypothetical protein	0.52	2	0.75	3
PFL1845c	calcyclin binding protein	0.71	3	0.45	2

Table 2 contains selected proteins regulated only under one treatment regimen (chloroquine or artemisinin) or equally regulated under both treatments. Proteins regulated only after chloroquine treatment include for example the conserved protein PFC0135c and the DNA replication licensing factor mcm5, which were both upregulated by more than fivefold and quantified with five or four peptides, respectively. These proteins were not upregulated under artemisinin treatment; rather, PFC0135C was markedly down regulated (0.28). Overall, sixteen proteins (with six hypothetical and conserved proteins) were specifically upregulated in chloroquine treated cells; six proteins were specifically down regulated. These include four hypothetical proteins and the basic transcription factor 3b.

Proteins specifically upregulated (>1.5) in artemisinin treated cells include 18 proteins (with seven hypothetical proteins). These comprise *e.g.* three proteasomal subunits, a thymidylate kinase and a long-chain fatty acid CoA ligase. Artemisinin treatment led to a reduced expression of five proteins, which include a hypothetical and a conserved protein, a eukaryotic translation initiation factor, a putative glutamate dehydrogenase and the putative co-chaperone GrpE. Interestingly, the conserved protein PFC0135c was down regulated after artemisinin treatment by a factor of 0.28, whereas it was upregulated under chloroquine pressure more than fivefold, as described above.

Table 2 furthermore contains proteins which were up- or down regulated similarly under both treatment regimens. Twenty-six proteins were upregulated under both drugs (>1.3). These proteins include histones H3 and H4, the lipoamide dehydrogenase, a steroid dehydrogenase and a multidrug resistance protein. Again, a huge proportion of proteins (10 out of 26) has not been assigned a function yet. Twenty proteins were down regulated by a factor of at least 0.75 in both samples. To this group eleven hypothetical proteins are counted as well as a putative calcyclin binding protein, a GTP cyclohydrolase I, the ribosomal protein S26e or a putative ubiquitin-conjugating enzyme.

750 proteins were observed in both studies (CQ and arte) and 680 of these showed the same trend (less than 20% difference among the two ratios) in both samples. The rest of the proteins displaying different regulations under artemisinin and chloroquine treatment, as summarized in Table 2, are of special interest as they should represent a drug specific response.

## Discussion

The first large-scale comparative study on the effects of treatment with the antimalarial drugs artemisinin and chloroquine on the proteome of *P. falciparum* was performed using metabolic labeling followed by identification and quantification with MudPIT LC-MS. The strategy of labeling proteins with stable isotope labeled isoleucine as described by Nirmalan *et al.* [7] for 2DE was used to create internal standards. By replacing the 2DE procedure with high-resolution LC-MS, where separation, identification and quantification are performed in parallel, we were able to identify and quantify a significantly larger number of proteins. To establish the method described here, the efficiency of labeling parasite proteins with ^13^C_6_^15^N_1_ isoleucine in cell culture was optimized until it reached 99%. Different methods were compared to fractionate cell extracts into sub-proteomes, including weak anion exchange chromatography with salt elution or pH elution and a sequential ammonium sulfate precipitation. As determined by direct comparison of fractionated and unfractionated samples, the fractionation procedure described in the methods section enhanced the number of identified proteins by 30%. Combining sample fractionation with the use of a high mass accuracy Orbitrap enabled the identification of an additional 384 proteins that had not been determined previously in proteome analyses. More than 800 proteins could be quantitatively compared between drug treated and untreated samples. Only 100 µg of protein was required for each LC-MS analysis of *Plasmodia*; thus the method is likely to be applicable for studying proteomic changes also in other apicomplexa for which the yield of cell extracts is limited. The experimental setup (parasite stage, drug dose, incubation time) was chosen on the basis of the available literature indicating that both CQ and artemisinin do act on the late ring/early trophozoite stages of the parasites [9], [10], [11] on desired high protein yields and on pretesting of the drugs in our laboratory.

### Effects of chloroquine

Chloroquine has been the mainstay of malaria prophylaxis and therapy for the last 50 years. Today, resistance to this affordable drug constitutes a major problem in the fight against malaria in most of the malaria endemic countries [12]. A range of possible mechanisms of action has been proposed for CQ. This includes interference with heme polymerization leading to the release of free parasitotoxic heme, intercalation with DNA and possible interference with DNA excision repair [13]. More recently, studying the effects of CQ on endocytosis of *Plasmodium falciparum* revealed an inhibition of vesicle trafficking [14]. As reported by Radfar *et al.*, CQ furthermore mediates specific proteome oxidative damage in the parasite [15]. As suggested by these independent results, CQ may actually act on multiple molecular targets in parallel, which could explain its extraordinary effectiveness over a long period of time.

In our study, most proteins showing increases in expression are localized to the nucleus or interact with chromosomes. Also proteins involved in translation show obvious quantitative expression changes. The amplitude of change remains low and variation in specific functional cascades is difficult to characterize, as already reported [16], [17]. One of the largest hindrances to identifying particular pathways affected by the drug is the high number of hypothetical proteins that show changes. These proteins can currently not be classified to a particular pathway, which will, however, improve over time. In addition, our data is in accordance with the observation of Gunasekera *et al.*, who observed broad mRNA expression changes in parasites treated with CQ [16], [17]. These changes affected particularly ribosomal proteins, signaling molecules, protein processing (heat shock proteins, proteasome subunits, cyclophilin), as well as RNA metabolism (transcription factors). There is a high degree of overlap between the functional group of proteins in the transcriptome data and our proteome study, however, the observed changes are less pronounced at the protein level. A direct and quantitative comparison of the proteome and transcriptome datasets has its limitations since parasite stages employed, synchronicity, drug doses and exposure times varied between the studies.

### Effects of artemisinin

Over the last years artemisinin has become indispensable as an alternative treatment of malaria as the CQ and the sulfadoxine/pyrimethamine combination have become increasingly ineffective [18]. As for the quinolines many hypotheses for the mechanism of action of artemisinin have been proposed [13], [19]. A key feature of all artemisinins is the 1,2,4-trioxane structure with its endoperoxide, which is essential for antimalarial activity. Active endoperoxides are thought to interact with reduced hemin or other sources of ferrous iron inside the parasite forming cytotoxic carbon-centered radical intermediates. These intermediates can alkylate biomolecules like lipids, heme and parasite enzymes including the ATP-dependent Ca^2+^ pump located on the endoplasmic reticulum, *Pf*ATP6.

Indeed, our data shows changes of the vacuolar ATP synthase; subunits α and γ (PF13_0130) display down regulation when the parasite is treated with artemisinin. In addition, several processes show slight upregulation under artemisinin when classifying the data with GO annotations. This upregulation affects mainly nucleotide and nucleic acid metabolism, transport and secretion as well as the expected response to stimuli. One of the most important observations is that the multidrug resistance gene was found to be upregulated under CQ and artemisinin indicating that *pfmdr1* indeed mediates resistance to a number of unrelated classes of agents [20].

Given the fact that approximately 40% of the identified and regulated proteins are hypothetical proteins with unknown functions in the parasite, the complexity of understanding the biology of the malaria parasite is illustrated. Our study is the first to dissect the protein expression response of chloroquine and artemisinin treated *Plasmodium falciparum* cells on the proteome level. We gain a list of sets of proteins specifically regulated under each of the two drugs and sets of proteins similarly regulated. Thus, our studies represent a basis for quantitative assessment of protein changes induced by drug treatment. Using this method, further investigations including different drug concentrations and time points in combination with cell biological parameters will allow to further understand mechanisms of action of single drugs or drug combinations.

## Methods

### Cultivation of *Plasmodium falciparum*

The chloroquine sensitive *P. falciparum* strain 3D7 was grown in continuous culture with slight modifications as described before [21]. The 3D7 strain is an established reference strain which had been employed in most of the previous proteomic and transcriptomic studies [9], [10]. Briefly, parasites were maintained at 1–10% parasitaemia and 3.3% haematocrit in RPMI 1640 culture medium supplemented with A+ erythrocytes, 4% A+ human serum, 0.2% lipid-rich bovine serum albumin (Albumax), 0.16% glucose, 0.2 mM hypoxanthine, 2.1 mM L-glutamine, and 22 µg/ml gentamycin. Cultures were kept at 37°C, 3% O_2_, 3% CO_2_ and 94% N_2_. Synchronization of parasites in culture to ring stages was carried out by repetitive treatment with 5% (w/v) sorbitol. Parasite growth and parasitaemia were monitored by assessing Giemsa-stained blood smears under the microscope. For determining IC_50_ and IC_90_ values on the parasites the semi-automated microdilution technique based on ^3^H-hypoxanthine incorporation was applied. For immunoblotting analyses we prepared biological replicates without SILAC and treated cells equally as in the SILAC experiment.

### Heavy isotope labeling of *P. falciparum* proteins

For the drug exposure and heavy isotope labeling, specialized cell culture conditions were applied. For this purpose we combined the procedures published by Nirmalan *et al.*, 2004 [7] and Koncarevic *et al.*, 2007 [21]. In contrast to standard conditions, parasites were kept in the absence of human serum, which was replaced by Albumax only. For heavy isotope labeling studies, custom RPMI-1640 medium devoid of isoleucine was supplemented with ^13^C_6_,^15^N_1_-isoleucine (≥98% isotopic purity; Cambridge Isotope Laboratories, Andover, MA, USA) to yield 7 Da mass shifts per isoleucine in a peptide. Synchronized cultures were preincubated for 24 h in isoleucine free medium before 0.38 mM heavy (^13^C_6_,^15^N_1_) isoleucine (52.7 mg/ml) or light isoleucine (50 mg/ml) was added to the parallel cultures. Isoleucine incorporation was applied for three complete life cycles (3×48 h) before addition of the drugs. Blood stage cultures with a parasitemia of 8–10% at the late ring/early trophozoite stage (24–28 h post infection) were incubated with chloroquine (CQ) or artemisinin (Arte) (2×IC_50_ values = 17 nM for CQ and 35 nM for Arte, determined 72 hrs after addition of a drug which is not removed from the cell culture) or the used solvent (RPMI-1640 medium or DMSO) for control cultures. This experimental setup was based (a) on pretesting of stage specificity and drug concentrations in our lab (b) on the fact that it was desirable to harvest mature trophozoites in order to obtain enough protein for the proteome analyses, and (c) on the available literature which indicates that maximum effects of the two drugs occurred at the late ring and early trophozoite stage, which corresponds to the time at which the most rapid increases in synthetic and glycolytic activities occur. Mature schizonts and young rings are less affected by the antimalarial drugs [9], [10]. This data was recently substantiated by Maerki *et al.* [11] who confirmed in their experiments that trophozoites were more susceptible to CQ than rings or schizonts and that also for artemisinin good effects on trophozoites were determined after 12 h.

For each drug, four samples were run in parallel: labeled control, unlabeled control, labeled sample, and unlabeled sample. After 12 hours of drug exposure the parasites had reached the late trophozoite/schizont stage and were harvested in parallel with the controls. The window of the sorbitol-induced synchrony (>90%) at which the trophozoites were harvested was approximately 5 hours. This window was achieved by repetitive sorbitol-induced synchronizations (48 h, 96 h, 144 h, and 192, and 198 h before the ring stage parasites that were later used for the experiment were obtained). Since no reinvasion step was present during drug exposure, the incubation time was rather short and the drug concentrations were rather low (2×IC_50_) the parasitemia did not change within the 12 hours after drug treatment.

### Parasite viability after drug treatment

A control experiment was conducted in order to assess the viability of the parasites after drug exposure. Parasites (6.4% parasitemia) were exposed to the drugs as described above. After 12 h the drugs were removed by changing the medium and washing the cells once in medium. Cultures were then maintained under normal growth conditions. The next schizogony took place simultaneously in the three cultures resulting in 11.6% parasitemia with 5.4% residual schizonts (CQ), in 11.6% parasitemia with 5.1% schizonts (Arte) and in 11.7% parasitemia with 5.0% schizonts at the given window of the sorbitol-induced synchrony. In spite of similar growth behavior, drug treated schizonts appeared slightly denser under the light microscope. Cultures were then splitted (1∶5 dilution to reduce the high parasitemia) and incubated further. 17 h after peak schizogony parasitemias of 6.3% rings (CQ), 6.4% rings (Arte) and 6.4% (control) were determined.

The data indicates that–as intended-the drug treatment protocol applied in our study did only slightly affect the parasites and did not lead to major growth retardation or cell death. Treated and untreated parasites differed by less than 1 hour in their developmental stage. In fact, the aim in our study was to distinguish between the changes from halted growth and the direct effect of drug treatment using two drugs that are known to act on trophozoites. The protein expression changes observed under both drugs are likely to reveal the changes observed from halted development whereas the differences are related to the specific action of each drug. By using this method, further investigations using different drug concentrations and time points in combination with cell biological parameters will allow a better understanding of mechanisms of action of single drugs or drug combinations.

### Preparation of parasite extracts

The preparation of parasite extracts followed established protocols [21]. Briefly, parasites were isolated by lysing the red blood cell in saponin containing buffer (7 mM K_2_HPO_4_, 1 mM NaH_2_PO_4_, 11 mM NaHCO_3_, 58 mM KCl, 56 mM NaCl, 1 mM MgCl_2_, 14 mM glucose, 0.02 mM saponin, pH 7.5) followed by intensive washing. Parasites were disrupted by three cycles of freezing and thawing and ultrasonication in digestion buffer (4 M urea, 0.4% Triton X-100, 50 mM Tris-HCl, 5 mM EDTA, 10 mM MgSO_4_, pH 8.0) in the presence of protease inhibitors. RNA/DNA-digestion was performed with benzonase® (Merck) for 30 min at 4°C. A first centrifugation step at 15,000 g removed contaminating haemozoin. The supernatant after centrifugation at 100,000 g for 30 min was used as “parasite” extract for fractionation. The insoluble pellet was used as “insoluble fraction” for the analyses.

### Protein immunoblotting analysis

The electrophoretic pattern of some proteins was investigated by immunoblot analysis using specific polyclonal antibodies. Proteins were resolved by Tris-Tricine-PAGE and were transferred onto a PVDF membrane. The blots were incubated with antibodies and washed according to standard procedures. The following polyclonal antibodies were used: rabbit-anti-*Pf*TrxR, rabbit-anti-*Pf*GST, rabbit-anti-*Pf*GR (available in the Becker Lab), rabbit-anti-Histone H3 (Abcam, Cambridge, UK), rabbit-anti-*Pf*EBP1 (MRA-2 by MR4) at appropriate dilutions 1∶1,000–5,000 and peroxidase-conjugated anti-rabbit antibody (Dianova, Hamburg, Germany, anti-IgG, 1∶50,000–100,000). Vizualisation was performed by enhanced chemiluminescence (ECL, SuperSignal West Femto Maximum Sensitivity Substrate (Pierce Biotechnology, Rockford, IL, USA). Densitometric quantitation was performed using the Quantity One software (BioRad) by quantitating band volumina (trace intensity×mm^2^) of each sample and comparing treated and control band volumina.

### Proteome subfractionation

To reduce sample complexity (number of protein species/sample) and to enrich proteins being of “lower abundance” out of the unfractionated samples, we established a procedure for subfractionating the *Plasmodium falciparum* proteome. Before fractionation, samples to be compared (light and heavy) were mixed in a protein to protein ratio of 1∶1 wt/wt. Weak anion exchanger chromatography was used to subfractionate the soluble proteins of *P. falciparum*. Vivapure Mini H, D ion exchanger columns (Diethylamine (D). Weak basic anion exchanger (R-CH_2_-NH^+^-(CH_2_H_5_)_2_, Sartorius, Germany) were loaded with the sample according to the manufacturer's instructions. The columns were equilibrated with a buffer containing 2 M urea, 0.2% Triton, 25 mM Tris, 2.5 mM EDTA, 5 mM MgSO_4_, pH 8.0 and then loaded with 1∶1 mixed protein extract. Bound proteins were eluted according to two different procedures. The first procedure included elution according to pH (buffer containing 2 M urea, 0.2% Triton, 25 mM Tris (or sodium acetate), 2.5 mM EDTA, 5 mM MgSO_4_, pH 8.0, 7.0, and 3.0. For effective elution each step was performed with three single column volumina. The second elution procedure was performed by stepwise increasing salt concentrations (200–500 mM NaCl). Furthermore a third procedure for crude sample fractionation was applied by sequential NH_4_SO_4_ precipitation (40%, 70%, and 90% NH_4_SO_4_). Protein concentration in eluted samples was measured, fractions were precipitated by OrgoSol™-DETERGENT-OUT™ Detergent Removal Kit (Calbiochem, Darmstadt, Germany) and separated by SDS-PAGE to monitor fractionation or used for LC-MS analyses.

### Multidimensional protein identification technology (MudPIT)

Precipitated *P. falciparum* protein preparations were dissolved in digestion buffer, digested with trypsin and LysC, and analyzed by LC/LC/MS/MS according to published protocols [22], [23]. Approximately 100 µg of protein was used for a 6-step for soluble samples and 12-step for insoluble LC/LC/MS/MS analysis on a LTQ-Orbitrap, a hybrid mass spectrometer in which a linear ion trap is coupled to an Orbitrap mass analyzer (ThermoElectron, San Jose, CA). All samples were analyzed in triplicate. The obtained MS/MS spectra were analyzed with SEQUEST 2.7 [24], [25] using a non-redundant *Plasmodium* database (PlasmoDB version 5.0) [26]. The SEQUEST outputs were analyzed by DTASelect 2.0 [27]. DTASelect 2.0 uses a quadratic discriminant analysis to dynamically set XCorr and DeltaCN thresholds for the entire data set to achieve a user-specified false positive rate (5% in this analysis). The false positive rates were estimated by the program from the number and quality of spectral matches to the decoy database [28].

### Data processing

After filtering the results from SEQUEST using DTASelect2, ion chromatograms were generated using an updated version of a program previously written in our laboratory [29]. This software, called Census [30], is available from the authors for individual use (for details, see http://fields.scripps.edu/Census).

Census calculates peptide ion intensity ratios for each pair of extracted ion chromatograms. The basis of the program is a linear least-squares correlation that is used to calculate the ratio (i.e., slope of the line) and closeness of fit (i.e., correlation coefficient [*r*]) between the data points of the unlabeled and labeled ion chromatograms. In this study, only peptide ratios with determinant scores (r2>0.5) were used for further analysis [31].

Peptides were evaluated after first taking the union of search results (DTASelect2.0 output files) so that a peptide needs only be identified in one of the replicates to be quantified.

Quantification data of proteins were acquired by calculating a weighted average of the identified and quantified peptides of the protein from all fractions. The output from Census is a ratio 1 of the unlabeled and labeled sample for the drug treated sample (unlabeled drug treated_1_/labeled control_1_) as well as a ratio 2 for the control sample (unlabeled control_2_/labeled control_2_) of a different MudPIT run (normalization run). Through calculation of a ratio of ratios (1/2) for each quantified peptide we filtered out eventual labeling deficiencies and received a ratio that corresponds to (unlabeled drug treated/unlabeled control). The changes observed by Census identify proteins with differential expression status upon drug treatment.

## Supporting Information

Table S1 (1.69 MB DOC)
