# Supplementary material for: Large-Scale Differential Proteome Analysis in Plasmodium falciparum under Drug Treatment
Source: PLoS One. 2008 Dec 31;3(12):e4098. doi: 10.1371/journal.pone.0004098 (PMC2605551; doi:10.1371/journal.pone.0004098)
Supplement: Supplementary file 1 [file pone.0004098.s001.doc]

Locus	Sequence	Spectrum XCorr	Sequence DeltCN	Length Conf%	MolWt M+H+	pI CalcM+H+	Validation TotalIntens	Descriptive Name SpR	ZScore	IonProport	Redundancy
PFC0400w		1058	86.60%	112	11948	4.5	U	Annotation|Plasmodium_falciparum_Sanger|(protein coc			
	K.YVAAYL	4.6227	0.5025	100	2086.997	2086.994	7604.1	1	8.412	72.2	3
	K.NVLGAV	5.0457	0.4159	100	2489.261	2488.257	9337.4	1	6.942	36.4	4
	K.NVLGAV	6.2103	0.4379	100	2492.27	2488.257	11137.2	1	8.945	61.4	91
	V.LGAVNA	3.8015	0.444	100	2276.15	2275.146	7056.9	1	6.535	45	2
	L.GAVNAD	5.4397	0.4569	100	2165.076	2162.062	6719.5	1	8.871	65.8	4
	G.AVNADV	5.2856	0.3695	100	2106.045	2105.04	9300.6	1	6.933	44.4	7
	G.AVNADV	7.1926	0.5046	100	2113.074	2112.057	9043.2	1	10.053	72.2	264
	G.KSCHEL	4.0349	0.2697	100	1400.716	1400.721	6961.5	1	5.964	77.3	5
	K.SCHELI	4.0231	0.3038	100	1272.628	1272.626	4876.8	1	6.274	85	9
	K.SCHELI	4.546	0.231	100	1400.721	1400.721	5841.3	1	6.339	63.6	6
	K.SCHELI	4.7111	0.3553	100	1407.747	1407.738	9569.6	1	6.887	86.4	29
	K.KLQNIG	4.8058	0.4498	100	2266.222	2265.22	9328	1	8.597	35.4	5
	K.KLQNIG	6.9953	0.5433	100	2273.242	2272.238	7533.8	1	10.257	64.6	9
	K.KLQNIG	6.3334	0.5697	100	2394.321	2393.315	7396.9	1	10.772	41	6
	K.LQNIGG	5.84	0.5122	100	2138.132	2137.125	7431.7	1	8.803	43.5	13
	K.LQNIGG	6.4334	0.4274	100	2147.152	2144.143	5989	1	7.894	67.4	18
	K.LQNIGG	5.2045	0.2827	100	2272.242	2272.238	5840.7	1	7.234	54.2	3
	Q.NIGGGV	3.9855	0.5609	100	1895.981	1895.983	5212.8	1	9.816	52.4	2
	Q.NIGGGV	6.0653	0.5474	100	1898.997	1895.983	4697.9	1	9.137	64.3	24
	Q.NIGGGV	3.9188	0.3336	99.9	1903.003	1903	9924.5	1	6.655	40.5	6
	Q.NIGGGV	4.2069	0.4499	100	2025.086	2024.078	4716.2	1	7.984	40.9	3
	N.IGGGVA	4.5553	0.4839	100	1788.96	1788.957	4298.5	1	7.913	60	4
	A.AAPAGA	3.7817	0.5005	100	1327.686	1327.686	6949.1	1	8.615	60.7	12
	A.AAPAGA	5.117	0.4994	100	1328.688	1327.686	4313.8	1	8.796	85.7	12
	A.APAGAA	3.327	0.4386	100	1256.649	1256.649	5246.2	1	9.499	69.2	1
	A.APAGAA	4.1641	0.4587	100	1256.65	1256.649	8484	1	8.519	84.6	3
	K.KEEEEE	5.0001	0.4586	100	2004.86	2001.845	7825.9	1	8.041	75	513
MAL13P1.214		1023	82.00%	266	31043	5.6	U	Annotation|Plasmodium_falciparum_Sanger|(protein coc			
	M.TLIENL	4.7856	0.5212	100	2789.371	2785.353	7226.5	1	8.67	47.8	14
	M.TLIENL	6.08	0.3353	100	2794.382	2792.37	11346.8	1	6.385	34.8	12
	K.TFLENN	4.6091	0.3277	100	1660.783	1657.771	6916.4	1	7.26	84.6	21
	V.KVYEFI	5.2597	0.4537	100	2366.194	2366.211	8588	1	8.283	55	5
	K.VYEFIF	5.4634	0.4697	100	2240.121	2238.116	10023	1	9.135	65.8	83

	K.VYEFIF	4.8268	0.4414	100	2366.197	2366.211	9276.8		1	8.27	50	32
	E.FIFGEN	3.5078	0.3906	100	1960.987	1961.002	6779.5		1	6.211	61.8	2
	K.KILSDIE	3.5483	0.3057	100	1516.842	1516.841	7791.8		1	6.15	79.2	1
	K.ILSDIEL	4.7136	0.3439	100	1374.717	1374.712	6257.7		1	7.1	90.9	8
	K.VLDIGS	5.1919	0.5163	100	1882.909	1882.904	7304.4		1	10.21	79.4	5
	K.VLDIGS	3.8274	0.4849	100	1896.942	1896.939	5722.1		1	8.116	50	8
	K.YGAHTH	3.0734	0.3526	99.9	1444.632	1444.628	6474		1	5.938	66.7	6
	K.YGAHTH	3.4225	0.4838	100	1459.667	1458.662	3722.7		1	7.63	54.2	4
	K.YGAHTH	4.0044	0.4327	100	1770.826	1770.823	3625.9		1	8.972	60	9
	K.YGAHTH	3.8568	0.3582	100	1794.886	1791.875	3693.8		1	5.762	63.3	7
	K.YGAHTH	6.2058	0.4767	100	2393.122	2393.139	4814.5		1	8.662	42.5	36
	K.YGAHTH	6.4597	0.5833	100	2396.127	2393.139	8496.4		1	9.795	67.5	205
	K.IIFEAND	3.8319	0.3308	100	1297.769	1297.767	4004.1		1	6.309	90	28
	T.KEFPEN	4.091	0.3432	100	1779.888	1778.883	6837.2		1	6.598	69.2	6
	K.EFPENN	3.306	0.3544	100	1643.764	1643.771	4249.7		1	6.322	79.2	4
	R.DAILHL	3.2328	0.3557	100	1259.708	1259.707	7137.7		1	5.972	80	2
	K.WLKPTG	4.2376	0.304	99.9	2110.081	2110.089	8089.6		1	6.601	41.2	7
	K.WLKPTG	6.1255	0.456	100	2119.112	2117.107	5661.8		1	9.033	70.6	137
	K.PTGTLL	5.2675	0.5178	100	1691.854	1689.848	7241.4		1	8.416	71.4	16
	K.ENWDDI	3.7732	0.3086	100	1601.716	1601.712	8950.4		1	7.098	72.7	15
	K.YTLITV	4.1795	0.441	100	2279.155	2278.15	6565.8		1	7.247	55.6	42
	S.KDLSDY	4.8672	0.2055	100	2017.006	2017.003	6428.6		1	6.236	50	7
	S.KDLSDY	4.4983	0.4541	100	2017.007	2017.003	8365.9		1	7.943	60	21
	K.DLSDYW	3.2969	0.3404	99.8	1888.92	1888.908	5912.7		1	6.371	50	2
	K.DLSDYW	3.1274	0.378	99.9	1889.917	1888.908	2947.6		12	5.68	35.7	3
	K.DLSDYW	5.1434	0.4082	100	1891.917	1888.908	10805.7		1	7.174	75	209
	D.LSDYWN	3.3879	0.3363	100	1774.887	1773.881	6219.6		1	6.329	53.8	6
	K.YLHENK	3.9261	0.2446	100	1449.735	1449.738	5954.9		1	6.205	80	42
	K.YLHENK	3.4723	0.2739	99.9	1449.737	1449.738	3877.1		1	5.517	60	3
	L.HENKEE	2.7984	0.2862	99.9	1173.594	1173.591	6694.8		1	5.961	81.2	3
	K.KFISLD	3.8203	0.1874	99.9	1323.665	1323.67	6579.6		1	5.957	80	12
PF14_0598		7791	80.10%	337	36635	7.7	U	Annotation|Plasmodium_falciparum_TIGR|(protein codin				
	K.LGINGF	2.8065	0.3486	100	840.4771	840.4805	4639		1	6.234	92.9	6
	R.KDIEVV	4.1566	0.4274	100	2760.417	2760.41	5387.2		1	7.376	50	32
	R.KDIEVV	7.4524	0.4757	100	2778.449	2774.445	7546.5		1	8.786	43.2	11
	K.DIEVVA	5.1218	0.3644	100	2129.005	2129.023	10121.1		1	7.723	58.8	12

K.DIEVVA	4.2025	0.2937	99.9	2647.336	2646.35	5038.9	7	5.954	26.2	2
K.DIEVVA	5.2426	0.4937	100	2649.363	2646.35	4665.2	1	8.501	50	360
D.PFMDLN	4.9608	0.3663	100	1663.846	1663.834	11823.6	1	7.696	87.5	21
K.YDSVHC	5.6415	0.4528	100	2719.269	2719.283	8439.9	1	7.919	37	102
K.YDSVHC	5.2132	0.5284	100	2729.309	2726.3	8276.8	1	8.74	47.8	267
K.YDSVHC	5.1903	0.4618	100	2854.383	2854.395	9121.7	1	6.96	33.3	1
K.KVSVFA	2.9489	0.2886	99.9	907.5259	907.5253	3598.2	1	4.841	92.9	3
K.KVSVFA	5.3194	0.3415	100	1917.019	1916.028	6909.5	1	5.909	45.3	24
K.KVSVFA	5.2159	0.4234	100	1923.034	1923.045	6098.7	1	7.402	65.6	54
K.KVSVFA	4.371	0.3693	100	1925.06	1923.045	5450.3	1	5.911	56.2	11
K.VSVFAE	4.7157	0.4169	100	1795.956	1794.95	6430.2	1	6.923	66.7	18
K.CQVDVV	6.0685	0.5087	100	1841.865	1841.878	8107	1	9.39	83.3	1342
K.CQVDVV	3.6798	0.4023	100	1841.89	1841.878	3464.9	1	6.748	56.7	9
K.KVIMSA	3.5082	0.2483	100	977.5857	977.5931	4637.3	1	5.849	87.5	5
K.KVIMSA	2.9053	0.3224	99.8	1919.007	1919.021	7209	5	5.369	43.8	1
K.VIMSAP	6.578	0.4675	100	2991.511	2991.501	6706.8	1	8.093	37	61
K.VIMSAP	4.2607	0.3754	100	2995.533	2991.501	7652.5	1	7.302	40	55
I.MSAPPK	3.768	0.415	100	2758.312	2758.297	6569.2	1	6.509	31.5	2
M.SAPPKD	4.2795	0.4499	100	2627.26	2627.256	6752.1	1	8.026	47.7	6
A.PPKDDT	4.2221	0.3956	100	2483.236	2483.222	7601.8	1	6.63	37.5	1
A.PPKDDT	4.2818	0.4021	100	2484.256	2483.222	5522.5	1	7.816	52.5	8
K.DDTPIY	4.8019	0.5256	100	2161.024	2161.021	6910.6	1	8.317	55.9	21
V.MGINHH	3.1036	0.3923	100	1343.619	1343.617	4262.3	1	6.469	70	2
M.GINHHQ	3.2986	0.3506	100	1215.586	1212.576	5043.6	1	5.864	72.2	14
K.QLIVSN	5.7915	0.4684	100	2068.073	2068.065	7377.6	1	9.488	75	301
I.VSNASC	3.8733	0.489	100	1706.816	1706.821	6538.8	1	8.168	70	3
R.FGIVEG	7.1422	0.496	100	2671.363	2671.377	8326.8	1	9.543	45	1197
R.FGIVEG	6.3078	0.5288	100	2672.37	2671.377	7719.4	1	10.171	54	2118
I.VEGLMT	4.008	0.3481	100	2357.215	2354.203	5393.2	1	6.92	45.5	95
V.EGLMTT	3.6975	0.3261	100	2256.144	2255.134	5624.8	1	6.506	45.2	35
E.GLMTTV	3.9678	0.4179	100	2126.093	2126.092	5380.8	1	7.305	55	50
M.TTVHAS	4.1981	0.4178	100	1825.951	1824.946	5921	1	7.179	61.8	46
T.TVHAST	3.813	0.4616	100	1723.898	1723.898	5281.8	1	8.38	50	3
T.TVHAST	4.6612	0.4012	100	1724.901	1723.898	6629.6	1	8.065	62.5	125
V.HASTAN	3.2261	0.4571	100	1523.784	1523.782	5199.5	1	8.561	60.7	3
V.HASTAN	3.8214	0.3646	100	1523.786	1523.782	7078	1	8.712	71.4	6

	H.ASTANQ	3.4934	0.2534	99.9	1386.719	1386.723		6772.1	5	4.769	65.4	3
	R.CALSNI	3.4781	0.4592	100	1489.818	1487.808		3800.8	1	7.768	64.3	1
	R.CALSNI	4.9297	0.3936	100	1490.813	1487.808		5600.2	1	7.11	78.6	7
	A.FRVPIG	3.9528	0.4092	100	1660.898	1660.91		7539.5	1	6.901	78.6	3
	F.RVPIGT	3.0929	0.4063	100	1676.97	1676.96		5582.9	1	6.753	64.3	1
	R.VPIGTV	4.1008	0.3992	100	1520.859	1520.858		3845.9	1	7.584	69.2	79
	R.VPIGTV	4.5636	0.2614	100	1520.862	1520.858		6955.3	1	6.145	59.6	6
	R.VPIGTV	4.3354	0.5325	100	1520.863	1520.858		7448.7	1	8.615	76.9	252
	V.PIGTVS	4.444	0.497	100	1421.79	1421.79		6839.1	1	8.718	91.7	120
	R.LQKPAK	4.3474	0.4518	100	1766.007	1766.018		5930.2	1	7.215	75	5
	R.LQKPAK	4.8349	0.4834	100	1894.116	1894.113		6447.8	1	7.912	70	32
	K.YEEVAL	2.8538	0.2712	99.8	1221.678	1221.673		7799.2	1	5.785	88.9	2
	K.GILGYT	3.7311	0.4546	100	2300.076	2300.091		5437.8	1	7.059	36.8	6
	K.GILGYT	5.384	0.5038	100	2300.079	2300.091		7354.5	1	9.877	63.2	275
	I.LGYTED	4.0915	0.3911	100	2122.98	2122.968		6993.6	1	7.521	39.7	6
	I.LGYTED	4.3358	0.5178	100	2122.984	2122.968		7840.1	1	8.826	64.7	9
	L.GYTEDE	4.729	0.4977	100	2010.885	2009.884		6694.1	1	9.191	71.9	31
	G.YTEDEV	5.0961	0.5713	100	1952.857	1952.863		7012.4	1	10.226	76.7	117
	G.YTEDEV	3.1419	0.3654	99.9	1952.869	1952.863		4553.4	1	5.753	46.7	3
	Y.TEDEVV	3.5072	0.423	100	1789.803	1789.799		6991.8	1	7.598	57.1	6
	Y.TEDEVV	3.7031	0.3673	100	1789.812	1789.799		7433.7	1	6.901	64.3	9
	R.SSIFDM	2.7355	0.2924	99.9	827.3994	827.3973		4022.5	1	5.604	91.7	4
	K.AGLALN	3.8049	0.2288	99.9	1211.635	1209.627		6346.7	3	4.922	85	60
	K.LVSWY	4.8836	0.4386	100	1788.788	1788.798		9188.8	1	9.063	76.9	237
	K.LVSWY	3.3713	0.4947	100	1788.805	1788.798		4110.3	1	7.536	57.7	4
	L.VSWYD	2.9125	0.336	99.9	1675.717	1675.714		6734.5	2	5.213	54.2	2
	V.SWYDN	2.8236	0.4216	100	1576.648	1576.646		8329.8	1	6.54	63.6	3
	R.VLDLAV	3.9593	0.306	100	1215.673	1215.681		7064.8	1	7.189	80	75
PFE0660c		1809	76.30%	245	26858	6.5	U		Annotation|Plasmodium_falciparum_Sanger|(protein coc			
	K.ISKEQI	4.3888	0.4458	100	2263.327	2263.321		6831.6	1	7.264	40	3
	K.EQITPV	4.6919	0.4603	100	1581.898	1578.886		7643.9	1	8.316	75	9
	K.EQITPV	5.2201	0.5422	100	1921.082	1921.076		4406.8	1	9.224	64.7	8
	K.VVCDSY	4.6257	0.4594	100	1575.741	1573.732		8615.5	1	8.126	83.3	34
	K.FLCVS	6.1963	0.5317	100	2858.259	2857.253		7220	1	8.565	39	301
	K.FLCVS	5.7001	0.548	100	2859.265	2857.253		8812.9	1	9.328	52	152
	F.LCVSH	4.8578	0.4954	100	2711.195	2710.185		7430.2	1	7.813	36.5	3

	R.AGSCGS	3.9829	0.4024	100	1501.784	1501.78	5097.4	1	6.564	69.2	8
	K.RGDICI	3.4704	0.3208	99.9	1418.721	1418.718	3343.2	1	5.759	54.5	1
	K.RGDICI	4.4956	0.4305	100	1418.721	1418.718	6057.2	1	8.022	90.9	54
	R.GDICIC	3.8054	0.3717	100	1263.622	1262.617	6568	1	7.036	80	1
	R.VSHLLI	5.9946	0.5295	100	2675.353	2672.336	9772.3	1	10.113	58.7	493
	R.VSHLLI	8.111	0.5204	100	2680.365	2679.353	9596.4	1	9.825	46.7	367
	K.CAQELN	5.6927	0.5235	100	2740.295	2739.287	4398	1	8.924	58.7	21
	K.ANAAVV	5.6213	0.3843	100	2103.142	2102.135	6944.5	1	6.851	43.4	102
	K.ANAAVV	5.3242	0.3663	100	2105.148	2102.135	7711.9	1	7.522	57.9	192
	K.ANAAVV	4.3661	0.5261	100	2238.258	2237.248	10303.8	1	8.356	50	3
	K.TGGILI	3.1179	0.3647	99.8	1390.763	1390.759	4833	1	7.096	62.5	1
	K.TGGILI	4.7092	0.3364	100	1391.766	1390.759	6451.7	1	6.932	79.2	6
	K.TGGILI	7.2661	0.5046	100	3793.877	3792.867	4820.5	1	8.199	35.2	14
	K.TGGILI	5.5483	0.4212	100	3793.88	3792.867	5476.2	1	6.875	29.7	8
	K.WDEGD	4.6182	0.3966	100	2415.121	2414.109	8354.5	1	6.741	42.1	8
	K.WDEGD	5.0243	0.3075	100	2422.138	2421.126	4269.4	1	6.067	44.7	20
PF13_0141		4883	75.60%	316	34108	7.5	U	Annotation|Plasmodium_falciparum_Sanger|(protein coc			
	Q.KNLGDV	4.6474	0.372	100	1459.846	1459.852	7877.9	1	7.702	79.2	17
	K.NLGDVV	4.9188	0.4362	100	1338.77	1338.775	6953.8	1	8.565	86.4	82
	K.ALDTSH	5.9627	0.6062	100	1811.805	1811.806	8091.9	1	10.451	73.3	36
	K.ALDTSH	4.1016	0.5385	100	1811.809	1811.806	6285.2	1	9.275	60	6
	K.ALDTSH	3.8918	0.3818	100	1811.81	1811.806	8241	1	6.962	48.3	3
	D.TSHTNV	3.9145	0.5287	100	1512.662	1512.658	4334.8	1	9.247	66.7	3
	K.VSGSNT	6.8182	0.5928	100	2408.213	2407.211	8846.9	1	11.342	60.9	1763
	K.VSGSNT	4.0143	0.2996	99.8	2411.185	2407.211	8281.6	1	6.172	29.3	2
	K.APGKSD	5.1808	0.3144	100	2311.185	2310.184	6853.7	1	6.458	55.3	9
	K.SDKEWI	3.8562	0.2976	100	1956.97	1956.978	4871.4	1	5.573	66.7	10
	K.SDKEWI	3.5597	0.3074	99.8	1956.979	1956.978	6268.2	4	6.085	46.7	1
	K.SDKEWI	4.4322	0.2467	99.9	1956.982	1956.978	5703.9	1	5.087	48.3	1
	K.IMIEIGG	2.8229	0.3196	99.9	1138.696	1138.703	5199.4	1	5.84	83.3	2
	K.NCPNAF	5.5543	0.5156	100	2493.319	2493.3	9269.5	1	9.323	54.8	14
	K.NCPNAF	4.299	0.4144	100	2622.379	2621.358	8087.8	1	6.874	45.5	6
	F.IIVVTN	5.0621	0.4073	100	1804.065	1804.059	6415.5	1	9.155	83.3	28
	R.DVNAHI	4.897	0.4515	100	1331.682	1331.682	5854.6	1	8.512	83.3	128
	R.DVNAHI	4.6336	0.3868	100	1338.706	1338.699	4644.8	1	9.075	70.8	22
	K.RYITVG	6.4085	0.4663	100	1983.134	1983.133	6799.8	1	8.989	78.1	43

K.RYITVG	4.3447	0.46	100	1983.144	1983.133	4843.8	1	7.603	46.9	1
R.YITVGG	5.4892	0.3792	100	1808.987	1805.98	4250.9	1	6.886	73.3	136
I.PLQEFIN	3.7693	0.2827	100	1109.613	1109.607	6118.4	1	6.076	87.5	1
K.LISDAEL	3.4063	0.47	100	1505.806	1505.804	3859.1	1	7.335	79.2	3
K.LISDAEL	4.5268	0.4869	100	1507.82	1505.804	7331.6	1	8.126	79.2	116
R.TVNTAL	7.0351	0.4948	100	3399.81	3399.787	9948.6	1	8.454	26.3	57
R.TVNTAL	5.2464	0.5358	100	3424.872	3420.839	6925.5	1	8.393	32.3	318
R.TVNTAL	9.8928	0.5717	100	3426.831	3420.839	11269.7	1	10.989	37.9	714
R.TVNTAL	5.4006	0.2848	100	3759.054	3755.993	6636.6	1	6.235	22.1	2
V.NTALEI	4.679	0.5017	100	3220.722	3220.723	7639.9	1	8.888	32.8	18
N.TALEIV	5.9018	0.5311	100	3106.665	3106.68	9058.5	1	9.191	37.5	3
N.TALEIV	4.2318	0.5065	100	3106.681	3106.68	7556.4	1	8.299	37.5	35
T.ALEIVN	5.0964	0.5609	100	3006.64	3005.632	6705.1	1	9.704	46.3	44
A.LEIVNL	5.1437	0.4981	100	2935.599	2934.595	6536.7	1	9.348	51.9	59
L.EIVNLH	5.0032	0.3941	100	2822.521	2821.511	6854.4	1	8.782	50	50
E.IVNLHA	3.2999	0.3337	100	2693.476	2692.469	5252.2	1	5.585	37.5	1
I.VNLHAS	3.5661	0.241	99.8	2562.387	2558.333	6286.3	1	5.775	39.1	8
V.NLHASP	4.8522	0.3837	100	2476.309	2473.299	7412	1	8.119	68.2	33
L.HASPYV	4.5074	0.4057	100	2246.187	2246.172	10891.3	1	7.204	38.8	4
L.HASPYV	3.924	0.3344	100	2249.186	2246.172	6842.2	1	6.916	55	10
H.ASPYVA	4.1576	0.287	99.9	2095.067	2095.079	9900.8	1	6.072	42.1	6
H.ASPYVA	5.9112	0.5394	100	2095.074	2095.079	5121.1	1	9.488	71.1	87
A.SPYVAP	5.2302	0.2311	100	2038.063	2038.076	6606.1	1	6.766	43.1	14
A.SPYVAP	6.093	0.5338	100	2041.099	2038.076	4430.9	1	9.984	77.8	53
S.PYVAPA	4.9386	0.4703	100	1951.033	1951.044	7754.4	1	8.911	47.1	2
S.PYVAPA	4.8439	0.5234	100	1951.05	1951.044	5496.4	1	8.752	64.7	20
Y.VAPAAA	5.1608	0.4181	100	1693.912	1690.928	7697.7	1	7.843	76.7	40
V.APAAAI	4.552	0.4147	100	1577.828	1577.825	5581.1	1	7.76	82.1	3
A.PAAAII	5.3683	0.5426	100	1520.826	1520.822	7424.8	1	9.948	88.5	83
A.PAAAII	2.9922	0.383	99.8	1520.826	1520.822	4897.2	2	6.321	50	1
K.VLICST	7.226	0.5012	100	4637.384	4634.397	11574	1	8.582	25	485
E.GQYGHS	4.7755	0.4223	100	3585.808	3584.787	7624.4	1	7.924	31.8	17
E.GQYGHS	6.2494	0.3846	100	3599.868	3598.822	9426.2	1	7.256	26.5	8
G.HSDIFG	6.1973	0.4389	100	3193.64	3193.657	11027.4	1	7.391	32.8	13
G.HSDIFG	5.0535	0.5301	100	3194.678	3193.657	5222.8	1	9.024	41.4	16
G.ANGVEC	4.5402	0.3781	100	1899.971	1899.966	5985.3	1	6.772	56.2	18

	G.ANGVE(	5.7171	0.386	100	1910.012	1906.984	9725		1	8.026	59.4	186
	K.AKFDEA	4.0915	0.3348	100	1222.635	1222.632	4475.3		1	7.938	85	17
	K.AKFDEA	4.6774	0.3776	100	1378.726	1378.733	8191.3		1	7.568	86.4	16
	K.AKFDEA	4.2382	0.3071	100	1385.752	1385.75	3200.7		1	6.866	61.4	5
	K.AKFDEA	3.9327	0.4189	100	1385.753	1385.75	4960.9		1	7.429	68.2	2
	K.FDEAIA	3.3393	0.3151	100	1186.62	1186.618	6743.4		1	6.133	94.4	2
PF10_0121		1016	75.30%	231	26362	7.7	U	Annotation|Plasmodium_falciparum_TIGR|(protein codin				
	M.PIPNNP	5.2299	0.3871	100	1983.002	1982.998	7657.8		1	7.425	48.6	2
	M.PIPNNP	4.5412	0.4102	100	1990.021	1990.015	3783.4		1	7.885	61.1	29
	M.PIPNNP	5.3195	0.4999	100	3839.757	3837.738	6303.3		1	8.259	26.5	10
	M.PIPNNP	3.1102	0.3098	99.9	3965.838	3965.833	6499.2		1	6.01	22.9	8
	M.PIPNNP	6.0415	0.5602	100	3971.856	3965.833	5445.5		1	8.495	20.5	35
	M.PIPNNP	5.7783	0.466	100	3980.883	3979.867	5791.5		1	8.045	24.3	15
	M.PIPNNP	5.302	0.4402	100	4108.979	4107.962	6476.4		1	7.298	21.5	6
	M.PIPNNP	4.6942	0.4558	100	4109.983	4107.962	4188.9		1	7.769	20.4	1
	I.PNNPGA	4.0581	0.4873	100	1772.862	1772.861	6949.4		1	8.498	68.8	9
	K.DDDGYI	4.7817	0.4308	100	2010.876	2008.871	7497.6		1	7.979	59.4	16
	T.KVLVPN	2.955	0.3865	99.8	1073.717	1073.716	4652		1	7.412	77.8	2
	T.KVLVPN	3.0173	0.2798	99.9	1073.722	1073.716	4499.6		2	5.863	66.7	2
	K.VLVPNG	2.4106	0.3267	99.8	945.6233	945.621	3191.2		3	5.696	68.8	1
	K.VYNNEE	5.2108	0.3536	100	1798.93	1798.928	11244.7		1	6.772	65.4	176
	K.VYNNEE	3.5783	0.2786	99.8	1798.932	1798.928	3160.4		1	4.764	42.3	3
	V.YNNEEF	3.7105	0.2463	99.9	1702.891	1699.859	7146.8		1	4.801	75	3
	R.IHNYSA	3.3741	0.2528	99.9	1257.645	1255.64	4420.2		9	5.922	70	3
	R.IHNYSA	4.712	0.4305	100	1613.851	1612.845	8806.7		1	7.885	80.8	16
	R.IHNYSA	5.8963	0.5076	100	2347.165	2347.184	6572.2		1	9.732	60.5	8
	R.IHNYSA	4.1191	0.377	100	2354.182	2354.201	5759.8		6	6.34	32.9	1
	Y.SAVETS	4.3308	0.2603	100	1820.94	1819.934	3548.2		1	5.709	63.3	27
	S.AVETSK	4.3487	0.5269	100	1732.909	1732.902	3937.7		1	8.012	67.9	6
	K.PLFGEH	3.4034	0.4123	100	1117.579	1117.58	4597		1	6.894	87.5	87
	K.SYCNDC	5.0248	0.4712	100	2526.164	2526.145	9991.3		1	7.829	39.3	5
	K.SYCNDC	6.0506	0.5337	100	2530.163	2526.145	8786.7		1	9.451	61.9	160
	K.HVLIVEI	4.6354	0.4542	100	1472.865	1472.862	5320.5		1	8.03	87.5	135
	K.HVLIVEI	3.4736	0.432	100	1472.872	1472.862	5898.7		1	8.116	66.7	3
	K.TVAIACI	3.2739	0.377	100	1135.655	1135.655	4291.7		1	6.328	83.3	8
	K.RTPLWN	3.2483	0.3329	100	1118.609	1118.611	5247.5		1	6.301	87.5	15

	K.ADFVGF	5.4957	0.4954	100	2921.43	2921.434	6927.7	1	8.086	37.5	31
	K.ADFVGF	5.0867	0.5949	100	2923.438	2921.434	7957.9	1	9.866	43.8	182
	F.SIPDHF	3.7486	0.4374	100	2286.148	2285.143	6049.1	1	7.529	52.8	1
	R.DLDHCC	3.5085	0.3264	100	1574.658	1574.658	5195.6	1	6.67	70.8	5
	R.DLDHCC	3.7056	0.419	100	1574.659	1574.658	5314	1	6.036	58.3	2
	R.DLDHCC	3.9971	0.4637	100	1702.749	1702.753	7045.4	1	8.019	73.1	3
PF10_0289		1981	74.70%	367	42466	5.7 U	Annotation|Plasmodium_falciparum_TIGR|(protein codin				
	K.DELDID	4.3751	0.4131	100	1460.638	1460.636	7253.4	1	6.958	86.4	6
	K.DELDID	4.145	0.3458	100	1588.735	1588.731	7419.4	1	6.907	87.5	13
	R.KYNIQP	4.4965	0.397	100	2914.474	2913.464	7422.5	1	7.285	45.7	27
	R.KYNIQP	7.5833	0.5311	100	2914.475	2913.464	7305	1	9.215	38	58
	K.YNIQPN	5.4757	0.4749	100	2785.379	2785.369	6932	1	8.929	43.2	19
	N.MSDEEI	5.7913	0.5193	100	2049.013	2049.008	8059.4	1	8.81	68.8	14
	N.MSDEEI	5.2942	0.4657	100	2050.016	2049.008	3797.1	1	8.401	48.4	2
	K.SLDEFV	5.4852	0.4831	100	3173.706	3172.686	10445.5	1	7.617	30.8	1
	R.LTDIYI	6.3414	0.5418	100	1888.018	1885.003	8688.5	1	10.098	83.3	417
	K.YKEGVV	3.7712	0.2639	100	1370.716	1370.714	5004.4	6	5.815	50	12
	K.YKEGVV	4.0789	0.4846	100	1370.718	1370.714	5008.7	1	8.315	90	59
	K.EGVVLM	3.2652	0.3643	100	1079.559	1079.556	4777.4	1	6.796	93.8	27
	S.PSFMSF	2.4213	0.3154	99.9	843.4084	843.4075	3803.3	1	5.668	83.3	3
	K.HNLDKD	5.3403	0.3697	100	1644.909	1644.907	8871.8	1	6.27	44.9	12
	K.HNLDKD	4.6234	0.4389	100	1644.909	1644.907	5672.1	1	6.987	69.2	4
	K.HNLDKD	5.3171	0.4547	100	1647.916	1644.907	9121.1	1	8.533	80.8	67
	K.HNLDKD	5.6654	0.251	100	1659.947	1658.942	7067.2	1	5.734	61.5	24
	K.DLIHEAI	2.9991	0.3203	100	1051.635	1051.634	4920.8	1	5.368	81.2	4
	K.GLNEAV	3.8479	0.1765	99.8	1322.732	1319.721	8889.7	1	5.323	77.3	48
	K.GLNEAV	6.1774	0.3964	100	3120.634	3119.627	8415.1	1	8.037	41.1	3
	K.IQVGLL	3.8086	0.286	99.9	1812.914	1811.907	3264	1	5.476	45.3	11
	K.IQVGLL	5.5781	0.4682	100	1814.919	1811.907	8264	1	9.765	75	49
	K.KDFVGY	4.2835	0.2438	99.9	2203.106	2202.098	8084.1	1	5.095	63.9	3
	K.DFVGYD	4.8913	0.3641	100	2074.005	2074.003	8387.3	1	7.017	61.8	95
	K.DFVGYD	3.9225	0.2942	99.9	2075.006	2074.003	5043.1	1	6.002	42.6	5
	K.DFVGYD	4.8647	0.4095	100	2962.479	2961.472	4671.3	1	6.742	28.5	8
	K.DFVGYD	6.5745	0.4247	100	2962.483	2961.472	6487.3	1	7.261	42.7	22
	K.DFVGYD	4.6705	0.4201	100	2962.486	2961.472	7163.4	1	7.004	41.7	6
	Y.DHAGHE	3.7749	0.3369	100	1494.764	1492.755	7656	1	5.216	79.2	5

	H.AGHEVD	3.2025	0.3062	99.9	1240.672	1240.669		4859	1	6.999	80	2
	K.DIFDNI	6.8213	0.5766	100	4342.326	4341.322		6972	1	9.43	29.6	78
	K.DIFDNI	8.2558	0.4982	100	4342.336	4341.322		7094.8	1	8.629	26.8	134
	R.EEGISL	6.2177	0.5067	100	3454.858	3453.853		7709.4	1	8.599	32.3	239
	L.SVHAGE	6.3551	0.5154	100	2821.542	2818.529		6812	1	8.452	32	4
	S.VHAGED	5.4171	0.5242	100	2719.474	2717.463		7056.3	1	8.652	35.4	2
	L.YTAINLL	2.7154	0.4186	100	1178.702	1178.701		8198.6	1	6.947	77.8	1
	Y.TAINLLH	3.1294	0.3127	100	1008.622	1008.621		6449.5	1	5.782	93.8	4
	R.VSESQE	4.7381	0.3866	100	1366.757	1366.754		9087.6	1	7.464	86.4	8
	R.VSESQE	5.737	0.2994	100	3653.009	3650.993		7343	1	5.696	35.5	6
	K.EKDILL	6.398	0.3571	100	2311.28	2310.274		6764.2	1	7.356	48.7	28
	K.EKDILL	6.5755	0.5136	100	2324.315	2324.309		7072.4	1	8.861	63.2	71
	K.DILLEV	6.8838	0.5214	100	2068.2	2067.171		7564.2	1	9.93	73.5	101
	K.SMDTHF	2.9431	0.2886	100	963.483	963.4796		5126.4	1	5.912	92.9	5
	K.VSVNSD	7.3266	0.5326	100	4064.904	4061.89		4626.3	1	8.919	25.7	220
	K.VSVNSD	5.1567	0.3362	100	4064.91	4061.89		4968.5	1	5.768	20.6	6
	D.PGMFLT	4.4401	0.4008	100	3339.581	3338.575		9025.9	1	5.887	31.5	3
	K.MNLWAY	3.2262	0.2779	100	989.5256	989.5242		5230.8	9	5.631	92.9	44
	K.SFVDPD	2.7438	0.2472	99.8	927.4907	927.4901		3031	46	5.032	78.6	1
PF14_0378		1300	74.20%	248	27935	6.4	U		Annotation|Plasmodium_falciparum_TIGR|(protein codin			
	K.YFVAAN	2.7299	0.3134	99.9	998.5025	998.51		3222.5	2	4.787	85.7	3
	K.SLTNSF	5.5382	0.4901	100	3562.775	3562.761		8719	1	7.586	32.5	87
	K.SLTNSF	4.8703	0.407	100	3562.781	3562.761		7629.3	2	6.422	22.8	6
	K.SLTNSF	4.0503	0.3174	99.9	3562.781	3562.761		6714.3	14	4.91	19.6	1
	K.SLTNSF	3.9215	0.325	100	3562.782	3562.761		8187.8	1	6.72	26.7	34
	K.LDVVVF	4.932	0.4819	100	1882.97	1882.982		5314.2	1	8.525	73.3	146
	F.PVSVHY	3.0445	0.3313	100	1210.6	1210.597		4540.1	1	7.582	83.3	6
	K.FGNGSY	2.9771	0.3461	99.9	1638.786	1636.793		4783.9	1	5.886	56.7	1
	K.DLNIEYV	3.3051	0.2703	99.9	1738.953	1738.943		8519.2	1	5.195	61.5	9
	R.RKYFHE	3.0473	0.284	99.9	1594.771	1594.761		3899.7	1	5.885	72.7	3
	R.KYFHET	3.5806	0.4093	100	1438.665	1438.66		4887	1	6.954	90	3
	R.KYFHET	3.29	0.3628	100	1695.792	1695.798		5871.9	1	5.658	75	3
	R.KYFHET	3.5317	0.2872	99.9	1695.792	1695.798		6331.5	2	5.59	50	2
	K.YFHETD	2.8799	0.446	100	1310.569	1310.565		6015.6	1	6.694	83.3	3
	K.YFHETD	3.8024	0.4447	100	1567.706	1567.703		8595	1	7.737	77.3	7
	K.AVVCFG	3.0085	0.3128	99.9	1394.666	1394.674		7688.3	1	6.072	86.4	3

	K.AFVDLI	7.7506	0.4321	100	2916.568	2915.55		8094.8	1	8.489	37	197
	K.AFVDLI	5.7848	0.3166	100	2919.549	2915.55		8571	1	7.004	50	751
	L.IDNFDN	3.3775	0.3683	100	2370.277	2370.265		5904.9	2	6.275	40	1
	I.LAYEPL	2.7234	0.4217	99.8	1425.798	1425.786		6490.8	1	6.711	54.2	1
	K.TATPEQ	3.0969	0.348	100	1322.709	1322.707		4737.6	2	6.128	68.2	12
	K.TATPEQ	3.6173	0.3958	100	1727.955	1727.952		5406.5	1	6.756	57.1	1
	A.TPEQAQ	3.3698	0.2827	100	1150.622	1150.622		8728.5	1	6.293	83.3	6
	T.PEQAQL	2.8451	0.292	99.9	1049.574	1049.574		5439.5	1	5.941	81.2	3
	R.KIVKDT	5.1471	0.4725	100	1902.055	1902.042		7301.9	1	8.683	73.3	3
	R.ILYGGS	3.9536	0.4349	100	3462.752	3462.736		6549.4	1	7.711	32.3	8
PFL1420w		99	73.30%	116	12844	6.5	U		Annotation|Plasmodium_falciparum_Sanger|(protein coc			
	M.PCCEVI	6.576	0.4251	100	3664.704	3660.72		5268.4	1	7.023	30.5	7
	M.PCCEVI	3.8475	0.4411	100	3681.78	3681.771		6984.3	1	7.096	31.2	11
	M.PCCEVI	4.7098	0.3326	100	4113.012	4111.993		8595.7	1	5.526	22.9	2
	M.PCCEVI	6.9068	0.505	100	5235.398	5233.448		9219.8	1	8.498	21.5	11
	M.PCCEVI	6.9185	0.5921	100	5262.48	5261.517		5416.2	1	9.764	21.7	38
	L.PDDNVQ	2.9844	0.3231	99.9	3954.91	3954.904		6176.1	3	5.65	19.1	3
	L.SQIENA	3.0938	0.3526	99.9	2965.419	2964.412		6238.1	1	5.647	32	2
	R.FGGSNE	3.8728	0.4301	100	1409.63	1406.616		7728	1	7.455	86.4	12
	R.SNNSAL	4.2646	0.4175	100	1268.656	1268.656		8265.5	1	7.202	81.8	7
	R.DCSAQN	2.8614	0.4251	100	1607.672	1607.68		6218.1	2	7.345	46.4	6
PF11_0098		1103	67.60%	343	39374	4.7	U		Annotation|Plasmodium_falciparum_TIGR|(protein codin			
	K.LFHLID	4.6094	0.4692	100	3178.632	3177.629		7575.5	1	8.567	38	21
	K.LFHLID	7.3493	0.5358	100	3179.647	3177.629		9737.4	1	9.513	43	34
	H.LIDKNN	4.8773	0.4033	100	2780.422	2780.418		7863.4	1	8.15	54.5	30
	H.LIDKNN	5.2222	0.3132	100	2780.425	2780.418		9018.3	1	6.698	35.2	4
	K.NNDKEI	4.3563	0.2372	99.8	2304.117	2304.111		6868.3	1	4.817	37.5	1
	K.NNDKEI	5.7324	0.3951	100	2307.112	2304.111		8083.2	1	8.235	69.4	87
	K.EITEEE	4.4173	0.3817	100	1827.905	1825.886		3828.1	1	6.662	75	10
	K.EITEEE	2.8869	0.3972	99.8	1832.918	1832.903		3494.4	1	6.251	60.7	1
	K.QVQAEN	3.5395	0.3975	100	3625.737	3624.713		8844	1	7.676	26.6	20
	K.QVQAEN	7.2236	0.5384	100	3639.759	3638.747		14243.2	1	10.23	29.7	48
	Q.VQAEM(	5.7298	0.4974	100	3511.712	3510.689		8920.5	1	8.201	31.5	1
	Q.AEMGQ	6.3989	0.4876	100	3284.574	3283.562		10867.2	1	8.427	37.1	9
	K.DGFISL	5.4338	0.4866	100	2195.068	2195.062		9179.4	1	9.152	60.5	17
	K.RFQIVD	3.4648	0.3957	100	1455.804	1455.803		6563.7	1	6.09	50	2

K.RFQIVD	4.1188	0.2659	100	1455.804	1455.803	6621.6	1	5.72	81.8	19
R.FQIVDK	3.8684	0.2393	100	1295.696	1292.685	5393.9	2	5.282	85	12
K.LSINEV	4.9287	0.4507	100	2170.193	2170.187	5556.1	1	7.448	50	43
K.LSINEV	4.3242	0.369	100	2171.197	2170.187	5102.1	1	6.304	36.1	7
K.LSINEV	6.162	0.5218	100	3971.064	3969.053	6438.4	1	8.338	27.3	25
K.LSINEV	6.8654	0.3846	100	3999.143	3997.122	7040.3	1	6.622	23.2	18
K.LSINEV	7.4052	0.5488	100	4298.28	4297.265	7878.3	1	7.699	25	19
K.LSINEV	4.2731	0.3382	99.9	4298.281	4297.265	5767.9	5	5.491	17.7	1
K.LSINEV	6.0149	0.4309	100	4298.281	4297.265	7013.9	1	7.118	25.7	17
S.INEVGL	6.3992	0.3968	100	3769.949	3768.937	7351.1	1	6.59	22	6
S.INEVGL	5.6496	0.2898	100	3770.956	3768.937	9242.8	1	6.55	30.6	2
S.INEVGL	5.1797	0.2645	100	4071.097	4069.08	8647.8	1	5.29	22.1	2
S.INEVGL	5.8135	0.3156	100	4097.158	4097.149	8826.3	1	6.504	22.1	3
G.LLIDPM	4.8694	0.3405	100	3278.741	3277.729	9374.6	1	5.941	28.8	1
K.ELEINE	5.0299	0.407	100	1831.923	1831.919	5346.3	1	7.317	64.3	6
K.ELEINE	5.096	0.3679	100	2133.07	2132.063	3772.9	1	6.803	55.9	2
R.SDESSC	4.313	0.4824	100	2825.233	2824.226	9151.6	1	7.466	41.7	12
R.SDESSC	6.343	0.4206	100	2827.241	2824.226	8829.5	1	7.733	39.6	29
R.SDESSC	6.5319	0.3676	100	4113.915	4111.897	8337.1	1	6.947	23.8	10
R.SDESSC	5.2516	0.2726	100	4133.955	4132.949	8919.9	2	4.873	21.1	1
K.KDDEMA	5.2785	0.3951	100	2035.882	2034.876	9886.9	1	8.224	51.6	14
K.KDDEMA	6.8389	0.3716	100	2037.886	2034.876	8507.5	1	8.486	84.4	90
K.KDDEMA	7.3649	0.4986	100	3344.61	3343.598	8364.1	1	8.64	38.9	26
K.KDDEMA	8.081	0.5305	100	3346.615	3343.598	8086.3	1	8.525	34.6	32
K.DDEMAL	5.7074	0.4506	100	1907.79	1906.781	8011.5	1	8.04	80	42
K.DDEMAL	6.1899	0.4651	100	3218.52	3215.503	6569.7	1	8.182	36.5	47
K.DGFIDKI	3.4884	0.2848	100	1328.745	1327.741	6336.1	1	6.181	80	7
I.KVYFDP	5.6133	0.3822	100	2145.123	2145.117	6226.6	1	7.631	47.2	8
I.KVYFDP	5.6558	0.4682	100	2145.124	2145.117	4901.7	1	9.249	75	13
I.KVYFDP	6.2114	0.295	100	2950.486	2948.48	7891.4	1	6.147	35	4
K.VYFDPA	5.23	0.2555	100	2004.996	2002.987	3978.3	1	5.023	57.4	4
K.VYFDPA	5.7997	0.5513	100	2020.036	2017.022	8007.8	1	8.643	73.5	27
K.VYFDPA	4.5739	0.4349	100	2635.276	2635.268	8180.2	1	7.963	54.5	11
K.VYFDPA	5.6311	0.4365	100	2821.388	2820.385	5264.7	1	7.801	47.9	36
K.VYFDPA	7.1604	0.5085	100	2841.442	2841.436	4701.9	1	8.993	37.5	38
K.VYFDPA	4.9786	0.4123	100	2949.482	2948.48	4791.6	1	7.397	48	5

	K.VYFDPA	6.7464	0.5312	100	2970.543	2969.531		7251.9	1	9.312	36	26	
	K.KITYDL	3.9304	0.3256	100	1318.701	1316.696		4946.5	1	6.126	88.9	37	
	K.ITYDLW	3.3759	0.3281	100	1190.612	1188.601		3883.9	1	5.667	81.2	13	
	K.IAVTSL	3.9548	0.5063	100	1536.849	1536.846		3750.1	1	8.319	61.5	8	
	K.IAVTSL	5.3866	0.5487	100	1536.849	1536.846		7172.8	1	9.408	84.6	47	
	K.IAVTSL	3.938	0.3722	100	2317.202	2316.195		5803.2	1	6.225	38.2	1	
	K.IAVTSL	5.2595	0.5175	100	2319.204	2316.195		6250.1	1	8.462	55.3	34	
	T.SLTDYG	3.4944	0.3696	100	1917.928	1917.924		5657	1	6.608	63.3	3	
	R.AFEDD	5.679	0.3	100	2934.105	2933.103		7254.7	1	5.812	43	6	
	R.AFEDD	4.3924	0.2626	100	2934.107	2933.103		8897.2	1	6.632	44	3	
	R.AFEDDD	5.3644	0.4507	100	3667.494	3665.48		8730.6	1	7.368	28.1	1	
PF11_0208		305	67.20%	250	28770	8.3	U		Annotation|Plasmodium_falciparum_TIGR|(protein codin				
	R.HGESTV	3.7923	0.3886	100	1329.617	1329.619		4664.5	2	7.181	80	15	
	K.FTGWTD	3.3862	0.3323	100	1379.679	1379.685		7341.3	1	5.91	72.7	5	
	K.FTGWTD	4.9808	0.4462	100	2342.157	2342.163		4686.9	1	8.028	52.4	7	
	R.AICTAW	2.884	0.3518	100	1182.643	1182.642		5211	1	6.61	72.2	1	
	K.TADLLH	3.5294	0.4577	100	1191.71	1191.71		6191.5	1	7.449	80	12	
	R.RSYDIP	3.8598	0.1907	99.9	1435.794	1435.802		6444.3	1	5.423	81.8	7	
	K.EDNRW	3.7361	0.3508	100	1613.777	1613.783		5332.7	1	6.429	62.5	54	
	K.DALPFT	3.8093	0.4608	100	1793.877	1793.874		6017.2	1	7.365	64.3	6	
	R.VLPFWF	5.292	0.4482	100	2010.109	2010.104		8044.5	1	7.461	65.6	41	
	L.PFWFDH	4.4994	0.4348	100	1797.952	1797.952		6757.9	1	7.988	71.4	5	
	K.VMVAAH	2.4907	0.3555	99.9	1154.612	1154.611		7421.2	1	6.314	80	2	
	V.KHLDNL	6.3088	0.4029	100	3844.104	3843.098		10063.9	1	7.456	31.1	10	
	K.HLDNLS	4.0176	0.36	100	3700.975	3700.969		6046.5	1	6.271	25	16	
	K.HLDNLS	8.4129	0.4977	100	3706.966	3700.969		7759.9	1	8.56	34.4	93	
	E.LNIPTG	4.4979	0.4548	100	2365.341	2365.338		4008.1	1	7.413	55	10	
	K.HYYLLD	3.2573	0.417	100	1409.699	1409.695		6744	1	6.994	85	3	
	K.HYYLLD	4.9043	0.4252	100	1537.783	1537.79		7718.5	1	7.519	61.4	6	
	K.HYYLLD	4.5309	0.3844	100	1537.784	1537.79		6665.1	1	8.081	90.9	12	
MAL13P1.237		677	65.30%	372	42160	7.5	U		Annotation|Plasmodium_falciparum_Sanger|(protein coc				
	R.YIILSG	4.0794	0.3974	100	2618.45	2617.446		4828.1	1	6.741	45.7	5	
	L.SGLGDA	4.3758	0.4466	100	2102.1	2101.096		4554.2	1	7.196	57.9	4	
	L.PLCVQL	4.0775	0.294	100	1488.794	1487.789		8001.7	1	5.573	83.3	3	
	K.IETSYS	3.5071	0.479	100	2242.048	2241.047		5574.8	1	7.208	47.2	3	
	R.ISPGYV	2.7891	0.3087	99.9	1263.641	1263.637		2288.9	2	5.666	80	27	

	I.SPGYVS	3.1975	0.2102	99.8	1150.555	1150.553	5790.3	2	5.499	77.8	5
	S.PGYVSF	3.309	0.2885	100	1065.529	1063.521	5855.7	1	5.902	87.5	16
	K.TDSFTP	4.3736	0.2733	99.8	6295.923	6295.914	8723.9	16	4.577	12	1
	K.TDSFTP	6.7433	0.5701	100	6299.927	6295.914	4870.8	1	8.703	14.8	41
	L.NAGDNN	4.1018	0.3043	99.9	4024.934	4022.912	5892	2	5.277	18.1	1
	K.AVNENC	4.1083	0.3376	100	1557.742	1557.74	7573.8	1	6.515	73.1	44
	K.SVLYDN	2.962	0.2924	99.9	1180.622	1180.621	6742.6	1	5.932	77.8	3
	K.SVLYDN	3.7643	0.4024	100	1782.931	1782.928	6999.5	1	7.549	57.1	3
	K.GSDHST	5.4809	0.4777	100	1588.812	1588.809	4506.8	1	8.861	71.4	30
	K.GSDHST	3.8029	0.5352	100	1588.813	1588.809	6920.5	1	8.903	64.3	6
	K.GSDHST	5.2363	0.4182	100	1716.907	1716.903	6188.8	1	9.071	70	1
	K.NMGMV	3.5528	0.3145	100	1196.61	1194.602	6073.2	1	5.864	85	33
	K.GKNYNS	5.7849	0.4416	100	3824.784	3823.768	8660.2	1	7.397	26	10
	K.GKNYNS	5.5789	0.4334	100	3825.784	3823.768	10476.1	1	8.031	33.6	35
	K.GKNYNS	4.6404	0.4378	100	3826.772	3823.768	9182	1	7.996	34.4	12
	K.GKNYNS	5.1251	0.2384	99.9	3981.887	3979.869	7441.9	11	4.88	19.7	3
	K.GKNYNS	5.4161	0.418	100	3981.896	3979.869	10934.5	1	7.28	23.5	3
	K.NYNSVE	3.9787	0.3921	100	2691.231	2689.219	6769.9	1	6.575	36.4	3
	K.NYNSVE	5.8369	0.4477	100	2692.233	2689.219	8714.9	1	9.002	54.5	33
	K.NYNSVE	4.7808	0.5043	100	3638.66	3638.652	9878.6	1	8.926	31.7	103
	K.NYNSVE	7.4934	0.5847	100	3638.667	3638.652	10554.8	1	10.276	35.8	76
	K.NYNSVE	7.2423	0.5062	100	3640.668	3638.652	9105.7	1	8.89	32.2	27
	K.NYNSVE	5.7066	0.4035	100	3796.771	3794.753	7561.9	1	6.679	37.1	15
	K.NYNSVE	4.9485	0.2569	99.9	3796.773	3794.753	8257.2	1	5.626	28.5	3
	N.YNSVEE	4.6648	0.4516	100	3524.62	3524.609	10219.8	1	7.12	29.3	3
	N.SVEEFL	4.9448	0.5453	100	3248.522	3247.503	10301.9	1	9.984	42.6	6
	N.SVEEFL	4.4043	0.3267	100	3249.508	3247.503	7592.9	1	5.848	32.4	5
	E.VQETAE	3.4877	0.2915	99.9	2300.104	2299.095	5490.4	1	5.377	39.5	3
	V.QETAEN	3.9417	0.4215	100	2200.027	2200.027	5013.1	1	6.415	47.2	9
	R.ICLFSGS	3.5291	0.3383	100	1185.655	1185.653	3616.7	1	6.842	88.9	16
	K.AILNGL	3.9621	0.4596	100	1156.691	1153.681	7669.9	1	8.141	95	57
	R.LNFAYD	4.082	0.3684	100	1359.662	1359.658	5759.5	1	6.966	90	27
	K.DAWVET	2.7954	0.3081	99.9	1218.604	1218.601	3204.4	40	5.726	75	2
PF11_0043		160	65.30%	118	13014	4.6	U	Annotation|Plasmodium_falciparum_TIGR|(protein codin			
	A.ALILHEE	2.6909	0.265	99.8	959.5656	959.5639	5870.7	1	5.747	92.9	1
	K.NSNNTV	4.7716	0.4293	100	1908.975	1907.969	4611.3	1	7.58	60	52

	K.GKDIQS	6.8323	0.4815	100	3342.772	3340.76	10101.4	1	8.1	32.8	3
	K.DIQSLL	4.9029	0.4264	100	3163.669	3162.661	6625.5	1	6.549	25.8	1
	S.VGSAPA	4.9266	0.5349	100	2085.082	2085.083	8772.2	1	9.585	52.5	18
	V.GSAPAA	4.3277	0.4363	100	1989.025	1986.014	7745.9	1	8.198	44.7	6
	G.SAPAAA	4.4553	0.5129	100	1928.997	1928.993	7349.9	1	9.035	47.2	12
	S.APAAAA	3.2228	0.3826	99.9	1841.963	1841.961	5643.2	1	5.634	35.3	3
	S.APAAAA	4.7634	0.4855	100	1842.97	1841.961	7546	1	8.231	58.8	6
	A.PAAAAQ	3.8559	0.4583	100	1770.928	1770.924	5813.7	1	8.069	59.4	3
	A.AAQVTT	3.548	0.3489	100	1531.803	1531.797	6654.2	1	6.36	69.2	3
	K.KEEKVE	4.4303	0.4488	100	2359.061	2358.051	7462.2	1	7.373	55.3	44
	K.VEEEEE	3.1725	0.3555	99.9	1846.789	1843.776	3396	3	5.973	53.3	8
PFI1105w		608	64.70%	416	45427	7.8	U	Annotation|Plasmodium_falciparum_Sanger|(protein coc			
	V.RVDFNV	4.7952	0.3984	100	1634.959	1634.953	8169.7	1	8.144	73.1	7
	R.VDFNVF	3.6526	0.2117	99.8	1457.797	1457.8	3419.7	1	5.69	83.3	2
	R.ITATLPT	3.2579	0.2612	99.9	1322.789	1321.784	3468.6	4	5.455	68.2	8
	K.YTLKPV	3.3269	0.4042	100	1262.737	1262.736	5673.8	2	6.253	55	1
	K.YTLKPV	3.5567	0.3537	100	1262.738	1262.736	5021.2	1	6.264	80	9
	K.GLLGEE	5.7651	0.4423	100	1762.895	1762.905	7144.5	1	9.354	73.3	58
	K.GLLGEE	3.9028	0.4192	100	1762.916	1762.905	5066.4	1	8.891	56.7	17
	K.GLLGEE	4.6897	0.4217	100	2363.173	2363.18	8231.8	1	7.357	47.5	36
	G.EEVLFL	5.0982	0.3819	100	2022.976	2022.969	9634.9	1	7.192	78.1	3
	K.ENSVILL	3.4947	0.2126	99.9	1306.749	1306.744	5369.7	1	5.276	80	2
	R.FHIEEE	4.321	0.4237	100	1743.833	1743.83	5760.9	1	8.25	63.3	2
	R.FHIEEE	5.486	0.468	100	1750.85	1750.847	9538.8	1	9.118	83.3	7
	T.KLADVF	5.0893	0.3595	100	1774.912	1774.924	8421.7	1	8.011	48.3	7
	T.KLADVF	4.5084	0.5077	100	1774.917	1774.924	8394.7	1	8.599	80	8
	K.LADVFI	4.6093	0.4977	100	1646.83	1646.829	7391.3	1	9.74	75	104
	K.LADVFI	3.5861	0.3036	99.9	1654.852	1653.846	3843	15	5.489	39.3	1
	K.ALENPQ	2.9382	0.327	99.9	1150.66	1150.658	6024.9	2	6.855	83.3	3
	K.ALENPQ	4.0047	0.4412	100	1761.027	1761.039	5148.9	1	7.475	43.8	4
	K.ALENPQ	4.7422	0.469	100	1768.058	1768.056	8412.9	1	7.597	68.8	25
	L.ENPQRF	3.4445	0.4416	100	1583.942	1583.935	6946.1	1	6.524	53.6	1
	K.VSDKIQI	3.2943	0.3517	100	1057.675	1057.681	5342.4	1	5.981	81.2	1
	K.IGTSLFD	3.2945	0.4262	100	1231.625	1231.628	5629.7	1	6.708	77.3	8
	K.NVQIFLF	3.6635	0.2975	100	1326.757	1326.754	7578.8	1	6.857	75	20
	K.IADNFD	3.7192	0.4435	100	1343.639	1343.631	7647	1	7.815	86.4	14

	K.FVTDEE	5.8118	0.4756	100	2199.026	2198.019		3241.6	1	8.645	60.5	34
	K.SIENYKI	4.1558	0.4279	100	1509.82	1509.816		9911.4	1	7.998	83.3	4
	K.TVIWNG	5.4265	0.383	100	2045.04	2042.028		10424.8	1	8.018	64.7	62
	K.GSIECL	3.5419	0.2373	99.8	1561.812	1560.831		7507.3	1	5.27	69.2	6
	K.KGAITI	6.1179	0.5054	100	2087.095	2086.115		8586.8	1	9.062	75	19
	K.KGAITI	6.1878	0.5207	100	2228.232	2228.244		9018.4	1	9.741	71.4	27
	K.KGAITI	7.1472	0.5844	100	2228.243	2228.244		8763.7	1	9.583	48.8	32
	K.GAITIV	4.4246	0.491	100	1958.024	1958.019		7366.3	1	9.536	55.3	11
	K.GAITIV	5.9159	0.5422	100	1972.05	1972.054		8767.8	1	9.096	65.8	13
	K.GAITIV	4.7034	0.1514	99.9	2086.103	2086.115		8702.3	1	6.563	52.5	9
	K.KNEISH	5.7057	0.4794	100	2126.106	2126.109		7061.1	1	9.347	65	24
	K.KNEISH	4.8458	0.2989	100	2133.125	2133.127		8274.2	1	6.902	38.8	11
	K.NEISHV	4.0132	0.3463	100	2006.04	2005.032		7315.3	1	6.368	52.6	8
PFE0545c		568	64.30%	171	19979	4.7	U	Annotation|Plasmodium_falciparum_Sanger|(protein coc				
	K.VFKDVF	5.1063	0.4696	100	3397.546	3396.537		8109.2	1	6.867	30.6	16
	K.DVFTND	4.8784	0.4901	100	3025.332	3022.305		8997.7	1	7.791	43.8	105
	K.DVFTND	4.1931	0.4168	100	3026.323	3022.305		6389.3	1	6.48	34.4	17
	D.PFEVPE	2.9222	0.2913	99.9	1020.516	1020.515		4345.6	3	5.377	78.6	3
	R.IKGNED	3.7566	0.3346	99.8	3995.899	3993.893		6502	1	4.878	22.9	1
	R.IKGNED	5.7283	0.4846	100	4402.082	4400.078		7240.1	1	7.923	26.9	2
	R.IKGNED	5.1399	0.3277	100	4590.148	4587.136		6807.3	1	6.029	22.6	28
	R.IKGNED	4.5366	0.2753	99.9	4618.214	4615.205		8829.4	1	4.919	15.8	2
	R.IKGNED	6.4695	0.5771	100	4618.221	4615.205		9727.2	1	9.455	28.6	110
	R.IKGNED	6.6338	0.496	100	4743.302	4743.3		8720	1	8.444	20.2	8
	K.GNEDY(	6.9115	0.5449	100	4369.019	4367.008		8228.4	1	9.275	25	121
	K.YLEEKK	4.7292	0.3378	100	1800.999	1800.997		7186.8	1	5.972	73.1	1
	K.KPDRVE	3.4519	0.3619	100	1138.669	1138.67		7098	1	6.121	87.5	15
	Y.SYYKGE	3.2722	0.425	100	1349.682	1349.682		4797.5	1	7.066	75	2
	R.FVYISD	3.8057	0.3427	100	1455.739	1453.733		6438	1	7.222	81.8	8
	R.FVYISD	5.1079	0.456	100	1610.78	1609.779		8237.7	1	8.411	87.5	129
PF10_0155		2528	62.60%	446	48678	6.6	U	Annotation|Plasmodium_falciparum_TIGR|(protein codin				
	R.GNPTVE	5.0432	0.499	100	1880.974	1880.983		5877	1	9.049	71.9	386
	K.NINEIIA	3.0304	0.2593	99.9	1011.584	1011.584		3921.8	1	4.998	93.8	3
	K.KIDNLM	4.1128	0.3702	100	1590.809	1590.805		5591.7	1	6.321	61.5	6
	K.KIDNLM	4.9519	0.3813	100	1597.826	1597.822		8433.8	1	8.469	84.6	6
	K.KIDNLM	5.6964	0.4627	100	2485.23	2485.215		6601.2	1	8.396	62.5	40

K.KIDNLM	4.0862	0.3124	99.9	2487.221	2485.215	8704.2	1	4.96	38.8	1
K.IDNLMV	4.3012	0.3306	100	2357.112	2357.12	7805.6	1	6.278	50	16
K.YLAQLA	2.717	0.2771	99.8	991.5955	991.5941	4982.9	1	4.681	87.5	1
K.KSDQMV	4.1194	0.3361	99.9	2542.287	2542.298	7908	3	6.523	29.3	1
K.KSDQMV	4.4297	0.4304	100	2542.317	2542.298	7051.2	1	6.369	45.7	9
K.SDQMVI	3.5084	0.317	100	2415.21	2414.203	7143.9	1	4.87	40.9	4
C.LNVING	3.4509	0.2595	99.9	1281.68	1280.671	6973.8	1	5.313	75	2
K.LSFQEF	4.2642	0.3562	100	1896.999	1896.993	5392.4	1	6.034	62.5	12
K.LSFQEF	5.2918	0.4903	100	1907.022	1904.011	5844.6	1	8.407	68.8	107
S.FQEFMI	3.233	0.3478	99.8	1696.88	1696.877	5778.5	1	7.11	57.1	1
S.FQEFMI	4.7774	0.2961	100	1696.883	1696.877	5895.3	1	7.543	75	18
E.FMIVPV	2.9586	0.3892	99.8	1292.709	1292.708	4898.9	1	6.734	72.7	2
E.FMIVPV	3.8772	0.3665	100	1301.732	1299.725	4003	1	6.946	72.7	11
F.MIVPVG	2.8425	0.3839	100	1145.64	1145.639	3234.9	48	5.682	65	2
R.YGAEVY	3.6079	0.3197	100	1180.602	1180.6	7286.4	1	6.05	88.9	28
K.KYGIDA	3.8936	0.5424	100	3538.882	3537.874	5343.7	1	8.841	34.8	5
K.KYGIDA	5.4942	0.3208	100	3538.884	3537.874	7628.3	1	6.147	29.5	20
K.YGIDAT	5.1819	0.5331	100	3410.782	3409.779	4863	1	9.444	37.5	191
K.YGIDAT	7.1002	0.5283	100	3413.8	3409.779	7922.6	1	9.614	35.9	44
A.PNILNA	4.6258	0.4844	100	1936.136	1936.131	5728.4	1	8.851	64.7	9
N.ILNANEA	5.6297	0.541	100	1726.042	1725.035	9011.7	1	10.139	83.3	64
N.ANEALD	3.5945	0.4538	100	1377.81	1377.807	8362.6	1	7.772	70.8	4
K.IAMDVA	5.0128	0.4688	100	1788.824	1788.811	8550.9	1	7.765	70	22
V.KTGAQL	5.294	0.3994	100	1682.983	1682.981	9245.3	1	7.744	78.6	28
K.TGAQLV	3.8977	0.2757	99.8	1547.874	1547.869	11028.6	1	6.238	65.4	4
K.TGAQLV	5.4886	0.303	100	1557.896	1554.886	6485.2	1	7.097	80.8	170
K.TGAQLV	4.3551	0.434	100	1675.967	1675.964	5837.7	1	6.988	50	2
K.TGAQLV	4.6351	0.3297	100	1682.983	1682.981	4830	1	6.59	71.4	12
K.KYPIVS	7.933	0.4682	100	2587.231	2586.223	9242.3	1	8.951	47.5	26
K.KYPIVS	5.8573	0.4317	100	2589.236	2586.223	5667.1	1	8.705	55	108
K.YPIVSI	5.8786	0.413	100	2461.172	2458.128	6103.3	1	8.354	63.2	143
K.DVQIVG	5.0428	0.4606	100	1761.95	1761.946	8542.6	1	8.338	70	30
K.DVQIVG	4.066	0.4269	100	1763.956	1761.946	4582.1	1	6.661	56.7	12
K.NACNAL	2.8296	0.343	100	1016.559	1016.556	4923.5	1	5.677	87.5	2
L.KVNQIG	3.6972	0.3956	100	1967.069	1967.078	8857.7	1	6.871	52.9	2
K.VNQIGS	4.1525	0.2889	99.9	2095.141	2095.137	5795.6	2	5.202	36.1	4

	K.VNQIGS	6.0756	0.5315	100	2097.145	2095.137		9852.3	1	9.932	72.2	67
	K.NNWGV	2.9186	0.3531	100	1199.574	1199.574		4949.2	1	6.061	72.2	12
	R.SGETED	5.4586	0.4872	100	1841.983	1840.977		8724.8	1	8.849	75	880
	L.RIEESL	4.7987	0.4678	100	1733.883	1733.882		4085.5	1	8.657	63.3	3
	L.RIEESL	4.5837	0.4057	100	1740.904	1740.899		7989.2	1	8.135	83.3	6
	R.IEESLG	3.2056	0.3167	99.9	1577.786	1577.781		6596.3	1	5.278	60.7	2
PFI0875w		1933	61.80%	652	72388	5.3	U		Annotation|Plasmodium_falciparum_Sanger|(protein coc			
	R.ITPSYV	3.5868	0.4345	100	1471.736	1469.728		3504.4	1	6.449	79.2	8
	R.ITPSYV	2.8966	0.3384	99.9	1597.826	1597.823		4750.6	1	5.258	61.5	2
	K.LEATLH	3.4213	0.4273	100	1698.909	1698.907		5227.8	1	7.452	60.7	1
	K.LEATLH	3.6395	0.4124	100	1698.909	1698.907		6173.2	1	6.853	67.9	12
	E.ATLHPT	3.5705	0.3859	100	1456.78	1456.78		4326.1	1	7.518	75	13
	E.ATLHPT	2.8684	0.3271	99.9	1612.893	1612.881		5809.7	1	5.137	57.7	3
	I.GRKFDD	3.4237	0.3272	100	1591.814	1591.819		5436.9	1	6.116	70.8	3
	G.RKFDDQ	3.3312	0.229	99.9	1263.672	1263.67		7942.9	1	5	88.9	6
	G.RKFDDQ	4.1655	0.3744	100	1534.799	1534.798		7464.1	1	6.656	77.3	6
	R.KFDDQE	3.2137	0.2009	99.8	1107.57	1107.569		5921.9	6	5.921	81.2	1
	R.KFDDQE	3.951	0.314	100	1378.699	1378.697		6248.2	1	6.617	85	17
	K.FDDQEV	3.0563	0.2416	99.8	1250.599	1250.602		6465.2	1	5.364	77.8	6
	R.SLLPYE	4.8464	0.4442	100	1941.102	1941.1		6296.6	1	7.6	53.1	9
	R.SLLPYE	5.6566	0.4836	100	1941.103	1941.1		5818.3	1	9.011	62.5	99
	L.LPYEIV	4.1499	0.3692	100	1726.95	1726.949		6917.8	1	6.529	71.4	2
	L.PYEIVN	4.9022	0.4343	100	1627.901	1627.899		8608.1	1	7.124	76.9	29
	K.DKDTTF	4.4989	0.289	100	2024.013	2023.006		5542.7	1	5.706	44.1	2
	K.DKDTTF	5.8614	0.4748	100	2031.031	2030.023		5073.1	1	7.904	76.5	47
	K.DTTFAP	3.3973	0.3338	100	1787.904	1786.901		5742.5	1	5.65	56.7	1
	K.EIAQSF	3.6452	0.3447	100	1316.759	1316.758		6039.6	1	6.106	86.4	24
	K.NAVVTV	3.3319	0.424	100	1664.836	1664.84		3568.3	1	7.099	71.4	3
	T.VPAYFN	3.5628	0.4036	100	1181.58	1180.575		4808.1	1	7.211	88.9	3
	R.IINEPT	5.407	0.5558	100	1673.93	1673.93		5046.3	1	9.631	80	4
	R.IINEPT	4.6837	0.4101	100	1802.028	1802.025		4888.6	1	7.649	71.9	4
	T.AGNTHL	2.8581	0.3619	99.9	1517.68	1516.678		5596.7	1	5.875	61.5	3
	R.NLSVVH	7.1415	0.4844	100	3069.548	3067.534		6063.5	1	7.613	38.5	43
	R.NLSVVH	5.5065	0.5237	100	3088.588	3088.585		7019.9	1	9.276	44.2	23
	S.VVHSTQ	5.1823	0.4657	100	2753.343	2753.375		7396.1	1	7.025	31.5	11
	S.VVHSTQ	6.0071	0.4751	100	2754.356	2753.375		6974.3	1	9.175	52.2	30

H.STQIEI	5.8578	0.533	100	2439.235	2439.23	7249.2	1	10.106	57.5	32
R.AKFEEL	3.5715	0.4112	100	1496.74	1496.739	3760.3	1	7.729	68.2	3
R.AKFEEL	4.7412	0.3303	100	1496.741	1496.739	8438.5	1	6.996	65.9	11
R.AKFEEL	3.856	0.2362	99.9	1499.751	1496.739	6664.9	1	5.751	81.8	36
K.FEELND	3.3955	0.2058	99.9	1297.611	1297.606	5582.3	1	4.662	88.9	11
K.SKIDEIV	5.3749	0.5456	100	1488.867	1487.862	8092.8	1	9.719	88.5	52
K.SKIDEIV	4.1695	0.4667	100	1488.867	1487.862	4675.8	1	8.478	69.2	15
K.EFFNGK	2.8147	0.3687	100	1237.598	1237.597	5441.9	1	5.925	66.7	3
G.EELQDV	5.9411	0.3996	100	3259.85	3258.842	5904.7	1	6.746	27.6	24
G.EELQDV	7.345	0.543	100	3262.857	3258.842	9906.5	1	9.864	33.6	252
G.EELQDV	5.7111	0.4346	100	3262.858	3258.842	5592.4	1	8.377	34.5	199
Q.DVVLLD	6.3137	0.5816	100	2759.621	2759.615	7833.6	1	9.933	39	37
Q.DVVLLD	6.3078	0.4961	100	2761.626	2759.615	7933.7	1	10.171	56	32
D.VVLLDV	6.3864	0.5176	100	2644.594	2644.587	7856.3	1	9.727	41.7	31
D.VVLLDV	5.592	0.5723	100	2645.601	2644.587	6863.6	1	10.219	60.4	8
V.VLLDVT	3.9612	0.4067	100	2545.522	2545.519	7233.8	1	7.305	30.4	1
V.LLDVTP	4.7686	0.4036	100	2425.406	2425.399	7591	1	6.39	37.5	2
V.LLDVTP	3.6196	0.3515	100	2447.432	2446.451	6649.5	1	6.91	38.6	6
L.LDVTPL	5.3313	0.3605	100	2313.32	2312.315	8079	1	7.272	35.7	13
L.LDVTPL	4.5633	0.4919	100	2336.379	2333.367	8791.6	1	8.024	54.8	13
L.DVTPLT	4.3418	0.3057	100	2200.24	2199.231	6545.9	4	5.636	31.2	2
L.DVTPLT	5.4315	0.5135	100	2220.29	2220.283	7453.3	1	8.837	70	20
D.VTPLTL	3.8848	0.496	100	2084.209	2084.204	8297.9	1	8.32	60.5	2
L.TLGIET	5.3987	0.4919	100	1695.006	1695.003	6131.4	1	9.085	73.3	17
L.GIETVG	3.8105	0.2514	99.9	1459.821	1459.82	5821.2	1	5.34	73.1	4
E.TVGGIM	3.2745	0.4513	100	1174.709	1174.706	4014	1	7.425	85	2
K.KSQTFS	5.7303	0.3343	100	2786.388	2785.38	11198.4	1	7.108	38	25
K.KSQTFS	5.4262	0.4538	100	2786.391	2785.38	7024.8	1	8.595	52.2	4
K.SQTFST	3.2002	0.3119	99.9	1940.946	1939.94	5350.1	242	5.289	37.5	2
K.SQTFST	5.5755	0.5262	100	2665.313	2664.302	7299.8	1	8.912	50	128
K.SQTFST	5.8443	0.4234	100	2668.321	2664.302	8559	1	7.369	44.3	36
T.FSTYQD	4.1733	0.3838	100	2349.159	2348.164	8075.2	1	6.651	52.6	7
F.STYQDN	4.413	0.3605	100	2202.103	2201.095	6893.9	1	6.606	55.6	6
R.ALTKDN	3.1489	0.3211	99.9	1209.696	1209.696	7482.6	1	6.152	80	6
K.DNHLLG	5.3616	0.4846	100	2000.091	1999.084	7230.1	1	9.554	73.5	3
L.GKFELS	3.4888	0.4248	100	1406.79	1406.787	4700.8	1	6.527	75	7

G.KFELSG	4.225	0.3049	100	1350.771	1349.766	4501.6	1	5.341	86.4	4
K.FELSGI	3.757	0.3398	100	1221.673	1221.671	2911.2	1	6.985	75	17
K.NGILHV	3.4855	0.3856	100	1231.641	1231.64	6039.6	1	6.205	80	11
K.NGILHV	3.5991	0.4251	100	1574.827	1574.825	4941.2	1	6.999	42.9	4
K.NGILHV	4.8734	0.475	100	1575.83	1574.825	6948.8	1	7.86	67.9	20
R.GITITND	2.8436	0.3681	100	1089.629	1088.625	6359.7	1	6.053	83.3	3
K.FADEDK	3.1951	0.2519	99.9	1107.546	1107.544	6874.4	1	6.154	87.5	9
K.NNLDNY	3.9889	0.379	100	1346.652	1346.649	6848.6	1	6.437	80	11
K.ATVEDK	3.7339	0.3177	100	1335.712	1332.701	5483.2	4	6.001	72.7	12
K.ATVEDK	4.2441	0.3007	100	1709.943	1709.94	8325	1	6.853	71.4	4
K.ATVEDK	5.2873	0.4139	100	2075.09	2075.087	7318.9	1	7.069	51.5	4
K.ATVEDK	5.1162	0.3939	100	2085.114	2082.104	6166.8	1	6.846	58.8	5
K.ATVEDK	6.3497	0.3817	100	2902.586	2901.578	9724	1	6.838	32.7	9
K.ATVEDK	7.3251	0.4459	100	2902.587	2901.578	9429.9	1	7.848	41	6
K.ATVEDK	5.9022	0.3149	100	5145.611	5144.587	8916.5	1	5.683	19.1	2
K.ATVEDK	5.8609	0.3107	100	5146.611	5144.587	11495.8	1	5.407	19.2	2
K.DKLADK	4.7091	0.2022	100	1440.796	1438.787	8615.5	1	6.384	81.8	9
K.DKLADK	5.6151	0.3994	100	2259.27	2258.261	7281.2	1	7.511	50	4
K.LADKIE	3.395	0.201	99.8	1188.651	1188.648	10489.7	1	5.275	88.9	7
K.LADKIE	5.76	0.4277	100	2029.176	2029.173	10167	1	8.149	51.5	6
K.LADKIE	5.881	0.501	100	2029.177	2029.173	8198.7	1	8.944	70.6	22
K.LADKIE	6.2387	0.5088	100	4017.046	4016.029	8414.5	1	9.455	30	1
K.LADKIE	4.8191	0.2893	99.9	4018.043	4016.029	7165.8	1	4.854	22.9	1
K.LADKIE	6.5228	0.4577	100	4260.169	4258.148	9262.7	1	7.38	26.1	2
K.IEKEDK	5.096	0.1431	99.9	1587.898	1587.896	6650.7	3	5.028	57.7	5
K.IEKEDK	4.8023	0.2828	100	1587.898	1587.896	6216.9	1	6.01	84.6	9
K.EDKNTI	2.7026	0.3337	99.9	1217.675	1217.674	3552.5	31	5.432	75	2
K.EDKNTI	4.5098	0.3061	99.9	3213.565	3211.547	7240.7	1	5.638	27.7	1
K.NTILSA	4.7675	0.4089	100	2834.375	2832.365	6449.5	1	7.498	46	17
K.NTILSA	7.1038	0.4217	100	2840.4	2839.382	9523.3	1	7.997	38	6
K.NTILSA	5.5093	0.429	100	3089.525	3088.519	9980.2	1	6.295	32.4	4
K.DAEDWI	5.7223	0.5167	100	2006.88	2005.874	8113.4	1	8.743	76.5	64
K.DAEDWI	4.011	0.3224	100	2263.038	2262.027	7023.1	1	5.926	47.4	3
K.LKDLEA	5.12	0.3496	100	1639.968	1639.964	7676.2	1	6.426	59.6	14
K.LKDLEA	3.9825	0.4081	100	1639.969	1639.964	6054.6	1	7.242	69.2	2
K.LKDLEA	6.0062	0.4609	100	1639.969	1639.964	8668.1	1	8.413	88.5	69

	K.DLEAVC	4.3544	0.2785	100	1398.787	1398.785	6417.5	1	7.036	81.8	6
	K.LYGQPC	5.4705	0.5255	100	2363.036	2359.021	5162.4	1	9.34	56.8	89
PFF0435w		366	61.60%	414	46055	6.9 U		Annotation|Plasmodium_falciparum_Sanger|(protein coc			
	K.SSQDYN	4.5056	0.4239	100	2868.359	2868.351	8104.3	1	8.399	37.5	17
	K.SSQDYN	4.7243	0.262	99.9	2875.373	2875.369	8539.1	1	5.571	28.1	3
	T.YGAHNY	3.1714	0.3579	99.8	1592.857	1592.855	5297.7	1	7.887	53.8	2
	R.YYDFLS	4.421	0.3903	100	3370.573	3370.581	3725.6	1	6.224	27.4	2
	R.YYDFLS	3.6712	0.47	100	3370.599	3370.581	9987.3	1	8.078	26.8	27
	R.YYDFLS	6.3108	0.5095	100	3376.62	3370.581	6717.1	1	8.315	27.7	18
	Y.YDFLSA	3.246	0.3828	100	3207.525	3207.518	4894.5	1	6.263	31.5	1
	Y.DFLSAY	3.1091	0.3558	99.9	3030.431	3030.42	4890.1	2	5.855	32.7	2
	Y.DFLSAY	4.7567	0.3017	99.9	3032.432	3030.42	6773.9	1	5.648	32.7	5
	L.SAYSSV	4.8689	0.4025	100	2669.274	2669.275	6049.7	1	6.881	30.4	1
	S.AYSSVN	5.4563	0.4525	100	2568.192	2568.209	8681.6	1	8.975	39.8	2
	S.AYSSVN	5.4806	0.5698	100	2582.229	2582.243	7316.8	1	9.55	47.7	5
	Y.SSVNQC	4.5256	0.3323	100	2348.132	2348.143	7133	1	6.63	55	1
	G.HCHPDI	3.0945	0.3961	100	1761.844	1761.853	7071	1	6.758	60.7	2
	R.AFFSDS	4.0597	0.4867	100	1387.64	1387.632	6565.5	1	9.225	77.3	21
	R.YLTNLF	3.3393	0.337	100	1233.618	1233.616	5010.8	1	5.921	88.9	21
	K.IIVCNN	3.2562	0.2882	99.9	1294.647	1293.637	5842	1	6.91	85	4
	R.TLGCVS	2.9835	0.3528	99.9	1267.64	1266.636	7144.5	1	5.822	77.3	5
	K.NNFGPF	3.694	0.3065	100	1393.73	1393.727	5484.5	1	6.314	81.8	60
	K.VPYDDL	3.6676	0.3245	100	1294.654	1291.642	3529.7	1	6.813	90	37
	K.ELQDPN	3.8144	0.4297	100	3863.861	3863.851	5360.3	1	7.627	27.1	3
	K.ELQDPN	5.2956	0.4453	100	3881.889	3877.886	7244.6	1	7.664	26.4	13
	K.ELQDPN	2.9625	0.3459	99.9	3991.954	3991.946	5555	1	6.282	23.6	2
	K.ELQDPN	4.2082	0.4144	100	4007.997	4005.981	8664.7	1	6.077	20.1	1
	K.ELQDPN	4.9734	0.2578	99.9	4008.996	4005.981	7353.8	1	5.742	20.4	1
	K.YNVLFV	4.4605	0.4957	100	1780.912	1780.923	10102.8	1	9.255	73.3	9
	K.ALSGGH	4.6974	0.3163	100	3735.904	3734.885	7688.8	1	6.006	20.1	6
	K.ALSGGH	3.1933	0.2933	99.9	3748.942	3748.919	6701.1	1	6.348	27.8	4
	S.GGHYPI	5.1341	0.3713	100	3463.741	3463.732	5071.5	1	6.354	24.2	5
	S.GGHYPI	3.5353	0.2997	99.9	3464.749	3463.732	8069.3	1	5.647	28.8	5
	S.GGHYPI	4.9593	0.3841	100	4442.307	4440.3	9317.2	1	6.511	23.8	5
	K.PGEHGS	6.2767	0.5837	100	2322.157	2319.152	8649.4	1	10.276	65.9	29
	K.LGAPFL	3.4862	0.2158	99.9	1100.642	1100.647	3935.7	1	5.173	88.9	19

	K.NDLVNV	4.199	0.3622	100	1488.742	1488.752	8845.2	1	6.586	81.8	18	
	K.EQLDEC	3.3411	0.3126	99.9	1476.733	1476.726	6128.7	1	6.242	77.3	10	
MAL8P1.17		864	60.50%	483	55514	5.8	U	Annotation|Plasmodium_falciparum_Sanger|(protein coc				
	S.HGDLFN	4.6429	0.4299	100	2210.032	2209.032	5867.4	1	8.068	63.9	14	
	S.HGDLFN	4.3997	0.3507	100	2217.062	2216.049	9204.4	1	6.251	34.7	1	
	S.HGDLFN	6.0975	0.417	100	2712.367	2712.361	6486.4	1	8.233	36.4	18	
	S.HGDLFN	5.3841	0.5008	100	2713.37	2712.361	7270.3	1	9.047	52.3	24	
	S.HGDLFN	7.756	0.467	100	2713.371	2712.361	10248.9	1	9.024	48.9	37	
	R.LIPEYN	5.0092	0.2358	100	1749.857	1748.853	3619.9	3	5.575	75	10	
	I.KLVSID	4.4609	0.3985	100	3271.692	3269.731	10405.8	1	7.18	39.7	4	
	K.LVSIDA	3.6445	0.4427	100	3162.723	3162.688	6751.2	1	7.24	32.1	29	
	K.LVSIDA	5.0369	0.4848	100	3269.728	3269.731	7153	1	7.474	28.4	11	
	K.LVSIDA	3.3428	0.3465	99.9	3269.738	3269.731	7490.1	1	5.824	29.3	6	
	R.TAQSIV	7.2478	0.4679	100	3241.687	3240.68	6705.6	1	8.789	43.8	12	
	R.TAQSIV	4.6153	0.3005	100	3485.791	3483.783	5076.8	3	5.17	21.7	2	
	K.TPLEEF	3.6695	0.264	99.9	1745.866	1742.853	3733.8	1	6.139	64.3	3	
	K.TPLEEF	5.7556	0.5224	100	2820.368	2819.342	11529.5	1	8.63	37	33	
	K.TPLEEF	6.5151	0.5296	100	2828.374	2826.359	5613.4	1	8.595	52.2	139	
	K.THFVLL	4.8435	0.2488	100	1882.994	1881.986	5423.6	1	5.273	53.3	17	
	K.THFVLL	4.7479	0.4481	100	1889.009	1889.003	7277.5	1	7.954	56.7	9	
	K.THFVLL	5.4305	0.3814	100	1890.009	1889.003	8813.2	1	7.122	70	66	
	K.ASLGLT	5.9879	0.4803	100	1996.011	1993.998	4618.4	1	9.263	63.9	37	
	K.ESLLNH	3.7678	0.4112	100	1673.923	1673.92	4524	1	6.491	69.2	1	
	K.ESLLNH	5.4179	0.3216	100	1676.932	1673.92	6004.3	1	6.715	76.9	38	
	N.HNAIINF	2.2798	0.3759	99.9	1103.602	1103.6	5568.3	1	5.836	75	2	
	N.AIINFFK	2.564	0.2747	99.9	866.5347	866.5327	2253.9	2	4.732	91.7	1	
	K.SEPIPE	3.5202	0.3694	100	1545.791	1545.787	4113.2	6	6.451	61.5	8	
	K.IVVGNS	4.5148	0.3919	100	1390.825	1388.815	6409.2	1	8.233	87.5	73	
	K.DVLIEI	4.5879	0.4459	100	1876.92	1874.912	5940.6	1	7.308	71.4	17	
	C.KKLEPV	4.178	0.3792	100	1446.796	1446.796	4849.3	1	6.155	81.8	12	
	K.KLEPVY	3.4819	0.3296	100	1318.697	1318.701	5160.9	1	6.529	75	9	
	K.KLEPVY	3.8843	0.2874	100	1318.703	1318.701	6721.5	1	6.308	67.5	3	
	K.KLEPVY	3.4137	0.2853	99.9	1446.795	1446.796	6085.8	8	5.501	45.5	1	
	K.KYDSIIV	3.4141	0.3529	100	1051.644	1050.639	3622.6	1	6.166	81.2	4	
	K.MDGTLN	4.756	0.4615	100	2562.248	2559.231	7843.7	1	7.423	52.4	32	
	K.MDGTLN	4.0127	0.3416	100	2937.477	2933.462	5003.4	1	6.794	43.8	63	

	K.MDGTLN	6.6045	0.4531	100	2938.481	2933.462	7757.5	1	7.831	40.6	32	
	K.DFEWSC	4.9674	0.3222	100	1722.856	1719.842	7896.1	1	6.71	76.9	22	
	K.DFEWSC	3.7172	0.5059	100	1727.88	1726.859	5464.5	1	8.18	69.2	2	
	K.GFVDFL	2.9763	0.2019	99.8	939.4911	939.494	4405.4	1	5.269	85.7	30	
	K.HATNTP	5.896	0.4493	100	2472.167	2472.16	3635.8	1	10.331	52.3	42	
PF14_0323		76	60.40%	149	16931	4.2	U	Annotation|Plasmodium_falciparum_TIGR|(protein codin				
	K.LTEEQIS	3.6523	0.3543	100	1230.635	1230.633	7609.9	1	6.895	88.9	3	
	R.SLGQNF	6.0736	0.4093	100	4120.982	4116.968	9418.6	1	7.164	25	4	
	R.SLGQNF	6.8713	0.4713	100	4121.986	4116.968	9979.1	1	9.351	27.1	9	
	R.KLKDTD	5.4322	0.5989	100	1944.01	1944.004	8684.8	1	10.58	73.3	12	
	K.LKDTDT	5.4366	0.5476	100	1815.914	1815.909	8939.3	1	10.57	85.7	32	
	K.DTDTEE	4.012	0.4074	100	1567.716	1567.713	7425.6	1	7.009	83.3	3	
	R.VFDRDC	4.2811	0.2984	100	1827.853	1827.851	5796.5	1	6.659	70	7	
	R.DGDGYI	3.1492	0.3063	99.9	1310.588	1310.586	5114	1	6.31	81.8	2	
	K.LTNEEV	3.2814	0.3006	99.9	1348.642	1348.642	7055.3	1	6.338	85	4	
PF08_0054		2031	59.20%	677	73915	5.7	U	Annotation|Plasmodium_falciparum_Sanger|(protein coc				
	R.NENVDI	5.2934	0.4927	100	1585.779	1585.776	5238.4	1	8.544	84.6	9	
	R.NENVDI	4.054	0.3899	100	1587.782	1585.776	3171	1	6.914	65.4	2	
	R.NPENTV	3.5382	0.357	100	1134.545	1134.543	6839.8	1	6.612	88.9	18	
	R.NPENTV	3.0328	0.3783	99.8	1134.547	1134.543	3606.7	1	6.842	72.2	2	
	R.KFTESS	3.3583	0.4546	100	1386.659	1386.658	4856.5	1	7.23	72.7	3	
	R.KFTESS	3.8046	0.4024	100	1387.663	1386.658	5005.6	1	7.279	86.4	9	
	K.FTESSV	3.3568	0.373	100	1258.564	1258.563	7260.5	1	7.694	85	9	
	K.SGVDEK	4.2283	0.4248	100	1916.94	1916.939	8283.5	1	7.245	53.1	3	
	K.SGVDEK	5.0738	0.3597	100	1916.945	1916.939	4198.5	1	6.607	62.5	3	
	K.KLFHPE	4.207	0.3248	100	1657.854	1657.862	4451.9	1	6.172	73.1	6	
	K.KLFHPE	3.3344	0.2928	99.9	1785.97	1785.957	6299	1	5.43	60.7	2	
	K.LFHPEE	4.6483	0.3865	100	1664.872	1664.88	5178.9	1	6.352	73.1	26	
	K.LFHPEE	5.4932	0.3289	100	1664.873	1664.88	6430.9	1	6.657	57.7	11	
	K.LFHPEE	4.3188	0.395	100	1664.882	1664.88	6572.4	1	7.386	65.4	4	
	K.MKENAE	3.3821	0.2336	99.9	1237.626	1237.625	6117.9	1	5.066	85	9	
	K.DAGTIA	3.1642	0.262	99.8	1217.636	1217.631	3846	20	5.237	77.3	4	
	R.IINEPTA	4.3422	0.4638	100	1553.836	1553.833	5757.3	1	7.289	71.4	4	
	R.IINEPTA	5.9033	0.5216	100	1703.984	1702.979	4928.5	1	9.339	80	103	
	R.IINEPTA	4.2637	0.393	100	1704.989	1702.979	4826.5	1	8.072	56.7	9	
	R.IINEPT	4.4029	0.416	100	1810.036	1810.023	6619.3	1	6.875	68.8	6	

E.PTAAAI	3.896	0.4479	100	1219.69	1219.691	8776.2	1	7.53	81.8	4
I.FDLGGG	2.9152	0.3672	99.9	2486.303	2486.289	8899.2	1	5.573	36.4	1
R.LVNFCV	2.9924	0.3032	99.9	1270.618	1270.614	5056	1	6.418	77.8	3
R.LVNFCV	3.5057	0.3696	100	1429.727	1426.715	6529.1	1	6.26	85	37
R.TLSSST	3.349	0.485	100	2941.523	2940.499	8153.5	1	7.832	30.8	16
S.SSTQAT	2.9708	0.3894	100	2642.372	2639.335	10848.9	1	6.382	28.3	1
R.ARFEEL	4.3202	0.443	100	1625.789	1625.786	4832	1	7.557	81.8	160
R.FEELCI	3.6579	0.3593	100	1398.639	1398.648	7361	1	5.671	88.9	12
K.SVHEVV	4.8833	0.4826	100	1339.725	1339.733	5335.9	1	9.281	83.3	314
K.SVHEVV	3.7219	0.5004	100	1339.726	1339.733	5946.6	1	8.519	70.8	220
G.AAVQAA	5.0449	0.4163	100	4031.063	4029.089	7623.5	1	7.16	22.4	2
Q.AAILSG	4.7	0.3579	100	3660.842	3659.888	8405.3	1	6.885	22.1	4
A.AILSGD	5.2706	0.4637	100	3588.86	3588.85	7303.4	1	9.308	32.4	33
A.AILSGD	5.2847	0.3712	100	3590.828	3588.85	8494.9	1	6.412	26.5	6
S.GDQSNA	5.2222	0.4422	100	3206.625	3204.613	5992.4	1	7.635	38.3	64
S.GDQSNA	4.8767	0.3319	100	3210.623	3204.613	7123.3	1	6.061	27.5	25
K.KSQIFT	5.292	0.384	100	2757.398	2756.39	9171.9	1	7.281	33.7	11
K.KSQIFT	4.1828	0.3384	100	2770.442	2770.424	4892.8	1	7.517	45.7	7
K.SQIFTT	6.1675	0.4008	100	2629.304	2628.295	10378.3	1	7.84	40.9	30
K.SQIFTT	6.1829	0.512	100	2643.335	2642.329	6289.6	1	9.036	52.3	188
G.KFHLDG	3.6235	0.3564	100	1347.763	1347.754	4678.6	1	6.461	81.8	18
K.FHLDGI	3.1847	0.3129	100	1226.682	1226.676	4853.6	1	5.726	80	27
R.KVPQIE	4.5185	0.3496	100	2614.426	2613.414	7513	1	7.035	35.9	19
R.KVPQIE	6.0653	0.5028	100	2634.471	2634.465	10737	1	9.697	58.7	115
G.KQNHIT	4.3811	0.3679	100	1325.743	1325.737	7232.8	1	7.265	90	4
K.QNHITI	3.3521	0.4157	100	1396.737	1396.73	3494.9	1	7.107	86.4	12
K.QNHITI	3.1843	0.3557	99.9	1410.766	1410.764	3451.4	14	5.504	45.5	1
R.LSQDEI	2.9514	0.3177	100	982.4885	982.4919	7035.2	1	5.585	92.9	3
K.YKAEDE	3.1828	0.3426	100	1437.713	1437.709	6202.2	1	6.321	75	9
K.YKAEDE	3.4024	0.3388	100	1913.986	1913.991	4676	1	5.372	64.3	2
R.NSLENY	3.8072	0.2743	100	1349.616	1346.605	7508.4	1	5.764	85	18
K.TITTILE	4.7546	0.3743	100	1363.803	1360.792	6970.7	1	7.536	85	323
K.TITTIL	5.499	0.4325	100	2694.414	2693.404	6427.2	1	7.383	34.1	11
K.NQLAGK	3.7813	0.4068	100	1365.669	1365.665	3870.1	1	6.759	72.7	2
K.NQLAGK	4.1278	0.3942	100	1365.67	1365.665	7839.4	1	6.868	77.3	57
K.QKEAES	4.3872	0.4802	100	1577.764	1577.767	6412.4	1	8.794	76.9	4

	K.EAESVC	3.898	0.463	100	1328.627	1328.63		4831.4	1	7.38	72.7	4
	G.GMPGA	2.8111	0.3522	99.8	2098.925	2097.922		6541.8	6	5.482	34.1	3
	M.PGAGM	3.781	0.2966	100	1912.87	1909.86		8779	1	7.389	52.5	16
	M.PGNAP/	3.0841	0.4077	100	1496.687	1496.687		6628.2	2	7.294	53.3	3
PF08_0019		111	57.90%	323	35686	6.7	U		Annotation|Plasmodium_falciparum_Sanger|(protein coc			
	R.GVLEGC	3.9336	0.421	100	2169.054	2169.046		5006.8	1	7.999	50	3
	K.LIVWNI	6.143	0.509	100	2082.056	2082.065		8250.7	1	9.308	66.7	12
	A.LSGSWI	2.6589	0.3261	99.9	1143.552	1143.555		4133.6	1	5.929	77.8	3
	R.LWDLSL	3.2059	0.284	99.9	1309.732	1309.723		7543.9	1	5.22	75	2
	R.SFIGHT	4.1332	0.5383	100	2107.01	2106.001		5547.8	1	8.501	52.8	37
	K.LWNTLA	3.0972	0.2504	99.9	1133.58	1133.578		4867.6	1	5.634	81.2	3
	K.YTITDQ	4.8601	0.5292	100	2050.983	2050.984		6848.1	1	9.257	66.7	3
	R.FSPSPN	4.8287	0.5684	100	1919.948	1919.951		4113.5	1	8.146	65.6	23
	K.NLEGHT	5.4629	0.4547	100	2585.272	2584.268		6894.1	1	8.261	46	16
	K.HLYSLE	4.9243	0.547	100	3446.549	3444.541		9390.6	1	8.861	37.5	2
	K.LIISEIY	3.915	0.3969	100	1196.766	1195.76		5832.6	1	7.005	88.9	7
PF11_0164		206	57.90%	195	21731	7.6	U		Annotation|Plasmodium_falciparum_TIGR|(protein codin			
	K.TYFDITI	3.1962	0.428	100	1667.878	1667.883		8553.3	1	7.128	53.8	3
	R.IIPNFM	4.4382	0.4938	100	2686.33	2685.335		4777.7	1	7.38	44	23
	K.KFEDEN	5.347	0.3577	100	1621.796	1621.798		4494.1	1	6.773	79.2	6
	R.GLLSMA	2.951	0.2879	99.9	961.5143	961.5141		5386	1	6.887	88.9	3
	K.NTNGSC	5.4867	0.453	100	2323.203	2323.188		5490.2	1	8.211	57.9	16
	R.HVVFGE	4.3908	0.419	100	1427.745	1427.753		4726.9	1	8.677	87.5	82
	R.HVVFGE	3.4725	0.4485	100	1427.746	1427.753		6467.9	1	7.346	75	11
	R.HVVFGE	5.2532	0.3616	100	3230.724	3229.722		5843	1	6.202	24.1	5
	R.HVVFGE	4.7397	0.4416	100	3237.766	3236.739		8533.6	1	6.637	30.2	3
	K.LVHIEA	5.3683	0.4821	100	1828.003	1828.004		7922.3	1	9.94	78.1	51
	K.LVHIEA	4.0324	0.413	100	1828.005	1828.004		7425	1	8.553	50	3
MAL8P1.69		1183	57.80%	256	29476	5	U		Annotation|Plasmodium_falciparum_Sanger|(protein coc			
	R.YDEMAI	3.553	0.3386	100	1102.439	1101.434		5972.1	1	7.225	93.8	9
	R.TLVEQC	4.8091	0.3486	100	2292.09	2291.083		4202.3	1	6.16	43.1	72
	R.TLVEQC	5.9532	0.4737	100	2292.091	2291.083		9719.6	1	7.817	66.7	329
	R.KKVEEE	3.8772	0.3173	100	2300.24	2300.236		5083.4	1	6.363	44.4	1
	R.KKVEEE	4.7568	0.2366	99.9	2415.298	2414.296		5241.9	1	5.364	36.8	6
	R.KKVEEE	5.6533	0.5036	100	2428.33	2428.331		4313.8	1	8.241	60.5	36
	R.KKVEEE	6.4285	0.5204	100	2428.341	2428.331		7155.5	1	9.295	48.7	37

	R.KKVEEE	5.4677	0.3204	100	2543.395	2542.391	5150.8	1	5.991	37.5	8
	R.KKVEEE	6.7243	0.3714	100	2543.399	2542.391	7727.9	1	7.407	50	13
	K.KVEEEL	5.7455	0.3151	100	2287.208	2286.201	8722.3	1	5.73	44.4	38
	K.KVEEEL	6.7773	0.3676	100	2301.242	2300.236	8798.9	1	9.071	72.2	110
	K.KVEEEL	7.8716	0.1888	100	2415.305	2414.296	7651.8	1	6.889	51.3	10
	K.VEEELN	3.955	0.3	99.9	2173.146	2172.141	9462.7	1	5.527	44.1	2
	K.VEEELN	5.9556	0.3364	100	2175.151	2172.141	7933.9	1	7.32	67.6	125
	V.EEELNN	4.9211	0.4204	100	2073.074	2073.073	7153.9	1	7.299	65.6	5
	K.KLIPNT	3.4848	0.2309	99.9	1333.705	1332.701	3684.6	1	4.567	77.3	4
	R.YISEFS	3.3903	0.3618	100	1469.67	1469.67	6660.9	1	6.094	77.3	3
	K.EASNCA	3.7821	0.4483	100	1398.597	1398.596	11241.1	1	7.508	77.3	24
	K.ATDIAE	4.1116	0.3455	100	1763.887	1763.893	5786.4	1	6.62	51.7	3
	K.ATDIAE	5.1858	0.4893	100	1777.928	1777.927	4793.2	1	8.179	66.7	47
	Y.SVFFYE	5.1665	0.4773	100	2320.106	2319.102	7333.7	1	8.524	55.6	8
	K.RAFDDA	5.6114	0.5882	100	2232.016	2229.006	8578.3	1	10.821	77.8	65
	R.AFDDAI	6.6896	0.4977	100	2075.911	2072.905	6933.8	1	9.774	73.5	72
	R.AFDDAI	2.8549	0.3265	99.8	2727.211	2726.203	8915.3	1	5.355	34.8	4
	R.AFDDAI	4.7181	0.143	99.9	3251.592	3250.565	7789.1	1	8.658	33.3	85
	R.AFDDAI	6.1336	0.4955	100	3252.569	3250.565	5717.7	1	8.849	39.8	67
PF11_0313		879	57.30%	316	34967	6.7	U	Annotation|Plasmodium_falciparum_TIGR|(protein codin			
	K.ILIVHV	4.9797	0.5386	100	1966.075	1966.073	5052.5	1	9.381	67.6	4
	K.ILIVHV	5.5612	0.4652	100	1969.082	1966.073	8567	1	9.562	79.4	41
	I.PPGPTG	3.4451	0.3835	100	2396.152	2396.167	4062.3	1	6.037	36.4	1
	K.GQIEIQ	3.7835	0.3873	100	1564.903	1564.911	9128.1	1	6.672	79.2	13
	K.FNMKPF	3.8643	0.2554	99.9	1560.769	1559.768	7574	1	6.094	52.1	9
	K.FNMKPF	4.2587	0.3652	100	1563.78	1559.768	3788.8	1	6.327	87.5	18
	N.MKPFSY	2.9498	0.2733	99.8	1298.658	1298.657	4147.8	6	5.118	65	8
	M.KPFSYC	2.8335	0.3193	99.9	1167.619	1167.616	4390.9	1	5.702	83.3	3
	R.TVYDDC	3.3911	0.3473	100	1365.667	1365.665	4937.9	1	7.897	77.3	9
	K.VLDITDE	4.5525	0.3681	100	1405.744	1402.732	6029	1	7.083	90.9	25
	S.KGVSNV	3.4616	0.3709	100	1103.647	1101.638	4928	1	6.95	90	6
	K.GVSNVA	3.8478	0.273	100	975.5503	973.5431	5103.1	1	5.049	88.9	9
	S.RATGVI	5.6718	0.4198	100	2223.165	2222.161	6991.2	1	8.504	42.1	7
	S.RATGVI	5.6047	0.4181	100	2233.19	2229.178	8176.5	1	7.844	63.2	9
	R.ATGVIT	6.0736	0.5684	100	2067.067	2066.06	5800	1	9.71	75	116
	R.ATGVIT	4.6349	0.4208	100	2074.094	2073.077	4427.2	1	7.312	38.9	17

	E.ASYPHV	3.1702	0.4575	100	1394.713	1394.711	6060.4	1	7.697	63.6	4
	K.NIVALII	6.0524	0.413	100	2338.285	2337.279	6232.2	1	7.5	68.4	105
	K.NIVALII	4.6858	0.3758	100	2338.286	2337.279	7145	1	6.334	35.5	3
	K.NIVALI	5.1073	0.4107	100	2468.385	2465.374	8295.9	1	7.374	55	53
	K.NIVALI	6.3243	0.4692	100	2470.388	2465.374	5201.2	1	8.378	41.2	39
	L.IIDSDYT	4.0635	0.3843	100	1799.9	1798.894	6679.8	1	7.071	67.9	2
	K.KMVENF	6.6183	0.391	100	1974.023	1973.017	7811.8	1	9.341	78.9	6
	K.MVENPE	5.0019	0.3109	100	1845.927	1844.922	10672.5	1	7.725	58.3	12
	K.MVENPE	4.6901	0.3007	100	1847.927	1844.922	5611	5	5.893	37.5	3
	K.MVENPE	5.8313	0.4132	100	1847.928	1844.922	9176.6	1	8.047	66.7	21
	K.KVEEEE	4.4957	0.5078	100	2383.933	2382.927	2497.9	1	8.775	63.2	336
PF14_0078		861	56.50%	451	51693	8	U	Annotation|Plasmodium_falciparum_TIGR|(protein codin			
	G.SEFDNV	4.9606	0.4703	100	2326.174	2325.161	10568.2	1	8.216	52.5	51
	K.DLANVL	2.8685	0.4001	99.8	1263.663	1263.658	4506.8	1	6.756	68.2	4
	K.DLANVL	4.4403	0.3311	100	1264.665	1263.658	5614.2	1	7.19	81.8	33
	K.FNFLFH	3.0331	0.3014	100	996.4954	996.4943	5077.2	1	5.8	85.7	9
	K.FNFLFH	3.1396	0.3397	100	1284.604	1284.601	7485	1	7.269	80	6
	F.HTASSN	3.411	0.465	100	1432.769	1432.766	6252	1	7.29	79.2	1
	K.AGTISG	2.7391	0.3192	99.9	994.5781	994.576	2515.8	2	5.576	77.8	3
	K.FIEMTE	4.6813	0.5202	100	3200.515	3198.502	4884.8	1	8.89	42.6	62
	K.DLSIGSI	3.9203	0.4451	100	1684.959	1682.952	4389.5	1	7.054	60.7	2
	K.DLSIGSI	5.3514	0.5029	100	1685.966	1682.952	5901.7	1	9.242	78.6	39
	K.TQNKIE	5.5905	0.5205	100	2249.207	2249.2	5875.1	1	9.398	69.4	10
	K.NKGYLT	3.8953	0.3727	100	1463.808	1463.804	7605.6	1	7.001	79.2	1
	K.GYLTIG(	3.6489	0.3601	100	1221.67	1221.667	4382.8	1	6.93	80	7
	R.FFDGPL	3.3237	0.3315	100	1231.594	1229.584	3549.9	1	6.141	83.3	15
	K.LNHDL	3.2901	0.294	99.9	2455.21	2454.188	6388.9	1	5.251	40	5
	K.KANVIL	5.9202	0.3516	100	3394.795	3388.768	6286.2	1	7.222	35	16
	K.KANVIL	4.6811	0.5066	100	3405.815	3402.802	3469.2	1	8.465	33.3	23
	K.ANVILD	4.8564	0.2959	99.9	3275.721	3274.708	4650.7	1	5.749	32.8	8
	K.ANVILD	4.0479	0.2117	99.9	3278.723	3274.708	3227.6	1	6.021	27.6	46
	V.ITVPTE	3.7041	0.4122	100	2198.134	2197.129	5119.2	1	7.263	50	4
	I.TVPTEF	3.6197	0.3707	100	2078.03	2077.028	4510.4	2	6.809	44.1	9
	T.VPTEFF	4.7293	0.475	100	1976.985	1975.981	6820.4	1	8.352	71.9	24
	V.PTEFFN	5.4723	0.495	100	1876.915	1876.912	7280.7	1	9.657	80	63
	V.PTEFFN	4.0177	0.3572	100	1876.917	1876.912	5603.1	1	7.363	60	2

	K.VPFLSL	4.5327	0.403	100	1699.865	1699.873	8295.2	1	7.697	71.4	43	
	K.VPFLSL	3.9111	0.4324	100	1699.893	1699.873	5058.4	1	8.718	57.1	23	
	V.PFLSLY	3.7638	0.3725	100	1601.809	1600.804	6192.9	1	6.151	69.2	3	
	K.QYLEPL	2.7686	0.3159	99.8	1543.817	1543.812	8873.2	1	6.078	70.8	1	
	K.QYLEPL	5.5095	0.4158	100	2962.516	2962.531	5072.3	1	7.248	36.5	39	
	K.QYLEPL	4.9461	0.5029	100	2984.59	2983.583	6867.5	1	8.715	43.8	134	
	E.PLENIF	4.4905	0.4354	100	2450.339	2450.334	8192	1	7.371	57.5	3	
	K.NTFVLG	3.7707	0.2083	99.9	1299.654	1296.641	7309.4	1	5.636	80	26	
	K.YFTVYD	5.2717	0.5274	100	2125.016	2124.008	10279.9	1	8.251	67.6	146	
PFL0460w		35	56.50%	108	11996	6.1	U	Annotation|Plasmodium_falciparum_Sanger|(protein coc				
	R.EVVGTL	4.9542	0.4414	100	2067.173	2067.168	7111.1	1	8.423	58.3	14	
	R.EVVGTL	6.7118	0.4858	100	2865.566	2865.566	6338.6	1	8.354	37.5	2	
	R.EVVGTL	7.1091	0.3668	100	4084.118	4083.123	6011.4	1	6.06	23.3	17	
	R.GTSVAL	4.2287	0.4672	100	2661.381	2660.367	5309.9	1	8.703	43.8	2	
PFE1590w		347	56.40%	181	19030	5.4	U	Annotation|Plasmodium_falciparum_Sanger|(protein coc				
	G.NSSSH	5.3434	0.5539	100	4665.884	4663.872	9259	1	9.168	19.5	3	
	S.HSPSSS	6.5665	0.4573	100	4290.752	4288.733	8992.9	1	10.692	24.5	3	
	K.NASLDK	4.8399	0.1911	100	1502.767	1502.77	9676.5	2	5.241	79.2	12	
	K.NASLDK	4.2149	0.2226	99.9	1630.869	1630.865	5700.7	1	5.182	73.1	2	
	K.NNVDDL	3.964	0.371	100	2095.858	2094.847	5379.4	1	6.915	55.6	6	
	K.NNVDDL	6.3792	0.5822	100	2624.146	2623.138	6636.9	1	10.629	40.2	15	
	K.NNVDDL	7.0963	0.5882	100	2626.153	2623.138	9563.8	1	11.313	63	294	
	K.NNVDDL	4.1198	0.3716	100	2752.244	2751.233	3816.8	12	5.623	28.1	3	
	D.SDVEEA	5.5023	0.4659	100	1838.83	1837.819	7973	1	10.059	78.1	3	
	K.KDEVKT	4.5406	0.3108	100	1587.829	1587.823	9614	1	6.051	79.2	6	
PFE0285c		83	56.00%	100	11060	4.9	U	Annotation|Plasmodium_falciparum_Sanger|(protein coc				
	K.VRSPDG	3.8464	0.1712	99.8	1351.705	1351.701	7599.2	1	6.169	77.3	14	
	R.SPDGAE	3.5098	0.3613	100	1097.537	1096.532	5681.3	1	6.324	83.3	9	
	K.LMEVYC	3.0041	0.2188	99.9	1087.507	1084.492	4842.8	5	4.8	92.9	6	
	R.LGQSMI	2.9929	0.3237	100	993.507	990.5042	5421.6	1	6.167	93.8	9	
	R.IHGDNT	5.2477	0.4328	100	2830.356	2830.346	10620.8	1	7.902	30.8	3	
	R.IHGDNT	5.5589	0.5287	100	2832.358	2830.346	9032.8	1	9.436	46.2	42	
PF13_0304		1548	55.80%	443	48959	9.1	U	Annotation|Plasmodium_falciparum_Sanger|(protein coc				
PF13_0305		1548	55.80%	443	48959	9.1	U	Annotation|Plasmodium_falciparum_Sanger|(protein coc				
	K.THINLV	5.8288	0.5177	100	1602.915	1602.915	7945.7	1	9.793	85.7	113	
	K.THINLV	3.754	0.3623	100	1606.948	1602.915	7046.9	1	5.76	48.2	2	

K.STTTGH	3.5366	0.3742	100	1134.637	1134.635	5147.8	1	7.02	72.2	12
R.YFFTVI	3.7099	0.3719	100	1394.713	1394.711	4508.8	1	6.811	61.4	9
R.YFFTVI	3.0037	0.3801	99.8	1402.734	1401.728	4324	1	6.536	54.5	1
R.YFFTVI	4.6513	0.4324	100	1403.735	1401.728	5801.6	1	7.499	86.4	187
F.FTVIDA	2.9408	0.3024	99.9	1084.575	1084.579	6929.8	1	5.65	77.8	2
F.FTVIDA	3.6225	0.3437	100	1587.845	1587.854	7173.2	1	6.04	80.8	13
F.TVIDAP	3.1645	0.3877	100	1456.818	1454.82	7268	1	7.088	66.7	2
T.VIDAPG	3.1091	0.2842	99.9	1353.772	1353.772	6698.3	1	5.672	77.3	2
K.NMITGT	4.9162	0.341	100	2909.49	2908.477	9203.6	1	5.975	34.8	44
K.NMITGT	4.4445	0.4706	100	2916.496	2915.494	6345.5	1	7.034	42.9	70
T.GTSQAD	3.8299	0.2449	99.9	2450.254	2449.262	5747.5	1	5.494	43.8	12
G.TSQADV	5.344	0.4261	100	2392.248	2392.24	5028.4	1	7.538	58.7	177
G.TSQADV	4.5683	0.2747	99.9	2393.252	2392.24	6788.4	1	6.172	39.1	6
T.SQADVA	4.2671	0.3941	100	2291.179	2291.192	5971.2	1	6.548	47.7	6
L.LVVPAE	3.9516	0.4556	100	1606.852	1606.848	4952.5	1	7.432	76.7	3
L.LVVPAE	3.4146	0.5151	100	1606.854	1606.848	4749.6	1	8.093	56.7	4
V.PAEVGC	2.8621	0.398	99.8	1295.63	1295.627	4688.5	1	6.894	66.7	1
V.PAEVGC	3.1577	0.3396	99.9	1295.635	1295.627	4699.1	1	6.56	83.3	6
K.YSEDRY	3.3702	0.2421	99.9	1466.726	1466.724	6006.9	1	4.722	80	4
K.KVGYQA	4.9143	0.4335	100	2804.488	2803.482	5674	1	7.845	41.7	16
K.KVGYQA	6.251	0.4254	100	2805.501	2803.482	6311.6	1	7.319	38.5	13
K.VGYQAD	5.9518	0.5384	100	2675.392	2675.387	4936.4	1	9.127	52.2	38
K.VGYQAD	6.2578	0.4952	100	2676.394	2675.387	7334.5	1	8.606	38	15
G.YQADKV	5.1182	0.4908	100	2519.302	2519.297	5462.5	1	8.285	47.6	3
Q.ADKVDF	5.7306	0.5439	100	2228.176	2228.175	3950.2	1	8.865	68.4	39
Q.ADKVDF	4.9012	0.3964	100	2228.183	2228.175	5811.1	1	7.507	47.4	2
A.DKVDFI	3.7935	0.3083	100	2136.089	2136.086	4637.2	1	5.968	52.8	3
K.VDFIPI	3.963	0.3976	100	1916.028	1914.016	5652.4	1	7.776	56.2	6
K.VDFIPI	5.2157	0.397	100	1917.024	1914.016	4166.7	1	8.199	78.1	61
I.PISGFEG	4.6619	0.4083	100	1433.753	1432.751	5603.6	1	7.673	91.7	6
K.SDKTPW	3.07	0.3212	100	1025.513	1024.51	5699	3	5.354	85.7	3
R.TLIEAL	3.7687	0.3259	100	1457.76	1457.756	4846	1	6.418	79.2	3
R.TLIEAL	4.5312	0.4826	100	1873.977	1873.973	4874.3	1	8.521	63.3	2
R.TLIEAL	3.4217	0.2584	99.8	2117.097	2117.095	6858.7	1	6.215	44.1	2
R.TLIEAL	4.1777	0.3068	100	2483.318	2483.333	7724.9	1	5.739	40	12
N.FAPSAV	3.798	0.474	100	1194.582	1194.583	3094.2	1	8.313	85	15

	A.PSAVVS	3.2854	0.2618	99.9	976.4778	976.4774	6483.8	1	6.986	93.8	3	
	K.SVEMHK	5.5337	0.3676	100	2600.307	2598.299	9992.3	1	7.074	35.2	11	
	K.SVEMHK	4.508	0.2764	100	2606.304	2605.316	6790.9	1	6.498	43.2	5	
	K.EVLEEA	5.3818	0.4589	100	1886.961	1886.961	7062.5	1	8.546	68.8	30	
	K.EVLEEA	4.0472	0.3912	100	1895.986	1893.978	5351.3	1	6.641	53.1	1	
	E.EARPGD	3.4113	0.3201	100	1423.744	1423.741	9205.4	3	6.331	62.5	1	
	E.ARPGDN	3.355	0.3772	100	1294.703	1294.698	9425.7	3	6.142	63.6	3	
	K.RGYVAS	3.6257	0.3237	100	1535.78	1535.782	6617.9	1	6.65	46.2	3	
	K.RGYVAS	3.7917	0.3405	100	1538.791	1535.782	4684.9	1	6.325	73.1	12	
	R.GYVASD	3.5812	0.4411	100	1382.693	1379.681	8235.8	1	7.464	79.2	9	
	R.GYVASD	3.5968	0.4128	100	1812.868	1811.86	5245.1	1	6.69	56.2	3	
	K.FTAQVII	4.729	0.501	100	1700.99	1701	8611.4	1	8.744	71.4	66	
	T.AQVIILN	3.3478	0.3358	99.8	1431.834	1431.832	4766.3	1	6.735	58.3	2	
	T.AQVIILN	4.6159	0.4801	100	1454.885	1452.884	9377.1	1	8.133	79.2	50	
	A.QVIILNH	3.4786	0.2857	99.9	1360.794	1360.795	4958.7	8	5.253	68.2	3	
	Q.VIILNHP	3.8083	0.4212	100	1253.789	1253.788	7263.3	1	6.704	85	17	
	K.NGYTPV	5.5356	0.5567	100	1995.917	1995.913	6992.5	1	10.212	68.8	174	
	K.NGYTPV	4.0848	0.5138	100	1995.92	1995.913	5489.1	1	7.677	53.1	24	
	N.GYTPVL	3.4498	0.4105	100	1881.88	1881.87	7768.6	1	6.575	46.7	1	
	G.YTPVLD	3.3365	0.3774	100	1824.857	1824.849	6227.4	1	6.981	60.7	3	
	T.PVLDCH	3.5849	0.4553	100	1560.741	1560.738	5946	1	7.81	62.5	3	
	T.PVLDCH	3.8026	0.435	100	1561.741	1560.738	7624.5	1	8.175	79.2	10	
	K.SGDSAL	4.0422	0.3606	100	1202.623	1202.627	7951.6	1	6.984	86.4	9	
	K.KPMVVE	4.1166	0.3732	100	1863.961	1863.968	5387.8	1	5.84	46.7	3	
	K.KPMVVE	5.3614	0.5257	100	1863.968	1863.968	9159.6	1	9.862	73.3	165	
	K.KPMVVE	4.0338	0.4606	100	1863.97	1863.968	4073.4	1	8.96	66.7	22	
PFC0975c		275	55.60%	171	18953	8.1	U	Annotation|Plasmodium_falciparum_Sanger|(protein coc				
	K.VFFDISI	4.033	0.4329	100	1554.75	1554.755	7313	1	7.138	76.9	18	
	R.IIFELFS	2.836	0.4021	99.8	1472.856	1471.846	4091.8	1	6.216	68.2	1	
	R.IIFELFS	4.8225	0.4411	100	1474.847	1471.846	6988.2	1	8.431	86.4	214	
	R.IIPQFM	5.7522	0.5446	100	2728.254	2728.246	3557.6	1	10.302	50	13	
	I.PQFMCQ	4.2413	0.3953	100	2502.08	2502.078	4565	1	7.488	37	2	
	K.HDQPGI	3.9384	0.4066	100	3712.805	3712.789	5145.7	1	5.754	25	2	
	K.HDQPGI	3.6003	0.483	100	3719.821	3719.806	5727.6	1	7.994	30.3	19	
	N.SSQFFI	2.9698	0.3097	99.8	2002.026	2002.022	4513.2	1	5.105	53.1	2	
	K.VIEGMN	3.2102	0.3124	100	1016.553	1016.556	3315.6	1	6.109	93.8	4	

PF14_0655		437	54.80%	398	45310	5.7	U		Annotation|Plasmodium_falciparum_TIGR|(protein codin				
	K.EETFNN	4.8551	0.5253	100	3300.475	3299.471		8069.9	1	9.903	39.3	39
	K.EETFNN	5.6196	0.4526	100	3300.483	3299.471		6376.6	1	7.699	31.2	15
	Q.RGIKPI	4.1835	0.353	100	2296.258	2295.272		5608	1	6.461	50	1
	R.GIKPIL	4.8729	0.35	100	2140.179	2139.171		6711.2	1	6.59	40	4
	R.GIKPIL	6.3464	0.5439	100	2142.192	2139.171		7671.8	1	11.107	70	22
	K.PILNGY	3.4135	0.3834	100	1833.953	1833.953		5209.6	1	6.977	47.1	1
	K.CHACVC	2.9207	0.3356	99.9	1215.572	1215.573		4423.3	1	6.242	75	3
	K.QGVHM	2.4443	0.4301	99.9	1237.641	1237.648		3066.1	45	6.957	50	3
	K.LFILDE	4.5351	0.2957	100	1551.783	1551.773		8100.6	1	6.869	87.5	13
	K.KLVPDI	2.8909	0.3602	99.9	2828.596	2827.583		5437.2	1	5.291	41.7	1
	K.LVPDIQ	6.1788	0.474	100	2703.5	2699.488		6370.7	1	7.905	50	127
	L.VPDIQV	5.0761	0.4558	100	2586.412	2586.404		6756.7	1	7.626	45.5	1
	V.PDIQVA	6.059	0.4392	100	2488.344	2487.335		6035.6	1	8.929	61.9	42
	M.PQEILE	3.7359	0.3986	100	1206.68	1206.681		5436.3	1	6.936	88.9	3
	K.KDELTL	3.1323	0.2641	99.9	1180.664	1180.665		5065.2	2	5.682	77.8	1
	K.LDTLCD	4.5371	0.4835	100	2830.421	2827.416		5770.1	1	8.291	47.7	53
	K.KVDILT	4.5243	0.3658	100	1483.768	1483.769		8181.8	1	7.106	90.9	48
	R.GIDVQC	3.8095	0.3732	100	2835.501	2834.499		8037.3	1	6.39	41.7	10
	A.INFVTN	4.1445	0.3488	100	1806.955	1806.96		5401.6	1	6.521	60.7	2
	K.KIESYY	5.2085	0.4803	100	2536.254	2535.24		6536.6	1	8.589	52.5	48
PF14_0443		196	54.80%	168	19311	4.7	U		Annotation|Plasmodium_falciparum_TIGR|(protein codin				
	E.KSFTER	3.986	0.4104	100	2183.066	2183.062		4596.6	1	7.091	35.3	2
	E.KSFTER	3.7446	0.2285	99.9	2197.1	2197.096		7395.8	1	6.499	50	3
	K.SFTERP	4.5059	0.2366	100	2070.008	2069.002		7015	1	6.921	59.4	15
	K.SFTERP	4.0548	0.3584	99.9	3865.887	3863.876		2381.1	1	5.937	24.2	2
	R.EAFNLL	4.2648	0.3388	100	1816.905	1813.893		7730.8	1	6.325	68.8	8
	K.CAMQSI	3.5503	0.3903	100	1193.567	1193.566		7560.7	1	8.045	90	6
	K.DGYSSI	4.8243	0.5314	100	2111.972	2110.972		9263.4	1	8.492	70.6	115
	R.IFNLFD	4.0272	0.3217	100	1812.941	1812.938		3737.6	3	5.698	46.7	2
	R.IFNLFD	5.6853	0.4744	100	1829.985	1826.973		8202.5	1	8.267	80	34
	K.ELGETL	3.7391	0.4031	100	1407.697	1404.686		6326.4	1	7.629	81.8	9
PF14_0486		793	54.60%	832	93523	6.8	U		Annotation|Plasmodium_falciparum_TIGR|(protein codin				
	R.NMSVIA	3.799	0.2978	100	1314.662	1314.67		5228.1	1	6.66	77.3	46
	M.SVIAHV	3.3026	0.2065	99.8	1063.574	1062.57		6672.6	1	5.584	83.3	7
	K.PFLINL	3.7612	0.3244	100	2612.398	2612.391		5497.6	1	6.174	43.5	1

K.PFLINL	4.644	0.4098	100	2613.396	2612.391	7820.2	1	6.655	33.7	1
R.VTDGAL	6.1035	0.5229	100	3242.682	3241.674	8063.6	1	8.676	31.9	13
R.VTDGAL	5.4652	0.4769	100	3244.682	3241.674	6549.7	1	9.523	41.4	40
V.VVDTIE	3.284	0.3034	99.8	2587.33	2586.32	5176.5	3	5.748	34.1	4
R.ALLELQ	5.3244	0.5237	100	2177.113	2176.107	8244.5	1	9.002	67.6	14
R.LWGNSI	3.1324	0.2468	99.9	1201.573	1200.569	3743.8	2	5.199	83.3	3
R.GFCQFI	3.7367	0.305	100	2697.252	2694.27	7021.1	1	5.876	47.6	1
R.GFCQFI	3.1076	0.4122	100	2951.415	2951.408	5859.6	1	5.738	34.8	2
K.AMQLWI	3.5807	0.2566	99.9	3054.586	3053.592	4835.4	2	5.798	31.5	13
L.WLPAGD	3.9413	0.4591	100	2610.376	2610.372	7096.7	1	7.049	43.5	14
W.LPAGDT	4.2527	0.3696	100	2417.281	2417.275	7948.3	1	6.003	36.4	2
W.LPAGDT	4.9125	0.433	100	2425.3	2424.292	7650.9	1	7.829	54.5	15
L.PAGDTL	5.1342	0.4533	100	2314.222	2311.208	7481.4	1	7.929	52.4	7
E.MIVTHL	4.8271	0.3202	100	1515.816	1514.811	4852.3	1	6.894	80.8	30
M.IVTHLP	3.2851	0.318	99.9	1377.759	1376.754	3746	1	5.865	70.8	7
K.YRVENL	5.1918	0.468	100	2333.094	2333.095	8272.2	1	8.38	65.8	1
R.VENLYE	5.6216	0.5559	100	2013.939	2013.93	5784.6	1	10.113	76.5	5
R.IQGPHY	3.4153	0.3083	100	1231.648	1231.655	7913.3	3	6.959	75	11
R.IQGPHY	3.1917	0.3527	99.8	1231.65	1231.655	4598.3	2	6.462	60	1
Q.GPHYVI	2.4568	0.3823	99.9	983.4929	983.4951	3439.5	1	5.396	81.2	3
R.YTEQVC	3.2405	0.4493	100	2953.368	2952.366	4546.4	1	6.581	33.3	3
T.FKEAHN	3.7209	0.2317	99.9	1303.652	1303.647	4591.2	1	7.149	90	3
K.SDPLVL	5.2255	0.372	100	3410.679	3410.666	6767.8	1	6.999	34.5	6
K.DLKDEY	5.4397	0.4121	100	2487.273	2486.264	7512.9	1	8.499	62.5	58
R.ETVTEE	4.0405	0.4021	100	1574.769	1574.77	8408.8	1	7.734	69.2	8
K.AYPLAE	3.7055	0.4092	100	1493.802	1493.797	5335.5	1	8.214	69.2	5
K.AYPLAE	3.5857	0.4274	100	1493.802	1493.797	6464.4	1	7.677	65.4	1
K.AYPLAE	3.3388	0.4587	100	1735.938	1735.934	5161	1	7.272	53.3	1
R.ANYLHS	3.9638	0.3565	100	1522.71	1522.708	6971.6	1	6.597	81.8	51
R.ANYLHS	3.7111	0.3945	100	1522.717	1522.708	5319.1	1	7.117	63.6	2
K.IWAFGP	5.1035	0.3943	100	3150.633	3150.619	5853.7	1	6.422	37	5
K.IWAFGP	5.1676	0.4636	100	3151.632	3150.619	10309.3	1	7.965	48.1	23
K.VHCVAA	3.5858	0.4545	100	1403.699	1403.69	6068.1	1	8.267	81.8	11
R.GAGQIM	3.0842	0.3838	100	1039.515	1039.514	5552.9	1	6.042	72.2	1
R.GAGQIM	3.0985	0.2401	99.8	1160.593	1160.592	5296.5	1	5.655	70	8
K.KCIYAC	3.8441	0.4316	100	1629.787	1628.793	8073.6	1	7.069	75	3

	K.CIYACE	3.564	0.3816	100	1508.721	1507.715	11046.5	1	7.255	81.8	2
	P.RLVEPI	4.1821	0.4948	100	3032.645	3032.613	7930.9	1	7.964	36.5	7
	R.LVEPIY	6.1266	0.4915	100	2891.58	2890.546	7778.9	1	8.92	41	29
	R.LVEPIY	5.5769	0.4236	100	2894.565	2890.546	7880.3	1	7.664	48	113
	K.IQSHLP	4.8955	0.5253	100	1976.031	1976.024	5366.2	1	9.487	52.9	28
	K.IQSHLP	5.1556	0.4649	100	1983.049	1983.041	7609.5	1	7.836	45.6	37
	K.IQSHLP	5.3678	0.4823	100	1984.048	1983.041	3615.7	1	8.423	70.6	92
	R.AATSG	3.7354	0.4139	100	3203.423	3202.422	4712.3	1	8.095	29.6	33
	R.AATSG	4.9026	0.4151	100	3204.433	3202.422	7521.7	1	6.9	25.9	15
	K.VEMPQL	4.4712	0.2096	100	1591.804	1591.804	6513.6	1	5.927	87.5	7
PF08_0131		315	54.50%	220	25164	6.8	U	Annotation|Plasmodium_falciparum_Sanger|(protein coc			
	G.ATFPNF	4.0963	0.4442	100	2314.112	2312.088	5960.6	1	7.165	47.5	35
	K.YVGDNV	3.9031	0.4732	100	3352.586	3351.578	7816.8	1	7.116	28.6	21
	K.YVGDNV	4.723	0.3889	100	3358.603	3351.578	5167.5	1	6.072	25	6
	K.YVGDNV	6.0894	0.4396	100	3359.603	3358.596	9674.7	1	7.451	29.5	54
	I.LFSHPH	3.291	0.4283	100	2434.132	2433.155	5197.3	1	6.652	42.5	6
	L.FSHPHD	4.0203	0.4748	100	2321.076	2320.071	5832.3	1	7.701	52.6	6
	F.SHPHDF	5.6592	0.5525	100	2174.009	2173.002	5830.2	1	8.796	66.7	43
	F.SHPHDF	4.7259	0.4146	100	2174.011	2173.002	9466.5	1	7.852	44.4	3
	K.FYGNLD	5.054	0.4337	100	2246.014	2245.006	5413.9	1	8.217	61.8	8
	R.CVFFIS	3.1995	0.2355	99.9	1240.644	1240.64	7331.5	1	5.645	83.3	12
	K.ATVLYP	2.8858	0.33	99.9	1149.628	1149.627	3508.3	1	6.27	95	2
	K.SLQLTN	5.61	0.4776	100	1906.011	1906.019	8277.6	1	8.959	65.6	33
	Q.LTNTHP	4.9532	0.4925	100	1577.835	1577.844	5189.1	1	8.569	69.2	30
	Q.LTNTHP	3.3572	0.3208	99.9	1577.846	1577.844	5167.6	2	5.517	44.2	3
	Q.LTNTHP	4.1959	0.5238	100	1577.852	1577.844	4244.3	1	8.613	61.5	21
	L.TNTHPV	3.2505	0.4475	100	1464.763	1464.76	4538.2	1	7.301	62.5	1
	L.TNTHPV	3.4909	0.3756	100	1464.77	1464.76	5222.4	1	7.957	62.5	9
	T.NTHPVA	3.4776	0.4336	100	1363.704	1363.712	3857.5	1	6.802	68.2	2
	T.NTHPVA	3.2859	0.3656	100	1366.721	1363.712	4471.5	1	6.216	72.7	18
	K.CCILPS	2.939	0.3334	99.9	1601.762	1601.767	4401.4	1	5.857	65.4	2
PFE0165w		169	54.10%	122	13741	7.8	U	Annotation|Plasmodium_falciparum_Sanger|(protein coc			
	R.VNDNCV	4.4773	0.4579	100	1584.682	1584.679	7334.6	1	8.252	75	9
	K.GASTTL	3.9233	0.2196	99.9	1570.849	1569.845	8612.7	1	4.886	60.7	3
	K.GASTTL	5.6791	0.4874	100	3415.739	3414.729	10632.8	1	8.524	33.3	21
	K.NNEIQC	4.4735	0.4518	100	1863.901	1863.903	7530.6	1	9.082	73.3	7

	K.KIEGVN	8.3277	0.5444	100	2345.296	2342.301	11018.5		1	11.03	76.2	55
	K.KIEGVN	5.2787	0.2925	100	2345.297	2342.301	7578		1	5.956	35.7	3
	K.IEGVNV	6.0273	0.5799	100	2200.176	2200.171	8786.3		1	10.896	65	68
	K.IEGVNV	4.5913	0.2809	99.9	2214.2	2214.206	8898.2		1	6.785	33.8	3
PFF1300w		549	53.40%	511	55661	7.6	U	Annotation|Plasmodium_falciparum_Sanger|(protein coc				
	K.KTHIVC	3.7364	0.3505	100	1491.78	1491.789	8250.4		1	6.187	66.7	3
	K.THIVCT	3.5138	0.5048	100	1356.681	1356.677	6378.8		1	9.138	72.7	7
	K.THIVCT	4.1103	0.3948	100	1366.705	1363.694	7580.1		1	7.42	86.4	41
	R.FNFSHG	2.971	0.3091	99.8	1441.624	1441.625	4144.9		1	6.084	77.3	2
	K.LVTDYE	5.8795	0.5784	100	2291.018	2291.033	6799.5		1	10.821	66.7	64
	K.LVTDYE	3.5584	0.3108	99.9	2412.13	2412.11	6945.1		1	5.5	55.3	3
	K.LPQSVK	3.8483	0.3348	99.9	2196.193	2196.206	5729.5		5	5.293	28.8	3
	K.LPQSVK	4.8726	0.4237	100	2196.222	2196.206	4193.5		1	7.213	57.5	15
	Q.SVKPGN	3.0513	0.3115	99.9	1879.065	1879.062	6044.8		2	5.646	44.1	1
	K.VLETHE	3.2598	0.2909	99.9	1754.896	1754.904	5338.2		1	5.857	53.6	1
	K.VLETHE	4.8682	0.3	100	2460.282	2460.273	7309		1	5.828	42.9	10
	K.VLETHE	6.6082	0.5159	100	2460.285	2460.273	9115.9		1	10.256	66.7	53
	K.NDILNF	3.5125	0.3168	100	3028.514	3027.508	5244.5		1	6.357	40.4	2
	A.ASFIQS	3.4252	0.3223	100	1215.604	1215.608	8874.2		1	5.845	85	1
	K.IENIEGII	4.4121	0.3361	100	1455.83	1455.822	8328.3		1	6.73	86.4	29
	K.ILAESDC	4.2429	0.5107	100	1309.748	1309.745	5703.8		1	8.999	81.8	6
	K.CNLQGK	3.0593	0.3829	100	2264.172	2264.145	5827.8		28	5.737	31.6	2
	R.AEVTDV	5.3293	0.4345	100	2681.246	2680.245	7658.9		1	8.198	48.1	35
	R.AEVTDV	3.1747	0.368	100	3886.895	3882.882	8300.3		1	6.388	21.6	6
	R.AEVTDV	7.1321	0.5313	100	3891.908	3889.899	8316.8		1	8.309	25.7	21
	K.ICLEAEA	3.2716	0.3937	100	1498.705	1498.711	9322.2		1	6.608	72.7	2
	K.LLYQSL	6.1972	0.5117	100	2431.373	2428.364	9336.6		1	9.373	39.3	30
	K.LLYQSL	6.43	0.6163	100	2432.392	2428.364	7411.4		1	10.364	54.8	120
	L.LYQSLV	3.8467	0.3858	100	2316.272	2315.28	5528		1	6.803	42.5	1
	Q.SLVNAI	3.3938	0.29	99.9	1897.068	1897.039	7203.1		1	6.127	52.9	2
	K.YKPSCT	4.7457	0.5233	100	1941.002	1941	6015.8		1	9.373	73.5	28
	K.PSCTIL	4.3914	0.5057	100	1656.859	1656.859	6461.4		1	9.292	80	8
	A.KVGDSV	2.9417	0.295	99.8	1357.84	1357.847	6306.7		1	5.698	62.5	1
	K.VGDSVI	4.2221	0.3731	100	1229.752	1229.752	5451.2		1	7.953	86.4	38
	K.VGDSVI	3.5411	0.4617	100	1229.753	1229.752	5486.2		1	6.794	68.2	12
	K.VGDSVI	4.4341	0.4879	100	2354.236	2354.239	8416.4		1	8.661	54.5	2

PFA0400c		182	53.40%	204	23081	5.2	U		Annotation|Plasmodium_falciparum_Sanger|(protein coc				
	K.MNNNV'	4.575	0.3855	100	2712.435	2711.427		9136.5	1	6.701	38	3	
	K.MNNNV'	5.7587	0.5453	100	2713.441	2711.427		7326.5	1	10.583	56.5	53	
	R.QDAEMI	3.5146	0.3144	100	2601.158	2600.155		4971.1	1	6.983	38.1	6	
	R.FSPYFV	4.4554	0.512	100	1620.891	1620.89		5558.6	1	8.711	76.9	50	
	S.PYFVNP	4.3027	0.3794	100	1387.793	1386.79		6948.4	1	7.061	86.4	22	
	K.LKHYVD	2.7137	0.3251	99.9	1346.66	1346.659		6993.1	1	5.969	70	3	
	K.LKHYVD	3.5252	0.3594	100	1474.755	1474.754		5771.9	1	7.253	68.2	3	
	K.KVNYEF	6.0146	0.3928	100	1959.029	1958.028		4205.2	1	7.276	78.1	9	
	K.VNYEPY	4.5973	0.4238	100	1836.954	1836.95		5128	1	7.362	76.7	8	
	K.DQDEN(	4.8736	0.5117	100	2433.159	2431.152		5907.2	1	9.078	57.5	25	
PF07_0029		1177	53.00%	745	86166	5	U		Annotation|Plasmodium_falciparum_Sanger|(protein coc				
	R.ELISNA!	3.631	0.3188	100	1275.639	1275.643		6310.9	1	6.145	81.8	10	
	R.ELISNA	3.6906	0.3517	100	1544.828	1544.828		8245.2	1	5.679	76.9	9	
	K.IRYESIT	3.5664	0.2975	100	1367.738	1367.736		5600.7	1	5.32	80	2	
	K.LSAEPE	3.8861	0.3099	100	1217.658	1215.649		3376.6	1	5.7	94.4	18	
	K.NDLINNI	4.256	0.3164	100	1327.754	1327.752		6009.2	1	6.542	86.4	10	
	K.NNDDE	4.5537	0.5112	100	2319.026	2318.021		5787.5	1	9.034	57.5	7	
	K.IILHLKE	4.1088	0.3596	100	2183.224	2183.226		9601.3	1	6.049	45.3	1	
	H.LKEDQL	5.2781	0.1962	100	1536.784	1536.78		7635	1	6.226	86.4	12	
	H.LKEDQL	3.5983	0.268	99.9	1692.881	1692.881		4806.6	1	5.6	52.1	3	
	H.LKEDQL	4.4081	0.2511	100	1693.886	1692.881		9053	1	6.829	79.2	3	
	K.KHSEFI!	3.2852	0.3498	100	1346.775	1346.766		4212.1	1	5.343	80	3	
	K.HSEFISI	3.9459	0.3372	100	1218.675	1218.671		3115.7	1	6.649	83.3	46	
	K.KTGED	4.4352	0.3578	100	5606.469	5604.451		5981.7	1	5.988	17.6	3	
	K.KTGED	6.2892	0.4033	100	5606.47	5604.451		9932.2	1	6.544	17	6	
	K.KTGED	5.5805	0.4126	100	5608.473	5604.451		10214.2	1	6.858	15.2	6	
	K.KTGED	5.5111	0.221	99.9	5735.566	5732.545		7836.6	2	4.433	15.4	1	
	K.TGEDK	4.3644	0.3026	99.9	3990.684	3990.672		7519.9	1	4.837	22	3	
	K.TGEDK	4.8411	0.4853	100	3991.696	3990.672		9129.4	1	7.359	22	3	
	K.TGEDK	6.6771	0.558	100	3992.692	3990.672		9717.4	1	8.408	22.2	3	
	K.TGEDK	6.3799	0.4995	100	5478.369	5476.356		11087.3	1	7.515	21	24	
	K.TGEDK	7.6413	0.416	100	5478.376	5476.356		10810.1	1	6.681	20.4	18	
	K.TGEDK	6.9852	0.508	100	5479.373	5476.356		6442.2	1	6.982	18.5	13	
	K.TGEDK	7.1219	0.3433	100	5607.465	5604.451		10124.8	1	5.854	19.4	12	
	K.TGEDK	6.3609	0.4315	100	5607.466	5604.451		11908.8	1	6.322	17.9	3	

K.TGEDK	4.8637	0.2896	100	5607.471	5604.451	10773.9	1	4.918	15.2	3
K.NADES	5.5707	0.3681	100	3461.453	3460.439	8710.5	1	7.396	26.8	3
K.NADES	5.0015	0.4359	100	4947.125	4946.122	8053.1	1	6.471	19.1	8
K.NADES	4.5503	0.383	100	4948.133	4946.122	7760.5	1	6.3	18.9	3
K.NADES	5.8199	0.3315	100	4948.135	4946.122	11090.9	1	5.489	18.9	3
K.NADES	4.8816	0.3085	100	5076.234	5074.217	9757.1	1	5.4	17.5	4
K.NADES	4.9091	0.328	100	5076.234	5074.217	7392.6	1	4.529	17.6	2
K.NADES	4.4412	0.3731	100	5076.235	5074.217	7158.1	2	6.087	15.5	3
K.VEDVTE	5.3972	0.3051	100	1507.712	1504.702	8580.5	1	7.913	87.5	33
K.VEDVTE	4.1843	0.2503	100	1632.8	1632.797	7897.1	1	6.05	76.9	3
K.KIHTVE	4.4092	0.3922	100	1671.828	1670.825	5557.1	1	6.432	83.3	5
K.KIHTVE	5.3483	0.3208	100	1792.906	1791.903	8238.4	1	6.686	59.6	13
K.KIHTVE	5.9275	0.4338	100	1798.922	1798.92	9152.5	1	7.92	76.9	45
K.IHTVEH	5.648	0.3774	100	1673.833	1670.825	6192.1	1	7.056	79.2	210
K.IHTVEH	3.6172	0.2839	99.9	1791.902	1791.867	4501.8	1	5.644	59.6	2
I.HTVEHE	3.4583	0.312	100	1551.728	1550.724	6348.6	1	5.007	63.6	3
M.RKPEEV	4.527	0.5001	100	1989.934	1989.956	7434.7	1	8.767	73.3	18
R.KPEEVT	4.9297	0.4988	100	1833.857	1833.855	6561	1	9.032	71.4	9
R.KPEEVT	5.4366	0.5041	100	1833.862	1833.855	8495.1	1	9.837	85.7	69
K.SLTNDW	4.0969	0.3694	100	1527.744	1527.744	7624.9	1	8.401	75	21
K.HFSVEG	2.8762	0.3435	100	1320.66	1320.659	5822.1	2	6.303	65	5
K.ALLFIPK	2.9266	0.3459	100	808.5433	808.541	2625	1	6.634	91.7	8
K.RAPFDM	3.5972	0.277	100	1282.602	1282.6	5167.5	1	6.424	66.7	45
R.VFIMDD	6.0412	0.4902	100	2421.211	2418.203	4433	1	7.704	61.1	76
K.GVVDSE	4.9106	0.3582	100	1521.807	1520.803	4641.5	1	8.623	76.9	3
K.KCLDMF	3.869	0.2214	99.9	2118.992	2118.984	7074.3	1	5.683	62.5	3
K.KCLDMF	5.3097	0.3898	100	2247.069	2247.079	6579.6	1	7.689	67.6	3
K.CLDMFS	4.161	0.4158	100	1990.884	1990.889	5925.6	1	6.575	60	6
K.CLDMFS	4.0578	0.4315	100	2118.985	2118.984	6502.5	1	7.341	59.4	3
D.MFSELA	4.0292	0.3426	100	1730.834	1730.842	6738	1	6.349	65.4	3
K.LGIHED	3.2874	0.2564	99.9	1145.579	1145.578	4904.9	1	6.124	77.8	7
K.SGDEMI	3.1087	0.3163	100	956.4835	956.4836	6907.4	1	6.028	93.8	2
K.DIYYIT	4.3532	0.4445	100	2255.132	2253.123	6923.4	1	7.002	50	35
K.DIYYIT	5.8853	0.527	100	2669.38	2666.387	8209.8	1	9.657	58.7	103
K.DIYYIT	5.3688	0.2975	100	2671.395	2666.387	10090.9	1	7.165	39.1	14
T.KKGFEV	4.7217	0.4186	100	2613.345	2613.364	10021.5	1	7.048	35.7	2

	K.KGFEVI	4.9871	0.3453	100	2499.295	2499.303		6187.8	1	6.594	38.8	4	
	K.KGFEVI	6.2946	0.4799	100	2500.311	2499.303		6617.9	1	9.18	65	67	
	K.GFEVIY	5.5688	0.4672	100	2372.214	2371.208		6908.1	1	8.542	65.8	61	
	E.VIYMVD	4.7528	0.454	100	2024.049	2024.041		6577.8	1	9.039	78.1	5	
	K.EGLDID	3.726	0.2913	100	1320.584	1320.581		4092.4	1	7.407	81.8	2	
	K.EGLDID	3.9199	0.3787	100	2183.045	2182.04		5731.5	1	6.756	50	21	
	K.AEYEGL	2.9119	0.2374	99.9	969.438	969.4351		2983.3	1	4.891	85.7	3	
	K.VIKDVL	2.986	0.364	100	1087.66	1087.659		6360.1	1	5.656	87.5	5	
	K.KIMEINA	3.1814	0.1994	99.9	989.5828	988.58		3817	1	5.04	92.9	1	
	R.HPIISAL	2.5292	0.3091	99.9	892.5867	892.5807		3238.7	1	5.827	85.7	1	
	T.SGFALE	4.4823	0.306	100	1415.696	1413.69		6061.2	1	5.842	79.2	10	
PFC0271c		63	52.30%	111	12418	7.9	U		Annotation|Plasmodium_falciparum_Sanger|(protein coc				
	K.IIEENII	4.756	0.4569	100	1388.857	1387.857		6512.7	1	9.237	90.9	39	
	K.GYNLNS	4.3328	0.3598	100	1791.86	1791.856		5184.9	1	7.655	64.3	5	
	K.GYNLNS	4.8295	0.2657	99.9	3157.52	3157.539		8184.4	1	5.384	31.7	2	
	K.DVVGG(	5.339	0.4722	100	1848.821	1848.829		6260.1	1	8.549	68.8	12	
	K.DVVGG(	4.2134	0.3625	100	1848.83	1848.829		6678.9	1	7.285	62.5	2	
	K.DVVGG(	4.5871	0.4038	100	2090.012	2090.008		5998.7	1	7.134	58.3	3	
PFI1565w		209	51.50%	171	19017	4.3	U		Annotation|Plasmodium_falciparum_Sanger|(protein coc				
	K.EDYDIE	4.9382	0.4741	100	1662.711	1662.71		6838.7	1	8.806	76.9	3	
	K.TINEGQ	4.2491	0.4472	100	2893.508	2892.497		8642.2	1	8.738	42.3	21	
	K.YQFINIE	3.1489	0.2598	100	1096.598	1096.598		2946.1	2	5.7	92.9	3	
	R.DLEFEG	5.116	0.5334	100	1879.829	1878.822		8923.5	1	9.719	80	21	
	K.VPGGNI	4.4587	0.4313	100	1651.902	1651.902		8042.1	1	7.98	67.9	8	
	K.VPGGNI	5.1694	0.4515	100	2174.124	2173.114		8507	1	8.481	63.9	30	
	K.VPGGNI	4.3431	0.3836	100	2183.142	2180.131		3175.4	1	7.866	45.8	31	
	K.VPGGNI	3.9859	0.3232	99.9	2567.328	2566.323		4548.7	2	6.454	32.6	2	
	K.VPGGNI	5.0139	0.3761	100	2567.329	2566.323		6451.8	1	6.603	33	26	
	K.VPGGNI	4.6455	0.2921	100	2567.33	2566.323		8415.4	1	6.754	47.7	12	
	V.PGGNIL	4.6035	0.4305	100	1552.835	1552.834		8253.1	1	8.035	80.8	52	
PF14_0517		583	51.40%	764	88503	6.8	U		Annotation|Plasmodium_falciparum_TIGR|(protein codin				
	K.NKIDVYI	3.4451	0.3436	99.8	2600.336	2600.321		7935.8	1	5.109	32.1	1	
	K.DKPILY	5.5281	0.3193	100	3225.68	3224.667		5519.8	1	6.189	38.5	20	
	K.DKPILY	5.0771	0.5091	100	3232.697	3231.684		9276.1	1	8.791	46.2	10	
	R.IDNRDE	5.9652	0.5083	100	2802.429	2802.421		5559	1	9.184	52.2	36	
	R.IDNRDE	5.0938	0.4647	100	2802.43	2802.421		9441.7	1	7.184	38	2	

R.KALLNA	3.2123	0.2028	99.8	1020.618	1017.61	4861.4	1	4.955	93.8	6
K.ALLNAY	2.9504	0.2784	99.9	889.5159	889.5147	3290.3	1	5.17	92.9	3
K.IIYNNN	5.7626	0.2627	100	3126.593	3125.58	8929.6	1	5.127	40	13
K.IIYNNN	4.3997	0.3477	100	3140.619	3139.614	3296.7	1	5.811	32.7	2
K.KDDENV	4.8087	0.2872	100	1677.901	1675.891	7895.1	1	5.575	92.3	9
K.DDENVL	3.4912	0.2225	99.8	1548.804	1547.796	9372.2	1	5.3	79.2	2
K.NVDNLL	6.3378	0.3366	100	2333.281	2330.261	10228	1	7.312	68.4	89
K.NVDNLL	5.6064	0.3353	100	2338.283	2337.278	8568.4	1	6.523	43.4	17
R.GYDYQY	4.0932	0.5429	100	2586.214	2585.203	7541.7	1	8.959	55.3	9
R.GYDYQY	4.0023	0.3311	99.9	3449.575	3448.569	4436.4	1	5.996	26.9	7
K.HNDDNF	3.0409	0.3762	99.8	1229.557	1229.555	4100.3	1	6.543	77.8	1
K.HNDDNF	3.5974	0.4301	100	1229.557	1229.555	6501.4	1	7.277	88.9	3
K.HNDDNF	3.255	0.3459	100	1229.557	1229.555	4770.9	3	5.845	52.8	3
K.KYDISLS	4.3538	0.4906	100	1982.104	1982.097	8738.3	1	7.878	53.3	6
K.KYDISLS	5.665	0.4695	100	1985.118	1982.097	6798	1	8.578	80	14
K.YDISLSF	5.3793	0.4722	100	1854.008	1854.002	4794.9	1	8.599	78.6	12
R.KNVLLQ	4.1905	0.3631	100	1338.815	1338.811	7091.6	1	6.921	86.4	15
K.NVLLQN	3.7059	0.2363	99.9	1211.722	1210.716	7417.9	1	4.977	85	6
K.QAHILD	3.4539	0.3434	100	2454.251	2454.239	6625.4	1	5.846	47.4	3
H.ILDGLA	4.1693	0.332	100	2125.117	2125.102	9993.5	1	6.347	56.2	6
K.NFIFPS	4.9978	0.4002	100	2995.473	2994.461	4354.6	1	7.539	35.6	8
K.NFIFPS	4.2535	0.4224	100	2995.473	2994.461	8615.4	1	8.002	34.6	32
F.PSFSTI	3.6782	0.418	100	2467.184	2466.18	7738.2	1	7.431	38.6	1
K.TNATIK	6.496	0.3992	100	2911.531	2905.506	6713.8	1	6.927	35.6	83
K.TNATIK	4.8989	0.4841	100	2920.55	2919.54	5527.9	1	8.089	53.8	49
R.TTHFGE	3.2907	0.3501	100	1502.725	1502.724	5529.3	1	6.503	75	3
T.LNVHEG	5.615	0.5549	100	1868.968	1868.977	7641	1	9.712	75	14
N.VHEGG(	2.8908	0.4011	100	1635.84	1634.832	4618.3	1	7.131	53.1	2
K.NMVLSN	4.1981	0.4088	100	1545.713	1545.708	7477.9	1	7.429	83.3	3
R.IENMQY	3.3277	0.1888	99.8	1224.633	1224.63	5478.6	2	5.63	77.8	1
K.EITDTT	5.0803	0.3802	100	2584.182	2581.166	8669.4	1	7.295	55	32
K.KLLDFS	5.6169	0.522	100	1668.968	1668.965	8172	1	9.278	92.3	30
K.LLDFSL	4.9159	0.353	100	1540.88	1540.87	7973.7	1	6.802	79.2	21
K.ELNEYH	3.1308	0.2954	99.9	1282.646	1282.651	4785.6	1	4.92	72.2	1
V.KQSPQE	3.1147	0.4166	100	1508.725	1508.723	5375.6	1	6.697	70.8	3
K.YLIEITE	4.2424	0.3628	100	1667.943	1667.938	6596.8	1	7.318	76.9	5

PF14_0359		482	51.40%	424	48469	7.2	U		Annotation|Plasmodium_falciparum_TIGR|(protein codin			
	K.LAIIHHP	2.5299	0.3213	99.8	1043.599	1043.6		6417.2	1	5.814	81.2	1
	K.LAIIHH	3.3816	0.2825	99.9	1286.705	1286.704		5261.7	1	5.647	68.2	5
	K.LAIIHH	4.4476	0.4235	100	1626.871	1626.86		6002.3	1	7.816	71.4	6
	A.IIHHPD	3.8949	0.2027	99.9	1442.76	1442.739		5326.7	1	5.804	79.2	2
	R.AYEVLS	3.3641	0.2243	99.9	1182.557	1182.553		5915.5	1	5.674	88.9	3
	R.AYEVLS	3.0763	0.2662	99.9	1338.655	1338.654		5560.9	2	5.606	75	3
	R.KLYDEY	3.2665	0.4294	100	3562.643	3561.655		5888.4	1	6.463	24.2	3
	R.KLYDEY	6.666	0.4363	100	3562.658	3561.655		9525.8	1	7.82	30.6	31
	K.LYDEYG	3.2331	0.442	100	3434.576	3433.56		8756.3	1	6.459	30	7
	K.KRGEDI	3.342	0.341	100	1266.708	1266.713		6466.3	1	5.998	75	4
	V.KVTLEQ	4.5118	0.3393	100	1464.808	1464.806		7198.4	1	7.831	87.5	9
	K.VTLEQL	3.9238	0.2651	100	1336.714	1336.711		7533.4	1	6.905	81.8	30
	K.VTLEQL	3.5442	0.3655	100	1464.811	1464.806		7656.4	1	6.549	75	8
	K.DIICTN	3.9925	0.4169	100	1571.716	1571.713		8374.7	1	7.548	84.6	5
	R.YHSSVL	4.544	0.4519	100	2092.943	2091.934		4870.8	1	7.981	46.9	6
	R.YHSSVL	5.6262	0.4468	100	2094.946	2091.934		8271.5	1	8.82	68.8	110
	S.SVLHQT	4.3195	0.4218	100	1704.788	1704.78		7683.9	1	7.835	80.8	3
	R.KIIEVYI	3.4207	0.1939	99.9	1102.686	1102.688		4225.3	1	4.749	81.2	15
	K.IVFNGE	5.223	0.4389	100	2746.539	2746.529		9848.7	1	8.157	37.5	18
	K.ISLYES	4.3547	0.2702	99.9	2281.167	2279.156		6479.8	1	6.866	36.8	10
	K.ISLYES	4.7794	0.5245	100	2289.212	2286.173		9035.5	1	8.657	68.4	128
	K.ILVNCT	3.844	0.3759	100	1407.764	1407.761		5676.1	1	6.827	77.3	10
	K.ILVNCT	2.655	0.4285	99.8	1407.765	1407.761		2445.1	1	7.328	45.5	1
	R.EVLDEG	3.4222	0.3658	100	1768.852	1768.847		6041.8	1	6.563	57.1	3
	R.EVLDEG	3.0834	0.3222	99.9	1896.948	1896.942		6024.2	1	5.974	53.3	3
	K.KYDLEN	5.6772	0.4673	100	2212.052	2211.049		7445.4	1	8.916	72.2	27
	K.KYDLEN	7.7768	0.5235	100	2752.36	2751.351		7422.9	1	9.222	39.8	31
PFF1025c		255	51.20%	301	33013	7.2	U		Annotation|Plasmodium_falciparum_Sanger|(protein coc			
	K.HGWCE	2.9697	0.4235	100	1060.47	1060.471		4919.2	1	7.743	85.7	24
	K.IAEEAG	5.7412	0.3063	100	2212.175	2211.169		5218.3	1	6.181	46.2	8
	K.IAEEAG	4.6521	0.3049	100	2213.179	2211.169		5071	1	6.074	62.5	37
	R.SVDPSK	3.5456	0.3201	100	1365.766	1365.77		5404.8	1	5.705	72.7	2
	R.IGHFVE.	4.6979	0.4375	100	1639.928	1639.925		8350.9	1	8.018	80.8	10
	K.IDMIDE	4.5293	0.4692	100	2454.169	2454.153		6182.7	1	8.231	50	12
	K.FKTPFV	4.1658	0.4167	100	1970.971	1969.963		6844.3	1	7.498	65.6	9

	K.TPFVCG	4.074	0.4726	100	1694.801	1694.799		3276.3	1	8.701	75	15	
	R.TKGEAC	4.8468	0.5062	100	1522.877	1522.874		6640.7	1	8.7	85.7	4	
	K.YLCSLS	4.6486	0.4385	100	1818.841	1818.837		7011.4	1	8.55	75	24	
	K.KINAPID	5.4351	0.4002	100	1451.929	1451.939		7093.9	1	7.018	62.5	11	
	K.KINAPID	5.023	0.3935	100	1451.94	1451.939		6379.3	1	7.86	83.3	34	
	K.KINAPID	3.3971	0.4475	100	1451.947	1451.939		6045	1	7.018	62.5	7	
	K.INAPIDL	3.522	0.433	100	1323.848	1323.844		5608.7	1	6.945	86.4	5	
	K.INAPIDL	3.7754	0.371	100	1437.909	1437.904		7025.1	1	7.053	66.7	14	
	R.KMAASI	4.172	0.3317	100	1785.953	1784.944		6472	1	6.237	48.4	3	
	R.KMAASI	6.2747	0.5104	100	1785.953	1784.944		7760.8	1	9.707	81.2	8	
	K.MAASIV	5.051	0.3076	100	1652.845	1649.832		8568.4	1	6.42	70	28	
PFF1050w		148	50.50%	184	20623	4.9	U		Annotation|Plasmodium_falciparum_Sanger|(protein coc				
	K.VEGTDS	4.2928	0.4222	100	1507.743	1507.739		7174.2	1	8.406	80.8	16	
	K.TDDITN	5.4796	0.4373	100	2068.081	2066.071		5097.5	1	6.452	58.8	49	
	K.DVDVPV	3.9996	0.3673	100	2409.163	2408.158		6083.4	1	6.852	45	16	
	K.VKDLDD	6.0898	0.3595	100	2803.32	2802.314		9709.4	1	7.384	39.6	17	
	K.VKDLDD	6.4822	0.5414	100	2803.321	2802.314		8618.2	1	10.297	62.5	10	
	K.DLDDEN	5.7403	0.4551	100	2576.161	2575.15		8236.7	1	7.972	43.2	9	
	K.DLDDEN	5.6736	0.5428	100	2578.164	2575.15		7408.1	1	9.807	56.8	25	
	R.KNNNDL	3.8849	0.3412	100	1668.839	1668.834		5856	1	7.07	53.6	6	
PF13_0058		81	50.30%	143	16526	4.1	U		Annotation|Plasmodium_falciparum_Sanger|(protein coc				
	C.GVNCTS	3.9425	0.2157	99.9	2056.039	2054.034		8438	2	5.849	50	6	
	C.GVNCTS	4.6016	0.4517	100	2063.058	2061.051		6227.6	1	8.104	48.6	3	
	G.VNCTSF	4.5343	0.2926	100	2004.03	2004.03		7016.9	1	6.647	52.9	7	
	C.TSRPSA	3.8861	0.3258	100	1630.887	1630.888		9504.7	1	6.862	60.7	5	
	T.SRPSAS	4.2002	0.2561	100	1529.841	1529.84		7412.6	1	7.205	76.9	7	
	R.PSASTI	3.8079	0.4253	100	1279.692	1279.69		8977.3	1	7.663	81.8	7	
	T.KAQHDV	3.885	0.4874	100	1600.795	1600.793		5655	1	7.771	69.2	3	
	T.KAQHDV	4.7814	0.4831	100	1600.795	1600.793		6799	1	8.605	84.6	13	
	K.AQHDVT	3.6083	0.5089	100	1472.699	1472.698		7346.6	1	8.621	83.3	9	
	K.TGVTME	3.7479	0.2912	100	1068.514	1068.515		4298.6	1	7.001	93.8	9	
	R.NSLQEF	3.0591	0.3176	99.9	1386.64	1386.636		7958.2	1	6.765	63.6	3	
	Y.FYEPG	4.8523	0.3702	100	2217.972	2216.968		4363.8	1	7.464	69.4	9	
PF11_0224		329	50.00%	162	17296	5.9	U		Annotation|Plasmodium_falciparum_TIGR|(protein codin				
	K.KGSGEF	5.1286	0.481	100	2067.092	2067.08		7181.5	1	8.701	43.1	34	
	K.KGSGEF	5.5057	0.5128	100	2091.124	2088.131		9429.8	1	9.376	66.7	37	

	K.KGSGEF	5.2711	0.3723	100	2196.178	2195.175	5559.1		1	7.109	42.1	5
	K.KGSGEF	5.4353	0.3898	100	2196.184	2195.175	6481.5		1	7.711	42.1	19
	K.KGSGEF	5.8758	0.5321	100	2219.237	2216.226	10255		1	9.307	63.2	17
	K.GSGEPL	4.7772	0.4469	100	1962.044	1960.036	6843.4		1	8.832	47.1	40
	K.GSGEPL	5.9837	0.5639	100	1963.053	1960.036	7708.2		1	10.272	76.5	122
	K.GSGEPL	5.6514	0.545	100	2091.147	2088.131	8006.9		1	9.625	61.1	34
	G.SGEPLI	4.103	0.3481	100	1903.018	1903.015	8136.7		1	6.559	62.5	3
	E.PLIDVHI	4.7098	0.5624	100	1629.921	1629.919	7185.8		1	9.248	80.8	6
	K.KEEELV	3.5383	0.2312	99.9	1216.644	1216.643	6450.9		1	5.301	88.9	4
	K.KEEELV	3.4635	0.2199	99.9	1372.747	1372.744	8918.8		1	4.966	90	3
	K.IGSSD	4.1009	0.4026	100	5160.223	5158.214	5564.4		1	5.655	16.7	2
	Q.DVTPEC	3.9984	0.4885	100	2151.948	2151.943	4534.2		1	8.413	60.5	3
PFF1335c		118	49.20%	189	20292	7.4	U	Annotation|Plasmodium_falciparum_Sanger|(protein coc				
	V.AVASGS	5.259	0.3973	100	2029.045	2029.057	11131.1		1	8.524	63.9	3
	A.VASGSE	4.3117	0.4868	100	1958.012	1958.02	8018		1	7.815	61.8	10
	K.VRNNIY	3.7521	0.3307	100	1802.031	1802.019	5048.7		1	6.027	60	1
	R.NNIYDV	4.6121	0.4382	100	1546.851	1546.849	8212.5		1	7.456	76.9	6
	K.GSNTIS	4.8853	0.4491	100	1844.869	1844.86	5875.7		1	8.316	66.7	12
	R.HSLIDD	3.8246	0.5331	100	1947.947	1947.945	5478.6		1	8.776	68.8	11
	R.HSLIDD	5.7662	0.4922	100	1954.97	1954.962	9264.8		1	8.787	65.6	60
	K.NCITSV	4.7208	0.3604	100	1743.887	1742.886	5332		1	7.778	71.9	6
	K.IVEHLL	2.9902	0.257	99.9	936.5659	936.5631	4475.6		1	5.307	85.7	9
PF14_0142		141	49.00%	304	34904	5.4	U	Annotation|Plasmodium_falciparum_TIGR|(protein codin				
	R.EIFLNQI	5.4357	0.3347	100	1980.145	1980.142	5301.6		1	8.153	78.1	13
	K.ICGDIH	3.6083	0.4306	100	1721.873	1720.867	8506.4		1	7.372	73.1	1
	R.LFEYGG	4.8718	0.4793	100	2569.216	2568.209	4014.8		1	8.494	64.3	30
	K.TFIDCFI	5.4823	0.4271	100	2129.041	2126.03	6866.5		1	7.76	61.8	28
	K.IFCMHG	5.6462	0.4038	100	2261.092	2260.086	10111.9		1	6.998	40.3	6
	K.IFCMHG	6.3922	0.4791	100	2261.093	2260.086	8053.8		1	8.482	77.8	18
	R.KITRPT	4.9411	0.4295	100	3953.932	3952.914	6912		1	7.079	27.3	2
	K.ITRPTD	4.7475	0.3639	100	2663.335	2661.331	8646.8		1	6.797	37.5	2
	K.ITRPTD	6.1548	0.4967	100	3839.861	3838.853	9421.4		1	9.097	27.3	9
	R.GVSFTF	4.5771	0.5	100	1822.929	1822.924	7664.5		1	9.096	66.7	15
	F.TFGQDV	2.9693	0.3755	100	1432.734	1432.734	6949.9		1	5.89	77.3	10
	R.KHELDL	3.086	0.2789	99.9	1190.644	1190.643	4447.2		1	4.679	87.5	6
	R.KHELDL	2.9063	0.3746	99.8	1190.644	1190.643	4690.5		1	7.022	75	1

PF10_0264		240	49.00%	263	29856	6.2	U	Annotation|Plasmodium_falciparum_TIGR|(protein codin					
	R.AKDGVI	3.5087	0.3399	100	1292.796	1292.788	7075.1	1	6.272	72.7	5
	K.FAQYTG	3.8442	0.424	100	1353.693	1353.692	6997.5	1	8.411	83.3	9
	K.ESAYAN	2.6455	0.406	99.9	3454.702	3453.704	6359.6	1	6.155	30	1
	R.SEPWN	5.0835	0.4311	100	1810.85	1809.846	8354.2	1	7.156	53.8	40
	R.SEPWN	4.7971	0.4665	100	1812.856	1809.846	4920.3	1	8.339	80.8	103
	R.SEPWN	3.1407	0.3325	99.9	2797.315	2796.317	6288.9	1	5.916	35.7	4
	E.PWNVM	4.272	0.1999	99.9	1594.801	1593.771	8946.8	1	6.931	90.9	12
	L.WRDPEC	3.4553	0.2359	99.9	1350.682	1347.67	5925	5	4.972	72.2	3
	R.DPEQFE	3.1779	0.1867	99.8	1005.489	1005.489	9546.7	2	5.405	92.9	8
	K.NLANEE	4.4512	0.3607	100	3430.59	3429.587	9699.7	1	7.651	32.8	14
	K.NLANEE	4.5089	0.3384	100	3437.61	3436.605	8244.9	1	6.101	27.6	4
	K.NLANEE	4.7457	0.3315	100	3559.691	3557.682	5231	1	5.272	23.9	2
	K.NLANEE	5.586	0.4358	100	3568.717	3564.7	6548.6	1	6.919	31.7	7
	K.KEEWN	4.0912	0.3693	100	1708.728	1707.725	5419.5	1	6.52	70.8	6
	K.KEEWNI	4.8176	0.4726	100	2619.148	2618.144	5121.9	1	7.464	36.2	6
	K.KEEWNI	4.2116	0.3474	100	2626.169	2625.161	8056	1	6.923	47.5	11
	K.EEWNDI	4.0219	0.4145	100	2498.066	2497.066	11978.9	1	6.793	44.7	5
		363	48.90%	593	59519	5.2	U	no description					
	K.HYSSS	4.0023	0.4361	100	2267.988	2266.997	4675.4	1	6.554	48	6
	R.SGGGG	4.6614	0.5343	100	1551.686	1549.678	5354.3	1	8.577	65.8	3
	K.GSLGG	5.4052	0.602	100	1708.778	1707.773	7718.3	1	10.662	66.7	42
	R.ALEESN	3.8458	0.3401	100	1382.653	1381.649	10137	2	6.761	72.7	10
	K.HGNSH(	3.7391	0.3978	100	1121.523	1118.509	5424.8	1	8.386	88.9	51
	K.TIDDLK	5.8371	0.3536	100	3054.642	3052.628	8374.9	1	6.812	34.6	24
	K.TIDDLK	4.9615	0.3585	100	3054.644	3052.628	7216.3	1	7.893	44.2	8
	K.NQILNL	6.0931	0.1488	99.9	2368.264	2367.263	9095.2	3	4.625	48.8	10
	K.NQILNL	5.7508	0.4099	100	2368.27	2367.263	10590.1	1	7.611	55	44
	R.LKYENE	3.2727	0.2526	99.9	1235.684	1234.68	3469.5	1	4.823	88.9	3
	L.RQSVEA	4.6248	0.3635	100	1359.724	1357.719	4921.9	1	6.628	86.4	2
	R.VLDELT	3.189	0.2988	100	1031.6	1031.599	3274.9	1	5.97	87.5	9
	K.ADLEM(	4.1655	0.4162	100	2225.149	2224.142	7137.9	1	7.026	61.1	2
	K.KNHEEE	2.9976	0.2907	99.9	1430.697	1428.691	7211.1	2	4.897	70	3
	R.NVSTG	5.6802	0.4964	100	2873.408	2872.393	4591.8	1	8.445	48.1	15
	R.SQYEQI	3.2289	0.3522	99.8	1493.735	1493.735	3132.7	1	5.806	68.2	1
	R.SQYEQI	3.3096	0.238	99.8	1493.737	1493.735	9786.8	1	5.691	68.2	2

<table>
<tr><td></td><td>K.ELTTEI</td><td>5.0759</td><td>0.5244</td><td>100</td><td>1996.977</td><td>1996.972</td><td>10068</td><td>1</td><td>8.985</td><td>78.1</td><td>12</td></tr>
<tr><td></td><td>R.NVQALE</td><td>4.4429</td><td>0.3312</td><td>100</td><td>1799.02</td><td>1797.012</td><td>8393.5</td><td>1</td><td>6.514</td><td>63.3</td><td>12</td></tr>
<tr><td></td><td>R.YCVQLS</td><td>6.0583</td><td>0.4938</td><td>100</td><td>2747.433</td><td>2746.42</td><td>4215.1</td><td>1</td><td>8.371</td><td>42</td><td>14</td></tr>
<tr><td></td><td>R.YCVQLS</td><td>6.369</td><td>0.4421</td><td>100</td><td>2749.427</td><td>2746.42</td><td>8762.3</td><td>1</td><td>8.021</td><td>61.4</td><td>64</td></tr>
<tr><td></td><td>R.AETECQ</td><td>5.0284</td><td>0.2476</td><td>100</td><td>2083.973</td><td>2082.965</td><td>3936.9</td><td>1</td><td>5.893</td><td>71.9</td><td>2</td></tr>
<tr><td></td><td>K.IRLENEI</td><td>3.8577</td><td>0.3343</td><td>100</td><td>1434.77</td><td>1434.771</td><td>9102.8</td><td>1</td><td>6.675</td><td>85</td><td>16</td></tr>
<tr><td></td><td>R.SLLEG</td><td>3.3426</td><td>0.3884</td><td>100</td><td>1265.616</td><td>1262.598</td><td>3927.7</td><td>1</td><td>6.748</td><td>73.1</td><td>8</td></tr>
<tr><td>PF11_0282</td><td></td><td>196</td><td>48.60%</td><td>173</td><td>19573</td><td>7</td><td>U</td><td>Annotation|Plasmodium_falciparum_TIGR|(protein codin</td><td></td><td></td><td></td></tr>
<tr><td></td><td>K.THHEGD</td><td>5.0669</td><td>0.509</td><td>100</td><td>2484.313</td><td>2484.321</td><td>8134.5</td><td>1</td><td>8.733</td><td>54.8</td><td>3</td></tr>
<tr><td></td><td>K.THHEGD</td><td>4.2737</td><td>0.3722</td><td>100</td><td>2485.322</td><td>2484.321</td><td>7013.3</td><td>1</td><td>6.449</td><td>35.7</td><td>3</td></tr>
<tr><td></td><td>K.KKDDDK</td><td>5.6706</td><td>0.5683</td><td>100</td><td>2237.206</td><td>2237.193</td><td>10648</td><td>1</td><td>9.747</td><td>44.4</td><td>13</td></tr>
<tr><td></td><td>K.KKDDDK</td><td>5.916</td><td>0.5078</td><td>100</td><td>2244.227</td><td>2244.21</td><td>7477.5</td><td>1</td><td>9.993</td><td>72.2</td><td>12</td></tr>
<tr><td></td><td>K.DDDKSN</td><td>3.754</td><td>0.3329</td><td>100</td><td>1988.021</td><td>1988.02</td><td>9318.7</td><td>1</td><td>7.378</td><td>62.5</td><td>1</td></tr>
<tr><td></td><td>K.SNIVNT</td><td>3.6978</td><td>0.3593</td><td>100</td><td>1507.82</td><td>1507.827</td><td>7941</td><td>1</td><td>6.702</td><td>79.2</td><td>5</td></tr>
<tr><td></td><td>R.GEIIAA</td><td>4.0061</td><td>0.4006</td><td>100</td><td>2038.044</td><td>2038.039</td><td>6195.9</td><td>1</td><td>6.318</td><td>52.9</td><td>4</td></tr>
<tr><td></td><td>K.LVQLVS</td><td>4.792</td><td>0.4966</td><td>100</td><td>2839.492</td><td>2837.446</td><td>6247.9</td><td>1</td><td>8.308</td><td>41.7</td><td>155</td></tr>
<tr><td>PF14_0545</td><td></td><td>54</td><td>48.10%</td><td>104</td><td>11716</td><td>4.8</td><td>U</td><td>Annotation|Plasmodium_falciparum_TIGR|(protein codin</td><td></td><td></td><td></td></tr>
<tr><td></td><td>K.RIAPFY</td><td>3.9992</td><td>0.323</td><td>100</td><td>1399.67</td><td>1399.668</td><td>8808.4</td><td>1</td><td>7.272</td><td>90</td><td>3</td></tr>
<tr><td></td><td>K.VDVDEV</td><td>4.5425</td><td>0.4046</td><td>100</td><td>1349.654</td><td>1348.648</td><td>6629.8</td><td>1</td><td>7.83</td><td>90.9</td><td>13</td></tr>
<tr><td></td><td>K.VDVDEV</td><td>4.7332</td><td>0.4771</td><td>100</td><td>2497.22</td><td>2497.202</td><td>9644.5</td><td>1</td><td>7.957</td><td>54.8</td><td>14</td></tr>
<tr><td></td><td>K.VDVDEV</td><td>5.1995</td><td>0.4012</td><td>100</td><td>2505.229</td><td>2504.219</td><td>6437.7</td><td>1</td><td>7.842</td><td>36.9</td><td>6</td></tr>
<tr><td></td><td>K.NGSSVD</td><td>5.0044</td><td>0.4202</td><td>100</td><td>1661.831</td><td>1661.835</td><td>4727.3</td><td>1</td><td>8.555</td><td>71.9</td><td>14</td></tr>
<tr><td></td><td>K.NGSSVD</td><td>3.2664</td><td>0.3443</td><td>99.8</td><td>1662.827</td><td>1661.835</td><td>4446.7</td><td>1</td><td>5.699</td><td>59.4</td><td>4</td></tr>
<tr><td>PF14_0083</td><td></td><td>73</td><td>47.70%</td><td>218</td><td>25051</td><td>10</td><td>U</td><td>Annotation|Plasmodium_falciparum_TIGR|(protein codin</td><td></td><td></td><td></td></tr>
<tr><td></td><td>K.YELGRF</td><td>2.9786</td><td>0.3302</td><td>100</td><td>1261.654</td><td>1261.654</td><td>6714.1</td><td>1</td><td>5.9</td><td>75</td><td>2</td></tr>
<tr><td></td><td>K.LDSGSF</td><td>4.2449</td><td>0.2108</td><td>99.9</td><td>1635.812</td><td>1635.813</td><td>6281.5</td><td>1</td><td>6.642</td><td>60.7</td><td>4</td></tr>
<tr><td></td><td>R.IIDVVY</td><td>4.6221</td><td>0.4049</td><td>100</td><td>1732.942</td><td>1732.942</td><td>7640.5</td><td>1</td><td>8.219</td><td>85.7</td><td>25</td></tr>
<tr><td></td><td>K.NCIVVI</td><td>4.2173</td><td>0.338</td><td>99.9</td><td>2915.428</td><td>2914.427</td><td>9615.7</td><td>1</td><td>5.948</td><td>30.2</td><td>1</td></tr>
<tr><td></td><td>K.NCIVVI</td><td>4.9399</td><td>0.4684</td><td>100</td><td>2918.436</td><td>2914.427</td><td>5399.9</td><td>1</td><td>8.1</td><td>43.8</td><td>33</td></tr>
<tr><td></td><td>I.VVIDSH</td><td>3.4578</td><td>0.3491</td><td>100</td><td>2520.255</td><td>2520.252</td><td>5688.7</td><td>1</td><td>7.074</td><td>47.6</td><td>1</td></tr>
<tr><td></td><td>V.VIDSHP</td><td>3.5558</td><td>0.3621</td><td>100</td><td>2422.187</td><td>2421.184</td><td>6235.8</td><td>1</td><td>6.486</td><td>47.5</td><td>1</td></tr>
<tr><td></td><td>K.KEEDNK</td><td>4.9134</td><td>0.3679</td><td>100</td><td>3079.408</td><td>3078.399</td><td>6363.5</td><td>1</td><td>5.744</td><td>30</td><td>3</td></tr>
<tr><td></td><td>L.ACISSR</td><td>3.4708</td><td>0.4271</td><td>100</td><td>1328.639</td><td>1327.632</td><td>6333.5</td><td>1</td><td>7.017</td><td>72.7</td><td>3</td></tr>
<tr><td>PF14_0368</td><td></td><td>313</td><td>47.70%</td><td>195</td><td>21807</td><td>7.2</td><td>U</td><td>Annotation|Plasmodium_falciparum_TIGR|(protein codin</td><td></td><td></td><td></td></tr>
<tr><td></td><td>K.YVLLYF</td><td>4.9546</td><td>0.4096</td><td>100</td><td>2940.517</td><td>2940.534</td><td>6168.8</td><td>1</td><td>7.916</td><td>39.1</td><td>49</td></tr>
<tr><td></td><td>K.GGIGNI(</td><td>5.6148</td><td>0.5192</td><td>100</td><td>1694.982</td><td>1694.981</td><td>10548.3</td><td>1</td><td>9.295</td><td>76.7</td><td>76</td></tr>
</table>

	K.GGIGNI(	3.7759	0.4153	100	1694.982	1694.981	6379.3	1	7.196	50	1
	K.GGIGNI(	4.252	0.3732	100	1695.987	1694.981	4932.1	1	5.917	45	11
	K.QGVVQI	3.3824	0.4082	100	1731.015	1731.003	6459	1	6.513	56.7	4
	R.IIDAVQ	6.1892	0.4702	100	2368.144	2368.144	7027.7	1	7.998	40.8	21
	R.IIDAVQ	5.2167	0.4335	100	2371.164	2368.144	9427.6	1	7.692	44.7	116
	I.DAVQHH	3.7504	0.4684	100	2128.945	2127.942	4699.8	1	7.232	50	9
	K.VAMKPS	3.4073	0.3261	99.9	1753.872	1753.868	5897.6	1	5.46	53.3	2
	K.VAMKPS	3.8825	0.461	100	1753.872	1753.868	6244.4	1	7.622	60	6
	K.VAMKPS	4.2759	0.2968	100	1756.88	1753.868	3510.1	1	6.516	70	15
	K.VAMKPS	3.2196	0.3893	100	1866.956	1866.952	5164.4	2	6.473	50	3
PFI0265c		687	47.50%	897	104856	6.7	U	Annotation|Plasmodium_falciparum_Sanger|(protein coc			
	G.KDVFAC	3.3247	0.3179	100	1111.617	1111.615	4994	1	6.746	77.8	18
	G.KDVFAC	3.3514	0.4387	100	1111.618	1111.615	5596.6	1	7.715	77.8	2
	G.KDVFAC	4.5694	0.3331	100	1239.704	1239.71	6146.3	1	6.512	67.5	3
	G.KDVFAC	3.2414	0.2625	99.9	1239.717	1239.71	5843.3	1	6.026	75	3
	K.GNGPD/	5.1503	0.3286	100	3479.664	3476.612	9080.7	1	5.722	26.7	42
	K.GNGPD/	3.0821	0.3328	99.9	3480.626	3476.612	7012.1	1	6.366	24.1	12
	E.PEQFYV	3.5983	0.3397	100	1956.974	1955.97	5505	1	6.29	60.7	3
	K.NIHNFT	3.3317	0.2908	100	1393.695	1392.692	5846.5	1	5.202	85	4
	R.SWVSEI	4.9802	0.3421	100	2459.209	2458.2	8354.6	1	6.533	55.3	50
	K.EYEEPF	3.18	0.3106	99.9	1481.702	1481.699	4495.7	1	6.491	72.7	9
	R.LFFTYN	4.5486	0.4002	100	1761.85	1761.849	10224.1	1	7.529	67.9	67
	K.TNIKDG	3.4632	0.3144	100	1445.75	1444.74	6718.7	1	5.768	70.8	1
	K.NLYSTV	3.5887	0.2813	99.9	1481.725	1481.724	8666	1	6.567	72.7	12
	K.VIIENE	3.6397	0.4058	100	2465.263	2465.261	5056.2	1	6.222	47.5	6
	K.HPSADL	3.6652	0.2837	99.9	2171.072	2171.06	6078.8	1	5.883	50	6
	H.PSADLC	3.9654	0.3476	100	2035.003	2034.001	4749.9	1	6.932	65.6	15
	M.TNSDFT	5.8716	0.4779	100	2950.591	2950.572	5552	1	7.711	39.6	5
	K.PQSVWI	3.8804	0.3306	100	1719.93	1719.93	7875.6	1	6.955	61.5	1
	K.PQSVWI	5.558	0.475	100	1932.051	1931.043	7528.6	1	8.251	51.7	17
	K.PQSVWI	4.7243	0.4858	100	1945.077	1945.077	5798.2	1	7.62	66.7	23
	Q.SVWNVI	3.5589	0.2397	99.9	1719.957	1719.966	4044.6	1	4.961	69.2	3
	S.VWNVPI	3.2654	0.3269	99.9	1618.912	1618.9	4023.2	1	5.889	79.2	2
	K.NYLDSV	5.3352	0.4625	100	1845.828	1845.833	9803.1	1	8.408	78.6	21
	K.NYLDSV	4.5351	0.3394	100	1973.922	1973.928	9557.6	1	6.799	70	15
	K.NYLDSV	3.2233	0.3516	99.8	1973.929	1973.928	5636.3	1	6.023	63.3	1

	K.NLIFLS	4.9957	0.2506	99.9	3157.644	3155.635	4801.9	1	6.019	40	5
	K.NLIFLS	4.5823	0.436	100	3158.672	3155.635	6866.2	1	6.813	38	68
	K.KSGAPI	3.447	0.3023	100	1170.695	1169.689	4615.3	1	5.668	72.7	2
	K.ELSHNI	3.2964	0.377	100	1437.702	1437.701	6699.5	1	6.048	77.3	9
	K.ELSHNI	4.0118	0.427	100	2910.351	2910.333	3080.4	1	7.063	29.3	3
	K.ELSHNI	4.1097	0.4436	100	2910.353	2910.333	5223.6	1	8.404	45.7	2
	R.YTSLYIH	2.4829	0.363	99.9	1031.564	1031.564	4334.7	1	5.814	71.4	1
	K.ASLHVP	3.8648	0.325	100	1241.694	1241.701	5180.4	1	6.615	60	6
	K.VFTALY	3.9865	0.3764	100	1564.807	1564.805	6399.7	1	7.671	79.2	11
	V.FTALYN	3.497	0.3242	100	1472.754	1472.754	6970.4	1	6.918	77.3	1
	K.NLEEIS	6.2036	0.493	100	2498.278	2497.272	4036.9	1	8.837	61.9	31
	R.KQDVL	6.5961	0.4573	100	3895.74	3893.73	8240.5	1	7.19	22.4	9
	R.KQDVL	4.8434	0.2801	100	3895.743	3893.73	8536.3	1	5.08	23	3
	R.KQDVL	7.4744	0.4926	100	3895.746	3893.73	9810.4	1	9.509	29.7	21
	Y.ETDKP	6.4043	0.5009	100	3148.338	3147.334	5629.8	1	8.612	28.8	18
	Y.ETDKP	4.6558	0.5237	100	3149.34	3147.334	5178	1	9.387	40.4	6
	E.TDKPQ	6.4097	0.499	100	3019.3	3018.291	5092.7	1	7.928	34	15
	E.TDKPQ	4.9883	0.4614	100	3019.301	3018.291	5346.7	1	7.061	26.7	3
	E.TDKPQ	3.9421	0.3559	100	3020.296	3018.291	7952.2	1	6.49	42	3
	K.QHSAHI	3.6621	0.4114	100	1473.758	1473.756	6062	1	7.136	70.8	14
	K.QHSAHI	4.2913	0.4258	100	2208.148	2208.149	7439.6	1	6.93	52.8	15
	K.TKDQDL	5.2376	0.3539	100	1501.786	1501.786	5954.1	1	8.009	86.4	31
	K.STSAAS	4.874	0.6065	100	3012.286	3011.28	7716.8	1	10.729	40	3
	K.STSAAS	6.2077	0.5395	100	3012.293	3011.28	6623.6	1	8.887	33.3	6
	K.STSAAS	6.5225	0.5698	100	3835.655	3834.651	7643.1	1	9.857	29.1	6
	K.TTDNTY	5.2675	0.4225	100	2533.142	2532.13	11343.1	1	8.401	50	12
	K.TTDNTY	5.7684	0.3942	100	2661.234	2660.225	10159.3	1	7.13	36.4	12
	K.EMEELE	6.0071	0.413	100	1709.763	1708.759	9970	1	7.447	89.3	18
	K.EMEELE	4.4167	0.3573	100	1836.855	1836.854	7788.7	1	6.326	73.3	12
PFC0295c		107	47.50%	141	15396	5	U	Annotation|Plasmodium_falciparum_Sanger|(protein coc			
	K.AVFDNV	3.3855	0.4839	100	1212.672	1212.67	8544.7	1	8.527	75	16
	K.NAHVHD	3.0567	0.2152	99.8	994.5238	990.5121	4484.3	1	4.831	81.2	2
	A.KVCFLS	5.2504	0.4136	100	1805.851	1802.846	8173	1	8.896	78.6	6
	K.VCFLSD	4.8582	0.2997	100	1677.758	1674.751	7932.3	1	7.373	84.6	15
	K.VCFLSD	4.6008	0.323	100	1805.857	1802.846	8934.7	1	6.513	82.1	9
	F.LSDVCS	2.935	0.3949	100	1396.679	1396.678	7007.9	1	5.79	63.6	3

	K.KLITTLC	3.0861	0.3261	100	1183.682	1183.683		5578.5	1	5.832	88.9	1	
	K.LITTLCA	2.7582	0.2606	99.8	1048.572	1048.571		3315.6	1	4.878	87.5	1	
	K.NIPLFM	4.2791	0.3594	100	1413.737	1412.732		4811.9	1	5.739	86.4	6	
	K.DLGHW/	3.2489	0.3293	100	1143.596	1143.595		6523	1	5.884	77.8	48	
PFC0870w		156	47.40%	156	17706	4.6	U		Annotation|Plasmodium_falciparum_Sanger|(protein coc				
	K.SSLIID	4.721	0.4269	100	2384.297	2383.291		5221.2	1	6.743	55	9	
	K.NITMEG	3.4661	0.2921	100	1257.648	1256.642		5176.9	1	5.656	85	7	
	K.TPFAFG	2.9795	0.3811	100	1027.56	1027.562		4504	1	6.421	87.5	15	
	K.LQVSCV	6.3309	0.4149	100	3775.931	3769.906		8207.3	1	7.161	28.9	48	
	K.LQVSCV	4.5655	0.5022	100	3792.971	3790.957		8358	1	9.015	34.4	65	
	Q.VSCVIV	4.5261	0.387	100	3550.823	3549.815		5801.6	1	6.3	22.5	1	
	V.SCVIVD	3.1515	0.3602	100	3451.753	3450.746		5118.1	1	5.947	31	1	
	V.IVDDLV	4.3804	0.4982	100	3104.616	3104.615		6061.4	1	8.115	36.5	1	
	I.VDDLVN	4.2701	0.4781	100	2985.515	2984.514		6179.3	1	7.208	44	7	
	V.DDLVNT	4.0819	0.4921	100	2886.445	2885.445		5315.4	1	7.061	43.8	2	
PF11_0250		101	46.90%	145	16012	5.2	U		Annotation|Plasmodium_falciparum_TIGR|(protein codin				
	K.IFPLAS	6.24	0.5417	100	2191.276	2188.264		6478.1	1	9.541	72.2	57	
	K.IFPLAS	6.0727	0.4886	100	2191.276	2188.264		7255.2	1	8.204	45.8	28	
	K.GISELV	4.6994	0.3835	100	3057.71	3057.728		9222.7	1	6.092	25	2	
	A.ADAKPL	3.9507	0.4766	100	2148.139	2148.138		6668.8	1	7.209	47.2	5	
	K.DGSPLE	4.8251	0.141	99.9	2386.245	2386.243		7874.2	1	4.603	52.5	9	
PF14_0102		651	46.40%	782	90053	7.1	U		Annotation|Plasmodium_falciparum_TIGR|(protein codin				
	R.SASAAA	5.4384	0.4593	100	2171.972	2171.965		8322.9	1	8.887	65.8	11	
	K.SASPHG	3.8865	0.4225	100	1734.745	1734.742		4765.5	1	7.7	56.2	6	
	R.SASVAG	3.5474	0.4038	100	1772.919	1772.914		6696.3	1	6.607	55.6	6	
	R.SASVAG	4.0805	0.5253	100	1774.922	1772.914		6619.3	1	8.537	58.3	7	
	K.NTLTPL	3.4381	0.3926	100	1978.022	1978.017		5813.7	1	7.314	59.4	3	
	K.NTLTPL	3.9624	0.3719	100	1979.027	1978.017		8936	1	7.106	59.4	5	
	K.NTLTPL	4.8461	0.457	100	2092.064	2092.06		5253	1	8.899	61.8	12	
	K.NTLTPL	4.932	0.4205	100	2383.21	2383.219		7860.5	1	7.439	65.8	39	
	K.YSLNNM	4.0666	0.3906	100	1694.848	1694.842		7984	1	6.513	73.1	4	
	K.NEGDLV	4.9517	0.5631	100	2417.053	2417.045		8699.8	1	9.509	55.3	62	
	K.NEGDLV	5.776	0.4637	100	2418.056	2417.045		9565.6	1	7.466	46.1	18	
	K.KTFSGI	3.3437	0.2998	99.9	1448.78	1448.786		8190.6	1	6.126	66.7	3	
	K.NMGVLD	3.8796	0.3769	100	1749.817	1749.815		4256.6	1	6.719	57.1	4	
	K.FGTCIG	3.9447	0.431	100	1624.778	1624.777		6120.1	1	8.533	73.1	8	

	F.GTCIGS	3.4452	0.4012	100	1477.708	1477.708	6639.4	1	8.106	75	1
	R.LYEFEN	3.3482	0.2983	100	1283.651	1283.652	5276.1	2	5.082	88.9	9
	K.FHPNID	3.4613	0.4656	100	1773.892	1773.893	6917.1	1	7.586	57.1	3
	K.FHPNID	4.8005	0.4136	100	1776.9	1773.893	3403.6	1	7.665	78.6	72
	K.NDIYEL	5.7229	0.4608	100	2076.021	2076.023	8687	1	8.339	71.9	86
	K.ENFDNF	4.9826	0.3933	100	3944.945	3943.955	6343	1	6.377	23.5	1
	K.LHVITS	3.3674	0.4921	100	1224.707	1224.707	5576.9	1	9.401	80	3
	K.LHVITS	3.4513	0.3927	100	1224.707	1224.707	4813.6	1	6.862	75	11
	K.IYSEIQ	4.7446	0.2997	100	1869.934	1868.928	9026.9	1	6.097	75	2
	K.LTWIYDI	3.2773	0.3557	100	1572.805	1572.814	5582.1	1	5.911	63.6	6
	K.RYYTAY	5.3318	0.4963	100	2339.086	2338.081	6716.9	1	8.854	66.7	42
	R.YYTAYA	4.8601	0.4574	100	2182.984	2181.98	7441.2	1	8.716	67.6	54
	Y.ALEGVC	3.311	0.4139	100	1520.705	1520.706	6927.2	1	7.768	70.8	6
	K.SQMYTE	3.8141	0.3888	100	1534.751	1533.749	8477.8	1	6.774	72.7	2
	K.NVIVYNA	5.6592	0.5339	100	1700.003	1697.996	8450.4	1	9.232	82.1	74
	R.HNFLLD	2.5325	0.3425	99.9	1319.619	1319.617	7922.7	1	6.051	83.3	3
	R.HNFLLD	3.5647	0.3425	100	1931.944	1930.957	6639.3	1	6.195	64.3	3
	K.YSTSHI	4.7711	0.417	100	2656.239	2653.232	5393.3	1	7.332	54.8	68
	K.YSTSHI	4.0823	0.3128	99.9	2810.325	2809.334	5341.8	2	5.025	29.5	7
	S.TSHIYF	3.932	0.4025	100	2404.139	2403.137	4933.3	1	7.133	63.2	9
	R.TMVTII	3.4867	0.2856	99.9	1558.793	1558.801	6805.7	1	5.257	75	1
PF11_0069		296	46.40%	276	32076	5.1	U	Annotation|Plasmodium_falciparum_TIGR|(protein codin			
	M.LMKDE	5.5232	0.3933	100	2318.994	2317.984	4022.4	1	8.067	61.1	3
	M.KDEDN	5.0421	0.3295	100	2074.872	2073.86	4824.2	1	6.244	48.4	3
	K.DEDNHD	4.7802	0.3526	100	1948.783	1945.765	5147.9	1	6.294	60	6
	R.IIKGDS	5.9028	0.2177	100	2461.333	2460.321	5786.9	2	5.124	46.2	7
	K.GDSMTF	5.3742	0.1948	100	2106.06	2106.058	7417.6	15	4.565	47.1	4
	R.EDLLQL	5.2001	0.2215	100	1461.799	1458.788	8952.4	1	5.365	86.4	193
	K.NNNYDF	4.5334	0.4581	100	1665.769	1662.759	10162.8	1	8.11	80.8	15
	K.TYNTID	3.5	0.3624	100	1129.607	1128.601	4960.3	1	6.719	83.3	8
	K.YQIEMP	4.6512	0.3733	100	2730.292	2729.281	5068.8	1	6.099	38.1	9
	K.VVESYH	3.4667	0.2738	99.9	1450.717	1448.706	4332.4	33	5.445	63.6	3
	K.IIEIHHF.	2.6962	0.2645	99.9	908.4993	908.4994	3138.8	1	4.446	91.7	4
	K.KIPNKN	2.963	0.2928	99.9	1302.75	1302.75	3868.3	1	5.516	80	1
	K.LIALVIQ	4.4062	0.3936	100	1355.872	1354.867	8047.5	1	7.91	91.7	29
	R.VNIQMQ	4.0375	0.3856	100	1320.687	1319.682	9645.6	1	7.053	90	11

PFI1090w		627	45.50%	402	44844	6.7	U	Annotation|Plasmodium_falciparum_Sanger|(protein coc			
	K.RGNFLF	3.7256	0.3764	100	2035.974	2033.968	6724	1	6.78	58.8	24
	F.TSESVN	3.1868	0.4002	100	1299.586	1299.582	5849	1	6.533	68.2	3
	F.TSESVN	5.4882	0.3446	100	3061.419	3059.405	8470.7	1	6.584	33.7	6
	F.TSESVN	4.4675	0.496	100	3080.466	3080.457	8482.7	1	8.779	36.5	6
	K.ICDQISD	5.7302	0.5363	100	1799.897	1799.893	6837.7	1	9.572	82.1	26
	K.HIGYDD	3.7192	0.4239	100	1647.781	1646.778	8173.7	1	6.56	69.2	1
	K.VSIDEQ	4.1483	0.3459	100	2111.007	2111.002	5275.5	1	6.419	38.2	4
	K.VSIDEQ	5.2216	0.5203	100	2112.009	2111.002	6599.2	1	9.428	70.6	17
	R.KLGILP	3.8113	0.2706	99.9	1370.795	1370.805	5651.1	1	6.494	54.2	3
	R.KLGILP	4.4523	0.4136	100	1370.813	1370.805	6752.6	1	7.306	87.5	16
	R.KLGILP	3.7815	0.516	100	1377.831	1377.822	5832.4	1	7.974	66.7	5
	K.LGILPY	3.5516	0.3803	100	1250.732	1249.727	3357.2	2	6.575	77.3	3
	K.GSCGGI	3.3765	0.3285	100	1182.568	1182.569	7176.6	1	6.149	75	25
	G.SCGGHI	3.157	0.2915	99.9	1125.551	1125.548	7016.6	1	5.731	77.8	1
	R.VHTVLI	5.2164	0.4837	100	1704.947	1704.947	9757.8	1	8.904	78.6	93
	V.HTVLIS	4.7053	0.4523	100	1606.88	1605.879	6343	1	7.987	84.6	3
	I.STQHAE	3.8424	0.4129	100	1689.849	1689.845	6093.8	1	6.6	65.4	2
	I.STQHAE	5.1634	0.4046	100	1699.875	1696.862	9796.2	1	7.142	69.2	7
	S.TQHAED	4.2604	0.4207	100	1609.836	1609.83	6350.3	1	6.764	75	1
	K.LLDNET	5.145	0.3758	100	1740.892	1739.886	8496.1	1	7.081	82.1	19
	G.KFVLGG	4.1749	0.3073	100	1530.848	1529.844	7073.4	1	6.891	73.3	15
	K.FVLGGF	4.2939	0.4349	100	1401.745	1401.749	7579.9	1	7.511	71.4	21
	K.FVLGGF	3.6888	0.2985	100	1529.844	1529.844	8046.6	1	5.421	66.7	1
	K.IICDTY	6.713	0.5805	100	2027.955	2024.948	8816.2	1	10.875	76.3	146
	R.NFDLRF	3.6907	0.3825	100	1703.969	1703.967	3206.5	1	6.911	53.8	11
	R.NFDLRF	4.7545	0.2743	100	1703.99	1703.967	7120.3	2	5.817	73.1	129
	K.TSAYGH	3.2988	0.4727	100	995.4626	995.4699	5637.7	1	8.057	87.5	39
PFL0210c		490	44.70%	161	17631	5.7	U	Annotation|Plasmodium_falciparum_Sanger|(protein coc			
	T.YPVQAG	2.7083	0.3031	99.9	1081.65	1081.648	5686.9	1	6.228	66.7	1
	K.AHIVGID	3.6317	0.4542	100	1298.717	1298.722	7585.4	1	7.519	81.8	19
	K.AHIVGID	3.6591	0.4147	100	1319.767	1319.774	5540.8	1	7.83	68.2	8
	R.KYEDIC	5.0714	0.4043	100	2140.037	2139.033	6678.6	1	6.684	44.1	14
	R.KYEDIC	4.6427	0.4113	100	2140.042	2139.033	8111.5	1	6.963	67.6	10
	R.KYEDIC	5.4854	0.4398	100	2296.143	2295.134	7055.4	1	7.341	48.6	1
	K.YEDICP	4.1845	0.4559	100	2010.937	2010.938	7598.5	1	8.125	65.6	4

	K.RTELQL	7.422	0.4617	100	3638.89	3637.867		9473.2	1	8.797	27.4	430
	Y.DNGDTK	3.6989	0.2439	99.9	1419.691	1417.681		5484.4	1	5.86	83.3	3
PFF0860c		469	44.70%	132	14122	10.3	U	Annotation|Plasmodium_falciparum_Sanger|(protein coc				
	R.VGAGAF	4.6346	0.4083	100	2974.591	2974.571		9607.6	1	6.219	25	6
	R.VGAGAF	4.1056	0.4241	100	2982.597	2981.588		7006.8	1	7.605	33.9	5
	V.GAGAPV	5.46	0.4687	100	2886.542	2882.52		9612.5	1	9.39	46.3	43
	A.GAPVYL	4.6843	0.3821	100	2754.468	2754.461		5363.4	1	8.305	46	5
	A.PVYLAA	6.1122	0.3271	100	2629.414	2626.403		9378.1	1	8.299	58.7	57
	Y.LAAVLE	3.0096	0.305	99.8	2261.232	2260.201		5588	2	5.097	40	2
	L.AAVLEY	4.7243	0.382	100	2147.105	2147.117		7406	1	6.045	44.7	5
	L.AAVLEY	4.9873	0.3777	100	2147.136	2147.117		8773.1	1	7.558	57.9	11
	A.AVLEYL	4.6599	0.3394	100	2084.108	2083.097		7613.1	1	5.714	38.9	10
	A.AVLEYL	5.978	0.3645	100	2087.105	2083.097		7794.5	1	7.473	75	162
	A.VLEYLC	5.2795	0.3681	100	2014.064	2012.06		8963.4	1	7.651	73.5	53
	R.NDEELN	3.091	0.3073	99.9	3213.749	3213.739		5664	29	5.304	22.4	2
	R.NDEELN	5.6997	0.3718	100	3213.754	3213.739		8324.6	1	6.954	31.9	11
	K.FLAGVT	4.5832	0.4517	100	2372.362	2371.362		6373.5	1	8.049	43.2	51
	L.AGVTFA	4.5472	0.4175	100	2107.216	2104.192		7090.8	1	6.618	52.5	15
	A.GVTFAS	4.5982	0.409	100	2040.161	2040.172		9548.3	1	7.385	55.3	25
	G.VTFASG	3.3987	0.3431	100	1984.132	1983.151		6909	1	6.016	41.7	2
	F.ASGGVL	3.8701	0.3263	100	1628.96	1628.949		4494.2	1	6.192	63.3	2
	A.SGGVLF	3.0618	0.3414	99.9	1564.929	1564.929		6984.9	1	5.695	60.7	1
	L.PNIHNVI	3.3085	0.2593	99.9	1151.701	1151.701		5497.1	2	5.585	83.3	1
PFL2215w		378	43.90%	376	41871	5.3	U	Annotation|Plasmodium_falciparum_Sanger|(protein coc				
	K.AGVAGD	3.6047	0.3601	100	931.4594	928.4489		4038.4	1	6.554	83.3	18
	K.DAFVGD	2.9636	0.3066	99.9	1180.55	1180.549		4800.5	1	6.173	90	3
	K.DAFVGD	3.6232	0.4175	100	1336.652	1336.65		3863.4	1	6.289	68.2	1
	T.LKYPIE	4.7332	0.3712	100	2188.066	2188.075		8887.9	1	7.666	73.5	4
	K.YPIEHG	4.7132	0.4548	100	1946.898	1946.896		7883	1	9.112	56.7	3
	K.YPIEHG	5.3916	0.4571	100	1961.932	1960.93		10155.3	1	8.75	76.7	16
	Y.PIEHGI	4.7814	0.5175	100	1797.87	1797.867		6938	1	8.602	64.3	5
	Y.PIEHGI	5.2972	0.5241	100	1797.879	1797.867		9296.3	1	8.675	82.1	5
	R.AAPEEH	4.9118	0.4944	100	1926.034	1926.034		6263.1	1	8.437	61.8	66
	R.AAPEEH	4.0544	0.5443	100	1926.036	1926.034		6721.3	1	9.247	61.8	4
	R.TTGIVL	3.8323	0.4237	100	2712.375	2712.371		7979.4	1	6.398	34	13
	R.TTGIVL	5.0893	0.5299	100	3191.654	3190.651		5551.8	1	9.36	39.7	84

	R.TTGIVL	8.0944	0.5975	100	3191.655	3190.651	8123.2	1	9.446	36.2	82
	R.TTGIVL	5.4773	0.4274	100	3191.656	3190.651	5230.7	1	7.258	27	6
	I.VLDSGD	4.4137	0.3729	100	2812.437	2811.433	5745	1	7.15	44	12
	L.DSGDG\	4.0891	0.4837	100	2601.287	2599.28	5846.2	1	7.511	43.5	7
	K.SYELPD	5.2511	0.4529	100	1790.917	1790.911	4841.2	1	9.149	83.3	17
	R.CPEALF	4.1884	0.2866	100	1494.749	1493.746	8055.7	1	6.385	75	9
	K.EAAGIH	3.9352	0.3786	100	1631.9	1631.894	4313	1	6.131	67.9	1
	K.DLYGNI	4.0835	0.4044	100	2246.08	2245.081	5391	1	7.317	50	15
	T.RDITTLA	3.4037	0.4562	100	1333.71	1333.715	5864	1	7.377	86.4	7
		262	43.60%	622	61987	5.2	U	no description			
	T.SGGGG(	4.3842	0.4504	100	1232.604	1232.598	6523.2	1	7.9	80	30
	R.GGGGS	5.7123	0.526	100	2706.162	2705.162	8150	1	9.392	33.1	9
	R.GGGGS	5.1712	0.5708	100	2706.165	2705.162	8374.8	1	10.788	41.9	21
	S.SLGGG	2.7122	0.3156	99.8	951.465	951.4648	3460.2	1	4.53	80	3
	K.VQALEE	3.4317	0.4314	100	1586.766	1586.766	4524.4	1	6.803	53.8	3
	K.VQALEE	5.4896	0.3655	100	1589.776	1586.766	10023.2	1	7.42	84.6	18
	K.NYSPYY	5.2159	0.4582	100	2903.42	2902.411	3313.3	1	8.185	32.3	10
	K.NYSPYY	4.2711	0.3058	100	2906.424	2902.411	8878.7	1	6.243	39.6	32
	Y.YNTIDD	4.8789	0.4907	100	2279.165	2278.157	7741.9	1	8.113	52.6	2
	R.QGVDAI	3.1795	0.3566	100	1157.591	1157.591	3991.2	1	6.798	75	6
	K.SDLEMQ	3.9408	0.2789	99.9	2172.012	2171.025	8074.7	1	5.444	64.7	6
	K.SDLEMQ	4.3141	0.3912	100	2301.129	2299.12	9365.9	1	6.842	61.1	3
	K.DIENQY	6.3158	0.4646	100	3265.518	3264.514	6446.5	1	7.912	36.6	26
	K.DIENQY	4.8751	0.4793	100	3267.524	3264.514	7607.7	1	8.239	37.5	22
	R.HGVQEI	4.0597	0.2468	99.9	1837.969	1837.966	7278.7	1	5.159	46.7	2
	R.HGVQEI	5.725	0.4442	100	1838.971	1837.966	10781.7	1	8.242	83.3	22
	R.YCGQL(	4.9428	0.5219	100	2737.312	2737.329	9116.4	1	9.624	50	8
	K.EIETYH	4.9286	0.3662	100	2511.136	2510.132	8101.4	1	7.53	54.5	14
	K.EIETYH	5.1745	0.2056	99.9	2511.139	2510.132	11117.2	1	6.756	42	4
	R.GGSGG	4.9329	0.383	100	1791.73	1791.728	6058.5	1	8.889	54.5	18
	R.GGSGG	4.653	0.4716	100	3223.254	3223.282	8967.1	1	7.356	20.5	3
PFI1445w		1010	43.50%	1364	161096	8.5	U	Annotation|Plasmodium_falciparum_Sanger|(protein coc			
	K.YLYMDE	3.0249	0.5079	100	1625.708	1625.705	6311.4	1	8.011	66.7	2
	K.YLYMDE	4.3956	0.4919	100	2202.009	2201.995	9554.6	1	8.619	67.6	21
	K.CHNVIG	2.6853	0.2808	99.8	1084.564	1082.553	4049.4	1	5.966	93.8	2
	K.EGNEFL	3.5548	0.363	100	1555.772	1555.773	7793.7	1	7.425	62.5	16

K.SLYGNN	5.0053	0.4376	100	2111.97	2110.964	7482.5	1	7.444	57.9	3
K.GSQNTE	4.7532	0.2881	100	2034.878	2034.874	5126.2	1	6.485	69.4	6
K.KVHDFL	5.0811	0.4438	100	2296.215	2296.23	6260.1	1	8.348	63.2	15
K.VHDFLV	5.91	0.3825	100	2171.144	2168.135	5294.2	1	7.576	75	131
K.LFEQIVD	4.0214	0.2325	100	1246.726	1246.723	8168.1	1	4.902	88.9	2
K.YFDEYF	3.1809	0.3668	100	1430.634	1430.627	8112.5	1	6.674	72.7	9
Q.PVCSAY	3.3879	0.3452	100	1462.693	1462.694	6501.4	1	6.291	72.7	15
K.KVFNGI	3.2189	0.2373	99.8	1348.765	1348.763	7829.1	1	5.539	77.3	1
K.HNHIVEI	2.2531	0.3369	99.8	911.4921	911.4925	3216.8	1	4.445	91.7	1
K.IDDINNL	3.7742	0.3946	100	1621.848	1621.849	6015.6	1	6.615	70.8	2
R.KYVTTL	2.4636	0.3295	99.9	953.567	953.5671	3348	1	5.668	85.7	5
K.HLINAD	6.4032	0.4514	100	2007.045	2004.038	8328.9	1	9.039	81.2	76
R.SNNIVAI	3.5369	0.4112	100	1247.737	1247.736	5713	1	7.449	85	8
K.KDFTQT	5.0366	0.2105	100	2175.133	2175.141	6940.5	1	7.685	69.4	9
K.DFTQTN	3.4619	0.3428	100	1918.95	1918.951	6176.5	1	6.697	53.1	4
K.DFTQTN	4.8379	0.4156	100	2050.054	2047.046	8094.3	1	6.783	61.8	15
K.EYENFV	4.0032	0.3531	100	2276.048	2275.045	6530.4	1	7.053	64.7	2
K.LWFDLL	3.9665	0.451	100	1446.731	1446.731	6111.7	1	7.931	90	44
L.WFDLLY	3.4731	0.3554	100	1333.648	1333.647	6616.5	1	6.402	83.3	6
K.FGWFYI	4.8743	0.4508	100	2100.107	2099.101	7196.2	1	7.545	65.6	76
F.GWFYIH	4.401	0.4099	100	1952.034	1952.033	6924.1	1	6.59	63.3	1
F.YIHPNEY	3.6019	0.3934	100	1561.862	1561.864	5396.7	1	5.933	70.8	1
K.NNDQLT	4.7652	0.5233	100	1620.822	1620.823	8028.3	1	8.645	76.9	31
K.IVEIINL	4.0187	0.3931	100	1505.911	1505.909	6303.2	1	7.255	75	1
K.SPDSLD	5.0192	0.3872	100	2005.992	2002.945	5202.4	1	6.565	56.2	31
K.NGYHVM	3.983	0.3103	100	1433.685	1433.685	5082.1	1	6.684	77.3	3
K.LLTSYE	4.9682	0.4887	100	2711.352	2707.337	6695.2	1	8.15	52.4	60
K.LLTSYE	4.1235	0.3177	99.9	3326.623	3324.611	6364.8	1	5.617	30.8	4
K.YNASYA	5.2103	0.5415	100	1894.898	1893.899	8424.9	1	9.95	76.7	21
K.QSLPII	3.3717	0.3228	100	1463.812	1463.82	3968.3	1	7.005	77.3	4
L.PIIDHVY	3.6656	0.3273	100	1135.645	1135.645	5313.1	1	6.505	81.2	5
K.NSQNYS	5.2916	0.4251	100	1804.858	1801.85	7295.2	1	8.338	75	7
K.QAAELL	3.2151	0.3762	100	1341.812	1341.818	5878.1	1	6.41	72.7	1
R.VTDAID	4.5735	0.4621	100	1723.886	1722.882	7401.9	1	7.65	80.8	10
K.ELETIL	4.6576	0.4115	100	2117.021	2117.019	6867.2	1	7.448	55.9	9
K.LINSYE	5.3143	0.5341	100	2276.032	2273.02	9187.8	1	8.696	65.6	30

	L.INSYEC	4.8502	0.5629	100	2160.942	2159.936	4967.6	1	9.439	80	23
	R.ILWMHA	3.256	0.3652	100	1893.982	1891.974	4640.1	1	5.436	65.6	2
	K.NFFSEL	4.1247	0.3736	100	1454.725	1453.723	7157.5	1	6.505	85	34
	D.YYSPM	3.9089	0.4607	100	1881.798	1881.79	5433.2	1	8.057	64.3	3
	S.PMVHSE	2.8736	0.3377	99.9	1468.622	1468.631	4961.3	1	6.393	77.3	3
	R.IHPNDF	3.3419	0.2542	99.9	1218.634	1218.634	3116.6	2	5.108	77.8	2
	R.LFVTEG	5.0071	0.3529	100	1755.968	1753.963	8023.8	1	6.802	89.3	78
	L.FVTEGT	4.3559	0.3876	100	1641.882	1640.879	7263.9	1	8.032	84.6	21
	K.INIPECF	3.1372	0.343	100	1436.674	1435.671	4268.9	1	5.285	63.6	3
	R.IYDMTI	3.54	0.3211	100	2286.195	2285.19	4438.9	1	5.22	50	1
	K.NIVSDA	4.3529	0.3989	100	1574.841	1574.839	7706.7	1	7.685	84.6	22
	K.STYLTE	5.1047	0.4813	100	2274.149	2273.145	5470.8	1	8.315	58.3	30
	K.STYLTE	6.1078	0.4437	100	3303.674	3302.67	4416.2	1	7.249	31.5	28
	K.STYLTE	3.5412	0.3114	99.9	3306.686	3302.67	7865.5	1	6.746	33.3	35
	L.TEPILT	3.4379	0.3062	99.9	2839.447	2838.443	6335.3	1	5.855	37	4
	E.PILTEE	4.1249	0.4303	100	2609.355	2608.353	5740.3	1	7.208	47.6	22
	I.LTEEHF	3.3201	0.3245	99.9	2392.203	2391.199	4895.9	2	5.293	39.5	3
	T.EEHFNL	3.2559	0.3127	99.8	2179.075	2177.067	4836.2	1	5.975	47.1	6
MAL13P1.56		831	43.40%	1085	126062	7.6 U		Annotation|Plasmodium_falciparum_Sanger|(protein coc			
	G.SVVNNE	3.2558	0.3325	99.9	1651.74	1651.735	5640.2	1	6.533	57.7	6
	R.MKPFEE	3.6607	0.3975	100	1715.846	1715.843	5507.4	1	6.811	57.1	1
	R.MKPFEE	4.2886	0.2309	100	1722.853	1722.86	4768.3	1	5.803	71.4	10
	N.SNNVEN	3.8845	0.3552	100	1758.842	1758.837	6052.5	1	6.566	66.7	6
	K.HNVGED	4.131	0.4941	100	1598.819	1598.818	5873.3	1	8.299	64.3	12
	K.HNVGED	4.9739	0.5479	100	1598.819	1598.818	7619.6	1	10.68	89.3	42
	K.LVEGEE	5.5726	0.514	100	2304.091	2304.104	9566.8	1	9.936	63.9	29
	K.FAFSSE	4.4349	0.3838	100	2514.298	2514.311	6420.1	1	6.815	47.6	90
	R.ITFFID	3.7104	0.4032	100	1584.793	1584.792	7530.3	1	7.057	66.7	13
	K.YPVLLS	5.1606	0.4604	100	2211.155	2211.152	8311.8	1	9.442	57.9	4
	R.FNDPHL	3.3327	0.3056	100	1127.53	1127.531	5089.7	1	6.15	93.8	6
	R.FNDPHL	2.8567	0.3351	99.9	1403.684	1403.678	8559.8	1	5.616	75	3
	R.FNDPHL	3.3977	0.2566	99.9	1550.737	1550.747	6476.4	1	5.935	72.7	9
	K.HLSATY	2.716	0.2826	99.8	1040.586	1040.585	3479.3	1	6.377	68.8	3
	K.LQWALE	3.1742	0.2256	99.8	1288.711	1288.709	5139.9	1	5.691	88.9	3
	K.GLNIFN	4.0498	0.3558	100	1463.814	1461.807	6664.6	1	6.943	76.9	3
	R.ILTVVG	3.8602	0.4299	100	2028.027	2027.021	8088.9	1	7.326	56.2	15

	R.LSHVDL	2.7685	0.2384	99.8	952.5577	952.558	3836.3	1	5.608	92.9	2
	R.SVQFLE	5.8	0.4869	100	3975.896	3973.892	7640.7	1	7.42	25	3
	K.ADNSAN	4.5514	0.4528	100	2824.391	2824.369	7247.2	1	7.855	37.5	100
	K.ADNSAN	4.9982	0.4734	100	2825.371	2824.369	6163	1	7.12	30.2	20
	K.QYSIHV	3.3679	0.3611	100	2099.029	2099.027	4576.1	1	5.971	59.4	2
	Y.SIHVNQ	3.879	0.4043	100	1800.89	1800.888	7031.4	1	8.331	57.1	2
	Y.SIHVNQ	4.8126	0.521	100	1800.896	1800.888	8593.9	1	8.921	71.4	8
	K.KPLFIP	3.2072	0.3528	100	1958.162	1957.179	7882.5	1	5.964	47.1	1
	K.PLFIPI	4.317	0.3563	100	1809.035	1808.032	5548.5	1	7.097	65.6	11
	K.PLFIPI	4.3686	0.5728	100	1829.08	1829.084	5027.9	1	9.319	59.4	1
	F.IPISVGL	3.9576	0.3765	100	1450.829	1450.827	4544	1	7.316	69.2	2
	K.ESDTFV	3.417	0.367	100	2138.134	2138.117	8337.9	1	5.915	47.2	5
	K.ESDTFV	4.0972	0.3415	100	2295.225	2294.219	4493.8	1	5.969	39.5	4
	K.ESDTFV	4.9116	0.5456	100	2308.26	2308.253	6873.4	1	8.654	47.4	117
	R.GFSAPV	3.4454	0.3861	100	1954.907	1954.903	8162.7	1	6.946	53.1	2
	K.LESFNL	5.3822	0.5323	100	2034.117	2034.11	4305.8	1	8.407	64.7	24
	K.YLLEDP	3.7417	0.2204	99.9	1476.723	1475.717	7013.6	1	5.052	87.5	6
	R.YIINFV	4.941	0.5133	100	2055.072	2055.084	8503.4	1	8.644	67.6	128
	K.ADDLTY	3.7146	0.4355	100	2563.069	2563.051	8408.7	1	6.421	45	13
	S.KAQYPN	4.984	0.4279	100	1897.004	1897.018	7817.6	1	6.842	45	6
	S.KAQYPN	3.1327	0.3077	99.9	1897.021	1897.018	4759.8	1	6.185	63.3	2
	K.AQYPNI	3.196	0.3581	99.9	1768.925	1768.923	6355.4	1	6.385	60.7	2
	K.SPYPSN	4.5137	0.4648	100	2162.037	2162.045	4134.7	1	7.469	50	64
	K.LSKDDE	3.9698	0.3753	100	1729.941	1729.938	7735.3	1	5.977	69.2	2
	K.LSKDDE	5.7132	0.2997	100	1729.943	1729.938	11754.2	1	6.533	76.9	43
	K.TDKFNP	4.3663	0.3392	100	2025.983	2025.978	6107.9	1	6.556	71.9	6
PFI0930c		790	43.10%	269	31826	4.4	U	Annotation|Plasmodium_falciparum_Sanger|(protein coc			
	K.IENDDP	6.1215	0.4919	100	2342.088	2339.076	4309.1	1	8.555	72.2	33
	K.IENDDP	3.9242	0.3997	100	3281.555	3279.544	5027.7	2	6.067	30.8	10
	K.IENDDP	5.2565	0.4756	100	3310.626	3307.613	4253.1	1	6.987	30.8	21
	K.CAHEQN	3.8172	0.4186	100	1265.587	1265.584	6156.7	1	7.254	88.9	9
	K.IPGFWA	3.2262	0.2541	99.9	1181.66	1181.654	3511.6	1	6.135	88.9	20
	I.PGFWAN	3.6239	0.3243	100	1061.558	1061.553	5759.2	1	6.438	93.8	15
	R.KHPALS	5.0564	0.4969	100	2387.349	2387.36	7266.4	1	9.044	50	24
	R.KHPALS	5.47	0.4847	100	2387.351	2387.36	5347.7	1	7.231	47.5	13
	K.HPALSD	5.04	0.4881	100	2239.217	2238.213	7364.4	1	8.672	36.8	4

	K.HPALSD	4.4454	0.4953	100	2260.292	2259.265	6080.8		1	8.417	52.6	17
	V.PEDIDIL	4.3971	0.3756	100	1419.809	1419.804	8415		1	7.602	81.8	6
	K.AKEFME	3.3058	0.3492	100	1405.777	1405.777	7092.8		1	7.226	72.7	9
	K.EGKNPI	3.7105	0.3123	100	1415.775	1413.768	3272.7		1	6.499	70.8	2
	K.NPIAAV	3.8223	0.4333	100	1099.608	1099.609	5663.8		1	7.17	94.4	101
	K.NPIAAV	3.6022	0.4279	100	1099.61	1099.609	3103		1	7.487	72.2	10
	N.PIAAVT	2.9241	0.3233	100	978.5482	978.5485	5208.9		1	6.686	93.8	7
	K.WSIFEW	5.529	0.4684	100	2839.421	2838.418	8351.9		1	8.793	50	301
	K.WSIFEW	5.7644	0.5276	100	2840.423	2838.418	6089		1	8.276	39.8	44
	W.SIFEWF	4.3157	0.4372	100	2652.34	2652.338	7911.4		1	8.278	45.2	9
	S.IFEWFT	3.2727	0.4693	100	2565.311	2565.306	4929.8		1	7.176	45	1
	I.FEWFTT	4.903	0.4021	100	2447.216	2445.205	9148.9		1	7.596	63.2	39
	F.EWFTTD	4.2896	0.3397	100	2298.137	2298.137	7278.6		1	7.293	52.8	22
	E.WFTTDE	5.0503	0.49	100	2170.099	2169.094	7282		1	8.904	64.7	37
	W.FTTDEL	4.7462	0.4367	100	1983.015	1983.015	7104		1	7.999	65.6	34
	F.TTDELQ	3.5476	0.3321	100	1830.944	1828.929	4662.1		1	5.672	63.3	2
PFL2060c		350	42.90%	459	52339	7	U	Annotation|Plasmodium_falciparum_Sanger|(protein coc				
	K.ECILSG	3.7266	0.4369	100	1483.764	1483.769	8574.1		1	7.685	75	10
	K.ILVLDR	4.7563	0.3475	100	3316.733	3314.742	7559.5		2	5.489	24.1	2
	R.NPYYGC	4.4393	0.5251	100	2381.148	2380.146	5536.6		1	8.402	52.5	15
	R.NPYYGC	5.0452	0.4834	100	2605.306	2605.294	8737.9		1	8.678	54.5	109
	R.NPYYGC	4.2018	0.3341	100	2606.304	2605.294	5451.6		5	5.815	28.4	4
	R.HWNVD	2.9366	0.3571	100	1128.63	1128.628	4489		1	5.829	81.2	2
	K.VPATDM	5.4931	0.4974	100	2044.066	2044.071	7339.9		1	8.872	61.1	55
	K.VPATDM	4.5472	0.222	99.9	2045.08	2044.071	7661.4		1	5.454	45.8	3
	K.NFYQYV	4.1903	0.3583	100	1819.843	1819.84	6230.1		1	8.096	76.9	20
	K.SPFIYP	5.0609	0.5679	100	2084.106	2084.104	5837.6		1	9.361	66.7	53
	K.VICDPS	3.4383	0.3574	100	1461.717	1461.723	4996.1		1	6.472	77.3	3
	R.CICILS	4.2008	0.4573	100	3480.793	3480.796	4664.5		1	7.119	33.9	7
	K.SDIYIN	4.3233	0.4716	100	1948.074	1948.073	7948.6		1	7.726	59.4	5
	K.KPADNI	6.8131	0.6161	100	4141.015	4139.999	6537.7		1	10.145	27.1	16
	T.SSYDAT	5.4137	0.384	100	3156.488	3154.476	10450.8		1	7.981	46.2	19
	T.SSYDAT	5.66	0.431	100	3162.503	3161.493	9086.1		1	7.019	28.8	23
	S.SYDATS	4.5241	0.3907	100	3076.477	3074.461	8840.6		1	6.574	29	4
PFL1070c		1019	42.40%	821	95018	5.4	U	Annotation|Plasmodium_falciparum_Sanger|(protein coc				
	C.DSSVE	4.8218	0.5639	100	2096.878	2096.874	7634		1	10.021	62.5	21

C.DSSVE	4.878	0.4247	100	2225.984	2224.969	8751	1	7.162	39.3	11
C.DSSVE	5.5902	0.4513	100	2227.987	2224.969	8059.2	1	8.935	52.4	51
R.DRDTLE	6.1587	0.3435	100	3467.568	3466.556	8938.2	1	6.747	29.2	8
R.DRDTLE	7.5307	0.5337	100	3467.57	3466.556	6968.5	1	9.677	33.9	6
R.DTLEEI	5.8947	0.4444	100	3196.439	3195.428	7881.6	1	8.271	35.6	3
R.LMDIIV	5.1435	0.3935	100	1554.874	1551.864	9417.9	1	8.01	79.2	40
R.ELISNA/	4.1156	0.3023	100	1276.676	1273.664	7388.5	1	6.719	86.4	13
R.FLSLSD	2.893	0.393	99.8	1552.779	1552.775	5519.4	1	6.576	57.7	2
R.FLSLSD	4.6385	0.3025	100	1555.786	1552.775	6942.4	1	6.257	92.3	27
K.VDLINNI	4.2742	0.423	100	1284.773	1284.771	5132	1	7.542	86.4	1
K.SGTSNF	3.7317	0.3688	100	1253.638	1253.638	5965.7	1	7.064	81.8	6
K.NNDDE(	4.2888	0.513	100	1905.819	1905.822	5156.2	1	8.406	60	5
R.ISLHLK	4.7631	0.307	100	1959.098	1959.099	7683.7	1	6.274	46.9	1
K.YSQFIQ	3.3967	0.3218	100	1682.924	1682.913	8866.4	1	5.704	62.5	2
K.YSQFIQ	4.9376	0.4896	100	3266.718	3264.713	9183.2	1	8.193	38.5	144
K.YSQFIQ	6.3388	0.4445	100	3266.724	3264.713	6386.9	1	7.768	33.7	57
Q.FIQFPI	4.0933	0.4211	100	2889.575	2886.559	8416.5	1	7.241	45.7	38
F.IQFPIY	4.4497	0.4286	100	2739.49	2739.491	7589.4	1	6.853	45.5	42
I.QFPIYL	3.8001	0.249	99.9	2621.396	2619.39	7345.8	1	5.221	42.9	2
Q.FPIYLL	5.2037	0.3611	100	2492.335	2491.331	7853.8	1	8.496	65	44
F.PIYLLH	6.1334	0.5372	100	2344.264	2344.263	7341.4	1	9.98	60.5	16
R.RVLVAD	4.3016	0.2893	100	1689.933	1689.933	7440.1	1	6.394	80.8	21
R.VLVADE	3.894	0.3059	100	1533.84	1533.832	5711.6	1	6.011	83.3	22
V.LVADEF	3.8218	0.3461	100	1434.765	1434.763	7843.4	1	6.908	68.2	7
R.SELENE	3.5368	0.2233	99.8	1578.753	1578.75	4349.2	2	5.5	62.5	3
D.TYIEHM	3.1688	0.2733	99.9	1396.705	1396.701	6657.8	2	5.598	70	1
K.IPQLQIF	2.6398	0.3026	99.9	1000.639	1000.638	2892.5	4	5.588	78.6	1
K.NIDVLF	3.7683	0.4653	100	2139.036	2138.044	6726.1	1	7.206	58.8	2
F.LTESVD	2.7376	0.4172	99.8	1443.688	1443.686	4712.4	1	6.507	50	1
K.ALIDVIS	4.0915	0.4529	100	1201.725	1201.723	6431.4	1	7.917	85	14
K.ILEINPN	4.2612	0.2924	100	1873.072	1873.062	5199.6	1	6.857	63.3	28
K.ILEINPN	3.7483	0.3472	100	1901.13	1901.131	4386.2	52	5.429	36.7	1
E.INPNHP	4.1016	0.2404	100	1517.842	1517.851	4577.2	1	6.306	79.2	12
E.INPNHP	3.5454	0.3162	99.8	1538.908	1538.903	5293.3	1	6.394	62.5	1
K.LASGFD	6.335	0.4519	100	2677.324	2676.316	6424.6	1	8.465	43.5	63
K.LASGFD	5.3031	0.5496	100	2690.354	2690.35	9730.7	1	9.283	47.8	83

	A.SGFDLE	6.7066	0.5133	100	2507.237	2506.229		10793.4	1	9.799	57.1	105
	A.SGFDLE	6.7903	0.4335	100	2508.235	2506.229		5797.8	1	7.881	39.3	46
	A.QIVYDH	3.3351	0.3553	100	1271.693	1271.694		4076.1	1	6.602	83.3	3
	K.LGVDNN	3.6765	0.2993	99.9	2248.165	2246.156		6027.2	1	5.49	44.7	4
	K.IDDLDPS	4.8305	0.4131	100	1407.731	1406.724		4298.5	1	6.375	90.9	12
	K.KIEDEN	7.7598	0.4995	100	2924.453	2923.443		4848.4	1	8.905	43.5	40
	K.KDNNVD	3.8832	0.3076	100	1836.795	1835.79		6824	1	5.9	66.7	3
	K.DNNVDN	4.6184	0.2521	100	1709.701	1707.695		8434.1	1	7.27	75	6
PFL1720w		61	42.30%	442	49780	8	U		Annotation|Plasmodium_falciparum_Sanger|(protein coc			
	K.YDKELF	3.8859	0.2462	100	1412.727	1412.731		7815.7	1	5.123	90	18
	R.QIETIN	5.5376	0.5309	100	2100.119	2100.13		7431.5	1	10.679	69.4	11
	K.YSEGYF	3.2441	0.3343	100	1136.551	1136.549		4527.9	1	6.555	87.5	12
	R.YYGGNI	4.7303	0.4561	100	2128.96	2128.954		7033.4	1	8.32	59.4	3
	R.ALEAF	4.1658	0.3013	99.9	3761.936	3759.923		6388.4	7	5.021	19.3	1
	R.NLALSF	3.1479	0.2805	99.9	1017.574	1017.573		4550.9	1	5.716	87.5	6
	R.EICDEV	4.2228	0.3511	99.9	4793.284	4790.276		6584.1	1	5.816	17.7	2
	N.NPFTYA	3.8854	0.382	100	1694.833	1694.839		6484.4	1	7.999	75	3
	K.INSSVF	3.1701	0.344	99.9	1837.926	1836.911		5091	2	5.229	46.9	1
	K.NTIPSD	3.8523	0.4114	100	1730.859	1730.857		5565.3	1	6.42	63.3	1
	K.DMEFIA	3.2148	0.2442	99.9	1332.661	1332.666		8799.6	1	5.731	80	3
PFI0645w		135	42.00%	276	32027	5.1	U		Annotation|Plasmodium_falciparum_Sanger|(protein coc			
	K.GEEDYH	4.2098	0.4174	100	3085.334	3084.329		5626.5	1	9.204	42	33
	K.GEEDYH	5.5769	0.503	100	3085.337	3084.329		8502.6	1	8.011	33	15
	H.HISSLP	5.9352	0.353	100	2328.156	2327.149		4998.4	1	6.817	43.1	1
	M.SCIIYDD	7.003	0.5087	100	2094.039	2094.037		7853	1	9.684	81.2	36
	K.IENIDL	3.5643	0.2504	99.9	1460.695	1460.694		6507.7	1	5.799	77.3	1
	K.IENIDL	4.2084	0.2995	100	1588.792	1588.789		8474	1	6.32	79.2	5
	K.IENIDL	4.499	0.3299	100	1859.958	1858.951		8240.1	1	6.328	71.4	10
	K.RTLILG	5.9633	0.4416	100	4553.179	4551.173		6389.6	1	7.348	23	1
	R.TLILGL	6.7963	0.4639	100	4396.08	4395.071		6906.7	1	9.105	28.4	15
	R.TLILGL	5.5247	0.4184	100	4493.125	4487.104		6752.3	1	7.637	26.3	18
PF11_0062		196	41.90%	117	13125	10.3	U		Annotation|Plasmodium_falciparum_TIGR|(protein codin			
	K.SRYDSY	3.9111	0.3161	100	1519.772	1518.771		6224.5	1	5.825	77.3	112
	R.YDSYGL	2.9326	0.3165	99.9	1275.637	1275.638		7076.6	1	6.53	83.3	1
	L.KQVHPD	3.2437	0.3293	100	1237.672	1237.665		6202.1	1	6.363	75	5
	R.KSMNIM	4.5527	0.4082	100	1903.933	1903.93		5310.3	1	7.635	70	17

	K.SMNIMN	4.7309	0.3922	100	1783.856	1782.852		9463.2	1	7.827	82.1	60	
	I.RLVLPGE	2.7657	0.3106	99.9	1095.695	1095.689		5745.1	1	5.786	72.2	1	
PFA0345w		54	41.70%	168	19599	4.9	U		Annotation|Plasmodium_falciparum_Sanger|(protein coc				
	R.NELNEE	3.6585	0.312	100	1493.797	1493.793		8344.6	1	5.756	86.4	1	
	K.IISDVD	4.89	0.4251	100	2774.38	2772.365		9795.2	1	7.046	39.6	2	
	K.IISDVD	4.7851	0.4833	100	2808.44	2807.451		8913.7	1	8.571	45.8	26	
	K.ELGENI	6.8147	0.6028	100	3899.731	3898.713		5747.3	1	10.287	25	25	
PF11_0061		60	41.70%	103	11456	11.2	U		Annotation|Plasmodium_falciparum_TIGR|(protein codin				
	K.RISGLIY	3.2828	0.2987	100	1369.81	1369.81		5587.7	1	5.307	80	15	
	R.ISGLIYE	3.8071	0.3261	100	1192.659	1192.658		4336.2	1	5.975	88.9	7	
	K.VFLENV	5.5131	0.4166	100	2138.108	2137.1		6406.8	1	7.871	52.9	9	
	K.DSIMYT	3.1664	0.3672	100	1195.55	1194.547		5044.1	1	6.321	83.3	2	
	R.KTVTAM	3.5777	0.4625	100	1468.812	1468.809		8713.7	1	9.064	83.3	8	
	K.TVTAMD	3.4932	0.3567	100	1347.734	1347.731		6404.7	1	6.273	72.7	7	
	K.TVTAMD	3.4423	0.3921	100	1496.817	1496.815		7065	1	7.293	75	12	
MAL13P1.257		59	41.00%	156	18691	4.7	U		Annotation|Plasmodium_falciparum_Sanger|(protein coc				
	R.IKAELE	3.6307	0.1994	99.9	1206.73	1206.728		4641.5	1	5.929	94.4	4	
	R.LFCDDE	4.9623	0.4379	100	1918.945	1917.94		7227.7	1	7.876	84.6	30	
	R.KTDILEI	3.779	0.1761	99.9	1299.748	1299.746		8584.7	9	4.923	65	1	
	K.YSTINF	2.8429	0.3076	99.9	1194.636	1193.628		5563.1	1	6.166	83.3	1	
	R.GIELID	5.8233	0.4833	100	2222.165	2219.154		4797.6	1	8.177	66.7	23	
PF10_0370		27	41.00%	105	12320	8.6	U		Annotation|Plasmodium_falciparum_TIGR|(protein codin				
	K.LALNGI	3.4197	0.3158	99.9	1733.933	1733.926		7637	1	6.037	50	4	
	R.ITYNMN	5.3083	0.3999	100	2108.111	2108.104		9869.4	1	7.976	59.4	17	
	K.LVDHIKI	3.7566	0.3735	100	1279.756	1279.756		5694.1	1	6.133	75	6	
PFL2225w		44	40.70%	204	23492	4.3	U		Annotation|Plasmodium_falciparum_Sanger|(protein coc				
	T.WGPGFI	3.8287	0.5147	100	1591.721	1591.718		6681.3	1	7.431	79.2	3	
	K.ISIDNA	3.3092	0.3803	100	1237.637	1237.636		5784.1	1	7.677	75	1	
	R.KLGLAP	4.0536	0.3681	100	1264.722	1264.723		6827.2	1	6.726	86.4	2	
	R.KLGLAP	3.9385	0.3457	100	1392.818	1392.818		6932.5	1	6.458	66.7	1	
	K.LGLAPS	3.31	0.3442	100	1136.629	1136.628		3110.5	2	5.68	75	3	
	K.MFAHFD	4.6536	0.4396	100	1819.799	1818.794		3820.2	1	7.419	78.6	15	
	K.NILTTW	4.8891	0.4455	100	3465.671	3464.665		7912.4	1	6.895	28.3	5	
	K.NILTTW	4.6023	0.5538	100	3465.676	3464.665		7288.6	1	9.459	31.7	14	
PFL0740c		11	40.70%	91	9774	5	U		Annotation|Plasmodium_falciparum_Sanger|(protein coc				
	K.EGDVVV	3.5664	0.3054	99.9	1648.848	1648.843		4264.9	1	6.712	63.3	3	

	K.EGDVV\	3.6864	0.4391	100	1648.85	1648.843		3678.9	1	8.612	66.7	3
	K.IDGEEFI	4.7435	0.3975	100	2536.307	2536.295		6552	1	6.525	41.2	3
	R.DDDIIGI	3.3024	0.3078	100	1273.693	1273.69		7708.3	5	5.018	65	2
PFL0185c		578	40.40%	359	41997	5	U		Annotation|Plasmodium_falciparum_Sanger|(protein coc			
	K.MTDLTE	4.2453	0.3202	100	1696.88	1693.868		9208.8	1	5.965	73.1	6
	K.EYYDYE	3.755	0.3751	100	2308.095	2307.086		7657.1	1	7.258	56.2	13
	K.FEYELF	3.776	0.2637	100	1232.667	1229.657		4867.5	1	5.6	93.8	90
	R.QKYHDL	3.7087	0.3986	100	1639.815	1639.812		7041.7	1	6.954	83.3	15
	K.YHDLYG	3.8219	0.2428	100	1390.675	1390.676		5852.5	2	5.601	70	6
	K.YHDLYG	3.8473	0.3933	100	1390.678	1390.676		6187.3	1	7.34	75	2
	K.YHDLYG	4.7268	0.3468	100	1546.776	1546.777		5828.9	1	6.602	70.5	7
	K.YHDLYG	3.4102	0.4566	100	1546.779	1546.777		4913	1	6.947	68.2	1
	K.YHDLYG	4.5123	0.4007	100	1548.783	1546.777		6387.1	1	6.658	86.4	38
	K.IGTPNL	3.1634	0.2894	99.9	1445.791	1442.78		6012	1	5.672	72.7	27
	T.PNLPEF	3.1454	0.2483	99.9	1171.626	1171.626		3907.4	1	4.67	93.8	9
	R.NNNTVS	4.9745	0.4526	100	2872.442	2872.438		8430.7	1	6.777	28.3	3
	R.NNNTVS	6.3397	0.5501	100	2872.444	2872.438		7687.2	1	9.611	47.8	35
	R.NNNTVS	7.6387	0.5292	100	2873.436	2872.438		8523.9	1	8.989	39.1	233
	F.ILSFYF	4.7012	0.4223	100	2197.146	2196.138		6302.3	1	7.522	63.9	3
	L.SFYFAT	5.5012	0.4793	100	1970.975	1969.97		5508.6	1	7.981	78.1	24
	S.FYFATN	3.1814	0.3249	99.9	1883.946	1882.938		6281.5	1	5.411	60	3
	F.YFATNP	5.3828	0.3851	100	1736.873	1735.87		4503.7	1	8.418	78.6	12
	Y.FATNPF	4.3736	0.4579	100	1572.81	1572.806		4714.1	1	8.686	76.9	15
	K.CVDCDI	5.5253	0.4591	100	2914.317	2913.315		5615.3	1	7.849	40.2	24
	K.HEVAQL	4.6877	0.5381	100	2422.285	2422.284		4194	1	8.834	60	8
	K.HEVAQL	4.3842	0.3998	100	2422.287	2422.284		6615.8	1	6.981	43.8	4
PFE0810c		41	40.40%	151	16077	10.5	U		Annotation|Plasmodium_falciparum_Sanger|(protein coc			
	K.VKTPQF	3.9956	0.4138	100	1776.989	1776.986		5657.3	1	7.05	59.4	12
	K.TPQPET	4.4461	0.3567	100	1550.83	1549.823		4883.8	1	7.774	75	18
	K.TPQPET	3.3129	0.487	100	1556.841	1556.84		4273.5	1	8.322	57.1	1
	K.ADRDE	4.9585	0.5326	100	2226.995	2225.992		6335.6	1	8.826	65	3
	K.ELGVTA	2.9142	0.3365	100	1094.677	1094.676		3172.1	1	6.011	72.2	1
	K.SKTPGF	2.7616	0.391	99.9	1269.695	1269.692		3993.7	1	5.972	62.5	3
	K.TPGPGA	3.1219	0.2904	99.9	1054.566	1054.565		6072	1	6.783	80	3
PFI1475w		1334	40.20%	1720	195725	6.5	U		Annotation|Plasmodium_falciparum_Sanger|(protein coc			
	C.VTHESY	3.2256	0.3665	100	1332.677	1332.68		7805.5	1	6.44	80	12

K.KLEALE	4.8083	0.3486	100	2025.078	2025.091	7561.3	1	6.835	47.1	3
K.KLEALE	6.1575	0.4849	100	2025.098	2025.091	7172.6	1	9.445	82.4	24
K.MVLNEE	3.4839	0.3886	100	1306.658	1306.657	6802.9	1	6.266	85	2
R.SNTSSG	2.8345	0.3726	99.9	1764.758	1764.752	5504	1	6.352	58.3	3
K.ELKYPE	4.0876	0.3913	100	3052.513	3048.496	4523.2	1	6.237	41.7	15
K.YPELFD	6.6176	0.457	100	2687.3	2685.292	8103.6	1	8.249	45.2	39
K.YPELFD	4.994	0.383	100	2689.332	2685.292	6802	1	7.06	52.4	23
Y.PELFDL	5.629	0.3872	100	2523.232	2522.229	8227.9	1	6.904	41.2	2
K.YLIDGYI	5.233	0.4519	100	1874.93	1874.943	9465.8	1	8.798	85.7	11
K.LNFYFD	3.1076	0.3194	100	1200.633	1200.642	5490.3	1	5.667	93.8	17
K.LNDVCA	4.2647	0.4049	100	2090.981	2090.975	6616.3	1	7.255	53.1	6
R.ANELDV	3.0444	0.1929	99.8	901.5007	901.4995	5537.9	1	4.493	92.9	1
R.KPLDNI	3.5438	0.2638	99.9	1340.749	1340.754	2844.6	1	5.051	52.3	2
R.KPLDNI	4.0205	0.3768	100	1340.755	1340.754	4257.5	1	6.582	72.7	10
R.KPLDNI	4.9168	0.4513	100	1347.772	1347.771	7144.5	1	8.434	86.4	26
N.KTTIANI	4.7188	0.5283	100	1630.895	1630.902	8064.2	1	8.742	82.1	3
K.TTIANIN	3.8198	0.446	100	1523.865	1523.858	8676.9	1	7.448	65.4	7
K.TTIANIN	4.2733	0.4619	100	1651.954	1651.953	8062.7	1	8.275	67.9	14
D.KINEIK	4.2791	0.4187	100	2475.338	2475.321	5773.5	1	6.497	52.3	10
D.KINEIK	7.2955	0.4715	100	2476.325	2475.321	8848	1	8.922	44.3	11
K.NPPPAN	3.8113	0.4068	100	1749.879	1749.877	6048.9	1	7.01	56.2	1
K.NPPPAN	4.3556	0.3851	100	1750.879	1749.877	3097.3	1	6.207	71.9	21
K.FNIDSL	4.5189	0.4303	100	2255.123	2255.135	9423.7	1	6.857	42.6	1
K.FNIDSL	6.6762	0.5019	100	2255.14	2255.135	8019.3	1	10.128	70.6	143
F.NIDSLF	3.9049	0.3328	100	2102.054	2101.049	9460.8	1	6.212	50	2
K.VPYPNG	3.9078	0.3511	100	2293.231	2293.223	5528.5	1	6.009	47.5	2
K.VPYPNG	3.9828	0.474	100	2836.459	2836.452	7242.2	1	7.346	40	7
K.VPYPNG	6.8187	0.5189	100	2850.49	2850.486	8332.1	1	8.932	37	11
K.VPYPNG	4.5813	0.3444	100	3966.94	3964.918	7739.6	1	5.461	20.5	2
K.VPYPNG	6.2251	0.408	100	4196.051	4194.061	7765.2	1	6.322	21.6	12
K.VPYPNG	5.893	0.5066	100	4209.091	4208.095	9517	1	8.432	20.3	9
K.NSYGDL	3.4479	0.4161	100	1376.628	1376.627	5092.3	1	7.177	68.2	6
K.NSYGDL	2.69	0.4252	99.8	1376.63	1376.627	3509.1	1	6.23	59.1	1
R.KIFINNI	3.3496	0.157	99.9	1010.668	1010.666	3209	1	4.97	92.9	1
K.FNNNFN	3.2795	0.3313	100	1453.709	1453.708	7355.1	1	6.642	86.4	3
K.GISEKD	3.8963	0.3133	100	1715.848	1714.844	5487.3	1	5.818	76.9	4

K.DFNHYY	2.7664	0.2845	99.9	1200.563	1200.569	6820.4	1	5.166	81.2	3
K.DSIHVP	4.0764	0.4315	100	2139.075	2139.087	5042.1	1	6.286	41.2	4
K.DSIHVP	4.1793	0.3922	100	2139.091	2139.087	4468.7	1	6.171	61.8	20
K.DSIHVP	3.6685	0.3163	100	2302.164	2302.151	4969.2	1	6.141	55.6	2
K.DSIHVP	4.4618	0.3909	100	2415.224	2415.235	5263.7	1	7.042	63.2	18
K.DSIHVP	3.9015	0.2518	99.9	2528.337	2528.319	5171.3	1	6.016	50	4
T.ITLPPT	2.8907	0.2826	99.8	1275.726	1275.731	6187.8	1	5.331	77.3	1
T.ITLPPT	2.9884	0.3358	99.9	1638.947	1638.954	4160.8	1	5.591	60.7	1
K.LLEVYN	5.5149	0.157	100	1934.987	1932.981	5638.9	1	5.956	70	26
K.SCDPLD	5.5508	0.4718	100	4739.262	4738.25	5522	1	7.45	20.4	10
Y.SLYDSM	4.0039	0.349	100	2506.18	2505.176	4936.4	1	6.777	52.6	21
K.TKVPNP	3.8697	0.3252	100	1795.952	1795.959	5281.1	1	6.693	73.3	3
K.NNDTYF	3.6012	0.4003	100	1365.604	1365.604	5621.9	1	7.327	85	1
K.LNSLNN	3.2223	0.315	99.9	1362.717	1362.713	6448.4	1	6.144	81.8	3
K.EAEIAE	3.9575	0.4456	100	1691.8	1691.798	3110.3	1	7.872	53.6	6
K.EAEIAE	5.0741	0.4337	100	1694.807	1691.798	7393.6	1	7.588	82.1	5
K.TLSEVS	4.8617	0.406	100	2061.011	2061.01	7134.4	1	7.944	73.5	6
H.HLITELK	3.0086	0.174	99.8	860.5286	860.5319	5172.2	5	4.904	91.7	1
K.SYENFL	3.1606	0.2697	99.9	1197.576	1197.579	6056.4	1	6.548	77.8	7
K.VTTVVT	4.0358	0.427	100	2091.156	2090.15	5013.2	1	7.371	60.5	13
E.AISVTM	4.0381	0.3111	99.9	3392.723	3390.73	6377.1	5	5.464	21.6	2
K.NIFTFN	3.539	0.3143	100	1922.031	1922.032	8290.1	1	6.266	60	1
K.HISSNEY	4	0.4057	100	1702.86	1702.859	6357.2	1	7.878	61.5	3
K.HISSNEY	4.2609	0.3521	100	1702.869	1702.859	6044.3	1	7.036	76.9	7
A.KYKDDL	3.3443	0.3352	100	1245.683	1245.68	6987.1	1	6.076	88.9	1
A.KYKDDL	4.1982	0.2046	100	1366.752	1366.758	8005.9	1	5.91	90	11
A.KYKDDL	4.5726	0.2245	100	1366.764	1366.758	5200.8	1	5.934	57.5	2
K.YKDDLE	3.4863	0.1994	99.9	1238.658	1238.663	4940.3	1	5.994	88.9	8
K.EKFPSS	2.6831	0.4573	99.9	1667.854	1667.865	3241.4	1	6.594	43.3	1
K.EKFPSS	4.0286	0.2836	100	1667.854	1667.865	6351.7	1	7.086	63.3	101
K.EKFPSS	3.3649	0.4876	100	1667.867	1667.865	3843.5	1	7.925	53.3	5
K.FPSSPP	3.9546	0.23	99.8	1412.733	1410.727	6020.9	1	5.864	76.9	24
F.PSSPPT	3.0948	0.3136	99.9	1263.663	1263.658	6752.2	1	5.981	66.7	7
K.FLPFLTN	5.9714	0.3267	100	2154.171	2153.165	5338	1	6.198	51.5	26
K.FLPFLTN	6.3705	0.3932	100	2160.192	2160.182	6713.2	1	8.095	70.6	374
F.LPFLTNI	5.4835	0.4433	100	2014.116	2013.113	8290.8	1	8.235	78.1	9

	L.PFLTNIE	6.2213	0.4539	100	1904.044	1900.029		8319	1	8.742	83.3	69
	L.TNIETLY	4.217	0.4216	100	1542.829	1542.824		8679.9	1	7.762	83.3	2
	K.INDCNV	4.4346	0.4279	100	1677.801	1677.798		7171.9	1	7.897	76.9	22
	K.AIDDKID	3.4116	0.2909	100	1177.645	1177.647		6806.4	1	6.459	83.3	12
	F.KNPYDF	3.468	0.2378	99.9	1352.714	1352.721		6478.6	1	5.222	75	4
	K.NPYDFE	3.1275	0.3047	99.9	1224.628	1224.627		6150.4	1	5.396	83.3	6
	S.TGLVQN	3.8419	0.4244	100	1545.882	1545.883		8093.2	1	7.203	65.4	1
	K.FQDMLN	4.127	0.2945	100	1755.843	1754.843		6272.4	1	6.11	76.9	4
	K.KQCPEN	2.8431	0.3269	99.9	1382.597	1382.595		5648.2	1	6.135	75	6
	R.HLDERE	2.7747	0.3221	99.9	1215.543	1215.543		7936	1	5.825	87.5	1
	K.CVENP	4.6058	0.6516	100	3417.266	3417.26		6879.8	1	10.846	38.3	3
PF10_0063		44	40.20%	107	11977	9.3	U		Annotation|Plasmodium_falciparum_TIGR|(protein codin			
	R.MFTGNE	5.0316	0.4521	100	2625.296	2625.287		8542.8	1	8.223	45.7	12
	F.TGNEEK	5.0394	0.44	100	2361.217	2361.212		9469.5	1	8.14	54.8	6
	K.NPFDEV	5.1636	0.4088	100	1702.916	1702.92		10542.3	1	7.453	70	9
	K.NPFDEV	4.2926	0.4617	100	1702.922	1702.92		4153.1	1	9.63	60	8
	K.IAIGAAS	5.2702	0.4894	100	1986.087	1986.095		8234.4	1	8.372	66.7	9
PF11_0183		679	39.70%	214	24875	8	U		Annotation|Plasmodium_falciparum_TIGR|(protein codin			
	K.YIPTLG	3.5951	0.414	100	1465.847	1465.842		3244.3	1	6.791	83.3	52
	I.PTLGVE	3.2234	0.3787	100	1189.693	1189.695		8183.7	1	7.755	75	46
	K.TQFNVW	4.0891	0.4565	100	1523.718	1523.713		5615.5	1	7.775	83.3	7
	K.RNLQYY	3.7103	0.3098	100	1399.716	1398.713		4421.8	1	5.409	75	14
	R.NLQYYD	3.0287	0.2672	99.9	1242.615	1242.612		7561.5	1	5.617	83.3	3
	R.LSNQPN	5.0243	0.4224	100	1652.882	1652.876		5639.9	1	7.15	55.4	6
	R.LSNQPN	4.8656	0.1949	99.9	1655.886	1652.876		4704.5	1	6.195	75	36
	L.SNQPNL	3.6012	0.4019	100	1539.811	1539.792		5244	1	6.804	76.9	6
	S.NQPNLV	3.9381	0.4316	100	1452.761	1452.76		4472.1	1	7.467	79.2	48
	K.APEFQI	2.9423	0.3864	99.8	1428.809	1428.804		3028.5	1	6.044	63.6	1
	K.APEFQI	4.622	0.4616	100	1428.809	1428.804		7257.1	1	7.432	81.8	128
	A.PEFQIDI	3.1909	0.2934	99.9	1357.743	1357.767		6281.7	1	5.709	85	1
	R.EAEKEL	4.5524	0.33	100	2317.084	2316.073		5299.1	1	6.496	46.2	9
	R.EAEKEL	5.6059	0.4622	100	2333.123	2330.107		4528.4	1	9.069	55	288
	K.ELEQAA	3.8628	0.3944	100	1872.895	1872.89		7519	2	6.232	43.8	1
	K.ELEQAA	4.7391	0.4498	100	1875.888	1872.89		5913.8	1	6.834	62.5	33
PF10_0068		255	39.40%	246	29530	9.1	U		Annotation|Plasmodium_falciparum_TIGR|(protein codin			
	R.VYVGNL	2.7217	0.3219	99.9	1075.59	1075.594		3259.7	15	5.521	75	4

	K.GCGIVE	5.1971	0.4543	100	2634.202	2634.188	8930.2	1	7.316	34.8	15
	K.GCGIVE	6.4396	0.6048	100	2648.213	2648.222	10746.1	1	10.589	52.2	47
	K.GCGIVE	3.9344	0.4837	100	3147.463	3147.443	8088.8	1	8.03	38.9	20
	K.GCGIVE	5.7092	0.5524	100	3161.487	3161.477	6235.5	1	8.546	31.5	6
	R.EDREEN	3.1533	0.2998	99.9	1453.622	1453.62	4434.3	30	5.716	54.5	6
	R.NCTLIV	3.5739	0.3102	99.8	1946.023	1946.021	5593.2	1	6.229	46.7	2
	R.NCTLIV	5.1754	0.5135	100	1953.049	1953.038	4761.7	1	8.848	70	96
	K.ELIGVV	3.592	0.3659	100	1736.868	1736.878	9144.2	1	6.928	67.9	2
	K.NAIDAL	3.6963	0.4001	100	1987.96	1987.959	6452.4	1	7.026	53.1	5
	K.NAIDAL	6.0091	0.4534	100	2005.013	2001.993	9683.8	1	9.533	71.9	52
PFD1055w		93	39.40%	170	19722	10.2 U	Annotation|Plasmodium_falciparum_Sanger|(protein coc				
	K.RLLEPV	3.0152	0.3528	100	1197.736	1197.736	5649.5	1	5.909	77.8	12
	R.KLAPLN	4.145	0.4463	100	1671.901	1671.897	5996.1	1	7.547	75	10
	K.LAPLNE	2.949	0.3641	100	1543.802	1543.802	3179.6	3	6.128	59.1	1
	R.LYLHPD	3.8337	0.3501	100	1506.854	1506.855	3720.9	1	7.478	79.2	16
	R.RGVAPN	3.7925	0.4087	100	1394.75	1394.751	6708.2	1	8.541	76.9	38
	R.GVAPNH	3.5195	0.3767	100	1238.648	1238.649	4817.3	1	7.958	79.2	6
	R.SILQQL	5.4098	0.3961	100	1982.055	1980.052	9368.8	1	7.278	65.6	10
PF14_0077		255	39.30%	453	51481	5.6 U	Annotation|Plasmodium_falciparum_TIGR|(protein codin				
	T.FILDTG	3.272	0.2975	99.9	1746.95	1746.943	8873.9	1	5.636	53.3	2
	K.DLVTVG	3.7455	0.2702	99.9	1418.791	1418.79	11006.7	1	5.874	79.2	6
	K.NQNKIE	4.3862	0.1608	99.8	2292.193	2291.182	7418.3	1	6.807	61.1	2
	K.IENALF	3.9229	0.3049	99.9	1814.967	1813.96	4961.6	1	6.051	51.8	1
	K.IENALF	4.2908	0.4002	100	1814.969	1813.96	7625.8	1	7.154	75	13
	K.HTGFLT	4.4423	0.4723	100	1443.781	1443.778	5497.2	1	8.338	83.3	24
	K.ANCIVD	5.4533	0.5113	100	2224.101	2223.097	4168	1	8.044	62.5	13
	K.MLQNLD	3.1951	0.2445	99.9	1080.623	1080.62	5192.3	2	5.346	87.5	1
	K.VPFLPF	4.8689	0.4114	100	1798.908	1798.92	4062.1	1	8.511	82.1	74
	K.VPFLPF	3.9737	0.3044	100	1798.924	1798.92	6542.2	4	5.452	46.4	3
	K.VPFLPF	4.1505	0.3513	100	1799.928	1798.92	5250.2	1	7.164	64.3	7
	K.LPTFEF	3.2941	0.4766	100	1369.665	1369.664	8231	1	7.47	86.4	9
	K.LPTFEF	2.9757	0.3864	99.8	1369.67	1369.664	3383.2	1	7.395	68.2	1
	K.YTLEPE	5.7407	0.4154	100	4885.443	4883.418	7563.7	1	7.659	20.7	7
	N.IIGLDF	4.2537	0.4662	100	2170.215	2169.208	2940	1	7.07	55.6	23
	K.YFTVFD	5.3624	0.4965	100	2059.033	2058.03	9410.3	1	9.935	76.5	69
PF14_0510		195	39.30%	275	30492	4.4 U	Annotation|Plasmodium_falciparum_TIGR|(protein codin				

	-.MPLYPI	4.2572	0.4246	100	1465.838	1465.835	4315.4	1	7.635	81.8	10
	M.PLYPIV	3.2254	0.3482	99.8	1334.796	1334.795	7736.8	1	6.836	70	1
	M.PLYPIV	4.1161	0.4058	100	1334.798	1334.795	3893.6	1	7.796	90	46
	K.ECLYLT	4.3653	0.3054	100	1981.007	1980	7827.4	1	7.168	66.7	24
	K.DKNEYE	6.6921	0.3139	100	2558.29	2557.283	5721.1	1	6.64	42.5	25
	K.DKNEYE	5.1919	0.3563	100	2559.297	2557.283	9046.2	1	6.69	55	26
	N.FLKPIN	3.8199	0.2487	100	1310.745	1310.743	3383.5	1	5.056	75	1
	G.NMGGM	3.458	0.3765	100	1271.542	1271.54	5272.7	1	7.867	77.3	3
	K.LGNMG	5.2947	0.5107	100	2159.966	2156.957	5871.7	1	8.878	65	33
	G.NMGGD	4.9023	0.5125	100	1987.857	1986.851	5934.8	1	8.114	75	3
	K.TLDSDK	3.9381	0.3961	100	2854.445	2853.437	8293.4	1	6.925	38	11
	K.TLDSDK	4.7213	0.3296	100	2861.459	2860.454	4879.3	1	5.941	32	3
	K.LKDEEN	5.0764	0.4006	100	2202.147	2201.141	6634.4	1	7.688	57.9	9
PFF0880c		21	39.20%	199	23334	4.8	U	Annotation|Plasmodium_falciparum_Sanger|(protein coc			
	K.TNEVDD	5.6687	0.31	100	4054.812	4052.798	10903.7	1	7.278	27.3	5
	K.TNEVDD	4.9621	0.2994	100	4054.814	4052.798	7741	1	4.996	20.8	2
	K.ILQNNL	5.8576	0.5509	100	2167.124	2167.118	11580	1	8.953	63.9	1
	K.ILQNNL	5.0193	0.4271	100	2275.17	2274.162	7786	1	7.822	63.2	7
	Q.NNLDDI	3.9972	0.3649	100	1934.977	1933.969	10332.6	1	6.523	50	4
	K.IITLEST	3.7738	0.4646	100	2771.524	2771.524	7001.9	3	6.793	24	1
	K.IITLEST	4.0087	0.4439	100	2772.552	2771.524	8694.5	1	7.767	41.7	1
PF10_0084		429	38.70%	445	49751	4.8	U	Annotation|Plasmodium_falciparum_TIGR|(protein codin			
	K.FWEVIS	5.0699	0.4322	100	3128.417	3125.38	4218.4	1	6.652	29.8	2
	R.VDVFYN	3.6878	0.4479	100	1327.631	1327.628	6550.2	1	7.569	90.9	3
	R.AGPFGC	3.0426	0.3342	99.9	1997.997	1997.987	8696.8	1	5.848	47.1	3
	R.AGPFG	3.2399	0.3025	99.9	2798.371	2796.365	5182.4	1	4.736	34	2
	F.RPDNF	3.7407	0.3625	99.9	1978.938	1978.952	4735.9	2	5.578	44.1	3
	F.RPDNF	5.084	0.2696	100	1979.959	1978.952	7232.5	1	8.711	73.5	10
	A.KGHYTE	3.9393	0.4026	100	2085.109	2085.098	8778.7	1	6.975	58.3	2
	K.GHYTEC	4.8116	0.2763	100	1957.005	1957.003	7175	1	5.945	42.6	6
	K.GHYTEC	4.9926	0.4932	100	1964.024	1964.02	7361.7	1	9.276	64.7	22
	K.GHYTEC	7.6201	0.5686	100	1967.04	1964.02	10848.7	1	10.337	85.3	88
	K.GHYTEC	4.3717	0.2216	99.9	2085.115	2085.098	7991.9	1	6.672	63.9	2
	K.GHYTEC	4.5564	0.458	100	2092.117	2092.115	6164.1	1	7.515	37.5	4
	R.IMETFS	3.6919	0.4574	100	1389.722	1389.72	9466.1	1	8.111	86.4	1
	K.LTTPTY	5.1981	0.5017	100	2664.303	2664.313	9191.5	1	9.774	50	156

	T.PTYGDL	5.6922	0.4891	100	2352.14	2349.133		7475	1	8.623	69	20
	K.LAVNLIF	4.0761	0.4584	100	1146.711	1146.711		3801.2	1	7.901	94.4	29
	K.NSSYFV	5.1645	0.5038	100	1728.857	1728.846		6181.4	1	9.096	69.2	72
	K.NSSYFV	3.1432	0.3656	99.8	1728.857	1728.846		4972.1	1	6.238	53.8	1
	K.MAVTFV	5.1657	0.4805	100	1880.936	1880.936		4383.2	1	8.84	62.5	1
	R.VSDQFT	2.7534	0.2921	99.8	1201.569	1201.568		8344.4	1	5.483	83.3	2
PF13_0178		40	38.50%	247	27250	4.8	U	Annotation|Plasmodium_falciparum_Sanger|(protein coc				
	R.LTNSYA	7.0542	0.5167	100	4174.152	4172.138		5525.6	1	8.15	24.3	13
	K.GLLVSS	4.7915	0.4686	100	1962.067	1961.06		7660.3	1	8.676	65.6	7
	R.LSALGN	5.5677	0.407	100	4505.347	4504.337		10192.6	1	6.907	18.4	7
	R.LSALGN	7.3262	0.5971	100	4506.346	4504.337		10710.8	1	11.009	25.7	13
PFE1195w		781	38.20%	1123	127353	4.9	U	Annotation|Plasmodium_falciparum_Sanger|(protein coc				
	K.IVEVIE	5.4134	0.5747	100	1775.984	1775.98		6649.5	1	9.365	80	31
	R.NECENT	3.9018	0.2895	100	1575.715	1575.711		7897.9	1	6.119	72.7	3
	K.KNDLNN	4.3234	0.3556	100	1471.849	1471.848		9054.8	1	7.033	75	16
	K.NDLNNT	3.7353	0.2248	99.9	1343.756	1343.753		8156.9	1	5.68	77.3	3
	K.NENSIG	4.168	0.364	100	2569.239	2568.233		8443.7	1	6.429	42.9	20
	R.GECINL	5.2531	0.4703	100	2237.081	2237.079		6998.7	1	8.022	65.8	29
	K.SVHGCI	4.5848	0.2721	99.9	1967.07	1967.082		9407	5	5.791	42.6	4
	K.SVHGCI	4.5803	0.5025	100	1984.113	1981.117		5057.7	1	8.931	67.6	14
	K.AFSELE	5.6262	0.4132	100	2054.077	2051.068		9914.3	1	8.556	68.8	44
	R.YAACQA	4.2699	0.3798	100	2305.167	2304.163		7712.9	1	6.854	38.2	4
	R.YAACQA	4.9633	0.4555	100	2307.171	2304.163		9094.7	1	7.614	55.3	33
	R.VQSHAT	5.7343	0.5279	100	1993.97	1992.967		6148	1	9.902	58.8	5
	R.VQSHAT	5.9781	0.4831	100	1993.972	1992.967		8888.7	1	9.219	76.5	51
	K.MALLPY	4.9142	0.4795	100	1971.202	1971.198		3636.4	1	8.679	75	5
	K.MALLPY	6.2136	0.372	100	1972.203	1971.198		8163.2	1	6.687	48.4	12
	L.PYADIII	5.4279	0.3328	100	1542.952	1542.952		8701.2	1	8.001	83.3	5
	K.LNSSNY	2.9508	0.3652	100	1178.655	1178.653		7181.6	1	6.753	88.9	3
	R.EQAVTA	5.8339	0.4795	100	2118.162	2116.154		8756.8	1	8.279	76.3	45
	R.EQAVTA	6.0819	0.5124	100	2138.212	2137.206		8586.8	1	8.151	40.8	19
	K.MDPDDT	3.3156	0.3315	99.9	1979.941	1979.939		4367.7	1	5.773	46.9	2
	L.GNDFYF	4.6599	0.4375	100	2637.482	2636.478		6153	1	7.73	43.5	2
	D.FYPYLS	3.6218	0.438	100	2351.39	2350.387		5703.2	1	6.616	50	2
	F.YPYLSS	4.8815	0.4575	100	2204.318	2203.318		4941.2	1	7.622	57.9	7
	Y.PYLSSI	5.7074	0.5882	100	2040.254	2040.255		4180	1	10.088	75	15

	I.VPTILSV	3.4616	0.4974	100	1372.888	1372.889		5519.9	1	8.986	75	8
	V.PTILSVL	4.8517	0.494	100	1275.826	1273.821		8657.4	1	9.293	90.9	17
	K.ALTAVS	4.3588	0.3731	100	1366.768	1366.766		7486.6	1	7.129	75	21
	K.SMLLAI	4.796	0.3592	100	1331.764	1331.761		8916.2	1	7.822	83.3	9
	K.AGSNVL	3.3068	0.2607	99.9	1242.669	1242.669		4281.7	3	4.819	68.2	1
	K.NNDDVI	3.2993	0.3805	100	2992.386	2992.385		6099.8	1	6.568	32.6	3
	R.TNLLDIL	4.8454	0.3707	100	1325.861	1325.86		8216.4	1	8.591	86.4	61
	K.SVNLWI	4.3404	0.4708	100	2617.297	2616.292		8302.6	1	8.495	45.2	37
	K.SVNLWI	4.1898	0.3013	99.9	2624.318	2623.309		5042.3	1	6.323	33.3	5
	K.YANIAVI	3.1768	0.4008	100	1305.721	1303.738		6884.9	1	6.882	90	1
	K.EYISAI	5.1854	0.5816	100	2453.309	2452.306		8193.1	1	9.733	54.5	54
	Y.ISAIDN	3.7821	0.3414	100	2160.199	2160.2		6297.1	1	5.973	42.5	1
	I.SAIDNA	5.4272	0.54	100	2043.106	2040.099		7376	1	9.426	60.5	20
	S.AIDNAI	5.1983	0.4586	100	1953.067	1953.067		7498	1	7.988	69.4	16
	A.IDNAIA	4.7392	0.4813	100	1882.03	1882.029		8135.7	1	8.777	70.6	11
	I.DNAIAA	5.1285	0.4348	100	1761.929	1761.928		7134.6	1	10.198	75	7
	K.VWLNHI	5.1899	0.4078	100	1898.988	1898.984		4911.8	1	7.563	70	12
	K.VWLNHI	4.2474	0.3918	100	1899.991	1898.984		6661	1	6.883	46.7	7
	K.NLIDLV	3.1436	0.2837	99.8	1623.897	1622.891		7609.7	1	5.616	61.5	3
	K.NLIDLV	5.5823	0.3629	100	1815.028	1815.024		6158	1	8.655	80	93
	L.IDLVSQ	3.6224	0.2608	99.8	1589.912	1587.897		4476.1	1	5.797	61.5	3
	K.ISTLINS	2.8472	0.2892	99.9	1117.667	1117.666		5116	1	5.261	83.3	1
	K.SYLNNL	3.7224	0.2833	99.9	1535.788	1534.787		6032	1	5.876	73.1	16
PF10_0203		128	38.10%	181	20912	6.2	U	Annotation|Plasmodium_falciparum_TIGR|(protein codin				
	K.LGEVVT	3.1126	0.3408	99.9	2336.279	2335.274		4505	1	5.914	42.5	1
	R.MINEEE	4.4518	0.2828	99.9	2090.111	2090.121		7033.7	1	5.129	50	4
	R.MINEEE	5.8215	0.3776	100	2091.128	2090.121		9346.7	1	7.391	73.5	41
	R.NWFIQS	2.8476	0.2805	99.8	1390.656	1390.665		6772.8	1	5.393	70	4
	R.GDGLYE	5.356	0.4253	100	2152.025	2151.015		8461.8	1	7.361	63.9	78
PFI1270w		102	37.80%	217	24698	5.6	U	Annotation|Plasmodium_falciparum_Sanger|(protein coc				
	N.NNVNNI	4.4405	0.4408	100	1508.787	1508.782		6451	1	8.664	76.9	6
	N.NVNND(	3.911	0.4549	100	1394.742	1394.739		7071.7	1	8.221	79.2	3
	K.NLQGTF	3.9743	0.4086	100	1778.874	1778.867		4357.8	1	8.509	56.7	8
	K.NLQGTF	5.4281	0.563	100	1778.878	1778.867		10412.9	1	10.314	76.7	39
	K.NLQGTF	4.2885	0.4469	100	2700.367	2700.364		10385.4	1	7.826	39.1	22
	K.NLQGTF	4.5406	0.2753	99.9	2700.382	2700.364		7239.5	1	5.493	29.3	2

	R.DGNLGY	4.8212	0.4958	100	1705.835	1705.845	8980.9	1	9.15	78.6	14
	K.LINSLY	4.6944	0.3621	100	3418.705	3418.692	6785.9	1	6.17	33.9	2
	K.LINSLY	3.7757	0.4403	100	3420.705	3418.692	6659.6	1	7.297	37.5	6
PF14_0257		132	37.40%	281	33006	4.4	U	Annotation|Plasmodium_falciparum_TIGR|(protein codin			
	K.DATNEH	5.9804	0.4881	100	2139.041	2139.032	11098.6	1	9.679	69.4	33
	K.DATNEH	5.0245	0.4343	100	2267.133	2267.127	7497	1	8.03	63.2	7
	C.LNQTGI	5.6762	0.4264	100	3818.047	3817.06	7061.7	1	6.799	26.5	16
	N.QTGIQT	4.6391	0.3834	100	3610.958	3610.984	5098.4	1	6.232	21.9	7
	I.PSIATL	3.7752	0.3711	100	2492.379	2492.361	6053.8	1	6.816	40.9	2
	K.TLEIGG	2.9291	0.2605	99.8	1122.598	1122.602	7264.6	1	4.42	83.3	1
	G.VQFNPF	4.3479	0.4084	100	1581.749	1581.745	5854	1	7.409	83.3	9
	R.KELFEF	4.3591	0.259	100	1366.773	1363.763	8969.5	2	5.558	85	19
	K.ELFEFLI	3.3028	0.3076	100	1238.678	1235.668	5261.6	1	5.192	88.9	14
	K.RKQDAL	4.788	0.5187	100	1776.856	1776.855	7565.9	1	8.356	82.1	6
	K.RKQDAL	4.32	0.511	100	1904.952	1904.95	7936.9	1	8.402	70	3
	R.KQDALD	4.6212	0.3971	100	1621.756	1620.754	7827.5	1	8.555	92.3	9
	R.KQDALD	4.3576	0.2914	100	1751.859	1748.849	7724.4	1	6.737	67.9	3
	K.QDALDN	3.223	0.3022	99.9	1492.66	1492.659	5294.2	1	6.481	75	3
PFE0040c		1035	37.00%	1434	168287	4.8	U	Annotation|Plasmodium_falciparum_Sanger|(protein coc			
	R.TFDEDH	3.7751	0.3629	100	1404.68	1402.672	8592.7	1	7.773	80	7
	R.NAPYID	4.8992	0.503	100	1898.957	1896.946	4917.6	1	8.539	71.9	5
	R.NAPYID	4.5435	0.395	100	2053.05	2053.047	4683.5	1	8.776	73.5	14
	D.PLWGSY	2.7405	0.3521	99.9	1259.707	1257.696	3525.4	2	5.712	75	3
	R.RKQQEI	3.9804	0.3498	100	2016.022	2015.016	5982.8	1	5.96	46.7	2
	R.RKQQEI	5.2726	0.4298	100	2030.054	2029.05	6819.9	1	7.749	76.7	3
	K.QQEEEN	4.0694	0.4059	100	1744.859	1744.854	5825.1	1	7.608	57.7	9
	M.KELEEM	3.9788	0.4083	100	1580.704	1580.7	5937.8	1	7.145	79.2	3
	K.ELEEMD	4.1068	0.3002	100	1455.617	1452.605	7406.5	1	6.842	86.4	3
	R.KHVNVM	2.9181	0.397	99.8	1128.587	1128.584	5084.5	1	6.691	77.8	1
	R.KHVNVM	3.3214	0.2575	99.9	1131.602	1128.584	3497.3	1	5.181	83.3	22
	K.HVNVMC	3.0234	0.3563	100	1000.488	1000.489	4679.9	1	6.523	87.5	6
	K.ETDESK	4.9602	0.4012	100	2029.882	2028.873	6175.7	1	8.685	61.8	9
	K.VSGPYE	4.169	0.343	100	2416.088	2415.08	4228.7	1	6.134	50	3
	K.KLAEKE	3.7469	0.3442	100	1403.707	1403.702	8392.5	1	6.352	77.3	3
	K.KLTEQE	3.2263	0.3071	99.9	1276.642	1276.638	6324.6	1	6.015	80	3
	K.SFFQML	4.4594	0.3745	100	1767.792	1765.786	6950	1	6.302	80.8	12

K.VLGEGD	3.7238	0.1929	99.8	1446.753	1445.749	8332.8	4	5.133	70.8	4
K.VLGEGD	4.6996	0.3337	100	2303.128	2303.137	5907.1	1	6.134	55.3	3
K.VKGPEII	4.2213	0.3058	99.9	1981.163	1981.159	3276.5	1	6.523	45.3	6
K.VKGPEII	4.5138	0.2929	100	2009.229	2009.227	4430.5	23	5.414	36.5	5
K.VKGPEII	5.2296	0.3763	100	2009.232	2009.227	8283.6	1	7.918	62.5	7
K.GPEIIIE	5.3158	0.2846	100	1654.977	1653.969	7970.8	1	6.531	84.6	13
K.GPEIIIE	5.4806	0.2508	100	1784.073	1782.064	8545.6	1	6.462	82.1	24
K.QVEDGI	3.0484	0.3448	99.9	1797.825	1797.822	5753.9	1	6.737	56.7	3
K.VKGPEI	3.8448	0.3996	100	1969.125	1969.122	5016.1	8	6.589	33.3	4
K.VKGPEI	4.5829	0.3547	100	1970.132	1969.122	3849.1	1	6.195	43.8	6
K.VKGPEI	6.2904	0.411	100	1990.178	1990.174	7516.6	1	7.888	68.8	38
K.GPEIITE	4.9384	0.3251	100	1634.917	1634.915	7726.2	1	6.85	80.8	22
K.GPEIITE	5.1821	0.2988	100	1763.013	1763.01	7546.2	1	6.323	75	86
K.GPEIITE	3.9266	0.3344	100	1763.016	1763.01	5506.5	1	5.738	48.2	4
K.QVEEGI	3.6919	0.3211	100	1805.825	1804.82	3908.7	1	6.913	50	3
K.QVEEGI	3.5861	0.2958	99.9	2039.007	2039.001	4062	1	5.503	58.8	3
K.KQVEEC	6.1001	0.4137	100	1985.004	1983.995	6020.8	1	7.636	65.6	4
K.QVEEGI	3.634	0.4258	100	1612.782	1612.778	5526.2	1	8.065	61.5	10
K.QVEEGI	3.4917	0.4209	100	1858.911	1855.9	3764.5	1	6.723	53.3	12
K.LIGQEII	4.1367	0.4561	100	1392.829	1392.825	5317.4	1	7.195	90.9	9
K.LIGQEII	4.8261	0.524	100	1827.09	1827.085	6932.2	1	8.704	66.7	6
K.EGIKEN	3.4776	0.2682	99.9	1526.744	1526.741	3987.3	1	5.303	66.7	2
K.VIGQEII	4.4435	0.3773	100	1379.816	1378.809	5198.9	1	7.262	90.9	36
K.VIGQEII	4.821	0.481	100	1813.074	1813.07	8029.5	1	8.262	66.7	5
K.VIGQEII	4.5451	0.327	100	1506.907	1506.904	6853.9	1	6.167	83.3	27
K.DKVIGQ	5.4633	0.4744	100	2255.28	2255.276	4792.2	1	8.547	45.8	6
K.VIGQEII	5.9787	0.1649	100	2015.165	2012.154	7767.6	1	7.561	75	43
K.DTANKD	5.0426	0.2602	99.9	3040.553	3039.545	8404.7	1	6.207	29.2	3
K.DKEIEQ	4.5603	0.1122	99.8	1760.896	1760.892	7011.3	1	4.631	73.1	12
K.EIEQEK	3.7567	0.1946	99.8	1781.927	1781.925	4497.7	21	4.884	57.7	1
K.EKEEVK	4.2796	0.1387	99.9	1503.794	1503.791	6143.3	1	5.387	86.4	108
K.EKEEVK	5.3081	0.1124	99.9	1763.94	1760.928	5887.2	1	5.631	84.6	144
K.EKEEVK	6.0904	0.0619	99.9	2247.185	2246.177	6531.4	2	4.497	58.8	30
K.EKEEVK	5.3582	0.0775	99.8	2989.574	2988.563	5867.7	1	5.558	43.5	24
K.EKEEVK	7.7062	0.0728	99.9	3246.709	3245.7	10235.7	1	5.552	35	24
K.EEVKEK	4.8372	0.0721	99.8	1506.804	1503.791	4864.5	2	4.291	86.4	36

	K.EEVKEK	5.8522	0.0855	99.9	1990.048	1989.039	7884.4		2	4.917	70	30
	K.VIGQEII	4.0971	0.1862	99.9	1546.993	1546.991	7803.9		1	5.671	70.8	25
	K.DKVIEQ	4.803	0.2346	99.9	2481.33	2480.321	8354.5		2	5.074	36.8	1
	K.VIEQEK	5.2166	0.1881	100	2237.203	2237.199	6203.6		1	5.805	58.8	1
	K.VIEQEK	5.2607	0.3384	100	2239.207	2237.199	6787.5		1	6.931	45.6	1
	K.VIEQEK	6.9869	0.2653	100	2495.343	2494.336	12991.5		1	6.143	39.5	3
	K.VIEQEK	5.1381	0.2435	99.9	2980.591	2979.585	8988.2		5	6.076	29.3	1
	K.VIEQEK	5.3281	0.2178	99.9	3237.734	3236.723	10152.6		2	4.829	27	1
	K.EVIIEEV	3.8253	0.2642	100	1444.801	1443.795	10481.7		1	5.728	77.3	6
	K.EVIIEE	4.9648	0.3171	100	1585.926	1585.924	10203		1	7.133	87.5	8
	R.KDVIEQ	6.4561	0.4585	100	1901.986	1901.989	5311.4		1	8.696	83.3	6
	R.KDVIEQ	5.0712	0.4166	100	2246.167	2246.159	6073.2		1	6.429	38.9	3
	R.KDVIEQ	6.3213	0.3478	100	2247.161	2246.159	5610.3		1	7.418	69.4	2
	R.VKKPID	3.2031	0.3065	100	1237.708	1237.705	4853		1	5.171	77.8	1
	K.KYNNGE	4.5664	0.2736	100	1807.86	1806.855	6538.3		1	5.641	80.8	3
	R.EFHNIL	4.1813	0.3586	100	2198.111	2198.099	5606.3		2	6.127	50	9
	R.ILYNLF	3.4868	0.309	99.9	2023	2023.011	10173.3		1	5.917	60	2
	K.FSEMNV	5.3469	0.5029	100	2015.981	2014.977	7681.5		1	7.951	73.3	23
	R.LNTVIL	4.5578	0.4777	100	2655.458	2654.453	7099.9		1	8.963	52.3	28
PF07_0033		756	37.00%	873	99964	5.7	U	Annotation|Plasmodium_falciparum_Sanger|(protein coc				
	R.VLSALL	3.6649	0.3771	100	1200.787	1200.779	8428.7		1	8.406	85	3
	K.IINANVL	2.9888	0.3141	100	926.5984	926.5974	2921.5		1	5.179	92.9	1
	K.YITNIF	4.3787	0.2993	100	1404.763	1402.756	6296.2		1	7.168	90	30
	K.NNTPEL	3.463	0.4136	100	1446.65	1446.647	5301.5		1	7.601	62.5	3
	K.FLVTST	3.7017	0.3579	100	1836.911	1836.909	6787.9		1	6.228	65.6	2
	R.ETFEEL	4.1525	0.3768	100	1764.821	1764.815	7859.9		1	7.471	76.9	21
	R.ETFEEL	3.3805	0.3315	99.8	1764.824	1764.815	3863.3		1	5.846	53.8	1
	K.HLLDTA	3.4124	0.4023	100	1270.683	1270.683	4965.1		1	6.251	90	12
	K.VNIQDI	5.7111	0.4766	100	1859.033	1859.029	7421.5		1	9.097	78.1	38
	R.VPFIQN	4.89	0.4044	100	1808.971	1806.966	9727.3		1	7.587	76.9	8
	V.PFIQNF	4.9224	0.4053	100	1707.897	1707.897	10038.5		1	8.569	87.5	9
	K.VTHPIN	3.6795	0.3172	99.9	1973.985	1973.983	4478.7		1	6.212	46.9	2
	K.VTHPIN	5.7162	0.5004	100	1988.021	1988.018	7959.7		1	10.089	75	109
	K.VTHPIN	4.587	0.4364	100	1988.027	1988.018	7730.8		1	7.818	59.4	15
	K.KKADDK	3.7512	0.2764	99.9	1491.769	1491.766	5893.8		1	7.073	66.7	3
	K.KADDKT	3.2712	0.287	99.9	1363.681	1363.671	2984		1	5.482	72.7	6

	K.NNETNS	3.4568	0.3162	100	1488.733	1488.729	5390	5	6.04	62.5	1
	K.DIFNFS	5.0594	0.5133	100	2672.259	2672.256	8538.8	1	8.773	47.6	7
	K.DIFNFS	3.8623	0.3062	99.8	2672.263	2672.256	7067.6	1	6.208	32.1	1
	R.LKHLNE	4.0186	0.3185	100	1858.014	1858.007	5522.4	1	5.701	50	2
	R.LKHLNE	5.4588	0.4612	100	1872.048	1872.042	6531.1	1	9.042	82.1	11
	K.HLNELE	3.7377	0.325	100	1632.871	1630.863	5178.8	16	5.403	45.8	1
	K.HLNELE	5.5463	0.424	100	1633.871	1630.863	6206.1	1	7.499	91.7	64
	R.ILLSLD	4.1894	0.2598	99.8	2514.235	2513.228	8705	2	4.635	32.9	1
	R.ILLSLD	6.1318	0.4698	100	2516.235	2513.228	9303.1	1	8.426	57.9	110
	R.ILLSLD	4.4307	0.2749	100	3134.536	3132.524	7842	1	5.97	37.5	46
	R.ILLSLD	6.4265	0.4683	100	3154.584	3153.575	5743.5	1	7.849	33.3	34
	K.KKEEIRI	3.9385	0.2968	100	1286.808	1285.803	6683.3	1	5.051	77.8	4
	K.QQNLGI	4.8713	0.3141	100	3731.949	3731.945	8680.4	2	5.22	17.7	2
	K.QQNLGI	5.3803	0.4532	100	3736.957	3731.945	9415.9	1	7.321	25	7
	N.LGNIIN	7.4503	0.4981	100	3362.791	3361.785	7533.8	1	9.319	36.6	4
	N.LGNIIN	5.6485	0.3321	100	3362.792	3361.785	7705.8	1	6.426	26.2	1
	N.IINHLN	7.51	0.4593	100	3077.64	3077.636	7184	1	8.081	42	15
	K.FLNNIN	5.4764	0.4358	100	1583.818	1583.814	6583.7	1	6.918	87.5	15
	K.NKPLYE	4.3292	0.4294	100	3264.659	3263.648	9830.2	1	7.825	42.6	77
	K.NKPLYE	5.1699	0.2824	100	3265.665	3263.648	9651.2	1	6.004	32.1	3
	K.NKPLYE	7.8359	0.4543	100	3276.679	3270.665	8362.3	1	8.328	36.1	69
	K.EKNEET	5.9342	0.4563	100	2221.983	2218.97	6675.7	1	7.759	58.3	15
	K.NEETNL	5.4853	0.4502	100	1961.837	1961.832	7784.8	1	8.679	75	3
PF08_0071		101	36.90%	198	22734	6.8	U	Annotation|Plasmodium_falciparum_Sanger|(protein coc			
	K.YALNAL	5.3238	0.3737	100	2461.23	2461.215	7800.2	1	8.709	55	68
	K.YALNAL	5.2148	0.2763	100	2468.249	2468.232	8001.7	3	6.508	32.5	6
	K.ESSGA	5.6621	0.4342	100	4043.822	4043.802	3253.6	1	7.197	23.6	3
	K.AWWNL	5.7109	0.4121	100	2018.978	2018.988	8399.7	1	8.604	76.7	24
PFE0585c		260	36.40%	604	69112	7.4	U	Annotation|Plasmodium_falciparum_Sanger|(protein coc			
	R.KEDENI	4.7332	0.4342	100	2501.164	2501.147	7890.2	1	7.67	60.5	19
	K.EDENFI	3.6112	0.3797	100	2373.055	2373.052	5195.3	1	6.569	55.6	4
	R.LGYNEK	4.5284	0.5554	100	2046.031	2046.041	7403.4	1	9.419	68.8	7
	R.LGYNEK	4.2637	0.385	100	2046.049	2046.041	7200.9	1	6.583	43.8	2
	G.YNEKDI	4.7456	0.3762	100	1875.927	1875.935	7343.8	1	7.096	46.4	1
	G.YNEKDI	4.6134	0.4498	100	1875.938	1875.935	7655.3	1	8.168	67.9	4
	G.YNEKDI	4.0432	0.4619	100	1875.938	1875.935	5851.9	1	7.299	53.6	2

	K.DKEHAY	4.0173	0.379	100	1341.693	1341.692	8243.3		1	7.288	85	10
	K.LIDIYN	4.9562	0.3855	100	2708.36	2708.339	8228.8		1	7.302	54.5	25
	K.IKDDLD	3.3365	0.3454	100	1325.745	1325.743	7566.5		1	6.574	85	2
	K.NKLEIL	3.2415	0.2026	99.8	1248.735	1248.739	7017.2		1	4.827	83.3	1
	K.KQNNLN	5.3803	0.557	100	2107.114	2107.126	6927.6		1	9.584	73.5	25
	K.KQNNLN	4.2178	0.3523	100	2107.129	2107.126	9508.3		1	6.309	38.2	5
	K.QNNLNE	4.0043	0.4413	100	1972.003	1972.014	7856.8		1	7.328	53.1	6
	K.NIPHIP	4.0486	0.2596	99.8	1914.027	1914.024	7339.8		1	6.311	56.2	3
	K.NIPHIP	4.5462	0.3499	100	1935.063	1935.075	5096.8		2	7.547	42.2	1
	K.NIPHIP	4.594	0.4068	100	1938.074	1935.075	4356.7		1	6.712	65.6	19
	K.SIVSYN	4.0527	0.3555	100	1518.737	1517.735	9714.3		1	7.464	76.9	4
	R.ANENLY	5.2432	0.3724	100	2080.031	2079.025	8863.3		1	7.161	67.6	18
	K.VSADIE	5.3616	0.5132	100	1972.872	1972.87	7112.7		1	8.802	71.9	8
	K.VSADIE	3.8653	0.4879	100	1979.889	1979.887	6972.8		1	8.12	59.4	2
	K.AIDEYI	3.8994	0.4505	100	1629.773	1629.783	10883		1	7.429	65.4	5
	R.VFFKPN	2.842	0.2946	99.9	1136.612	1136.611	4761		1	5.052	81.2	6
	K.TNEDEH	5.7551	0.4004	100	2232.092	2231.065	5599.4		1	8.451	61.8	14
	K.NLDSVL	4.6436	0.4786	100	1642.849	1642.851	10098.9		1	9.386	84.6	67
PF11_0314		125	36.40%	439	49541	5.2	U	Annotation|Plasmodium_falciparum_TIGR|(protein codin				
	K.MLPYLV	5.6765	0.2848	100	4312.03	4310.019	7739.4		1	6.059	25.7	8
	L.VANVVE	5.7495	0.4718	100	3687.692	3685.677	8987		1	7.86	33.3	9
	T.IFLPVP	3.8243	0.4822	100	2506.464	2505.452	7630.7		1	8.07	45.7	7
	K.AMEVIE	5.954	0.4672	100	2210.079	2210.073	4609		1	8.515	71.1	3
	K.QIEDLV	5.0857	0.204	99.9	1720.944	1719.936	5144.6		4	5.01	50	9
	K.QIEDLV	4.3179	0.4375	100	1734.978	1733.97	5774.6		1	7.524	75	7
	K.QIEDLV	4.8969	0.4778	100	1862.073	1862.065	8545.4		1	8.64	73.3	25
	K.EKAPAII	4.4779	0.4479	100	1972.131	1972.138	8955.8		1	8.583	58.8	22
	K.APAIIFI	4.3148	0.5097	100	1714.993	1715.001	7510.9		1	8.735	70	11
	K.RFDSEL	2.9528	0.2447	99.8	1181.557	1181.555	6750.3		1	5.002	88.9	1
	R.TMLELL	4.1676	0.4638	100	2140.065	2140.048	7838.7		1	7.792	66.7	15
	K.VIAATN	3.7258	0.3582	100	1836.031	1836.034	3665.7		1	6.892	53.1	8
PFA0520c		29	35.70%	446	50733	5.1	U	Annotation|Plasmodium_falciparum_Sanger|(protein coc				
	K.IDEDEE	4.1701	0.3731	100	2146.922	2146.917	8730.1		1	7.674	59.4	1
	W.KTNSGI	3.6062	0.2672	99.9	1573.788	1572.784	9628.2		1	5.975	75	6
	K.TNSGLL	3.2492	0.272	99.9	1444.691	1444.69	7117.9		1	5.571	68.2	6
	K.EPNFLY	3.24	0.2917	99.9	2565.278	2564.275	8165.2		1	5.74	37.5	2

	K.SNENYE	3.5656	0.5219	100	1725.674	1725.67		8582.1	1	8.593	65.4	3
	K.AVDGNV	4.3433	0.3456	100	1448.764	1448.761		8452.5	1	7.182	79.2	2
	R.LNFSPN	4.9326	0.4724	100	1892.941	1890.931		8548.9	1	7.997	67.6	3
	R.FINVYDI	2.8187	0.2892	99.9	1132.614	1132.612		5331.2	1	5.462	87.5	1
	K.IGEELD	4.9467	0.3837	100	4114.942	4112.938		6086.8	1	5.459	21.5	4
	K.MFIGST	3.4615	0.4494	100	2152.053	2152.05		7970.2	1	7.662	55.9	1
PF07_0088		190	35.40%	195	21863	9.6	U		Annotation|Plasmodium_falciparum_Sanger|(protein coc			
	K.ACVYTP	2.9065	0.3242	99.9	1232.585	1232.585		4999.9	1	6.688	80	12
	R.LVNSM	3.1616	0.3222	100	1175.549	1175.549		7932.6	1	6.531	88.9	15
	R.RQAVDV	3.0555	0.2922	99.9	1140.653	1140.649		4975.4	1	5.719	77.8	4
	R.RVNQAI	5.3357	0.4147	100	1648.914	1648.914		5883.5	1	9.249	76.9	35
	R.VNQAIY	3.6408	0.3778	100	1478.778	1478.779		7521.5	1	7.223	75	4
	K.SISECL	4.0173	0.3771	100	2677.232	2675.218		7456.9	2	6.025	29.3	2
	K.SISECL	5.9315	0.5335	100	2707.316	2703.287		7651.8	1	9.781	47.8	118
PFC0920w		372	35.40%	158	16453	10.6	U		Annotation|Plasmodium_falciparum_Sanger|(protein coc			
	R.VGSTAA	6.3656	0.3581	100	2910.556	2909.551		8476	1	7.068	28	15
	R.VGSTAA	5.0796	0.5234	100	2910.592	2909.551		9647.8	1	8.837	42.9	7
	R.VGSTAA	5.8866	0.415	100	2913.569	2909.551		10171.3	1	7.893	34.8	16
	G.STAAVY	5.9216	0.4715	100	2755.473	2753.461		9665.1	1	8.185	34.6	7
	A.AVYAAA	5.0366	0.3929	100	2495.349	2494.345		8838	1	7.826	35.9	2
	Y.AAAILE	6.252	0.3972	100	2161.165	2161.176		7886	1	7.811	60	94
	Y.AAAILE	6.9808	0.4798	100	2168.205	2168.193		8394.8	1	8.68	47.5	58
	A.AAILEY	5.7419	0.3658	100	2090.117	2090.138		10422.6	1	6.892	43.4	43
	A.AAILEY	6.0444	0.3923	100	2090.144	2090.138		7348.8	1	7.611	71.1	73
	A.AILEYL	5.3338	0.4412	100	2026.111	2026.119		7553.7	1	7.876	69.4	48
	L.AIRGDEI	4.2269	0.3183	100	1486.835	1486.83		6245.8	1	6.654	75	1
	K.ATIAGG	3.7739	0.3733	100	992.5492	992.5529		6643.3	1	6.534	95	4
	K.ATIAGG	3.0499	0.4141	100	1391.844	1391.842		5738.2	1	5.965	65.4	3
	T.IAGGGV	3.655	0.3869	100	1219.757	1219.758		6262.1	1	7.134	68.2	1
PF14_0448		348	35.30%	272	29958	10	U		Annotation|Plasmodium_falciparum_TIGR|(protein codin			
	K.IVSIEEI	4.8524	0.5689	100	1784.084	1782.079		8919.9	1	9.701	71.4	53
	I.VSIEEIY	4.5402	0.3715	100	1661.978	1661.978		7682.6	1	7.01	69.2	11
	K.EYQIID	4.7565	0.3463	100	2446.175	2442.163		6329.3	1	7.64	58.3	47
	K.AFVAIG	4.3699	0.4379	100	1681.853	1678.845		7505.2	1	7.734	68.8	14
	K.IGDPHT	3.1149	0.3032	100	1101.585	1101.584		5453.3	1	5.914	77.8	3
	S.KTYGYL	3.5685	0.3047	100	1484.784	1484.779		8040	1	6.306	77.3	6

	K.TYGYLT	3.3881	0.4315	100	1356.688	1356.684		6824.8		1	7.047	80	14
	K.VTNFDK	4.8803	0.2656	100	2855.337	2853.326		9713.2		1	5.384	31.8	2
	K.VTNFDK	4.6934	0.4571	100	2855.34	2853.326		7660.9		1	7.982	47.7	20
	K.SPYEEV	6.3105	0.4921	100	2148.981	2148.977		10110.9		1	9.976	75	178
PFB0300c		89	35.30%	272	27957	5.6	U		Annotation|Plasmodium_falciparum_TIGR|(protein codin				
	K.TTTTTT	4.6157	0.4231	100	2597.15	2597.145		8167.8		1	7.58	39.6	45
	K.GKGEV(	3.9472	0.3549	100	1398.7	1398.698		6321.3		1	6.562	70.8	13
	K.GKGEV	3.6696	0.4868	100	3101.461	3100.453		5167.7		1	7.574	29.6	3
	K.GKGEV	4.4984	0.3101	100	3103.472	3100.453		7204.4		1	5.89	28.7	6
	K.ETQNNS	4.0556	0.2935	100	1721.785	1720.774		4799.1		1	6.69	67.9	3
	K.SNVPPT	2.6015	0.3464	99.8	1273.612	1272.607		3410.4		1	5.598	77.3	1
	K.SPTAQF	5.0255	0.5961	100	3266.483	3266.494		6073.3		1	10.532	40	12
	K.SPTAQF	5.3709	0.4552	100	3268.499	3266.494		7632		1	7.752	29.2	6
PFE1035c		14	35.30%	153	17838	5.4	U		Annotation|Plasmodium_falciparum_Sanger|(protein coc				
	K.YNPVTT	3.7428	0.2939	99.9	2146.117	2146.123		7334		2	5.654	44.1	7
	K.GLHENN	3.3575	0.4055	100	1625.791	1625.788		3754.4		1	7.268	67.9	3
	K.WIGSHE	3.6974	0.3282	100	2388.182	2388.172		6661.4		1	6.564	45	4
MAL8P1.95		221	34.80%	325	37500	4.3	U		Annotation|Plasmodium_falciparum_Sanger|(protein coc				
	C.KVVKPD	4.3	0.3345	100	1566.901	1566.911		5860.1		2	6.326	42.3	3
	C.KVVKPD	4.8006	0.3538	100	1566.905	1566.911		6106.9		1	7.297	80.8	12
	C.KVVKPD	3.8418	0.3791	100	1566.913	1566.911		5273.4		2	6.742	69.2	1
	K.VVKPDE	3.7015	0.2649	99.9	1438.816	1438.816		6576.3		1	4.832	52.1	3
	K.VVKPDE	3.9907	0.3162	100	1441.826	1438.816		4634.9		1	6.228	70.8	12
	K.DGYSVI	3.6522	0.397	100	1153.608	1153.608		5570.8		1	7.914	83.3	14
	R.ACLNNF	4.0656	0.408	100	1269.575	1268.569		8078.4		1	6.702	80	24
	K.IYVQVE	4.0191	0.3888	100	2347.072	2347.071		3410.7		1	6.811	52.8	6
	K.NVCEDT	5.3369	0.2688	100	2251.067	2251.063		10009.3		2	5.315	40.3	2
	K.NVCEDT	6.1611	0.4239	100	2261.085	2258.08		8377.3		1	7.815	66.7	60
	K.TSGSS	6.2537	0.55	100	4638.934	4636.926		10240.2		1	8.966	23.1	84
PFD0950w		63	34.60%	280	33196	5	U		Annotation|Plasmodium_falciparum_Sanger|(protein coc				
	K.TGEEDE	2.7395	0.4211	99.8	1512.665	1512.661		4635.9		1	6.954	66.7	2
	K.VVANHY	5.6166	0.5424	100	1863.882	1863.881		7768.9		1	10.002	78.6	5
	K.KDDNSD	3.1157	0.2595	99.9	1177.549	1177.545		4689.4		3	5.363	77.8	3
	K.TKEDEE	4.0819	0.2551	100	1381.685	1378.67		5031.5		3	5.167	80	9
	K.TKEDEE	4.5389	0.1865	99.9	1735.887	1734.876		7207.9		2	5.432	73.1	7
	K.TKEDEE	5.3692	0.3683	100	2107.05	2107.041		7497.5		1	6.893	71.9	6

	K.EDEEEK	4.1071	0.2003	99.9	1878.909	1877.898		4614.9	1	4.674	64.3	1	
	K.KDVKED	4.5722	0.1634	99.9	1836.865	1835.851		5364.3	1	4.766	78.6	3	
	K.DVKEDD	4.6277	0.2042	99.9	1707.763	1707.756		6333.2	1	5.902	73.1	6	
	K.DVKEDD	4.6091	0.2303	100	1936.907	1936.899		4906	1	6.129	60	3	
	K.DVKEDD	5.8687	0.4042	100	2971.335	2970.319		7247.7	1	6.879	36.5	3	
	K.EDDEEK	4.284	0.4137	100	2629.146	2628.128		6764.3	1	6.557	45.2	3	
	K.EKEVEQ	4.205	0.2183	99.9	1617.839	1617.834		5277.5	3	5.143	75	3	
	K.EKEVEQ	5.3391	0.1615	100	2005.024	2004.014		4834	1	5.19	70	6	
	K.EVEQKE	4.4321	0.1694	99.9	1747.888	1746.876		4969.8	1	4.25	76.9	3	
PFB0450w		46	34.60%	81	9286	8.3	U		Annotation|Plasmodium_falciparum_TIGR|(protein codin				
	K.IPEFLT	3.591	0.434	100	3235.545	3235.537		6418.1	1	7.39	33.3	18	
	K.IPEFLT	6.0998	0.5005	100	3249.556	3249.571		8247.3	1	8.778	40.7	28	
PFE0080c		486	34.40%	398	46739	8.8	U		Annotation|Plasmodium_falciparum_Sanger|(protein coc				
	K.LYPESN	5.7233	0.5495	100	2335.231	2335.216		6694.3	1	9.391	50	17	
	Y.PESNSL	5.695	0.5923	100	2059.068	2059.069		6188.6	1	9.536	36.1	21	
	Y.PESNSL	4.702	0.5176	100	2059.069	2059.069		6517.8	1	8.536	58.3	41	
	K.TNINTL	5.6122	0.5266	100	1899.869	1899.873		7020.9	1	9.285	83.3	16	
	K.SMLSTD	2.7196	0.3455	99.9	1568.698	1568.694		7218.3	1	6.265	58.3	2	
	K.FKDINPI	5.0969	0.1734	99.8	2293.269	2292.264		11261.8	1	4.948	61.1	4	
	K.FKDINPI	5.3127	0.4608	100	2320.351	2320.333		8351.7	1	8.118	63.9	12	
	K.FKDINPI	5.3847	0.2861	100	2421.379	2420.359		7302.6	1	5.614	55.3	4	
	K.DINPLFI	5.4704	0.424	100	2047.179	2045.17		10146.4	1	8.686	75	53	
	K.DINPLFI	4.9152	0.3567	100	2145.201	2145.196		8702.9	1	7.526	67.6	63	
	N.PLFINDI	5.2952	0.4189	100	1697.002	1695.999		9318.7	1	8.093	76.9	61	
	K.SNPYFI	3.277	0.4275	100	1139.585	1139.585		4508.8	1	7.256	88.9	2	
	R.VHTPYK	4.0179	0.2951	100	1697.836	1696.834		5398.7	1	6.696	73.1	6	
	R.SSSLAL	4.7995	0.4181	100	2308.107	2307.104		8213.7	1	7.984	55	30	
	K.DNKSEY	3.6483	0.3781	100	2574.299	2574.292		5510.4	1	6.531	38.1	11	
	K.SEYYGT	5.807	0.555	100	2217.128	2217.127		4999.2	1	9.915	66.7	143	
PF11_0096		172	34.00%	335	39890	8.8	U		Annotation|Plasmodium_falciparum_TIGR|(protein codin				
	K.TPSLIFE	3.9289	0.4324	100	1813.926	1813.938		9833.2	1	7.649	64.3	8	
	T.PSLIFEY	4.7853	0.3996	100	1733.942	1733.941		7231.7	1	7.473	80.8	10	
	K.ALDYCH	4.1085	0.4299	100	1597.735	1594.733		7379.5	1	9.592	79.2	43	
	R.DVKPHN	4.081	0.3781	100	1717.847	1717.844		4461.9	1	6.515	51.9	7	
	R.DVKPHN	4.3485	0.4413	100	1731.882	1731.879		7486.5	1	8.152	69.2	23	
	R.DVKPHN	3.9243	0.4368	100	1731.887	1731.879		4746.8	1	7.436	53.8	3	

	K.PHNIMI	3.1539	0.3107	100	1389.688	1389.688		5452.9	1	5.759	75	1	
	R.LIDWGL	4.6461	0.4915	100	2308.123	2307.12		5640.6	1	8.217	55.6	69	
	K.KEPFFC	3.5405	0.2789	99.9	1996.923	1996.923		6588.8	1	5.199	56.7	1	
	K.VLGTED	3.1072	0.2674	99.8	1358.734	1358.732		9094.9	1	4.635	68.2	2	
	R.KPWSHI	5.1338	0.4145	100	2953.624	2953.605		8556.7	1	6.878	37.5	3	
	R.KPWSHI	3.145	0.3501	99.9	2954.625	2953.605		5730.3	1	5.703	33.3	1	
	K.PWSHFL	4.6351	0.4555	100	1771.94	1770.937		4843.4	1	7.907	75	1	
MAL8P1.62		79	33.60%	277	32165	5.3	U		Annotation|Plasmodium_falciparum_Sanger|(protein coc				
	K.ETASYÇ	5.1647	0.5332	100	2510.335	2510.325		6557.1	1	9.533	52.4	8	
	K.IYNLTD	5.4822	0.5657	100	2424.302	2424.291		4134.7	1	8.374	60	15	
	K.GAPLNN	3.5996	0.2304	99.9	1190.689	1187.679		2460.9	2	6.025	80	41	
	K.KVDTQÇ	3.5251	0.2536	99.9	1302.669	1302.665		6500	1	5.579	75	3	
	K.NENVNN	6.1216	0.2295	100	2314.079	2313.071		8829.8	1	5.982	73.7	10	
	K.NENVNN	4.1483	0.3615	100	3199.435	3198.429		5924.7	1	6.181	37	2	
PF11_0332		19	33.30%	273	31731	5.2	U		Annotation|Plasmodium_falciparum_TIGR|(protein codin				
	K.STDKEE	3.9498	0.2774	100	1578.806	1577.802		5379	1	5.853	54.2	2	
	K.STDKEE	3.6255	0.2998	100	1584.821	1584.82		5790	1	6.283	62.5	5	
	K.IKENEE	4.4535	0.2516	100	1427.776	1427.775		4843.3	1	5.061	81.8	3	
	K.VKNHIE:	3.2644	0.3256	100	1081.657	1081.656		5466.3	1	6.151	81.2	3	
	K.NICTPN	5.6145	0.3137	100	4314.113	4313.102		9723.1	1	6.002	20.7	3	
	K.NICTPN	5.4062	0.3688	100	4314.114	4313.102		6478.6	1	5.874	22.4	1	
	K.YASQQI	3.5818	0.3402	100	1575.793	1575.788		8259.9	1	6.68	75	1	
	K.YHSEFN	3.2852	0.2924	99.8	1045.525	1044.523		3955.8	1	5.897	78.6	1	
PF08_0110		13	33.30%	201	23163	7.9	U		Annotation|Plasmodium_falciparum_Sanger|(protein coc				
	R.ESFENL	2.9319	0.335	99.9	2458.191	2458.197		7320.1	1	5.464	36.8	1	
	V.ANKIDK	3.3875	0.4366	100	1493.815	1493.808		5360.4	1	7.338	62.5	2	
	N.KIDKPN	4.1627	0.3759	100	1744.939	1744.931		5636.9	1	6.926	60.7	1	
	K.IDKPNH	3.4393	0.2523	99.8	1609.825	1609.819		5070.8	1	5.165	73.1	5	
	K.NDINIT	4.3505	0.3614	100	3537.858	3534.841		8207.1	1	6.131	25.9	4	
MAL8P1.121		44	33.00%	261	31191	4.6	U		Annotation|Plasmodium_falciparum_Sanger|(protein coc				
	K.NENLYL	4.3473	0.255	100	1609.836	1608.831		8543.9	1	5.585	86.4	6	
	K.AVASIL	3.7626	0.2234	99.9	1561.802	1561.798		9118.7	1	4.503	61.5	1	
	K.AVASIL	4.3713	0.3847	100	2079.092	2079.088		10665	1	7.188	47.1	3	
	K.NQINNG	6.592	0.5099	100	3731.854	3730.846		9519.4	1	8.919	33.3	29	
	K.LYDNYS	3.655	0.2315	99.9	1441.661	1441.66		7629	1	6.546	81.8	3	
	K.KIDDEV	3.3737	0.3418	100	1556.716	1556.712		6119.3	1	7.394	70.8	2	

PF11_0280		8	32.90%	70	8050	9.5	U		Annotation|Plasmodium_falciparum_TIGR|(protein codin			
	K.PFLNSL	3.1964	0.3398	100	1088.588	1088.585		6170	1	6.475	88.9	6
	R.LTNAEE	3.2337	0.3452	100	1573.769	1573.765		9423.8	1	7.36	70.8	2
		278	32.70%	643	65494	6.6	U		no description			
	K.SLNNQF	4.136	0.4162	100	1386.7	1383.691		8633.5	1	6.815	81.8	14
	K.SLNNQF	3.6154	0.4209	100	1638.862	1638.86		8730.7	1	7.287	69.2	12
	R.FLEQQN	4.6666	0.3472	100	1475.788	1475.786		11713.9	1	6.673	90.9	17
	K.WELLQC	3.5527	0.345	100	1475.752	1475.749		8896.4	1	6.465	86.4	15
	R.THNLEP	5.5239	0.3575	100	1994.983	1993.977		7655.3	1	6.638	51.7	9
	R.THNLEP	5.0271	0.445	100	1996.99	1993.977		4005.3	1	7.901	73.3	80
	K.NMQDM	2.8767	0.3856	100	1301.536	1300.53		6795.7	1	7.31	83.3	3
	R.NKYEDE	2.9269	0.2513	99.8	1308.657	1308.655		5918.4	1	4.991	83.3	1
	R.TNAENE	3.6738	0.2863	100	1395.738	1393.733		8794.1	1	5.44	72.7	6
	K.LDNLQQ	6.6588	0.5584	100	4529.214	4527.202		8744.7	1	10.574	28.3	50
	K.LDNLQQ	5.5815	0.4302	100	4529.229	4527.202		8678.4	1	6.849	19.7	12
	K.SKAEAE	4.0421	0.4559	100	1340.673	1340.67		6665	1	7.592	86.4	7
	K.AEAESL	3.6128	0.3369	100	1125.544	1125.543		5954.3	1	6.897	83.3	6
	K.KQISNL	4.8201	0.396	100	1845.95	1844.947		6208.9	1	7.98	83.3	2
	K.QISNLQ	4.5774	0.5251	100	1716.856	1716.852		7073.6	1	9.091	71.4	4
	K.NKLNDL	5.0792	0.2368	100	1602.844	1599.834		9090.8	1	6.152	84.6	27
	R.LLRDYQ	3.6676	0.2702	99.9	1523.789	1523.789		7354.6	1	6.361	77.3	3
	R.DYQELM	3.2003	0.2066	99.8	1141.521	1141.52		9166.6	1	5.634	87.5	4
	R.GGGGG	5.5597	0.3374	100	2383.956	2383.952		7950.6	1	11.364	38.3	6
PF11_0048		59	32.70%	245	28365	7.4	U		Annotation|Plasmodium_falciparum_TIGR|(protein codin			
	R.ALSNFL	4.1091	0.4323	100	2772.372	2771.389		5990.5	1	7.561	43.5	11
	K.TEIPNF	3.2496	0.338	99.9	2726.271	2726.28		4049.4	1	5.828	39.6	2
	R.VSCAQH	5.4442	0.5627	100	2064.052	2064.048		7088.2	1	9.073	67.6	34
	K.VHVYCF	3.9347	0.2921	100	1717.842	1715.836		6747.4	1	7.491	75	12
		238	32.60%	607	69271	6.1	U		BSA			
	K.LVNELT	3.3058	0.2952	100	1163.635	1163.631		5980.3	1	5.753	88.9	9
	K.TCVADE	4.1213	0.5447	100	1463.593	1463.59		7501.2	1	8.742	79.2	12
	K.SLHTLF	3.8615	0.352	100	1419.696	1419.694		8456.1	1	6.681	56.8	3
	K.SLHTLF	4.1494	0.3858	100	1419.697	1419.694		5860.4	1	8.21	72.7	12
	K.SLHTLF	3.6397	0.3696	100	1420.699	1419.694		6108.3	1	6.941	81.8	9
	R.NECFLS	4.4133	0.3616	100	1903.88	1901.87		8251.5	1	6.427	66.7	6
	K.LKECCD	4.2154	0.2082	99.9	1532.781	1532.782		9880.9	1	6.002	77.3	11

	K.SHCIAE	5.1131	0.3872	100	3513.69	3511.673	7610.4	1	6.354	34.2	2
	K.DAIPEN	3.8785	0.2917	100	2459.19	2458.181	4848.9	1	6.479	40.5	2
	K.DAFLGS	3.8957	0.3098	100	1568.751	1567.743	7613.4	1	6.056	79.2	15
	K.HLVDEP	3.3684	0.3514	100	1305.713	1305.717	5745.3	1	6.679	75	6
	K.LGEYGF	3.7013	0.4067	100	1479.8	1479.796	7707.4	1	7.387	70.8	8
	R.KVPQVS	4.0649	0.5005	100	1639.941	1639.938	4039.3	1	8.379	53.6	15
	R.KVPQVS	4.6911	0.4474	100	1639.942	1639.938	6544	1	7.842	53.6	6
	R.KVPQVS	4.1862	0.4369	100	1643.952	1639.938	4918.3	1	7.888	78.6	36
	V.PQVSTF	2.8612	0.3318	99.9	1412.774	1412.775	5618.4	1	6.586	75	3
	R.MPCTED	4.8337	0.4523	100	1724.841	1724.835	8612.2	1	8.198	84.6	20
	R.RPCFSA	3.7822	0.2867	99.9	1880.924	1880.922	5196.6	1	5.3	46.7	3
	R.RPCFSA	3.8085	0.3258	100	1883.932	1880.922	4600.6	1	5.946	60	34
	K.KQTALV	3.1807	0.2363	99.9	1142.717	1142.715	5446.1	1	5.164	83.3	24
	K.TVMENF	3.1608	0.2971	99.9	1399.698	1399.693	9251.8	1	6.066	68.2	2
PFI1110w		180	32.50%	428	49704	8.1	U	Annotation|Plasmodium_falciparum_Sanger|(protein coc			
	K.NDLSSI	3.5489	0.293	99.9	1566.801	1566.799	5452	1	5.983	65.4	3
	K.VEPHKD	4.8541	0.3737	100	2647.224	2646.23	7701	1	7.273	47.6	1
	K.VEPHKD	5.0459	0.4607	100	2767.319	2767.308	6333.9	1	8.1	50	10
	K.VEPHKD	5.7081	0.2052	99.9	2776.343	2774.325	5516.7	2	5.581	36.4	2
	R.YHHEVS	4.713	0.4493	100	1801.896	1801.895	6655.5	1	8.881	71.4	73
	K.PLVNDN	4.6145	0.484	100	2052.025	2051.013	5802.4	1	7.632	70.6	5
	K.NIFYHN	4.6263	0.2653	100	1832.896	1829.886	7202	1	6.753	75	53
	K.RLVPGF	2.7728	0.3626	100	1334.688	1334.689	7535.3	1	5.676	65	6
	K.DNHFYIS	3.4334	0.3295	99.8	1471.73	1471.741	6783.4	1	7.332	68.2	3
	K.DNHFYIS	3.6879	0.3392	100	1471.748	1471.741	7809.2	1	6.403	72.7	9
	K.YVQHPE	6.6942	0.4347	100	3671.811	3670.801	8391.7	1	7.332	25	5
	K.YVQHPE	7.4294	0.5031	100	3674.801	3670.801	6768.2	1	7.967	35.8	10
PFA0460c		55	32.50%	166	19591	5.5	U	Annotation|Plasmodium_falciparum_Sanger|(protein coc			
	R.SKLNTL	6.0385	0.422	100	2116.146	2116.136	6772.9	1	7.984	70.6	3
	R.SKLNTL	5.2703	0.2994	100	2123.139	2123.153	8497.6	1	7.048	50	5
	K.LNTLKD	3.667	0.4216	100	1901.009	1901.009	6843.2	1	7.272	46.7	2
	K.LNTLKD	5.4808	0.3906	100	1911.03	1908.026	7633.9	1	7.92	76.7	9
	K.VVPHIN	2.9487	0.3904	99.8	1228.715	1228.713	4102.2	1	6.824	70	1
	K.TNYEEF	5.3841	0.4153	100	3120.661	3119.654	10470.5	1	7.499	36.5	1
	R.NEINIIN	5.8792	0.551	100	2180.251	2180.245	7868.2	1	9.776	73.5	34
MAL7P1.208		315	32.40%	786	95646	4.3	U	Annotation|Plasmodium_falciparum_Sanger|(protein coc			

E.KEEGE	5.2089	0.4028	100	2847.216	2846.209	8033.6	1	6.155	37.5	3
E.KEEGE	4.1827	0.4516	100	2848.214	2846.209	9011.6	1	7.339	45.5	3
K.NEESKN	4.8935	0.3111	100	3135.41	3135.403	6003.7	1	6.834	32.3	3
Y.KKKEED	3.789	0.3482	100	1917.924	1917.919	7212.4	1	6.821	43.3	3
Y.KKKEED	5.348	0.3791	100	1918.924	1917.919	6423.9	1	6.6	80	3
K.KKEEDE	3.548	0.3012	99.9	1789.826	1789.825	6086.7	1	5.178	41.1	2
K.KKEEDE	4.5061	0.4192	100	1790.83	1789.825	4942.5	1	7.53	64.3	6
K.KKEEDE	5.1361	0.3284	100	1790.832	1789.825	6812	1	6.092	85.7	36
K.KKEEDE	5.2307	0.4085	100	1918.922	1917.919	7061.6	1	6.707	70	9
K.KKEEDE	5.486	0.3818	100	1920.928	1917.919	5490.3	1	6.939	53.3	3
K.KEEDES	4.5019	0.3782	100	1790.829	1789.825	6231.4	1	6.312	48.2	3
K.KEEDES	4.8866	0.3108	100	1791.831	1789.825	6223.7	1	5.871	82.1	3
K.EEDESY	2.9997	0.33	99.9	1533.635	1533.635	4120.5	1	5.607	62.5	3
K.KNNTSN	3.6887	0.2659	100	1281.646	1281.644	6469.6	1	5.963	80	9
K.DLQGDS	4.8648	0.4515	100	1959.861	1956.846	8548.7	1	7.883	71.9	21
K.NVDKEN	3.8135	0.2398	99.9	2907.196	2906.192	10127	1	6.586	37	4
K.NVDKEN	4.1592	0.3419	99.9	2913.214	2913.209	10312	1	6.585	32.6	1
K.KMVFYD	4.6679	0.3027	100	2475.145	2475.143	6518.2	1	5.276	45.8	3
K.KMVFYD	5.4572	0.2868	100	2477.151	2475.143	6324.9	1	6.167	69.4	9
K.KMVFYD	4.3891	0.2696	100	2604.242	2603.238	6570.3	1	6.218	55.3	12
K.KMVFYD	4.8634	0.2899	100	2604.245	2603.238	7171.1	1	5.713	43	6
K.KMVFYD	5.0369	0.3302	100	2604.246	2603.238	7257.1	1	5.965	46.1	3
K.MVFYDL	5.4544	0.3266	100	2350.059	2347.048	4555.5	1	6.445	70.6	21
K.MVFYDL	4.2562	0.2753	100	2479.159	2475.143	5401.4	1	5.168	50	3
F.YDLYKP	4.1528	0.4172	100	1970.874	1969.871	3720.8	1	6.53	78.6	9
F.YDLYKP	4.241	0.2748	100	2097.97	2097.966	6227.4	1	5.662	56.7	3
F.YDLYKP	3.5228	0.3295	100	2100.975	2097.966	4825.1	1	5.866	60	6
Y.DLYKPE	3.2537	0.2752	99.8	1934.904	1934.902	4397.4	1	5.379	53.6	3
D.LYKPEE	4.4936	0.307	100	1693.789	1691.78	3189.8	1	5.259	83.3	3
D.LYKPEE	4.5475	0.3565	100	1819.876	1819.875	4031.1	1	6.032	84.6	6
K.KEEKEE	5.9393	0.1168	99.9	2006.014	2005.009	8052.6	1	4.706	58.3	3
K.KEEKEE	5.5604	0.2181	100	2008.021	2005.009	7690.7	1	6.114	73.3	9
K.EEKEEK	4.3579	0.1814	99.9	1877.922	1876.914	3901.2	3	5.394	60.7	3
K.SFDNEH	4.709	0.4251	100	2067.941	2067.937	9185.8	1	9.066	64.7	2
K.SFDNEH	5.2981	0.5061	100	2222.023	2222.023	9125.1	1	9.122	58.3	31
K.SFDNEH	4.3793	0.3463	100	2222.026	2222.023	6921.3	1	5.715	38.9	1

	K.SFDNEH	5.6521	0.3954	100	2599.285	2598.278		9915.8	1	7.081	36.9	1
	K.YLDDLI	4.4782	0.3576	100	1594.781	1594.785		7459.9	1	6.731	87.5	2
	K.YLDDLI	4.0517	0.3763	100	2008.016	2008.013		7204	1	7.181	62.5	9
	K.SQMILE	6.3409	0.3764	100	2775.204	2774.195		4951.5	1	8.531	56.8	27
	K.SQMILE	4.3254	0.2855	99.9	2775.205	2774.195		7079.1	1	5.54	30.7	2
	K.SQMILE	4.9387	0.3656	100	3534.566	3532.558		4375.7	10	5.307	22.3	8
	K.INDELL	3.9378	0.4176	100	2036.984	2035.979		6171.7	1	7.159	55.9	5
	K.INDELL	6.0623	0.2442	100	2500.188	2499.185		8765.9	1	8.904	57.1	7
	K.VQNKED	3.7892	0.2192	99.9	1620.757	1620.754		5803.1	3	5.485	70.8	3
PFD0810w		113	32.30%	192	22020	7.3	U	Annotation|Plasmodium_falciparum_Sanger|(protein coc				
	R.FKTFDL	3.8455	0.4535	100	1478.741	1478.739		6683.3	1	8.83	87.5	9
	K.TFDLGG	3.3467	0.369	100	1203.575	1203.576		5712.5	1	6.979	90	10
	R.HLLETE	6.1346	0.4467	100	2141.158	2138.15		6795.4	1	9.312	75	90
	K.IDKPDA	3.4856	0.2993	100	1465.721	1465.725		4531.5	1	6.943	79.2	1
	K.GGSGVI	3.6797	0.4625	100	1870.974	1870.975		6295.3	1	7.266	53.1	3
PF14_0038		43	32.20%	115	12894	9.4	U	Annotation|Plasmodium_falciparum_TIGR|(protein codin				
	M.SRPEP	4.0341	0.464	100	1856.957	1856.951		6172.2	1	7.783	63.3	3
	K.HLFEYL	3.6421	0.467	100	1273.703	1273.695		4761.9	1	7.91	83.3	38
	K.ERADLI	2.9879	0.2973	99.9	1377.769	1377.774		7297.6	8	4.724	55	2
PF13_0214		347	32.00%	434	50461	8.7	U	Annotation|Plasmodium_falciparum_Sanger|(protein coc				
	K.LNMPNF	3.2923	0.3066	99.9	1519.763	1519.758		6796.8	1	5.978	70.8	1
	R.LPVLVT	6.462	0.5664	100	2066.094	2065.086		10859.1	1	10.788	75	156
	R.LPVLVT	5.9938	0.4691	100	2068.096	2065.086		7944.8	1	8.031	45.8	14
	L.PVLVTS	3.9343	0.4047	100	1944.986	1944.985		6634.1	1	6.473	42.6	1
	L.PVLVTS	4.2597	0.3535	100	1952.032	1952.002		5119.7	1	7.241	61.8	3
	P.VLVTSH	3.3433	0.4337	100	1847.932	1847.933		5937.2	1	7.27	56.2	2
	K.KDDNN	4.7625	0.4723	100	3027.301	3026.296		7311.5	1	7.121	30.8	3
	K.KDDNN	4.2099	0.5118	100	3028.309	3026.296		5746.9	1	8.601	36.5	3
	K.DDNNN	4.1655	0.3327	100	2898.202	2898.201		5487.7	1	5.148	42	3
	K.DDNNN	3.4999	0.4953	100	2899.213	2898.201		7079.4	1	7.557	32	3
	K.KTNPLD	5.2729	0.3811	100	2214.148	2214.145		4104.6	1	6.814	58.3	70
	K.TNPLDL	3.8474	0.2406	99.8	2089.057	2086.05		3425.4	4	4.774	52.9	2
	K.FSNEKD	4.4113	0.2355	99.9	2091.027	2091.012		7146.1	1	6.589	50	3
	K.FSNEKD	4.0623	0.4076	100	2091.027	2091.012		7731.4	1	7.28	65.6	6
	K.DLLNNA	4.264	0.3274	100	1488.74	1485.731		5520.9	1	5.933	77.3	37
	K.YSFAVV	3.5511	0.3735	100	1426.758	1426.758		8999.5	1	6.84	79.2	6

	R.GTDIPF	4.6378	0.4013	100	2452.183	2452.183		3348.3	1	6.784	40.8	1	
	K.DHPSFE	3.8247	0.4254	100	1429.695	1426.687		4633.5	1	6.999	70	30	
	K.KLDINNT	3.7048	0.1703	99.8	1324.739	1323.735		6105	1	3.963	85	3	
PF07_0054		87	31.70%	123	13763	10.8	U		Annotation|Plasmodium_falciparum_Sanger|(protein coc				
	R.TESFSL	3.3077	0.4139	100	1242.657	1241.653		4979.7	1	6.173	83.3	1	
	K.VLKQVH	2.8023	0.3308	99.8	1435.829	1435.827		5752.3	1	6.014	62.5	3	
	L.KQVHPE	3.188	0.2525	99.9	1223.676	1223.675		7297.4	3	5.57	70	3	
	K.KSMNIM	4.8346	0.5277	100	1965.987	1965.983		8094.1	1	9.066	83.3	9	
	K.SMNIMN	5.9993	0.5098	100	1840.892	1837.888		9449.5	1	9.514	82.1	62	
	M.NIMNSF	4.0837	0.4071	100	1620.82	1619.815		7492	1	7.871	87.5	9	
PFC0120w		437	31.50%	1417	167241	7.2	U		Annotation|Plasmodium_falciparum_Sanger|(protein coc				
	K.NITDAD	4.0389	0.3526	100	2766.416	2763.41		8148.9	1	6.43	37	3	
	K.EQSIEIY	3.4831	0.4646	100	1640.767	1640.766		3503.6	1	8.317	53.8	2	
	K.SALEEL	4.21	0.3231	100	1478.743	1478.749		8227.7	1	7.65	79.2	21	
	K.EAQEES	4.5798	0.5468	100	1906.856	1906.857		5205.4	1	9.076	68.8	6	
	R.TIKENE	5.1659	0.3774	100	2394.271	2394.266		5081.1	1	8.516	55	5	
	R.SNTSME	3.5376	0.4119	100	2384.137	2383.118		8340.3	1	5.991	47.2	1	
	T.SMECNI	3.4159	0.2734	99.8	2081.998	2080.995		6121.7	1	5.694	53.3	1	
	K.ECIIYHT	3.6596	0.4004	100	1452.689	1452.695		5093.4	1	6.477	80	5	
	K.ECIIYH	3.9091	0.2776	100	1594.826	1594.824		5447.4	1	6.121	81.8	2	
	K.LFESLIQ	3.8072	0.189	99.9	1379.726	1379.725		8226.7	2	5.423	85	2	
	K.TVVTNY	4.4213	0.4027	100	1575.837	1574.833		8508.5	1	6.999	66.7	2	
	K.TVVTNY	3.9066	0.3643	100	1695.9	1695.911		8408.2	1	7.35	61.5	30	
	R.KHIPNN	4.0163	0.1644	99.8	1556.861	1555.856		4913	1	4.949	79.2	8	
	N.SFLQLD	3.8874	0.4216	100	2368.247	2368.245		8062.1	1	7.865	47.4	9	
	R.SYPLSL	3.5302	0.3216	100	1285.716	1285.716		3991.9	2	6.215	60	23	
	Y.PLSLVL	2.9956	0.369	100	1035.618	1035.62		6176.3	1	5.817	81.2	4	
	K.SLIECA	4.2174	0.4713	100	1831.803	1831.811		9030.3	1	8.556	76.9	3	
	K.YHNIYN	3.5188	0.3115	100	1306.673	1306.673		5311.2	1	5.617	83.3	3	
	K.NNFDDV	5.922	0.5351	100	1986.94	1984.934		8343.9	1	9.914	83.3	54	
	R.VYHTLH	4.2362	0.4561	100	1988.018	1987.015		4890.5	1	7.743	59.4	28	
	Y.HTLHSI	3.0888	0.4301	100	1724.885	1724.883		3548.1	1	5.837	50	6	
	H.TLHSIT	3.2873	0.3971	100	1587.824	1587.825		6747.5	1	7.105	61.5	1	
	K.ISVNNV	3.6673	0.4171	100	1967.976	1967.976		6935.5	1	7.122	62.5	3	
	K.NLSLLC	4.8184	0.4465	100	2250.094	2249.091		8038.8	1	7.982	60.5	33	
	F.TTGQHL	4.3181	0.3671	100	1961.967	1960.973		5005.8	1	7.376	52.6	2	

	K.SPESFF	5.7206	0.3941	100	1826.879	1825.876		10007	1	8.874	75	75
	S.PESFFF	4.5837	0.4891	100	1739.846	1738.844		6896.7	1	8.054	76.7	6
	K.EQTQH\	3.3243	0.4398	100	1675.771	1675.768		5248	1	7.623	66.7	10
	K.IMNYVH	4.9619	0.44	100	2079.966	2078.964		6504.6	1	8.106	71.9	4
	K.IMNYVH	4.1477	0.2969	100	2079.969	2078.964		8215.5	1	5.558	43.8	2
	R.RYDMEI	2.8216	0.2537	99.8	1173.537	1173.536		5744.7	1	6.21	81.2	2
	R.YDMEN/	2.9672	0.2396	99.9	1017.437	1017.435		4072.6	6	4.958	85.7	3
	K.MSTYVD	3.9394	0.5444	100	1917.769	1917.768		7585.6	1	9.208	73.3	9
	K.MSTYVD	4.5561	0.4408	100	2949.257	2948.251		5827.3	1	7.407	43.5	22
	K.MSTYVD	4.6074	0.5463	100	2950.264	2948.251		7864.8	1	9.272	47.8	18
	K.NEQFLN	2.5527	0.2795	99.8	1049.502	1049.502		5679.9	1	5.132	85.7	2
	R.CDYCP\	4.9066	0.5226	100	2641.176	2641.176		5572.6	1	8.947	47.6	4
	R.CDYCP\	3.8037	0.4066	100	3092.398	3091.388		4575.8	1	6.147	27	2
	K.KQTSTY	3.1706	0.3731	100	1320.677	1320.676		7132.1	5	5.746	65	3
	K.MNEADS	7.209	0.5708	100	2909.173	2908.17		9414.4	1	9.695	41	6
	K.MNEADS	5.4073	0.5886	100	2909.175	2908.17		8808	1	9.749	44	12
MAL7P1.77		141	31.40%	522	60601	4.5	U		Annotation|Plasmodium_falciparum_Sanger|(protein coc			
	R.LDDTSS	4.0904	0.2525	99.9	1781.794	1780.784		6728.7	8	5.261	63.3	3
	R.HNNNDI	4.0655	0.2567	100	1523.697	1523.695		5564.3	1	6.858	70.8	30
	K.NENNNS	5.3995	0.3888	100	1999.887	1999.885		4631	1	8.096	68.8	14
	K.MHVPSE	6.4634	0.5042	100	4944.351	4941.321		10509.8	1	8.666	25	16
	E.SFMDYI	5.5884	0.5903	100	3021.481	3021.478		9074.8	1	10.539	48	10
	E.SFMDYI	5.3904	0.5241	100	3022.491	3021.478		8940.8	1	8.897	36	3
	R.ETNIRP	5.1344	0.4079	100	2135.998	2132.982		3808.5	1	6.606	55.9	24
	R.ETNIRP	6.3326	0.2928	100	2145.013	2139.999		4194.4	1	7.022	51.5	14
	K.QQSLFD	4.6065	0.4359	100	2264.089	2263.084		8509	1	7.512	61.1	12
	K.QQSLFD	3.5532	0.2743	99.9	2385.172	2384.162		4831.8	1	4.913	57.9	2
	K.LNSEDK	6.3735	0.3443	100	2831.21	2830.205		9989	1	7.155	38.6	2
	K.LNSEDK	4.4248	0.3977	100	2831.214	2830.205		8766.9	1	8.149	45.5	2
	K.LNSEDK	5.8165	0.389	100	2966.323	2965.317		8906	1	7.136	33.7	3
	K.LNSEDK	4.0256	0.3545	100	2967.328	2965.317		7122.4	1	6.058	34.8	3
	K.DTCFLD	3.5987	0.2999	99.9	1842.848	1842.845		9019.5	1	5.721	61.5	3
PF13_0180		20	31.40%	258	29063	7.9	U		Annotation|Plasmodium_falciparum_Sanger|(protein coc			
	R.GPLTPL	4.0696	0.4072	100	1612.953	1612.95		4401.7	1	6.788	73.1	4
	K.DEAGDT	5.4763	0.4868	100	1976.967	1975.957		7920.3	1	8.297	66.7	1
	K.VLSVGA	3.0427	0.3482	100	1136.676	1136.675		5739.3	1	7.568	77.3	3

	K.INDSNE	6.3083	0.5379	100	2122.115	2122.108		8155	1	9.752	64.7	7	
	K.LINPNVI	4.9591	0.4273	100	1994.161	1994.155		7920.3	1	6.724	61.8	5	
PFL2095w		8	31.30%	115	12891	9.5	U		Annotation|Plasmodium_falciparum_Sanger|(protein coc				
	K.SNATNL	3.3445	0.2883	100	1152.674	1152.668		8253.1	1	6.611	83.3	7	
	K.KEFNCN	3.9753	0.3963	100	3049.536	3049.586		3503.5	4	5.837	25	1	
PF13_0213		23	31.20%	221	25532	10.1	U		Annotation|Plasmodium_falciparum_Sanger|(protein coc				
	K.ILNSGL	5.323	0.5798	100	2181.291	2180.288		6152.1	1	8.61	55	6	
	R.YLVVTS	4.0769	0.4133	100	1988.077	1987.073		6918.8	1	7.021	50	1	
	K.LKDDFL	5.9579	0.4724	100	2289.113	2289.111		3453.9	1	7.78	72.2	8	
	K.DDFLNY	5.3778	0.4445	100	2047.931	2047.932		6992.3	1	8.643	78.1	6	
	K.DIGSLLI	3.6179	0.2067	99.9	1339.839	1339.839		7757	1	5.179	81.8	2	
PFI1780w		252	31.10%	383	45500	9.7	U		Annotation|Plasmodium_falciparum_Sanger|(protein coc				
	K.KYTAEE	5.3934	0.4442	100	2403.139	2402.13		8630.9	1	7.892	60.5	7	
	K.YTAEEI	5.8123	0.4943	100	2296.093	2295.086		8185.9	1	9.25	63.9	24	
	R.NDMNII	5.061	0.5161	100	1867.886	1867.884		6692.2	1	8.327	71.4	18	
	K.FIQSIVE	3.5676	0.2351	99.9	1228.666	1227.663		5555.3	1	4.604	83.3	3	
	K.FIQSIV	5.2654	0.5213	100	1995.083	1994.079		7536.5	1	8.514	73.3	3	
	K.FAHFEN	3.8161	0.326	100	1443.701	1441.694		4648.6	1	5.567	86.4	12	
	K.FAHFEN	4.6638	0.4837	100	1826.892	1826.89		8748.6	1	8.573	78.6	3	
	K.FLLEYA	4.9945	0.3212	100	1517.84	1514.826		9899	1	6.892	87.5	37	
	K.FKEVLE	3.2257	0.2279	99.9	1154.646	1154.646		4528.1	2	5.176	81.2	10	
	K.FKEVLE	3.5055	0.3613	100	1310.749	1310.747		5765.9	1	6.007	88.9	15	
	K.FKEVLE	4.2723	0.2737	100	1311.751	1310.747		4746.9	1	5.881	75	15	
	K.GYLGEV	3.5814	0.2794	99.9	1459.778	1459.776		7301.9	1	6.456	70.8	6	
	K.KKTEHH	6.3079	0.1935	100	2176.086	2175.079		7899.3	1	5.601	55.9	9	
	K.KKTEHH	5.6028	0.3007	100	2176.087	2175.079		7854.3	1	6.219	70.6	18	
	K.KTEHHE	4.4016	0.3943	100	1824.848	1821.837		6752.6	1	7.113	67.9	9	
	K.KTEHHE	5.981	0.238	100	2047.989	2046.985		7449.7	1	5.665	51.6	3	
	K.KTEHHE	5.2471	0.2649	100	2047.993	2046.985		8406.1	1	6.8	75	32	
	K.TEHHEN	5.2697	0.3318	100	1918.887	1918.89		9537.8	1	7.818	70	25	
	K.TEHHEN	5.185	0.3653	100	1919.893	1918.89		7422.2	1	6.46	48.3	3	
MAL7P1.117		9	31.10%	106	12369	4.1	U		Annotation|Plasmodium_falciparum_Sanger|(protein coc				
	K.NMQDN	4.3388	0.3345	100	2574.106	2573.1		6009	1	5.596	39.3	3	
	K.NMQDN	3.9363	0.374	100	2574.106	2573.1		7605.6	1	5.987	42.9	3	
	K.ANMEAE	3.0496	0.2627	99.8	1262.606	1262.605		9121.8	1	5.387	80	3	
PFF0510w		168	30.90%	136	15446	11.1	U		Annotation|Plasmodium_falciparum_Sanger|(protein coc				

	R.KSAPIS/	2.7757	0.3457	99.9	986.6245	985.6233		3531.9	1	5.799	83.3	3
	R.FQSSAV	3.8633	0.3118	100	3497.688	3497.709		7096.3	1	6.205	24.2	7
	R.FQSSAV	6.1174	0.3097	100	3506.738	3504.726		9040	1	5.887	26.9	38
	R.FQSSAV	8.0874	0.4836	100	3508.742	3504.726		7205.4	1	8.374	29.8	58
	Q.SSAVM/	4.9944	0.4031	100	3222.571	3222.582		7606	1	6.379	25.9	4
	S.SAVMAL	4.7005	0.2992	100	3136.563	3135.55		8539.1	1	5.55	30.4	4
	V.MALQE/	4.7679	0.4806	100	2886.433	2885.429		7292.2	1	7.315	42	21
	M.ALQEA/	4.7064	0.3588	100	2748.379	2747.371		4922.3	1	6.518	41.7	19
	A.LQEAAE	3.9012	0.3183	100	2684.358	2683.352		6805.8	1	6.518	41.3	6
	L.QEAAEA	3.2973	0.3234	99.9	2571.272	2570.267		7338.2	1	6.126	36.4	8
PF08_0059		54	30.80%	130	14700	6.4	U	Annotation|Plasmodium_falciparum_Sanger|(protein coc				
	R.GEVPVD	4.2752	0.3839	100	1435.663	1435.659		4762.4	1	7.004	79.2	3
	R.GEVPVD	6.1968	0.5272	100	3700.032	3699.017		6863.6	1	7.699	28.9	15
	K.VIAFNDI	5.0139	0.4095	100	2247.303	2247.291		4572.6	1	7.833	55.3	22
	K.VIAFNDI	4.1591	0.3784	100	2247.306	2247.291		4983	1	6.48	36.8	8
	F.NDIYPQ/	3.7553	0.4545	100	1845.105	1845.101		5959.4	1	7.158	63.3	5
	K.HKEILGI	2.5108	0.2891	99.9	840.482	840.4805		3119.8	2	5.967	83.3	1
PFF0940c		197	30.60%	806	89891	5.1	U	Annotation|Plasmodium_falciparum_Sanger|(protein coc				
	R.LIVEEA	4.7627	0.4702	100	2052.059	2052.057		6232.7	1	8.172	63.9	28
	K.RMEELN	3.285	0.2996	100	1241.611	1241.61		6930.1	1	5.431	87.5	15
	R.MEELNF	2.9247	0.2359	99.9	1085.512	1085.509		4406.6	1	5.667	92.9	1
	K.IQVLPID	4.3748	0.4213	100	1645.973	1645.968		3718.4	1	7.342	75	5
	K.DTLFEI	4.2374	0.2588	99.9	2507.307	2506.302		7155.9	1	6.177	39.5	2
	K.VVEVDF	3.2241	0.4	100	3142.527	3140.514		5175	1	6.313	34.6	2
	K.VVEVDF	5.9302	0.319	100	3162.575	3161.565		4190.2	1	6.755	31.7	1
	R.DDEEKL	4.8139	0.5369	100	2199.985	2198.976		7912	1	8.035	66.7	4
	R.DDEEKL	4.8407	0.3677	100	2213.016	2213.011		7100.9	2	6.7	38.9	3
	R.AVANET	3.4889	0.3262	99.9	2244.112	2242.122		5944.8	1	5.823	45	2
	R.EMILYP	3.8663	0.3866	100	1874.939	1874.936		5598.3	1	6.208	64.3	2
	R.AAAPCV	4.1136	0.4265	100	2117.052	2117.045		7573.1	1	8.522	40.3	3
	R.AAAPCV	5.4126	0.5408	100	2120.057	2117.045		7422.5	1	10.139	66.7	62
	F.FIGATN	4.775	0.349	100	2238.225	2238.236		8548.6	1	6.285	35.5	11
	F.IGATNR	4.9724	0.5025	100	2098.183	2098.185		7801.6	1	7.848	36.1	10
	F.IGATNR	3.9424	0.4087	100	2098.184	2098.185		5351.8	3	6.814	44.4	8
	G.ATNRPE	3.5366	0.3146	99.9	1921.063	1921.062		4797.9	2	5.302	56.2	3
	G.ATNRPE	4.3286	0.3806	100	1921.065	1921.062		7000.3	1	6.984	50	9

	R.KCPVAE	4.3951	0.3745	100	1828.966	1828.963		8272.6		1	7.383	60	4
	K.CPVAEN	4.7939	0.2877	100	1709.874	1707.885		4770.9		1	5.972	67.9	7
	K.TAGFSG	4.2873	0.317	100	1595.752	1595.749		8589.4		1	6.812	71.4	4
	K.TGGTGD	5.3314	0.465	100	3566.454	3563.477		5945.6		1	7.407	24.2	11
PF10_0325		153	30.60%	288	32841	5.9	U		Annotation|Plasmodium_falciparum_TIGR|(protein codin				
	K.IIFTDL	5.0524	0.3304	100	1794.941	1792.933		9920.9		1	6.033	83.3	5
	K.VSEQNL	3.5643	0.3133	100	1294.704	1294.708		7724.2		1	6.417	75	1
	R.SIFSVE	4.0331	0.4192	100	1572.856	1571.862		9118.8		1	6.89	65.4	14
	R.SIFSVE	4.1146	0.3405	100	1699.959	1699.957		10297.4		1	6.337	53.6	14
	S.VENVIG	3.4731	0.3186	100	1258.718	1258.723		8389.8		1	6.394	70	1
	K.VLFSLP	2.8969	0.3978	99.8	1737.006	1736.991		4439.9		1	6.686	53.6	1
	K.VLFSLP	4.5483	0.4292	100	1740.002	1736.991		3457.2		1	7.682	75	51
	T.HSVGVH	3.0993	0.3677	100	1164.57	1164.576		4756.1		1	7.095	85	3
	K.NSAAYV	5.2464	0.4533	100	2115.075	2115.07		7208.3		1	9.651	52.6	62
	V.GPSNNE	3.6112	0.428	100	1509.793	1509.789		5010.7		1	7.105	65.4	1
MAL13P1.92		187	30.60%	147	17040	10.2	U		Annotation|Plasmodium_falciparum_Sanger|(protein coc				
	K.NCEPGE	4.1242	0.4163	100	1844.909	1844.908		6249.6		1	8.245	66.7	165
	K.PNPVPT	2.55	0.3243	99.8	1030.579	1030.58		4036.2		8	5.186	75	3
	R.NMTIIP	3.8522	0.4479	100	2032.106	2031.103		4825.5		1	6.955	55.6	15
	K.PGIGAT	2.9748	0.4244	100	989.5231	989.5242		4470.9		1	6.844	83.3	4
PF11_0301		59	30.50%	321	36596	7.7	U		Annotation|Plasmodium_falciparum_TIGR|(protein codin				
	K.SKYQNV	2.9387	0.3185	99.8	1864.945	1863.949		7036.5		1	5.203	56.7	3
	K.SVENIDI	2.7532	0.3656	99.9	2192.055	2192.065		4429.1		4	5.301	38.9	2
	K.RVNVFI	3.229	0.2701	99.9	1277.686	1277.685		6023.4		1	6.395	75	3
	D.PIGPAE	4.1806	0.3472	100	1877.945	1874.94		8020.8		1	5.896	76.7	3
	K.KVEYAN	4.1829	0.5184	100	2831.414	2831.398		4227.9		1	7.315	43.5	14
	K.YYNYEN	4.1439	0.5448	100	1506.666	1506.665		10202.8		1	9.544	86.4	27
	K.YYNYEN	3.4258	0.5117	100	1506.669	1506.665		4249.2		1	8.001	59.1	7
PFF0885w		25	30.40%	148	16723	10.5	U		Annotation|Plasmodium_falciparum_Sanger|(protein coc				
	R.TYCPTI	3.4814	0.3331	100	2276.167	2276.164		7408.6		1	6.001	44.4	2
	K.KKEFSE	3.9141	0.4349	100	2090.135	2089.129		5825.7		1	6.894	44.1	2
	K.KKEFSE	7.1891	0.5075	100	2106.174	2103.164		10689.2		1	10.008	73.5	13
	K.EFSENK	3.9047	0.3066	100	1832.941	1832.94		6312.2		1	7.308	60	3
	K.HNQPIV	2.8687	0.3081	99.9	934.549	934.5474		4613.1		1	6.528	85.7	5
PFF0340c		40	30.10%	219	26035	5.7	U		Annotation|Plasmodium_falciparum_Sanger|(protein coc				
	K.GYQLFY	4.1943	0.4228	100	1507.746	1507.743		5902.5		1	7.983	79.2	12

	K.SFVNTN	4.3862	0.4751	100	1655.881	1655.876	6625.1		1	8.274	75	15	
	K.FSNAVI	4.7476	0.4088	100	1504.756	1501.755	7058.2		1	6.978	83.3	6	
	K.IYSNWP	4.145	0.4933	100	2983.632	2983.606	5043.4		1	7.507	35.4	7	
PF10_0210		108	30.00%	263	28980	7.2	U	Annotation|Plasmodium_falciparum_TIGR|(protein codin					
	K.TCPFAA	4.874	0.3941	100	1823.92	1823.919	7523.7		1	7.656	63.3	4	
	K.TCPFAA	4.7004	0.3547	100	1826.925	1823.919	6203.1		1	6.404	73.3	72	
	K.VLEDGV	3.9617	0.3432	100	1834.941	1833.949	10018.3		1	6.543	63.3	2	
	K.KVLQNV	3.1824	0.3802	100	1256.715	1256.721	6955.9		1	5.922	70	3	
	K.VLQNVE	4.1647	0.4014	100	1901.025	1901.023	5980.2		1	6.527	59.4	6	
	K.TSSGKV	3.6176	0.4454	100	1512.84	1512.839	4624.1		1	7.902	56.7	3	
	K.TSSGKV	3.4427	0.3397	100	1512.84	1512.839	3950.7		1	6.023	60	3	
	R.FLSALA	3.7391	0.3891	100	1546.761	1546.759	7242.4		1	7.41	79.2	15	
PFI0895c		54	29.70%	320	36820	6.7	U	Annotation|Plasmodium_falciparum_Sanger|(protein coc					
	K.IPQHFD	3.706	0.4131	100	1680.974	1680.974	8256.5		1	6.305	69.2	1	
	I.PQHFDIL	3.7785	0.2928	100	1546.839	1546.838	6971.9		1	6.733	79.2	5	
	R.DQVVG	3.8391	0.4172	100	2886.306	2885.303	6609.4		1	7.96	41.7	12	
	R.DQVVG	3.6334	0.346	99.8	2886.311	2885.303	3566		1	5.599	33.3	1	
	V.GWFCS	3.7101	0.4731	100	2445.052	2444.08	4834.3		1	8.042	50	18	
	K.AYVQLP	3.0886	0.257	99.9	1237.764	1237.763	6773		1	5.823	70	1	
	K.DYFVHF	4.4596	0.4166	100	2554.239	2553.231	7671.4		1	7.233	52.6	14	
	K.DNHTNN	6.2103	0.3463	100	2987.388	2986.383	5999		1	6.698	37.5	2	
PFE0965c		48	29.70%	165	17065	8.3	U	Annotation|Plasmodium_falciparum_Sanger|(protein coc					
	R.QCDPNS	4.5257	0.5065	100	3185.515	3184.487	9224.8		1	9.477	30	4	
	K.SGVGVC	5.1309	0.4949	100	1911.996	1911.993	8240.7		1	8.849	67.6	44	
PF10_0187		11	29.60%	108	11775	9.4	U	Annotation|Plasmodium_falciparum_TIGR|(protein codin					
	K.LVIVSS	3.8948	0.3044	100	1488.832	1486.824	6561.7		1	6.355	75	1	
	K.CGVHD	4.2509	0.5087	100	2090.872	2089.846	4218.4		1	8.264	55.6	10	
PFE0625w		29	29.50%	200	22886	6.6	U	Annotation|Plasmodium_falciparum_Sanger|(protein coc					
	K.ILLIGDS	3.253	0.2667	99.9	1085.678	1085.676	4637.7		1	5.499	80	1	
	R.NVSYEE	5.1088	0.4226	100	2733.281	2732.273	10818.8		1	7.517	39.1	7	
	K.IAHNVE	3.6918	0.3684	100	1163.629	1163.629	5678.6		1	6.518	88.9	12	
	K.TNINLN	4.028	0.2929	100	1611.938	1611.937	7290.3		1	5.843	65.4	9	
PFD0895c		103	29.40%	187	21316	4.6	U	Annotation|Plasmodium_falciparum_Sanger|(protein coc					
	K.VNSELF	4.164	0.3142	100	2117.14	2116.133	6665.2		1	5.338	34.7	5	
	K.VNSELF	5.8134	0.3351	100	2120.153	2116.133	11418.7		1	6.705	66.7	36	
	K.VNSELF	4.2099	0.325	100	3771.959	3769.943	8347.6		1	5.135	27.3	3	

	S.FTYGAL	3.753	0.3956	100	1339.765	1339.763		8427.4	1	6.819	86.4	12
	K.ESNIFS	5.4997	0.4115	100	2542.292	2538.277		7372.9	1	7.394	61.9	47
MAL7P1.141		10	29.40%	109	13000	9.7	U		Annotation|Plasmodium_falciparum_Sanger|(protein coc			
	L.RLDTPE	4.7913	0.5075	100	2222.064	2222.062		7079.9	1	9.648	61.1	6
	K.DKHGNI	3.5907	0.359	100	1493.808	1493.819		7068	1	5.757	75	1
	K.HGNLIT	3.52	0.4065	100	1243.688	1243.68		6465.4	1	8.374	90	3
PF11_0351		107	29.30%	663	73298	6.9	U		Annotation|Plasmodium_falciparum_TIGR|(protein codin			
	R.ASGDII	3.5864	0.4219	100	2322.12	2322.132		7944.9	1	7.046	40.9	2
	K.KYSPSQ	3.5621	0.3946	100	1586.824	1586.833		6050.2	1	6.653	73.1	2
	K.VHQAVI	4.8559	0.31	100	1947.999	1944.993		4999	1	7.049	65.6	2
	K.ATNGNT	3.2815	0.2779	99.8	1681.747	1681.742		6874.8	1	5.616	56.7	3
	R.ILEYFIS	3.5843	0.3395	100	1288.685	1288.683		6273.1	1	6.14	88.9	2
	R.AKLEEL	4.3763	0.3001	100	1468.778	1468.783		9937	1	6.721	81.8	3
	K.DLLLLD	4.0216	0.4754	100	2427.428	2426.416		7011.1	1	7.939	33	3
	K.DLLLLD	6.5573	0.5676	100	2444.457	2440.45		4774.5	1	9.899	70.5	56
	I.PLSLGIE	3.7474	0.4441	100	1532.882	1531.874		3498.4	1	7.262	53.6	2
	K.NLTDSK	5.4469	0.5083	100	2043.003	2041.993		7841.2	1	9.099	73.5	7
	K.NEAETL	3.5815	0.4074	100	1390.684	1390.682		9322	1	7.448	81.8	3
	K.DKISDS	3.037	0.3771	99.8	1420.695	1420.692		4036.2	2	6.131	63.6	1
	K.ISDSDK	3.6628	0.2632	100	1177.572	1177.57		6542.3	1	5.716	88.9	5
	K.LTSEDL	3.9587	0.3755	100	1535.785	1535.78		7845.3	1	6.926	69.2	6
	K.SWAISQ	3.0579	0.3313	100	1249.604	1249.6		5030.6	1	6.119	77.8	1
	K.NNAQQ	4.5434	0.4973	100	2783.232	2783.222		6124.8	1	7.776	37.5	9
PFC0350c		234	29.30%	539	59577	5.6	U		Annotation|Plasmodium_falciparum_Sanger|(protein coc			
	R.NINACQ	3.9591	0.3859	100	1527.887	1527.894		7943.5	1	7.079	79.2	5
	N.ISHPAAS	3.2002	0.4975	100	1434.859	1434.832		5181.8	1	7.772	57.7	2
	N.ISHPAAS	3.9538	0.4013	100	1455.888	1455.884		4203.1	1	6.909	73.1	15
	K.SQDDEV	4.8812	0.3839	100	2533.24	2532.232		8541.9	1	6.372	34.4	11
	K.SQDDEV	5.7458	0.4862	100	2535.243	2532.232		9859.5	1	9.227	58.3	74
	K.GLLNDG	4.3425	0.526	100	1873.952	1873.948		6755.2	1	8.799	62.5	11
	R.IENPNE	4.4996	0.4093	100	2451.271	2450.262		7485	1	7.534	44.7	6
	K.LPIGDI	5.1677	0.5792	100	2385.196	2385.196		9102.7	1	9.822	50	69
	K.LPIGDI	4.6848	0.4139	100	2385.202	2385.196		5448.9	1	6.217	40	9
	L.PIGDIA	4.5409	0.4521	100	2272.092	2272.112		6187.8	1	8.515	55.3	1
	R.HLSHNA	5.7271	0.5829	100	1791.874	1791.874		7836.3	1	11.046	80	29
	K.HSEQTS	4.5455	0.4294	100	3811.627	3809.641		7808.9	1	6.715	29.8	2

PF14_0678		261	29.30%	287	33412	5.3	U		Annotation|Plasmodium_falciparum_TIGR|(protein codin				
	T.TVIKDPI	3.1075	0.3364	99.9	1327.823	1327.82		5677.5	1	6.275	77.3	4	
	R.EIWDQC	4.5035	0.4847	100	2483.214	2482.211		7937.2	1	8.083	55	15	
	R.EIWDQC	3.6329	0.3016	100	2954.528	2952.501		7686.5	1	5.595	45.8	4	
	R.EIWDQC	8.0132	0.5956	100	4053.082	4052.067		6511.5	1	9.181	27.3	46	
	R.EIWDQC	4.5081	0.2755	99.9	4057.067	4052.067		4904.9	1	5.519	21.7	5	
	K.PLLFLH	4.9879	0.5293	100	1588.875	1588.875		6521.9	1	9.819	83.3	56	
	L.LFLHGI	3.0878	0.3765	100	1378.737	1378.738		5690.4	1	6.76	75	2	
	L.FLHGIL	2.7248	0.4713	100	1265.655	1265.654		5402.5	1	7.43	72.2	1	
	L.HGILNE	3.1936	0.3324	100	1005.501	1005.501		6207.8	1	6.344	92.9	21	
	K.LRQDPS	4.0319	0.2408	100	1239.744	1239.742		8513.3	1	5.327	67.5	2	
	K.LRQDPS	3.3989	0.3811	100	1247.763	1246.76		5458.6	1	6.132	75	13	
	R.SHTLYT	4.9654	0.5086	100	2025.089	2025.088		8091.2	1	8.929	64.7	43	
	Y.THITPD	3.7342	0.4039	100	1416.784	1416.785		5798	1	7.384	79.2	32	
	T.HITPDA	3.3845	0.3729	100	1323.759	1322.755		5410.6	1	5.858	81.8	16	
	K.HLSSYM	2.6916	0.2768	99.8	1123.507	1123.509		4651.2	1	6.109	87.5	1	
PF14_0075		299	29.20%	449	51046	5.5	U		Annotation|Plasmodium_falciparum_TIGR|(protein codin				
	K.HLYDAS	3.1385	0.3095	100	991.4851	991.4849		3888.1	1	6.553	93.8	3	
	K.DVISLG	3.5204	0.2864	99.9	1419.775	1419.774		7186.2	1	5.568	83.3	7	
	K.DLSIGS	3.8504	0.4774	100	1726.021	1726.019		7045.6	1	8.569	60	1	
	K.IDNALF	4.3252	0.4888	100	1799.946	1799.945		8021.1	1	7.746	75	48	
	K.IDNALF	4.2428	0.3643	100	1799.947	1799.945		6820.3	1	5.888	44.6	1	
	K.HVGYLT	5.3221	0.5361	100	2603.293	2602.29		5208.6	1	8.59	54.5	11	
	K.ANAVVD	6.466	0.5306	100	2095.077	2094.072		5668.1	1	9.069	67.5	24	
	K.VPFLPL	5.8734	0.3695	100	2850.392	2849.382		5258.3	1	6.981	38	42	
	K.VPFLPL	4.589	0.3382	100	2853.395	2849.382		8272.9	1	6.237	45.7	139	
	V.PFLPLY	3.6068	0.2717	99.8	2752.319	2750.314		5726	1	5.803	38.6	6	
	L.PLYVTT	4.3381	0.5098	100	2393.109	2393.108		5636.4	1	8.397	55.3	16	
	R.KYFTVF	2.992	0.2876	99.9	1339.658	1339.658		5568.2	1	5.138	83.3	1	
PFL2055w		22	29.20%	137	16067	10.4	U		Annotation|Plasmodium_falciparum_Sanger|(protein coc				
	K.ITEEVAI	3.02	0.3428	99.9	1376.837	1376.841		7235.2	1	6.048	72.7	1	
	K.SQIDVS	4.1456	0.3479	100	1833.945	1833.96		6495.4	1	6.923	80	18	
	K.VHNPMI	3.4011	0.3942	100	1430.73	1430.729		4755	1	5.663	77.3	3	
PFE0185c		9	29.20%	120	14065	10.1	U		Annotation|Plasmodium_falciparum_Sanger|(protein coc				
	R.KRNEDE	3.3814	0.2475	99.9	1377.666	1377.661		5983.2	1	5.647	85	3	
	K.MYTIVE	4.6262	0.5016	100	1715.823	1714.819		8634	1	7.875	80.8	3	

	K.GLVNEC	2.9616	0.2853	99.9	1134.476	1134.474	5369.3	1	5.739	72.2	3
MAL13P1.308		1460	29.10%	2605	291021	5.2	U	Annotation|Plasmodium_falciparum_Sanger|(protein coc			
	K.LLDYCL	4.3144	0.4612	100	3155.664	3154.624	6378.5	1	6.54	25	3
	K.LLDYCL	5.8957	0.5437	100	3156.611	3154.624	11382.4	1	10.013	40.4	62
	D.YCLNIN	3.1427	0.3008	99.9	2814.455	2813.429	6187.6	1	5.964	34.8	1
	C.LNINSL	3.8409	0.365	100	2464.263	2462.267	8708.8	1	5.919	52.4	2
	K.VVESGC	6.3216	0.5181	100	2934.586	2933.577	12137.2	1	9.569	34.6	9
	K.VVESGC	5.8398	0.4785	100	2937.596	2933.577	9877.7	1	8.482	48.1	34
	R.SNYVSE	5.1433	0.4311	100	3449.808	3447.794	5461.6	1	7.7	36.7	19
	R.SNYVSE	6.1669	0.466	100	3466.84	3461.828	11048	1	8.494	30	15
	R.LSDYIT	3.4783	0.4018	100	3331.746	3330.737	4756.6	1	6.726	27.4	1
	K.LNASAT	4.8547	0.5157	100	2592.386	2591.365	7257.3	1	8.924	47.8	9
	K.INSAEIV	4.1152	0.3313	100	1870.983	1870.988	9946.7	1	6.25	53.3	3
	R.LTQINLI	4.9792	0.506	100	2109.232	2108.205	7254.7	1	8.275	69.4	13
	L.SVLMPL	5.1298	0.3835	100	2030.119	2029.089	5691.4	1	8.018	73.5	13
	S.VLMPLD	5.6143	0.4959	100	1963.113	1963.109	5021.1	1	8.731	68.8	16
	K.LHEYLV	5.6842	0.4138	100	2227.193	2226.184	6390.4	1	8.083	75	49
	R.SNIGEF	5.1928	0.3791	100	1762.896	1761.893	8865.8	1	8.856	78.6	99
	R.SNIGEF	3.8736	0.4031	100	1882.97	1882.97	9906.3	1	6.912	66.7	6
	K.KNPTVY	4.6701	0.4411	100	2170.031	2168.022	7142.2	1	7.621	58.3	2
	R.SLFDAV	5.7169	0.4537	100	2261.162	2260.157	6685.4	1	8.414	45	45
	R.SLFDAV	6.3472	0.5008	100	2263.168	2260.157	6761.7	1	8.903	62.5	97
	R.LGLVHL	3.2654	0.2427	99.9	883.5705	880.562	6139.9	1	6.015	92.9	6
	K.CNMVSS	4.5802	0.4786	100	1756.908	1756.909	7552	1	8.988	82.1	19
	K.LPGDNY	6.2698	0.4589	100	2510.36	2509.352	8266.3	1	7.919	34.1	16
	K.LPGDNY	6.0311	0.4667	100	2513.372	2509.352	7619.3	1	10.365	63.6	98
	L.PGDNYT	6.3655	0.5379	100	2399.278	2396.267	7651.4	1	10.533	61.9	20
	K.LCAGIN	4.8756	0.5268	100	1938.957	1938.957	6929.2	1	8.919	75	8
	R.VGQLIS	4.416	0.3425	100	1719.92	1719.918	4695.1	1	7.232	64.3	11
	K.TEDEDII	4.3716	0.3506	100	1586.791	1586.788	8823.7	1	6.953	70.8	5
	K.VLLECL	3.0391	0.2881	100	988.551	988.5501	3495.9	1	5.524	92.9	1
	K.SKNDEL	5.4487	0.2875	100	1932.073	1932.073	10167.1	1	6.227	50	27
	K.SKNDEL	5.9854	0.3252	100	1935.082	1932.073	8722	1	8.034	81.2	30
	R.APLENP	5.0613	0.364	100	4559.352	4556.337	8613	1	6.072	22.6	5
	R.VKHDSE	6.2498	0.479	100	3249.588	3246.574	8047.5	1	8.523	33.6	10
	K.FVSPVY	6.1193	0.4939	100	2117.066	2116.061	10511.6	1	8.994	77.8	27

	K.FVSPVY	5.8012	0.416	100	2117.069	2116.061	7983	1	7.698	55.6	21	
	K.LPLLID	5.8509	0.4812	100	1930.073	1930.072	7761.2	1	10.308	80	33	
	K.NRPSVV	5.5938	0.4328	100	2707.347	2706.337	8386.7	1	7.406	37	29	
	K.NRPSVV	5.0356	0.4507	100	2709.35	2706.337	9001.1	1	8.553	52.2	80	
	Q.GVFEFL	4.562	0.4152	100	1926.928	1925.913	9778.6	1	7.733	68.8	21	
	K.KYNGSN	4.9227	0.5092	100	2397.231	2396.225	6128.3	1	9.155	65	9	
	R.ANDSVY	3.3295	0.3802	100	1183.646	1183.644	6277.4	3	6.614	65	2	
	R.TVEFLN	5.9979	0.4456	100	2306.261	2305.255	5678.7	1	8.21	44.7	41	
	R.TVEFLN	5.7054	0.4563	100	2306.262	2305.255	9670.9	1	8.267	57.9	35	
	K.KINSLV	4.2338	0.2987	100	1445.805	1444.801	8100.1	1	6.66	83.3	3	
	K.DCAATC	3.9041	0.4434	100	1338.673	1338.67	6459.8	1	7.575	81.8	12	
	T.SSLPII	5.939	0.4927	100	4250.205	4247.189	10685.2	1	9.118	25.7	5	
	R.DNILND	6.569	0.5713	100	2993.552	2992.549	7550	1	8.911	31.5	65	
	R.DNILND	4.19	0.4229	100	3003.584	2999.566	7990.9	1	7.235	37	104	
	R.DNILND	6.0264	0.4248	100	3004.588	2999.566	6498.3	1	7.06	29.6	15	
	I.LNDAAT	3.9789	0.3754	100	2652.398	2650.395	8631.9	1	7.128	39.6	48	
	L.NDAATA	4.8188	0.4873	100	2538.317	2537.311	8285.9	1	8.999	52.2	73	
	N.DAATAE	3.9774	0.3696	100	2424.276	2423.268	6232.6	1	7.167	47.7	6	
	A.TAEAFL	4.5002	0.4748	100	2167.17	2166.167	8712.1	1	7.977	52.6	12	
	R.QLINNT	3.7363	0.198	99.8	1541.876	1540.87	5906	2	4.81	75	2	
	R.KEELGN	4.3475	0.3059	100	1571.719	1569.711	6482.4	1	6.932	83.3	9	
	K.ISNICN	4.1415	0.3456	100	1564.84	1564.842	7453	2	6.727	65.4	10	
	K.DVYDVL	2.9405	0.2891	99.9	1358.686	1358.684	9365.4	1	5.68	75	6	
	K.QSNQN\	3.0356	0.3578	100	1505.693	1505.692	5083	1	6.379	68.2	3	
	R.SNFDVT	3.8992	0.4149	100	2142.093	2140.086	5872.5	1	6.401	47.2	6	
	K.NFGVTL	4.7241	0.3528	100	1528.939	1526.931	5934.3	1	7.307	84.6	25	
	K.NTLLIF	4.4462	0.3499	100	3453.75	3452.738	7784.4	1	5.835	28.4	4	
PF13_0222		100	29.00%	575	67555	5.4	U	Annotation|Plasmodium_falciparum_Sanger|(protein coc				
	R.YNVDNE	4.4779	0.4683	100	1535.716	1535.716	6483.2	1	9.664	83.3	12	
	K.ILTIFV	4.0347	0.3435	100	1856.012	1856.01	6580.5	1	6.713	68.8	2	
	K.YYESYP	2.8509	0.3492	99.9	1542.703	1542.7	6535.4	1	6.071	63.6	2	
	K.HGDVHI	3.4214	0.3434	100	1061.552	1061.549	7510.1	1	8.797	93.8	18	
	K.YHFQSD	5.4886	0.5303	100	2718.336	2717.332	8218.3	1	9.334	56.8	12	
	K.YHFQSD	7.0829	0.5036	100	2725.353	2724.35	6293.5	1	8.862	45.5	15	
	K.YSALYI	3.0457	0.3018	99.9	1359.682	1358.682	8637	1	5.246	75	1	
	K.DSSIHL	3.6005	0.3075	100	1515.77	1515.767	6008.6	1	5.839	62.5	10	

<table>
<tr><td></td><td>K.DSSIHL</td><td>3.7708</td><td>0.4333</td><td>100</td><td>1515.77</td><td>1515.767</td><td></td><td>5160.8</td><td>1</td><td>6.738</td><td>52.1</td><td>4</td></tr>
<tr><td></td><td>K.MDIQND</td><td>4.9119</td><td>0.4914</td><td>100</td><td>2362.036</td><td>2361.031</td><td></td><td>6574</td><td>1</td><td>8.593</td><td>57.9</td><td>1</td></tr>
<tr><td></td><td>K.ANHHLI</td><td>3.6751</td><td>0.4457</td><td>100</td><td>1359.657</td><td>1359.655</td><td></td><td>5712.6</td><td>1</td><td>7.368</td><td>77.3</td><td>1</td></tr>
<tr><td></td><td>K.ANHHLI</td><td>5.3782</td><td>0.5153</td><td>100</td><td>2792.342</td><td>2791.336</td><td></td><td>7953.7</td><td>1</td><td>9.523</td><td>50</td><td>2</td></tr>
<tr><td></td><td>K.ANHHLI</td><td>5.5987</td><td>0.4681</td><td>100</td><td>2793.346</td><td>2791.336</td><td></td><td>9574.9</td><td>1</td><td>7.627</td><td>37</td><td>6</td></tr>
<tr><td></td><td>K.ANHHLI</td><td>5.4911</td><td>0.2971</td><td>100</td><td>3922.842</td><td>3921.835</td><td></td><td>8622.6</td><td>1</td><td>5.515</td><td>23.4</td><td>2</td></tr>
<tr><td></td><td>K.ANHHLI</td><td>6.7908</td><td>0.5299</td><td>100</td><td>3923.844</td><td>3921.835</td><td></td><td>9768.2</td><td>1</td><td>8.701</td><td>29.7</td><td>6</td></tr>
<tr><td></td><td>K.GKDYYC</td><td>5.1977</td><td>0.5316</td><td>100</td><td>1956.865</td><td>1955.86</td><td></td><td>10680.4</td><td>1</td><td>10.062</td><td>76.7</td><td>6</td></tr>
<tr><td>PF10_0272</td><td></td><td>206</td><td>29.00%</td><td>386</td><td>44221</td><td>10.2</td><td>U</td><td>Annotation|Plasmodium_falciparum_TIGR|(protein codin</td><td></td><td></td><td></td><td></td></tr>
<tr><td></td><td>R.HGSLGF</td><td>3.2058</td><td>0.3269</td><td>100</td><td>983.5408</td><td>983.5427</td><td></td><td>3640.1</td><td>1</td><td>6.409</td><td>81.2</td><td>47</td></tr>
<tr><td></td><td>K.PPHFTA</td><td>2.8195</td><td>0.3543</td><td>99.9</td><td>1295.624</td><td>1295.625</td><td></td><td>4443.7</td><td>1</td><td>6.12</td><td>75</td><td>12</td></tr>
<tr><td></td><td>K.AGMTHI</td><td>2.913</td><td>0.3191</td><td>100</td><td>891.4934</td><td>891.4948</td><td></td><td>4153.8</td><td>1</td><td>5.866</td><td>78.6</td><td>1</td></tr>
<tr><td></td><td>R.ILSTVW</td><td>4.1879</td><td>0.4553</td><td>100</td><td>1783.919</td><td>1780.91</td><td></td><td>6336.4</td><td>1</td><td>8.244</td><td>71.4</td><td>66</td></tr>
<tr><td></td><td>R.ILSTVW</td><td>3.6621</td><td>0.2726</td><td>99.9</td><td>1939.015</td><td>1937.011</td><td></td><td>5936.3</td><td>6</td><td>5.559</td><td>43.3</td><td>1</td></tr>
<tr><td></td><td>K.AHIMEV</td><td>3.0465</td><td>0.2853</td><td>99.8</td><td>1531.819</td><td>1528.78</td><td></td><td>2772.5</td><td>12</td><td>5.263</td><td>50</td><td>1</td></tr>
<tr><td></td><td>K.NLPVST</td><td>4.5489</td><td>0.474</td><td>100</td><td>2332.248</td><td>2331.246</td><td></td><td>7244.3</td><td>1</td><td>7.592</td><td>50</td><td>23</td></tr>
<tr><td></td><td>K.VACIGA</td><td>3.3918</td><td>0.3576</td><td>100</td><td>1245.645</td><td>1244.644</td><td></td><td>6154.7</td><td>1</td><td>6.546</td><td>80</td><td>17</td></tr>
<tr><td></td><td>V.ACIGAW</td><td>2.8438</td><td>0.3185</td><td>99.9</td><td>1139.56</td><td>1138.558</td><td></td><td>5255.1</td><td>1</td><td>5.408</td><td>77.8</td><td>2</td></tr>
<tr><td></td><td>K.KITPMG</td><td>3.3141</td><td>0.3407</td><td>100</td><td>2242.152</td><td>2241.149</td><td></td><td>8321</td><td>1</td><td>5.453</td><td>44.7</td><td>2</td></tr>
<tr><td></td><td>K.ITPMGG</td><td>4.6771</td><td>0.3615</td><td>100</td><td>2347.221</td><td>2347.216</td><td></td><td>6508.5</td><td>1</td><td>7.953</td><td>57.5</td><td>29</td></tr>
<tr><td></td><td>T.PMGGFI</td><td>4.6999</td><td>0.3227</td><td>100</td><td>2133.093</td><td>2133.084</td><td></td><td>4735.1</td><td>1</td><td>6.066</td><td>40.3</td><td>3</td></tr>
<tr><td></td><td>G.GFPHYC</td><td>3.137</td><td>0.364</td><td>99.8</td><td>1847.973</td><td>1847.97</td><td></td><td>4979.4</td><td>2</td><td>6.512</td><td>46.7</td><td>1</td></tr>
<tr><td></td><td>G.GFPHYC</td><td>3.1798</td><td>0.3206</td><td>99.9</td><td>1847.982</td><td>1847.97</td><td></td><td>8670.3</td><td>17</td><td>6.213</td><td>43.3</td><td>1</td></tr>
<tr><td>MAL13P1.413</td><td></td><td>70</td><td>28.80%</td><td>250</td><td>29048</td><td>6.4</td><td>U</td><td>Annotation|Plasmodium_falciparum_Sanger|(protein coc</td><td></td><td></td><td></td><td></td></tr>
<tr><td></td><td>G.TVPQAD</td><td>3.8144</td><td>0.3116</td><td>100</td><td>2301.094</td><td>2297.08</td><td></td><td>4960.1</td><td>1</td><td>6.227</td><td>47.5</td><td>9</td></tr>
<tr><td></td><td>V.PQADVF</td><td>3.5939</td><td>0.3227</td><td>100</td><td>2097.992</td><td>2096.964</td><td></td><td>5621.6</td><td>7</td><td>6.42</td><td>38.9</td><td>3</td></tr>
<tr><td></td><td>V.PTEGMD</td><td>3.1827</td><td>0.4094</td><td>100</td><td>1586.741</td><td>1586.72</td><td></td><td>7219.3</td><td>1</td><td>7.237</td><td>53.8</td><td>4</td></tr>
<tr><td></td><td>R.NWFTN</td><td>4.9132</td><td>0.5129</td><td>100</td><td>2587.056</td><td>2586.048</td><td></td><td>4358.5</td><td>1</td><td>8.738</td><td>38.1</td><td>3</td></tr>
<tr><td></td><td>R.NWFTN</td><td>5.371</td><td>0.4137</td><td>100</td><td>2589.059</td><td>2586.048</td><td></td><td>8341.3</td><td>1</td><td>7.461</td><td>52.4</td><td>39</td></tr>
<tr><td></td><td>K.KLHLEQ</td><td>5.4761</td><td>0.5022</td><td>100</td><td>2222.961</td><td>2221.954</td><td></td><td>7508.8</td><td>1</td><td>8.905</td><td>71.9</td><td>3</td></tr>
<tr><td></td><td>K.KLHLEQ</td><td>3.7887</td><td>0.3291</td><td>100</td><td>2363.015</td><td>2359.013</td><td></td><td>3728.3</td><td>7</td><td>5.349</td><td>30.9</td><td>3</td></tr>
<tr><td></td><td>K.KKPGEF</td><td>2.9683</td><td>0.2653</td><td>99.8</td><td>1346.623</td><td>1346.62</td><td></td><td>5192.9</td><td>1</td><td>5.473</td><td>65</td><td>6</td></tr>
<tr><td>PFF1155w</td><td></td><td>469</td><td>28.60%</td><td>493</td><td>55261</td><td>7.1</td><td>U</td><td>Annotation|Plasmodium_falciparum_Sanger|(protein coc</td><td></td><td></td><td></td><td></td></tr>
<tr><td></td><td>K.LDTIECD</td><td>5.3173</td><td>0.4706</td><td>100</td><td>2126.069</td><td>2124.058</td><td></td><td>4043</td><td>1</td><td>8.125</td><td>65.6</td><td>6</td></tr>
<tr><td></td><td>R.ISYSTL</td><td>5.9551</td><td>0.4091</td><td>100</td><td>2206.103</td><td>2203.092</td><td></td><td>9912.9</td><td>1</td><td>8.607</td><td>70.6</td><td>137</td></tr>
<tr><td></td><td>R.ISYSTL</td><td>2.9021</td><td>0.3506</td><td>99.9</td><td>2331.193</td><td>2331.187</td><td></td><td>5141.7</td><td>2</td><td>5.319</td><td>36.1</td><td>1</td></tr>
</table>

	K.HATASQ	5.2694	0.5283	100	1629.781	1629.777		5583.9	1	8.839	57.7	45
	K.HATASQ	5.1086	0.4889	100	1632.788	1629.777		8442.3	1	9.081	84.6	216
	K.LMNDAF	2.8378	0.2739	99.9	965.4883	965.4879		3072.9	1	5.484	85.7	1
	K.VCCVLN	4.3194	0.4238	100	2189.993	2188.986		6451.3	1	7.72	69.4	6
	K.IINIEF	5.4212	0.4679	100	3383.641	3383.662		7358.1	1	7.944	27.7	16
	K.IINIEF	3.6956	0.3515	100	3383.669	3383.662		9241.8	1	6.275	30.4	10
	K.DLPTSP	4.6815	0.5383	100	2101.024	2100.019		4935.5	1	8.704	64.7	18
	R.FMVNVL	3.1337	0.3473	100	1383.675	1383.677		7109.2	1	7.027	81.8	6
	R.KICEAV	3.3889	0.3415	100	1152.585	1152.584		4266.9	1	5.793	93.8	6
	R.SAALAA	3.443	0.4083	100	1242.763	1242.761		9498.5	1	7.423	76.9	1
PFE1370w		137	28.40%	458	51125	4.8	U		Annotation|Plasmodium_falciparum_Sanger|(protein coc			
	K.QFVTLC	5.4333	0.4212	100	2616.404	2615.402		5570.6	2	7.205	28.6	11
	K.QFVTLC	4.6049	0.4481	100	2616.407	2615.402		7608.8	1	7.148	42.9	44
	Q.ENPSIL	3.4931	0.3216	100	1738.988	1738.986		8208.5	1	6.075	60.7	1
	R.CKDFMV	7.6862	0.5959	100	4099.994	4097.98		5319.6	1	9.84	35.3	26
	K.IEAAEL	3.8127	0.3334	100	1236.655	1236.655		8757.8	1	7.456	90	3
	K.IEAAEL	5.5676	0.3781	100	2086.076	2083.067		9573.9	1	7.964	70.6	25
	K.IIAFGKP	2.5043	0.3417	99.9	1034.571	1034.571		2873.4	1	5.193	77.8	2
	R.ASVLLS	2.3637	0.3324	99.8	830.5356	830.5352		5496.5	1	6.452	92.9	1
	L.GKWECA	4.82	0.3198	100	1757.813	1756.808		6106.4	1	6.436	71.4	5
	K.WECAHA	4.9556	0.4949	100	1578.712	1578.708		8105	1	8.271	83.3	11
	K.IDYDED	4.008	0.4013	100	1585.695	1584.689		6622.8	1	7.101	81.8	5
	K.ELFNNP	3.2276	0.2827	99.9	1284.611	1283.606		4681.4	9	5.34	77.8	3
PF14_0361		92	28.40%	335	39088	7.8	U		Annotation|Plasmodium_falciparum_TIGR|(protein codin			
	K.RAVEYF	2.9486	0.2474	99.9	940.5017	940.5005		4669.8	1	5.687	100	3
	R.GDDFVN	3.5571	0.2999	99.9	1786.835	1786.832		6202.8	1	5.531	56.7	3
	R.NLSDMK	4.4948	0.4939	100	2202.049	2202.065		5427.3	1	8.685	55.9	5
	R.NFATQS	4.3042	0.3857	100	1919.981	1919.98		5564.4	1	6.897	63.3	9
	R.NFATQS	5.0978	0.4386	100	2049.082	2048.075		8223.7	1	7.78	65.6	39
	K.ATFEGN	5.034	0.619	100	2971.182	2970.175		5931.4	1	10.824	45.8	3
	K.ATFEGN	4.0155	0.5392	100	3098.261	3098.27		5691.8	1	9.046	40	6
	K.CGFTSF	3.228	0.423	100	1344.628	1344.626		7040.6	1	8.261	75	24
PF13_0021		17	28.40%	211	25195	6.4	U		Annotation|Plasmodium_falciparum_Sanger|(protein coc			
	R.NEALEN	3.565	0.458	100	2735.158	2735.154		6150.5	1	7.127	47.7	12
	R.LDQYHS	2.964	0.3377	100	1278.549	1278.554		5449.5	1	6.212	83.3	1
	R.RDGYSN	3.9912	0.3638	100	1936.949	1936.944		7824.4	1	6.439	56.2	3

	K.SHEQKD	2.4341	0.3578	99.9	1284.596	1284.593		5175.8	1	6.108	77.8	1	
MAL8P1.40		50	28.00%	286	32441	9.1	U		Annotation|Plasmodium_falciparum_Sanger|(protein coc				
	K.YGTITQ	4.8206	0.499	100	2016.029	2015.024		5061.8	1	8.239	64.7	3	
	R.GWAFIT	3.9674	0.3779	100	1277.631	1277.639		7251.3	1	7.945	83.3	8	
	K.HVTTDG	4.2066	0.4351	100	1947.871	1947.863		7912.6	1	8.715	65.6	6	
	K.HVTTD	3.5154	0.4057	100	2607.172	2606.17		4951.6	1	6.596	42.9	3	
	Y.NSVTGH	3.7236	0.3443	100	1497.754	1497.745		7014	1	5.797	70.8	8	
	K.GYGFVS	4.4566	0.525	100	1927.959	1927.955		6029.1	1	8.376	64.7	12	
	K.GYGFVS	3.2627	0.4855	100	1934.976	1934.973		7337.4	1	8.308	41.2	1	
	K.GMHGFY	2.9313	0.4882	100	1082.516	1082.509		3749.2	1	6.905	66.7	9	
PF13_0272		114	27.90%	208	23988	9.4	U		Annotation|Plasmodium_falciparum_Sanger|(protein coc				
	K.FYAPWC	2.4027	0.3683	99.9	1067.443	1067.441		3660.8	1	6.17	85.7	2	
	K.TWAQLA	3.8435	0.2375	100	1160.635	1160.632		4173.5	1	6.336	88.9	30	
	R.FKIEGF	3.6563	0.2792	100	1609.921	1609.911		7558.5	1	4.973	66.7	1	
	K.IEGFPTL	3.6617	0.3246	100	1327.732	1327.73		4150.1	1	6.675	90	29	
	K.ASEPPK	3.6343	0.2731	99.9	1716.911	1715.904		4694.1	1	4.948	64.3	4	
	K.ASEPPK	3.8572	0.231	99.9	3212.614	3208.599		6378.8	1	5.101	34.6	5	
	K.ASEPPK	4.8239	0.355	100	3223.648	3222.633		8382.2	1	6.362	28.2	5	
	K.ASEPPK	6.2861	0.4309	100	3224.652	3222.633		7834.3	1	7.925	38.5	38	
PFC1025w		6	27.80%	54	6065	9.8	U		Annotation|Plasmodium_falciparum_Sanger|(protein coc				
	K.KGPVEL	3.7232	0.3926	100	1703.924	1703.922		6149.6	1	6.778	60.7	1	
	K.KGPVEL	4.1612	0.3896	100	1710.945	1710.939		8072.2	1	6.946	71.4	5	
PFE0060w		80	27.70%	408	48721	7.2	U		Annotation|Plasmodium_falciparum_Sanger|(protein coc				
	K.NEQDTN	3.8649	0.4449	100	2605.136	2605.13		5124.4	1	7.714	47.6	6	
	K.NEQDTN	4.8579	0.3448	100	2605.14	2605.13		7580.3	1	6.608	36.9	3	
	F.ENYAND	3.1381	0.3406	99.9	3134.468	3134.464		4934.2	2	5.248	28	1	
	K.HEEVHK	5.5204	0.3794	100	2313.119	2313.105		6785.3	1	8.434	58.8	3	
	K.HEEVHK	4.4363	0.4134	100	2313.12	2313.105		8841	1	6.96	45.6	3	
	K.HEEVHK	4.6166	0.3612	100	1499.752	1497.745		4922.4	1	6.721	86.4	35	
	R.TLILGS	2.935	0.3327	99.8	1615.925	1614.922		5787.8	1	5.824	57.1	2	
	K.LLGSIFC	3.3985	0.3254	100	1151.661	1151.658		3616.2	1	6.333	88.9	4	
	K.HLLQQI	3.8417	0.1746	99.9	1266.721	1264.713		4876.2	2	5.659	88.9	23	
PFE0065w		148	27.60%	337	36299	4.4	U		Annotation|Plasmodium_falciparum_Sanger|(protein coc				
	R.AFDFFT	5.7655	0.417	100	2831.324	2828.315		5505.2	1	8.083	47.9	51	
	V.GGAASC	4.6882	0.4191	100	3121.632	3120.614		6567.8	1	7.582	35.3	3	
	G.GAASGI	4.9145	0.5491	100	3064.602	3063.592		8430.8	1	9.998	39.4	4	

	G.GAASGI	4.8142	0.3424	100	3065.606	3063.592		7686.1	1	6.217	21.7	1
	G.GAASGI	6.3019	0.5088	100	3067.614	3063.592		9534.4	1	9.269	35.6	51
	G.DAASGL	5.7529	0.4007	100	2188.124	2187.115		7115.6	1	7.653	43.2	12
	G.DAASGL	6.8471	0.558	100	2190.13	2187.115		7134.8	1	9.863	63.6	18
	G.NLVGD/	3.4528	0.4304	100	1615.852	1615.854		10076.9	1	7.81	66.7	3
	R.MAQEAI	3.5984	0.2764	99.9	2708.305	2707.296		6297.2	1	5.88	37	2
	R.VQFFAD	2.854	0.3138	99.9	1073.553	1072.547		2829.3	1	4.572	81.2	3
PF14_0716		133	27.60%	254	28838	5.7	U		Annotation|Plasmodium_falciparum_TIGR|(protein codin			
	K.SKDYVV	2.5787	0.3252	99.8	1194.669	1194.674		4939.2	1	5.696	75	1
	K.SKDYVV	3.1079	0.427	100	1322.769	1322.768		7966.2	1	6.299	81.8	3
	K.NLHLFE	5.2827	0.4532	100	2400.2	2397.195		4711.9	1	7.481	63.9	20
	K.NLHLFE	5.8275	0.3685	100	2405.221	2404.212		5415.9	1	6.742	38.9	23
	G.KNHPW	4.8274	0.4264	100	2336.209	2336.2		8207.2	1	7.71	50	1
	K.NHPWQ	4.0258	0.3415	100	2203.101	2201.088		6841.6	1	6.265	47.1	2
	K.NHPWQ	5.9923	0.5059	100	2209.109	2208.105		7047.3	1	9.66	76.5	74
	K.VKMDAE	4.5474	0.2883	100	2334.028	2333.02		9175.1	1	6.543	57.9	3
	K.MDAEQI	4.8384	0.3756	100	2106.865	2105.857		9008.4	1	7.461	61.8	6
PF13_0349		35	27.50%	149	17012	8.9	U		Annotation|Plasmodium_falciparum_Sanger|(protein coc			
	K.SFIMIK	3.8782	0.3391	100	1404.793	1404.786		5678.5	1	6.713	72.7	15
	K.GPVVA	3.9343	0.4696	100	1616.806	1616.818		11116	1	8.779	75	6
	K.NVIHGS	4.4916	0.4289	100	1405.716	1405.715		7206.9	1	8.925	84.6	10
	K.NVIHGS	4.2387	0.4046	100	1405.718	1405.715		4327.5	1	8.594	73.1	4
PFB0340c		98	27.30%	997	111768	5.4	U		Annotation|Plasmodium_falciparum_TIGR|(protein codin			
	K.LPSNGT	4.9033	0.5426	100	1748.842	1748.841		4740.4	1	10.355	61.8	6
	R.GDTEPI	5.0958	0.2199	99.9	4934.139	4933.13		8413.9	1	7.46	19.6	1
	K.ETPFTN	4.1677	0.4767	100	1560.863	1560.861		4914.6	1	7.655	70.8	26
	K.LTESIDI	3.4586	0.3811	100	1244.713	1244.71		8199.6	1	6.881	85	4
	K.TNENNE	4.0475	0.3374	100	1412.718	1411.714		4891.1	4	6.338	68.2	1
	R.LLIYHSE	4.0017	0.3803	100	1700.942	1700.941		7611.8	1	7.258	80.8	5
	L.KNVDDV	3.6351	0.3671	100	1386.75	1386.749		7321.9	1	7.125	85	2
	K.NVDDWI	3.1297	0.2379	99.9	1265.672	1265.672		7117.3	4	6.079	66.7	1
	R.GLVLPE	3.8228	0.328	100	2699.307	2698.304		7700.9	1	6.413	45.2	20
	K.ISALYV	3.6567	0.4041	100	1753.88	1752.874		8374.1	1	6.634	57.1	3
	K.DRCDE(	4.5216	0.3938	100	4061.852	4060.845		6168.5	1	5.916	24.2	1
	K.VEDHWI	3.7457	0.3835	100	1644.734	1643.728		9002.8	1	6.781	70.8	3
	K.ILHNKN	4.2285	0.3093	100	1585.844	1585.841		6150	1	6.488	73.1	14

	R.NSWGP	3.619	0.5711	100	1705.732	1705.729	5845		1	8.804	73.1	6
	K.ESNTAL	3.5463	0.4326	100	1880.85	1880.847	3091.6		1	7.679	47.1	3
	R.KYIDTQ	3.596	0.1896	99.8	1351.726	1351.722	3687.7		1	5.981	80	2
PF10_0086		30	27.30%	242	27611	8.9	U	Annotation|Plasmodium_falciparum_TIGR|(protein codin				
	K.SHCYCH	4.1654	0.5929	100	1718.775	1718.774	9330.6		1	10.099	76.9	9
	K.LVDDQN	3.3211	0.336	99.8	1603.83	1603.825	8270.6		1	7.039	57.7	3
	K.LVDDQN	4.4484	0.3519	100	1603.836	1603.825	10195.6		1	7.449	76.9	9
	K.LDGVFY	2.9117	0.3439	99.9	1995.995	1996.018	6604.3		2	5.56	43.8	1
	K.IFNPPK	3.7593	0.3855	100	2509.356	2509.376	5607.6		1	6.375	33.8	1
	K.IFNPPK	3.7294	0.2522	99.9	2509.39	2509.376	7890.8		1	5.821	37.5	1
	K.VPFRDD	4.6479	0.3963	100	1798.949	1798.945	8865.2		1	6.885	67.9	6
PFI0755c		484	27.20%	1418	159451	6.8	U	Annotation|Plasmodium_falciparum_Sanger|(protein coc				
	K.LKEHGH	3.9906	0.3895	100	1960.974	1960.973	6227.8		1	7.657	52.9	3
	K.LKEHGH	4.5923	0.4108	100	1960.976	1960.973	6563.9		1	7.429	67.6	6
	A.SEYQIL	4.2654	0.294	99.9	3097.42	3096.408	5740.3		2	4.947	27.1	2
	K.NLHNLP	3.0635	0.2995	99.9	1292.722	1292.715	5988.9		3	5.116	65	2
	K.IGIILS	5.1494	0.4931	100	2504.416	2504.4	8139		1	8.683	47.9	15
	I.ILSGGP	4.2734	0.4806	100	2207.175	2207.176	7218.5		1	8.204	54.8	4
	I.LSGGPA	3.3733	0.3345	100	2073.041	2073.041	6146.4		1	5.742	47.5	2
	L.SGGPAF	4.5229	0.555	100	1973.994	1973.991	7811.4		1	9.585	57.9	11
	S.GGPAPC	4.8793	0.5265	100	1886.959	1886.959	6506.4		1	9.108	69.4	14
	K.RYNEQS	4.6801	0.4754	100	2359.231	2358.228	7601		1	8.075	60	10
	R.YNEQSC	4.1484	0.4206	100	2202.152	2202.127	5504.3		1	7.596	50	11
	R.QIPISII	2.2743	0.4088	99.9	1192.805	1192.804	4906.6		1	6.837	70	1
	K.TGHNVY	2.8067	0.3092	99.8	1181.619	1181.618	5715.4		1	5.918	77.8	5
	K.NYGVIL	3.7479	0.2476	99.8	2164.167	2161.162	4896.6		1	5.306	61.1	7
	K.SKEVWI	4.3229	0.5118	100	1613.927	1613.924	5300.5		1	8.451	83.3	29
	R.NLEDSY	5.3843	0.4462	100	2137.125	2134.116	5713.1		1	7.937	61.8	24
	R.YLVDLN	4.9056	0.3477	100	1635.92	1634.916	9090.3		1	6.936	88.5	59
	S.PGPIQF	4.0931	0.53	100	1484.783	1484.784	6674.2		1	9.325	80.8	5
	R.QAPGAN	2.9393	0.3321	99.9	1549.765	1549.762	3789.8		1	6.2	65.4	3
	K.YVNQGC	4.9722	0.3713	100	2306.113	2305.11	7396.2		1	6.975	50	3
	K.YVNQGC	4.1118	0.3348	100	2704.304	2704.297	6959.4		19	6.354	26.1	3
	T.GNSPEH	3.4544	0.2728	99.9	1629.755	1629.762	6649.2		1	6.02	69.2	3
	K.KIPTSV	4.2497	0.4482	100	3527.815	3526.847	6625.3		1	6.259	22.7	4
	K.IPTSVV	4.2878	0.3988	100	3400.76	3398.752	5510.7		1	7.442	33.9	31

	I.PLTGSN	3.2028	0.3577	100	2646.301	2646.327		4142	14	5.456	30.4	1
	C.ALQTHF	5.5617	0.5075	100	2171.143	2169.138		5523.1	1	8.76	63.2	7
	K.NYGTVL	3.5035	0.301	99.9	1903.999	1904.01		5180.8	1	5.419	50	7
	K.ILLSEIS	5.9223	0.3794	100	1775.958	1773.949		9547.5	1	7.887	83.3	89
	R.NDLVNL	5.11	0.3924	100	2309.143	2307.137		6287.6	1	9.221	57.5	33
	T.PWSLAL	3.0122	0.2118	99.8	927.5671	927.5668		3563.7	1	5.096	92.9	2
	R.FEQLET	3.4219	0.3243	99.8	1848.98	1848.978		8384.3	2	5.545	57.1	1
	R.FEQLET	5.0379	0.2905	100	1849.984	1848.978		8040.4	1	6.059	85.7	62
	R.FCNPGF	5.3101	0.5279	100	2256.015	2255.005		5033.5	1	9.631	66.7	25
PFD0725c		40	27.20%	379	43317	5.2	U		Annotation|Plasmodium_falciparum_Sanger|(protein coc			
	K.EMFDNI	4.761	0.4259	100	3632.815	3628.804		6514	1	6.72	29.2	3
	K.YSVIVF	4.8289	0.4852	100	1785.953	1783.946		6311.7	1	8.35	66.7	21
	R.LLAFPD	2.7412	0.313	99.9	1157.731	1157.73		4998.1	1	5.998	72.2	3
	K.NEQIQS	3.7762	0.3255	100	1712.87	1712.868		9283.7	1	5.785	69.2	3
	K.NLYSND	4.6979	0.3901	100	1986.972	1985.969		9684.9	1	7.414	60	6
	R.GLNNIS	3.8796	0.4465	100	1768.902	1767.895		6747.4	1	7.332	56.7	4
PF14_0421		62	27.20%	265	30444	9	U		Annotation|Plasmodium_falciparum_TIGR|(protein codin			
	K.VPIGGQ	4.242	0.4437	100	2146.187	2146.189		5166.3	1	7.706	50	3
	V.PIGGQA	5.2032	0.5603	100	2047.122	2047.12		7206.7	1	9.605	66.7	11
	R.SVQGHI	3.1776	0.3656	100	1156.65	1156.648		5410	1	6.363	77.8	13
	R.FAIENN	4.8433	0.3767	100	2204.055	2201.048		5347.9	1	6.704	52.8	19
	K.GTIYFS	4.4004	0.3329	100	2668.291	2667.281		9304.2	1	6.041	33	9
	K.GTIYFS	3.3254	0.3502	100	2668.292	2667.281		5237.1	1	6.543	47.7	4
	F.SFGEPF	3.4241	0.3086	99.9	2086.997	2085.996		4068.1	1	5.8	55.9	3
MAL13P1.209		19	27.10%	188	21617	11	U		Annotation|Plasmodium_falciparum_Sanger|(protein coc			
	R.YRPPLS	2.8803	0.2721	99.8	1060.615	1060.616		6602.8	1	5.467	81.2	5
	K.LQYHMA	4.641	0.4928	100	2551.281	2551.28		7147.4	1	8.171	54.5	12
	M.ANHPNI	3.1373	0.3508	99.9	2036.085	2035.076		6377.1	1	5.416	44.4	1
	K.RIEDAG	3.3303	0.3159	99.9	2043.031	2043.029		5895.2	1	6.655	55.9	1
PF11_0272		134	26.90%	156	17891	10.5	U		Annotation|Plasmodium_falciparum_TIGR|(protein codin			
	R.AGELTT	5.6952	0.4783	100	2575.283	2574.276		7009.2	1	8.022	39.8	27
	R.AGELTT	5.0094	0.4656	100	2595.323	2595.328		9339.6	1	9.106	47.7	76
	K.NIHVIA	4.2434	0.4743	100	1655.885	1655.887		8036.2	1	7.419	73.1	27
	K.NIHVIA	3.9377	0.3063	100	2300.19	2298.184		6369.6	1	5.933	41.7	4
PFC0290w		67	26.90%	145	16130	10.8	U		Annotation|Plasmodium_falciparum_Sanger|(protein coc			
	K.ITAFVP	4.4122	0.5131	100	2956.438	2955.439		4835.3	1	8.273	40.4	37

	V.PGDGCI	5.1311	0.3966	100	2418.12	2417.116	5831.1		1	7.505	54.8	13
	R.SGHSVC	3.2857	0.4533	100	1152.602	1152.601	7258.4		1	8.374	72.7	17
PF14_0341		114	26.80%	579	67368	7.2	U	Annotation|Plasmodium_falciparum_TIGR|(protein codin				
	K.LVEYAE	3.3576	0.3788	100	1449.789	1449.784	8449.8		1	6.775	77.3	3
	K.NVLEDV	3.3919	0.3492	100	1222.682	1222.68	6062.4		1	6.522	85	3
	K.NVLEDV	2.9696	0.3126	99.9	1350.78	1350.775	4652.9		1	5.943	63.6	2
	R.FLANVD	3.2644	0.2916	99.9	1373.684	1373.681	6082.9		1	5.571	77.3	3
	R.DNVFE	4.0807	0.5352	100	1626.727	1626.734	11135.6		1	8.981	83.3	30
	R.NFLNGC	4.0906	0.4519	100	2314.01	2313.018	4834.8		1	6.437	55.6	9
	K.ENIPVL	5.6329	0.4738	100	2525.269	2525.283	10085.1		1	8.715	57.5	40
	I.PVLLAL	3.4811	0.3763	100	2163.103	2162.096	4658.9		1	5.957	55.9	4
	K.NVAILP	3.4281	0.2258	99.8	1541.907	1539.901	4322.3		2	4.135	70.8	1
	Q.LIHQGQ	3.7459	0.4744	100	1791.065	1791.053	7030		1	8.067	63.3	13
	K.EVVSNH	3.4773	0.3358	100	2620.267	2620.272	6443.9		13	6.416	26.1	3
	K.KSDNTY	3.7686	0.4521	100	1547.7	1547.698	6001.6		1	8.636	79.2	3
PFE0050w		84	26.50%	260	30716	9.5	U	Annotation|Plasmodium_falciparum_Sanger|(protein coc				
	R.DLDICN	3.6958	0.3366	100	1888.831	1888.825	7245.5		1	7.737	71.4	1
	R.DLDICN	7.5982	0.5044	100	4646.215	4644.2	7629.7		1	7.464	25	6
	R.DLDICN	5.1209	0.3421	100	5160.44	5159.422	7020.2		12	5.355	16.1	1
	R.DLDICN	6.4432	0.3561	100	5161.45	5159.422	5377		1	6.385	19.8	1
	K.NPCAIP	5.5565	0.4645	100	2619.232	2618.23	6708.9		1	8.863	54.5	49
	K.NPCAIP	4.2925	0.3682	100	2775.401	2774.393	4003		1	6.672	30.4	2
	K.NPCAIP	4.1143	0.3764	100	3290.632	3289.616	3445		2	6.496	26.9	1
	K.DIYKPI	4.415	0.3161	100	1801.934	1801.931	3610.6		1	6.693	75	5
	K.VVDHYV	2.4283	0.3625	99.8	1116.605	1116.605	4583.1		1	6.432	77.8	3
	K.VVDHYV	3.6314	0.2941	100	1272.714	1272.706	6667.1		1	7.02	90	15
PFC0360w		16	26.40%	140	16156	7.5	U	Annotation|Plasmodium_falciparum_Sanger|(protein coc				
	V.PPEVLF	5.0599	0.54	100	1954.994	1954.992	8448		1	9.318	78.1	12
	K.LTHNNI	3.5561	0.4054	100	1208.64	1208.639	4988.1		1	7.468	70	3
	K.LTHNNI	5.1257	0.4658	100	2233.154	2233.144	4664.1		1	7.884	57.9	1
PF14_0391		106	26.30%	217	24805	9.8	U	Annotation|Plasmodium_falciparum_TIGR|(protein codin				
	G.DAVHVE	3.1553	0.2757	99.9	1125.551	1125.554	7000.6		1	6.109	83.3	3
	K.KYDAFL	4.993	0.5167	100	1945.137	1945.135	6330.2		1	8.998	81.2	13
	K.FPSLITH	3.4262	0.2958	100	1179.633	1178.628	8074.5		1	5.321	88.9	9
	K.FPSLITH	3.8094	0.3528	100	1655.883	1655.894	4276.4		1	5.608	48.1	1
	K.FPSLITH	5.3526	0.4312	100	1655.896	1655.894	7024		1	8.46	80.8	19

	K.FPSLITH	3.98	0.3719	100	1655.898	1655.894	5894.5	1	6.828	61.5	1
	C.MGVPV	3.3181	0.3864	100	1122.616	1122.609	4029.2	1	7.038	80	6
	C.MGVPV	4.6634	0.4261	100	1778.914	1778.922	5172.4	1	7.687	80	24
	C.MGVPV	4.2503	0.3565	100	1778.924	1778.922	5910.9	1	6.996	66.7	3
	M.GVPVGI	4.826	0.4225	100	1647.882	1647.882	3610.2	1	7.368	75	12
	G.VPVGH	3.9558	0.3871	100	1590.862	1590.86	4386.7	1	7.499	65.4	4
	G.VPVGH	3.5412	0.3893	100	1590.866	1590.86	5387.3	1	7.356	73.1	3
	V.PVGHAN	4.0515	0.4236	100	1491.793	1491.792	6890.5	1	7.26	75	3
	G.HANLKE	3.2247	0.2445	99.9	1238.644	1238.649	6441.7	1	7.012	88.9	5
PF14_0732		57	26.20%	572	68142	6.6	U	Annotation|Plasmodium_falciparum_TIGR|(protein codin			
	K.HMSDVN	5.9753	0.582	100	2105.959	2105.956	9600.6	1	10.65	71.9	5
	K.HIDSDV	6.4181	0.5969	100	2045.871	2045.869	9322.2	1	11.087	84.4	5
	K.KKEEET	4.3201	0.2395	100	1575.863	1575.859	6633.6	1	6.569	79.2	5
	K.KKEEET	5.7071	0.3229	100	1710.977	1710.971	8077.8	1	6.868	84.6	7
	K.EEETKK	5.7876	0.5012	100	3468.798	3467.791	5089.8	2	7.668	24.1	4
	K.EEETKK	5.1228	0.3437	100	3468.798	3467.791	6658.2	1	5.457	22	1
	K.KEEGIK	4.0051	0.3552	100	1438.789	1438.787	6674.4	1	6.547	81.8	1
	K.KEEGIK	3.3309	0.2789	99.9	1566.889	1566.882	4543	10	5.332	58.3	1
	K.KEEGIK	7.0179	0.4651	100	2852.525	2851.52	9096.3	1	7.734	39.1	4
	K.EEGIKK	4.8662	0.3824	100	2723.424	2723.425	6440.4	1	6.665	37.5	1
	K.EEDVKK	4.6739	0.4041	100	2032.029	2032.027	4006.2	1	7.609	59.4	3
	K.VGIQYD	4.7081	0.5107	100	2105.94	2103.932	9101.4	1	8.116	63.9	1
	K.VGIQYD	4.7507	0.4411	100	2457.177	2457.175	7651.4	1	7.818	45.2	4
	K.EYNANE	3.8323	0.3578	100	1897.922	1896.909	6471.6	1	6.747	57.1	1
	K.KEELLD	5.0326	0.3063	100	1532.744	1532.744	7035.7	1	6.409	88.5	4
	K.VTDVKD	6.6437	0.5124	100	3871.79	3870.78	9923	1	8.546	28	10
PF14_0164		117	26.20%	470	52546	7.6	U	Annotation|Plasmodium_falciparum_TIGR|(protein codin			
	K.LDPNQV	5.6148	0.4359	100	2415.288	2411.273	3695.8	1	7.263	68.4	50
	K.LDPNQV	3.8652	0.3159	99.8	3376.761	3375.734	4845.6	320	5.095	19.4	1
	K.LDPNQV	4.1432	0.399	100	3382.77	3382.751	4292.4	1	7.411	33.3	11
	D.PNQVE	4.5705	0.4204	100	2184.164	2183.161	7225.4	1	6.661	44.1	2
	K.YLPIIET	3.6027	0.2389	99.9	1559.834	1559.832	6885.5	1	5.767	79.2	2
	K.FCQAF	3.1422	0.4276	100	1478.658	1478.656	8454.3	1	7.249	75	3
	R.HIGPCT	4.3776	0.5667	100	1791.899	1791.9	5152	1	9.857	61.8	1
	R.HIGPCT	4.7631	0.4747	100	1791.906	1791.9	6576.1	1	9.624	70.6	27
	R.IKEYLN	3.7899	0.3199	100	1390.726	1390.733	7544.4	1	6.423	77.3	16

	K.AANAGC	3.16	0.3092	99.9	2680.294	2679.274		5789.3	1	5.808	39.6	2
	K.NKYDLC	4.015	0.3384	100	1730.927	1729.935		8216.9	1	6.094	70	2
PF14_0053		218	26.10%	349	40600	5.5	U		Annotation|Plasmodium_falciparum_TIGR|(protein codin			
	K.ESDRFT	3.8843	0.3803	100	2575.274	2574.267		6521.5	1	6.575	44.7	6
	R.FTLYPI	4.7786	0.3325	100	2088.068	2087.064		5422.4	1	7.266	76.7	61
	R.LNLFHA	4.398	0.4313	100	1592.938	1592.935		5272.6	2	7.513	53.8	25
	R.LNLFHA	5.0123	0.4865	100	1592.939	1592.935		7079.1	1	9.033	84.6	93
	K.LHGLTF	2.9107	0.3392	99.9	1493.792	1493.819		4902.7	3	6.073	54.2	1
	R.DEGLHT	4.9236	0.5161	100	2275.082	2274.083		4656.4	1	8.387	58.3	8
	R.DEGLHT	4.6714	0.2539	99.9	3549.747	3546.761		9799.7	1	4.707	22.4	5
	K.LPEQMV	4.0152	0.2282	100	1298.716	1298.714		7483.8	1	5.947	85	2
	K.NPFNWI	4.4659	0.5244	100	1669.849	1669.849		7030	1	8.67	80.8	17
PF14_0104		76	26.00%	465	51245	8	U		Annotation|Plasmodium_falciparum_TIGR|(protein codin			
	K.STLVHA	3.6605	0.4603	100	1485.848	1483.846		8159.8	1	7.532	73.1	13
	Q.NKVELI	4.8691	0.2866	100	1541.884	1541.89		9020	1	6.416	83.3	7
	K.VELIKE	3.646	0.3142	100	1306.771	1306.77		8366	1	5.636	85	3
	R.NFVSGT	5.4516	0.5271	100	2093.196	2092.195		6840.6	1	8.866	57.5	24
	R.SFDVNK	4.5626	0.3589	100	2555.298	2554.298		6560	1	7.33	47.9	2
	R.SFDVNK	4.2658	0.3952	100	2952.567	2951.566		6532.2	1	7.318	35.7	8
	R.SFDVNK	6.445	0.453	100	2952.568	2951.566		6845.4	1	8.432	33	4
	K.IEIRPGI	3.004	0.3018	99.9	1153.769	1153.768		6224.5	1	5.26	66.7	1
	K.GEITCR	3.229	0.3548	100	2676.422	2676.423		4976.2	1	5.627	38.6	1
	R.LVGQVI	3.5135	0.2917	100	1184.722	1184.723		5906.3	1	6.712	90	13
PF11_0352		149	26.00%	423	49195	8.9	U		Annotation|Plasmodium_falciparum_TIGR|(protein codin			
	K.ITEYPT	4.0889	0.3236	100	2173.075	2172.074		5481.3	1	6.236	58.8	6
	K.IKDVVSI	4.4076	0.4577	100	1531.897	1531.904		7532.6	1	8.5	79.2	16
	K.IKDVVSI	3.4299	0.5538	100	1531.908	1531.904		5233	1	7.9	70.8	6
	R.LKELNI	3.0892	0.2806	99.9	1288.751	1288.748		4625.6	21	4.987	60	3
	R.TAQTYC	5.2361	0.48	100	1732.841	1732.852		8247.6	1	10.168	73.1	66
	R.TAQTYC	3.2417	0.3499	99.8	1732.863	1732.852		4961.8	1	6.292	50	2
	K.KPHTAI	3.5868	0.4218	100	1416.69	1416.687		7583.5	1	7.507	68.2	19
	R.TVDDLY	3.4425	0.3513	100	1397.72	1397.711		7221.8	1	6.162	75	18
	K.DVCLLA	4.6254	0.3894	100	2352.187	2352.18		5454.9	1	6.901	50	5
	K.SYVHILS	3.4251	0.3464	100	1286.758	1286.766		6731.6	1	6.372	75	7
	K.SYVHILS	3.0763	0.3755	99.8	1286.774	1286.766		5219.6	1	6.461	65	1
PF14_0046		32	25.90%	297	34725	8.1	U		Annotation|Plasmodium_falciparum_TIGR|(protein codin			

	K.AMDNSN	4.1263	0.3315	100	2535.232	2535.241		6396.7	1	5.864	50	8
	K.FYMYLQ	4.5272	0.3815	100	1540.804	1540.795		10272.2	1	7.386	86.4	9
	K.LNQILIG	3.7804	0.1567	99.9	1156.734	1156.731		7228.4	1	5.719	88.9	10
	K.FIDYTN	6.9863	0.4714	100	3804.874	3804.898		9894.7	1	8.086	29.7	5
MAL8P1.83		78	25.90%	266	31024	6.3	U		Annotation|Plasmodium_falciparum_Sanger|(protein coc			
	K.KKWEDI	6.4075	0.4904	100	2040.005	2040		8729.7	1	9.174	81.2	8
	K.KKWEDI	5.7252	0.4962	100	2041.007	2040		7609.3	1	7.665	51.6	1
	K.KWEDID	6.3056	0.393	100	1907.897	1904.888		9875.6	1	7.889	86.7	5
	K.KWEDID	3.6309	0.4237	100	1911.908	1911.905		3001.7	1	7.389	46.7	1
	K.WEDIDA	4.1806	0.3984	100	1649.703	1648.698		7786.6	1	7.01	88.5	3
	K.WEDIDA	5.3613	0.4248	100	1776.795	1776.793		8690	1	8.424	82.1	7
	K.NNPLDF	4.6629	0.3052	100	1859.874	1859.87		7737.1	1	7.417	67.9	15
	K.NNPLDF	4.4526	0.3654	100	2007.946	2006.939		6220.4	1	6.078	70	29
	K.ILKDEE	5.5162	0.4388	100	1945.958	1945.961		5435.3	1	9.621	71.9	2
	K.ILKDEE	3.8356	0.4266	100	1952.979	1952.978		3863.9	1	7.236	56.2	1
	R.VTNLSE	5.1786	0.525	100	2123.036	2122.031		9110.8	1	9.809	66.7	6
MAL13P1.233		27	25.60%	211	24984	8	U		Annotation|Plasmodium_falciparum_Sanger|(protein coc			
	K.REDNSC	2.9409	0.2449	99.8	1046.563	1046.559		5586.5	17	4.466	75	3
	R.TVEFITT	4.0473	0.3722	100	1435.741	1432.729		4966.1	1	6.346	77.3	7
	L.SKNEDK	4.5809	0.2679	99.9	2491.259	2491.268		7764.8	1	6.137	32.5	2
	K.NEDKID	6.0901	0.3401	100	2291.179	2290.175		5743.3	1	6.704	48.6	3
	K.NEDKID	5.6073	0.3255	100	2291.179	2290.175		6490	1	6.48	58.3	7
	K.IDKNEI	4.5965	0.3209	100	1790.941	1789.934		7313.2	1	5.7	71.4	4
	K.EVNVMT	3.3597	0.3809	100	1407.765	1407.763		6000.9	7	6.436	54.5	1
MAL13P1.337		30	25.60%	172	20036	4.6	U		Annotation|Plasmodium_falciparum_Sanger|(protein coc			
	K.NTASMS	4.7414	0.5176	100	3176.602	3175.594		7472.3	1	8.824	35.7	12
	R.AEFDIV	3.6593	0.3475	100	1326.636	1326.633		6990.3	1	7.267	80	15
	R.AEFDIV	4.1044	0.2906	100	1842.861	1841.856		7489.4	1	6.154	67.9	3
PFI0880c		152	25.50%	396	44604	8.6	U		Annotation|Plasmodium_falciparum_Sanger|(protein coc			
	R.VTFIVS	4.3694	0.4919	100	1693.935	1693.935		5456.7	1	7.339	66.7	10
	H.YFTHFT	3.7234	0.4236	100	1677.881	1676.876		6209.8	1	7.189	67.9	6
	K.IADFII	5.1324	0.5111	100	2229.201	2228.198		7445.6	1	8.555	63.2	23
	R.GSSYLA	4.2877	0.2619	100	1603.912	1600.899		5085.9	1	5.417	76.9	39
	K.KGEVIY	3.3416	0.2752	100	1074.596	1074.595		4040.7	1	5.851	87.5	5
	K.VNALQH	5.0829	0.4007	100	2807.423	2806.42		7112.3	1	8.329	51.9	9
	Q.HFAALP	5.4478	0.5248	100	2282.134	2281.129		6334.8	1	9.157	71.4	60

MAL13P1.307		20	25.50%	137	16568	8.7	U		Annotation|Plasmodium_falciparum_Sanger|(protein coc			
	K.LPLHWE	4.1735	0.4031	100	2492.163	2491.16		9537.9	1	6.868	57.9	4
	R.FEQLEN	5.319	0.3989	100	1692.912	1692.907		10334.9	1	8.258	88.5	12
	R.FEQLEN	3.7061	0.2924	99.9	1807.978	1806.968		11337.5	1	4.674	71.4	4
PF14_0546		119	25.40%	639	75987	5	U		Annotation|Plasmodium_falciparum_TIGR|(protein codin			
	K.KCDLLI	4.6117	0.3708	100	2829.427	2828.418		9255.2	1	7.096	50	3
	K.KCDLLI	7.0331	0.4467	100	2857.493	2856.487		9721.3	1	7.801	45.5	11
	K.CDLLID	4.4609	0.3989	100	2729.398	2728.392		5681.8	1	7.425	45.2	13
	K.IYNDYL\	4.3453	0.2845	100	1525.874	1522.863		6770.9	1	5.286	90.9	12
	K.DDESQM	4.0286	0.2426	99.9	2221.112	2221.103		5169.1	1	6.095	44.4	5
	K.ILLSLDD	4.1381	0.3505	100	2568.372	2567.351		8459.4	1	6.477	57.5	4
	R.FSILDNI	3.8108	0.2807	99.9	1844.005	1843.001		8002.7	1	5.955	64.3	4
	K.LPTAFF	5.0871	0.5702	100	1907.01	1906.008		7022.4	1	10.049	78.1	14
	L.PTAFFD	3.8846	0.3785	100	1793.927	1792.924		7976.4	1	7.386	53.3	2
	M.KHTLHE	4.9903	0.2767	100	1568.768	1565.756		6175.1	1	6.641	79.2	18
	K.HTLHEE	4.1586	0.445	100	1437.664	1437.661		6321.5	1	8.84	86.4	12
	K.SRDFLI	5.8439	0.4758	100	4178.011	4176.004		8343.7	1	8.701	27.3	3
	R.DFLIDF	5.7592	0.3506	100	3914.834	3911.819		6380.3	1	6.237	26.6	12
	K.LVDHLF	2.9453	0.2326	99.9	985.5473	985.5471		5010.5	1	6.067	92.9	6
PF11_0257		207	25.30%	423	49914	5.8	U		Annotation|Plasmodium_falciparum_TIGR|(protein codin			
	K.VEEFMT	4.153	0.3297	100	1954.92	1954.933		8155.3	1	6.978	66.7	6
	K.IYVFFPN	2.8803	0.2945	99.9	1119.607	1119.606		5415.6	2	5.859	81.2	2
	R.ILHDIQL	4.6547	0.3376	100	1715.945	1714.938		10635.9	1	7.227	80.8	4
	K.KLQALQ	3.6636	0.3192	100	1389.797	1389.804		7050	1	6.845	72.7	1
	R.SSFLWS	3.1828	0.2465	99.9	1256.66	1254.652		6006.3	6	4.812	72.2	9
	K.YFNTLK	3.1283	0.2968	100	1202.618	1199.606		7137.1	1	5.729	81.2	1
	K.LIDFDMI	4.6597	0.467	100	2498.278	2498.261		9989.5	1	7.518	39.5	15
	K.LIDFDMI	5.9258	0.4614	100	2501.254	2498.261		9754.2	1	8.166	57.9	133
	K.NSPVVL	5.1474	0.4463	100	2085.091	2083.087		7766.5	1	8.043	61.8	36
PF11_0142		30	25.30%	388	42706	4.7	U		Annotation|Plasmodium_falciparum_TIGR|(protein codin			
	K.KICAEH	5.116	0.4548	100	1778.934	1778.941		8301.2	1	9.182	75	7
	K.KICAEH	3.9311	0.4709	100	1778.942	1778.941		5505	1	7.219	57.1	1
	K.DKESLT	5.7074	0.4778	100	2220.12	2219.113		8039.6	1	8.727	42.1	9
	K.DKESLT	5.1074	0.4701	100	2220.121	2219.113		7679.7	1	7.895	68.4	9
	Q.IENAL	4.005	0.4006	100	3008.374	3008.36		6984.4	4	5.996	25	1
	R.YASQLS	5.9456	0.5061	100	3800.843	3798.825		8393.1	1	8.283	30	3

PF13_0328		97	25.20%	274	30587	4.9	U		Annotation|Plasmodium_falciparum_Sanger|(protein coc				
	K.DLVNDA	3.2316	0.5122	100	1674.785	1674.782		5038.6	1	8.12	66.7	5
	K.DLVNDA	3.3117	0.3589	100	1674.785	1674.782		4885.8	1	6.017	46.7	3
	R.NLSEFS	3.1904	0.2953	99.9	2233.062	2231.054		7894.8	1	6.088	55.6	10
	K.GIVGDA	3.2557	0.3853	100	1331.775	1331.776		9390.2	1	6.908	83.3	3
	K.SNILAD	5.3568	0.5014	100	2289.299	2286.29		7622.4	1	8.999	62.5	76
PF13_0171		32	25.20%	139	14993	9.9	U		Annotation|Plasmodium_falciparum_Sanger|(protein coc				
	R.ITLSLP	5.0218	0.453	100	2106.08	2103.076		5304.5	1	8.38	60.5	26
	R.LPAASL	3.8098	0.5527	100	1485.836	1485.835		8108.4	1	8.595	71.4	6
PF13_0122		33	25.20%	123	14438	5	U		Annotation|Plasmodium_falciparum_Sanger|(protein coc				
	K.SLYDIF	4.8791	0.4703	100	1533.783	1533.778		6992.9	1	9.05	79.2	32
	K.KADHYK	4.9741	0.2991	100	2316.13	2315.124		9270.4	1	6.561	38.2	1
PF07_0087		23	25.00%	244	29250	8.6	U		Annotation|Plasmodium_falciparum_Sanger|(protein coc				
	L.KQVFYV	4.223	0.4487	100	1845.925	1845.921		5622.9	1	7.903	67.9	3
	K.QVFYVF	4.1099	0.2496	99.9	1718.835	1717.826		3919.5	1	6.545	73.1	9
	R.EISLLE	4.0906	0.355	100	2528.202	2528.194		5263.1	2	6.207	38.1	4
	K.KNVSNF	3.9734	0.2865	100	1342.745	1342.744		5674.4	1	5.885	85	2
	K.NVSNFE	2.9517	0.2698	99.9	1214.646	1214.649		5116.8	1	5.275	77.8	1
	K.DISEGN	3.7354	0.3923	100	1553.764	1553.76		5854.2	1	6.801	73.1	4
PFL1180w		94	24.90%	273	31547	4.5	U		Annotation|Plasmodium_falciparum_Sanger|(protein coc				
	K.VIVNNP	5.412	0.4542	100	2949.594	2948.586		5909.2	1	8.341	45.8	47
	K.VIVNNP	6.3476	0.4304	100	2952.604	2948.586		8606.8	1	8.173	37.5	28
	R.GVMMF	3.0798	0.2972	99.8	1746.79	1746.787		6869.7	1	5.559	67.9	3
	K.NMDIDS	3.1171	0.3033	99.9	2200.196	2199.2		4954.9	2	5.203	42.1	1
	R.HIFVENI	2.6629	0.3365	99.9	1012.54	1011.538		2701.3	1	5.536	78.6	15
PFB0635w		57	24.80%	524	58922	7.4	U		Annotation|Plasmodium_falciparum_TIGR|(protein codin				
	K.NISNNY	5.0478	0.372	100	1767.972	1764.965		10186.8	1	7.296	75	37
	K.GTVLLH	3.6474	0.4171	100	1814.957	1814.965		7747.3	1	6.462	66.7	2
	K.ANVDVI	4.4953	0.3556	100	3222.673	3221.679		8786.4	1	5.626	27.7	1
	K.KVTIINS	2.4783	0.3062	99.8	916.6022	916.6018		3806.7	1	5.713	85.7	1
	R.GATFNL	3.3987	0.2789	99.9	1363.688	1363.686		8331.7	1	5.651	72.7	3
	R.NAGYNT	5.129	0.501	100	2166.052	2166.054		5930.5	1	9.058	61.1	2
	K.GNTHSC	3.3941	0.3123	100	1271.634	1271.635		5590.2	1	5.585	70	4
	K.DSHITS	6.0714	0.5412	100	2308.019	2308.024		6241.2	1	9.946	66.7	6
	K.DSHITS	4.5409	0.4484	100	2436.126	2436.119		5249.5	1	7.998	57.9	1
PF14_0230		35	24.80%	294	33998	9.8	U		Annotation|Plasmodium_falciparum_TIGR|(protein codin				

	K.TNSQVI	5.5994	0.5587	100	2065.01	2065.011		8085.8	1	9.958	67.6	9
	K.VTGEIE	4.7917	0.3603	100	2429.183	2429.18		8409	1	6.276	36.2	2
	K.VTGEIE	5.1522	0.4502	100	2444.221	2443.214		5886.4	1	7.3	45	10
	K.GACDG(	4.0051	0.3284	100	1551.703	1551.709		4265.2	1	6.679	82.1	7
	K.TLQEED	3.5421	0.2668	99.9	1384.648	1382.644		5770.2	1	5.296	75	5
	K.THFNDY	2.5201	0.3133	99.9	1037.505	1037.506		4177.2	1	5.748	85.7	2
PFE0850c		17	24.80%	165	18112	9.5	U		Annotation|Plasmodium_falciparum_Sanger|(protein coc			
	K.LGPLGL	2.5279	0.3364	99.9	881.5455	881.546		2994.8	5	7.043	81.2	2
	K.VEVVPT	3.167	0.3574	100	1346.841	1346.837		3589.1	1	6.816	75	1
	K.KPTTIQ	4.7593	0.513	100	2136.109	2136.105		3230.4	1	8.069	52.8	14
PF14_0411		58	24.80%	101	11370	5	U		Annotation|Plasmodium_falciparum_TIGR|(protein codin			
	K.LVGFDE	4.9576	0.5343	100	2926.397	2925.387		7306.1	1	9.269	50	33
	K.LVGFDE	5.2179	0.4161	100	2927.4	2925.387		8795.7	1	8.658	33.3	25
PFC0581w		24	24.40%	324	38640	8.4	U		Annotation|Plasmodium_falciparum_Sanger|(protein coc			
	K.CWFNIT	3.0067	0.325	99.9	1889.924	1888.916		7764.6	1	5.675	75	2
	K.VKDEEI	3.8957	0.384	100	1181.686	1181.685		4887.2	1	6.863	83.3	2
	K.YIVDLTL	3.7715	0.3601	100	1980.049	1979.059		8380.1	1	6.015	68.8	2
	R.YHDSV(	3.1127	0.3123	99.9	1250.616	1250.617		4780.1	1	5.413	88.9	12
	R.YHDSV(	3.3964	0.2482	99.9	1637.8	1636.797		7946.3	1	5.173	75	1
	K.NNSDKL	5.8631	0.4386	100	2987.371	2986.363		8808.9	1	8.386	35.9	1
	K.LQDDID	4.2848	0.4051	100	2415.097	2414.089		9159.6	1	7.073	58.3	4
MAL8P1.103		43	24.30%	548	64411	6.4	U		Annotation|Plasmodium_falciparum_Sanger|(protein coc			
	K.TLCSIL	4.3974	0.4056	100	2196.124	2194.118		7395.3	1	6.775	60.5	6
	R.KEDIYD	5.8709	0.5298	100	2057.054	2056.05		9458.2	1	8.896	78.1	11
	R.CLYSLC	3.9963	0.4572	100	1693.782	1693.779		8396.6	1	7.68	70.8	1
	K.MFCQP(	4.2721	0.4523	100	1874.889	1874.886		4823	1	8.181	73.1	1
	R.LIDTGQ	2.4031	0.3573	99.8	1093.607	1093.608		5132.9	3	5.431	88.9	1
	R.MQSGH	3.5418	0.3796	100	1315.662	1315.662		6350.6	1	6.82	90.9	3
	R.ILNPIV	4.4769	0.4823	100	2711.44	2710.435		8743.8	1	8.52	54.5	4
	K.NYINTE	4.495	0.3758	100	1641.808	1641.81		8331.5	1	6.755	69.2	10
	R.LTSAWN	2.6331	0.3747	99.9	1252.579	1252.579		6144.1	1	5.646	72.2	6
PFE1350c		12	24.30%	152	17411	6.9	U		Annotation|Plasmodium_falciparum_Sanger|(protein coc			
	R.HFNILI	5.5817	0.4679	100	2208.096	2207.096		6873.8	1	8.743	65.8	9
	K.VAEHFK	5.3459	0.5071	100	1993.995	1993.984		8538	1	9.136	71.9	3
PFE0690c		7	24.20%	207	23859	7.4	U		Annotation|Plasmodium_falciparum_Sanger|(protein coc			
	R.NSFLHI	4.2299	0.3693	100	1888.92	1888.92		4509.2	1	6.602	67.9	5

	K.ADCKDI	4.0182	0.4354	100	1981.905	1981.903		4652.3	1	6.782	62.5	1
	K.DATNVE	4.3752	0.3596	100	2051.081	2051.092		9226.7	1	6.25	55.9	1
PFD0310w		4	24.20%	157	16626	6.2	U		Annotation|Plasmodium_falciparum_Sanger|(protein coc			
	K.MPLETQ	3.7364	0.2452	99.9	1514.816	1514.814		9620.1	1	5.121	75	2
	K.NADDD	3.615	0.3545	99.9	2714.06	2713.047		10631.6	6	5.925	25	2
PF13_0011		32	24.00%	217	26005	6.9	U		Annotation|Plasmodium_falciparum_Sanger|(protein coc			
	K.FGNIYD	4.5301	0.392	100	2453.125	2453.139		5870.1	1	6.83	39.5	10
	K.FGNIYD	5.2551	0.4777	100	2453.156	2453.139		9702.3	1	9.403	55.3	13
	R.ISQFLID	3.3006	0.3353	100	1781.046	1781.047		5819.9	1	5.793	53.6	1
	R.ISQFLI	5.758	0.4029	100	3638.011	3637.024		9707.7	1	7.35	25.8	4
	R.ISQFLI	3.7594	0.4952	100	3638.04	3637.024		7867.3	1	8.333	30.6	4
PF13_0197		102	23.90%	351	41276	4.8	U		Annotation|Plasmodium_falciparum_Sanger|(protein coc			
	K.AQSET	5.1987	0.4162	100	3832.819	3830.803		8322.9	1	6.96	22.8	3
	Q.SETDT	5.3387	0.3772	100	3631.71	3631.707		7156.8	1	6.523	23.4	3
	K.NEQEI	4.8926	0.4357	100	2748.31	2748.308		6457.9	1	8.435	41.7	11
	Q.KPAQG(	2.8683	0.3383	99.9	1277.672	1277.649		6369.4	21	5.329	63.6	1
	K.KLYNLG	4.6811	0.44	100	2075.082	2075.093		7725.6	1	7.814	73.5	12
	K.KLYNLG	4.9211	0.4062	100	2082.123	2082.11		8163.1	1	7.226	45.6	5
	K.LYNLGD	4.8556	0.4799	100	1954.017	1954.015		7262.5	1	8.706	75	9
	K.LYNLGD	3.2696	0.3942	100	2075.079	2075.093		4478.1	1	5.637	35.3	2
	K.LYNLGD	4.8261	0.344	100	2075.106	2075.093		7797.9	1	6.949	58.8	33
	G.DVFNH\	4.2941	0.232	100	1522.818	1521.814		6320	1	5.426	83.3	8
	N.HVVDIS	3.1467	0.343	100	1046.61	1046.607		4806.8	1	6.526	75	5
	K.NLIIMF	3.6635	0.3601	100	1435.738	1435.744		8197.4	1	6.506	77.3	5
	K.HMTEIFI	2.7599	0.2774	99.9	1032.575	1032.574		4144.8	1	6.157	92.9	3
	K.KIKPEE	3.3222	0.1922	99.8	1298.743	1298.743		6596.1	2	5.155	77.8	2
PF14_0344		107	23.80%	993	112409	4.4	U		Annotation|Plasmodium_falciparum_TIGR|(protein codin			
	K.MKPTD	4.2704	0.4182	100	2696.236	2695.231		7441.4	1	8.01	43.8	9
	K.GDANN	4.9754	0.4733	100	3841.6	3840.592		9749.1	1	7.258	28.7	1
	K.NFGGAI	4.1432	0.3637	100	1631.843	1631.839		8024.5	1	7.048	64.3	2
	K.KYVTID	5.5994	0.4741	100	4099.546	4097.519		11563.2	1	8.507	22.1	4
	K.LIEEGI	6.1765	0.5044	100	2166.073	2164.066		8704.7	1	9.404	73.5	20
	K.LIEEGI	4.7827	0.5183	100	2307.2	2306.195		7202.7	1	8.692	58.3	14
	R.KLAKPD	5.6068	0.3388	100	2331.127	2330.119		7049.1	1	6.738	46.1	3
	R.KLAKPD	5.3872	0.3958	100	2331.127	2330.119		9511.3	1	7.797	60.5	3
	R.KLAKPD	5.2845	0.3077	100	4073.994	4072.988		7609.8	1	5.23	22.4	3

	R.KLAKPD	6.017	0.453	100	4075.002	4072.988		11222.3	1	8.226	28.6	3
	K.LAKPDD	5.6153	0.4352	100	2203.03	2202.024		7754.3	1	8.865	66.7	9
	K.LAKPDD	5.9928	0.4897	100	3946.908	3944.893		6528.7	1	7.982	27.9	9
	K.DLSLNE	5.0456	0.4587	100	1761.89	1761.887		6919.1	1	7.83	83.3	3
	K.KEDQQ	5.249	0.5382	100	1968.81	1965.799		8670.6	1	9.083	80	9
	K.KNSEYD	5.063	0.4937	100	2252.971	2251.963		5981.4	1	7.818	55.6	3
	K.KKEEME	4.9882	0.3555	100	1918.892	1917.89		7084.2	1	6.522	71.4	6
	K.PSAHVT	3.2699	0.4046	100	1203.575	1203.576		5550.1	1	7.128	85	3
	R.KENPFV	3.5507	0.2679	99.9	1275.68	1274.674		6492.7	1	5.244	85	3
PF14_0329		35	23.80%	252	28872	5	U		Annotation|Plasmodium_falciparum_TIGR|(protein codin			
	K.SNYEAF	3.572	0.2792	100	1287.657	1285.65		6418.3	1	5.948	75	2
	K.NSVSAY	5.7961	0.4711	100	5200.335	5197.313		7412.5	1	7.575	21.7	11
	K.NSVSAY	6.5767	0.5323	100	5613.531	5610.515		11567.3	1	9.388	17.2	12
	K.NSVSAY	5.4	0.3741	100	5614.526	5610.515		7386.5	1	5.817	17.7	7
	A.NLGVDS	4.6751	0.5102	100	2200.972	2200.993		5371.4	1	8.7	66.7	3
PF14_0018		90	23.70%	493	57783	7.3	U		Annotation|Plasmodium_falciparum_TIGR|(protein codin			
	K.KKYNVD	3.939	0.3733	100	1458.724	1458.723		4532.2	1	7.25	86.4	9
	K.SINTTF	4.8538	0.3264	100	1960.031	1959.024		8623.9	1	7.911	76.7	15
	R.HNMLQ/	3.5164	0.2562	99.9	1249.598	1246.589		6620	1	5.455	77.8	12
	S.TKLDLD	3.993	0.3976	100	1602.85	1602.849		8679.7	1	7.437	83.3	2
	K.LDLDYV	3.7066	0.3122	100	1376.719	1373.707		6929.4	1	5.708	90	11
	K.LDLDYV	3.2315	0.3039	99.9	1501.802	1501.802		7010.5	1	5.437	86.4	6
	K.TSDYID	5.804	0.3308	100	7160.079	7156.07		7289	1	5.176	13.7	8
	K.KEEEEE	5.2291	0.3899	100	3830.648	3829.638		6801.4	1	6.469	21.1	6
	K.KEEEEE	7.0415	0.4583	100	3831.647	3829.638		10311.4	1	9.213	31.6	6
	K.EEEEEE	4.8527	0.4468	100	2550.079	2550.074		8316.7	1	9.14	50	3
	K.EEEEEE	6.3296	0.549	100	3702.552	3701.543		9864.3	1	9.419	28	6
	K.GVDVAE	5.4457	0.5059	100	2413.071	2412.065		9455.4	1	9.861	58.7	6
PF10_0115		341	23.60%	1139	131625	9.2	U		Annotation|Plasmodium_falciparum_TIGR|(protein codin			
	R.KATTSS	3.6903	0.3288	100	1644.906	1644.917		6829.1	2	5.337	44.6	2
	R.KATTSS	3.6462	0.3569	100	1644.92	1644.917		5177.6	1	6.653	64.3	2
	K.KKEENE	3.9068	0.1579	99.8	1502.78	1502.782		4726.2	2	4.452	68.2	2
	N.HKNNLE	3.7719	0.3169	100	1504.771	1504.766		6060	1	6.219	86.4	10
	K.KIEIENF	5.2651	0.4091	100	2000.157	2000.152		8634.2	1	8.141	59.4	15
	K.IEIENFI	4.6623	0.4787	100	1836.962	1836.971		8431.8	1	7.085	73.3	18
	R.FNGQTT	3.7193	0.3417	100	1413.73	1413.739		5723.7	1	6.647	72.7	5

	K.NLEEIE	5.8519	0.5017	100	2778.389	2777.403	8660.7	1	9.194	54.5	66
	K.NLEEIE	6.3028	0.5013	100	2781.44	2777.403	9908.1	1	8.153	44.3	3
	K.TNLHIA	2.9598	0.3334	100	1020.54	1020.537	5799.9	1	5.623	87.5	1
	K.TNLHIA	3.4959	0.3791	100	2366.137	2365.15	6562.5	1	6.58	36.8	19
	K.TNLHIA	6.4174	0.4986	100	3432.664	3432.679	10191	1	8.212	33	13
	K.TNLHIA	4.9418	0.5737	100	3433.695	3432.679	7645.1	1	10.138	41.1	8
	K.DDIEQF	4.5363	0.4344	100	2432.163	2431.16	5378.3	1	7.227	55.3	32
	K.NSDIEE	4.9539	0.358	100	1888.982	1887.974	10508	1	7.732	60	14
	K.NLDSEF	4.4216	0.3565	100	1456.697	1453.685	4868	1	6.939	86.4	9
	K.TSHVAQ	3.6517	0.3248	99.9	2034.025	2033.021	6426.8	1	6.269	44.1	3
	K.TSHVAQ	5.6951	0.5677	100	2050.064	2047.055	7991	1	9.517	70.6	69
	H.VAQIYG	3.8262	0.4823	100	1707.892	1707.882	5000.9	1	7.686	64.3	2
	K.DVLENL	4.2528	0.3814	100	1330.683	1330.685	7616	1	6.859	86.4	9
	R.SKEEM	4.988	0.5231	100	3203.318	3203.307	5031.6	1	7.308	27.7	3
	K.AFPSLH	3.9955	0.3525	100	1574.764	1574.764	4622.8	1	7.413	73.1	21
	K.AFPSLH	3.9285	0.4123	100	1574.765	1574.764	5411.5	1	8.319	61.5	4
	K.KHEQAF	2.9885	0.2821	99.9	1193.63	1193.628	4180.7	1	5.76	77.8	2
	K.NEVHAE	3.1897	0.3242	100	1242.598	1242.597	7771.8	1	7.503	75	5
	K.NEVHAE	6.0736	0.5798	100	2871.329	2871.325	7903.3	1	9.238	39.1	3
	K.DKQEEN	3.7681	0.2395	99.9	1733.803	1732.799	5198.8	1	4.773	76.9	1
PFE1050w		255	23.60%	479	53839	5.9	U	Annotation|Plasmodium_falciparum_Sanger|(protein coc			
	K.VKDISL	3.6388	0.4168	100	1181.703	1181.701	5319.3	1	6.784	80	11
	R.IREEYG	3.4579	0.2145	99.8	1482.809	1482.803	7025.2	3	5.286	77.3	2
	K.IIGVSEE	3.8846	0.3048	100	1474.803	1474.812	7520.6	1	7.71	73.1	7
	R.ATDFLIS	3.3008	0.3651	100	951.5161	951.5151	4715.7	1	8.168	87.5	2
	R.VYITEI	5.3045	0.5455	100	3432.832	3429.803	9663.3	1	9.556	36.2	89
	R.VYITEI	6.3623	0.4582	100	3433.854	3429.803	7468.5	1	8.105	41.4	2
	K.NNAVVC	5.4891	0.4864	100	2764.363	2763.356	5133.9	1	9.83	47.8	39
	K.GIHIEN	4.5584	0.4097	100	1504.814	1504.824	7359	1	7.924	83.3	96
	K.GIHIEN	3.3572	0.314	99.9	1504.826	1504.824	5009.9	1	5.824	45.8	2
	K.GIHIEN	3.2137	0.3524	99.8	1518.859	1518.858	5113.3	1	5.825	58.3	1
	G.IHIENVK	3.4398	0.2585	99.9	1461.837	1461.836	6702.7	1	5.687	72.7	1
	I.HIENVKP	3.1416	0.354	100	1334.717	1334.718	6628.6	1	5.724	80	3
PF14_0439		181	23.50%	605	67821	8.6	U	Annotation|Plasmodium_falciparum_TIGR|(protein codin			
	K.GGCNVI	5.3859	0.5047	100	2025.008	2025.019	11120.1	1	9.299	61.1	16
	K.GGCNVI	3.8647	0.404	100	2652.402	2649.342	5948	1	6.005	35.4	1

	K.VNDKNV	5.0673	0.3874	100	2038.981	2038.976	8669	1	8.238	68.8	3
	K.NVSEFL	3.601	0.4121	100	1582.747	1582.742	4307	1	7.408	62.5	3
	K.NVSEFL	4.2519	0.4524	100	1582.747	1582.742	8424.8	1	8.187	79.2	6
	K.HFYMFN	2.8554	0.4551	100	1215.528	1215.526	5525.4	1	7.791	87.5	10
	R.VVLSLV	3.3858	0.3084	99.9	1468.81	1468.82	11433.1	1	6.003	70.8	3
	K.HLGVYI	3.3509	0.3777	100	1515.774	1514.772	6546.7	1	6.336	70.8	2
	K.NSYRPG	4.7048	0.4681	100	1606.838	1606.838	7146.9	1	8.239	64.3	18
	K.NSYRPG	3.4599	0.4541	100	1608.847	1606.838	3913.1	1	6.772	57.1	1
	R.LTLADA	4.0024	0.3686	100	1306.732	1306.726	6526.6	1	8.088	86.4	33
	K.TSNEPV	5.125	0.5112	100	2032.057	2031.053	6189.8	1	8.399	73.3	83
	K.ASSIVAS	2.5113	0.3937	99.9	1142.683	1142.69	6690.7	1	6.595	70	1
	R.LLTEFVI	3.001	0.3847	100	1247.69	1247.689	6533.9	1	6.604	80	1
PF10_0081		99	23.50%	455	51060	8.2	U	Annotation|Plasmodium_falciparum_TIGR|(protein codin			
	R.IKDYLL	5.7309	0.3536	100	2383.248	2382.244	7553.7	1	7.104	69.4	22
	K.TNSVVG	5.9666	0.478	100	2135.177	2135.175	9455.7	1	9.318	73.7	47
	K.APLESY	5.0941	0.4723	100	2182.157	2182.154	7409.4	1	8.452	57.9	7
	K.GVILYG	3.068	0.3519	100	1165.672	1165.669	4949.9	1	5.33	77.3	1
	K.VAEEHA	4.0882	0.3992	100	2240.163	2240.145	5406	1	7.969	55	18
	K.VAEEHA	4.2244	0.2907	99.9	2261.183	2261.197	9375.8	1	5.891	36.2	1
	R.TMLELL	3.4432	0.2842	99.9	1751.873	1751.864	7445.3	1	5.953	71.4	3
PF11_0281		66	23.50%	247	29353	6.4	U	Annotation|Plasmodium_falciparum_TIGR|(protein codin			
	R.LHIVDV	5.8881	0.4256	100	1944.046	1941.038	10107.5	1	8.159	83.3	43
	K.EGCSYT	3.2248	0.4429	100	1618.629	1618.627	5422.6	1	7.784	54.2	3
	K.EGCSYT	3.7197	0.2584	99.9	1746.715	1746.722	4161.7	1	7.494	57.7	6
	K.EGCSYT	2.8449	0.4032	99.8	1746.725	1746.722	4347	1	7.124	50	2
	K.NNIFNH	5.9414	0.4052	100	3131.528	3130.521	6995.1	1	7.187	34	1
	K.NNIFNH	7.3628	0.4183	100	3337.621	3336.614	3915.8	1	7.398	38.9	4
	K.NNIFNH	6.0737	0.465	100	3337.623	3336.614	4232.9	1	6.448	30.9	7
PFF0865w		75	23.50%	136	15441	11.2	U	Annotation|Plasmodium_falciparum_Sanger|(protein coc			
	R.FQSQAV	6.0988	0.4575	100	3520.778	3520.779	8191.7	1	8.32	27.4	19
	R.FQSQAV	3.2448	0.3587	100	3520.781	3520.779	6628.3	1	5.87	25.8	2
	R.FQSQAV	6.6079	0.3319	100	3529.812	3527.796	8740.8	1	6.262	26.9	12
	Q.SQAVLA	5.1244	0.3575	100	3247.666	3245.652	7030.5	1	6.464	28.4	6
	V.LALQEA	3.7216	0.2583	99.8	2869.481	2867.473	7087.1	1	5.065	40	3
	L.ALQEAA	4.7064	0.3588	100	2748.379	2747.371	4922.3	1	6.518	41.7	19
	A.LQEAAE	3.9012	0.3183	100	2684.358	2683.352	6805.8	1	6.518	41.3	6

<table>
<tr><th></th><th></th><th></th><th></th><th></th><th></th><th></th><th></th><th></th><th></th><th></th><th></th><th></th></tr>
<tr><td></td><td>L.QEAAEA</td><td>3.2973</td><td>0.3234</td><td>99.9</td><td>2571.272</td><td>2570.267</td><td></td><td>7338.2</td><td>1</td><td>6.126</td><td>36.4</td><td>8</td></tr>
<tr><td>PFF0530w</td><td></td><td>75</td><td>23.40%</td><td>672</td><td>75815</td><td>7</td><td>U</td><td></td><td>Annotation|Plasmodium_falciparum_Sanger|(protein coc</td><td></td><td></td><td></td></tr>
<tr><td></td><td>K.SGHQG</td><td>3.2176</td><td>0.3818</td><td>100</td><td>1486.751</td><td>1486.741</td><td></td><td>6508.2</td><td>1</td><td>7.172</td><td>64.3</td><td>1</td></tr>
<tr><td></td><td>K.KFEALN</td><td>3.0226</td><td>0.2763</td><td>99.9</td><td>1291.68</td><td>1291.68</td><td></td><td>8077.1</td><td>1</td><td>5.243</td><td>88.9</td><td>1</td></tr>
<tr><td></td><td>K.ILHEIEQ</td><td>3.3809</td><td>0.3192</td><td>100</td><td>1194.678</td><td>1194.685</td><td></td><td>4515.7</td><td>1</td><td>5.486</td><td>77.8</td><td>9</td></tr>
<tr><td></td><td>K.NLQQPT</td><td>2.8121</td><td>0.308</td><td>99.9</td><td>1294.79</td><td>1294.785</td><td></td><td>6143.9</td><td>1</td><td>5.909</td><td>75</td><td>2</td></tr>
<tr><td></td><td>K.SFFGLD</td><td>2.9644</td><td>0.3442</td><td>100</td><td>1166.626</td><td>1166.621</td><td></td><td>6149.5</td><td>1</td><td>5.957</td><td>72.2</td><td>3</td></tr>
<tr><td></td><td>K.YPQVSC</td><td>2.8604</td><td>0.298</td><td>99.9</td><td>1246.701</td><td>1246.698</td><td></td><td>6405.3</td><td>2</td><td>5.893</td><td>77.8</td><td>1</td></tr>
<tr><td></td><td>R.RFQNDL</td><td>3.7049</td><td>0.2075</td><td>99.9</td><td>1431.704</td><td>1431.713</td><td></td><td>4963.4</td><td>1</td><td>5.493</td><td>80</td><td>3</td></tr>
<tr><td></td><td>R.NLSGIVI</td><td>3.1569</td><td>0.3579</td><td>99.8</td><td>1287.775</td><td>1285.767</td><td></td><td>3859.6</td><td>1</td><td>6.797</td><td>68.2</td><td>1</td></tr>
<tr><td></td><td>R.PADGNE</td><td>5.4169</td><td>0.4639</td><td>100</td><td>3010.434</td><td>3010.448</td><td></td><td>8302</td><td>1</td><td>7.624</td><td>37</td><td>6</td></tr>
<tr><td></td><td>R.PADGNE</td><td>4.7893</td><td>0.4684</td><td>100</td><td>3011.475</td><td>3010.448</td><td></td><td>7730.9</td><td>1</td><td>8.459</td><td>35.2</td><td>10</td></tr>
<tr><td></td><td>K.VPHLNN</td><td>3.466</td><td>0.3211</td><td>99.8</td><td>1616.876</td><td>1616.876</td><td></td><td>4906.1</td><td>1</td><td>6.197</td><td>53.8</td><td>1</td></tr>
<tr><td></td><td>K.VPHLNN</td><td>3.9859</td><td>0.4288</td><td>100</td><td>1616.879</td><td>1616.876</td><td></td><td>7100.5</td><td>1</td><td>7.092</td><td>76.9</td><td>18</td></tr>
<tr><td></td><td>R.IVSFPS</td><td>3.3189</td><td>0.3709</td><td>100</td><td>1331.741</td><td>1331.748</td><td></td><td>5047.3</td><td>1</td><td>6.062</td><td>85</td><td>2</td></tr>
<tr><td></td><td>K.IWEHLG</td><td>5.1826</td><td>0.4179</td><td>100</td><td>1827.986</td><td>1824.984</td><td></td><td>6318.6</td><td>1</td><td>7.658</td><td>71.4</td><td>17</td></tr>
<tr><td>PFB0385w</td><td></td><td>45</td><td>23.40%</td><td>137</td><td>15809</td><td>8.8</td><td>U</td><td></td><td>Annotation|Plasmodium_falciparum_TIGR|(protein codin</td><td></td><td></td><td></td></tr>
<tr><td></td><td>K.DLGADS</td><td>4.2752</td><td>0.2681</td><td>99.9</td><td>2075.072</td><td>2074.063</td><td></td><td>8930.7</td><td>1</td><td>5.543</td><td>41.7</td><td>2</td></tr>
<tr><td></td><td>K.DLGADS</td><td>6.6654</td><td>0.5189</td><td>100</td><td>2082.092</td><td>2081.08</td><td></td><td>9942.7</td><td>1</td><td>9.16</td><td>75</td><td>38</td></tr>
<tr><td></td><td>K.INTVQDA</td><td>4.2082</td><td>0.3714</td><td>100</td><td>1521.784</td><td>1521.78</td><td></td><td>8645.5</td><td>1</td><td>8.07</td><td>87.5</td><td>5</td></tr>
<tr><td>PFI1030c</td><td></td><td>43</td><td>23.30%</td><td>163</td><td>18688</td><td>5.5</td><td>U</td><td></td><td>Annotation|Plasmodium_falciparum_Sanger|(protein coc</td><td></td><td></td><td></td></tr>
<tr><td></td><td>K.FTQEM</td><td>3.9744</td><td>0.5161</td><td>100</td><td>1860.848</td><td>1860.849</td><td></td><td>5610</td><td>1</td><td>8.619</td><td>64.3</td><td>19</td></tr>
<tr><td></td><td>R.VCISIL</td><td>4.3902</td><td>0.4223</td><td>100</td><td>2746.407</td><td>2743.403</td><td></td><td>5352.9</td><td>1</td><td>7.788</td><td>50</td><td>15</td></tr>
<tr><td></td><td>R.VCISIL</td><td>6.2141</td><td>0.5143</td><td>100</td><td>2746.41</td><td>2743.403</td><td></td><td>5537.2</td><td>1</td><td>9.558</td><td>39.8</td><td>9</td></tr>
<tr><td>PF08_0081</td><td></td><td>56</td><td>23.20%</td><td>577</td><td>67077</td><td>6.2</td><td>U</td><td></td><td>Annotation|Plasmodium_falciparum_Sanger|(protein coc</td><td></td><td></td><td></td></tr>
<tr><td></td><td>R.ILLFGD</td><td>4.3721</td><td>0.3497</td><td>100</td><td>3737.664</td><td>3735.684</td><td></td><td>9027.5</td><td>1</td><td>6.194</td><td>28.3</td><td>3</td></tr>
<tr><td></td><td>K.NVVIHY</td><td>4.3017</td><td>0.5054</td><td>100</td><td>1610.954</td><td>1607.946</td><td></td><td>8218.4</td><td>1</td><td>8.611</td><td>80.8</td><td>19</td></tr>
<tr><td></td><td>K.FPPNTF</td><td>6.3526</td><td>0.5739</td><td>100</td><td>3888.896</td><td>3887.878</td><td></td><td>7017.8</td><td>1</td><td>8.97</td><td>28.9</td><td>5</td></tr>
<tr><td></td><td>K.KSSLPF</td><td>3.5716</td><td>0.2019</td><td>99.8</td><td>1356.8</td><td>1355.794</td><td></td><td>5426.5</td><td>1</td><td>6.014</td><td>81.8</td><td>9</td></tr>
<tr><td></td><td>K.SSLPFLI</td><td>2.9574</td><td>0.3331</td><td>99.9</td><td>1234.735</td><td>1234.716</td><td></td><td>4660.1</td><td>2</td><td>5.383</td><td>70</td><td>1</td></tr>
<tr><td></td><td>H.SSWLPV</td><td>4.4291</td><td>0.4501</td><td>100</td><td>2527.377</td><td>2526.366</td><td></td><td>7976.1</td><td>1</td><td>7.701</td><td>50</td><td>2</td></tr>
<tr><td></td><td>K.IPPQVFI</td><td>3.4705</td><td>0.3805</td><td>100</td><td>1445.768</td><td>1445.768</td><td></td><td>3965.2</td><td>1</td><td>6.452</td><td>81.8</td><td>4</td></tr>
<tr><td></td><td>K.LGHVNE</td><td>3.954</td><td>0.2083</td><td>99.9</td><td>1363.685</td><td>1363.683</td><td></td><td>5633.7</td><td>1</td><td>4.232</td><td>67.5</td><td>4</td></tr>
<tr><td></td><td>K.LGHVNE</td><td>3.1866</td><td>0.2816</td><td>99.9</td><td>1366.695</td><td>1363.683</td><td></td><td>5139.2</td><td>1</td><td>4.793</td><td>80</td><td>9</td></tr>
<tr><td>PFI0180w</td><td></td><td>65</td><td>23.20%</td><td>453</td><td>50297</td><td>5.1</td><td>U</td><td></td><td>Annotation|Plasmodium_falciparum_Sanger|(protein coc</td><td></td><td></td><td></td></tr>
<tr><td></td><td>R.CVFVDL</td><td>4.4159</td><td>0.3194</td><td>100</td><td>1777.886</td><td>1776.884</td><td></td><td>5451.4</td><td>1</td><td>7.094</td><td>78.6</td><td>3</td></tr>
</table>

	R.QLFHPE	3.1679	0.3303	100	1404.783	1403.776	3620.6		1	6.032	77.3	9
	R.QLFHPE	3.6476	0.2426	99.9	2385.195	2385.195	5546.6		1	5.274	52.5	2
	R.QLFHPE	4.7055	0.4067	100	2393.213	2392.212	7002.5		1	7.671	32.5	5
	R.LIAQVIS	3.7596	0.4251	100	1485.921	1485.919	8753.3		1	7.344	76.9	5
	R.FDGALN	4.2136	0.3293	100	2397.2	2395.193	7777.4		1	7.151	52.5	24
	R.IHFMLS	5.1782	0.4937	100	1785.935	1785.932	10288.3		1	8.827	76.7	15
	K.CGINYQ	4.3935	0.4898	100	1893.974	1892.965	4981.4		1	8.357	47.1	2
PFF1360w		9	23.10%	173	20061	6.4	U	Annotation|Plasmodium_falciparum_Sanger|(protein coc				
	K.LDHHFIL	3.646	0.3681	100	1824.028	1824.025	5548.7		1	6.902	64.3	5
	K.HSSTEE	3.9155	0.3794	100	2889.461	2889.456	5504.2		1	6.79	41.7	4
PFD0665c		33	23.00%	392	44666	7.5	U	Annotation|Plasmodium_falciparum_Sanger|(protein coc				
	R.IQSVPL	4.1141	0.4499	100	3630.936	3629.886	9949.9		1	8.112	32.8	7
	R.IQSVPL	3.5991	0.3485	99.8	3672.971	3671.989	8916.2		1	5.07	22.7	1
	V.PLIIGQ	4.0338	0.5102	100	3238.752	3237.728	7379.5		1	8.479	37.5	7
	K.IGIEPP	4.237	0.1996	99.9	1911.053	1910.046	4206.3		1	7.291	63.9	3
	A.RENSPS	3.9045	0.3873	100	2120.079	2118.108	6927.2		1	7.421	55.6	6
	R.ENSPSII	3.8262	0.4603	100	1990.059	1990.076	7399.7		1	6.29	44.1	7
	K.ISAADI	4.7725	0.5351	100	1928.075	1928.065	8712.2		1	9.264	58.3	2
PFL2505c		463	22.90%	2215	263153	9.2	U	Annotation|Plasmodium_falciparum_Sanger|(protein coc				
	K.YLISEF	3.8169	0.332	100	2225.081	2222.073	6557.7		1	6.679	44.1	6
	K.NLGTGF	4.3198	0.4668	100	1696.833	1693.823	8868.5		1	7.781	75	66
	K.YVMNYL	3.7168	0.3604	100	1833.954	1833.953	7378.5		1	7.174	64.3	3
	K.LLHYKP	4.3755	0.2845	100	2672.381	2669.362	5923		1	6.873	50	21
	N.PYSSLY	4.1389	0.2941	100	1475.705	1475.706	6797.3		1	7.902	86.4	3
	K.NPIGYQ	4.2744	0.4025	100	3087.559	3086.556	6575.9		2	7.172	31.5	7
	K.NPIGYQ	5.6949	0.4492	100	3088.564	3086.556	6976.4		1	7.15	33.3	4
	K.YSHHYE	2.7449	0.3792	100	1703.792	1703.825	6058.7		4	5.635	50	1
	K.AQEASC	5.3932	0.5401	100	2003.921	2003.916	7156.3		1	9.774	75	3
	K.AQEASC	5.3144	0.4543	100	2132.014	2132.011	6784.3		1	8.161	55.3	9
	R.INFNDI	3.7563	0.3821	100	1950.984	1949.983	5919.8		1	6.69	66.7	3
	K.NLHGLE	2.8896	0.3164	99.9	1152.644	1152.638	4777.3		21	5.452	60	3
	K.DLINIL	4.323	0.3331	100	1689.909	1688.906	7150.2		1	6.486	69.2	18
	R.NVVNNF	5.6285	0.3418	100	2475.274	2471.264	5831.7		1	7.169	47.6	19
	K.KYNVDI	2.942	0.2951	99.9	1278.702	1278.703	6153.3		1	5.795	77.8	1
	K.YPIDPY	3.7516	0.3589	100	2354.097	2353.088	5203.2		1	6.229	44.4	2
	K.SYQDIE	4.6318	0.4368	100	1941.933	1941.931	3888.1		1	8.098	63.3	1

	K.AHGSNV	4.4447	0.4924	100	1601.829	1601.829		3475.1	1	8.286	76.7	23
	K.NMSFSD	5.2511	0.3986	100	2640.263	2637.258		5949.4	1	8.664	50	31
	K.FLADSN	4.627	0.5069	100	2025.063	2025.063		4547.2	1	7.681	61.8	13
	R.RVNDGI	2.9006	0.3209	99.8	1575.746	1575.745		6010	1	5.213	62.5	2
	R.VNDGMI	2.5958	0.3553	99.9	1419.644	1419.644		6823.7	1	5.568	59.1	1
	K.DNVLDQ	2.9699	0.2706	99.8	1377.71	1376.706		8454.9	1	5.361	77.3	2
	L.NPGKPN	3.6302	0.4112	100	1248.705	1248.703		4607.4	1	6.507	86.4	3
	K.TGLIKED	3.5485	0.3597	100	1355.801	1355.797		5314.4	2	6.49	81.8	3
	K.LKEEEE	3.3869	0.1895	99.8	1276.653	1276.65		6611.3	1	5.184	77.8	1
	K.IVENILS	5.3618	0.4485	100	1704.93	1702.92		7602.2	1	8.692	84.6	31
	K.IVENILS	4.8392	0.3019	100	1925.999	1924.991		7078.5	1	6.542	76.7	12
	K.DKSDDD	8.0336	0.4542	100	2905.198	2904.188		10848.8	1	10.064	42.3	3
	K.DKSDDD	5.3716	0.5401	100	2905.198	2904.188		9257.8	1	11.48	51.9	3
	K.SDDDQS	5.59	0.5494	100	2662.076	2661.066		6535.1	1	10.366	50	6
	K.NTTYAE	3.247	0.4531	100	3333.477	3333.474		6461.5	2	6.657	23.2	1
	K.VVHALIS	2.5344	0.3405	99.9	1100.689	1100.691		5353.5	1	6.072	77.8	2
	K.IFNNFQ	4.5877	0.4218	100	2231.033	2230.031		4383.8	1	7.253	61.1	27
	K.IFNNFQ	3.794	0.4332	100	3500.722	3500.709		8392.9	1	6.823	25	6
	K.IFNNFQ	5.7309	0.4122	100	3501.72	3500.709		9564.9	1	6.755	33	20
	R.YTHDHD	5.7173	0.4851	100	2320.988	2319.98		4631.9	1	8.42	58.3	9
	S.LEKPSL	4.4449	0.4268	100	3023.544	3022.52		5262.8	1	6.365	29	3
	K.PSLEQP	4.7387	0.4086	100	2653.304	2652.299		7771.6	1	6.836	28.4	10
	K.PSLEQP	4.6622	0.3971	100	2656.308	2652.299		8327.7	1	7.39	50	45
	Q.PVLTLE	4.9301	0.5126	100	2099.036	2098.029		7466.5	1	8.621	58.8	36
PF14_0567		62	22.90%	340	40069	9.6	U		Annotation|Plasmodium_falciparum_TIGR|(protein codin			
	K.EKEDDH	3.8544	0.3984	100	1945.907	1945.91		4972.2	1	6.285	65.6	4
	K.NDKNTS	4.8908	0.5593	100	1679.902	1679.902		5441	1	9.097	75	1
	K.NTSIHP	3.3609	0.4371	100	1308.701	1308.702		3287.4	1	7.939	63.6	1
	K.NTSIHP	3.2537	0.3535	100	1322.739	1322.737		3906.5	1	6.014	68.2	1
	R.EALVFN	3.2339	0.3707	100	1397.749	1397.747		7389.9	1	6.067	72.7	6
	K.KNDVHV	4.8421	0.4857	100	1790.915	1787.911		6245.9	1	7.703	82.1	28
	K.NVETFY	3.3815	0.4611	100	2282.975	2281.972		5529.6	1	7.679	52.9	12
	K.NVETFY	3.4983	0.4362	100	2412.069	2410.067		7519.9	1	6.879	44.4	9
PF08_0075		9	22.80%	215	25441	10.8	U		Annotation|Plasmodium_falciparum_Sanger|(protein coc			
	H.PIVHCP	2.5332	0.3245	99.8	1114.59	1114.591		7027.5	1	5.557	81.2	1
	G.KGFTFE	2.7616	0.2934	99.9	1098.579	1098.584		4744.1	1	5.502	81.2	2

	K.KGIGGI	4.1073	0.4137	100	1618.95	1618.943		6513.8	1	6.505	70	5
	K.EKNTKP	2.9502	0.3901	99.8	1819.958	1819.956		4500.7	1	6.79	53.6	1
PFD0305c		31	22.70%	494	55789	5.7	U		Annotation|Plasmodium_falciparum_Sanger|(protein coc			
	K.TISGVQ	3.5004	0.3175	99.8	1689.013	1689.01		6664.9	1	6.405	50	1
	K.TISGVQ	3.6783	0.4416	100	2061.23	2061.226		7506	1	7.139	44.4	11
	R.VFNGSC	2.8685	0.373	100	1161.628	1161.627		5306.1	35	6.686	60	2
	K.EMIQTG	3.4686	0.3228	99.9	2010.041	2007.026		6250.2	1	6.288	58.8	2
	F.SAAGLP	3.989	0.3564	100	1707.879	1707.879		5886.4	1	6.825	66.7	5
	F.SAAGLP	4.3023	0.5233	100	1707.88	1707.879		5587.3	1	9.027	53.3	3
	S.AAGLPH	2.9564	0.4155	100	1606.819	1606.812		2873.3	1	6.152	64.3	2
	K.AVIGEE	3.2145	0.3094	99.9	2772.407	2771.403		5038.6	1	5.885	39.1	2
	R.FITQNT	2.5608	0.3121	99.8	1338.624	1338.623		7282.3	1	5.533	88.9	1
	R.DIYQSLI	3.6827	0.3395	100	1748.941	1748.941		6334.7	1	6.875	73.1	2
PF11_0461		24	22.70%	176	20093	6	U		Annotation|Plasmodium_falciparum_TIGR|(protein codin			
	R.DSAAAI	3.0687	0.3025	99.9	1507.768	1507.776		7698.4	1	7.489	73.1	2
	R.GKDVII	4.2336	0.5149	100	1240.782	1240.782		7444.8	1	8.703	86.4	19
	K.AQEYNT	4.0194	0.4208	100	1656.736	1656.733		5248.5	1	7.538	69.2	3
PF10_0268		220	22.60%	393	43930	9.7	U		Annotation|Plasmodium_falciparum_TIGR|(protein codin			
	K.VEVLNH	5.6629	0.4413	100	2209.048	2209.041		7584.5	1	8.847	58.3	42
	N.HNGETT	5.1544	0.5054	100	1654.736	1654.735		6870.3	1	10.203	84.6	9
	K.HKENNE	3.8075	0.3855	100	1774.914	1774.913		6843.4	1	6.457	71.4	2
	F.KEKHEE	3.5251	0.209	99.9	1401.706	1401.713		6049.7	1	5.486	75	3
	K.EKHEEF	3.3313	0.3369	99.8	1273.615	1273.618		3783.3	1	5.969	77.8	1
	K.EKHEEF	3.5436	0.2745	100	1273.622	1273.618		3860.5	1	5.446	83.3	10
	K.YVVYGL	4.0085	0.5034	100	1771.883	1771.875		5705.4	1	9.412	66.7	9
	K.YVVYGL	4.9323	0.5002	100	1771.888	1771.875		8893.4	1	8.779	76.7	19
	G.LSADTA	3.3573	0.2315	99.9	1190.592	1190.591		7891.8	1	6.47	90	3
	K.EKLELP	3.3156	0.2293	99.8	1406.729	1406.724		5989.6	1	5.431	70	3
	K.EKLELP	5.0866	0.1539	99.9	1863.944	1863.941		4623.4	1	6.206	71.4	20
	K.EKLELP	4.3428	0.3257	100	1863.944	1863.941		4575.3	1	6.63	60.7	3
	K.EKLELP	3.5023	0.2847	99.9	2205.166	2204.152		7723.7	68	5.362	32.4	3
	K.EKLELP	5.1354	0.4181	100	2332.255	2332.247		6435.7	1	7.409	63.9	36
	K.LELPYE	3.3165	0.3409	99.8	1606.808	1606.804		7631	1	6.711	62.5	1
	K.LELPYE	3.7253	0.3586	100	1606.809	1606.804		5272.3	1	6.893	79.2	2
	K.LELPYE	5.7336	0.2234	100	2075.115	2075.11		7635.2	1	5.57	71.9	48
	L.PYELLC	4.3228	0.2687	100	1719.904	1719.899		8284.6	1	5.625	80.8	3

	K.NDFTVS	2.7551	0.2913	99.9	1072.533	1072.532		6231.6	1	7.054	87.5	3
PF10_0166		25	22.60%	310	36405	5.9	U		Annotation|Plasmodium_falciparum_TIGR|(protein codin			
	K.HFGSNS	3.5221	0.332	100	1578.759	1578.751		4594.6	1	7.642	73.1	3
	N.KNMMT	4.4631	0.527	100	1580.653	1580.651		6700.6	1	9.454	79.2	3
	K.NVCQFI	4.4404	0.2992	100	1805.839	1805.835		8955.6	1	6.687	73.1	6
	K.NFCNITS	4.9306	0.4743	100	1729.868	1729.866		7227.3	1	8.321	84.6	7
	K.FCYGGI	4.8056	0.5091	100	1737.828	1737.824		4331.4	1	9.485	75	6
PF07_0080		46	22.60%	137	16478	9.7	U		Annotation|Plasmodium_falciparum_Sanger|(protein coc			
	K.HQYFIL	4.8807	0.4518	100	1922.994	1922.995		7964	1	9.472	78.6	44
	R.EFLHLP	4.2979	0.3939	100	1804.018	1804.012		3807.4	1	6.728	50	2
PF11_0338		50	22.50%	258	28297	7.3	U		Annotation|Plasmodium_falciparum_TIGR|(protein codin			
	K.IPQFAW	2.931	0.3233	99.9	1235.628	1234.622		4615.6	1	5.969	88.9	8
	I.PQFAWE	2.4514	0.3436	99.8	1121.538	1121.538		4746.1	1	6.044	81.2	3
	R.FLSLIAY	3.2833	0.2193	99.9	1018.607	1018.605		3540.8	1	5.906	93.8	10
	Y.FWVPLV	3.5448	0.3793	100	2519.238	2518.23		6086.6	1	6.302	50	3
	W.VPLVAF	3.2911	0.369	100	2186.05	2185.083		5225.5	1	6.351	58.3	3
	V.PLVAPC	4.3987	0.2571	100	2086.017	2086.014		7174	2	6.441	52.9	3
	A.PCVGSV	4.4951	0.475	100	1706.777	1705.772		8866.9	1	7.819	76.9	9
	K.VICPLV	3.4136	0.2712	99.9	1493.797	1491.795		5701	9	6.465	58.3	2
	K.VICPLV	4.1168	0.3449	100	1985.015	1984.006		8980.9	1	6.816	64.7	9
PFC0255c		5	22.50%	142	16140	5	U		Annotation|Plasmodium_falciparum_Sanger|(protein coc			
	R.IYSLTI	3.9061	0.3087	100	2362.157	2359.136		3557.6	1	6.896	42.1	4
	R.NYTIETI	3.2497	0.2588	99.9	1456.869	1456.868		8729.4	1	5.502	72.7	1
MAL7P1.119		242	22.40%	749	87879	5.5	U		Annotation|Plasmodium_falciparum_Sanger|(protein coc			
	K.VPNVAH	3.2119	0.3577	99.8	1135.624	1135.622		4293.4	1	6.357	70	4
	K.VPNVAH	3.8058	0.3443	100	1138.631	1135.622		6404.2	1	5.942	90	9
	K.EVIKDS	5.0783	0.4226	100	1936.96	1935.956		4430.1	1	7.571	66.7	6
	K.DSNKDN	3.4548	0.325	100	1466.669	1466.666		5556.6	1	6.597	72.7	7
	K.QNVNKF	3.0064	0.2907	99.9	1337.77	1337.766		5298	2	5.043	65	1
	K.YNENDY	4.3631	0.343	100	1911.813	1911.811		4322.2	2	7.548	64.3	6
	R.QNFNNF	4.8791	0.4509	100	2373.093	2372.084		7304.1	1	7.763	52.8	103
	K.NYIDKL	4.034	0.2195	99.9	1563.807	1563.802		7855.3	2	5.228	75	2
	K.NIIDTIL	4.66	0.3886	100	1989.046	1989.04		9140	1	7.352	62.5	1
	K.NIIDTIL	6.6314	0.5312	100	2120.146	2117.135		9405.4	1	9.724	73.5	38
	K.ILDLEE	4.121	0.2601	100	1558.846	1557.838		8380.2	2	5.356	87.5	6
	K.NIIEVLM	3.1918	0.2123	99.9	1016.582	1016.581		2782.7	1	4.363	87.5	7

<table>
<tr><td></td><td>R.VDLAQN</td><td>3.5765</td><td>0.3693</td><td>100</td><td>1419.792</td><td>1419.792</td><td></td><td>6760.8</td><td>1</td><td>6.577</td><td>70.8</td><td>2</td></tr>
<tr><td></td><td>R.VDLAQN</td><td>4.8863</td><td>0.4672</td><td>100</td><td>2123.201</td><td>2122.199</td><td></td><td>6714.2</td><td>1</td><td>7.364</td><td>63.9</td><td>43</td></tr>
<tr><td></td><td>K.NIDKAE</td><td>3.2289</td><td>0.2505</td><td>99.9</td><td>1294.709</td><td>1294.708</td><td></td><td>6169.4</td><td>3</td><td>5.853</td><td>70</td><td>1</td></tr>
<tr><td></td><td>K.YFLNSH</td><td>4.4328</td><td>0.4185</td><td>100</td><td>1737.795</td><td>1735.79</td><td></td><td>6618.2</td><td>1</td><td>7.893</td><td>83.3</td><td>6</td></tr>
<tr><td>PF13_0142</td><td></td><td>21</td><td>22.10%</td><td>77</td><td>8844</td><td>4.6</td><td>U</td><td></td><td colspan="4">Annotation|Plasmodium_falciparum_Sanger|(protein coc</td></tr>
<tr><td></td><td>K.GILACL</td><td>2.4816</td><td>0.3317</td><td>99.9</td><td>1046.533</td><td>1046.53</td><td></td><td>5902.6</td><td>1</td><td>6.131</td><td>87.5</td><td>2</td></tr>
<tr><td></td><td>R.GNNVFY</td><td>3.4682</td><td>0.306</td><td>100</td><td>989.5303</td><td>989.5282</td><td></td><td>3793.9</td><td>1</td><td>6.056</td><td>85.7</td><td>19</td></tr>
<tr><td>PF08_0091</td><td></td><td>151</td><td>21.90%</td><td>1210</td><td>144007</td><td>7</td><td>U</td><td></td><td colspan="4">Annotation|Plasmodium_falciparum_Sanger|(protein coc</td></tr>
<tr><td></td><td>G.DDNGE</td><td>5.6821</td><td>0.4285</td><td>100</td><td>2371.007</td><td>2371.003</td><td></td><td>4736.8</td><td>1</td><td>9.21</td><td>62.5</td><td>3</td></tr>
<tr><td></td><td>K.MYALET</td><td>4.2307</td><td>0.4832</td><td>100</td><td>1554.79</td><td>1554.788</td><td></td><td>9120.5</td><td>1</td><td>9.068</td><td>79.2</td><td>6</td></tr>
<tr><td></td><td>K.IWSAQG</td><td>4.3463</td><td>0.3377</td><td>100</td><td>1855.971</td><td>1852.974</td><td></td><td>9330.5</td><td>1</td><td>6.093</td><td>73.3</td><td>10</td></tr>
<tr><td></td><td>K.ESNKPI</td><td>4.2014</td><td>0.3849</td><td>100</td><td>2684.239</td><td>2684.23</td><td></td><td>7900</td><td>1</td><td>6.965</td><td>30.7</td><td>1</td></tr>
<tr><td></td><td>K.DLDNPE</td><td>3.1224</td><td>0.3937</td><td>100</td><td>2482.059</td><td>2482.061</td><td></td><td>4183.9</td><td>1</td><td>5.813</td><td>42.9</td><td>1</td></tr>
<tr><td></td><td>K.FSYDYT</td><td>3.9551</td><td>0.369</td><td>100</td><td>2048.952</td><td>2047.948</td><td></td><td>6093.5</td><td>1</td><td>6.926</td><td>65.6</td><td>18</td></tr>
<tr><td></td><td>R.SLTYYL</td><td>3.2018</td><td>0.2955</td><td>99.9</td><td>1289.691</td><td>1289.689</td><td></td><td>7771.5</td><td>1</td><td>6.126</td><td>77.8</td><td>6</td></tr>
<tr><td></td><td>R.IPSHLS</td><td>5.0622</td><td>0.4089</td><td>100</td><td>2497.24</td><td>2497.24</td><td></td><td>8131.6</td><td>1</td><td>8.645</td><td>60</td><td>51</td></tr>
<tr><td></td><td>K.NPYLEH</td><td>4.3165</td><td>0.2698</td><td>100</td><td>1845.961</td><td>1844.959</td><td></td><td>7987.8</td><td>1</td><td>5.484</td><td>71.4</td><td>16</td></tr>
<tr><td></td><td>K.HIAGSA</td><td>4.6433</td><td>0.4243</td><td>100</td><td>1765.895</td><td>1765.895</td><td></td><td>6831.5</td><td>1</td><td>8.239</td><td>81.2</td><td>5</td></tr>
<tr><td></td><td>K.FNDEEN</td><td>3.9311</td><td>0.413</td><td>100</td><td>2566.192</td><td>2565.19</td><td></td><td>6786.3</td><td>1</td><td>7.377</td><td>55.3</td><td>16</td></tr>
<tr><td></td><td>K.ESVEAG</td><td>4.629</td><td>0.4994</td><td>100</td><td>3014.544</td><td>3013.538</td><td></td><td>8284.3</td><td>1</td><td>7.738</td><td>35.6</td><td>4</td></tr>
<tr><td></td><td>H.HRNDDI</td><td>4.3721</td><td>0.3762</td><td>100</td><td>1914.934</td><td>1913.929</td><td></td><td>7470.5</td><td>1</td><td>6.659</td><td>80</td><td>2</td></tr>
<tr><td></td><td>K.NGLNTD</td><td>6.3779</td><td>0.5209</td><td>100</td><td>3441.676</td><td>3441.662</td><td></td><td>8255.3</td><td>1</td><td>8.544</td><td>33.9</td><td>9</td></tr>
<tr><td></td><td>K.NGLNTD</td><td>6.4159</td><td>0.466</td><td>100</td><td>3442.678</td><td>3441.662</td><td></td><td>8617</td><td>1</td><td>7.272</td><td>26.8</td><td>3</td></tr>
<tr><td>PF11_0302</td><td></td><td>55</td><td>21.90%</td><td>452</td><td>51952</td><td>5.1</td><td>U</td><td></td><td colspan="4">Annotation|Plasmodium_falciparum_TIGR|(protein codin</td></tr>
<tr><td></td><td>K.SAEAVH</td><td>3.2476</td><td>0.2951</td><td>99.9</td><td>1128.609</td><td>1127.606</td><td></td><td>6289.1</td><td>1</td><td>5.643</td><td>85</td><td>13</td></tr>
<tr><td></td><td>K.VFSDEN</td><td>3.813</td><td>0.3027</td><td>100</td><td>1342.674</td><td>1341.669</td><td></td><td>6140.4</td><td>1</td><td>5.961</td><td>80</td><td>6</td></tr>
<tr><td></td><td>K.GVMPSE</td><td>3.4274</td><td>0.309</td><td>99.9</td><td>1452.665</td><td>1451.659</td><td></td><td>3652.1</td><td>1</td><td>6.769</td><td>75</td><td>9</td></tr>
<tr><td></td><td>K.GVMPSE</td><td>3.088</td><td>0.4469</td><td>100</td><td>1579.756</td><td>1579.754</td><td></td><td>4705.3</td><td>1</td><td>7.014</td><td>57.7</td><td>3</td></tr>
<tr><td></td><td>K.NQNEDN</td><td>5.5326</td><td>0.4935</td><td>100</td><td>3648.621</td><td>3646.604</td><td></td><td>8443.7</td><td>1</td><td>8.18</td><td>30.8</td><td>1</td></tr>
<tr><td></td><td>K.NVTLLI</td><td>3.5123</td><td>0.3304</td><td>100</td><td>1818.878</td><td>1817.87</td><td></td><td>6313.1</td><td>1</td><td>5.573</td><td>50</td><td>2</td></tr>
<tr><td></td><td>K.HISTTC</td><td>5.6576</td><td>0.5669</td><td>100</td><td>1770.871</td><td>1770.867</td><td></td><td>4898.1</td><td>1</td><td>10.237</td><td>83.3</td><td>21</td></tr>
<tr><td>MAL7P1.122</td><td></td><td>67</td><td>21.90%</td><td>393</td><td>45164</td><td>7.3</td><td>U</td><td></td><td colspan="4">Annotation|Plasmodium_falciparum_Sanger|(protein coc</td></tr>
<tr><td></td><td>K.NAHLGE</td><td>6.3415</td><td>0.4962</td><td>100</td><td>2864.5</td><td>2864.5</td><td></td><td>7948.3</td><td>1</td><td>9.207</td><td>36.5</td><td>9</td></tr>
<tr><td></td><td>K.NAHLGE</td><td>4.747</td><td>0.4477</td><td>100</td><td>2864.509</td><td>2864.5</td><td></td><td>6517.2</td><td>1</td><td>8.766</td><td>44.2</td><td>27</td></tr>
<tr><td></td><td>S.NIAAVD</td><td>4.7445</td><td>0.3897</td><td>100</td><td>1441.821</td><td>1440.815</td><td></td><td>5485.8</td><td>1</td><td>7.711</td><td>83.3</td><td>10</td></tr>
<tr><td></td><td>R.AFENED</td><td>5.6208</td><td>0.5547</td><td>100</td><td>2089.069</td><td>2089.062</td><td></td><td>6506.2</td><td>1</td><td>9.815</td><td>67.6</td><td>7</td></tr>
</table>

	K.VKQNEH	3.9521	0.4289	100	2000.095	2000.082		7916.8	1	7.7	62.5	9	
	K.KQYFET	2.7875	0.2766	99.8	1284.654	1284.659		8865.5	1	5.134	77.8	1	
	K.APQAAC	3.9305	0.3326	100	1492.791	1490.772		8420.6	1	6.722	76.9	4	
PFL1110c		77	21.80%	441	50833	7.4	U		Annotation|Plasmodium_falciparum_Sanger|(protein coc				
	R.KVLDLE	3.3294	0.3329	100	1456.846	1455.84		8864.1	2	6.304	59.1	3	
	R.KVLDLE	4.4121	0.1919	99.9	1569.905	1569.9		5333	1	4.994	64.6	3	
	R.KVLDLE	4.5716	0.1915	100	1584.943	1583.935		4111	1	5.353	75	16	
	K.VLDLES	4.5427	0.3881	100	1456.847	1455.84		8455.8	1	6.468	77.3	25	
	R.EALNES	3.3468	0.2698	99.8	1803.943	1803.939		4118.1	1	5.03	57.1	2	
	K.GVNIIN	4.5005	0.3544	100	2930.446	2928.452		9898.8	1	6.616	36	9	
	K.MYEDIL	4.0242	0.3993	100	1588.859	1588.86		6617.9	1	7.359	75	1	
	K.EGEEGI	5.1003	0.5124	100	2126.043	2126.048		9518	1	9.093	50	9	
	L.GPIEDIL	3.2005	0.4121	100	1063.611	1063.609		5512.4	1	6.744	93.8	9	
PF13_0275		20	21.80%	285	32784	9.2	U		Annotation|Plasmodium_falciparum_Sanger|(protein coc				
	R.SSSNSH	3.3886	0.3556	100	1665.748	1665.747		4744.9	1	6.459	64.3	3	
	R.SSSNS	5.9412	0.4187	100	4187.848	4185.835		6355.9	1	8.248	27.1	3	
	K.KADNFII	3.0405	0.2808	99.9	1065.56	1065.558		5277.5	1	5.096	87.5	2	
	K.RYGSGS	3.2515	0.4335	100	1801.783	1800.779		5439.1	1	6.753	53.3	12	
PFC0300c		44	21.80%	257	30523	10.3	U		Annotation|Plasmodium_falciparum_Sanger|(protein coc				
	R.LLQVHN	3.2863	0.283	99.9	1253.737	1253.737		7536.7	2	6.083	70	24	
	K.IVEPYV	3.9295	0.369	100	1959.031	1958.028		8077.4	1	7.159	56.2	5	
	K.YNVHGI	5.9669	0.4851	100	2535.194	2533.19		8504.3	1	9.687	65	12	
	R.HAFNEF	2.3785	0.3469	99.9	870.4217	870.4222		3718	1	6.665	83.3	3	
PF11_0174		103	21.60%	700	80412	6.2	U		Annotation|Plasmodium_falciparum_TIGR|(protein codin				
	R.TKTSPN	4.7462	0.4615	100	1821.89	1820.881		7563.3	1	8.424	71.9	6	
	K.TSPNLT	3.9079	0.4841	100	1591.74	1591.739		3993.2	1	7.843	64.3	7	
	K.NTYNVG	3.3847	0.275	99.9	1423.737	1422.734		5653.8	1	5.26	77.3	6	
	K.YLLENN	4.4591	0.3725	100	4389.077	4384.076		9338.1	1	5.722	20.7	2	
	R.VIGTWT	4.2113	0.403	100	1889.968	1889.962		8813.2	1	7.725	63.3	13	
	K.IGNETY	5.0624	0.4932	100	1914.919	1914.913		4532.5	1	8.402	59.4	8	
	R.YGWLD'	3.3781	0.3053	100	1252.588	1252.585		4594.4	1	6.221	88.9	3	
	R.YGWLD'	5.6646	0.4077	100	2767.245	2766.237		7385.9	1	7.176	42.9	27	
	R.YGWLD'	4.3817	0.3965	100	2768.244	2766.237		7580.8	1	7.115	42.9	6	
	K.NEHLHG	3.0438	0.3668	100	1532.674	1532.67		5728.7	1	6.178	77.3	3	
	R.NGPIVS	5.4209	0.5086	100	3520.602	3518.593		6402.5	1	7.861	26.7	22	
PF13_0287		38	21.50%	442	50065	7.6	U		Annotation|Plasmodium_falciparum_Sanger|(protein coc				

	R.FNGGAI	3.6332	0.2639	99.9	1608.783	1608.784		5758.7		1	5.213	80	2
	R.FNGGAI	4.0704	0.3282	100	1729.856	1729.862		5724.2		1	5.991	56.2	3
	K.AHILFDI	5.96	0.4693	100	2156.221	2156.22		9130.4		1	8.824	67.6	8
	K.ELNLFY	4.0349	0.165	99.9	1454.753	1453.748		6923.9		1	4.728	85	17
	R.IVDVIS	3.8405	0.449	100	1980.036	1979.027		7811.9		1	7.4	43.8	4
	R.VGCGPI	3.3379	0.3035	99.9	2152.055	2152.05		8468.2		1	5.473	55.6	3
	R.CGWLDI	3.7209	0.265	99.9	1614.853	1614.85		6730.2		3	5.659	62.5	1
PF13_0143		67	21.50%	437	49383	9.3	U		Annotation|Plasmodium_falciparum_Sanger|(protein coc				
	S.SRVPIS	3.5581	0.3394	100	1248.712	1248.714		4770.3		1	6.559	68.2	4
	R.VPISAA	3.1157	0.2541	99.9	1005.578	1005.581		5319.3		1	6.24	88.9	1
	R.VVAIDL	5.2593	0.513	100	1942.052	1941.035		8099		1	8.94	73.5	18
	R.VVAIDL	3.671	0.3655	100	1955.061	1955.069		5716.5		1	6.178	36.8	1
	R.VPVDNL	5.3774	0.5606	100	2042.072	2042.092		9443.9		1	9.715	64.7	16
	K.KDLYKP	5.4257	0.5299	100	1977.084	1977.081		3783.3		1	9.185	70.6	10
	F.AFATHG	3.1551	0.4191	100	1559.809	1559.797		7764.8		1	7.311	71.4	4
	F.ATHGLF	2.8779	0.3984	99.8	1348.712	1348.709		4247.7		1	7.017	66.7	1
	K.SPLEEV	3.9508	0.3718	100	1415.767	1415.763		6825		1	6.684	75	3
	K.SPLEEV	3.5744	0.4339	100	1415.768	1415.763		5737.5		1	7.862	70.8	9
PF07_0112		6	21.50%	256	28388	5.1	U		Annotation|Plasmodium_falciparum_Sanger|(protein coc				
	K.NGPCLV	2.7967	0.4415	100	1852.833	1852.829		7130.4		1	6.643	50	3
	R.FSAASI	3.7763	0.2953	100	2335.183	2333.174		4452.1		1	5.76	40.5	1
	R.IIDVLPS	3.5395	0.5308	100	1866.04	1866.023		6328.6		1	8.781	50	2
		48	21.40%	645	65865	8	U		no description				
	R.GFSSGS	4.1423	0.3674	100	1254.608	1254.608		8149.2		1	8.801	80.8	3
	R.FLEQQN	4.6666	0.3472	100	1475.788	1475.786		11713.9		1	6.673	90.9	17
	K.WELLQC	5.1338	0.3556	100	3304.736	3302.725		7361.3		1	6.542	31.5	2
	R.PINLEP	4.673	0.3032	100	2005.102	2003.097		4553.9		1	6.179	42.2	2
	R.TAAENE	3.4169	0.2633	99.9	1336.712	1336.711		6747.1		1	5.731	81.8	3
	K.VDLLNQ	3.5859	0.2042	99.8	1460.804	1460.8		7575.9		1	5.597	77.3	2
	R.NLDLDS	4.4547	0.2712	100	1332.738	1329.727		5961.4		1	6.08	81.8	12
	R.LQGEIA	2.7187	0.3176	99.9	1122.664	1122.664		4862.6		1	5.551	83.3	2
	R.NKLNDL	4.2001	0.2318	99.9	1614.856	1613.85		8808.3		1	4.927	80.8	3
	R.GGSGG	4.4827	0.3805	100	1741.752	1741.749		5572.5		1	9.984	45.5	2
PFC0745c		45	21.40%	252	29289	6.8	U		Annotation|Plasmodium_falciparum_Sanger|(protein coc				
	R.IYHVNN	6.1474	0.5291	100	2311.193	2310.189		7032.1		1	9.261	63.2	2
	K.NNLQNL	4.7888	0.4841	100	1757.897	1756.892		9722.2		1	8.602	76.9	41

	K.ESSYEF	4.3601	0.3668	100	2378.197	2376.191		7454.2	1	7.257	50	2	
PFD1120c		36	21.30%	136	14891	9.4	U		Annotation|Plasmodium_falciparum_Sanger|(protein coc				
	G.KNVNVD	3.6525	0.4423	100	1340.728	1340.732		6859.1	1	7.792	72.7	1	
	K.SNKKPE	4.0412	0.4847	100	1776.855	1776.862		5636.8	1	7.944	63.3	12	
	K.SNKKPE	4.1572	0.4331	100	1904.952	1904.957		6160.2	1	8.011	62.5	7	
	K.KPEVSG	3.7267	0.2673	99.9	1447.691	1447.692		3603	1	5.961	70.8	3	
	K.KPEVSG	4.6538	0.3888	100	1575.788	1575.787		5311.3	1	7.695	84.6	13	
PF14_0324		76	21.10%	564	66057	7	U		Annotation|Plasmodium_falciparum_TIGR|(protein codin				
	K.YFSDAI	4.7584	0.3091	99.9	3044.473	3043.48		9808.5	1	6.705	30.6	12	
	K.YFSDAI	5.5041	0.5632	100	3050.507	3050.497		8758.9	1	9.809	40.7	41	
	K.VRNENM	7.6177	0.5024	100	3167.67	3164.679		4860.7	1	9.741	40.4	6	
	R.NENMLE	3.986	0.4956	100	2909.543	2909.509		6808.4	1	8.558	47.9	6	
	K.SYKEEN	6.0611	0.2758	100	2180.071	2179.067		6038.5	1	7.744	67.6	5	
	R.TPEQIQ	3.613	0.3939	100	1288.626	1288.625		6910.8	1	6.717	70	3	
	K.EAYIDP	3.4222	0.2456	99.9	1551.737	1551.741		5316.4	3	5.767	66.7	1	
	K.SMADPE	3.526	0.2485	99.8	2542.335	2542.323		7022.6	1	4.806	47.6	2	
PF14_0124		67	21.00%	376	42606	5.3	U		Annotation|Plasmodium_falciparum_TIGR|(protein codin				
	T.LKYPIE	4.7332	0.3712	100	2188.066	2188.075		8887.9	1	7.666	73.5	4	
	K.YPIEHG	4.7132	0.4548	100	1946.898	1946.896		7883	1	9.112	56.7	3	
	K.YPIEHG	5.3916	0.4571	100	1961.932	1960.93		10155.3	1	8.75	76.7	16	
	Y.PIEHGI	4.7814	0.5175	100	1797.87	1797.867		6938	1	8.602	64.3	5	
	Y.PIEHGI	5.2972	0.5241	100	1797.879	1797.867		9296.3	1	8.675	82.1	5	
	R.VSPEEH	3.4709	0.3345	100	1971.064	1970.06		6516.1	2	5.755	44.1	6	
	R.TTGIVL	6.1023	0.4994	100	3202.689	3201.684		8823.4	1	8.27	31.9	8	
	R.TTGIVL	4.2574	0.4759	100	3202.69	3201.684		6353	1	7.79	34.5	16	
	R.CPEALF	4.3613	0.4467	100	1495.79	1494.785		6525.9	1	7.357	75	4	
PF14_0231		27	20.90%	282	32580	10.2	U		Annotation|Plasmodium_falciparum_TIGR|(protein codin				
	K.VPPSIN	2.9111	0.3807	100	1598.878	1598.877		6438.5	9	5.866	50	3	
	V.PPSINQ	3.3659	0.3098	99.9	1502.818	1499.808		5856.9	5	5.94	66.7	9	
	K.KANLVV	3.6605	0.3662	100	2068.211	2068.224		10539.8	1	7.018	47.2	9	
	K.ANLVVI	2.7198	0.356	99.8	1927.111	1926.095		7005	1	6.014	55.9	2	
	K.KEDQEK	3.4945	0.2673	99.9	1514.76	1513.754		8056.1	1	5.198	72.7	3	
	R.KIEEAKI	3.3626	0.3999	100	1629.025	1629.014		4581.5	1	7.11	61.5	1	
PFE0075c		85	20.80%	400	47004	8.5	U		Annotation|Plasmodium_falciparum_Sanger|(protein coc				
	K.HEYFFD	4.7266	0.5	100	1749.979	1749.977		9420.4	1	8.224	73.1	4	
	K.VFIFNEI	3.9116	0.3019	100	1459.795	1459.793		7944.8	1	7.461	85	6	

	R.TFQLEG	5.1043	0.5224	100	2267.143	2266.14		6505.7	1	8.94	66.7	29
	K.ENYYNS	5.3278	0.5748	100	2400.083	2400.082		6065.3	1	9.132	60.5	18
	K.EEFFGT	4.2848	0.4613	100	2221.006	2218.997		5371.1	1	7.743	58.3	28
MAL8P1.142		45	20.80%	265	30872	6.4	U	Annotation|Plasmodium_falciparum_Sanger|(protein coc				
	K.TVMGFS	4.4888	0.2874	100	2352.169	2351.171		8271	2	7.47	47.5	18
	F.SGELAD	4.746	0.4396	100	1815.923	1815.924		9509.3	1	7.745	80	6
	K.EDMYTF	3.104	0.3337	99.9	1913.831	1912.829		5584.9	1	5.811	60.7	7
	K.NRIDPLI	4.3245	0.4329	100	2175.289	2175.274		7218.8	1	7.277	52.8	4
	R.IDPLFNI	5.1716	0.5334	100	1906.133	1905.13		6387	1	9.393	75	10
MAL7P1.94		6	20.80%	192	22642	7.1	U	Annotation|Plasmodium_falciparum_Sanger|(protein coc				
	K.FIEHVT	4.6657	0.428	100	1511.792	1511.797		9196.4	1	7.088	86.4	3
	R.AINLSQ	3.4141	0.3695	100	3340.752	3339.727		6505.8	1	7.094	27.8	3
PFB0915w		48	20.60%	1558	175658	4.2	U	Annotation|Plasmodium_falciparum_TIGR|(protein codin				
	S.AALENT	5.7389	0.3976	100	7740.736	7735.715		11668.3	1	5.883	12.1	2
	K.LNETVF	5.5535	0.3373	100	3533.706	3532.697		9582.4	1	6.479	27.5	9
	K.AFFSEI	3.4572	0.3988	100	1323.673	1323.67		9116.1	1	6.713	85	2
	K.SESDVI	4.5219	0.367	100	2046.039	2045.029		9254.4	1	6.555	55.9	2
	K.EVEKET	4.5532	0.403	100	3665.773	3663.757		8780.5	1	6.754	25	9
	K.ETVSII	4.8823	0.4084	100	3200.566	3199.56		4413.6	1	7.08	27.9	2
	K.NMEEEL	3.7512	0.321	100	2224.079	2223.071		8357.1	1	6.14	41.7	1
	K.LIEETQ	4.7114	0.4698	100	2504.272	2503.268		10517.6	1	8.212	47.5	3
	K.VSDLKD	4.2775	0.4422	100	1525.839	1523.828		7404.6	1	7.604	79.2	3
	K.ELESEI	4.9018	0.3688	100	1737.884	1737.88		8195.2	1	6.423	73.1	3
	K.KEIEKD	3.4226	0.2018	99.9	1302.672	1302.669		3900.9	1	3.954	77.8	3
	K.DHFEKF	4.6899	0.3187	100	2678.294	2677.289		10248.6	1	5.855	39.3	2
	K.FEEEAE	5.2536	0.3757	100	3295.636	3293.624		10737.7	1	6.38	35.2	1
	K.KLEEVH	6.1454	0.4446	100	2704.467	2703.462		7839.1	1	7.531	44.3	3
	K.GLEEDD	3.2097	0.3879	100	2476.204	2476.202		9974.2	1	5.521	42.9	2
	K.KVEEGV	3.0838	0.2781	99.9	1173.684	1173.684		7205.8	1	4.874	75	1
PFL2150c		24	20.60%	335	39693	5.6	U	Annotation|Plasmodium_falciparum_Sanger|(protein coc				
	K.HCLPQC	2.6132	0.32	99.8	1214.637	1214.636		6430.9	1	5.454	66.7	6
	K.KQEPTE	6.4967	0.5159	100	2642.296	2642.287		11214.8	1	9.365	61.9	3
	K.KQEPTE	4.9167	0.3843	100	2642.296	2642.287		8200.9	1	6.76	31	1
	K.NCVNGE	3.7812	0.2121	99.9	1637.752	1637.73		6157.4	1	4.71	69.2	1
	K.NCVNGE	4.667	0.3849	100	4366.039	4365.025		8530.4	1	5.634	20.1	1
	K.REDEVF	4.7602	0.4586	100	2735.296	2732.279		7319.2	1	7.814	50	12

PFD0525w		25	20.60%	326	37065	6	U	Annotation|Plasmodium_falciparum_Sanger|(protein coc				
	K.LLDNSN	5.1122	0.3682	100	3823.103	3820.068		8852	1	5.842	26.5	2
	K.NIDETG	5.2067	0.3862	100	2868.498	2867.484		5738.9	1	7.187	29.2	14
	K.SGYLHT	3.1554	0.3519	100	925.5231	925.522		4339.3	1	6.41	92.9	9
PF13_0014		40	20.60%	194	22481	9.8	U	Annotation|Plasmodium_falciparum_Sanger|(protein coc				
	K.EIAQCLI	3.7346	0.4419	100	2042.053	2042.063		7565.5	1	6.799	61.8	1
	R.TLTSVY	4.7888	0.4356	100	2408.352	2407.323		5598	1	6.997	47.6	22
	T.SVYDSIL	4.674	0.4297	100	2093.163	2092.144		5992.3	1	7.285	50	17
PFE0630c		54	20.30%	281	33006	7.3	U	Annotation|Plasmodium_falciparum_Sanger|(protein coc				
	K.ENEFLC	3.4485	0.2083	99.8	1595.706	1595.701		6389.6	4	5.379	63.6	1
	K.FGEFILK	2.8763	0.2457	99.9	860.5012	860.4995		3610.2	1	5.697	100	1
	K.NVIVGN	5.982	0.5112	100	2027.089	2027.085		9540.6	1	9.788	70.6	19
	K.RVGIPLY	5.0085	0.472	100	2339.312	2339.306		7521.6	1	8.439	52.6	3
	R.VGIPLYS	4.0217	0.437	100	1368.828	1366.817		3487.1	1	7.328	86.4	10
	R.VGIPLYS	5.4017	0.4421	100	2185.214	2183.205		9983.1	1	8.634	66.7	20
PFB0200c		45	20.20%	405	47005	6.8	U	Annotation|Plasmodium_falciparum_TIGR|(protein codin				
	K.EKPYLL	4.2291	0.5068	100	3008.522	3007.513		6163.2	1	8.353	36.5	2
	K.EKPYLL	5.0652	0.4379	100	3009.525	3007.513		7846.5	1	6.601	27.9	5
	R.AGALHI	3.5369	0.3493	100	978.5651	975.5523		6418.5	1	6.148	87.5	10
	K.FYSSPV	3.5003	0.4342	100	1327.69	1327.687		6408.1	1	7.902	75	7
	R.ILCQLL	4.284	0.3276	100	1477.831	1477.827		8392.7	2	6.775	81.8	8
	K.YNLNYD	3.4366	0.3065	100	1619.789	1619.786		9117.6	1	5.775	86.4	4
	R.GLFSFV	3.5699	0.3719	100	1191.716	1191.714		5417.3	1	7.994	85	9
PF11_0065		142	20.20%	282	32354	10	U	Annotation|Plasmodium_falciparum_TIGR|(protein codin				
	R.VNAPS	3.5644	0.3156	100	1298.659	1296.652		6443	1	6.106	80	19
	K.LLESIPL	3.4052	0.4491	100	1392.938	1392.938		3375.5	1	8.712	77.3	18
	R.TDCTFP	4.0046	0.3497	100	1962.01	1961.006		5611.2	1	6.161	71.9	10
	S.NVFVIG	4.075	0.4001	100	1933.059	1933.055		3931.5	1	7.392	53.1	7
	S.NVFVIG	3.9206	0.4356	100	1947.075	1947.089		3793.8	1	6.641	40.6	5
	S.NVFVIG	5.7398	0.4128	100	1950.088	1947.089		6678	1	9.577	71.9	64
	N.VFVIGD	4.4276	0.4353	100	1833.037	1833.046		6315.2	1	7.687	66.7	19
PFC0285c		137	20.10%	532	59068	5.7	U	Annotation|Plasmodium_falciparum_Sanger|(protein coc				
	R.SHQHTV	4.2131	0.4587	100	1628.852	1628.847		7979.1	1	7.529	67.9	9
	K.IHPQIIC	3.071	0.2575	99.8	1376.733	1376.741		3114.1	1	5.275	70	1
	K.VNVHSF	5.1866	0.4731	100	2058.945	2058.937		9260.2	1	9.107	71.9	3
	K.VENIIS	4.7495	0.4568	100	1793.945	1792.943		6994.5	1	7.936	78.6	7

	K.LGYCDK	4.08	0.3751	100	1923.029	1923.016		8205.4	1	7.665	73.3	10
	R.SLHDAL	5.3095	0.537	100	1498.789	1498.794		6406.7	1	8.987	84.6	36
	R.SLHDAL	4.5782	0.3764	100	1883.956	1883.965		4768.5	1	7.212	46.9	18
	R.SLHDAL	5.7392	0.513	100	1883.968	1883.965		8318.9	1	9.561	78.1	42
	K.LSQITS	4.4722	0.4708	100	1775.97	1775.976		7479.8	1	8.927	73.3	11
PFD0240c		36	20.10%	378	43088	8.3	U		Annotation|Plasmodium_falciparum_Sanger|(protein coc			
	N.KIDSLC	3.6802	0.2814	99.9	1675.894	1675.903		5872.2	1	6.73	69.2	3
	K.NYFSHF	3.7575	0.4487	100	1326.683	1326.692		8446.1	1	7.859	77.3	14
	K.NYFSHF	3.5548	0.4294	100	1326.699	1326.692		4881.8	1	8.017	68.2	2
	K.IQGKPG	3.071	0.2988	99.9	1272.733	1272.732		4032.6	1	4.891	68.2	8
	K.IPHFVN	3.7829	0.4123	100	1776.877	1776.874		4614.4	1	6.898	57.7	3
	K.IPHFVN	4.2836	0.4473	100	1791.922	1790.909		3983.3	1	7.61	73.1	5
	K.SNNSQN	3.5876	0.2831	99.9	2734.266	2733.261		5428.7	1	5.139	32.6	1
PF10_0271		22	20.10%	249	29644	6.3	U		Annotation|Plasmodium_falciparum_TIGR|(protein codin			
	R.GRNEIT	4.9974	0.4295	100	1687.883	1687.88		6319.1	1	7.537	73.1	8
	R.ELGENL	6.1202	0.5602	100	4187.926	4184.902		8280.2	1	8.849	28.6	14
PFI0155c		4	20.10%	179	20856	5.1	U		Annotation|Plasmodium_falciparum_Sanger|(protein coc			
	K.ETIVDN	3.4665	0.3117	99.9	2499.183	2498.213		6550.2	1	6.109	45	1
	R.GADCCY	3.6304	0.3738	100	1774.803	1774.815		8329.8	1	7.186	60.7	3
PFA0315w		33	19.90%	156	18395	4.5	U		Annotation|Plasmodium_falciparum_Sanger|(protein coc			
	R.LAVTNN	4.573	0.3949	100	1752.901	1752.899		7982.2	1	7.315	76.7	3
	K.LIEYVH	5.4897	0.4161	100	1785.933	1782.925		7589.8	1	7.649	82.1	30
PF13_0316		28	19.90%	151	17250	10.4	U		Annotation|Plasmodium_falciparum_Sanger|(protein coc			
	K.KGQTPS	3.5375	0.377	100	1363.783	1363.777		4798.9	1	6.932	75	15
	R.AQGIAT	4.0871	0.3413	100	1896.047	1893.037		4336.1	2	5.6	56.2	13
PFD0080c		22	19.80%	560	60270	8.6	U		Annotation|Plasmodium_falciparum_Sanger|(protein coc			
	K.RSPSTA	3.8102	0.2577	99.9	1351.703	1350.698		6702.3	1	5.946	75	3
	R.SPSTAS	2.9996	0.3412	99.9	1194.597	1194.597		5852.9	1	7.051	68.2	3
	R.SASTAS	3.4286	0.3671	100	1599.771	1598.762		5855.1	1	6.972	58.8	3
	R.LPSTGS	2.4349	0.3221	99.8	931.5215	931.5212		4345.3	1	5.327	75	1
	K.EGSNVD	4.3546	0.3703	100	3762.541	3761.537		7031.9	1	5.801	22.7	2
	K.NLDLYD	2.8745	0.2798	99.8	1346.622	1346.623		5482.1	1	5.67	75	1
	K.NLDLYD	3.7412	0.1747	99.8	1477.728	1474.718		7513.9	1	5.295	77.3	2
	K.LIDLED	3.8053	0.4366	100	1798.897	1798.886		9084.3	1	7.49	75	6
	R.KLSDKV	3.1639	0.2638	99.9	1291.696	1290.691		5033.4	2	4.8	75	1
PF13_0165		83	19.60%	596	68010	5.3	U		Annotation|Plasmodium_falciparum_Sanger|(protein coc			

	K.SKDKKE	4.3581	0.3785	100	1467.849	1467.85		8116.3	1	6.072	81.8	4
	K.NENKNE	4.3859	0.3118	100	1932.911	1932.901		3970.9	1	5.944	63.3	3
	K.ESDKVV	3.3218	0.2587	99.8	1586.831	1582.819		4760.2	1	5.024	65.4	2
	K.STHNLE	6.4155	0.4717	100	2214.071	2213.069		9147.4	1	9.137	68.4	15
	K.GEYILT	4.2745	0.3429	100	1423.782	1423.781		8755.7	1	6.619	77.3	5
	K.FNGVPV	4.6222	0.4412	100	1826.929	1825.924		4296.7	1	7.226	73.3	3
	K.FNGVPV	4.709	0.3862	100	1962.039	1961.036		7404.3	2	6.956	56.2	21
	R.NMFEQ	5.2391	0.4687	100	1997.957	1996.952		8315.3	1	8.273	83.3	27
	K.ALEQDG	3.1784	0.2549	99.9	1146.586	1146.583		7447.9	1	5.879	83.3	3
PFF1295w		88	19.60%	372	43650	7.6	U		Annotation|Plasmodium_falciparum_Sanger|(protein coc			
	R.FHTDEI	4.7019	0.417	100	1524.824	1524.821		7789.7	1	8.105	91.7	11
	K.LYTSFI	6.0798	0.5624	100	2715.346	2715.334		9139	1	10.128	43.5	9
	K.LYTSFI	4.8627	0.5064	100	2718.349	2715.334		9642.3	1	8.799	52.2	21
	R.GLKDEQ	3.3146	0.2727	99.9	1345.735	1345.733		7865.6	2	5.133	68.2	3
	K.IPGLTFV	2.9433	0.4083	100	1046.569	1046.568		3339.5	1	6.359	87.5	6
	K.IPGLTF	4.9654	0.5478	100	2580.367	2580.354		8224.8	1	9.268	54.3	6
	K.IPGLTF	6.9358	0.5181	100	2582.36	2580.354		6920.2	1	9.399	43.5	32
PFB0395w		3	19.60%	235	28164	8.7	U		Annotation|Plasmodium_falciparum_TIGR|(protein codin			
	K.NINNFV	3.3742	0.3214	99.9	2761.485	2760.459		6787.8	1	6.393	36.4	1
	K.FPTVST	3.3556	0.3907	100	2610.374	2610.36		6430.6	3	6.656	36.4	2
PF10_0343		153	19.50%	585	62947	4.4	U		Annotation|Plasmodium_falciparum_TIGR|(protein codin			
	K.YEDLEE	8.8644	0.5813	100	3919.556	3918.544		7223	1	10.239	33.6	9
	K.GQEGV	5.1038	0.4784	100	2517.081	2516.077		6735.1	1	7.714	45.5	3
	K.VSNGG	4.9002	0.2302	100	2480.05	2479.046		10937.3	1	6.44	47.8	12
	E.VSNGGE	3.4526	0.2323	99.8	1319.583	1319.583		5164.2	1	6.168	75	60
	R.EDKVS	4.0312	0.2125	99.9	2064.919	2063.912		3593.5	2	6.122	52.8	54
	K.GGAGTI	4.0464	0.4664	100	1785.798	1783.785		6071.1	1	8.063	52.9	15
PF14_0105		45	19.50%	334	39832	8.4	U		Annotation|Plasmodium_falciparum_TIGR|(protein codin			
	K.TNILDQ	4.7279	0.4076	100	2119.025	2119.017		8748.4	1	7.972	71.9	6
	K.HGDNIS	3.3155	0.3823	100	1152.552	1152.551		4969.9	1	7.158	83.3	3
	K.YNNNVF	5.0912	0.4352	100	1625.83	1624.824		5252.3	1	7.319	83.3	18
	K.TYEYVY	3.6586	0.3748	100	1735.834	1735.829		7475.9	1	7.345	65.4	3
	R.NVHFNY	2.7568	0.4131	100	1411.675	1411.676		5796.9	1	7.393	80	15
PFB0620w		9	19.50%	154	17808	6.5	U		Annotation|Plasmodium_falciparum_TIGR|(protein codin			
	K.GTEHF	5.5666	0.3214	100	3659.677	3658.648		8813	1	6.104	26.7	9
PF11_0179		20	19.50%	128	15262	9.7	U		Annotation|Plasmodium_falciparum_TIGR|(protein codin			

	K.INNQNN	3.8428	0.2672	99.9	1591.81	1590.803	5423	1	6.115	79.2	11	
	K.YLVNPL	4.0995	0.2972	100	1464.791	1464.785	6316.2	1	5.823	86.4	9	
PFI1170c		59	19.40%	541	59686	7.7	U	Annotation|Plasmodium_falciparum_Sanger|(protein coc				
	R.VLLFDY	4.2813	0.4252	100	1681.91	1681.917	5838.4	1	8.203	71.4	30	
	R.VLLFDY	3.5026	0.4291	100	1681.918	1681.917	5269.2	1	7.23	64.3	3	
	K.YILIAT	4.2092	0.3519	100	2246.194	2246.19	6088	1	6.485	50	1	
	K.TSELYD	3.9597	0.3743	100	1600.814	1600.822	10605.3	1	8.567	69.2	12	
	K.IIADHL	4.0862	0.5293	100	3474.874	3473.835	6100.6	1	8.867	32.8	6	
	R.VIGFHY	4.7918	0.3879	100	2407.251	2407.267	9783.5	1	6.865	33	1	
	R.VIGFHY	5.0755	0.4581	100	2408.255	2407.267	6679.5	1	8.662	54.5	6	
PF13_0119		72	19.40%	216	24752	8.9	U	Annotation|Plasmodium_falciparum_Sanger|(protein coc				
	K.LAFIET	7.3456	0.5432	100	3320.745	3318.737	6846.9	1	8.829	38.4	32	
	K.LAFIET	4.9952	0.5342	100	3332.779	3332.772	8853.9	1	9.3	35.7	18	
	I.ETSALE	3.1317	0.3371	99.9	2882.487	2881.481	5383.6	1	5.821	33.3	1	
	S.ALEATN	4.4535	0.4299	100	2566.392	2564.359	5251.8	1	7.907	50	16	
	A.LEATNV	3.3423	0.3534	100	2487.352	2486.304	4412.2	1	6.198	37.5	3	
	L.EATNVE	3.6183	0.3366	100	2381.248	2380.238	5349.8	17	6.225	34.2	1	
	K.INVDDD	3.5167	0.2582	99.9	1526.71	1526.705	4500.4	4	5.928	62.5	1	
PF14_0092		31	19.30%	83	9493	5	U	Annotation|Plasmodium_falciparum_TIGR|(protein codin				
	N.SKDREI	4.339	0.3718	100	1785.839	1785.837	6709.1	1	7.532	66.7	12	
	N.SKDREI	4.5454	0.3695	100	1785.84	1785.837	6008.8	1	7.709	55	3	
	K.DREDES	3.7039	0.372	100	1570.706	1570.71	7595	1	6.657	76.9	15	
	K.DREDES	3.4396	0.3233	99.8	1570.712	1570.71	4485	1	6.201	65.4	1	
PF14_0187		95	19.00%	211	24789	6.4	U	Annotation|Plasmodium_falciparum_TIGR|(protein codin				
	K.EKDTPF	5.3678	0.2932	100	3021.64	3021.662	8243.2	1	7.77	32.7	16	
	K.EKDTPF	4.1969	0.4853	100	3052.797	3049.731	5958.6	1	7.976	34.6	22	
	K.DTPFEQ	5.1722	0.2844	100	2767.55	2764.525	11415.7	1	5.405	29.2	3	
	K.DTPFEQ	5.4932	0.576	100	2792.572	2792.594	7447.2	1	9.633	45.8	34	
	K.AHNEFI	4.2686	0.4095	100	1510.823	1510.82	6597.8	1	7.916	83.3	20	
PFA0420w		57	19.00%	179	19734	4	U	Annotation|Plasmodium_falciparum_Sanger|(protein coc				
	Q.AKIPDL	3.596	0.3197	100	1481.793	1478.782	3697.8	1	5.848	75	3	
	Q.AKIPDL	5.0541	0.4234	100	3874.933	3872.923	3452.3	1	6.107	22.7	1	
	K.IPDLVT	7.0048	0.5139	100	3539.69	3538.679	6510.8	1	8.57	35.8	9	
	K.IPDLVT	6.6561	0.4488	100	3668.78	3666.773	6654.2	1	7.134	25.8	6	
	K.IPDLVT	6.2144	0.4538	100	3668.782	3666.773	9506.2	1	7.798	30.6	11	
	K.TNVEET	5.7876	0.4651	100	2286.069	2285.065	5308.7	1	8.363	65.8	6	

	K.TNVEET	4.2485	0.3315	99.9	2414.164	2413.159		5163.7	3	5.564	31.2	3	
	K.TNVEET	4.5494	0.411	100	2414.165	2413.159		5895.1	1	7.492	52.5	15	
	E.NVNNVN	2.8464	0.3116	99.9	1286.648	1286.645		5912.7	1	5.552	65	3	
PFC0270w		19	18.90%	349	41591	5.3	U		Annotation|Plasmodium_falciparum_Sanger|(protein coc				
	K.SNQNIN	3.676	0.3425	100	1658.828	1658.829		5344.3	1	5.752	69.2	1	
	K.WAIEEL	4.871	0.3498	100	1518.817	1518.814		9156	1	7.841	90.9	8	
	K.GHVSIN	6.3756	0.5252	100	2078.993	2077.987		8401.1	1	10.517	77.8	4	
	K.YSYYFI	3.5511	0.405	100	1366.645	1366.643		9179.3	1	7.292	83.3	1	
	K.INQIIDD	4.3015	0.2733	100	1359.83	1359.826		6581	1	5.159	90	5	
PFL0280c		50	18.90%	333	39018	4.9	U		Annotation|Plasmodium_falciparum_Sanger|(protein coc				
	R.TAQELF	3.219	0.3857	100	1167.549	1167.547		5397.7	1	7.3	94.4	6	
	R.LGDINL	4.8707	0.42	100	1901.038	1898.029		8989.9	1	7.303	70	31	
	R.LGDINL	5.75	0.3921	100	2443.274	2440.255		6205.9	1	6.801	44.7	4	
	R.QIEEEG	4.5543	0.4275	100	2076.023	2075.014		3924.7	1	6.476	52.8	7	
	K.ITVSTV	3.1092	0.3971	100	1540.793	1540.797		7167	1	6.984	61.5	2	
PF13_0282		23	18.70%	246	27948	6.1	U		Annotation|Plasmodium_falciparum_Sanger|(protein coc				
	R.YLYNYN	4.3938	0.3391	100	2700.398	2699.394		7804.7	1	6.953	52.4	15	
	K.QSYTQY	4.031	0.3642	100	2678.324	2677.338		6396.9	1	6.897	37	8	
MAL13P1.540		294	18.60%	932	108185	5.7	U		Annotation|Plasmodium_falciparum_Sanger|(protein coc				
	R.NINLNI	4.6667	0.3788	100	2229.149	2226.142		4013.9	1	7.199	58.3	25	
	L.GIINGV	3.1454	0.3201	99.9	2398.347	2397.363		6121.5	1	5.144	34.1	1	
	G.IINGVT	4.1572	0.2749	100	2313.292	2312.273		6717.5	1	6.961	52.4	2	
	T.AAAIHN	4.0534	0.439	100	1729.959	1728.958		6367.7	1	9.007	60	3	
	K.SADVFI	3.7883	0.3228	100	1506.743	1506.755		7975.1	1	6.017	70.8	3	
	R.QDFEEL	6.118	0.342	100	1878.937	1878.935		9650.9	1	7.599	85.7	95	
	R.QDFEEL	5.2964	0.2098	100	1878.937	1878.935		8436.1	1	6.284	55.4	11	
	K.ILNEVT	4.1478	0.2142	99.9	1564.85	1563.842		8190.5	1	5.366	79.2	15	
	K.SKYEHL	3.0276	0.3558	100	1159.613	1159.611		3962.8	1	5.781	83.3	3	
	D.KVLVVY	3.7669	0.2791	100	1234.706	1234.705		6363	1	6.313	88.9	8	
	K.VLVVYE	3.0333	0.4451	100	1106.611	1106.61		4305.8	1	6.673	87.5	12	
	K.VLVVYE	4.304	0.4185	100	1752.893	1750.886		4784.9	1	6.866	66.7	3	
	K.HNIPIEF	3.171	0.2883	99.9	1269.697	1268.694		3506	1	5.136	83.3	9	
	K.HNIPIEF	2.9446	0.3882	99.8	1269.7	1268.694		4480.2	1	6.645	72.2	1	
	R.NEYLNK	6.3802	0.4716	100	3505.649	3504.634		10783.7	1	8.344	38.9	18	
	K.LEEYED	4.6213	0.4243	100	2744.274	2743.263		9129.2	1	7.159	50	9	
	K.LEEYED	4.62	0.3845	100	2745.278	2743.263		4604.1	1	6.191	38.1	3	

	K.IHELQDI	4.6332	0.3757	100	1503.851	1503.847	6713.4	1	6.208	86.4	12
	K.IHELQDI	5.6214	0.4337	100	1788.996	1788.991	10446.8	1	7.661	80.8	10
	K.KLDNYT	3.9523	0.2962	100	1343.702	1343.7	8839.5	1	6.454	80	51
PFL1425w		100	18.50%	542	60663	7.1	U	Annotation|Plasmodium_falciparum_Sanger|(protein coc			
	K.NPGTVL	3.6186	0.5084	100	1414.815	1414.806	7370.9	1	8.058	70.8	6
	R.SQDEEV	4.5258	0.4942	100	3053.545	3051.537	8361.9	1	7.365	21.4	6
	R.SQDEEV	4.7797	0.5072	100	3062.565	3058.554	8792.5	1	9.403	39.3	71
	V.GDGTTS	4.2284	0.2937	100	2371.286	2371.283	5580	1	7.778	47.7	1
	R.QNIHPT	3.11	0.3209	99.9	2453.271	2452.263	4513.9	1	6.079	37.5	4
	K.GVSDLA	3.1936	0.2711	99.9	1316.733	1313.722	7259.1	1	4.806	77.3	8
	R.ISGATIV	3.2484	0.3118	100	945.5745	944.5716	4708.2	1	5.913	87.5	1
	R.KSITEAV	3.9902	0.461	100	1691.032	1691.026	8041.7	1	7.498	70	3
PFL1170w		153	18.40%	875	97230	8.9	U	Annotation|Plasmodium_falciparum_Sanger|(protein coc			
	A.SLYVGD	3.7829	0.4695	100	3354.718	3353.709	5751	1	8.281	29.3	3
	K.KEEATA	6.9049	0.6213	100	2155.993	2153.98	7235.4	1	10.735	76.2	3
	K.KEEATA	5.823	0.4715	100	2499.158	2498.149	6437.2	1	8.623	37.5	3
	K.KEEATA	6.07	0.5435	100	2502.173	2498.149	6138.7	1	9.462	58.3	9
	K.KEEATA	6.9096	0.4393	100	2856.308	2855.314	9316.8	1	7.677	40.7	6
	K.KEEATA	4.6283	0.4828	100	2856.325	2855.314	7662.9	1	8.062	46.3	9
	K.KEEATA	5.455	0.4078	100	3212.499	3211.495	5807.7	9	7.164	22.5	22
	K.KEEATA	3.9581	0.437	100	3212.505	3211.495	6462	2	6.92	26.7	9
	K.EEATAS	4.876	0.4729	100	2371.065	2370.054	4883.6	1	8.508	47.8	3
	K.AKFDNL	3.5824	0.2895	100	1291.667	1291.665	6519.3	1	5.948	85	3
	K.NLDDGI	3.5959	0.4831	100	1389.665	1389.669	6946.5	1	8.131	81.8	4
	R.ELFEPF	3.743	0.3538	100	1446.764	1446.759	3551.9	1	6.298	79.2	8
	K.GFGFVC	5.0676	0.4083	100	1690.748	1690.754	7393.2	1	9.561	82.1	27
	K.GFGFVC	2.78	0.4127	99.8	1690.765	1690.754	5420.5	1	6.926	57.1	1
	K.IINGKP	3.7294	0.3206	100	1514.886	1514.895	4576	1	5.69	48.1	2
	K.IINGKP	4.6677	0.4873	100	1528.922	1528.929	4001.1	1	7.648	88.5	35
	K.QQNAA	3.5795	0.3297	100	2706.269	2705.256	4179	1	6.503	37.5	3
	V.ANYHPT	3.1306	0.2959	99.9	1071.561	1071.559	4613.8	1	5.477	72.2	3
PFB0525w		82	18.40%	610	70498	6.7	U	Annotation|Plasmodium_falciparum_TIGR|(protein codin			
	Q.KAQEFC	4.6478	0.42	100	1712.899	1712.893	6200.9	1	7.531	65.4	1
	K.KLKPIT	3.7469	0.3462	100	2034.238	2034.221	7497.7	1	5.401	52.9	5
	K.LKPITE	4.2302	0.3474	100	1913.146	1913.144	7267.3	1	7.327	53.1	2
	K.LKPITE	4.4264	0.4258	100	1913.146	1913.144	7029.3	1	7.644	59.4	22

	K.LKPITE	4.5503	0.4889	100	2391.375	2391.386	5246.4	1	8.173	52.5	4	
	K.YCIDYV	4.2209	0.4597	100	3464.652	3463.646	6578.5	1	8.503	36.5	17	
	K.ITYTNA	2.9664	0.3344	99.9	2370.216	2369.218	4622	3	5.379	34.2	1	
	K.KPVIVY	3.7508	0.3954	100	1228.727	1227.721	4219.4	1	6.738	88.9	6	
	R.LIMLVT	5.3463	0.5822	100	2163.238	2163.251	10701	1	10.034	69.4	24	
PFC0910w		66	18.40%	429	50151	5.5	U	Annotation|Plasmodium_falciparum_Sanger|(protein coc				
	T.NHTEHA	5.9738	0.4439	100	1954.974	1954.967	6197.9	1	8.528	76.7	2	
	K.KIDVNNI	5.129	0.4945	100	2389.328	2389.324	8546.7	1	8.707	60.5	3	
	K.IDVNNN	5.1938	0.3946	100	2261.234	2261.229	6359.7	1	7.741	61.1	20	
	K.NNNIHT	6.2131	0.4786	100	1838.967	1837.96	5839.1	1	8.986	80	11	
	K.SKNDIM	3.4735	0.1951	99.9	1271.612	1271.617	7034.8	2	5.45	77.8	3	
	K.INFLNE	5.4239	0.4576	100	2111.098	2111.094	8089.3	1	8.612	75	25	
	K.INFLNE	4.4919	0.3498	100	2111.099	2111.094	4325.4	1	6.368	46.9	2	
PF11_0055		84	18.40%	424	49266	9.1	U	Annotation|Plasmodium_falciparum_TIGR|(protein codin				
	A.YNTSSL	4.4516	0.3579	100	3220.708	3219.684	9356.2	1	6.04	27.8	3	
	T.SSLLET	4.7873	0.3551	100	2827.511	2827.495	7178.6	1	6.594	33.3	3	
	T.SSLLET	4.8468	0.5567	100	2841.547	2841.53	6531.9	1	9.166	43.8	24	
	K.HDLQIV	5.1099	0.5249	100	1963.012	1963.011	6955.7	1	9.257	75	7	
	K.FVFKDE	3.0924	0.3201	99.9	1569.817	1569.816	6695.9	1	5.903	62.5	2	
	K.FVFKDE	4.4541	0.4417	100	1697.919	1697.911	8075.6	1	7.147	80.8	16	
	K.KPNYVL	4.5255	0.4888	100	1708.9	1708.902	6495.3	1	8.381	78.6	16	
	K.LSHGDI	2.7906	0.2584	99.8	1083.532	1083.537	3471.2	1	5.61	75	1	
	K.KYPSVV	3.6051	0.3072	100	1440.788	1440.789	5094.4	1	6.551	86.4	12	
PF10_0104		5	18.40%	223	25678	7	U	Annotation|Plasmodium_falciparum_TIGR|(protein codin				
	K.WGTGS(	2.9311	0.3947	100	1734.886	1733.882	5015.8	1	6.319	56.2	3	
	K.SYEFNQ	4.5223	0.3334	100	3104.333	3104.353	8419.5	1	6.527	29.3	2	
PF14_0138		6	18.40%	206	23847	5.7	U	Annotation|Plasmodium_falciparum_TIGR|(protein codin				
	K.QFNSII	5.7147	0.393	100	4126.063	4124.079	10423.1	1	7.125	25.7	6	
PFE1020w		1	18.40%	103	12056	8.8	U	Annotation|Plasmodium_falciparum_Sanger|(protein coc				
	K.NDLQIS	4.4795	0.3144	100	2171.175	2170.169	5048.2	1	6.532	52.8	1	
PF14_0060		23	18.30%	475	56317	5.5	U	Annotation|Plasmodium_falciparum_TIGR|(protein codin				
	K.DNQVIV	7.2005	0.5197	100	2547.313	2546.304	7508.1	1	8.61	42	4	
	K.GNKEDL	4.6576	0.2751	100	2124.181	2122.174	7769.4	3	6.051	36.8	1	
	K.GNKEDL	6.7406	0.4666	100	2124.183	2122.174	9817.6	1	9.906	79.4	6	
	K.GNKEDL	5.5535	0.5437	100	2528.361	2528.359	7503.3	1	8.364	45	4	
	K.EDLGLY	4.27	0.2865	100	1823.018	1823.014	8656.1	1	7.337	64.3	1	

	K.IHKQEN	5.5642	0.459	100	2763.271	2762.26	4012.2	1	7.487	39.3	4
	K.NSIVVHS	5.8217	0.4425	100	2372.341	2372.331	6162.1	1	9.389	60	3
PF14_0192		33	18.20%	500	56492	7.9	U	Annotation|Plasmodium_falciparum_TIGR|(protein codin			
	R.LGGTCV	3.4606	0.4214	100	1360.668	1360.672	8159.4	1	6.983	70.8	1
	K.FSFNLP	3.4275	0.3065	100	1334.753	1334.747	4360.1	1	7.015	75	7
	K.DKVDLY	4.8437	0.4812	100	1943.951	1943.935	6050.9	1	8.47	81.2	3
	R.KFDESV	3.7257	0.3666	100	1780.884	1780.879	8797.2	1	6.92	64.3	2
	R.IYEHFD	4.8769	0.561	100	1821.884	1821.893	9317.1	1	10.133	80.8	8
	K.GLHIIG	5.5072	0.5168	100	2199.268	2199.28	9346.1	1	9.844	70	12
MAL8P1.156		94	18.10%	1011	117662	7.1	U	Annotation|Plasmodium_falciparum_Sanger|(protein coc			
	K.GKDGLV	3.1142	0.3166	99.9	1239.651	1238.646	7118.4	1	6.122	85	3
	M.KNIVQD	2.9546	0.3618	99.9	1585.937	1585.933	2653.8	1	6.41	70.8	1
	K.NIVQDN	3.6649	0.3712	100	1329.744	1329.743	8077.3	1	5.861	70	7
	K.NIVQDN	4.8423	0.4418	100	1457.839	1457.838	8607.8	1	6.846	77.3	8
	K.EIDHLEY	2.6308	0.2979	99.8	1187.607	1187.606	3435	1	5.455	93.8	2
	R.EYMDE	6.0528	0.5189	100	3511.352	3510.341	9042.2	1	9.022	31.2	3
	K.YAELWI	4.0953	0.3382	100	1352.674	1352.671	6818.7	1	6.131	80	10
	K.SPNLVV	3.0499	0.2329	99.9	919.5268	919.5253	5925.3	1	6.02	92.9	3
	K.EKEDMN	5.2173	0.237	99.9	3489.657	3487.647	8096.2	2	4.603	33.3	10
	R.GHQLN	4.1599	0.5213	100	1666.739	1666.736	5306.2	1	8.45	73.1	24
	R.GNGLSY	4.015	0.3589	100	2358.131	2358.129	7320	1	7.855	60	5
	K.FLELNIN	4.2485	0.2849	100	1500.878	1500.879	7941.2	1	5.862	87.5	6
	K.ECATGC	4.8937	0.4352	100	2252.929	2251.924	7729.4	1	7.788	66.7	3
	K.AFYEHM	2.7057	0.3511	100	1088.488	1088.488	4331.8	1	6.525	85.7	9
PFI1695c		4	18.10%	193	22295	9.2	U	Annotation|Plasmodium_falciparum_Sanger|(protein coc			
	K.SNLIYT	3.6659	0.322	100	2088.14	2087.139	6585.9	1	5.761	50	2
	K.SNLIYT	4.8292	0.3383	100	4143.19	4141.165	5619.7	1	5.487	21.3	2
PF14_0240		28	18.00%	161	18795	10	U	Annotation|Plasmodium_falciparum_TIGR|(protein codin			
	K.LNYGDY	4.6646	0.5079	100	2025.939	2024.935	7775.4	1	7.815	65.6	27
	R.KTEGPK	2.9245	0.3176	99.9	1277.729	1277.737	8497.3	1	6.008	68.2	1
PFE0790c		8	17.90%	84	9598	7.8	U	Annotation|Plasmodium_falciparum_Sanger|(protein coc			
	K.LSSALK	4.5335	0.2779	100	1661.96	1660.953	5028.1	1	5.613	75	8
PF10_0153		22	17.80%	580	62551	7.1	U	Annotation|Plasmodium_falciparum_TIGR|(protein codin			
	K.DVTTTE	4.2694	0.3735	100	2111	2111.014	7534.1	1	7.034	52.6	6
	K.NIGQLI	3.3229	0.3366	100	1345.766	1345.77	8289.5	1	5.991	77.3	1
	Q.NQSSLL	5.0542	0.4159	100	2529.342	2528.346	10095.9	1	7.746	54.3	4

	K.ALIHDI	3.298	0.4367	100	1353.78	1353.775		5998.3	1	8.108	70.8	2
	K.VGGISE	3.6959	0.3776	100	1657.9	1657.895		5968.1	1	6.934	53.6	3
	K.QIAENA	4.161	0.4813	100	1921.038	1921.033		6113.7	1	7.64	55.6	6
MAL13P1.270		11	17.80%	241	27228	6.5	U	Annotation|Plasmodium_falciparum_Sanger|(protein coc				
	R.AITVFS	4.2417	0.445	100	2375.271	2374.277		8500.2	1	7.512	42.9	7
	K.DLTFIE	4.4056	0.4397	100	2573.281	2572.275		8926.6	1	7.82	57.5	4
PFB0335c		69	17.70%	893	103984	6.5	U	Annotation|Plasmodium_falciparum_TIGR|(protein codin				
	K.LERPFT	4.6215	0.4664	100	1860.994	1860.993		8886.7	1	8.104	66.7	19
	K.LIESIDN	3.7778	0.2881	100	1265.765	1265.762		4743.1	1	5.486	85	1
	K.KANKPF	3.2835	0.3807	100	1168.696	1168.68		7023.2	1	5.807	77.8	1
	K.DLINLE	3.9898	0.4101	100	1632.827	1632.824		9091.9	1	7.257	75	1
	K.KVDTSG	4.7823	0.3308	100	2852.381	2851.371		9422.6	1	6.99	35.4	1
	K.SHSDEN	3.1708	0.3636	100	1427.714	1427.713		6799.4	1	6.849	70.8	2
	K.LHFETIF	2.6613	0.2794	99.9	923.5273	922.5224		3131.8	1	5.658	91.7	2
	K.KFLPLE	5.5335	0.5212	100	2524.19	2523.187		5129.5	1	9.201	64.3	21
	K.FLPLES	4.263	0.4046	100	2396.097	2395.092		5951.6	1	7.436	60	3
	K.LPNSWT	2.5553	0.3612	99.8	1531.757	1531.755		7383.9	23	6.675	45.8	3
	K.GFISHE	3.2649	0.4264	100	1322.653	1322.649		6888.9	1	7.54	85	3
	R.TPDHAA	5.7462	0.5178	100	1881.98	1881.976		8064.9	1	9.889	75	12
PF14_0122		126	17.70%	124	14171	6.8	U	Annotation|Plasmodium_falciparum_TIGR|(protein codin				
	N.PQFEEI	3.2597	0.436	100	2687.317	2686.305		6990.8	1	6.121	31	2
	K.EFVNHY	4.9559	0.4111	100	1757.831	1757.84		7042.2	1	8.846	73.1	101
	K.EFVNHY	4.5092	0.4351	100	1757.848	1757.84		3329	1	8.078	61.5	23
MAL8P1.128		49	17.60%	284	32427	6.2	U	Annotation|Plasmodium_falciparum_Sanger|(protein coc				
	M.VRPSQS	2.7771	0.3102	99.9	1238.596	1238.595		5365	1	6.549	77.8	3
	R.HLTIFS	6.2487	0.5952	100	2244.206	2243.201		7975.4	1	10.167	69.4	31
	R.SEASEF	4.7589	0.4507	100	2426.068	2424.078		6796.4	1	8.181	57.5	15
PF14_0520		124	17.50%	468	52994	7	U	Annotation|Plasmodium_falciparum_TIGR|(protein codin				
	K.CSAQVC	3.1605	0.3937	100	2509.161	2509.157		6396.9	2	6.376	34.8	1
	K.KDDLTN	6.576	0.3548	100	2223.146	2222.138		10812.4	1	7.745	76.3	110
	K.DDLTNN	4.2347	0.4577	100	2108.075	2108.077		5393.7	1	7.952	58.3	4
	K.DNILID	4.1749	0.3605	100	3141.425	3140.419		4953.8	1	6.892	40	7
	K.IISYTQG	3.3748	0.3784	100	1409.861	1409.86		6841.1	1	6.547	72.7	2
PF14_0076		53	17.50%	452	51461	7.2	U	Annotation|Plasmodium_falciparum_TIGR|(protein codin				
	K.FAFIFD	5.3575	0.4665	100	2820.361	2818.352		4836.7	1	7.985	45.8	2
	K.ATAIVD	4.3837	0.4544	100	2125.11	2123.087		3701.6	1	5.983	55	2

	K.IPFLPLY	3.4661	0.3194	99.8	1804.994	1804.986		5795.3	1	6.365	53.6	1
	K.IPFLPLY	4.5161	0.3545	100	1806.992	1804.986		4555.1	1	7.022	75	41
	I.PFLPLYI	3.8806	0.2385	99.9	1684.883	1684.885		6378.8	1	5.105	76.9	1
	K.YFTVFD	4.1996	0.4604	100	2109.021	2108.013		5569.6	1	7.505	58.8	6
PFD0830w		115	17.40%	608	71737	7.2	U		Annotation|Plasmodium_falciparum_Sanger|(protein coc			
	K.KNEVFN	3.4869	0.2495	99.9	1431.704	1431.702		6611.8	1	5.959	90	2
	K.KLQNVV	3.2644	0.2683	99.9	1143.675	1143.667		5329.1	1	6.483	83.3	9
	K.NNDMP	3.3839	0.3746	100	2075.901	2075.895		5189.8	1	6.903	46.9	3
	K.NSIHPN	4.9265	0.4361	100	1803.925	1803.922		4618.1	1	8.154	71.4	25
	K.NSIHPN	4.3418	0.3383	100	1805.929	1803.922		5099.3	1	7.586	64.3	2
	K.YHPEYQ	4.6847	0.4949	100	2541.246	2540.225		8093.2	1	8.729	55.3	11
	K.FDLSQY	4.8368	0.4283	100	1575.843	1572.831		7091.7	1	7.522	87.5	42
	K.NIEDFT	4.2973	0.5281	100	2471.199	2471.214		7547.6	1	8	50	21
PFE0865c		93	17.40%	270	31389	10.9	U		Annotation|Plasmodium_falciparum_Sanger|(protein coc			
	R.IYVGNL	3.8863	0.336	100	1428.761	1428.76		4334.6	1	7.027	75	54
	R.IYVGNL	2.925	0.3902	99.8	1428.763	1428.76		3931.5	1	6.827	54.2	1
	Y.VGNLPS	2.9056	0.3073	99.8	1152.612	1152.613		4333.2	1	5.401	80	2
	R.YVVEVT	6.1125	0.4625	100	1999.07	1998.066		4040	1	7.913	82.4	22
	R.YVVEVT	3.9906	0.4254	100	2513.325	2512.32		4147.1	1	7.35	50	2
	R.YVVEVT	4.8083	0.3342	100	2520.344	2519.337		6574.6	1	6.077	32.1	4
	R.EAGECC	3.2707	0.374	100	1321.577	1319.569		3545.3	1	6.156	72.7	8
PFC0775w		42	17.40%	161	18760	10.2	U		Annotation|Plasmodium_falciparum_Sanger|(protein coc			
	K.NIPCHC	3.3317	0.367	100	1640.71	1640.71		3967.1	1	6.397	62.5	25
	I.PCHCSP	3.1122	0.3492	100	1409.564	1406.566		6513.5	1	7.281	75	13
	K.EGDIVT	3.4487	0.4045	100	1665.872	1665.871		7626.7	1	7.049	53.6	4
PF10_0281		21	17.30%	498	58085	4.8	U		Annotation|Plasmodium_falciparum_TIGR|(protein codin			
	K.WGEWS	2.8894	0.3234	99.9	1409.592	1409.591		8762	1	6.397	80	3
	R.KVLNCF	3.1929	0.2094	99.9	1125.657	1125.657		4586.2	2	5.27	81.2	1
	K.VKNNDI	4.5369	0.3814	100	2225.032	2225.03		5466.4	1	7.803	55.3	3
	K.NNDIHN	4.2407	0.4811	100	1997.868	1997.866		6779.6	1	8.078	61.8	3
	R.KSDEDL	4.4155	0.4476	100	1805.853	1805.841		9089.5	1	8.124	73.3	2
	K.NYDNHD	3.9905	0.4239	100	2226.93	2226.926		5914.4	9	6.398	36.8	3
	K.NYDNHD	4.6891	0.3912	100	2227.934	2226.926		5339.9	1	8.109	58.8	3
	K.KPMDSI	3.8366	0.3522	100	1433.733	1433.731		4482.9	1	6.679	77.3	3
PFB0445c		39	17.30%	457	52224	6	U		Annotation|Plasmodium_falciparum_TIGR|(protein codin			
	K.DFFLKP	2.8254	0.3055	99.9	1277.726	1277.726		4342.4	1	5.43	77.8	3

	K.EDNIPH	4.2073	0.4564	100	1559.891	1559.892		5528.4	1	7.503	69.2	8
	K.FLQNPV	4.8427	0.4219	100	1778.904	1777.901		7661.9	1	7.633	75	14
	K.LLTECN	3.9758	0.3409	100	2426.233	2426.232		6938.5	1	6.74	52.5	4
	C.NFPSIA	3.1505	0.3116	99.9	1809.946	1809.944		5642	2	5.11	50	1
	A.VTFVSS	5.1913	0.4776	100	2137.066	2137.078		6218.4	1	8.329	66.7	6
	A.VTFVSS	4.3059	0.2637	99.9	2138.065	2137.078		9183.8	1	4.824	37.5	3
PFE1285w		40	17.30%	300	34341	7	U		Annotation|Plasmodium_falciparum_Sanger|(protein coc			
	R.IVEQIRF	4.0084	0.4291	100	1215.809	1215.809		8077.8	1	7.93	88.9	32
	R.ESIMEV	5.3126	0.3916	100	2120.06	2118.051		3914.7	1	7.43	67.6	2
	K.EVEDKE	4.7466	0.1629	99.9	1684.868	1684.865		5076.1	1	4.74	76.9	5
	K.SKDSGF	2.7464	0.3412	99.9	1100.577	1100.581		4814	1	5.575	72.2	1
		8	17.10%	76	8565	7.2	U		no description			
	K.IQDKEG	3.7747	0.4579	100	1523.778	1523.782		3738.3	1	7.333	83.3	8
PFF0870w		42	17.00%	795	92755	6.8	U		Annotation|Plasmodium_falciparum_Sanger|(protein coc			
	K.IQNYND	6.6083	0.4551	100	3193.431	3191.423		7990.1	1	7.883	41	3
	K.LINKDNI	4.3689	0.3432	100	1480.796	1480.795		8138.9	1	6.403	86.4	3
	E.DEEEDM	4.8013	0.4422	100	2965.181	2964.172		7785.9	1	7.933	45.7	3
	E.DEEEDM	6.4662	0.4205	100	2966.154	2964.172		7447.5	1	7.139	44.6	3
	K.NKNENF	4.498	0.1998	99.9	1886.909	1886.904		5214.3	1	5.579	75	9
	K.VSENNG	4.9199	0.3547	100	3541.865	3541.842		8675.2	1	6.583	22.6	6
	K.NPGDVC	3.0541	0.4899	100	1257.634	1257.633		3615.1	1	8.605	77.3	1
	R.KYIFGP	4.6079	0.4576	100	1746.9	1745.894		8008.6	1	7.891	84.6	14
PF10_0390		11	17.00%	188	22272	9.7	U		Annotation|Plasmodium_falciparum_TIGR|(protein codin			
PFB0985c		11	14.00%	229	27063	9.6	U		Annotation|Plasmodium_falciparum_TIGR|(protein codin			
PFF1525c		11	13.80%	232	27282	9.6	U		Annotation|Plasmodium_falciparum_Sanger|(protein coc			
PF11_0025		11	13.90%	231	27194	9.6	U		Annotation|Plasmodium_falciparum_TIGR|(protein codin			
	K.NLDPQI	3.6088	0.3577	100	2459.248	2459.234		5741.1	1	6.008	40.5	6
	K.TKLNTF	3.1116	0.244	99.9	1240.67	1240.669		5513	1	5.693	83.3	5
PF14_0167		5	17.00%	147	16653	5.1	U		Annotation|Plasmodium_falciparum_TIGR|(protein codin			
	R.TVGEIK	3.2711	0.404	100	1445.791	1445.79		6590.8	1	6.351	62.5	1
	K.NKVEQV	4.3515	0.3252	100	1452.757	1452.759		8961	1	6.545	81.8	4
PFI0965w		24	16.90%	242	28395	8.1	U		Annotation|Plasmodium_falciparum_Sanger|(protein coc			
	K.YVGKEE	4.6942	0.2774	100	1895.95	1895.962		7322.3	1	6.321	70	8
	K.YVGKEE	3.8163	0.2947	99.9	1895.958	1895.962		4404.1	1	5.961	43.3	2
	K.KATLLNI	3.2323	0.2682	99.9	1177.702	1177.695		6215.8	1	5.791	77.8	13
	R.KLLSNQ	4.1526	0.3953	100	1815.037	1815.024		5661.6	1	7.036	71.4	1

PF14_0578		4	16.90%	148	17117	6.2	U		Annotation|Plasmodium_falciparum_TIGR|(protein codin				
	K.FNDINCI	2.903	0.252	99.8	1315.683	1315.677		7037	1	5.551	66.7	1	
	R.NSCETL	3.799	0.3639	100	1777.867	1777.864		8311	1	6.691	64.3	3	
PF10_0340		97	16.80%	889	104168	7.6	U		Annotation|Plasmodium_falciparum_TIGR|(protein codin				
	K.KPLLIY	4.4809	0.4513	100	1775.958	1775.966		5983.5	1	8.114	75	9	
	K.KPFDEN	4.8916	0.432	100	2745.355	2745.345		9321.4	1	8.498	52.4	37	
	K.NIIESLD	4.9727	0.3255	100	1692.915	1691.911		10043.7	1	6.566	84.6	33	
	R.LKEPLA	3.2154	0.3296	100	1388.724	1388.721		5076.7	1	5.949	81.8	3	
	R.FDVDNL	4.5069	0.4352	100	2733.274	2731.278		3353.1	1	7.756	50	2	
	I.PAFYPEY	4.2592	0.4178	100	2625.427	2625.424		7101.6	1	7.12	37.5	3	
	K.KVIADL	3.4451	0.2718	99.9	2926.56	2925.573		8807.9	1	5.4	34	6	
	K.VIADLS	3.636	0.3095	100	2953.672	2953.641		4991.8	1	5.895	30	1	
	L.SPWLNN	3.4042	0.3123	99.9	2436.341	2435.324		6487.2	3	5.612	40	1	
	S.PWLNNL	5.8996	0.5139	100	2349.296	2348.292		9145.7	1	8.711	68.4	1	
	T.INNITVN	2.7249	0.3461	99.8	1601.997	1601.987		5947.3	3	6.077	42.3	1	
PFL0050c		97	16.70%	653	77116	4.5	U		Annotation|Plasmodium_falciparum_Sanger|(protein coc				
	K.IDNELD	5.7222	0.4257	100	3500.569	3499.557		8589.2	1	6.461	28.6	17	
	K.IDNELD	5.419	0.3381	100	3501.562	3499.557		5120.1	1	5.174	26.2	4	
	K.YFQNFV	5.025	0.4127	100	2148.065	2145.054		8206.4	1	7.328	59.4	36	
	K.YFQNFV	4.1563	0.2825	100	2148.067	2145.054		4625.7	1	6.481	45.3	13	
	R.EFETLIN	2.7379	0.3076	99.9	1207.633	1207.632		4461.5	1	6.326	83.3	2	
	K.ALTNYA	2.748	0.3477	99.9	1345.646	1345.646		8667.2	1	7.176	75	3	
	K.NLDEIE	4.9922	0.3765	100	2745.192	2744.139		9621.9	1	7.251	54.8	3	
	K.NLDEIE	5.4398	0.4134	100	3111.309	3109.298		5660.2	1	7.067	36.5	4	
	K.NLDEIE	4.1004	0.342	100	3119.328	3116.315		8122.5	1	6.952	41.7	5	
	K.EANEIV	5.3195	0.4968	100	1954.995	1953.988		9512.9	1	9.653	78.1	10	
PF08_0087		111	16.70%	545	61159	5.3	U		Annotation|Plasmodium_falciparum_Sanger|(protein coc				
	K.GPPIQE	5.1565	0.38	100	2299.282	2298.275		6014.5	1	6.518	40	8	
	K.GPPIQE	6.4198	0.5229	100	2322.339	2319.326		7235.8	1	9.18	65	34	
	K.VVIDNN	3.8225	0.3201	100	1447.83	1445.823		3765.5	1	6.091	62.5	12	
	K.EDVCEQ	4.3296	0.4966	100	2458.094	2457.089		5620.1	1	8.777	57.1	11	
	L.SHCSFL	2.8056	0.4541	100	1515.776	1515.774		5781.1	1	6.77	66.7	3	
	K.LRDNLP	6.3462	0.3824	100	2684.303	2683.294		9840.4	1	7.949	39.3	15	
	K.LRDNLP	5.3541	0.4853	100	2684.303	2683.294		7294.7	1	8.52	57.1	18	
	R.DNLPTN	3.8161	0.3363	100	2415.111	2414.109		7249.7	1	6.416	52.6	10	
PF11_0266		9	16.70%	126	14337	10.2	U		Annotation|Plasmodium_falciparum_TIGR|(protein codin				

	R.YFILSD	4.6108	0.4925	100	2368.272	2366.238		4221.2	1	7.329	52.5	9
PFL1245w		146	16.60%	1140	131778	5.8	U		Annotation|Plasmodium_falciparum_Sanger|(protein coc			
	K.ELQELN	4.6074	0.36	100	1939.96	1939.955		9403.1	1	7.333	64.3	2
	K.KSYAVE	3.207	0.2981	99.9	1193.69	1193.689		6879.2	1	4.896	83.3	11
	K.SHIAPV	5.8	0.5277	100	2100.193	2098.189		6140.5	1	10.808	73.7	7
	K.KNNNMI	3.9346	0.3468	100	1542.76	1542.77		7461.3	1	6.635	83.3	6
	K.NNNMHY	3.725	0.1747	99.8	1414.676	1414.675		5017.4	1	5.086	72.7	1
	K.VGAENE	4.4467	0.4241	100	1956.927	1956.921		9741.9	1	7.884	70	3
	R.DIFQGL	3.2812	0.3434	99.8	1755.934	1755.932		4853.6	1	6.761	53.6	1
	K.IQEEGN	5.8608	0.4436	100	2441.278	2440.268		10029.1	1	8.339	57.1	70
	K.IQEEGN	5.4886	0.4379	100	2455.311	2454.303		9081.3	1	7.146	39.3	3
	K.SVELFH	4.1017	0.3366	100	3056.575	3056.552		9597.7	1	6.147	31.2	2
	K.SVELFH	4.6713	0.4556	100	3070.608	3070.586		11176.2	1	8.075	45.8	4
	K.KIEVKP	3.1128	0.2239	99.8	1223.739	1221.732		6346.1	2	5.294	77.8	2
	K.IIPALAT	4.9073	0.3586	100	2239.383	2237.374		4692.4	1	6.296	34.5	2
	K.IIPALAT	4.1785	0.5194	100	2272.465	2272.46		6736.6	1	8.001	54.8	8
	I.PALATTT	5.7321	0.6088	100	2032.259	2032.257		7389	1	11.904	60.5	17
	K.NAFINS	4.1481	0.3036	100	2375.271	2374.263		5116.9	1	7.042	52.5	7
PF11_0396		21	16.60%	938	107251	4.5	U		Annotation|Plasmodium_falciparum_TIGR|(protein codin			
	K.GNTQNI	4.4783	0.3287	100	1685.811	1684.804		7685.8	1	6.557	65.4	3
	R.NNGDEY	4.087	0.3197	100	1482.654	1482.653		6652	1	7.88	79.2	3
	G.MSTDHI	3.3861	0.3922	100	1779.855	1779.845		4297.1	1	6.071	64.3	6
	K.ISAFPE	5.48	0.4254	100	4615.245	4613.223		6404.7	1	7.404	22.4	2
	K.FEEIPD	4.8673	0.4057	100	3706.519	3705.512		5035.6	1	6.997	28.2	3
	K.LKSEDQ	3.8595	0.4111	100	2143.046	2143.045		6263.6	1	6.067	55.9	1
	K.SETEED	5.1035	0.3801	100	2845.331	2844.325		8617.7	1	7.247	34.8	3
PFL1385c		15	16.60%	743	86623	4.9	U		Annotation|Plasmodium_falciparum_Sanger|(protein coc			
	K.KAPSDF	3.9819	0.535	100	1875.853	1875.858		4952	1	8.52	70	3
	K.NTLAQS	4.8761	0.4148	100	2262.16	2261.153		8480.4	1	7.248	47.6	2
	K.GLPEPT	4.3538	0.4505	100	2075.063	2075.055		4588.9	1	7.32	61.8	3
	K.ELIENN	3.5269	0.2743	99.9	2002.043	2001.037		4515.5	8	6.136	47.1	3
	K.NIQQIF	5.4073	0.4575	100	3197.557	3197.557		7127.2	1	7.403	38	1
	K.EEEQEE	4.0901	0.4096	100	2786.211	2785.202		5032.9	1	6.697	43.2	3
PFI1570c		53	16.50%	570	65635	7.1	U		Annotation|Plasmodium_falciparum_Sanger|(protein coc			
	K.NFNIDTC	4.7036	0.4169	100	2425.316	2423.309		7464.4	1	8.685	50	13
	R.NNVHHI	4.2605	0.4768	100	1608.8	1608.798		4909	1	7.364	69.2	5

	R.NNVHHI	4.5917	0.3792	100	1615.808	1615.815		7187.6	1	8.091	84.6	12
	K.RDDQM(	3.7163	0.4792	100	1552.627	1552.627		5132	1	7.242	72.7	6
	K.DIVEHI	4.4403	0.3692	100	2361.259	2361.253		6338.7	1	6.819	50	4
	K.NYVTSP	3.6554	0.2434	99.9	1449.825	1449.818		6221	1	6.572	75	12
	R.EIAAVH	3.1876	0.3268	100	1515.871	1515.876		6493.9	1	6.227	66.7	1
MAL7P1.81		29	16.50%	327	37284	6.9	U		Annotation|Plasmodium_falciparum_Sanger|(protein coc			
	R.FVEFNK	4.6293	0.243	100	1593.793	1593.791		6159.1	1	5.766	87.5	6
	K.NHIILA	7.6434	0.5925	100	2277.186	2276.182		4552.6	1	11.174	65.9	17
	A.GGQAAI	3.9943	0.367	100	1601.764	1600.757		6587.1	1	7.206	68.8	3
	K.FLPHGD	3.8716	0.4557	100	1864.866	1864.862		5437.4	1	7.285	64.7	3
PFI0605c		32	16.40%	446	52705	9.1	U		Annotation|Plasmodium_falciparum_Sanger|(protein coc			
	K.LTNFVF	4.5227	0.3314	100	1636.815	1633.805		5835.2	1	6.496	83.3	15
	K.GKVNNN	3.9741	0.2827	100	1442.797	1442.797		5158.2	1	6.068	70.8	2
	K.YLDYLN	3.6385	0.3631	100	1626.815	1626.813		6882.1	1	6.597	70.8	6
	R.FHSSIF	3.2151	0.2929	99.8	1556.699	1556.699		5535.2	1	6.248	62.5	3
	K.TSVNDS	4.5836	0.4371	100	2264.103	2263.121		6759.7	1	8.06	57.5	6
PF10_0346		67	16.40%	371	42276	4.4	U		Annotation|Plasmodium_falciparum_TIGR|(protein codin			
	K.KPDNEI	4.0449	0.2577	99.9	1801.903	1800.898		4944.9	1	5.777	57.1	2
	K.YSSPSD	4.3252	0.4997	100	1750.863	1750.861		6885.4	1	7.531	73.3	6
	K.TLVGLF	3.241	0.2301	99.9	1020.575	1020.573		3951.2	1	5.452	87.5	3
	K.NEIDST	4.4638	0.4806	100	2151.106	2151.106		9255.6	1	8.138	61.8	8
	K.NEIDST	4.2066	0.3078	99.9	2468.233	2466.223		6037.4	1	4.791	37.5	3
	K.NEIDST	5.2218	0.4601	100	2469.231	2466.223		7751.3	1	8.073	47.5	45
PFF0335c		2	16.40%	299	34503	6.1	U		Annotation|Plasmodium_falciparum_Sanger|(protein coc			
	K.ELIQNE	6.5876	0.4984	100	5636.91	5633.885		8476	2	7.712	16	2
PF13_0070		68	16.30%	429	49871	7.5	U		Annotation|Plasmodium_falciparum_Sanger|(protein coc			
	R.GFTYTD	4.3282	0.4394	100	1717.894	1717.88		8697.1	1	7.7	67.9	9
	K.DLNIHT	5.5226	0.4932	100	2738.409	2738.394		3925.8	1	8.071	29	4
	K.DLNIHT	5.1683	0.4851	100	2753.448	2752.428		7143.3	1	8.268	44	5
	R.YGHHST	5.0357	0.3935	100	1850.855	1849.847		6798.7	1	7.238	51.7	3
	R.YGHHST	4.513	0.419	100	1850.856	1849.847		7552.3	1	8.347	66.7	41
	K.NLYSEK	3.509	0.3286	100	1675.804	1675.804		5412.6	1	7.083	66.7	6
PFF0515c		43	16.30%	123	14457	7.4	U		Annotation|Plasmodium_falciparum_Sanger|(protein coc			
	K.IWTEYP	4.4648	0.4669	100	2460.28	2460.285		5734	1	7.737	60.5	43
PFB0260w		160	16.10%	969	108704	6.4	U		Annotation|Plasmodium_falciparum_TIGR|(protein codin			
	K.IKKEEL	3.9864	0.2085	99.9	1523.833	1523.84		9588.9	1	4.935	77.3	1

	K.HDLINY	3.9473	0.3686	100	1515.777	1515.778		8983.4	1	6.093	77.3	4	
	K.LMDVILI	3.1961	0.3706	100	1187.746	1185.747		7517.7	1	8.31	88.9	1	
	K.YTHLNL	5.4695	0.4514	100	2728.421	2727.436		7703.4	1	8.136	52.2	6	
	K.YTHLNL	6.7296	0.5794	100	2742.477	2741.47		7074.3	1	9.327	44.6	5	
	K.DLDVEE	3.738	0.3562	100	1788.894	1788.891		6902.8	1	7.102	64.3	3	
	K.RNTTVW	2.8252	0.3014	99.9	1177.597	1177.597		5065.9	1	6.877	72.2	2	
	K.QNLSST	5.0351	0.5934	100	2018.985	2017.981		6406.6	1	9.627	70.6	39	
	R.EIAEYII	4.0733	0.2349	99.9	1535.809	1535.821		8564.2	1	5.841	83.3	4	
	R.IIDHFL	5.193	0.3788	100	1732.926	1732.921		9223	1	7.297	80.8	37	
	V.PLALAL	4.5571	0.4416	100	1417.846	1417.846		6061.1	1	8.789	87.5	30	
	R.VGQAVI	4.6559	0.348	100	1466.827	1466.833		8669.7	1	7.3	78.6	28	
PF13_0065		194	16.00%	611	68576	5.7	U		Annotation|Plasmodium_falciparum_Sanger|(protein coc				
	K.LNDIVT	4.941	0.4657	100	1953.955	1952.961		7471	1	8.914	70.6	4	
	R.GNEMAI	4.0421	0.4139	100	1901.933	1901.928		8620.1	1	7.82	59.4	11	
	R.TCLVAN	4.0675	0.3551	100	1605.793	1604.789		5785.9	1	6.662	71.4	3	
	R.QLEQYF	4.6382	0.4986	100	2179.024	2178.014		9913.7	1	8.632	65.6	75	
	K.ISDILQ	5.6416	0.5131	100	2248.24	2248.234		10728.7	1	9.626	68.4	100	
	R.IICHFYA	2.6611	0.383	100	1494.755	1494.754		6600	1	6.47	80	1	
PFI1020c		31	15.90%	510	56150	7.9	U		Annotation|Plasmodium_falciparum_Sanger|(protein coc				
	K.TPVISS	3.5368	0.3705	100	1576.817	1576.812		6611.8	1	7.197	64.3	4	
	K.RFENGF	4.9056	0.3668	100	3067.507	3066.496		7527.6	1	6.345	30	4	
	K.KSVLPI	2.9948	0.4438	100	1004.651	1004.658		4753.2	1	7.331	87.5	5	
	K.SAHPDI	5.2877	0.4728	100	2053.142	2053.134		5588	1	9.805	63.2	9	
	R.AQGTA\	3.7068	0.4356	100	1160.609	1160.606		5904.6	1	7.546	85	9	
PF14_0016		7	15.90%	107	11427	9.6	U		Annotation|Plasmodium_falciparum_TIGR|(protein codin				
	K.NKNDKC	3.8886	0.3139	100	1887.943	1887.941		4434	1	6.221	59.4	7	
PF14_0248		15	15.90%	88	10587	8	U		Annotation|Plasmodium_falciparum_TIGR|(protein codin				
	K.CVRPVN	4.6938	0.5053	100	1780.824	1780.822		6326.7	1	8.422	73.1	15	
PF10_0217		167	15.80%	538	62117	10	U		Annotation|Plasmodium_falciparum_TIGR|(protein codin				
	K.SSSCIY	4.1707	0.3313	100	2790.341	2789.335		8077.3	1	6.784	36.5	4	
	K.SSSCIY	6.1246	0.5091	100	2804.375	2803.369		4033.8	1	9.156	50	80	
	R.SSSSSY	4.4735	0.3818	100	1756.817	1753.807		6574.4	1	7.681	71.4	49	
	R.KKDDG\	3.7055	0.1731	99.8	1188.681	1188.681		5920.6	1	6.336	85	5	
	R.VIVTNL	3.6664	0.2825	100	1303.68	1300.668		6679.8	1	5.777	90	8	
	K.VYDDVT	3.6295	0.3687	100	1073.558	1073.559		3447.2	1	6.929	87.5	6	
	K.EKPEVV	3.9194	0.3623	100	1662.856	1659.844		5502.8	1	6.485	76.9	15	

PF13_0224		88	15.80%	184	21746	10.6	U		Annotation|Plasmodium_falciparum_Sanger|(protein coc			
	K.IDNSLN	5.4501	0.387	100	2019.083	2015.072		7241.8	1	7.672	68.8	75
	K.IDNSLN	4.947	0.4865	100	2086.134	2086.109		6346.5	1	8.444	61.8	10
	R.FPALHL	3.1929	0.3485	100	1265.744	1264.742		6668.7	1	6.205	75	3
PF13_0129		31	15.80%	190	21601	9.9	U		Annotation|Plasmodium_falciparum_Sanger|(protein coc			
	K.VVMWF	3.8941	0.3397	100	1757.902	1756.899		4644.5	1	6.586	60.7	14
	H.AHFPIN	4.0927	0.429	100	1725.886	1725.886		5329.2	1	8.467	71.4	12
	H.AHFPIN	4.3656	0.3645	100	1725.892	1725.886		4239.4	1	7.434	60.7	5
PF11_0190		7	15.70%	306	36489	6.4	U		Annotation|Plasmodium_falciparum_TIGR|(protein codin			
	K.LITFDL	3.1048	0.3106	99.9	3172.543	3170.514		7695.6	1	5.516	34	2
	R.SMDYNI	3.3746	0.4188	100	2584.338	2584.331		5110.2	1	6.522	45.2	5
PFC1020c		11	15.60%	262	30047	9.8	U		Annotation|Plasmodium_falciparum_Sanger|(protein coc			
	R.IYEVNL	4.7031	0.4652	100	2100.983	2100.984		7259.1	1	8.512	64.7	2
	K.LSCDHII	3.3192	0.3598	100	1127.566	1127.563		4795	1	6.237	87.5	6
	K.LMELHT	3.2859	0.3624	100	1613.788	1613.785		5327.5	1	6.855	61.5	3
PFL0145c		3	15.50%	97	11331	10	U		Annotation|Plasmodium_falciparum_Sanger|(protein coc			
	K.EKAPFE	3.3383	0.3465	99.9	1817.004	1816.992		4761.8	1	5.474	60.7	3
PF08_0109		25	15.40%	481	55067	4.8	U		Annotation|Plasmodium_falciparum_Sanger|(protein coc			
	K.VSLTND	4.6377	0.4124	100	2027.069	2026.064		4839.1	1	6.581	41.2	6
	K.VSLTND	4.3563	0.4451	100	2048.123	2047.116		7823.1	1	7.671	52.9	9
	R.SLLIAQL	3.565	0.3127	100	1076.717	1076.716		6697.1	1	6.765	83.3	2
	F.IGSPFH	2.8526	0.3806	100	1134.602	1134.602		5661.4	1	6.795	77.8	1
	K.NNFDKN	4.9576	0.2886	100	1798.859	1795.846		4560.7	1	7.266	75	6
	K.IDKEEN	5.7915	0.5058	100	2592.298	2592.288		8048.4	1	8.547	42.5	1
PFL2140c		35	15.40%	332	37288	5.7	U		Annotation|Plasmodium_falciparum_Sanger|(protein coc			
	R.SIVHNV	3.3915	0.3904	100	1905.025	1905.02		4767.6	1	5.606	67.6	1
	K.NQYSNE	4.443	0.4958	100	3717.785	3717.777		8095.4	1	8.807	26.6	25
	K.NQYSNE	6.6286	0.513	100	3746.827	3745.846		10546.6	1	8.488	29.7	9
PFI1625c		22	15.30%	484	55735	6.8	U		Annotation|Plasmodium_falciparum_Sanger|(protein coc			
	K.VATVHT	5.1404	0.4923	100	2391.265	2391.264		6754.3	1	9.021	52.4	13
	K.LAELNF	3.1372	0.2359	99.9	1198.659	1198.658		9682.8	1	5.702	88.9	2
	K.NNSIIH	4.16	0.3495	100	2603.292	2602.284		8339.4	1	5.957	36.9	2
	R.DIAVAA	5.1701	0.5067	100	2145.18	2145.179		8860.1	1	8.604	60.5	5
PF13_0301		39	15.30%	202	22883	5.5	U		Annotation|Plasmodium_falciparum_Sanger|(protein coc			
	K.DTNIFE	3.9282	0.3461	100	1469.754	1468.748		5104.5	1	5.932	81.8	10
	K.IWHPNI	4.4655	0.2784	100	2152.137	2152.123		7516.4	1	5.578	45.8	5

	K.IWHPNI	4.7689	0.5121	100	2176.2	2173.174		5700	1	8.458	77.8	24
PFL2345c		13	15.20%	435	49545	7	U		Annotation|Plasmodium_falciparum_Sanger|(protein coc			
	K.NSSALN	3.9906	0.4828	100	1610.805	1610.81		8068.7	1	7.824	67.9	1
	K.NSSALN	4.0794	0.2676	100	1759.89	1759.894		6430.3	1	6.739	73.3	2
	R.ELCDEI	4.3266	0.4572	100	2456.249	2456.246		5829.1	1	7.735	50	7
	R.HINISHC	3.2921	0.3164	100	1206.588	1206.588		3561.2	1	5.561	83.3	1
	R.TMMELL	4.6178	0.3854	100	2213.069	2212.063		8403.2	1	7.901	66.7	2
PFC0725c		81	15.20%	309	34458	8.5	U		Annotation|Plasmodium_falciparum_Sanger|(protein coc			
	K.AHYSNL	2.8993	0.353	99.9	1243.682	1243.68		4520.5	1	6.604	80	15
	K.NHFFQF	4.4894	0.4639	100	1629.814	1629.814		7680.8	1	8.722	79.2	63
	L.PTLLGN	4.4049	0.3715	100	2531.501	2530.499		8378.1	1	7.488	52.3	3
PFA0440w		5	15.20%	224	25477	4.9	U		Annotation|Plasmodium_falciparum_Sanger|(protein coc			
	R.VDPYEC	3.7718	0.2698	99.9	2476.103	2475.091		3926.1	1	4.791	50	3
	K.TVFNFD	2.9698	0.2955	99.8	1765.723	1764.718		4826.7	4	5.665	57.7	2
MAL8P1.72		19	15.20%	99	11535	10	U		Annotation|Plasmodium_falciparum_Sanger|(protein coc			
	G.KLIGEA	4.1078	0.3738	100	1625.893	1625.902		7450	1	6.752	71.4	4
	K.LIGEAW	4.1328	0.417	100	1497.81	1497.807		7958.9	1	7.499	73.1	15
PF14_0697		4	15.10%	358	41718	7.4	U		Annotation|Plasmodium_falciparum_TIGR|(protein codin			
	K.NYFYIP	3.0676	0.3662	99.9	2078.992	2078.977		2634.7	1	6.558	56.7	1
	R.VLVMPN	3.7798	0.3647	100	2126.099	2125.097		3428.5	3	5.973	44.4	1
	K.SIHIHC	3.3348	0.2915	99.8	2294.083	2293.092		6232.3	1	5.791	47.2	2
PFC0535w		3	15.10%	126	14813	11.1	U		Annotation|Plasmodium_falciparum_Sanger|(protein coc			
	K.VNGEST	4.0307	0.4346	100	2012.08	2012.082		6330.1	1	7.119	41.7	3
PF13_0033		62	15.00%	393	44677	8.9	U		Annotation|Plasmodium_falciparum_Sanger|(protein coc			
	K.RLPCEV	3.3055	0.3369	99.9	2261.136	2261.131		5457.3	1	5.658	44.4	3
	K.RLPCEV	4.9107	0.4407	100	3035.51	3035.501		10087.2	1	6.933	28	1
	R.EVVELP	3.3152	0.3393	99.8	1696.968	1696.964		4073	1	6.393	61.5	1
	R.TLMELL	5.4648	0.4609	100	2172.109	2172.101		9561.4	1	8.081	63.9	57
PFE1340w		18	15.00%	214	24561	9.3	U		Annotation|Plasmodium_falciparum_Sanger|(protein coc			
	K.DGLYHI	4.2209	0.4215	100	2097.946	2097.953		8398.9	1	7.539	53.1	16
	K.KSELSE	3.5576	0.4044	100	1710.009	1709.976		7263.7	1	7.329	71.4	2
MAL7P1.13		39	14.90%	248	29898	5.4	U		Annotation|Plasmodium_falciparum_Sanger|(protein coc			
	R.ETYSEN	4.7188	0.3924	100	2080.019	2078.008		6751.2	1	7.209	68.8	14
	S.ENLVSL	3.5641	0.3405	100	1597.824	1597.823		8244.5	1	6.484	83.3	4
	R.LEREDT	5.0561	0.3901	100	2401.166	2400.16		9983.4	1	6.77	44.7	12
	R.LEREDT	3.6613	0.3994	100	2401.173	2400.16		8012.5	1	6.32	52.6	3

	R.EDTEET	4.2653	0.3384	100	2001.938	2001.933		7444.1	1	7.075	65.6	6
PF14_0377		43	14.90%	241	27687	8	U		Annotation|Plasmodium_falciparum_TIGR|(protein codin			
	K.NIEFPL	5.0502	0.4174	100	1979.119	1976.108		7173.1	1	8.155	78.1	42
	K.LNHSYI	3.9104	0.4703	100	2178.073	2178.073		6052.4	1	7.306	52.8	1
PFC0365w		36	14.80%	532	60754	6.6	U		Annotation|Plasmodium_falciparum_Sanger|(protein coc			
	K.HIINYG	4.2503	0.4956	100	2772.512	2771.507		5632.2	1	8.227	45.7	2
	R.NELSHS	4.4355	0.5197	100	1767.836	1767.83		7349.9	1	8.923	78.6	6
	K.VNSIISH	5.6239	0.5637	100	2246.167	2245.166		5781	1	9.03	52.6	8
	K.DHVTSL	5.0804	0.4771	100	2265.166	2265.163		7427.2	1	8.747	57.9	20
PF11_0162		42	14.80%	492	56666	7	U		Annotation|Plasmodium_falciparum_TIGR|(protein codin			
	R.LHGGVT	4.1667	0.4506	100	1494.777	1494.774		5855.6	1	7.748	80.8	8
	K.ALFLFS	3.6784	0.384	100	1884.947	1884.942		8278.3	1	6.49	70	11
	R.YLGPIS	3.2016	0.4052	100	2106.013	2106.055		6835.5	1	5.871	44.4	2
	R.GGFYD	4.1223	0.3949	100	2491.171	2490.166		7218.7	1	7.602	45.7	20
	R.GGFYD	3.5233	0.3648	99.9	2491.176	2490.166		9901.1	1	5.719	30.4	1
PFE0290c		3	14.80%	149	17947	9.1	U		Annotation|Plasmodium_falciparum_Sanger|(protein coc			
	K.KKCEAA	4.9566	0.4383	100	2667.244	2667.24		7899.7	1	8.456	50	3
PFL1140w		34	14.70%	273	30984	5.4	U		Annotation|Plasmodium_falciparum_Sanger|(protein coc			
	S.KEAHDF	3.4162	0.3095	99.9	1516.72	1516.719		6953.6	2	5.996	50	3
	S.KEAHDF	3.6399	0.4041	100	1516.724	1516.719		4650.2	1	7.125	68.2	2
	S.KEAHDF	4.7578	0.3585	100	1517.726	1516.719		5171.1	1	7.124	90.9	21
	K.EAHDFY	2.7047	0.3427	99.9	1388.625	1388.624		5477	1	6.144	80	3
	K.HGENHI	3.536	0.2919	99.9	1533.742	1533.741		6349.7	3	4.936	50	1
	K.HGENHI	4.4244	0.3533	100	1535.748	1533.741		5097.6	1	5.303	83.3	3
	K.KREEWI	2.8417	0.3874	100	1969.905	1969.904		4626.8	1	5.393	53.6	1
PFF0835w		112	14.60%	446	52981	9.1	U		Annotation|Plasmodium_falciparum_Sanger|(protein coc			
	K.IEKPDF	3.6402	0.2466	99.9	1475.712	1475.713		5091.3	3	4.74	68.2	1
	K.IDCFQS	4.1064	0.3526	100	1881.867	1881.865		7320.9	1	7.055	67.9	2
	K.KLEDID	5.1825	0.335	100	2992.613	2991.612		6183.4	1	5.909	31.2	5
	K.KLEDID	3.6696	0.4354	100	3005.669	3005.646		8643.8	1	7.213	37.5	24
	K.KLEDID	6.92	0.4318	100	3007.65	3005.646		9805.3	1	8.057	44.8	59
	K.LEDIDV	4.5586	0.2787	99.9	2877.565	2877.551		7138.5	1	6.37	30.4	3
	K.LEDIDV	3.0628	0.3619	99.9	2881.571	2877.551		9269.5	1	5.453	34.8	2
	R.EELALI	4.5116	0.3502	100	1649.874	1649.882		8603.4	1	7.023	80.8	4
	K.KLQEEF	4.8656	0.2082	100	1635.889	1635.896		6545.3	1	5.154	54.2	3
	K.KLQEEF	5.32	0.1611	100	1635.89	1635.896		6567.4	1	6.065	87.5	9

PFE0915c		15	14.60%	260	29729	8.2	U		Annotation|Plasmodium_falciparum_Sanger|(protein coc				
	K.RWYPYI	4.7368	0.3934	100	2011.047	2010.045		6726.2	1	7.764	70.6	2	
	R.FFPYYA	4.7316	0.3796	100	2130.047	2129.046		7972.1	1	5.858	73.5	12	
	R.FFPYYA	3.7095	0.4108	100	2286.148	2285.136		7316.8	1	6.739	47.4	1	
PF07_0127		1	14.60%	158	17801	9.3	U		Annotation|Plasmodium_falciparum_Sanger|(protein coc				
	K.VMPLNT	2.858	0.3447	99.9	2507.31	2507.3		6126.6	3	5.054	29.5	1	
PFB0100c		62	14.50%	654	71303	9.1	U		Annotation|Plasmodium_falciparum_TIGR|(protein codin				
	H.HQLQP	3.3431	0.459	100	2493.284	2493.285		7465.9	1	7.613	50	3	
	H.HQLQP	5.0455	0.2555	100	2494.292	2493.285		8789.8	1	5.452	38.6	6	
	K.LKENVV	2.9825	0.2336	99.8	1001.564	1001.563		5326	1	4.907	93.8	1	
	R.KRFPLG	5.659	0.3698	100	2391.21	2390.203		6002.5	1	6.132	62.5	2	
	R.KRFPLG	7.4081	0.395	100	2391.21	2390.203		7424.1	1	7.705	53.8	13	
	R.KRFPLG	6.6157	0.3869	100	2634.332	2633.325		7304.6	1	8.062	48.9	3	
	K.RFPLGM	5.664	0.4472	100	2263.116	2262.108		12229.6	1	7.29	47.4	3	
	K.DKLPGG	3.6843	0.3768	100	1752.85	1752.845		8293.1	1	6.325	60.7	6	
	Y.AYGGS	4.6571	0.4246	100	1498.68	1498.679		6211.5	1	8.449	75	15	
	K.KHKDNE	2.7954	0.3087	99.8	1399.683	1399.682		5005	3	6.389	63.6	6	
	K.HKDNED	2.7108	0.3023	99.8	1271.59	1271.587		5523.6	4	6.03	65	4	
PFC0525c		13	14.50%	440	51616	5.6	U		Annotation|Plasmodium_falciparum_Sanger|(protein coc				
	K.NWPIDE	3.1429	0.3198	99.9	1798.883	1798.88		6059.6	1	5.436	65.4	1	
	K.GTPDEA	3.8095	0.3187	100	1659.902	1659.896		6120.6	1	6.407	67.9	2	
	K.RLNPIE	3.7116	0.3444	99.9	2645.386	2643.379		4503.4	2	5.503	31	1	
	R.LNPIEA	4.3087	0.2437	100	2487.28	2487.278		4402	1	6.561	45	7	
	K.YIDKLP	4.2991	0.4373	100	1694.878	1693.874		3955.7	1	6.992	87.5	2	
PF14_0436		12	14.50%	352	39869	8.8	U		Annotation|Plasmodium_falciparum_TIGR|(protein codin				
	K.NFYKEH	3.7574	0.2855	100	1416.682	1416.687		6617	1	6.263	80	1	
	K.ITILEGE	3.0093	0.3142	99.8	1950.128	1950.128		7115.5	1	5.431	47.1	2	
	K.NNNIVA	3.672	0.4612	100	2359.354	2359.351		6338.3	1	7.684	42.9	9	
MAL13P1.284		19	14.50%	262	28379	8.1	U		Annotation|Plasmodium_falciparum_Sanger|(protein coc				
	R.HCDIIV	6.3678	0.5727	100	2464.345	2464.343		8202.8	1	11.13	69	16	
	K.YVNDIFI	3.9665	0.3868	100	1950.023	1950.017		8354.6	1	7.256	53.3	3	
PF14_0676		41	14.50%	214	25264	8.6	U		Annotation|Plasmodium_falciparum_TIGR|(protein codin				
	K.NVHLYQ	4.5975	0.4055	100	2014.952	2013.949		5836.1	1	7.533	73.3	22	
	R.RNPFEV	5.194	0.4676	100	1802.898	1802.897		8957.8	1	8.938	78.6	19	
PF08_0099		2	14.40%	90	10775	7.9	U		Annotation|Plasmodium_falciparum_Sanger|(protein coc				
	R.YIDIVT	3.5412	0.4111	100	1595.857	1595.847		7126.6	1	7.266	75	2	

PF10_0293		2	14.30%	133	15178	8.9	U		Annotation|Plasmodium_falciparum_TIGR|(protein codin				
	K.YAPGFY	3.2783	0.2978	99.9	2124.127	2123.118		7711	1	4.801	50	2	
PF13_0262		52	14.20%	583	67591	7.4	U		Annotation|Plasmodium_falciparum_Sanger|(protein coc				
	K.DLGNGE	3.5241	0.3181	99.9	1968.012	1966.983		7495.9	1	6.055	55.9	2	
	R.RGDIVG	3.9638	0.3319	100	1328.779	1328.788		4880.3	1	6.613	87.5	15	
	R.RGDIVG	3.8845	0.3922	100	1328.797	1328.788		3783.4	1	7.264	70.8	4	
	K.VTNTIL	4.4743	0.4087	100	2078.033	2078.029		5935.8	1	7.315	64.7	2	
	K.EHKIEL	3.385	0.2886	99.9	1551.862	1551.861		6318.7	1	5.872	65.4	1	
	K.YNDKPF	3.6924	0.3183	100	2260.155	2260.166		6647.9	1	6.516	50	2	
	K.YNDKPF	5.2766	0.4408	100	2374.214	2374.227		5572.8	1	7.414	44.7	12	
	K.YNDKPF	5.5665	0.504	100	2388.278	2388.261		9763.7	1	9.627	60.5	14	
PFI1435w		45	14.20%	190	22005	8.5	U		Annotation|Plasmodium_falciparum_Sanger|(protein coc				
	K.NNTIIV	5.0671	0.5221	100	3020.539	3020.552		9044.3	1	9.453	44.2	43	
	K.NNTIIV	4.8617	0.372	100	3020.574	3020.552		8253.9	1	6.936	30.8	2	
PFL0300c		90	14.10%	304	35686	8.8	U		Annotation|Plasmodium_falciparum_Sanger|(protein coc				
	K.KGEVLY	5.4627	0.4067	100	2025.158	2024.147		6997.2	1	7.684	81.2	67	
	K.VNDILF	4.6589	0.4044	100	1965.067	1965.067		7278	1	8.04	62.5	22	
	K.LVNGHT	2.5017	0.3232	99.8	1044.604	1044.603		5594.3	2	5.705	75	1	
PFI0720w		95	14.00%	516	58421	8.3	U		Annotation|Plasmodium_falciparum_Sanger|(protein coc				
	K.TGAFSE	3.3584	0.4056	100	1012.478	1012.477		4859.5	1	7.186	93.8	3	
	K.YFPGQE	4.4668	0.3657	100	1855.983	1854.975		6575	1	5.965	45.3	4	
	K.YFPGQE	5.7071	0.552	100	1869.011	1869.01		4640.9	1	9.489	75	50	
	F.PGQESL	4.1658	0.4664	100	1558.879	1558.878		6732.3	1	8.408	64.3	7	
	K.NNNNN	4.2862	0.3223	100	2893.274	2892.268		6738.9	1	5.344	29.2	2	
	R.NSLSKP	5.1971	0.4963	100	2405.209	2405.206		4570.5	1	8.142	57.5	15	
	R.NSLSKP	4.7124	0.3778	100	2413.232	2412.223		6789.2	1	7.19	36.2	12	
	S.KPQNNN	4.4896	0.3268	100	2005.023	2004.015		4963.5	1	5.888	62.5	2	
PFA0555c		17	14.00%	371	44245	8.6	U		Annotation|Plasmodium_falciparum_Sanger|(protein coc				
	K.HQEIVE	4.783	0.37	100	1569.747	1569.752		6910.7	1	7.917	87.5	1	
	R.KKQEH	4.3656	0.3535	100	2068.992	2068.99		6571.9	1	6.841	39.7	3	
	R.KKQEH	5.4137	0.4625	100	2069.998	2068.99		7255.1	1	8.373	70.6	9	
	K.RFDTHN	4.382	0.4241	100	2588.278	2587.273		4738.2	1	7.226	47.5	4	
PFI1685w		60	14.00%	342	40197	8.8	U		Annotation|Plasmodium_falciparum_Sanger|(protein coc				
	R.TYTLCG	4.6574	0.4188	100	2561.334	2560.331		6675.7	1	6.888	36.4	1	
	R.TYTLCG	4.1782	0.2991	100	2563.338	2560.331		5900.6	1	7.6	45.5	16	
	K.EHPWFS	4.0375	0.4602	100	2019.024	2019.02		9500.3	1	8.385	63.3	41	

	N.KNVEVF	2.9818	0.2397	99.8	1201.7	1201.695		6364.3	1	5.333	83.3	2
PFC0205c		3	14.00%	171	19920	9.2	U		Annotation|Plasmodium_falciparum_Sanger|(protein coc			
	K.GTPEKF	3.3306	0.2631	99.8	2597.352	2596.334		5820.5	1	5.337	39.1	3
PF14_0748		18	13.90%	274	32558	7.3	U		Annotation|Plasmodium_falciparum_TIGR|(protein codin			
	K.DNSYKD	3.2708	0.3596	100	1494.692	1494.698		6073.5	1	6.586	68.2	3
	R.NDLISIW	3.7275	0.4164	100	1479.835	1479.826		5326.6	1	6.683	77.3	13
	K.KIEDIKD	3.8481	0.3841	100	1709.909	1709.915		8177.7	1	6.448	69.2	2
PFL1005c		78	13.90%	266	31018	9.5	U		Annotation|Plasmodium_falciparum_Sanger|(protein coc			
	K.NLHNVG	4.8084	0.3766	100	2307.194	2306.189		6434.8	1	6.879	44.7	9
	K.NLHNVG	6.4758	0.5511	100	2327.241	2327.241		6179.2	1	10.006	63.2	18
	K.VIEYIYP	5.8362	0.4547	100	2128.205	2124.193		5437.2	1	8.013	78.1	44
	I.YPQELLN	3.6114	0.2545	99.9	1492.817	1492.816		5906.1	1	5.107	81.8	4
	Y.PQELLN	3.6599	0.2946	100	1329.754	1329.753		5775.5	1	6.206	90	3
PF14_0261		21	13.80%	377	42641	7.8	U		Annotation|Plasmodium_falciparum_TIGR|(protein codin			
	K.YTHSGS	4.3789	0.4166	100	1399.735	1399.73		4461.9	1	9.221	75	5
	K.YTHSGS	3.9872	0.3852	100	1527.835	1527.825		6031.3	1	6.831	80.8	2
	K.GISFPV	3.6352	0.403	100	3195.607	3195.608		5883.2	1	6.799	27.8	12
	K.VGINASI	3.1978	0.2657	99.9	1017.558	1017.558		5984.1	1	5.907	88.9	2
PF14_0373		38	13.80%	355	40981	7.6	U		Annotation|Plasmodium_falciparum_TIGR|(protein codin			
	N.GGTFNH	3.2275	0.2416	99.9	994.5201	994.5184		3309.7	1	4.423	75	1
	R.YAHYNC	4.5586	0.5435	100	1692.816	1692.814		7690.1	1	9.408	76.9	35
	R.NFRPAC	5.126	0.4733	100	3041.336	3041.335		6242.5	1	8.362	30	2
PF13_0179		70	13.70%	1272	151311	7.6	U		Annotation|Plasmodium_falciparum_Sanger|(protein coc			
	K.CNTPIS	2.9544	0.3964	100	1833.89	1833.892		5527.1	1	5.597	57.1	1
	K.NVYNIQ	4.8327	0.4721	100	1800.966	1800.968		9166.6	1	8.035	67.9	1
	K.YGIIDP	3.9805	0.3576	100	3426.727	3424.721		6655.8	1	6.543	32.1	8
	K.YGIIDP	5.7532	0.4817	100	4085.057	4082.048		5993	1	7.464	31.8	13
	K.LVENNE	4.1809	0.3718	100	1914.983	1913.976		6705.4	1	6.574	70	5
	K.RIPEVF	4.4824	0.4336	100	2327.08	2326.075		7277.4	1	8.422	58.3	3
	R.IPEVFD	3.4188	0.2857	99.9	2169.975	2169.974		6163.9	1	5.525	52.9	2
	K.VHYPFS	4.1157	0.4054	100	1721.793	1721.792		4627.1	1	6.872	73.1	15
	K.NDNIMD	3.6727	0.4163	100	2972.467	2971.46		5993	1	7.21	33.3	9
	K.LLNFIE	4.8286	0.501	100	1824.01	1823.004		7343.4	1	8.48	76.9	11
	R.NLNIID	4.9143	0.4067	100	2679.413	2679.407		8054.4	1	6.175	38.1	1
	K.DVQNLT	2.8944	0.2969	99.8	1612.788	1612.786		6224.7	1	5.032	75	1
PF11_0284		15	13.70%	293	35557	9.3	U		Annotation|Plasmodium_falciparum_TIGR|(protein codin			

	R.AHCNPL	3.5418	0.4136	100	1411.678	1411.675		5985.5	1	8.664	77.3	4
	R.NYTYVD	4.6432	0.5133	100	2223.067	2222.06		9200.9	1	8.716	68.8	9
	K.LIHESS	2.9042	0.3053	99.9	1286.66	1286.659		5510.7	1	5.905	65	2
PF14_0141		192	13.70%	219	25216	10	U		Annotation|Plasmodium_falciparum_TIGR|(protein codin			
	K.KADVNE	4.535	0.5159	100	3348.654	3347.651		8816	1	8.382	32.8	35
	K.KADVNE	6.6525	0.4324	100	3348.665	3347.651		8879.4	1	7.646	33.6	43
	K.ADVNEF	4.7212	0.423	100	3216.532	3212.539		7676.5	1	7.538	35.7	104
	K.ADVNEF	5.2402	0.4313	100	3224.565	3219.556		8034.5	1	6.8	31.2	4
	V.NEFSGV	3.8057	0.2825	99.9	2928.41	2927.406		4496.1	1	6.49	36	5
	F.SGVVHL	3.2415	0.361	100	2545.252	2544.27		5327	1	5.883	43.2	1
PF13_0204		41	13.50%	386	46017	5.7	U		Annotation|Plasmodium_falciparum_Sanger|(protein coc			
	R.NSYDQV	3.8253	0.2059	99.9	1294.607	1294.603		9085.2	1	4.565	80	3
	K.NNLNDS	4.6237	0.2296	100	1681.784	1678.772		4605.4	1	5.78	73.1	33
	K.QIKPEE	3.7919	0.3347	100	1843.969	1843.963		5447.2	1	6.098	60.7	1
	K.LNGMEV	4.0757	0.4336	100	1499.773	1497.764		7874.7	1	7.575	86.4	4
PF13_0232		10	13.50%	385	45285	3.8	U		Annotation|Plasmodium_falciparum_Sanger|(protein coc			
	K.QNLFLV	3.3864	0.3292	100	3010.581	3009.576		6431.4	2	5.902	33.3	5
	K.DMHEQI	5.2649	0.5281	100	2021.938	2021.94		7377.9	1	9.5	71.9	1
	M.HEQNA	4.4723	0.448	100	1775.874	1775.872		4601	1	8.468	75	3
	K.FLSPLL	2.9481	0.2546	99.8	1265.672	1265.672		6656.6	1	5.252	83.3	1
PFL2275c		74	13.50%	304	34827	5.5	U		Annotation|Plasmodium_falciparum_Sanger|(protein coc			
	K.KGNEVT	3.3872	0.443	100	1330.715	1330.712		5604.2	1	7.671	72.7	2
	K.KGNEVT	4.5795	0.4301	100	1330.731	1330.712		5655.3	1	7.504	68.2	9
	K.KGNEVT	4.0913	0.4066	100	1333.722	1330.712		7214.3	1	7.168	81.8	57
	K.VFDSSF	2.6293	0.3456	99.9	972.4429	972.4427		3445.4	4	6.2	85.7	3
	K.KSIYDY	3.4387	0.3885	100	1390.639	1390.638		7610.6	1	6.457	80	2
	K.KNEINE	3.3675	0.215	99.9	1171.693	1171.687		5731.5	1	5.849	83.3	1
PFI1730w		235	13.40%	1340	160417	8.8	U		Annotation|Plasmodium_falciparum_Sanger|(protein coc			
	K.SILDND	5.8529	0.4449	100	3385.727	3383.718		9701.9	1	7.753	33.9	19
	K.SILDND	4.0912	0.3467	100	3387.73	3383.718		9155.9	1	6.579	32.1	40
	K.SILDND	7.0821	0.4453	100	3982.127	3980.109		7028.1	1	8.4	33.3	56
	K.SDIIDY	3.8456	0.2727	99.9	2974.492	2971.455		5049.3	2	5.897	29.2	7
	K.NNIFST	3.6689	0.328	100	2665.272	2663.278		5255.4	1	5.683	42.9	3
	I.FSTSEE	3.2136	0.2987	99.8	2315.072	2315.091		5723.4	1	6.135	38.9	1
	K.YYLHNII	4.0157	0.3205	100	1890.026	1890.021		6648.2	1	7.629	75	3
	K.HGLIYA	2.8847	0.3179	99.8	1292.699	1292.7		6105.2	1	6.841	75	3

	K.IQGTYF	2.699	0.2934	99.9	1108.554	1108.554		3501	1	5.245	81.2	2
	R.LSVHDE	2.8229	0.3058	99.8	1212.637	1212.638		6468.9	1	6.114	77.8	3
	R.NILYFP	4.4667	0.3925	100	1754.922	1754.923		5663.7	1	7.182	80.8	30
	R.NILYFP	3.4904	0.3828	100	1890.037	1890.035		6987.8	34	6.085	42.9	3
	Y.FPNHLP	3.3838	0.2196	99.9	1253.656	1251.649		6037.1	2	4.84	83.3	6
	R.NKENVV	4.8951	0.2378	100	1559.774	1558.771		8901.8	1	5.649	87.5	6
	R.NKENVV	6.2816	0.506	100	3305.647	3304.645		7627.8	1	8.76	35.7	12
	K.ENVVQE	5.306	0.2983	100	3063.517	3062.507		6064.7	4	6.055	26.9	2
	K.GTDITP	4.3548	0.4239	100	1764.892	1764.892		3845.4	1	7.374	66.7	5
	K.RYELIP	3.7788	0.3259	100	1194.709	1194.707		6650.6	1	6.101	87.5	34
MAL8P1.10		10	13.40%	216	25930	9.3	U		Annotation|Plasmodium_falciparum_Sanger|(protein coc			
	R.NEQNNY	4.1893	0.517	100	3375.613	3373.601		7460.7	1	8.113	35.7	10
PF11_0454		7	13.40%	82	9145	8.5	U		Annotation|Plasmodium_falciparum_TIGR|(protein codin			
	K.TETFAI	3.2245	0.3509	100	1217.62	1217.628		5419.2	1	6.665	85	7
MAL13P1.283		77	13.30%	528	57973	6.5	U		Annotation|Plasmodium_falciparum_Sanger|(protein coc			
	G.GTIEDTI	2.9971	0.3337	99.9	1944.021	1943.027		4371.3	4	5.395	47.1	1
	R.DAVNDL	2.9238	0.3252	99.9	1404.741	1404.737		8777.4	1	5.728	75	3
	R.SLHDAL	3.2891	0.3184	100	1169.61	1169.61		4112.5	1	5.893	88.9	3
	K.AFSDAL	4.4445	0.4465	100	3169.721	3167.718		6856.7	1	7.818	37.5	30
	L.ELIPYT	4.5687	0.4239	100	2563.436	2563.432		6336.9	1	7.691	43.2	3
	E.LIPYTL	3.9277	0.4011	100	2435.389	2434.39		6650.3	1	6.978	45.2	1
	I.PYTLAE	6.1593	0.5434	100	2203.21	2201.204		5961.5	1	9.533	63.2	32
	Y.TLAENA	4.5083	0.4648	100	1941.088	1941.088		6839.1	1	8.536	64.7	4
PF08_0074		21	13.30%	248	27259	10.6	U		Annotation|Plasmodium_falciparum_Sanger|(protein coc			
	K.KDREPI	3.7587	0.3451	100	1539.714	1539.719		7902.5	1	6.307	68.2	1
	K.DREPID	3.7597	0.3916	100	1411.627	1411.624		5724.1	1	7.109	85	4
	R.MTNYVN	2.969	0.3399	100	1160.543	1160.541		6960.3	1	6.362	83.3	6
	K.TVSFIEI	3.4443	0.2947	100	1291.791	1291.781		6869.5	1	6.371	85	10
PFF0250w		4	13.20%	755	85631	6.9	U		Annotation|Plasmodium_falciparum_Sanger|(protein coc			
	E.FDVSVY	5.2534	0.2481	99.9	8214.529	8212.679		8652.4	3	4.342	11.2	1
	R.SSCEIN	3.598	0.3816	100	1797.881	1797.881		7404.4	1	6.959	56.7	2
	R.MHPNNS	2.6219	0.3758	99.9	1252.599	1252.597		2548	1	6.002	88.9	1
PFL1200c		3	13.20%	106	12263	9.6	U		Annotation|Plasmodium_falciparum_Sanger|(protein coc			
	K.NALDHL	3.338	0.3862	100	1470.737	1470.745		6395.7	1	7.533	73.1	3
PF13_0082		113	13.10%	206	24443	6.4	U		Annotation|Plasmodium_falciparum_Sanger|(protein coc			
	C.THFNIG	2.8496	0.3357	99.9	1213.618	1212.613		4870.3	1	6.066	77.8	1

	K.TSELFD	6.2246	0.4355	100	2134.999	2131.982		8423	1	8.315	75	112
PFC0262c		11	13.10%	137	17020	10	U		Annotation|Plasmodium_falciparum_Sanger|(protein coc			
	R.HTAYGC	5.5844	0.5406	100	2082.07	2081.064		6794	1	9.072	64.7	11
PF11_0465		38	13.00%	837	96374	8.2	U		Annotation|Plasmodium_falciparum_TIGR|(protein codin			
	K.TISQAL	3.8179	0.2189	99.9	1804.969	1803.961		8732	1	6.25	67.9	3
	K.KLPPAL	3.4371	0.3167	99.9	1617.89	1617.885		3214.3	1	6.069	64.3	13
	K.LYELGT	3.4055	0.3598	100	1533.821	1533.816		9236.1	1	6.62	65.4	4
	R.TIFNEF	3.652	0.4825	100	1583.802	1581.791		7731.8	1	8.051	75	15
	K.NVTSEL	4.4211	0.2659	99.8	4583.211	4582.198		7169.2	1	5.07	15.8	1
	K.INAPVF	3.2483	0.3583	100	1318.66	1318.658		4953.7	1	6.635	85	2
PF10_0120		1	13.00%	192	23020	9.3	U		Annotation|Plasmodium_falciparum_TIGR|(protein codin			
	R.LFLHPS	4.136	0.3979	100	3025.522	3024.522		3595.7	1	7.877	35.4	1
PFB0161c		1	13.00%	100	11014	9.8	U		Annotation|Plasmodium_falciparum_TIGR|(protein codin			
	K.AAGNLD	3.2649	0.2641	99.9	1387.702	1386.698		5671.4	1	5.527	58.3	1
PFC0900w		9	12.90%	535	59176	6	U		Annotation|Plasmodium_falciparum_Sanger|(protein coc			
	K.SQDNEI	4.4267	0.3854	100	3132.685	3131.674		7867.1	1	6.918	30.2	2
	R.SVHDAL	2.8392	0.3189	99.9	1143.565	1143.558		3306.9	1	6.846	88.9	6
	R.AFGNAL	2.9344	0.3325	99.8	3186.745	3186.747		4834.4	1	5.286	26.8	1
MAL13P1.343		11	12.90%	311	35212	6.7	U		Annotation|Plasmodium_falciparum_Sanger|(protein coc			
	R.AGVPMI	5.1708	0.4796	100	2437.227	2437.237		10965.7	1	9.125	45.2	8
	H.SHPGF	3.5587	0.4163	100	1990.907	1990.908		5881.3	1	7.197	52.9	3
PFL1845c		16	12.90%	256	30224	8.2	U		Annotation|Plasmodium_falciparum_Sanger|(protein coc			
	K.INNLND	4.4257	0.3492	100	2170.177	2170.161		7296.7	1	7.38	50	14
	K.IQECIEN	4.0448	0.44	100	1672.863	1672.858		7650.6	1	6.997	76.9	2
PF14_0091		3	12.90%	132	15540	7.8	U		Annotation|Plasmodium_falciparum_TIGR|(protein codin			
	K.YSVAD	4.8797	0.5265	100	2042.873	2041.872		6692.3	1	8.68	68.8	3
MAL8P1.52		7	12.90%	124	14060	9.7	U		Annotation|Plasmodium_falciparum_Sanger|(protein coc			
	K.KPNSKE	5.1802	0.2812	100	1898.011	1897.003		6471.5	1	5.728	63.3	5
	K.KPNSKE	4.4416	0.2812	100	1904.023	1904.02		5057.7	5	6.055	38.3	1
	N.SKEEEE	3.3463	0.2873	99.9	1564.833	1564.83		5344.5	1	5.33	62.5	1
PFL0660w		3	12.90%	93	10764	8	U		Annotation|Plasmodium_falciparum_Sanger|(protein coc			
	R.KYDPTV	2.8543	0.3573	99.9	1518.736	1517.732		5561.5	1	6.272	72.7	3
PFI0235w		50	12.80%	484	56235	7.7	U		Annotation|Plasmodium_falciparum_Sanger|(protein coc			
	K.FWGSS/	4.3394	0.4799	100	1934.007	1933.993		7360.9	1	8.487	63.3	5
	K.HIGTVS	2.8225	0.3851	100	976.5737	975.5701		3519.6	1	6.664	93.8	3
	R.AFSDEG	3.327	0.4013	100	1313.637	1313.638		6216.8	1	7.286	81.8	3

	K.NLDEDT	5.2642	0.3883	100	2302.066	2301.06	8569	1	7.157	73.5	36	
	R.VNFTAM	2.614	0.2713	99.9	810.42	810.4184	3341.9	1	6.297	91.7	3	
PFI0385c		22	12.80%	367	43495	9.3	U		Annotation|Plasmodium_falciparum_Sanger|(protein coc			
	M.HFNLQE	2.9232	0.3627	100	1332.575	1332.575	5663.1	1	6.442	77.8	3	
	K.YLISLIS	4.7906	0.4252	100	1681.974	1681.983	8627.6	1	9.268	73.1	15	
	R.IGFTHD	4.1141	0.3434	100	2515.204	2514.201	5999.4	1	6.742	52.3	2	
	R.IGFTHD	5.4653	0.3273	100	2516.213	2514.201	9486.1	1	6.614	42	2	
PF11_0166		5	12.80%	304	33881	10	U		Annotation|Plasmodium_falciparum_TIGR|(protein codin			
	R.MNNAG	3.8832	0.3856	99.9	2588.178	2588.176	6225.7	1	7.28	38.5	3	
	R.KTNINN	3.8801	0.3856	100	1531.786	1531.783	5958.7	1	6.31	76.9	2	
PF11_0040		1	12.80%	94	10231	10	U		Annotation|Plasmodium_falciparum_TIGR|(protein codin			
	K.AGAKTI	2.873	0.3841	100	1179.686	1179.681	5126.9	1	6.676	72.7	1	
PFI1735c		16	12.60%	713	83041	5.6	U		Annotation|Plasmodium_falciparum_Sanger|(protein coc			
	R.QVDNQ	5.7018	0.4446	100	2143.883	2142.874	10021.8	1	8.836	67.6	3	
	K.TQGDVI	3.3304	0.2735	99.9	1157.583	1157.58	4348.4	1	6.786	70	3	
	K.IRDLQV	2.7267	0.3006	99.9	1147.705	1147.703	6795.8	1	5.612	87.5	1	
	K.EYYDKE	4.5871	0.3831	100	5051.303	5049.291	6826.6	1	5.737	20.1	3	
	K.DEQHEI	4.0093	0.1891	99.9	1451.709	1451.713	9070.2	2	6.011	72.7	6	
PF11_0099		66	12.60%	540	62377	9	U		Annotation|Plasmodium_falciparum_TIGR|(protein codin			
	A.KEYHPD	3.3957	0.2248	99.8	1576.802	1576.809	4659.7	1	5.552	62.5	1	
	R.GEHAH	4.6777	0.5457	100	2227.049	2226.037	7180.2	1	8.392	58.3	5	
	R.GEHAH	5.2489	0.4317	100	2234.064	2233.054	10788.1	1	8.122	44.4	3	
	K.TPSLLL	5.9927	0.5854	100	2253.252	2250.22	7001.3	1	11.023	75	45	
	K.NNLYGH	3.5836	0.3021	99.9	1769.878	1766.871	4431.6	1	5.952	67.9	12	
PFD1170c		49	12.60%	309	35962	8.6	U		Annotation|Plasmodium_falciparum_Sanger|(protein coc			
	R.LNTIGN	2.8538	0.3764	100	1341.715	1341.713	6812	1	6.482	59.1	1	
	K.KIESHL	4.7969	0.4305	100	1469.756	1469.754	9104	1	8.444	86.4	28	
	K.KIESHL	3.601	0.4115	100	1469.757	1469.754	4564.2	1	7.274	68.2	2	
	K.TVSNAV	3.9981	0.4095	100	1759.825	1759.822	5169.6	1	7.318	60.7	7	
	K.TVSNAV	4.8412	0.5506	100	1766.841	1766.839	9398.2	1	8.863	67.9	11	
PF10_0372		16	12.50%	120	13840	9.5	U		Annotation|Plasmodium_falciparum_TIGR|(protein codin			
	K.DDAHK	3.5658	0.4553	100	1751.809	1751.806	5544	1	7.716	64.3	16	
PF13_0276		8	12.40%	137	15849	7.4	U		Annotation|Plasmodium_falciparum_Sanger|(protein coc			
	R.IVNITP	2.9669	0.3011	99.8	1392.749	1392.749	5765.9	1	5.114	77.3	2	
	R.IVNITP	3.9453	0.3761	100	1878.995	1878.993	5078.7	1	8.187	68.8	6	
PFE1500c		3	12.40%	97	11540	4.7	U		Annotation|Plasmodium_falciparum_Sanger|(protein coc			

	K.RYVPTQ	4.1019	0.2331	100	1561.809	1561.809		7539.5	1	5.953	47.7	3
PFB0680w		21	12.30%	951	112487	5.4	U		Annotation|Plasmodium_falciparum_TIGR|(protein codin			
	K.GKQDIS	3.5148	0.3238	100	1539.788	1539.784		6896	1	6.025	61.5	2
	K.ISDDHK	3.3354	0.2284	99.8	1444.742	1441.729		5489.7	1	4.258	77.3	1
	K.KSDDHK	3.7422	0.2283	99.9	1457.748	1456.74		4251.1	1	6.048	72.7	8
	K.KSDDHK	3.3726	0.2091	99.8	1334.705	1334.703		4758.8	17	5.092	75	1
	K.NKDENK	5.4626	0.3997	100	6952.949	6951.935		9663.2	2	6.443	12.3	3
	K.VNNHIF	3.8434	0.4324	100	1349.707	1349.704		7211.2	1	6.391	85	6
PF14_0615		47	12.30%	382	44532	5.1	U		Annotation|Plasmodium_falciparum_TIGR|(protein codin			
	K.KLTEAD	3.9159	0.332	100	1375.733	1375.732		7530.5	1	6.22	81.8	3
	K.LTEADT	3.4734	0.3284	100	1247.64	1247.637		5977.8	1	6.596	80	3
	K.MAHEFN	3.3864	0.4337	100	1187.576	1187.575		3657.5	1	6.737	87.5	12
	K.MIDNVI	5.091	0.3482	100	1658.916	1658.915		9625.1	1	6.581	82.1	18
	K.AWLEDF	3.5676	0.3342	100	1480.725	1480.734		9141.7	1	6.266	85	10
	A.WLEDFY	3.2903	0.3622	100	1409.698	1409.697		5906.5	1	6.45	83.3	1
PFE0435c		29	12.30%	284	33860	8.3	U		Annotation|Plasmodium_falciparum_Sanger|(protein coc			
	N.LQNDFN	3.1098	0.297	99.9	1441.69	1440.687		7106	1	4.897	75	2
	K.VATFSL	4.4418	0.5309	100	1542.779	1541.775		7004	1	9.076	83.3	24
	K.WHTND	3.1539	0.436	100	1357.599	1357.596		6076.4	1	6.095	80	3
MAL7P1.67		255	12.30%	204	23844	5.6	U		Annotation|Plasmodium_falciparum_Sanger|(protein coc			
	K.DIQGFD	5.5821	0.5162	100	2853.457	2853.45		8203.9	1	8.207	38.5	19
	K.DIQGFD	5.5663	0.5189	100	2857.449	2853.45		11438.9	1	9.458	52.1	236
PFL0670c		51	12.20%	746	86647	7.4	U		Annotation|Plasmodium_falciparum_Sanger|(protein coc			
	K.NMQEVI	3.7283	0.2697	100	1344.734	1344.729		8790.3	1	5.79	75	1
	K.NNSNSN	4.5166	0.3434	100	2524.218	2524.208		3409.7	1	7.187	40.5	3
	K.KLNVEN	3.9344	0.3336	100	1798.982	1798.974		8673	1	6.369	64.3	3
	K.NHIEGF	4.3302	0.3617	100	1721.879	1720.877		8720.8	1	6.936	75	2
	R.EFLWQE	4.2007	0.38	100	1485.724	1482.713		3611.1	1	6.505	81.8	25
	R.AIQAAT	5.1874	0.493	100	1702.898	1699.888		10015.6	1	8.293	80	17
PFL0105w		10	12.20%	401	46668	5.4	U		Annotation|Plasmodium_falciparum_Sanger|(protein coc			
	K.IINVCLW	4.238	0.4333	100	1868.984	1867.978		5510.7	1	7.475	73.3	2
	K.KTPYYI	4.2458	0.4586	100	1696.849	1696.841		7237.3	1	8.393	76.9	5
	K.ELSDEA	4.7988	0.4097	100	2209.997	2209.992		6485.7	1	7.124	58.3	3
PFL0190w		17	12.20%	147	16539	7.4	U		Annotation|Plasmodium_falciparum_Sanger|(protein coc			
	K.IYHPNIN	4.383	0.3197	100	2027.074	2026.08		5162.2	1	6.636	67.6	17
PF13_0324		43	12.10%	956	108681	7.6	U		Annotation|Plasmodium_falciparum_Sanger|(protein coc			

	K.TNEGP	4.8534	0.4359	100	3706.709	3704.687		5704.3	1	6.767	27.4	3
	K.AYVPLG	3.5288	0.4172	100	3616.953	3614.945		7616.9	1	8.261	31.8	8
	K.AAFMVL	2.6527	0.3569	100	907.5441	907.544		5879	1	7.499	92.9	3
	K.NSGGSL	3.6296	0.3382	100	2395.082	2394.079		5433.1	1	6.459	47.4	18
	R.LHTYAL	3.7043	0.3665	100	1525.894	1525.893		7300.3	1	6.353	66.7	9
	R.MFSLHN	2.5813	0.3035	99.8	1118.567	1118.567		5028.4	1	6.555	81.2	2
PF14_0700		18	12.10%	454	52454	5.3	U		Annotation|Plasmodium_falciparum_TIGR|(protein codin			
	K.MENQN	5.5641	0.512	100	2152.899	2151.885		6098.1	1	9.49	60.5	3
	K.FHGDLN	5.8885	0.5173	100	2593.294	2592.292		5556.8	1	9.829	66.7	12
	R.KKEDFF	4.3347	0.1929	99.9	1606.799	1606.796		7627	1	5.811	83.3	3
PF14_0027		28	12.10%	149	17400	9.5	U		Annotation|Plasmodium_falciparum_TIGR|(protein codin			
	K.RQCDN	5.2372	0.4343	100	2136.92	2135.917		7522.8	1	8.117	51.5	12
	K.RQCDN	3.6251	0.3902	100	2136.924	2135.917		7232	1	5.97	67.6	4
	R.QCDNC	4.3641	0.4079	100	1979.819	1979.816		6541.6	1	7.21	46.9	6
	R.QCDNC	4.1132	0.3172	100	1980.82	1979.816		5410.9	1	7.691	68.8	6
PF14_0434		17	12.00%	351	41934	9.4	U		Annotation|Plasmodium_falciparum_TIGR|(protein codin			
	K.IIEVHDN	4.7556	0.407	100	1588.917	1587.911		7240.8	1	7.08	80.8	8
	K.RNDTLE	3.6785	0.4724	100	1796.882	1796.878		4387	1	7.045	71.4	3
	K.KVDEGN	4.2853	0.3995	100	1623.773	1623.777		5737.4	1	7.232	79.2	6
PFE0395c		4	12.00%	349	40587	7.5	U		Annotation|Plasmodium_falciparum_Sanger|(protein coc			
	K.FVPLNL	3.6804	0.3517	100	2880.43	2880.424		7826.8	1	6.379	41.7	2
	K.RYPNEE	3.0832	0.2899	99.8	2208.07	2208.08		2895.8	1	5.497	46.9	2
PFI0475w		31	12.00%	125	14178	10.1	U		Annotation|Plasmodium_falciparum_Sanger|(protein coc			
	K.LLHEGI	3.7895	0.396	100	1633.893	1633.891		7266.9	1	6.255	46.4	2
	K.LLHEGI	4.7904	0.4461	100	1647.928	1647.926		8838.5	1	9.057	85.7	29
PF08_0032		79	11.90%	655	76682	9.2	U		Annotation|Plasmodium_falciparum_Sanger|(protein coc			
	K.IYPTIF	5.084	0.4575	100	2996.595	2994.588		5597.9	1	7.044	34	2
	K.IYPTIF	4.9072	0.4346	100	2998.599	2994.588		5194.9	1	8.334	40	67
	K.DFQNFI	3.7808	0.3586	100	2220.089	2220.08		4070.9	1	5.689	52.9	1
	K.YDSFLL	4.367	0.4936	100	2358.14	2357.138		5179.1	1	7.834	52.6	4
	K.YCLPFN	3.6109	0.3366	100	1829.929	1829.927		4538.3	1	6.597	65.4	5
PFL2375c		4	11.90%	159	18685	9.2	U		Annotation|Plasmodium_falciparum_Sanger|(protein coc			
	K.HLFDEI\	3.1117	0.2773	100	1007.565	1007.564		4062.6	1	5.584	92.9	1
	K.DYLDW\	3.4164	0.3038	100	1352.652	1352.649		6312.6	1	7.25	80	3
PFF1415c		41	11.80%	380	44669	8.1	U		Annotation|Plasmodium_falciparum_Sanger|(protein coc			
	K.FSEIAE	4.6421	0.3356	100	1842.871	1842.865		7459.3	1	7.999	80	13

	K.FSEIAE	4.6373	0.5392	100	1999.973	1998.966		9673.8	1	8.11	65.6	4	
	R.VNNDLH	5.1164	0.4726	100	1699.92	1699.921		8854.6	1	7.996	76.9	1	
	K.NLEHIS	3.9949	0.4267	100	1597.943	1597.933		4429	1	7.282	76.9	3	
	K.NLEHIS	5.0186	0.3752	100	1598.935	1597.933		7238.7	1	6.984	84.6	20	
PFL2035c		3	11.80%	186	22132	8.5	U		Annotation|Plasmodium_falciparum_Sanger|(protein coc				
	K.APNSVL	4.0318	0.3777	100	2500.252	2497.243		5319.3	2	6.381	42.9	3	
MAL13P1.253		1	11.80%	93	10927	9.2	U		Annotation|Plasmodium_falciparum_Sanger|(protein coc				
	K.IMTQPIN	3.2318	0.361	100	1381.796	1381.793		4486	1	6.018	70	1	
PFD1015w		4	11.70%	385	44930	6.2	U		Annotation|Plasmodium_falciparum_Sanger|(protein coc				
	K.MKPNA	6.1356	0.5149	100	3455.488	3454.473		11447.7	1	8.611	32.1	1	
	K.KLTTVA	4.2408	0.4668	100	1669.97	1668.965		7334.9	1	7.643	73.3	3	
PFA0260c		7	11.70%	162	18528	5.2	U		Annotation|Plasmodium_falciparum_Sanger|(protein coc				
	K.CNVVLN	6.3328	0.4505	100	2058.172	2058.164		7325.2	1	9.609	76.5	5	
	K.CNVVLN	3.6034	0.3483	99.9	2158.194	2158.191		4976.2	1	6.455	37.5	2	
PFE1250w		79	11.60%	673	76852	8.2	U		Annotation|Plasmodium_falciparum_Sanger|(protein coc				
	K.DKLCEN	3.4767	0.3482	100	1411.649	1411.653		6470.6	9	5.939	70	3	
	K.KFNNMV	3.1213	0.354	100	1485.705	1485.695		8206.1	1	6.266	65	6	
	K.FNNMW	3.4416	0.3476	100	1357.603	1357.6		8052.7	1	5.942	88.9	3	
	K.TLILVDE	3.2037	0.3367	99.9	1664.95	1664.962		8346	1	5.692	65.4	4	
	K.VGYYSC	4.1504	0.3723	100	2635.381	2634.378		5021.2	1	6.954	43.2	6	
	K.VGYYSC	4.0183	0.3389	100	3194.735	3193.734		6426.8	1	6.444	35.2	5	
	K.VGYYSC	5.4438	0.4204	100	3194.741	3193.734		7059.6	1	7.301	35.2	10	
	R.NIGHIG	4.3316	0.4202	100	1658.91	1658.899		5161.8	1	8.774	60.7	8	
	R.NIGHIG	4.8256	0.3425	100	1659.912	1658.899		7563.5	1	6.995	71.4	34	
PFL2005w		37	11.60%	336	37638	7.3	U		Annotation|Plasmodium_falciparum_Sanger|(protein coc				
	K.YRPEFL	4.4774	0.4137	100	2357.268	2357.262		8691.4	1	7.035	63.2	21	
	K.IIILDE	4.9243	0.3991	100	2104.109	2104.096		7461.6	1	7.55	66.7	16	
PF14_0579		2	11.60%	121	13795	10.3	U		Annotation|Plasmodium_falciparum_TIGR|(protein codin				
	R.ERPYSY	3.8599	0.3174	100	1684.837	1684.837		7874.2	4	6.331	57.7	2	
PF11_0377		10	11.50%	323	37631	9.3	U		Annotation|Plasmodium_falciparum_TIGR|(protein codin				
	R.YASINT	3.4041	0.4292	100	1602.842	1602.843		5188.3	1	6.474	61.5	1	
	R.FEDRPD	2.4491	0.3759	99.8	1474.696	1474.697		5346.2	1	6.176	65	3	
	K.NRFDQT	3.4559	0.3455	100	1436.655	1436.652		6028.1	2	5.888	68.2	6	
PF13_0116		57	11.40%	1258	149179	6.9	U		Annotation|Plasmodium_falciparum_Sanger|(protein coc				
	K.HSNDVF	4.1017	0.4846	100	1362.777	1362.775		6919.3	1	9.764	81.8	7	
	K.HFDFDY	3.3307	0.4452	100	1365.624	1365.623		6751.9	1	8.302	85	4	

	K.NSNEHH	4.6276	0.2457	99.8	5012.082	5009.065		5710.8	4	5.1	14.6	1
	R.FQDKDY	4.4954	0.326	100	1553.762	1553.76		7995.5	1	6.816	90.9	3
	K.FKIPTN	3.896	0.3326	100	1597.909	1597.907		4454.3	2	5.579	61.5	6
	K.IPTNVG	3.4393	0.3944	100	1315.729	1315.726		7892.4	1	7.955	77.3	4
	K.FDNKND	4.6682	0.3838	100	1735.84	1735.84		5729.7	1	7.251	76.9	9
	K.NDLNHY	3.1165	0.2674	99.9	1231.608	1231.607		5576.4	1	5.82	83.3	2
	R.WISDVL	4.9339	0.3047	100	2987.43	2986.42		5361.9	1	5.774	37	17
	K.AIQNIFD	3.5118	0.3645	100	1693.933	1693.928		8233.1	1	6.639	61.5	4
PF08_0065		18	11.40%	491	56509	5.3	U		Annotation|Plasmodium_falciparum_Sanger|(protein coc			
	R.RKENID	6.3501	0.4197	100	3106.563	3105.557		11122	1	8.406	41.3	2
	K.KGNGLG	4.5633	0.4938	100	1757.779	1757.777		7854.8	1	9.919	76.7	6
	R.NTNNAV	3.8033	0.4005	100	1476.805	1476.804		9406.2	1	6.784	75	10
PF11_0295		67	11.40%	369	44002	7.8	U		Annotation|Plasmodium_falciparum_TIGR|(protein codin			
	K.NAVNDV	5.7698	0.5202	100	1763.947	1763.945		6886.5	1	9.823	78.6	43
	K.AFELCS	4.8185	0.4854	100	1848.941	1848.935		4914.6	1	8.647	71.4	4
	K.FINDIYE	4.6723	0.4377	100	1617.837	1617.833		6007.5	1	7.649	90.9	20
PFD0770c		107	11.40%	220	25923	11.3	U		Annotation|Plasmodium_falciparum_Sanger|(protein coc			
	K.YYEVIL	5.1821	0.4229	100	1811.947	1808.941		4838.4	1	7.945	75	105
	K.INWICN	3.1002	0.2983	99.9	1296.699	1294.692		8076.2	1	5.423	83.3	2
PF11_0170		4	11.40%	167	18588	6.4	U		Annotation|Plasmodium_falciparum_TIGR|(protein codin			
	K.VIDGMD	4.0504	0.3951	100	2051.057	2050.049		8832.3	1	7.334	63.9	4
PFL0895c		79	11.30%	1061	122755	6.6	U		Annotation|Plasmodium_falciparum_Sanger|(protein coc			
	K.ICNIHNE	3.0361	0.2836	99.9	1279.671	1279.677		6626.5	1	5.632	83.3	1
	K.KVNIDD	3.3468	0.2217	99.9	1217.656	1217.66		6600.7	1	5.522	77.8	1
	R.ELVNIV	4.4448	0.2911	100	2325.329	2324.323		8957.5	1	5.895	40	5
	R.ELVNIV	5.4742	0.3964	100	2325.335	2324.323		8861.3	1	7.722	62.5	33
	K.QGLYDE	4.4068	0.3046	100	2252.073	2251.067		9348	1	6.368	55.9	21
	K.IKIENK	5.1367	0.5614	100	1818.145	1818.14		4735.3	1	9.994	70	1
	K.IENKPL	4.4316	0.4187	100	1562.929	1562.927		3765.3	1	7.115	73.1	5
	R.NNNTDE	3.3574	0.2269	99.9	1288.615	1288.613		4207.5	1	5.236	80	3
	K.AVETLN	4.6862	0.3596	100	2252.149	2251.14		7255.1	1	7.517	52.6	6
	K.FIEELN	4.4114	0.3488	100	1763.864	1762.859		9848.1	1	7.581	80.8	3
MAL8P1.125		37	11.30%	373	43453	7.5	U		Annotation|Plasmodium_falciparum_Sanger|(protein coc			
	K.SDENSA	3.6043	0.406	100	2165.941	2165.937		7601.2	1	6.311	50	3
	K.TYYTLQ	5.3574	0.4644	100	2809.395	2808.388		4682.1	1	7.484	40.9	14
	K.TYYTLQ	4.2412	0.476	100	2816.406	2815.406		5462.8	1	7.493	40.9	20

PF14_0627		33	11.30%	221	24668	10.2	U		Annotation|Plasmodium_falciparum_TIGR|(protein codin				
	K.FINDGV	5.0495	0.4212	100	1984.001	1982.998		7425.2	1	7.747	71.9	30	
	K.RFVNTA	2.9888	0.2467	99.8	965.5349	964.5328		4281.1	3	4.856	85.7	3	
PF10_0036		3	11.20%	152	17935	8.6	U		Annotation|Plasmodium_falciparum_TIGR|(protein codin				
	R.VSNNAA	3.555	0.3016	99.9	1987.029	1987.029		5153.1	1	5.735	46.9	3	
PF10_0170		10	11.20%	134	15677	5.1	U		Annotation|Plasmodium_falciparum_TIGR|(protein codin				
	K.HINNVN	5.0041	0.4908	100	1778.927	1778.934		7864.5	1	8.637	85.7	6	
	K.HINNVN	4.456	0.331	100	1778.936	1778.934		6230.8	1	6.955	60.7	4	
PF11_0270		43	11.10%	1013	119546	8.7	U		Annotation|Plasmodium_falciparum_TIGR|(protein codin				
	R.KPITIEL	3.9023	0.4894	100	1447.897	1447.904		6692.5	1	7.376	75	2	
	K.SILWDL	3.3263	0.3153	99.9	1770.945	1769.937		4599.9	1	5.605	57.1	7	
	K.LFGGFL	2.9752	0.3077	99.9	1340.799	1340.806		7792.3	1	5.48	70.8	1	
	K.ETSPGS	3.4988	0.3969	100	1690.773	1689.77		6776.5	1	6.125	60.7	3	
	R.LADFGV	3.1054	0.2906	99.9	1029.574	1027.569		6381.1	1	6.372	93.8	14	
	K.FIGQIS	3.9738	0.4434	100	1899.034	1899.032		7436.5	1	6.466	70	5	
	K.DSQNHN	3.8356	0.511	100	3633.711	3632.708		5286.3	1	8.125	26.7	5	
	K.DSQNHN	5.6849	0.3748	100	3647.758	3646.742		5778	1	6.376	26.7	6	
PFI1310w		90	11.10%	839	97764	6.8	U		Annotation|Plasmodium_falciparum_Sanger|(protein coc				
	K.ENLTDN	3.0485	0.3261	99.9	2335.14	2334.138		5891.3	2	5.44	38.9	1	
	K.NSDENI	4.5415	0.4203	100	2048.986	2048.994		6177.1	1	6.971	51.6	3	
	K.NSDENI	4.2721	0.401	100	2057.014	2056.012		5160.1	1	6.368	62.5	19	
	K.DINSYH	4.0895	0.4457	100	2373.126	2373.14		9192.4	1	7.087	38.9	2	
	K.DINSYH	4.4153	0.4346	100	2387.175	2387.175		5558.2	1	7.844	52.8	36	
	K.INQYHP	4.3583	0.4503	100	1873.992	1873.989		8127.4	1	7.715	63.3	26	
	K.INQYHP	3.8558	0.3898	100	1873.993	1873.989		5138.7	1	6.414	53.3	1	
	K.LFILPP	3.8403	0.446	100	2492.319	2492.301		4923.7	1	6.812	40.5	2	
PFI0950w		63	11.10%	515	61001	6	U		Annotation|Plasmodium_falciparum_Sanger|(protein coc				
	R.FGEYSD	4.355	0.3203	100	2199.056	2199.057		6487.5	1	7.151	58.8	10	
	K.IMDENI	4.8379	0.4151	100	2367.152	2366.159		7183.7	1	8.352	60.5	33	
	K.IMDENI	4.1882	0.3837	100	2380.176	2380.193		3435.5	1	6.554	42.1	2	
	K.LDTLID	4.9908	0.4428	100	2237.072	2236.076		10593.6	1	7.879	66.7	18	
PF10_0328		12	11.10%	488	55938	5.7	U		Annotation|Plasmodium_falciparum_TIGR|(protein codin				
	K.LQYKPL	4.1369	0.4572	100	2461.392	2460.35		4472	1	7.824	50	5	
	R.NVSQSN	5.5394	0.4446	100	3737.938	3734.914		9926.8	1	7.527	28.1	7	
PF10_0174		135	11.10%	467	55209	5.8	U		Annotation|Plasmodium_falciparum_TIGR|(protein codin				
	R.LAQLLN	5.9716	0.4908	100	2468.337	2464.326		8406	1	9.585	61.4	116	

	L.AQLLNA	5.4683	0.5441	100	2352.247	2351.242		6122.1	1	9.734	54.8	9
	L.LNASIE	5.0975	0.3974	100	2040.064	2039.062		7958.2	1	8.587	69.4	2
	K.NPENLL	5.9427	0.3983	100	3375.75	3375.744		8596.3	1	7.645	29.5	8
PF14_0453		8	11.10%	342	40695	9.5	U		Annotation|Plasmodium_falciparum_TIGR|(protein codin			
	K.YREFAD	3.7549	0.3417	100	2425.24	2424.235		6356	1	6.639	52.5	4
	R.EFADAS	3.3953	0.2772	99.8	2112.082	2112.088		5480.7	1	5.898	52.8	1
	R.NIMGQN	3.3571	0.2822	99.9	1787.96	1787.951		6398.1	1	5.46	56.2	3
MAL7P1.225		4	11.10%	253	29941	4.9	U		Annotation|Plasmodium_falciparum_Sanger|(protein coc			
	R.KCDNIN	3.7122	0.2125	99.9	1490.705	1490.702		6787.7	1	6.236	77.3	1
	K.QLTLEE	4.0763	0.3738	100	1881.981	1881.981		8533.4	1	6.756	66.7	3
PFC0435w		100	11.00%	1294	154088	5.3	U		Annotation|Plasmodium_falciparum_Sanger|(protein coc			
	K.NLYTLD	5.0559	0.2834	100	2635.335	2631.331		7571.3	1	5.789	50	49
	K.NIKENE	3.3271	0.3074	99.9	1601.796	1601.788		5808.8	17	5.454	58.3	2
	K.LYNNND	5.3302	0.4159	100	2422.248	2421.245		6886	1	8.176	57.9	8
	K.NLIDVT	4.3631	0.4074	100	1937.991	1936.988		5941.3	1	6.724	56.2	18
	K.NNDSD	4.073	0.5595	100	1718.673	1718.676		3456.1	1	8.501	64.3	6
	K.KSSVPT	4.6364	0.4528	100	1900.969	1897.958		6238.2	1	8.12	56.2	5
	K.SSVPTN	3.8754	0.4024	100	1770.873	1769.863		4619	1	6.978	60	2
	F.HTSNM	3.9215	0.4975	100	1533.688	1533.687		5085	1	7.803	66.7	3
	K.DIAQPP	4.6188	0.5457	100	2885.232	2884.255		7563.9	1	9.615	47.9	7
PFL1545c		34	11.00%	702	79605	5.1	U		Annotation|Plasmodium_falciparum_Sanger|(protein coc			
	L.YNENKD	5.1471	0.4354	100	4332.159	4331.197		10829.4	1	7.171	22.9	2
	Y.NENKDY	5.5947	0.3347	100	4217.185	4217.254		10678.8	1	6.976	23.6	11
	K.SNFIQEI	3.3769	0.2374	99.9	1257.716	1257.714		8507.1	1	5.744	77.8	17
	K.IEEDLQ	3.582	0.2588	99.9	1761.852	1761.851		4572.3	1	5.238	64.3	2
	K.MGANIV	3.3121	0.3284	99.9	1547.848	1546.851		8859.3	1	5.829	64.3	2
PF10_0330		1	11.00%	191	21571	4.7	U		Annotation|Plasmodium_falciparum_TIGR|(protein codin			
	K.VHVTLP	4.7318	0.5156	100	2344.163	2342.16		6004.5	1	8.527	52.5	1
PF10_0038		6	11.00%	118	13513	9.6	U		Annotation|Plasmodium_falciparum_TIGR|(protein codin			
	R.KSPCGE	3.2055	0.4277	100	1507.663	1507.66		5219	1	6.769	62.5	6
PFF0330w		133	10.90%	1537	180019	8.5	U		Annotation|Plasmodium_falciparum_Sanger|(protein coc			
	K.FEEHEG	2.5455	0.3204	99.8	1099.517	1099.517		5297.5	2	5.499	75	2
	K.GVNCCT	4.9175	0.528	100	2350.086	2349.083		6052.8	1	8.492	47.5	7
	K.NNNSNV	4.367	0.4177	100	1887.974	1884.968		6820.9	1	7.062	53.3	24
	K.IYNNHL	3.5548	0.3677	100	1496.783	1496.784		5859.4	1	7.279	75	1
	K.DKNNPI	4.5859	0.2814	100	1720.85	1719.846		5507.2	1	6.327	73.1	14

	K.NNPIYN	4.168	0.425	100	1483.745	1483.741		6094.7	1	7.175	86.4	8
	H.TNYKDH	3.1475	0.3183	100	1147.59	1147.586		4643.8	1	6.748	87.5	6
	K.TNISQEI	4.6075	0.3552	100	1876.995	1876.992		6496.4	1	6.897	66.7	1
	K.TNISQE	5.8649	0.5029	100	2006.095	2005.087		8362.2	1	8.102	71.9	3
	K.KKDEES	3.7782	0.3774	100	1569.747	1569.74		5477.6	1	5.994	75	3
	K.KKDEES	5.5574	0.4789	100	2031.975	2030.967		6819.2	1	7.844	71.9	6
	K.GENILQ	3.5392	0.3457	100	1136.641	1136.639		6975.3	1	6.145	83.3	1
	K.GENILQ	3.251	0.3118	100	1292.74	1292.74		6744.4	1	5.784	80	1
	K.RLINGN	3.2878	0.2119	99.8	1347.755	1347.757		6561.2	1	4.602	75	1
	R.LINGNY	3.5159	0.308	100	1191.656	1191.656		4202.5	1	5.617	83.3	4
	K.KILLMC	3.3924	0.2642	100	1169.651	1169.65		5575	1	5.419	87.5	5
	R.IKPKDE	4.2972	0.245	99.9	2132.145	2132.144		6571.6	1	6.786	42.6	1
	R.IKPKDE	5.0836	0.453	100	2132.148	2132.144		7198.5	1	8.143	70.6	13
	K.ALGFDF	3.4332	0.2205	99.9	1024.549	1024.547		3262.9	1	5.658	87.5	32
PF14_0563		26	10.90%	741	84314	5.3	U		Annotation|Plasmodium_falciparum_TIGR|(protein codin			
	K.QNGDAI	3.3132	0.4033	100	1975.983	1975.976		5738.8	1	6.915	47.1	3
	K.KKEENS	3.911	0.368	100	1816.871	1816.868		5127.5	1	6.759	60	3
	K.KKEEN	4.808	0.2929	100	3017.374	3013.399		5214.5	8	5.784	26	3
	K.VSGEEN	4.2291	0.362	100	1889.85	1889.848		4516.8	1	6.563	68.8	9
	K.EPVEEE	4.6584	0.2888	100	2203.199	2203.195		7702.8	1	6.089	44.4	2
	K.EPVEEE	4.8965	0.4516	100	2203.2	2203.195		8086.4	1	8.01	63.9	6
PFF0430w		8	10.90%	543	61534	6.9	U		Annotation|Plasmodium_falciparum_Sanger|(protein coc			
	R.TKLPIC	4.4392	0.3232	100	2243.239	2242.237		4709.4	1	6.668	34.2	2
	K.VLLENS	4.2015	0.4169	100	2282.259	2282.258		6775.6	1	7.309	50	1
	R.SEPLGL	5.2595	0.4697	100	2032.114	2032.115		6780.4	1	8.418	61.1	5
MAL7P1.36		3	10.90%	119	14284	9.7	U		Annotation|Plasmodium_falciparum_Sanger|(protein coc			
	R.EAVNDF	4.0292	0.4116	100	1529.742	1529.739		6026.6	1	7.88	70.8	3
PF11_0161		52	10.80%	482	55803	8	U		Annotation|Plasmodium_falciparum_TIGR|(protein codin			
PF11_0165		52	10.70%	484	55927	7.4	U		Annotation|Plasmodium_falciparum_TIGR|(protein codin			
	K.YKGNEN	5.0137	0.4878	100	1888.837	1885.826		5587.9	1	8.383	82.1	21
	K.GNENFD	4.1922	0.4676	100	1594.672	1594.668		4750.3	1	7.565	79.2	6
	R.LHSGVT	3.6553	0.3504	100	1308.729	1308.728		6545	1	6.854	72.7	18
	K.LITLSE	4.9398	0.428	100	1781.902	1781.9		8286.1	1	7.89	82.1	4
	K.NSWGQ	2.5224	0.3479	99.9	1247.558	1247.556		6727.7	1	5.775	83.3	3
PFE0225w		9	10.80%	381	43032	8.3	U		Annotation|Plasmodium_falciparum_Sanger|(protein coc			
	K.GLLLSA	3.9592	0.2922	100	2152.129	2152.123		8630.9	1	6.509	50	3

	R.VCGYDT	4.5366	0.407	100	2670.173	2670.18		4895.7	1	6.764	36.9	3
	R.VCGYDT	4.3685	0.3458	100	2671.174	2670.18		8925.2	1	7.567	52.4	3
MAL13P1.236		2	10.80%	139	16847	9.3	U		Annotation|Plasmodium_falciparum_Sanger|(protein coc			
	V.VPSLIIL	2.7633	0.4421	100	1744.184	1743.155		8953.2	1	6.134	42.9	2
MAL13P1.336		29	10.70%	685	78574	9.4	U		Annotation|Plasmodium_falciparum_Sanger|(protein coc			
	K.EVVNSE	5.3123	0.4371	100	3059.607	3058.598		8349.9	1	7.287	26	1
	K.KKETEN	5.3696	0.3602	100	1934.913	1934.919		6641	1	7.072	76.7	15
	K.KKETEN	4.245	0.2332	99.9	1935.926	1934.919		5066.9	1	5.387	46.7	3
	K.ETENVE	4.0232	0.1822	99.8	1678.737	1678.73		5572.9	1	5.06	73.1	3
	K.KIDDES	5.689	0.4469	100	1872.926	1872.919		7149.8	1	9.193	80	4
	K.KVEDNV	3.245	0.4486	100	1739.84	1739.835		7069.3	1	7.194	57.1	3
PFI0630w		37	10.70%	338	39136	8.1	U		Annotation|Plasmodium_falciparum_Sanger|(protein coc			
	K.IVGWYT	4.3517	0.328	100	2833.467	2832.461		6645.5	1	6.278	32.6	3
	K.IVGWYT	4.5977	0.4326	100	2867.565	2867.547		6822.2	1	7.049	43.5	13
	T.TGSNIKI	3.1651	0.3262	99.9	2113.089	2113.097		6413.6	1	5.072	61.8	2
	R.SVIALHI	4.2169	0.2941	100	1349.81	1349.809		7802.7	1	6.683	81.8	19
PF07_0073		16	10.60%	539	62454	7	U		Annotation|Plasmodium_falciparum_Sanger|(protein coc			
	K.GIQVAG	2.5011	0.3427	99.9	845.4916	844.4867		4758.6	1	5.947	92.9	1
	K.NIMEEC	5.7257	0.4949	100	2249.969	2248.963		8685	1	8.212	73.5	9
	K.ISSQAD	4.1339	0.3685	100	1925.919	1924.911		5489.7	1	6.812	53.1	1
	K.VEQFCI	4.2701	0.3423	100	1677.849	1677.85		5861.7	1	6.603	69.2	5
PFI1545c		15	10.60%	282	32602	6.4	U		Annotation|Plasmodium_falciparum_Sanger|(protein coc			
	K.FNDYNF	3.5471	0.2973	99.8	1874.952	1873.945		4565.5	1	5.62	51.7	2
	K.FNDYNF	5.091	0.4896	100	1880.967	1880.962		6048.5	1	8.781	80	12
	K.YAMHNI	2.809	0.3748	99.9	1541.719	1541.724		7312.8	1	5.575	57.7	1
PFC0200w		9	10.60%	104	12157	10.5	U		Annotation|Plasmodium_falciparum_Sanger|(protein coc			
	K.GFGGQT	2.815	0.3697	99.9	1165.631	1165.637		8156	1	5.903	70	9
PF11_0207		119	10.50%	964	116349	5.9	U		Annotation|Plasmodium_falciparum_TIGR|(protein codin			
	K.KKEDDL	4.2963	0.2839	100	1479.747	1479.744		8277.3	1	6.056	72.7	6
	K.KKEDDL	4.0414	0.2753	100	1465.731	1465.729		8057	1	5.86	81.8	81
	K.KKEDAL	4.2621	0.3241	100	1421.734	1421.739		8223.9	1	6.318	90.9	6
	K.KKEDDL	3.829	0.238	99.9	1464.75	1464.745		7441.6	1	6.212	81.8	6
	R.KEEIKE	5.1639	0.2902	100	1671.894	1671.892		7599.3	1	6.56	87.5	1
	K.EEIKEEI	4.6164	0.0922	99.8	1537.865	1537.863		9248.3	1	5.119	86.4	6
	K.EEIKEEV	4.8547	0.0961	99.9	1518.838	1516.83		5730.8	1	5.142	81.8	12
	K.EEIKEEV	6.0603	0.1802	100	2023.116	2023.111		7122.6	1	5.98	73.3	1

PF13_0063		7	10.50%	420	46835	6.7	U		Annotation|Plasmodium_falciparum_Sanger|(protein coc				
	K.LREVVE	3.6208	0.3005	99.9	1708.946	1708.942		7048.6	3	5.684	57.7	3	
	R.GDESAH	2.8041	0.3441	99.9	1436.614	1436.616		3446.6	1	6.055	66.7	1	
	M.ATNRPD	3.457	0.2995	99.9	1838.973	1838.984		3764.9	13	5.333	50	3	
PFL1825w		7	10.50%	210	23659	9.6	U		Annotation|Plasmodium_falciparum_Sanger|(protein coc				
	K.LANHNL	3.0645	0.2923	99.9	1078.615	1078.612		7108.9	1	6.423	93.8	4	
	K.KESLYN	3.895	0.3472	100	1538.788	1538.786		5013.9	1	7.029	83.3	2	
	K.ESLYND	3.3822	0.3051	100	1417.711	1417.708		11679.3	1	5.903	72.7	1	
PFL2015w		14	10.40%	676	79801	4.9	U		Annotation|Plasmodium_falciparum_Sanger|(protein coc				
	K.NVINDD	4.3437	0.3236	100	1609.79	1609.791		5267	1	5.987	83.3	1	
	K.YKDDLD	5.41	0.3895	100	2436.192	2436.19		5027.5	1	6.851	45	6	
	K.YKDDLD	4.3971	0.4022	100	2110.031	2110.024		6607.6	1	7.36	65.6	2	
	K.YKDDLD	3.8605	0.3825	100	2117.046	2117.041		5953.4	1	6.843	43.8	1	
	K.NNHLSE	3.2253	0.2278	99.9	1217.57	1214.559		5717.3	1	4.963	77.8	3	
	K.HIQDDIL	3.7391	0.1343	99.9	1109.615	1109.614		7374.7	1	5.199	87.5	1	
PF11_0191		22	10.40%	594	69390	5.9	U		Annotation|Plasmodium_falciparum_TIGR|(protein codin				
	K.ATTFLL	4.205	0.2558	100	1951.04	1950.034		4679.1	1	5.539	56.2	14	
	K.VANLSI	4.6608	0.4736	100	3582.892	3581.86		6263.2	1	8.548	30.6	5	
	R.LISHAG	3.0983	0.4315	100	1329.795	1329.797		6129.4	1	6.653	70.8	3	
PFI0720w		1	10.30%	516	58421	8.3	U		Annotation|Plasmodium_falciparum_Sanger|(protein coc				
	I.FVTILY	4.7204	0.2445	99.8	6221.119	6218.207		3754.5	150	4.427	11.8	1	
PFD1070w		6	10.30%	390	44800	7.6	U		Annotation|Plasmodium_falciparum_Sanger|(protein coc				
	K.ALNNGV	4.1755	0.4688	100	1498.823	1498.82		7066	1	8.694	71.4	5	
	R.FLSPNT	3.3628	0.3152	99.9	2778.592	2777.56		6093	1	6.297	37.5	1	
MAL7P1.38		35	10.20%	688	78897	6.9	U		Annotation|Plasmodium_falciparum_Sanger|(protein coc				
	K.TYSSGI	4.9338	0.5051	100	2596.35	2596.349		7243.2	1	7.792	37.5	3	
	K.DLEHSP	4.088	0.2335	99.8	1847.95	1845.939		8108.6	1	4.447	46.7	1	
	K.DLEHSP	3.9903	0.4202	100	1852.957	1852.956		6484.8	1	6.732	70	11	
	K.YVYPNC	4.5793	0.4665	100	2267.988	2264.975		6217.8	1	7.941	59.4	15	
	K.NITCGT	4.3576	0.3323	100	1918.885	1917.88		7402	1	6.486	71.9	3	
	K.KNQIQN	3.7915	0.3042	100	1605.758	1605.754		5420.7	1	5.151	70.8	2	
PF14_0369		4	10.20%	235	27151	7.6	U		Annotation|Plasmodium_falciparum_TIGR|(protein codin				
	A.DKSDNS	3.8061	0.3204	100	1484.752	1484.749		5214.8	1	5.744	75	1	
	K.EFTADP	2.2362	0.3948	99.8	1287.447	1287.445		3984.8	1	6.792	70	3	
PFL0595c		7	10.20%	205	23953	8.9	U		Annotation|Plasmodium_falciparum_Sanger|(protein coc				
	R.GLEILA	4.076	0.4269	100	2412.221	2412.209		5693	1	7.602	50	7	

PFL0415w		45	10.20%	157	18452	6.2	U		Annotation|Plasmodium_falciparum_Sanger|(protein coc				
	K.TVQEVII	5.5347	0.2635	100	1790.017	1789.011		7853.9	8	5.19	46.4	10	
	K.TVQEVII	5.4258	0.3723	100	1817.084	1817.08		8991.3	1	7.164	75	29	
	K.TVQEVII	5.2686	0.5167	100	1946.184	1945.175		8995.4	1	8.413	70	6	
PF10_0100		16	10.20%	118	13737	10.2	U		Annotation|Plasmodium_falciparum_TIGR|(protein codin				
	K.KGFEVF	3.1627	0.3282	99.9	1341.722	1341.721		6223.5	1	6.108	72.7	6	
	K.GFEVPC	3.4375	0.2143	99.9	1213.629	1213.626		3684.2	1	6.123	75	10	
PFA0210c		27	10.10%	523	60196	7.6	U		Annotation|Plasmodium_falciparum_Sanger|(protein coc				
	L.KENKEE	6.3422	0.4817	100	2035.008	2033.999		7562.7	1	8.669	70.6	6	
	Y.LINGLP	3.0013	0.283	99.9	1187.681	1184.683		5320.8	1	4.855	88.9	4	
	R.INNYEN	3.9922	0.2438	99.9	1721.806	1721.8		10139.1	1	5.948	75	2	
	K.NEQNAV	4.6664	0.2823	100	1435.69	1434.688		6302.3	1	6.154	86.4	15	
PFD1035w		4	10.10%	328	38093	9.5	U		Annotation|Plasmodium_falciparum_Sanger|(protein coc				
	K.EYNIQV	3.5479	0.2922	99.9	2102.13	2102.13		5319.5	1	6.447	56.2	3	
	K.IKNPGI	3.4161	0.35	100	1784.027	1784.026		6467.2	1	6.111	63.3	1	
PF14_0068		4	10.10%	318	34129	10	U		Annotation|Plasmodium_falciparum_TIGR|(protein codin				
	K.RSNIVP	3.7217	0.3099	100	1370.766	1368.76		4003.3	1	6.018	77.3	2	
	R.MLVDMV	3.0528	0.3052	99.8	2221.065	2219.084		8031.9	2	5.205	39.5	2	
MAL13P1.299		3	10.10%	257	29644	5.7	U		Annotation|Plasmodium_falciparum_Sanger|(protein coc				
	K.LFYNKP	4.1409	0.3253	100	3044.532	3042.525		5706	1	5.538	42	3	
PFC0635c		15	10.10%	227	26948	8.3	U		Annotation|Plasmodium_falciparum_Sanger|(protein coc				
	K.IDLSNP	4.1625	0.4239	100	2802.474	2801.47		6671.6	1	6.759	36.4	15	
PF14_0576		9	10.10%	208	24133	5	U		Annotation|Plasmodium_falciparum_TIGR|(protein codin				
	R.KDHPTV	4.6063	0.4014	100	2467.127	2466.115		7621.9	1	7.905	57.5	3	
	K.DHPTVH	5.1262	0.4624	100	2339.031	2338.02		4249.3	1	8.973	44.7	3	
	K.DHPTVH	5.3929	0.5163	100	2339.032	2338.02		6482.8	1	8.927	65.8	3	
PF08_0039		11	10.10%	139	16382	10.2	U		Annotation|Plasmodium_falciparum_Sanger|(protein coc				
	K.DTILDI	3.9544	0.3411	100	1640.88	1639.877		5915.9	1	6.859	73.1	11	
PFI0220w		5	10.10%	129	15325	5	U		Annotation|Plasmodium_falciparum_Sanger|(protein coc				
	K.FDLDYI	4.0654	0.4362	100	1694.777	1693.771		7685	1	8.077	87.5	5	
PF13_0233		51	10.00%	818	92277	8.2	U		Annotation|Plasmodium_falciparum_Sanger|(protein coc				
	N.HTNIPC	2.7987	0.3361	99.9	1456.755	1456.762		4647.2	18	5.106	59.1	2	
	K.LLNPNS	4.4574	0.3539	100	2885.477	2883.459		3556.5	1	5.596	31	3	
	K.NNSLEQ	5.0235	0.3863	100	2151.132	2149.096		7245.8	1	7.34	67.6	45	
	R.GDLVEV	3.0946	0.289	99.8	2895.565	2894.552		7577.4	1	6.055	30	1	
PFI0370c		13	10.00%	279	33126	6.8	U		Annotation|Plasmodium_falciparum_Sanger|(protein coc				

	K.TYASEL	3.8836	0.352	100	1563.866	1563.863		9466.1	1	6.853	69.2	9	
	R.VEESAF	3.6007	0.3317	100	1604.835	1604.829		7326.3	1	6.485	61.5	4	
MAL8P1.41		2	10.00%	269	32164	4.4	U		Annotation|Plasmodium_falciparum_Sanger|(protein coc				
	K.NVENQI	3.756	0.3754	100	3211.581	3210.574		7623.3	1	5.594	29.8	2	
PFF1345w		112	9.90%	1147	132838	5.3	U		Annotation|Plasmodium_falciparum_Sanger|(protein coc				
	K.DLNENV	4.4657	0.5347	100	2304.177	2304.17		9185.8	1	8.542	60.5	8	
	K.YEGIEQ	4.1461	0.3266	99.9	2476.345	2476.332		7903.2	1	5.839	31.6	5	
	K.YEGIEQ	4.643	0.4698	100	2477.346	2476.332		4880.6	1	8.157	60.5	18	
	R.GNNDVY	3.4291	0.4237	100	1163.545	1163.545		5138.6	1	7.037	80	3	
	R.KVQLEA	5.2484	0.541	100	1875.1	1875.097		10146	1	9.26	82.1	4	
	K.VQLEAL	5.3403	0.3686	100	1750.016	1747.002		7631.3	1	7.84	80.8	33	
	K.LLNDEK	3.1734	0.2221	99.8	1168.668	1168.669		5367.5	2	4.938	83.3	1	
	K.WFEPVL	3.9047	0.2716	100	1374.814	1371.804		4828.1	1	5.812	85	35	
	R.ILSVIN	4.4998	0.4294	100	2988.555	2988.576		5954.4	1	6.417	28	5	
PF08_0095		49	9.90%	706	83374	7.1	U		Annotation|Plasmodium_falciparum_Sanger|(protein coc				
	R.NAVLIL	4.1612	0.4529	100	1662.985	1662.979		11770.1	1	7.357	78.6	6	
	K.ECVDND	3.9304	0.4053	100	2902.412	2902.419		7630	1	7.254	33.3	1	
	R.LNFLVL	4.1553	0.4657	100	1263.773	1262.77		6457.4	1	7.695	90	17	
	K.LLQNIH	5.2318	0.4621	100	2355.24	2354.237		8542.9	1	8.771	66.7	25	
PFI0195c		48	9.90%	426	50301	4.9	U		Annotation|Plasmodium_falciparum_Sanger|(protein coc				
	K.VSEGQE	4.9394	0.3511	100	2161.114	2160.109		5266.2	1	6.549	45.8	9	
	K.VSEGQE	4.4555	0.4205	100	2182.164	2181.16		7564.2	1	7.783	50	31	
	R.TVDLSN	3.5176	0.256	99.9	1238.672	1238.671		4776.7	1	6.638	85	1	
	K.KNDKNE	4.277	0.3578	100	1388.741	1388.739		7088.7	1	6.777	86.4	7	
PFL1500w		13	9.90%	213	24424	6.8	U		Annotation|Plasmodium_falciparum_Sanger|(protein coc				
	R.KIYDNI	4.5712	0.4687	100	2491.228	2491.224		7984.3	1	7.358	47.5	10	
	K.IYDNIL	3.4792	0.3293	100	2384.184	2384.181		5079.7	1	5.789	47.4	3	
PFB0865w		1	9.90%	101	11714	9.6	U		Annotation|Plasmodium_falciparum_TIGR|(protein codin				
	R.HCNLLL	2.5906	0.323	99.9	1182.643	1182.642		6937.7	2	6.511	72.2	1	
PF10_0077		10	9.80%	543	63110	9	U		Annotation|Plasmodium_falciparum_TIGR|(protein codin				
	K.NSNDVN	4.7117	0.3945	100	2369.11	2368.106		8992.6	1	6.541	52.6	3	
	K.TKEDHN	4.9078	0.3725	100	1908.971	1908.978		6324.6	1	6.973	66.7	3	
	K.NANDPD	3.6813	0.3339	100	1941.941	1939.938		4960	1	5.494	50	4	
PF11_0145		11	9.80%	356	42441	6.1	U		Annotation|Plasmodium_falciparum_TIGR|(protein codin				
	R.GFGHIG	3.6898	0.4404	100	1989.969	1989.961		6651.5	1	8.028	62.5	1	
	K.NIAFIYD	4.4606	0.4871	100	2224.207	2224.203		6424.9	1	8.346	64.7	10	

PF07_0040		34	9.80%	368	42358	9.1	U		Annotation|Plasmodium_falciparum_Sanger|(protein coc			
	R.QFDDIV	5.2799	0.5161	100	1971.001	1969.996		6220	1	8.47	71.4	32
	R.GCIPLA	3.6669	0.3908	100	2265.171	2264.167		5353.9	1	7.08	60	2
PFL0720w		36	9.80%	245	27379	4.9	U		Annotation|Plasmodium_falciparum_Sanger|(protein coc			
	K.AENCCF	5.2902	0.4876	100	2565.241	2562.235		9048.2	1	8.998	56.5	34
	G.GIAGFG	2.682	0.3881	99.9	1166.599	1166.596		7549.3	1	6.818	75	2
PFE1150w		41	9.70%	1419	162252	8.8	U		Annotation|Plasmodium_falciparum_Sanger|(protein coc			
	R.SVFYQD	2.8605	0.345	99.8	1939.856	1939.857		6002.1	1	5.991	59.4	3
	K.TSLLYN	3.3995	0.313	99.9	2405.254	2404.252		5072.8	1	6.678	32.5	1
	K.DLEAME	6.4302	0.4801	100	2486.004	2484.999		9666.2	1	10.003	65.8	6
	K.KVLIHD	5.8498	0.4614	100	2734.45	2733.446		6212.4	1	8.014	38.5	2
	K.VLIHDF	4.9183	0.5429	100	2606.359	2605.351		7353.5	1	8.771	54.3	10
	K.VLIHDF	5.129	0.5736	100	2613.376	2612.368		9012.6	1	8.64	38	5
	K.ILILDEA	3.9264	0.2825	100	1531.818	1531.822		8613.7	1	6.413	69.2	6
	T.PGVLSA	3.1244	0.3694	100	1070.617	1070.618		6382.9	1	6.495	83.3	1
	K.FGREDA	3.2826	0.2748	99.9	1535.784	1535.782		6457	1	6.254	70.8	3
	K.ILLLDE	3.9245	0.4222	100	1834.931	1834.929		5000.8	1	7.813	65.6	4
PF14_0390		87	9.70%	227	26306	3.6	U		Annotation|Plasmodium_falciparum_TIGR|(protein codin			
	K.NSNTVD	6.1738	0.4464	100	2413.101	2409.091		11155	1	8.142	64.3	87
MAL7P1.159		24	9.70%	175	20584	5.1	U		Annotation|Plasmodium_falciparum_Sanger|(protein coc			
	K.ILLISL	4.7024	0.4242	100	1820.032	1819.004		3733.4	1	7.809	62.5	24
MAL7P1.202		57	9.60%	1229	145508	5.3	U		Annotation|Plasmodium_falciparum_Sanger|(protein coc			
	K.ILIHNN	3.8256	0.4062	100	2921.502	2920.493		6321.1	1	7.74	37	9
	K.WLCPFF	5.0002	0.4498	100	2379.165	2376.156		7418.5	1	8.113	70.6	23
	K.TLTDEC	3.5324	0.387	100	2890.507	2889.496		5062.3	1	6.754	39.6	7
	K.LFKHDE	3.9492	0.1837	99.8	1673.854	1672.848		7183.8	1	4.779	73.1	3
	R.KGFFDD	3.6559	0.4046	100	1301.652	1301.653		7238.4	1	7.22	85	4
	K.GFFDDII	3.5459	0.3995	100	1181.581	1180.575		5568.5	1	6.552	83.3	7
	K.YQNTYN	2.9197	0.3243	99.9	1463.726	1463.724		7592.4	1	5.888	63.6	1
	K.YLVDAD	4.348	0.4122	100	1686.81	1686.809		6925.9	1	6.949	80.8	3
PFL1880w		55	9.60%	792	92092	8.7	U		Annotation|Plasmodium_falciparum_Sanger|(protein coc			
	K.NSSNIY	4.4971	0.353	100	1525.751	1525.751		5224.7	1	7.195	72.7	26
	R.NIIPQT	3.7598	0.2856	100	1878.906	1875.9		6900.6	1	5.727	57.1	12
	I.PQTEMH	3.0558	0.2927	99.8	1521.654	1521.655		4782.7	1	6	59.1	3
	K.KYDVVF	3.5618	0.3774	100	2100.112	2099.097		7881.2	1	5.966	53.1	3
	K.ELVGDM	4.4092	0.4123	100	2180.195	2179.191		4946.3	1	6.794	44.4	5

	K.KHNLNL	3.931	0.3622	100	1596.867	1596.875		6443.1	1	6.186	70.8	6
PFC0275w		28	9.60%	653	74689	8.5	U		Annotation|Plasmodium_falciparum_Sanger|(protein coc			
	R.YLENAV	3.8803	0.4124	100	2921.392	2919.42		10836.5	1	7.615	43.5	11
	R.VLFLLP	3.2331	0.2944	99.8	2190.149	2189.149		4386.7	1	5.252	50	1
	K.NDITAA	4.8648	0.3868	100	2307.154	2306.154		6592.7	1	6.658	42.1	16
MAL13P1.225		10	9.60%	157	18630	9.8	U		Annotation|Plasmodium_falciparum_Sanger|(protein coc			
	R.KDHFVS	3.9564	0.3306	100	1713.985	1713.973		8283.1	1	6.049	60.7	10
PF11_0415		21	9.60%	167	18602	5.7	U		Annotation|Plasmodium_falciparum_TIGR|(protein codin			
	K.DAHMNS	4.6512	0.5021	100	1859.824	1859.835		6480.7	1	8.88	60	9
	K.DAHMNS	5.4959	0.573	100	1859.829	1859.835		8618	1	9.314	76.7	12
PFL1155w		42	9.50%	389	45877	9.3	U		Annotation|Plasmodium_falciparum_Sanger|(protein coc			
	K.NRENFN	4.6094	0.4019	100	2387.129	2387.126		5763.7	1	7.526	55.6	3
	R.ENFNEC	4.3181	0.4538	100	2116.985	2116.982		6458.8	1	7.361	59.4	1
	K.HHLLPF	4.7294	0.3752	100	2184.065	2183.06		7020.8	1	6.759	47.1	38
		15	9.50%	357	39219	5.2	U		no description			
	K.NKYEDE	2.9269	0.2513	99.8	1308.657	1308.655		5918.4	1	4.991	83.3	1
	K.ADTLTD	2.8957	0.3007	99.8	1407.714	1407.712		7217	1	5.116	77.3	2
	R.NLDLDS	4.4547	0.2712	100	1332.738	1329.727		5961.4	1	6.08	81.8	12
PFC0166w		1	9.50%	179	21698	8.8	U		Annotation|Plasmodium_falciparum_Sanger|(protein coc			
	K.NLPSFM	3.1247	0.3622	99.8	1942.128	1942.102		7291.4	1	6.242	46.9	1
MAL7P1.10		20	9.50%	105	12503	4.6	U		Annotation|Plasmodium_falciparum_Sanger|(protein coc			
	K.KADFIN	3.7613	0.3545	100	1250.667	1250.665		8539.5	1	6.662	88.9	20
PFA0310c		68	9.40%	1228	139414	7.2	U		Annotation|Plasmodium_falciparum_Sanger|(protein coc			
	K.TEIGHI	5.5002	0.5129	100	2816.478	2816.481		6625.4	1	9.145	47.9	2
	K.TGTLTT	4.0242	0.3794	100	2070.052	2069.049		6099.6	1	7.543	55.9	6
	K.FGDSTE	3.1019	0.4289	100	1202.607	1202.606		8405.5	1	7.941	85	3
	H.FVHNFD	3.565	0.3885	100	1564.808	1564.816		8006	1	6.49	75	6
	T.STHNNN	4.6395	0.5075	100	2860.21	2860.205		5896.3	1	7.802	52.1	3
	K.EASDIV	5.4986	0.5305	100	2198.139	2198.149		10223	1	9.111	63.2	46
	K.EASDIV	3.3187	0.3039	99.9	2520.287	2519.263		6299	1	5.707	34.1	2
PF14_0589		89	9.40%	1090	128070	6.9	U		Annotation|Plasmodium_falciparum_TIGR|(protein codin			
	K.WYSFW	3.9265	0.3577	100	1938.864	1938.87		9550.8	1	6.452	53.8	30
	K.KELIEE	5.426	0.3572	100	1754.951	1751.948		7493.1	1	6.478	76.9	1
	K.KELIEE	4.9288	0.2689	100	1866.001	1866.009		6690.2	1	6.97	67.9	6
	K.ELIEEN	3.1433	0.2835	99.8	1751.944	1751.948		4845.8	11	5.414	53.8	1
	K.ELEIIP	3.2932	0.3746	100	2714.406	2713.397		3641.1	1	5.52	47.5	1

	I.PPQHVN	4.0631	0.4872	100	2102.023	2102.025		5898.1	1	7.886	60	9
	R.LNTDSH	3.3371	0.3908	100	1546.751	1546.75		6667.5	1	6.849	69.2	3
	R.LLHPISF	5.1873	0.4529	100	1942.091	1942.088		4480.6	1	7.889	73.3	34
	K.VEELNS	3.4344	0.3095	99.9	2719.357	2718.369		7717.8	1	5.374	34.1	4
PF11_0320		3	9.40%	170	19992	4.7	U		Annotation|Plasmodium_falciparum_TIGR|(protein codin			
	K.DVQYVT	3.9252	0.3548	100	2004.913	2003.906		7239.9	1	6.97	63.3	3
MAL13P1.271		38	9.40%	181	19124	7.2	U		Annotation|Plasmodium_falciparum_Sanger|(protein coc			
	K.FSGLST	5.2306	0.322	100	1843.014	1839.002		5880.9	1	6.55	62.5	38
PFC0135c		127	9.30%	1254	147938	6	U		Annotation|Plasmodium_falciparum_Sanger|(protein coc			
	K.LLDNVV	4.8443	0.3371	100	1442.813	1442.811		6346.2	1	7.603	83.3	27
	R.SVSIIL	3.251	0.3623	100	1683.91	1683.91		5167.3	1	6.143	64.3	1
	K.FYGLQI	3.233	0.3677	100	1769.873	1768.869		3180.4	1	5.908	65.4	2
	K.KIENVT	4.4529	0.3326	99.9	2999.663	2997.677		6881.7	1	6.372	31	5
	K.KIENVT	4.4822	0.4577	100	3029.792	3025.746		6102.4	1	7.97	42	17
	K.IENVTC	3.9243	0.4401	100	2897.663	2897.651		6937.1	1	6.964	39.6	5
	R.ITQQFLI	3.2556	0.2963	99.9	1495.931	1495.937		8406	1	5.528	63.6	3
	K.HVQNFL	4.2423	0.3925	100	1946.974	1946.972		5696.4	1	8.257	56.7	6
	K.HVQNFL	4.4546	0.38	100	1946.976	1946.972		6809.9	1	7.026	46.7	6
	K.HVQNFL	5.5472	0.4702	100	1947.98	1946.972		9982.5	1	7.576	73.3	53
	K.EFATNQ	3.2443	0.3045	99.9	2426.124	2426.122		5235.4	1	5.674	36.8	2
PF14_0261		1	9.30%	377	42641	7.8	U		Annotation|Plasmodium_falciparum_TIGR|(protein codin			
	L.PLSLIN	4.6722	0.2565	99.9	3971.069	3964.068		8663.6	85	4.746	18	1
PF10_0366		65	9.30%	301	33726	9.7	U		Annotation|Plasmodium_falciparum_TIGR|(protein codin			
	R.YFPTQA	3.9281	0.4188	100	1480.727	1480.727		3723.3	1	6.494	81.8	47
	F.PTQAFN	3.0147	0.3145	99.9	1170.594	1170.595		6720.3	1	7.017	83.3	12
	R.GAGGAI	3.3437	0.2624	99.8	1680.906	1679.901		10253.6	1	5.01	56.7	6
PFF1130c		3	9.30%	258	30431	9	U		Annotation|Plasmodium_falciparum_Sanger|(protein coc			
	K.HDENLY	4.2795	0.2966	99.9	2949.447	2947.445		7942.1	1	5.089	29.3	3
PF13_0096		14	9.20%	640	74636	7.9	U		Annotation|Plasmodium_falciparum_Sanger|(protein coc			
	K.GTHAYT	3.1252	0.4141	100	1245.615	1243.607		4915.4	1	7.597	75	3
	K.NIIVVLS	3.4191	0.2243	99.9	1247.825	1247.824		8097.3	1	5.797	85	3
	K.GVVSPH	4.8062	0.4319	100	1912.002	1910.998		9107.8	1	8.58	70.6	5
	K.YNLIAN	3.9073	0.3247	100	2154.134	2154.117		6221.8	1	6.918	61.1	3
PFL0900c		59	9.20%	590	68436	6.9	U		Annotation|Plasmodium_falciparum_Sanger|(protein coc			
	K.FGHYQC	3.0365	0.3739	100	1741.811	1741.812		5791	1	6.588	57.7	2
	R.VNHVGI	3.8739	0.4453	100	2214.143	2213.148		7881.7	1	6.691	47.2	1

	K.LEYVGE	5.1222	0.4668	100	2418.283	2416.273		6077.5	1	8.587	57.5	56	
PF11_0245		15	9.20%	555	62882	5.2	U		Annotation|Plasmodium_falciparum_TIGR|(protein codin				
	K.LVLNDD	5.3924	0.4492	100	2383.156	2382.146		10118.7	1	8.087	63.2	3	
	R.KGEFET	3.5994	0.3432	100	1199.57	1199.57		6344.7	1	6.407	88.9	9	
	K.VMNVFL	4.8235	0.3146	100	2422.187	2421.176		5917.6	1	6.392	52.5	3	
PFL1550w		4	9.20%	498	55597	7.8	U		Annotation|Plasmodium_falciparum_Sanger|(protein coc				
	R.IVIATGS	2.9138	0.3977	99.9	1735.137	1735.147		5281.2	1	6.062	35	1	
	K.AEEEGY	3.6524	0.2994	99.9	1966.99	1967.001		8209.9	1	5.537	56.2	2	
	K.IIYAHPT	3.5001	0.3677	100	1538.879	1538.888		5833.7	1	6.214	66.7	1	
PFI0190w		2	9.20%	131	15527	11.1	U		Annotation|Plasmodium_falciparum_Sanger|(protein coc				
	K.YCVQIA	3.2305	0.2899	99.9	1412.705	1412.707		6057.8	1	5.392	72.7	2	
PF11_0188		34	9.10%	930	108465	6.7	U		Annotation|Plasmodium_falciparum_TIGR|(protein codin				
	K.FSSFIN	3.8736	0.3596	100	1560.85	1560.847		6648.1	1	6.516	79.2	15	
	K.NVVENK	3.5926	0.3642	100	1480.694	1480.692		5629.9	1	6.673	63.6	6	
	R.TSDNIK	3.3408	0.3092	100	1325.704	1325.703		7226.3	1	5.989	68.2	1	
	K.ELNEEH	4.8585	0.3372	100	1807.948	1807.944		8923	1	7.233	71.4	4	
	K.KQSIFF	4.1913	0.3008	100	1511.789	1511.786		7177.1	1	5.762	72.7	6	
	K.LLVQQL	3.7782	0.4289	100	2386.243	2386.263		9572.8	1	6.97	42.5	2	
PFE0445c		8	9.10%	298	34646	5.6	U		Annotation|Plasmodium_falciparum_Sanger|(protein coc				
	K.KFLDNI	4.647	0.3513	100	3296.602	3294.581		9418.5	1	6.315	30.8	2	
	K.FLDNII	4.7733	0.3884	100	3167.499	3166.486		6082.4	1	6.971	34	2	
	K.FLDNII	3.5069	0.3781	100	3189.54	3187.537		4418.5	1	6.965	30	4	
PF10_0047		23	9.00%	880	106298	8.7	U		Annotation|Plasmodium_falciparum_TIGR|(protein codin				
	K.ASHALN	3.2021	0.3737	100	1481.776	1481.771		6045.1	1	6.73	65.4	3	
	K.ENVDHD	3.6456	0.4127	100	1832.809	1832.805		4950	1	8.038	64.3	2	
	K.TKDELY	4.9073	0.428	100	1582.802	1582.804		7119.2	1	8.445	83.3	7	
	K.NIKDEF	4.327	0.1482	99.8	1348.751	1348.748		9667.8	2	5.251	67.5	1	
	K.NIKDEF	3.3358	0.2453	99.9	1357.775	1355.765		7189.8	1	5.034	80	1	
	K.IMKEEY	5.4356	0.4072	100	1882.965	1882.966		9987	1	8.111	78.6	1	
	K.IMKEEY	7.0512	0.3374	100	3133.63	3132.625		8891.2	1	6.579	42	6	
	K.IMKEEY	5.359	0.2994	100	3133.635	3132.625		10238.8	1	5.14	30.7	2	
PF11_0362		20	9.00%	689	79932	7.1	U		Annotation|Plasmodium_falciparum_TIGR|(protein codin				
	K.KAHLNF	4.6303	0.4042	100	1757.906	1756.898		7556.8	1	7.69	75	9	
	K.AHLNFD	3.8782	0.3149	100	1628.806	1628.803		7971.5	4	5.556	61.5	3	
	R.LFVPEP	4.188	0.3354	100	2236.163	2235.156		4096.3	1	6.322	37.5	2	
	R.LFVPEP	3.7695	0.401	100	2236.164	2235.156		6973.6	1	6.637	55.6	4	

	K.HPDDQP	4.6979	0.4178	100	3243.651	3241.636		4868	1	6.317	26.9	2
PF14_0191		81	9.00%	509	59693	5	U		Annotation|Plasmodium_falciparum_TIGR|(protein codin			
	R.VHNDEK	5.3139	0.3716	100	2162.083	2161.078		6944.5	1	7.93	47.2	6
	R.VHNDEK	4.9071	0.4042	100	2162.085	2161.078		6229.8	1	7.976	72.2	3
	R.VHNDEK	4.3463	0.4362	100	2419.223	2418.215		7658.2	1	7.753	37.5	9
	R.VHNDEK	6.2441	0.5047	100	2419.225	2418.215		7217.1	1	8.952	46.2	15
	R.VHNDEK	4.8083	0.3365	100	2419.226	2418.215		7528.3	1	6.922	50	9
	R.YNELLN	5.0748	0.3818	100	1759.911	1758.907		10600.3	1	6.614	92.3	33
	K.NILQPVI	2.9948	0.3365	99.9	1233.796	1233.794		3670.2	1	6.377	75	6
PF14_0127		6	9.00%	410	47970	8.2	U		Annotation|Plasmodium_falciparum_TIGR|(protein codin			
	K.EDVAHV	4.3477	0.3461	100	4362.205	4360.134		9165.7	1	5.88	21.5	6
PF11_0186		4	9.00%	200	23454	4.8	U		Annotation|Plasmodium_falciparum_TIGR|(protein codin			
	K.LPVDAY	4.1224	0.3572	100	2096.053	2096.059		8582.9	1	7.212	61.8	4
PF11_0511		5	9.00%	188	22912	9.4	U		Annotation|Plasmodium_falciparum_TIGR|(protein codin			
	K.LLDLVE	4.5828	0.5274	100	2120.111	2120.11		7537.8	1	8.932	62.5	5
PF10_0337		2	9.00%	178	20248	8.6	U		Annotation|Plasmodium_falciparum_TIGR|(protein codin			
	K.NIGITEA	3.2546	0.3025	99.9	1812.992	1812.986		5415.6	1	5.625	56.7	2
PFF0305c		2	9.00%	155	17815	6	U		Annotation|Plasmodium_falciparum_Sanger|(protein coc			
	K.ANWSPA	3.263	0.4284	100	1690.823	1689.817		6274.4	1	7.793	69.2	2
PF08_0076		4	9.00%	144	16285	10.3	U		Annotation|Plasmodium_falciparum_Sanger|(protein coc			
	K.KTAVAV	3.3756	0.4521	100	1259.734	1259.732		3646.8	1	8.267	66.7	3
	K.TAVAVA	2.5626	0.4461	99.8	1132.642	1131.637		4215.3	2	7.049	59.1	1
PF14_0072		7	9.00%	134	15462	5.7	U		Annotation|Plasmodium_falciparum_TIGR|(protein codin			
	K.VHIMEG	4.1236	0.4276	100	1369.699	1369.697		6407.5	1	8.284	81.8	7
PF08_0113		37	8.90%	1053	122999	7.1	U		Annotation|Plasmodium_falciparum_Sanger|(protein coc			
	R.FLEENIN	3.8648	0.1727	99.8	1557.867	1557.864		7956.8	1	4.8	87.5	1
	K.IDNFLE	5.7358	0.3939	100	2720.304	2719.304		7289.5	1	7.762	54.8	16
	R.FCNNNK	3.3569	0.2383	99.9	1516.721	1516.718		5895.2	1	4.571	72.7	1
	K.HVILTA	4.2971	0.3858	100	1794.067	1793.062		8238.6	1	7.422	70	5
	R.HIYNNL	4.8434	0.4127	100	2084.052	2083.044		2718.1	2	7.31	56.2	11
	K.SSNDLV	4.4425	0.3373	100	1461.782	1461.78		7738.6	1	6.625	76.9	3
PF14_0327		17	8.90%	628	71743	8.4	U		Annotation|Plasmodium_falciparum_TIGR|(protein codin			
	C.SLNHCA	4.566	0.4626	100	2291.059	2290.056		6553.4	1	8.608	55.3	9
	R.MCDIGE	3.4707	0.3129	99.9	2585.232	2583.196		4826	1	5.3	40.5	2
	K.GYVNH	3.6713	0.5774	100	1795.73	1795.728		4734.5	1	9.283	65.4	3
	K.GYVNH	4.026	0.4658	100	1795.73	1795.728		5733.8	1	8.838	69.2	3

PFI1260c		9	8.90%	449	51376	6.3	U		Annotation|Plasmodium_falciparum_Sanger|(protein coc			
	K.CVQTYF	4.3269	0.3748	100	2573.293	2572.291		5884	1	6.662	47.7	6
	R.SYNIPL	4.0591	0.3416	100	1807.031	1807.031		5733.3	1	7.693	65.6	3
PFC0170c		25	8.90%	448	50990	5.6	U		Annotation|Plasmodium_falciparum_Sanger|(protein coc			
	K.LFDIGE	4.2718	0.3566	100	1650.869	1649.864		6792.4	1	6.784	85.7	2
	K.YNGVLV	2.4027	0.3432	99.9	920.5582	920.557		3758.6	1	6.244	92.9	1
	K.VNISLE	5.1896	0.3755	100	2026.061	2025.054		7930	1	7.393	65.6	22
PFC0520w		15	8.90%	304	35104	5.4	U		Annotation|Plasmodium_falciparum_Sanger|(protein coc			
	K.NQTIAV	5.0143	0.4314	100	3015.603	3014.58		7865.4	1	6.479	34.6	2
	K.NQTIAV	4.073	0.4714	100	3042.648	3042.648		5681.8	1	7.866	34.6	13
PFE0730c		23	8.90%	236	26205	7.3	U		Annotation|Plasmodium_falciparum_Sanger|(protein coc			
	K.NYIVDF	4.0604	0.4158	100	2636.303	2635.294		6622.6	1	6.964	45	23
PFI1175c		5	8.90%	202	22979	9.5	U		Annotation|Plasmodium_falciparum_Sanger|(protein coc			
	K.VQVDDI	4.8993	0.3965	100	2120.053	2120.051		6714.5	1	7.371	58.8	5
MAL13P1.274		14	8.80%	658	76889	5.2	U		Annotation|Plasmodium_falciparum_Sanger|(protein coc			
	K.MPYYET	3.3319	0.3253	99.9	1571.806	1571.803		9061.5	1	6.121	75	3
	K.FLPLAY	2.8409	0.3292	99.9	1177.702	1177.699		3265.1	1	5.339	88.9	6
	K.NIFICHG	3.478	0.3652	100	1363.755	1363.746		7045.7	1	6.326	77.3	1
	K.GNSIKP	4.6604	0.3414	100	2589.316	2589.31		6448.6	1	7.172	36.4	4
PF10_0257		60	8.80%	420	50561	9	U		Annotation|Plasmodium_falciparum_TIGR|(protein codin			
	K.YTSWAD	5.9919	0.4966	100	2559.142	2555.126		6691.3	1	9.665	60.5	40
	T.SWADIV	4.6434	0.4597	100	2292.019	2291.015		8037.8	1	8.946	70.6	1
	R.EKIDDII	4.9424	0.4459	100	2007.183	2007.171		8236.3	1	8.816	68.8	19
PFE1140c		3	8.80%	147	17389	8.9	U		Annotation|Plasmodium_falciparum_Sanger|(protein coc			
	K.NEILWP	3.4419	0.3827	100	1675.954	1672.948		6657	1	6.286	66.7	3
PFC0710w		23	8.70%	380	45232	6.3	U		Annotation|Plasmodium_falciparum_Sanger|(protein coc			
	K.INLNFQI	4.7439	0.4165	100	2727.404	2725.395		2776.4	1	7.16	52.3	22
	K.ISNIFIN	3.2088	0.2926	100	1211.733	1211.741		5533.1	1	6.824	88.9	1
PF11_0303		19	8.70%	380	45134	6.6	U		Annotation|Plasmodium_falciparum_TIGR|(protein codin			
	R.NDLLQS	4.0977	0.3717	100	1434.758	1434.748		7876	1	6.462	86.4	2
	R.NDLLQS	4.1311	0.3778	100	1569.865	1569.86		7432.7	1	6.548	79.2	8
	K.LSESAE	4.4116	0.3586	100	2252.12	2251.109		8541.2	1	7.004	57.9	9
PFL0780w		2	8.70%	367	41234	7.3	U		Annotation|Plasmodium_falciparum_Sanger|(protein coc			
	K.NIITLA	3.1161	0.3348	99.9	2223.117	2222.114		4449	1	5.145	42.1	1
	K.KTWEEL	4.1868	0.2839	100	1538.821	1538.818		9749.3	1	7.005	72.7	1
PF14_0148		3	8.70%	322	37461	9.2	U		Annotation|Plasmodium_falciparum_TIGR|(protein codin			

	K.VVIVGQ	3.0023	0.2791	99.8	1382.739	1382.743		7310.9	1	6.466	72.7	2
	K.ILAQHN	3.0484	0.3637	99.9	1915.113	1915.099		6528	1	6.095	46.7	1
PF14_0275		2	8.70%	219	26164	7.8	U		Annotation|Plasmodium_falciparum_TIGR|(protein codin			
	R.REENED	4.848	0.4558	100	2314.048	2314.055		5239.7	1	8.235	66.7	2
PFL0095c		5	8.70%	207	23678	8.5	U		Annotation|Plasmodium_falciparum_Sanger|(protein coc			
	R.AFYESL	5.0073	0.4246	100	2052.075	2051.07		10321.6	1	8.124	58.8	5
PF10_0154		137	8.60%	324	39030	5.5	U		Annotation|Plasmodium_falciparum_TIGR|(protein codin			
	R.AFLEYL	5.8737	0.385	100	1775.928	1772.914		10280.7	1	6.866	82.1	128
	R.AFLEYL	3.6943	0.3787	100	2162.135	2161.126		10271.2	1	7.22	47.1	6
	K.YPFSWF	3.322	0.3147	100	1303.628	1301.621		6545.7	2	5.807	77.8	3
MAL13P1.130		23	8.60%	303	33980	5	U		Annotation|Plasmodium_falciparum_Sanger|(protein coc			
	-.MFFTYV	3.6504	0.227	99.8	1858.923	1855.917		6922.1	1	6.364	56.7	11
	T.YVVRPG	3.1802	0.3182	99.9	1329.693	1329.692		5532.5	1	5.451	77.3	1
	Y.VVRPGE	3.0366	0.2584	99.8	1166.629	1166.628		5643.7	1	5.82	75	8
	K.NPLGML	2.8533	0.3158	99.9	1174.642	1174.641		5409.6	1	6.65	94.4	3
PFL0565w		2	8.60%	244	28062	9	U		Annotation|Plasmodium_falciparum_Sanger|(protein coc			
	R.RVNYYE	4.5927	0.4112	100	2421.326	2420.319		5184.6	1	7.275	38.8	2
PFC0730w		5	8.60%	221	26041	9.6	U		Annotation|Plasmodium_falciparum_Sanger|(protein coc			
	K.ISQTAT	6.1887	0.5692	100	2057.1	2057.106		7559.3	1	9.993	66.7	5
MAL13P1.135		6	8.60%	209	24366	8	U		Annotation|Plasmodium_falciparum_Sanger|(protein coc			
	R.IAFSLID	2.8406	0.3082	99.9	1257.714	1257.721		8463.8	1	5.697	75	3
	R.GMYEHY	2.3941	0.3056	99.8	891.4156	891.4147		4677.9	2	4.903	83.3	3
PFL1520w		15	8.60%	139	16539	5.3	U		Annotation|Plasmodium_falciparum_Sanger|(protein coc			
	K.QEFIDIV	3.8914	0.3284	100	1510.801	1509.795		6612	1	6.518	81.8	15
PFL0930w		171	8.50%	1997	232949	6.4	U		Annotation|Plasmodium_falciparum_Sanger|(protein coc			
	K.ENVNEN	3.9155	0.425	100	1971.039	1971.037		4336.5	1	6.797	59.4	1
	K.AESVIIH	4.9109	0.4478	100	1751.036	1751.037		6655.6	1	7.216	70	2
	V.IGVLLD	4.2758	0.3202	100	2457.385	2456.384		5839	1	5.994	50	1
	R.ANEGYE	4.0667	0.3901	100	1792.894	1792.894		6465.8	1	6.874	53.3	1
	K.NFHEFS	5.7999	0.3597	100	2505.088	2501.076		6510.1	1	8.969	50	36
	K.ENNFYE	4.8149	0.3616	100	2041.026	2040.019		6994.3	1	6.813	70	43
	K.HSPTAY	4.343	0.4846	100	1954.965	1953.961		5458.8	1	7.722	56.2	5
	K.VIDFYIE	5.0096	0.4784	100	2249.231	2247.225		6714.6	1	7.985	67.6	40
	K.LENHKL	2.7666	0.2841	99.8	1266.655	1266.663		4908.4	1	5.18	83.3	2
	K.NNVYIEI	5.8397	0.4589	100	1996.042	1995.038		9378	1	8.745	73.3	40
MAL13P1.121		7	8.50%	565	66200	6.2	U		Annotation|Plasmodium_falciparum_Sanger|(protein coc			

	R.TTPGLV	2.9176	0.4066	100	1246.718	1246.716		3798.6	1	5.706	75	2
	R.YFSGEK	5.1814	0.5341	100	1811.811	1810.808		9409.7	1	8.73	88.5	3
	K.SFIYDN	2.9991	0.3968	100	1535.768	1534.762		8178.1	1	6.629	72.7	1
	R.KVVRPE	3.326	0.2436	99.9	1305.765	1305.76		6092.4	1	5.101	85	1
PF14_0560		2	8.50%	353	42068	6.2	U		Annotation|Plasmodium_falciparum_TIGR|(protein codin			
	K.NDILSP	4.8848	0.3773	100	3524.766	3523.748		8687.9	1	7.013	34.5	2
MAL13P1.172		7	8.50%	260	30891	8.4	U		Annotation|Plasmodium_falciparum_Sanger|(protein coc			
	K.YSATFL	3.9843	0.3769	100	2555.287	2553.285		6618.8	1	7.042	47.6	7
PFI0230c		8	8.50%	189	22515	9.3	U		Annotation|Plasmodium_falciparum_Sanger|(protein coc			
	R.TKEDIE	3.7208	0.401	100	1941.956	1941.957		5083.6	1	7.349	53.3	1
	R.TKEDIE	4.5526	0.4643	100	1941.958	1941.957		4944.3	1	7.944	76.7	7
PF14_0632		18	8.40%	1172	132766	5.3	U		Annotation|Plasmodium_falciparum_TIGR|(protein codin			
	K.HDIVTS	3.7681	0.3304	100	2340.249	2340.249		7074.4	1	6.002	38.1	1
	K.LNAIVD	3.5983	0.3728	100	2108.139	2107.13		5822.7	1	6.071	55.9	6
	R.NNHADL	3.4003	0.344	100	1537.8	1536.814		5290.2	1	5.83	70.8	1
	R.EADSLI	3.8799	0.4862	100	2037.104	2037.102		8932.5	4	7.52	41.2	1
	K.LLHFSV	3.2875	0.3526	100	1744.896	1744.898		5708.5	2	5.763	50	6
	K.ILSDKHE	4.1914	0.3739	100	1402.822	1402.821		3896.4	1	6.312	72.7	3
PF14_0527		14	8.40%	610	69527	9.2	U		Annotation|Plasmodium_falciparum_TIGR|(protein codin			
	S.AGDSTF	3.6538	0.3157	100	1236.64	1235.638		5295.2	1	5.896	77.3	6
	K.TNDNLI	4.0955	0.4457	100	2541.43	2541.423		6557.8	2	6.708	36.4	5
	R.ILVPNNI	4.0252	0.4752	100	1848.077	1848.072		3609.2	1	8.171	63.3	3
PFD0850c		4	8.40%	296	34295	7.4	U		Annotation|Plasmodium_falciparum_Sanger|(protein coc			
	R.AYHSGS	2.933	0.3902	99.8	1313.594	1313.591		3854.5	1	7.067	55	2
	K.IVPIYVG	2.8035	0.3375	99.9	1487.85	1487.847		5404.9	1	5.447	61.5	2
PFL2245w		24	8.40%	261	30624	8.9	U		Annotation|Plasmodium_falciparum_Sanger|(protein coc			
	R.FVDFPG	2.7859	0.2836	99.9	1043.533	1043.532		3494.6	1	5.279	87.5	6
	K.APCHIE	3.4484	0.4379	100	1471.706	1471.704		7360.3	1	7.928	62.5	2
	K.APCHIE	3.8927	0.5073	100	1478.723	1478.721		8998.6	1	8.17	79.2	16
PFF1185w		101	8.30%	2719	315620	7	U		Annotation|Plasmodium_falciparum_Sanger|(protein coc			
	K.WYCPIC	3.8945	0.2593	100	1628.74	1625.732		6392.4	2	5.277	72.7	12
	K.GFVLD	6.6887	0.4845	100	2872.29	2869.282		7778.6	1	7.839	39.6	12
	K.LLGCDF	5.7399	0.5677	100	4219.744	4217.727		8554.5	1	9.059	28	2
	K.NEDPLL	3.3816	0.388	100	1420.728	1420.73		4057.5	1	6.059	81.8	12
	K.IDGPHL	3.9986	0.2806	100	2100.218	2100.216		6968.3	4	5.626	38.9	3
	K.GKFDLK	4.2399	0.2549	100	1868.007	1864.984		6598.9	1	5.467	66.7	4

	K.SFMHS\	4.1829	0.2585	100	1938.893	1937.889		4976.2	1	6.322	63.3	3	
	M.HSVPVI	4.2189	0.4087	100	1572.748	1572.748		7290.7	1	6.903	47.9	3	
	R.GVNFTF	4.2565	0.3878	100	1957.917	1956.91		4691.4	1	7.349	66.7	31	
	R.SQLHDS	4.1004	0.4237	100	1641.811	1641.808		9024.9	1	7.92	76.9	6	
	R.KRIDAL	3.4902	0.4127	100	2019.071	2019.062		6778.7	1	6.766	60	4	
	K.NDSCSS	3.2455	0.4313	100	1338.603	1338.597		5658.8	1	6.856	65	2	
	K.NSGYSI	4.4334	0.3901	100	2110.055	2110.039		7283.1	1	7.287	50	6	
	R.RNTSS\	5.7229	0.5035	100	1799.881	1799.878		6690.7	1	8.226	80	1	
PFE1345c		30	8.30%	962	109798	5.5	U		Annotation|Plasmodium_falciparum_Sanger|(protein coc				
	K.TVHLRP	3.7031	0.3789	100	1863.956	1863.947		8440.6	1	6.863	71.4	1	
	K.AQHISI	3.0499	0.3993	100	1244.659	1244.658		4996.3	1	6.409	83.3	9	
	R.TDGWKI	4.1849	0.3707	100	3057.439	3056.42		7996.7	1	6.09	26	2	
	R.LIVENV	7.1296	0.3868	100	3135.756	3132.741		8116.1	1	8.242	40.2	18	
MAL13P1.303		5	8.30%	408	47154	9.4	U		Annotation|Plasmodium_falciparum_Sanger|(protein coc				
	K.TFGNLE	3.237	0.3983	100	1237.644	1237.643		6408	1	6.667	77.3	3	
	K.GYGFV\	3.3764	0.3368	99.9	2356.172	2355.144		6566.3	1	6.186	38.1	2	
PF10_0033		1	8.30%	108	12369	6.5	U		Annotation|Plasmodium_falciparum_TIGR|(protein codin				
	K.RNVNFI	3.1295	0.3915	100	1200.602	1200.602		5629.3	1	6.064	81.2	1	
PF14_0201		25	8.20%	969	112574	4.6	U		Annotation|Plasmodium_falciparum_TIGR|(protein codin				
	K.CSQGNI	5.5086	0.3925	100	3015.435	3013.427		8138.1	1	7.482	35.4	5	
	K.SALLHS	3.0111	0.3802	99.8	1401.722	1401.72		5227.1	1	6.772	63.6	1	
	K.SALLHS	3.6019	0.3234	100	1408.739	1408.737		6251.9	3	6.227	63.6	14	
	K.DYDVVS	3.408	0.362	99.9	2273.139	2273.141		4954.8	1	6.268	32.9	2	
	K.QDDQLI	3.9373	0.4087	100	2533.11	2532.109		7185.4	1	7.409	42.9	3	
PF13_0338		49	8.20%	796	92728	6.9	U		Annotation|Plasmodium_falciparum_Sanger|(protein coc				
	R.SIILLC	3.3584	0.3069	100	1612.87	1612.871		7864.4	1	6.504	66.7	12	
	K.LFCGLP	2.7133	0.2956	99.9	1083.611	1083.61		4510.8	5	6.209	68.8	1	
	R.KIGCEL	4.1492	0.2325	99.9	1605.787	1605.791		5371.6	1	6.718	76.9	2	
	K.LYSNES	4.0604	0.4143	100	1423.697	1423.697		7861.4	1	8.601	81.8	18	
	K.SIIHFY	5.2969	0.4869	100	2145.054	2145.052		6034.4	1	8.368	65.6	16	
PF08_0036		103	8.20%	759	86169	6.3	U		Annotation|Plasmodium_falciparum_Sanger|(protein coc				
	K.NPFPLH	3.762	0.4022	100	1691.86	1688.851		6321.8	1	7.039	73.1	18	
	R.QAYLQF	4.2976	0.4985	100	1936.968	1936.967		5847.6	1	9.296	63.3	13	
	R.SHFLQT	7.0702	0.5279	100	2147.986	2146.983		8907	1	10.384	82.4	45	
	R.EQGFHI	4.0197	0.3075	100	1833.84	1832.836		5601.8	1	6.834	65.4	27	
PFD0295c		30	8.20%	731	85461	5.8	U		Annotation|Plasmodium_falciparum_Sanger|(protein coc				

	K.KKDDEE	6.6436	0.5418	100	2595.081	2595.078	7575.4		1	10.589	57.1	5	
	K.KDDEDE	6.3288	0.5578	100	2467.993	2466.983	11173.1		1	10.566	65	3	
	K.DDEDDI	5.8623	0.4222	100	2339.895	2338.888	6637.4		1	10.036	65.8	5	
	K.NNNIDD	5.9198	0.4048	100	2542.02	2541.014	7934.4		1	6.34	59.5	4	
	K.NNNIDD	4.5932	0.3661	100	2670.113	2669.109	6698.8		1	5.981	52.3	3	
	K.NNNIDD	4.167	0.3802	100	2677.131	2676.126	5868.8		1	5.94	33	1	
	K.DLTEQY	5.0725	0.287	100	1952.897	1951.893	6073.9		1	6.267	67.9	9	
PF10_0345		7	8.20%	354	40120	4.7	U		Annotation|Plasmodium_falciparum_TIGR|(protein codin				
	K.KDMEAC	3.5336	0.3028	100	1283.677	1283.674	7403.2		1	6.203	75	1	
	K.GNNQID	3.7312	0.3452	100	2003.034	2003.03	5860.9		1	6.381	38.2	2	
	K.GNNQID	4.7494	0.4994	100	2010.072	2010.047	8401.7		1	8.815	64.7	4	
PFB0595w		16	8.20%	328	37356	8.8	U		Annotation|Plasmodium_falciparum_TIGR|(protein codin				
	M.GKDYYS	3.6143	0.4384	100	1364.724	1364.729	8721.5		1	7.69	81.8	10	
	K.NIAEAY	4.329	0.3542	100	1586.771	1586.766	11122.9		1	8.378	84.6	3	
	K.NIAEAY	4.2529	0.4753	100	1735.854	1735.85	5179.4		1	9.055	71.4	3	
PF10_0119		3	8.20%	267	31791	7.1	U		Annotation|Plasmodium_falciparum_TIGR|(protein codin				
	K.INKENL	3.2861	0.2517	99.8	1680.859	1680.867	5738		1	5.74	76.9	1	
	K.IACSHV	2.3606	0.3349	99.8	945.4647	945.465	5256.5		1	6.427	85.7	2	
MAL13P1.61		4	8.20%	245	28255	9.9	U		Annotation|Plasmodium_falciparum_Sanger|(protein coc				
	K.VHNDLN	2.8648	0.255	99.8	1023.559	1023.559	3836.5		2	5.068	87.5	3	
	R.KNEITN	3.1967	0.2948	99.9	1397.688	1397.689	5618.9		1	5.408	65	1	
PF13_0170		7	8.10%	918	108532	8.2	U		Annotation|Plasmodium_falciparum_Sanger|(protein coc				
	K.EDNTPS	3.0163	0.303	99.8	2579.204	2579.194	4767.1		2	5.094	38.1	2	
	R.GNLKDF	3.2117	0.3029	99.9	1417.703	1417.708	4952.1		1	5.385	70.8	3	
	R.RGYSPD	3.571	0.4487	100	1677.824	1677.813	5283		1	6.637	65.4	1	
	K.VIITNY	3.8159	0.3337	99.9	2895.459	2895.469	6974.7		1	5.666	25	1	
PFA0530c		5	8.10%	186	22173	5.1	U		Annotation|Plasmodium_falciparum_Sanger|(protein coc				
	R.KVPNII	3.3466	0.3957	100	1479.899	1479.89	7789.6		1	6.639	71.4	5	
PF14_0445		36	8.10%	172	20343	4.5	U		Annotation|Plasmodium_falciparum_TIGR|(protein codin				
	K.TLYNLC	5.1652	0.3537	100	1824.905	1821.892	8979.7		1	6.498	80.8	36	
PF07_0079		6	8.10%	173	20228	10.1	U		Annotation|Plasmodium_falciparum_Sanger|(protein coc				
	R.VLEQLT	3.6853	0.3196	100	1629.926	1629.922	9054.7		2	6.622	65.4	6	
PF11_0286		9	8.00%	424	51186	6.9	U		Annotation|Plasmodium_falciparum_TIGR|(protein codin				
	T.KVNFTY	3.2854	0.2931	99.9	1642.846	1641.839	5331.3		4	5.543	54.2	6	
	K.SWIYYI	2.8905	0.3408	99.8	2531.32	2529.31	4987.6		96	5.851	30	3	
MAL13P1.231		34	8.00%	461	50912	8.3	U		Annotation|Plasmodium_falciparum_Sanger|(protein coc				

	R.GTLMEL	5.1485	0.4225	100	2533.349	2533.344		8557.6	1	7.576	35.4	4
	R.GTLMEL	4.8111	0.4152	100	2534.334	2533.344		6254.1	1	8.06	43.8	29
	K.IIDVDQ	2.9361	0.2759	99.8	1430.749	1430.749		9329	1	6.263	72.7	1
PFI0580c		12	8.00%	413	46958	6.7	U		Annotation|Plasmodium_falciparum_Sanger|(protein coc			
	K.KPLIDP	3.1207	0.2603	99.8	1356.754	1356.753		5821.3	1	5.785	68.2	5
	K.RVTQSY	4.6514	0.4933	100	2311.287	2311.285		6153.8	1	9.096	52.5	6
	R.VTQSYV	5.1622	0.5534	100	2155.189	2155.184		6892.2	1	9.12	68.4	1
PFL1285c		15	8.00%	264	30163	5.1	U		Annotation|Plasmodium_falciparum_Sanger|(protein coc			
	R.ASSLSD	4.5474	0.4569	100	2235.302	2234.302		7220.5	1	7.136	45	15
PFC0785c		36	8.00%	225	26466	6.9	U		Annotation|Plasmodium_falciparum_Sanger|(protein coc			
	K.KNVFAI	6.5982	0.4344	100	2061.116	2061.128		9625.7	1	8.673	51.5	7
	K.KNVFAI	4.9504	0.5177	100	2061.122	2061.128		9434.9	1	8.37	61.8	5
	K.NVFAII	5.6767	0.4941	100	1911.969	1911.982		9366.1	1	9.679	75	24
PFC0395w		28	7.90%	610	69864	7.2	U		Annotation|Plasmodium_falciparum_Sanger|(protein coc			
	R.YYNPNV	4.0281	0.3203	100	2132.015	2131.011		8424.7	1	7.05	62.5	14
	K.IHDLHY	3.4884	0.3932	100	1443.74	1443.739		8225.2	1	7.578	80	8
	R.VPFLDI	4.7832	0.4123	100	2360.262	2359.256		6247.8	1	8.071	65.8	6
PFE0785c		25	7.90%	456	51800	7.9	U		Annotation|Plasmodium_falciparum_Sanger|(protein coc			
	K.IMHLVD	2.608	0.3486	100	919.5156	919.5148		4471.7	1	5.98	85.7	1
	K.GTSHM	4.8245	0.4114	100	2934.307	2933.291		8848.2	1	6.943	36.1	24
PF13_0268		5	7.90%	203	23415	10.9	U		Annotation|Plasmodium_falciparum_Sanger|(protein coc			
	R.INPFMS	2.9529	0.4126	100	1913.036	1912.031		4889.7	1	6.185	53.3	1
	N.PFMSSF	3.663	0.3016	100	1657.844	1656.835		5830.5	1	6.557	80.8	2
	F.MSSPCH	3.6706	0.4698	100	1433.766	1433.766		6322.4	1	7.852	68.2	2
PFL0625c		106	7.80%	1377	166061	6.8	U		Annotation|Plasmodium_falciparum_Sanger|(protein coc			
	R.IICQHG	3.2225	0.4483	100	1431.804	1431.804		6321.1	1	6.932	58.3	3
	K.SNIMME	3.681	0.4485	100	1919.013	1919.006		6834.6	1	6.274	62.5	1
	K.TTDGNV	4.4634	0.3068	100	2057.96	2054.949		6965.2	1	5.963	55.3	37
	K.NINYNT	4.4297	0.2584	100	3209.569	3208.554		8784	1	5.422	34.6	3
	K.NINYNT	7.0172	0.4399	100	3238.637	3236.622		7075.6	1	7.759	26.9	27
	T.ATLEDI	3.8876	0.2899	100	2490.241	2489.23		11183.4	1	5.819	50	3
	R.HDDHHI	3.406	0.3823	100	1229.517	1226.505		5360.8	1	6.575	88.9	26
	K.KRENDI	3.7301	0.1532	99.9	1148.652	1145.639		5032.9	3	4.417	81.2	2
	K.NAWNLI	3.1566	0.2578	99.8	1434.651	1434.65		5686.9	1	6.323	72.7	4
PFD0405c		4	7.80%	344	42215	9.2	U		Annotation|Plasmodium_falciparum_Sanger|(protein coc			
	K.GGGHYI	4.5867	0.442	100	1631.781	1631.775		6362.6	1	8.279	76.9	1

	R.SNKYDD	3.0996	0.2858	99.9	1643.791	1642.783		7180.2	1	5.812	62.5	3
PFL1270w		11	7.80%	295	33268	7.2	U		Annotation|Plasmodium_falciparum_Sanger|(protein coc			
	K.VGILSA	3.2113	0.3397	99.9	1376.745	1376.743		7480.2	1	6.654	62.5	2
	K.NLVNQT	3.2611	0.2441	99.8	1241.676	1241.676		6424.2	2	5.518	77.8	9
PFL0590c		76	7.70%	1208	133874	6.5	U		Annotation|Plasmodium_falciparum_Sanger|(protein coc			
	K.TNESLL	3.5019	0.3892	100	1670.857	1670.856		6135.7	1	6.2	60.7	1
	K.WGIHGN	3.8346	0.407	100	1836.935	1836.943		5272.8	1	7.234	55.9	4
	K.VGYSFI	3.1642	0.3423	100	1389.691	1389.692		7879.4	1	6.662	72.7	9
	G.YVASFD	3.3959	0.3089	99.9	1432.759	1432.759		3768.1	4	5.894	66.7	3
	Y.VASFDP	2.6055	0.337	99.8	1269.687	1269.696		3860.2	2	5.409	63.6	2
	K.VIMITG	4.708	0.4798	100	1741.993	1741.989		9079	1	9.11	81.2	29
	K.GKVENI	6.1301	0.4702	100	2030.096	2030.104		8000.2	1	8.847	57.4	15
	K.GKVENI	5.5615	0.4309	100	2044.145	2044.138		6362.8	1	9.406	73.5	11
	K.VENINP	4.0805	0.3519	100	1859.021	1859.021		5865.9	1	6.678	70	2
PFB0585w		39	7.70%	365	42940	7.6	U		Annotation|Plasmodium_falciparum_TIGR|(protein codin			
	K.HGQNW	4.6339	0.4177	100	1922.928	1921.924		6540.4	1	6.961	71.4	36
	K.NVELHS	3.1933	0.3401	100	1558.81	1557.807		6928.6	1	6.208	70.8	3
PFD0311w		5	7.70%	272	31629	7.5	U		Annotation|Plasmodium_falciparum_Sanger|(protein coc			
	K.NKEHSV	5.1832	0.3884	100	2500.238	2500.247		4511.4	1	8.397	65	3
	K.NKEHSV	3.8604	0.3239	99.9	2500.238	2500.247		6696.4	5	5.185	27.5	2
MAL13P1.16		4	7.70%	208	24947	6.2	U		Annotation|Plasmodium_falciparum_Sanger|(protein coc			
	K.NQISNIQ	4.1125	0.2136	99.9	1884.043	1883.024		8755.2	1	5.161	70	4
PF13_0322		244	7.60%	1193	138862	7	U		Annotation|Plasmodium_falciparum_Sanger|(protein coc			
	K.NNPTEL	6.0731	0.4271	100	2475.221	2471.21		11944.1	1	7.817	62.5	177
	R.GLNDSL	5.2297	0.5505	100	1781.005	1781.004		8221.5	1	10.102	73.3	48
	K.LNYNRD	4.0467	0.2982	100	1919.026	1918.019		8139.6	1	5.561	64.3	14
	K.HYAHNI	3.5952	0.3581	100	1913.985	1911.958		4497.4	1	6.002	57.1	3
	K.HLFVNS	2.7341	0.3334	99.9	1287.671	1287.67		5250.9	1	5.567	75	1
	R.YIINTIG	3.5822	0.367	100	1531.925	1531.922		6373.4	1	6.529	83.3	1
PFE0280c		26	7.60%	924	112700	6.4	U		Annotation|Plasmodium_falciparum_Sanger|(protein coc			
	K.DKDNDI	5.9878	0.2375	100	1659.787	1659.783		7058.2	1	5.317	88.5	2
	R.NQNVIH	3.4641	0.2456	99.9	1233.612	1233.612		5653.8	1	5.463	77.8	1
	K.EQHTNC	2.9075	0.4426	100	1367.67	1367.667		4476	1	6.795	70	2
	K.TIKEDL	5.3709	0.3419	100	1819.945	1819.941		7992.9	1	8.515	78.6	7
	K.LNDSSE	4.9126	0.4037	100	2300.195	2300.193		7099.9	1	7.631	50	14
PF07_0023		22	7.60%	821	94237	6.4	U		Annotation|Plasmodium_falciparum_Sanger|(protein coc			

	K.IYNHDT	3.7852	0.3118	100	2032.071	2031.055	9487.9	1	6.26	62.5	2
	K.AGHCSN	2.43	0.4205	99.9	1100.475	1100.473	4344.5	1	7.425	83.3	3
	K.LAEHVL	3.0193	0.3209	100	1156.711	1156.717	7369.5	1	6.283	77.8	3
	R.RKDVSN	3.8216	0.2359	99.9	1560.863	1559.858	10657.3	1	5.422	79.2	4
	K.DVSNEII	4.1673	0.3573	100	1296.716	1296.713	7804.6	1	7.03	75	3
	R.WNGWII	3.843	0.2451	99.9	1543.782	1541.775	4425.1	1	5.434	81.8	7
PF13_0350		17	7.60%	576	65789	7.7	U	Annotation|Plasmodium_falciparum_Sanger|(protein coc			
	K.NKDVFD	5.9402	0.4199	100	3497.732	3496.721	5585.4	1	6.975	32.1	3
	K.NKDVFD	4.8374	0.3417	100	3497.733	3496.721	11006.8	1	6.125	29.6	4
	L.PIDFEE	5.3996	0.3459	100	2368.173	2366.167	7949	1	6.677	66.7	7
	K.FNQALT	4.6125	0.5208	100	1840.857	1840.861	7455	1	8.695	73.3	3
		20	7.60%	458	49644	4.9	U	no description			
	L.RQSVEA	4.6248	0.3635	100	1359.724	1357.719	4921.9	1	6.628	86.4	2
	R.YALQLQ	4.1742	0.289	99.9	2601.429	2601.425	10943	1	6.128	28.4	2
	R.YALQLQ	5.7825	0.4402	100	2602.452	2601.425	8688	1	8.137	61.4	16
MAL8P1.108		2	7.60%	303	34520	7.4	U	Annotation|Plasmodium_falciparum_Sanger|(protein coc			
	R.YLNNSN	3.7743	0.2429	99.9	2724.429	2724.436	6602.2	1	5.739	36.4	2
PFA0110w		45	7.50%	1085	126521	4.5	U	Annotation|Plasmodium_falciparum_Sanger|(protein coc			
	R.ASITNS	3.7143	0.3709	100	1673.86	1673.857	5261.1	7	6.23	50	1
	K.QVNFMN	3.6675	0.3333	100	2138.101	2137.112	6936.5	1	6.166	52.9	9
	K.FKDFTG	4.9578	0.4397	100	1742.983	1742.992	8215.8	1	7.359	75	26
	K.IVSYIV	6.0646	0.4383	100	3659.851	3654.828	8261.5	1	8.552	35.2	9
PF13_0257		70	7.50%	863	101417	8.7	U	Annotation|Plasmodium_falciparum_Sanger|(protein coc			
	K.SIIEDLE	3.7378	0.2407	99.9	1343.746	1343.742	6385.7	1	6.755	81.8	4
	R.IEQYNW	3.937	0.3472	100	1810.959	1810.968	9352.1	1	6.588	69.2	1
	K.NSSILLI	5.4736	0.4565	100	2035.195	2035.195	8794	1	8.776	64.7	63
	K.YLIELED	5.1197	0.4769	100	2491.313	2490.323	7325.9	1	8.69	60	2
PF14_0541		54	7.50%	717	76417	6.5	U	Annotation|Plasmodium_falciparum_TIGR|(protein codin			
	T.KAADVG	3.5493	0.2493	99.9	1131.603	1131.601	6492.6	1	6.677	86.4	3
	K.SVAIAA	4.9734	0.3467	100	2618.372	2615.376	6586.1	1	7.011	45.7	46
	V.PGLISV	3.7676	0.465	100	1874.073	1873.07	6931.8	1	8.414	50	4
	V.PGLISV	4.2528	0.5785	100	1894.121	1894.121	6192.3	1	10.156	58.8	1
PF14_0223		7	7.50%	440	51835	7	U	Annotation|Plasmodium_falciparum_TIGR|(protein codin			
	R.NFIQHC	4.3103	0.3205	100	1727.809	1727.796	2514.4	1	6.208	79.2	2
	K.IIENPF	4.8602	0.4227	100	2450.306	2449.282	9042	1	7.829	47.4	5
PFE0160c		2	7.50%	308	37699	9.8	U	Annotation|Plasmodium_falciparum_Sanger|(protein coc			

	R.KEEEDE	4.1676	0.3987	100	2872.212	2871.207		4464.6	1	6.164	36.4	2
PF14_0309		3	7.50%	240	27439	7.9	U		Annotation|Plasmodium_falciparum_TIGR|(protein codin			
	K.LIIPIEE	4.0065	0.4684	100	2208.232	2208.243		6162	1	7.512	47.1	3
PFL2090c		1	7.50%	226	26512	5.8	U		Annotation|Plasmodium_falciparum_Sanger|(protein coc			
	K.VNNIPA	3.3614	0.3466	100	1882.056	1881.049		5056.1	1	6.252	53.1	1
PF14_0493		13	7.40%	895	102270	6.7	U		Annotation|Plasmodium_falciparum_TIGR|(protein codin			
	K.NHYISE	3.4027	0.4806	100	1561.702	1561.7		7003.7	1	8.347	75	1
	R.NDVGVL	3.9973	0.4602	100	2274.141	2273.139		4584.1	1	7.729	50	6
	K.KPCECT	4.178	0.4697	100	2279.912	2278.906		6511.1	1	8.384	55.9	3
	K.NLFYNY	3.41	0.3304	99.9	1871.872	1870.865		9245.6	1	5.751	57.1	3
PF14_0311		5	7.40%	269	31248	5.2	U		Annotation|Plasmodium_falciparum_TIGR|(protein codin			
	K.NIQLLI	4.4992	0.349	100	2327.247	2327.221		6044.1	1	6.806	52.6	2
	K.NIQLLI	5.2458	0.2979	100	2348.288	2348.273		6296	1	6.441	39.5	3
MAL7P1.58		1	7.40%	231	27329	9.6	U		Annotation|Plasmodium_falciparum_Sanger|(protein coc			
	M.AMAAIL	1.9945	0.5156	99.9	1803.26	1802.229		7954	1	6.936	31.2	1
PFE1005w		11	7.40%	189	22123	10.4	U		Annotation|Plasmodium_falciparum_Sanger|(protein coc			
	K.HIDFAT	3.7325	0.3693	100	1476.724	1476.724		6989.2	1	7.147	73.1	11
PF14_0205		16	7.40%	135	15741	9.9	U		Annotation|Plasmodium_falciparum_TIGR|(protein codin			
	R.AVINHL	3.0495	0.3002	99.9	980.5517	980.5529		5834.7	1	6.565	93.8	2
	R.AVINHL	3.4284	0.2759	100	1115.666	1115.665		4456.2	1	5.576	88.9	14
PF10_0135		5	7.30%	491	59187	6.1	U		Annotation|Plasmodium_falciparum_TIGR|(protein codin			
	K.NDHNTI	3.4511	0.3113	100	1277.665	1276.661		5021.1	1	5.55	80	1
	K.NDHDHE	2.5201	0.3764	99.9	1677.679	1677.675		3832.7	1	6.196	54.2	3
	K.HTPINN	3.1044	0.2858	99.9	1484.769	1484.768		5252.8	1	6.12	81.8	1
MAL7P1.84		15	7.30%	438	51111	7.2	U		Annotation|Plasmodium_falciparum_Sanger|(protein coc			
	K.NESVDH	3.3569	0.3572	100	1812.82	1811.816		3525.5	1	6.237	56.7	9
	K.NESEDH	2.7942	0.3586	99.9	1851.817	1851.811		4309.4	4	5.999	43.3	6
PF10_0323		7	7.30%	355	38926	9.4	U		Annotation|Plasmodium_falciparum_TIGR|(protein codin			
	L.KAIEQD	2.9632	0.3379	100	1207.677	1207.676		5413.5	1	5.799	72.2	1
	K.NVDSEV	4.7588	0.441	100	1889.902	1889.898		8778.5	1	7.905	66.7	6
PF14_0526		3	7.30%	177	20387	5.4	U		Annotation|Plasmodium_falciparum_TIGR|(protein codin			
	K.KMQELY	3.8195	0.219	99.9	1620.844	1620.842		6909.6	1	4.93	83.3	3
PF11_0053		33	7.20%	1426	167440	6.6	U		Annotation|Plasmodium_falciparum_TIGR|(protein codin			
	K.KNDVG	6.1176	0.5338	100	3022.34	3021.34		9934.1	1	8.375	33	3
	K.KNDVG	4.0058	0.45	100	3023.346	3021.34		6110	1	8.053	40	3
	K.KLNVSS	3.095	0.2736	99.9	1219.672	1218.669		7440.5	1	4.501	70	2

	R.KLNSSV	4.1369	0.3906	100	1785.804	1785.8		4567.4	1	7.464	66.7	11
	R.KLNSS	4.8726	0.4009	100	2293.075	2292.072		7120.3	1	7.187	36.8	6
	K.LNSSV	3.3926	0.3565	100	2163.982	2163.977		5834.7	4	6.94	33.3	3
	R.NIGNDT	3.8646	0.4222	100	1768.87	1768.869		4716.3	1	7.4	75	1
	R.LLITGT	3.5362	0.333	100	1440.864	1440.865		4904.2	1	6.829	70.8	2
	K.NIHILT	3.7064	0.3285	100	2330.202	2330.184		5725.5	1	5.347	52.9	2
PF11_0175		75	7.20%	906	102874	9.1	U		Annotation|Plasmodium_falciparum_TIGR|(protein codin			
	F.KENNVN	3.1998	0.2481	99.9	1214.651	1214.649		7030.6	1	5.679	85	1
	K.VDVEHL	5.7812	0.5297	100	3517.77	3517.759		9318.2	1	10.421	38.3	41
	K.VDVEHL	6.3048	0.3538	100	3535.775	3531.793		10216.6	1	8.547	27.5	13
	S.GLSNDE	4.3041	0.4111	100	2626.317	2625.294		6133.1	1	7.29	43.2	14
	R.EKPHSV	3.1642	0.3279	99.9	1669.878	1669.88		7278.3	1	5.9	61.5	3
	K.VFKPEF	2.6644	0.2853	99.8	1135.619	1135.626		3198.8	3	6.067	93.8	3
PFB0685c		28	7.20%	885	102147	5.3	U		Annotation|Plasmodium_falciparum_TIGR|(protein codin			
	K.LIYEYA	3.5342	0.3601	100	1304.666	1304.664		6360.5	1	6.477	80	2
	R.NVHQTV	4.9985	0.3673	100	1755.887	1754.883		7165	1	7.658	80	9
	K.HLLEDN	6.195	0.5211	100	4269.066	4267.046		10171.7	1	8.915	27.1	17
PF14_0129		2	7.20%	471	55050	10	U		Annotation|Plasmodium_falciparum_TIGR|(protein codin			
	N.TSNTNN	4.1478	0.3152	99.9	3697.679	3695.728		5353.3	10	5.062	18.2	2
PFE0640w		4	7.20%	265	31952	9.2	U		Annotation|Plasmodium_falciparum_Sanger|(protein coc			
	K.KPQILP	4.1798	0.2925	100	2336.256	2336.266		6122.4	1	6.483	55.6	4
PF14_0649		55	7.10%	2558	295829	5.3	U		Annotation|Plasmodium_falciparum_TIGR|(protein codin			
	K.ISDWLE	4.7745	0.3809	100	1645.866	1645.87		8554.2	1	7.232	83.3	19
	N.ASNQQI	5.1755	0.3254	100	4465.25	4464.216		7474.4	1	6.792	17.6	1
	R.PTLFNL	4.1194	0.3285	99.9	2414.285	2413.295		11210.1	22	5.327	30	1
	K.NSVFGD	3.0902	0.3269	99.8	2089.052	2089.072		6600.8	1	5.799	50	3
	K.NVLLDD	4.482	0.5093	100	1720.963	1720.96		8206.4	1	9.033	80	9
	K.VQHDES	2.8815	0.3189	99.9	1521.686	1520.68		5509.5	2	5.449	62.5	2
	K.NLQEES	5.1577	0.6013	100	1895.884	1895.883		6957.6	1	11.217	78.1	6
	K.KKQEDN	3.7498	0.349	100	1880.949	1880.947		8050	2	6.468	41.1	2
	K.KKQEDN	5.516	0.5402	100	1887.969	1887.964		8091.8	1	9.253	78.6	3
	K.YTLYNF	2.082	0.4224	99.9	1089.574	1089.573		4606.6	2	6.356	78.6	3
	K.NAQSP	4.8184	0.5184	100	2185.956	2184.951		8840.4	1	8.539	63.2	3
	K.NNSDDV	4.0533	0.3897	100	2496.026	2495.018		8072.6	1	6.653	42.9	3
PF11_0091		29	7.10%	1828	206874	6	U		Annotation|Plasmodium_falciparum_TIGR|(protein codin			
	K.SGLVSN	4.6171	0.4273	100	1662.802	1662.801		6953.5	1	8.307	78.6	3

	Y.KDDIIG	4.0226	0.2201	99.9	1466.752	1466.749		6562.9	1	5.967	70.8	2
	K.NHQSD	6.6473	0.2744	100	3584.479	3583.47		8890.3	1	4.819	30.8	3
	K.NNSSSY	4.1692	0.3915	100	1392.702	1390.693		6270.1	1	7.043	72.7	4
	K.NGSIDP	3.8328	0.4108	100	2084.004	2083.995		5664.5	1	6.888	50	1
	K.NNNDSF	3.3308	0.2498	99.9	1322.637	1322.634		4346.7	2	5.279	70	3
	R.TFESHY	7.1547	0.4721	100	3501.639	3500.633		10431	1	8.646	33	13
MAL8P1.73		27	7.10%	1213	140528	9.7	U		Annotation|Plasmodium_falciparum_Sanger|(protein coc			
	K.THVVGC	3.553	0.3678	100	1943.002	1942		6731.3	1	6.659	52.8	3
	R.NYNDLV	4.0202	0.3647	100	1975.967	1973.961		6504	1	6.924	63.3	18
	K.IVLGCI	2.3908	0.4484	100	1359.685	1359.684		4791.6	2	6.779	59.1	2
	R.EAQNVI	3.8268	0.3391	100	1911.99	1911.989		6751.4	1	6.402	65.6	3
	K.HLPSDV	3.1409	0.3235	99.9	2374.247	2373.264		4634.1	3	5.243	33.3	1
PFF1410c		13	7.10%	665	77126	7.6	U		Annotation|Plasmodium_falciparum_Sanger|(protein coc			
	K.IIYLYDV	4.4807	0.4296	100	2303.247	2303.257		8481.2	1	7.748	47.2	9
	K.NVEHNN	3.0799	0.2952	99.9	1310.637	1310.634		4608.8	1	5.475	70	3
	K.LISNLLD	3.4512	0.3683	100	1976.111	1976.112		6765.1	1	6.313	46.9	1
PF13_0102		45	7.10%	651	75872	6.7	U		Annotation|Plasmodium_falciparum_Sanger|(protein coc			
	R.RHDLLS	3.1634	0.2592	99.9	1353.752	1353.749		6279.2	1	5.047	80	5
	K.EVLDFV	3.2257	0.3216	99.9	1765.862	1765.862		4526.3	2	5.789	50	9
	R.SNLKEN	5.3481	0.4358	100	2375.188	2374.183		5987.8	1	7	41.2	9
	R.SNLKEN	5.1651	0.4515	100	2382.205	2381.2		8272	1	8.478	55	12
	K.ENEAAC	4.134	0.3919	100	1932.93	1931.929		5640.2	1	6.748	59.4	10
PFE1600w		12	7.10%	509	60188	5.1	U		Annotation|Plasmodium_falciparum_Sanger|(protein coc			
	K.NGKHEN	3.1917	0.3698	100	1430.657	1430.652		5261.3	1	5.827	72.7	5
	K.NGKHEN	3.3952	0.3321	99.8	1437.671	1437.669		4136.1	1	7.675	68.2	1
	K.NKVPHE	2.8644	0.286	99.9	1142.597	1142.596		3679.1	42	5.161	66.7	3
	K.VYMGKF	3.2171	0.2912	99.9	1717.874	1717.862		5820	2	5.764	57.7	3
PF11_0413		44	7.10%	449	52813	4.5	U		Annotation|Plasmodium_falciparum_TIGR|(protein codin			
	R.KDVADF	5.2029	0.4927	100	2134.012	2133.998		7299	1	8.778	73.5	24
	K.DVADFL	4.4867	0.4321	100	2005.895	2005.903		7651.8	1	6.844	65.6	18
	K.NKLPTP	3.4556	0.2328	99.8	1710.917	1709.911		4002.4	1	5.388	69.2	2
PFA0580c		34	7.10%	434	51370	8.5	U		Annotation|Plasmodium_falciparum_Sanger|(protein coc			
	K.KHENDL	3.5361	0.3155	100	1480.77	1479.767		4166.1	1	5.514	81.8	15
	K.KIPLNL	4.6296	0.4347	100	2180.232	2180.215		10406.6	1	7.678	58.3	19
PFL0585w		25	7.10%	381	42829	7.6	U		Annotation|Plasmodium_falciparum_Sanger|(protein coc			
PF13_0346		5	21.10%	128	14617	9.9	U		Annotation|Plasmodium_falciparum_Sanger|(protein coc			

	-.MQIFVK	4.7744	0.3628	100	3020.607	3020.623		10327.9	1	5.987	30.8	10	
	K.TITLDV	4.2997	0.4174	100	1787.947	1787.947		6508.8	1	7.067	63.3	15	
PF11_0409		3	7.10%	198	23233	6	U		Annotation|Plasmodium_falciparum_TIGR|(protein codin				
	K.NYNDAF	3.1085	0.3323	99.9	1720.799	1719.805		7709.8	1	6.293	53.8	3	
PF11_0331		12	7.00%	544	60261	7.1	U		Annotation|Plasmodium_falciparum_TIGR|(protein codin				
	K.VGDWD	3.2506	0.2927	99.9	1257.602	1257.598		8514.5	1	6.486	83.3	3	
	R.SIHDAL	3.4258	0.4992	100	1244.607	1244.606		3877.3	1	7.485	75	7	
	R.EQLAIAI	2.9601	0.3125	99.8	1871.073	1871.053		9675.7	9	5.46	43.8	2	
PFI1365w		3	7.00%	273	32364	6.7	U		Annotation|Plasmodium_falciparum_Sanger|(protein coc				
	R.VVCCTC	3.621	0.323	100	2180.011	2179.006		5341.6	1	6.876	47.2	3	
PFB0310c		12	7.00%	272	30548	4.7	U		Annotation|Plasmodium_falciparum_TIGR|(protein codin				
	K.HNNGG	3.9808	0.3098	100	2065.868	2064.862		7984.9	7	6.223	36.8	3	
	K.HNNGG	3.9688	0.3309	100	2221.966	2220.963		5582.1	2	6.169	38.9	3	
	K.HNNGG	5.0773	0.4746	100	2221.968	2220.963		5516.1	1	7.287	41.7	6	
PF14_0593		139	6.90%	1357	161218	6.4	U		Annotation|Plasmodium_falciparum_TIGR|(protein codin				
	K.SFLLNL	4.6354	0.4318	100	1993.136	1993.131		4742.3	1	7.166	65.6	22	
	K.SANQNL	4.1249	0.4778	100	1771.878	1771.877		7713.9	1	8.544	71.4	33	
	K.EHNIKD	3.5151	0.3692	100	1664.768	1664.763		4446.8	1	6.154	57.7	3	
	K.LPPLLNI	2.8292	0.2832	99.8	1164.738	1164.736		4117.7	1	5.956	83.3	2	
	K.DLTTFF	4.9179	0.3749	100	1778.916	1776.906		8879.1	1	7.119	78.6	36	
	K.NFLENF	4.712	0.2438	99.9	2796.475	2795.466		9358.3	1	5.115	36.4	2	
	K.NFLENF	5.0955	0.3422	100	2805.497	2802.483		6320.3	1	6.757	47.7	41	
PFF0590c		21	6.90%	656	78129	8.2	U		Annotation|Plasmodium_falciparum_Sanger|(protein coc				
	K.FIDPLS	4.0035	0.4924	100	3630.828	3629.825		6565.2	1	8.283	35	19	
	K.IFIDYF	3.9566	0.4089	100	1776.949	1775.945		8118	1	7.177	73.1	2	
PFA0255c		5	6.90%	568	67157	8.2	U		Annotation|Plasmodium_falciparum_Sanger|(protein coc				
	K.ETISNI	3.7055	0.349	100	2510.295	2509.286		7916.2	1	6.177	33.3	2	
	K.TFHEVQ	3.6217	0.3883	100	1958.015	1958.014		5906.6	1	7.05	65.6	3	
PF11_0292		3	6.90%	248	29141	5.4	U		Annotation|Plasmodium_falciparum_TIGR|(protein codin				
	K.ENTDIN	4.0172	0.3658	100	2092.945	2091.932		5174.5	1	6.302	50	3	
PF11_0139		33	6.90%	218	25102	8.2	U		Annotation|Plasmodium_falciparum_TIGR|(protein codin				
	K.NNCAVA	4.7297	0.4222	100	1600.779	1597.769		7613.6	1	7.6	78.6	33	
PF08_0121		9	6.90%	217	24892	6.8	U		Annotation|Plasmodium_falciparum_Sanger|(protein coc				
	K.KIENVSY	4.8637	0.4763	100	1796.061	1793.04		8337	1	8.398	67.9	9	
MAL13P1.290		1	6.90%	160	18955	8.2	U		Annotation|Plasmodium_falciparum_Sanger|(protein coc				
	R.KKYEEQ	3.3379	0.3296	100	1384.785	1384.791		8127.8	1	6.605	80	1	

PFC0735w		1	6.90%	130	14874	10	U		Annotation|Plasmodium_falciparum_Sanger|(protein coc				
	R.QVLIRP	2.4969	0.333	99.9	1034.643	1034.644		2306	1	5.97	75	1
PFI1085w		45	6.80%	1542	182087	6	U		Annotation|Plasmodium_falciparum_Sanger|(protein coc				
	R.GSSNST	3.0774	0.3245	99.9	1440.675	1440.672		6019.9	1	6.317	50	3
	R.NVKEEE	3.6698	0.2611	100	1495.762	1495.761		6358.9	1	5.355	81.8	1
	K.IYDEEQ	4.9407	0.4039	100	2182.999	2181.992		6117.2	1	7.092	65.6	6
	K.KLENYD	5.4312	0.2811	100	1651.817	1651.818		7093.5	1	6.352	87.5	9
	K.KLENYD	5.2277	0.2075	100	1781.92	1779.913		6586.4	1	5.289	76.9	6
	R.FRNEYD	3.5753	0.3198	99.9	2363.065	2362.06		6453.4	1	5.979	55.9	3
	K.KNNLHN	4.4588	0.2385	100	1385.682	1382.678		5626	1	5.692	86.4	14
	K.LNENTG	5.1703	0.3246	100	2047.906	2047.903		5762.3	1	6.999	61.8	3
PFD0595w		24	6.80%	778	92196	5.3	U		Annotation|Plasmodium_falciparum_Sanger|(protein coc				
	R.NVSSFN	4.1288	0.5099	100	1510.692	1510.693		6578.5	1	8.843	75	6
	K.GQILKD	3.8171	0.4376	100	1566.803	1566.799		6230	1	7.647	79.2	5
	K.DNKHDH	5.154	0.2858	100	1847.922	1847.919		3818.4	1	5.996	71.4	2
	K.HDHIQD	4.2846	0.3316	100	1491.76	1490.754		5873.7	1	6.688	81.8	6
	K.DNKHDH	3.2596	0.3798	100	1433.692	1433.689		5812.4	1	6.848	77.3	4
	K.HDHNQA	2.5514	0.2993	99.8	1083.541	1083.541		3724.7	1	5.809	68.8	1
PF14_0713		11	6.80%	701	83749	5.5	U		Annotation|Plasmodium_falciparum_TIGR|(protein codin				
	K.IQIQEDI	3.3153	0.344	100	2826.652	2826.643		5020.8	1	5.477	30.4	1
	K.NLPCSI	4.3562	0.3408	100	2848.352	2846.32		5054.1	1	8.358	47.8	10
PFA0195w		16	6.80%	575	67802	9.1	U		Annotation|Plasmodium_falciparum_Sanger|(protein coc				
	K.APFNVE	4.2181	0.3314	100	2585.208	2584.196		6793.1	1	6.436	52.3	3
	K.NIPIDMI	4.1187	0.3132	100	1919.078	1915.1		5469.5	1	6.236	63.3	13
PF13_0147		22	6.80%	527	62883	9.5	U		Annotation|Plasmodium_falciparum_Sanger|(protein coc				
	K.TNQTNF	3.8542	0.3859	100	2054.009	2053.995		6130.4	1	6.304	61.8	3
	K.MDKCDC	5.375	0.5003	100	2268.101	2267.095		8183.1	1	7.934	45.6	2
	K.MDKCDC	5.8189	0.5147	100	2282.137	2281.13		7425.4	1	8.262	64.7	3
	K.CDQHTF	5.3306	0.3873	100	1893.941	1892.933		7032.3	1	6.814	42.9	7
	K.CDQHTF	5.0494	0.4681	100	1907.97	1906.967		7240.8	1	8.258	71.4	7
PFE1405c		107	6.80%	517	61419	7.3	U		Annotation|Plasmodium_falciparum_Sanger|(protein coc				
	R.GVINNY	5.3726	0.4493	100	2977.39	2976.383		8071.2	1	7.262	43.8	89
	R.GVINNY	5.3255	0.499	100	2978.392	2976.383		12629.9	1	7.826	34.4	11
	K.LKPYIE	3.3086	0.3698	100	1353.7	1351.691		3887.7	1	7.046	83.3	7
PFL1295w		12	6.80%	381	44980	5.9	U		Annotation|Plasmodium_falciparum_Sanger|(protein coc				
	R.MCGLHC	4.2776	0.3803	100	2925.4	2925.395		6850.5	1	7.879	48	2

	R.MCGLH(	7.4867	0.481	100	2947.456	2946.446		9477	1	8.42	36	10
MAL13P1.228		4	6.80%	370	43220	8	U		Annotation|Plasmodium_falciparum_Sanger|(protein coc			
	R.NNNVCI	4.0517	0.3079	100	1888.933	1888.924		5429.1	1	6.843	63.3	3
	K.IEYMQV	3.3695	0.2741	100	1179.611	1179.606		7106.1	1	6.484	87.5	1
PFA0505c		9	6.80%	249	29688	4.8	U		Annotation|Plasmodium_falciparum_Sanger|(protein coc			
	K.NKQEEE	4.5415	0.3427	100	2109.02	2108.015		5580.8	1	5.311	40.6	3
	K.NKQEEE	5.112	0.3071	100	2112.031	2108.015		6888.6	1	6.01	68.8	6
PFF0420c		12	6.80%	235	26528	5.5	U		Annotation|Plasmodium_falciparum_Sanger|(protein coc			
	K.DIEIEDA	4.0484	0.358	100	1823.057	1823.054		5374.9	1	6.353	66.7	12
PF13_0280		54	6.80%	221	26447	9.2	U		Annotation|Plasmodium_falciparum_Sanger|(protein coc			
	K.YLIPPC	3.3249	0.3557	100	1797.013	1797.013		3691.6	1	6.371	60.7	3
	I.PPCLVLS	4.2627	0.4151	100	1400.765	1400.765		7970.5	1	8.352	81.8	51
PF13_0099		2	6.80%	205	23193	9.1	U		Annotation|Plasmodium_falciparum_Sanger|(protein coc			
	R.IDVIGG(	4.266	0.293	100	1372.787	1372.784		6316	1	6.256	84.6	2
PF10_0344		11	6.70%	1233	141051	4.3	U		Annotation|Plasmodium_falciparum_TIGR|(protein codin			
	K.ADNDGI	5.0213	0.4186	100	2798.198	2796.183		7682.4	1	8.966	45.8	3
	K.VEHEIV	5.2477	0.2452	100	2278.152	2278.146		6088.7	1	7.875	61.1	6
	K.GQHEIV	5.0311	0.3001	100	4541.353	4540.339		5409.9	2	5.771	19.3	1
	K.GQHEIV	4.4845	0.3069	99.9	4541.395	4540.339		8588.8	12	5.66	17.1	1
PFL0310c		29	6.70%	984	116014	5.4	U		Annotation|Plasmodium_falciparum_Sanger|(protein coc			
	K.KPIVSA	3.0392	0.2748	99.9	1098.626	1098.627		4167	1	5.985	83.3	2
	R.LHFYET	5.5183	0.4409	100	3006.422	3004.415		8246.2	1	7.152	37.5	12
	K.ESADCII	4.7393	0.3329	100	1869.918	1868.91		3701.2	1	7.016	66.7	9
	K.LNLYNE	4.5434	0.418	100	1867.846	1867.846		7988.2	1	7.819	78.6	6
MAL13P1.120		9	6.70%	864	100943	5.7	U		Annotation|Plasmodium_falciparum_Sanger|(protein coc			
	K.INGSSS	3.529	0.2441	99.8	1647.823	1647.819		4273.3	1	4.725	71.4	2
	K.ELKNDN	3.183	0.293	99.8	2151.025	2150.015		3890.3	2	5.282	44.1	2
	K.HKNDNS	3.7867	0.4801	100	1598.773	1598.764		4923.3	1	8.111	70.8	3
	R.DIYEFF	3.2912	0.3279	100	1407.7	1404.669		9072	1	6.258	77.3	2
PFI0820c		14	6.70%	388	45143	6.6	U		Annotation|Plasmodium_falciparum_Sanger|(protein coc			
	R.LVADPF	3.5088	0.4022	100	1547.755	1547.749		5748.1	1	6.842	75	6
	K.LEECVI	3.9855	0.3899	100	1555.747	1555.743		7326.4	1	6.662	79.2	8
PFI0300w		7	6.70%	195	21851	7.5	U		Annotation|Plasmodium_falciparum_Sanger|(protein coc			
	K.LDGQIG	4.0498	0.4214	100	1391.779	1391.783		7200.7	1	6.672	79.2	7
PF14_0492		4	6.70%	179	20426	4.9	U		Annotation|Plasmodium_falciparum_TIGR|(protein codin			
	K.VSFVEF	4.0929	0.3902	100	1338.771	1338.767		7781.9	1	8.621	86.4	4

PFE1400c		13	6.60%	893	102049	5.7	U		Annotation|Plasmodium_falciparum_Sanger|(protein coc				
	K.LSPSLV	4.7615	0.4901	100	2329.349	2327.341		8172.7	1	7.743	47.5	9	
	K.SMDLIG	3.897	0.4952	100	1796.885	1796.881		7049	1	8.082	66.7	3	
	K.NSFGLS	4.3728	0.4057	100	2345.172	2345.149		8653.3	1	7.71	42.9	1	
PF10_0298		5	6.60%	393	46801	6.1	U		Annotation|Plasmodium_falciparum_TIGR|(protein codin				
	K.LHHELT	2.4585	0.3568	99.8	1346.742	1346.743		5060.5	2	5.625	60	1	
	K.QREDIS	4.3649	0.4633	100	1911.942	1911.932		3893.5	1	7.399	75	4	
PFE0350c		7	6.60%	411	46212	10.5	U		Annotation|Plasmodium_falciparum_Sanger|(protein coc				
	K.NVVGE\	4.1897	0.4299	100	1910.104	1910.094		5399.1	1	7.019	65.6	1	
	K.SIVHNPI	3.0235	0.4229	100	1227.668	1227.667		5424.3	1	7.066	83.3	6	
PFD1130w		13	6.60%	362	42776	5.6	U		Annotation|Plasmodium_falciparum_Sanger|(protein coc				
	K.VKDSWI	4.2657	0.4435	100	1579.86	1578.853		8392.8	1	7.613	83.3	10	
	K.LLSSYV.	3.1487	0.3496	100	1219.734	1219.73		6920.2	1	6.113	85	3	
PF08_0006		3	6.60%	272	30558	8.7	U		Annotation|Plasmodium_falciparum_Sanger|(protein coc				
	H.TQHLPY	3.4192	0.3277	100	2144.014	2142.026		5605.5	1	5.95	47.1	3	
PF11_0427		6	6.60%	259	30101	8.5	U		Annotation|Plasmodium_falciparum_TIGR|(protein codin				
	K.SKLETT	4.109	0.4	100	1963.101	1963.087		6986.1	1	7.255	68.8	6	
PF11_0293		4	6.60%	136	15525	9.9	U		Annotation|Plasmodium_falciparum_TIGR|(protein codin				
	K.KLTQAC	3.0009	0.2289	99.8	1028.621	1028.622		4695.4	1	4.535	87.5	4	
PF14_0428		42	6.50%	1132	133668	7.7	U		Annotation|Plasmodium_falciparum_TIGR|(protein codin				
	K.KPPVDC	3.0929	0.355	100	1093.717	1093.717		3541.5	1	6.356	72.2	2	
	K.VSSCVH	4.7349	0.5094	100	1792.811	1792.811		8565.4	1	9.67	80	15	
	K.LVTSHF	3.7645	0.3349	100	1498.753	1498.758		7177.1	1	7.529	77.3	3	
	K.LEIQNC	3.8932	0.2923	100	2240.179	2240.175		6530.9	1	5.794	55.9	3	
	K.QIPLVLI	4.0674	0.3237	100	2012.237	2012.249		7716.5	1	6.693	35.3	1	
	K.QIPLVLI	5.7314	0.5258	100	2012.239	2012.249		8679.2	1	10.281	67.6	18	
PFL1340c		5	6.50%	771	91292	5.9	U		Annotation|Plasmodium_falciparum_Sanger|(protein coc				
	K.KETNEN	4.8563	0.2141	99.8	2718.293	2717.284		9401.4	1	5.58	35.2	1	
	K.GHDVIT	7.0313	0.4891	100	3128.63	3127.621		7206.8	1	8.075	38.5	4	
PFC0720w		22	6.50%	461	54677	7.3	U		Annotation|Plasmodium_falciparum_Sanger|(protein coc				
	K.LVDLLT/	4.8025	0.4482	100	1973.116	1973.126		7289	1	8.292	65.6	12	
	K.NILLITE	4.3771	0.363	100	1512.891	1512.9		8475.2	1	7.188	87.5	10	
PF11_0177		7	6.50%	465	54460	5.6	U		Annotation|Plasmodium_falciparum_TIGR|(protein codin				
	R.NIHNSY	3.8539	0.3379	100	1717.939	1715.931		6805.2	1	6.532	65.4	6	
	K.STLEIN	3.5724	0.4121	100	1923.029	1923.03		6410.6	1	7.379	53.3	1	
PF11_0443		16	6.50%	321	39362	9.6	U		Annotation|Plasmodium_falciparum_TIGR|(protein codin				

	K.VLHHFY	4.9821	0.4626	100	2646.203	2645.199		5957.3	1	7.888	47.5	16
PF13_0228		52	6.50%	306	35384	10.4	U		Annotation|Plasmodium_falciparum_Sanger|(protein coc			
	R.IGNAVP	4.4833	0.3948	100	2145.089	2142.069		3941.8	1	6.916	55.3	52
PFL0180w		2	6.50%	184	21846	9.4	U		Annotation|Plasmodium_falciparum_Sanger|(protein coc			
	Y.YPSQKQ	3.0276	0.2709	99.8	1504.763	1504.744		6692.5	1	4.613	63.6	2
MAL13P1.288		12	6.50%	153	18422	9.3	U		Annotation|Plasmodium_falciparum_Sanger|(protein coc			
	K.NVSNFF	3.3942	0.3821	100	1294.612	1294.611		4036	1	6.589	77.8	12
PFC0150w		27	6.40%	1171	135864	8.9	U		Annotation|Plasmodium_falciparum_Sanger|(protein coc			
	K.VKEVEN	3.6563	0.2131	99.9	1474.771	1474.775		4501.2	2	4.961	72.7	4
	K.GDQDAI	3.7194	0.3866	100	1622.714	1621.709		5408.1	1	6.283	73.1	2
	R.FDKKDF	3.4074	0.2427	99.9	1415.699	1415.696		4045.5	2	5.233	85	3
	R.VALPLL	6.3234	0.5084	100	2514.494	2514.481		9838.5	1	8.98	45.5	5
	R.VALPLL	5.9724	0.4649	100	2515.489	2514.481		9991	1	9.763	59.1	11
	K.SLRPTS	4.0077	0.2211	99.8	1714.953	1713.95		8288.9	1	5.374	67.9	2
PF07_0129		27	6.40%	811	93284	8.9	U		Annotation|Plasmodium_falciparum_Sanger|(protein coc			
	S.NENFHN	5.0994	0.4829	100	2178.096	2178.095		6284.6	1	8.185	55.9	16
	R.IYTNIM	3.0753	0.3158	99.9	2037.1	2037.084		6704.9	1	5.467	50	2
	R.KANNNC	5.0077	0.477	100	1900.97	1900.956		6786.2	1	8.819	75	3
	K.ANNNGS	3.7323	0.4069	100	1772.854	1772.861		6545.4	1	7.331	60	6
PF11_0171		3	6.40%	485	56531	8	U		Annotation|Plasmodium_falciparum_TIGR|(protein codin			
	K.IVCTCSS	2.9177	0.2978	99.8	1406.642	1406.641		4048.5	1	6.262	68.2	2
	K.FIWIND	4.0883	0.3606	100	2357.18	2357.179		6032.8	1	6.736	44.4	1
MAL8P1.8		3	6.40%	438	51310	6.1	U		Annotation|Plasmodium_falciparum_Sanger|(protein coc			
	K.NNTTTN	5.4432	0.4403	100	3115.455	3114.444		4196.4	1	7.153	27.8	3
MAL13P1.242		1	6.40%	204	24482	9.6	U		Annotation|Plasmodium_falciparum_Sanger|(protein coc			
	N.LNNNLI	3.5873	0.2353	99.9	1509.894	1507.892		6543	1	5.025	75	1
PFL0675c		14	6.30%	1093	129284	5.2	U		Annotation|Plasmodium_falciparum_Sanger|(protein coc			
	R.ELFNNY	4.1627	0.4479	100	2087.997	2086.992		5103.4	1	7.073	60	3
	K.NKEEDI	3.2901	0.3335	100	1531.783	1531.783		8632.8	1	6.078	81.8	1
	K.NKEEDI	4.0166	0.3508	100	1659.879	1659.878		5524.2	1	7.635	66.7	1
	K.EVSDII	4.2733	0.5275	100	1652.767	1652.764		7679.9	1	8.59	70.8	3
	K.EVANSN	3.7228	0.4092	100	1856.947	1856.947		6297.5	1	6.662	53.3	3
	K.HFNDIN	3.6776	0.328	100	1383.609	1383.608		8082.1	1	7.873	85	3
PFF0850c		3	6.30%	302	33958	8.8	U		Annotation|Plasmodium_falciparum_Sanger|(protein coc			
	K.KGTKPV	4.5962	0.5009	100	2033.057	2033.045		5400.3	1	8.838	58.3	3
PFD0115c		1	6.30%	254	31019	7.8	U		Annotation|Plasmodium_falciparum_Sanger|(protein coc			

	K.KLWNEY	3.6614	0.3156	100	2072.164	2072.148		5213.2	1	5.702	63.3	1
PF14_0335		12	6.30%	190	21835	9.5	U		Annotation|Plasmodium_falciparum_TIGR|(protein codin			
	R.SGDYFY	4.0057	0.409	100	1501.714	1501.712		6228.3	1	7.633	77.3	12
MAL7P1.176		42	6.20%	1462	169910	6.1	U		Annotation|Plasmodium_falciparum_Sanger|(protein coc			
	R.RQELCL	3.0827	0.3402	100	1380.706	1380.713		5752.3	1	6.101	75	1
	K.INTNSN	3.2767	0.3752	100	1217.602	1217.603		6430.8	1	6.527	83.3	7
	R.NNEQTS	5.2981	0.5855	100	2276.06	2276.051		4404	1	9.495	47.4	6
	R.ESDDGE	5.6223	0.4948	100	2980.209	2976.188		7566	1	9.017	48.1	27
	K.MKGND	4.0513	0.2942	99.8	2627.151	2625.138		8799.2	1	5.794	42	1
PF14_0360		8	6.20%	661	76833	9	U		Annotation|Plasmodium_falciparum_TIGR|(protein codin			
	K.KVDMNI	2.8585	0.2956	99.9	1019.556	1019.553		4914.2	1	5.751	92.9	2
	R.NNNIIN	3.7954	0.2398	99.9	1971.002	1971.001		4650.7	1	5.252	50	3
	R.INFEELY	3.9563	0.224	99.9	1987.109	1987.121		7046.5	1	5.109	53.3	3
PFB0835c		11	6.20%	519	60598	7.9	U		Annotation|Plasmodium_falciparum_TIGR|(protein codin			
	K.KVDFFD	4.5236	0.4652	100	2017.135	2016.128		8384	1	7.759	71.9	7
	R.NDKYNI	4.4183	0.2192	99.9	1829.964	1828.96		8957.7	1	6.186	78.6	3
	K.YNFVTV	2.9715	0.2858	99.8	1471.798	1471.795		8860.9	1	5.501	63.6	1
PF13_0242		19	6.20%	468	53594	8.6	U		Annotation|Plasmodium_falciparum_Sanger|(protein coc			
	R.DKTNDC	3.9822	0.4388	100	1946.031	1946.027		4495.6	1	7.264	50	3
	K.SFEQHN	3.7222	0.4058	100	1617.745	1617.745		7400.8	1	7.706	81.8	16
PFL0110c		30	6.20%	324	36801	9.5	U		Annotation|Plasmodium_falciparum_Sanger|(protein coc			
	R.SLTLGW	4.1462	0.5203	100	2229.148	2228.143		6234.1	1	8.238	55.3	30
PFL0960w		2	6.20%	227	25549	6.5	U		Annotation|Plasmodium_falciparum_Sanger|(protein coc			
	K.AIIAPSV	3.44	0.4468	100	1383.825	1383.821		6479.2	1	8.28	61.5	2
PFI0355c		23	6.10%	922	106462	8.3	U		Annotation|Plasmodium_falciparum_Sanger|(protein coc			
	K.STIASE	3.4571	0.306	99.9	1709.886	1709.882		4968	1	5.563	56.7	5
	K.THNNLL	5.1626	0.4952	100	1626.943	1626.94		8939.6	1	9.318	80.8	12
	K.FTDDAI	3.4457	0.337	100	2926.353	2926.368		9185.9	1	6.452	32	2
	K.FTDDAI	4.7896	0.4749	100	2927.378	2926.368		10067.5	1	7.394	32	4
PFA0480w		54	6.10%	575	67720	8.6	U		Annotation|Plasmodium_falciparum_Sanger|(protein coc			
	K.KNEELY	5.517	0.3981	100	2460.283	2459.278		7695.6	1	7.806	63.2	30
	K.NEELYN	6.3422	0.4265	100	2334.191	2331.183		9150.9	1	8.919	66.7	21
	R.VHTHG	3.2553	0.4692	100	1767.79	1767.788		5668.3	1	8.385	64.3	3
PF13_0293		3	6.10%	509	60023	8.7	U		Annotation|Plasmodium_falciparum_Sanger|(protein coc			
	R.AGYCLV	3.7508	0.4914	100	2246.158	2246.154		7071.8	1	7.784	58.3	2
	K.IDYLTN	2.821	0.3156	99.8	1545.764	1544.761		5274.9	1	5.552	63.6	1

PF08_0120		49	6.10%	491	56501	7.7	U	Annotation|Plasmodium_falciparum_Sanger|(protein coc				
	K.SLIEEV	3.303	0.3194	99.9	1736.846	1736.834	6567.5	1	6.339	65.4	6	
	K.MYLDNL	5.3248	0.3723	100	1971.004	1971.015	10675.3	1	7.844	70	43	
MAL8P1.96		23	6.10%	313	37117	6.5	U	Annotation|Plasmodium_falciparum_Sanger|(protein coc				
	R.DFNSVD	5.1541	0.3245	100	2267.122	2267.12	8917.4	2	7.3	38.9	9	
	R.DFNSVD	5.5554	0.4567	100	2268.126	2267.12	8250.8	1	8.296	61.1	14	
PF11_0200		30	6.10%	294	35309	7.1	U	Annotation|Plasmodium_falciparum_TIGR|(protein codin				
	K.AADHFE	5.8328	0.5067	100	2249.98	2248.975	10988.1	1	10.301	73.5	30	
PFF0290w		8	6.10%	293	34732	9.7	U	Annotation|Plasmodium_falciparum_Sanger|(protein coc				
	-.MNNLNI	4.8475	0.4912	100	2135.134	2135.151	9236.3	1	8.001	55.9	8	
PF10_0150		6	6.00%	517	59724	7.1	U	Annotation|Plasmodium_falciparum_TIGR|(protein codin				
	R.KYWVYI	2.917	0.2896	99.9	1366.674	1366.68	7625.3	1	6.127	72.2	3	
	K.SCCTSV	3.4473	0.3906	100	2519.206	2519.203	7005.1	1	7.207	42.5	3	
PFL1725w		20	6.00%	535	58394	6.4	U	Annotation|Plasmodium_falciparum_Sanger|(protein coc				
	L.SQPFAV	3.2163	0.3288	99.9	1690.88	1690.892	7490.6	1	5.15	60	3	
	S.QPFAVA	3.1817	0.3067	99.9	1603.851	1603.86	7627	1	6.352	64.3	6	
	R.FVELED	5.0522	0.4662	100	1852.992	1852.995	7630.5	1	8.04	83.3	11	
PF13_0154		8	6.00%	332	38776	6.6	U	Annotation|Plasmodium_falciparum_Sanger|(protein coc				
	K.MTVDEY	2.8244	0.321	99.9	1157.486	1157.479	5856.5	2	5.671	81.2	3	
	K.LHDDKD	3.4544	0.2028	99.8	1427.718	1426.711	7216.3	1	6.163	70	5	
PFA0010c		5	6.00%	331	37416	8.7	U	Annotation|Plasmodium_falciparum_Sanger|(protein coc				
	K.LIPISVI	4.0454	0.4734	100	2314.38	2314.362	6626.2	1	7.033	47.4	5	
PFC0085c		6	6.00%	317	37389	8.1	U	Annotation|Plasmodium_falciparum_Sanger|(protein coc				
	R.VTVLEP	4.8221	0.4571	100	2254.008	2253.005	3886.4	1	7.938	69.4	6	
PF11_0111		48	5.90%	1483	173923	9.2	U	Annotation|Plasmodium_falciparum_TIGR|(protein codin				
	R.WADMC	4.4918	0.4257	100	2793.068	2792.058	6719.9	1	9.576	47.7	3	
	K.VVDNN	5.0244	0.4643	100	1869.783	1869.786	5639.7	1	8.374	80	9	
	R.NLHIDIT	5.3705	0.4784	100	2375.255	2374.234	6745.8	1	9.131	63.9	8	
	K.NINVSV	5.0368	0.527	100	2043.055	2043.067	5540.2	1	8.976	59.4	1	
	K.HYKDDK	4.3196	0.4105	100	1466.668	1463.656	6677.9	1	7.004	90	12	
	K.HYKDDK	4.8092	0.3715	100	1594.766	1591.751	7060.6	1	7.147	90.9	15	
PFL1415w		7	5.90%	990	117482	5.9	U	Annotation|Plasmodium_falciparum_Sanger|(protein coc				
	Y.NNSNN	3.6307	0.3215	100	3048.348	3048.343	4100.5	1	6.246	30	3	
	K.YFHKNE	3.2501	0.2761	99.9	1600.809	1600.809	3736.1	1	5.692	59.1	1	
	K.NGVNVD	3.7665	0.2487	99.9	2232.072	2231.081	5133.3	1	5.545	44.7	3	
PFF0345w		21	5.90%	977	112358	8	U	Annotation|Plasmodium_falciparum_Sanger|(protein coc				

	K.KKEDLD	4.2971	0.2342	99.9	1915.067	1914.044		6977.1	1	5.477	42.2	2
	K.DIVLDN	5.9158	0.5031	100	2086.04	2086.032		8875	1	8.829	68.8	15
	K.KQKPDT	3.1163	0.2887	99.9	1657.8	1657.794		6693	1	6.159	62.5	3
	K.ICGEPH	2.8912	0.2696	99.8	1268.625	1267.622		3338.9	1	5.793	70	1
PF08_0003		13	5.90%	675	80443	6.2	U		Annotation|Plasmodium_falciparum_Sanger|(protein coc			
	K.DLATNL	4.0563	0.3532	100	1596.867	1596.864		9857.8	1	7.326	69.2	3
	K.KKENITA	4.5986	0.3977	100	1288.789	1287.782		5832.7	1	7.795	86.4	1
	K.NKEYDI	5.216	0.4431	100	1798.868	1798.866		6076.1	1	8.113	76.9	9
PF13_0133		21	5.90%	590	68481	7.6	U		Annotation|Plasmodium_falciparum_Sanger|(protein coc			
	K.DCGIHM	4.0969	0.4765	100	1968.887	1968.895		6673.3	1	7.64	60	5
	R.LKLTNE	3.4792	0.4496	100	2298.243	2298.239		7322.6	1	6.774	47.2	6
	R.LKLTNE	4.0033	0.2822	99.9	2299.247	2298.239		8460.3	1	6.113	38.9	1
	K.LTNESL	4.6082	0.4551	100	2057.064	2057.06		6928.4	1	8.27	65.6	9
MAL7P1.27		12	5.90%	424	48675	8.6	U		Annotation|Plasmodium_falciparum_Sanger|(protein coc			
	R.YRELDN	3.1522	0.4099	100	1749.858	1749.852		7118.7	1	6.083	71.4	3
	K.NGFACL	3.4088	0.4039	100	1154.57	1154.578		5761.7	1	7.103	88.9	9
PFA0230c		3	5.90%	255	30564	8.5	U		Annotation|Plasmodium_falciparum_Sanger|(protein coc			
	R.NTLITE	3.6199	0.4289	100	1574.831	1574.836		7461.5	1	7.58	75	3
PF08_0096		28	5.80%	941	108635	9	U		Annotation|Plasmodium_falciparum_Sanger|(protein coc			
	K.TAGYLL	3.5139	0.316	100	2141.222	2141.209		6030.6	1	6.018	55.6	1
	K.FCFETG	5.2172	0.5166	100	2170.158	2170.156		6436	1	8.728	52.8	13
	K.HVINFDI	4.9156	0.4912	100	2089.069	2089.077		7478.8	1	8.621	68.8	11
	K.HVINFDI	5.6309	0.4699	100	2089.071	2089.077		7966.6	1	8.096	53.1	3
		16	5.80%	590	62461	8.1	U		no description			
	K.NKYEDE	2.9269	0.2513	99.8	1308.657	1308.655		5918.4	1	4.991	83.3	1
	R.NLDLDS	4.4547	0.2712	100	1332.738	1329.727		5961.4	1	6.08	81.8	12
	R.SRTEAE	3.3506	0.3307	100	1485.7	1485.697		7468.9	1	6.024	72.7	3
PF14_0601		3	5.80%	344	39232	7.1	U		Annotation|Plasmodium_falciparum_TIGR|(protein codin			
	K.YRPNVL	4.0215	0.3998	100	2339.289	2339.272		6739.2	1	6.778	44.7	2
	K.YRPNVL	5.6598	0.5544	100	2368.361	2367.341		9998	1	8.384	44.7	1
PF14_0241		1	5.80%	171	19393	9	U		Annotation|Plasmodium_falciparum_TIGR|(protein codin			
	K.FNEHKII	3.0699	0.228	99.8	1271.687	1271.694		4982.9	1	4.769	83.3	1
PFD1145c		7	5.70%	526	62996	8.5	U		Annotation|Plasmodium_falciparum_Sanger|(protein coc			
	K.EGHLDF	4.0249	0.3317	100	2371.191	2370.181		5183.6	1	5.487	63.9	2
	K.KLEHPY	3.9433	0.2154	99.9	1370.706	1370.707		9583.3	1	5.506	90	5
PF14_0020		3	5.70%	440	52207	5.5	U		Annotation|Plasmodium_falciparum_TIGR|(protein codin			

	R.IAPLLLN	3.0939	0.4243	100	1386.778	1386.775		4869.4	1	7.846	75	2	
	K.SILVGIA	3.2873	0.4396	100	1197.778	1197.776		6861.2	1	7.287	77.3	1	
PF11_0438		21	5.70%	140	16265	10.5	U		Annotation|Plasmodium_falciparum_TIGR|(protein codin				
	K.HAQFYV	2.8598	0.3369	100	949.4896	949.4896		6193	1	6.167	92.9	21	
PF10_0213		43	5.60%	2290	267385	9.3	U		Annotation|Plasmodium_falciparum_TIGR|(protein codin				
	R.FNKEEQ	5.5378	0.3542	100	2213.015	2213.022		4242.2	1	6.954	67.6	6	
	K.NKDFEV	3.557	0.3468	100	1509.747	1509.745		4937.2	1	5.464	54.5	2	
	K.NKDFEV	4.2816	0.405	100	1516.765	1516.762		6234.9	1	7.089	86.4	5	
	K.GIEEIFE	4.8831	0.3805	100	2302.095	2300.082		10800.3	1	7.398	58.3	13	
	K.GIEEIF	5.1752	0.3167	100	2576.251	2575.246		6300.4	1	6.165	37.5	4	
	K.INDNKD	3.9554	0.2573	100	1716.866	1716.859		4933.1	2	5.309	65.4	1	
	K.NDANIH	2.7146	0.3772	99.9	1875.989	1875.99		6329.6	1	5.672	40.6	1	
	R.LPIHSA	4.7024	0.5082	100	1714.948	1713.944		7975	1	9.255	73.3	7	
	K.GNNVGT	2.8191	0.304	99.8	1282.686	1282.687		6077.2	1	6.192	77.3	2	
	R.MQDSY(	3.8606	0.3041	100	2132.969	2131.962		5801.7	1	6.176	52.9	2	
PF14_0316		16	5.60%	1472	169227	8.3	U		Annotation|Plasmodium_falciparum_TIGR|(protein codin				
	K.GGSHVN	5.0444	0.5126	100	1939.047	1938.041		9488.6	1	9.161	70.6	1	
	R.SIPNIM	3.2021	0.3982	100	1598.821	1598.811		4436.1	1	7.045	57.7	2	
	K.IDNDTLE	3.7916	0.4026	100	1741.963	1741.959		9187.9	1	6.982	67.9	3	
	K.EFLEEL	3.3457	0.2309	99.9	1365.683	1365.679		8918.4	1	4.582	85	3	
	K.VLVEEL	2.9232	0.2561	99.9	1020.574	1020.573		4170.8	1	5.298	92.9	3	
	K.DYDYLL	3.754	0.2722	99.9	2049.037	2048.03		6491.5	3	5.275	46.9	4	
PF13_0095		9	5.60%	1005	115217	6.4	U		Annotation|Plasmodium_falciparum_Sanger|(protein coc				
	R.LPHFID	3.204	0.2887	99.9	1567.867	1567.867		3578.7	3	6.05	54.2	1	
	R.SISEYIL	2.8948	0.3158	99.9	1230.686	1230.685		6232.5	2	5.36	72.2	2	
	K.NTFELW	3.3001	0.4187	100	1943.817	1943.817		5063.6	1	6.737	53.6	3	
	R.FDLIYL	4.407	0.4076	100	2210.12	2210.117		5213.9	1	7.701	64.7	3	
MAL13P1.163		11	5.60%	284	32806	7.3	U		Annotation|Plasmodium_falciparum_Sanger|(protein coc				
	K.VAAFTT	4.1358	0.5101	100	1725.896	1725.897		10151	1	8.661	63.3	11	
MAL8P1.31		13	5.60%	266	30792	5.8	U		Annotation|Plasmodium_falciparum_Sanger|(protein coc				
	K.NYSFLW	4.752	0.5002	100	1926.985	1926.983		8773.8	1	8.314	67.9	13	
PF11_0086		87	5.50%	3334	381873	7	U		Annotation|Plasmodium_falciparum_TIGR|(protein codin				
	K.NLSHN	6.0607	0.4512	100	3155.375	3154.363		4104.7	1	7.837	30.4	3	
	Y.NNEHV	6.1455	0.5503	100	2266.975	2265.968		8769.4	1	9.716	71.1	3	
	K.KEDFEE	4.6028	0.1892	100	1409.68	1409.68		8291.6	2	5.644	90	8	
	R.VNINND	3.9729	0.2887	100	1631.834	1630.829		6397	1	6.321	76.9	4	

	K.EKNDKN	4.1496	0.1382	99.9	1412.698	1409.691		3753.7	2	4.666	85	18
	K.VNDEGT	5.4981	0.1487	100	1715.873	1715.87		8567.4	1	4.575	57.1	3
	K.VNDEGT	5.5224	0.2987	100	1718.882	1715.87		9518.3	1	6.564	78.6	24
	K.VQNADE	3.9364	0.3831	100	1552.757	1552.757		6729.2	1	7.091	75	3
	K.KNIVND	2.8077	0.2964	99.9	1223.673	1223.671		4435.5	5	4.997	66.7	1
	K.EGNLHL	5.0149	0.3416	100	2884.414	2883.402		8343.8	1	6.261	33.3	14
	K.KNNNIS	4.3827	0.2378	99.9	1830.931	1830.931		5292.3	1	6.503	70	4
	K.VNEILE	4.3832	0.4611	100	2405.244	2404.232		7369.4	1	7.73	57.9	2
PF14_0495		101	5.50%	2189	249485	8.7	U		Annotation|Plasmodium_falciparum_TIGR|(protein codin			
	K.NNMHN	5.8699	0.6023	100	2533.066	2533.055		4394.2	1	10.956	61.4	6
	G.AGFSDS	4.5571	0.535	100	2277.967	2277.964		6711.9	1	9.364	52.5	3
	K.EHYSSD	2.6756	0.393	100	1355.562	1355.562		3846	1	7.109	75	3
	K.EYLCHL	4.9734	0.3432	100	2187.096	2187.102		9110.9	1	8.071	55.9	28
	K.EYLCHL	4.7357	0.3554	100	2187.115	2187.102		7228.3	1	7.344	39.7	18
	S.KGFEEY	2.5862	0.2863	99.8	1056.548	1056.548		4451.7	1	6.266	85.7	3
	R.ALGLWL	5.3024	0.4258	100	2387.152	2386.172		6387.7	1	8.071	62.5	39
	K.MILGGD	3.4907	0.3249	100	2025.088	2023.08		6874.7	1	6.157	50	1
PF10_0361		45	5.50%	1681	192630	9.3	U		Annotation|Plasmodium_falciparum_TIGR|(protein codin			
	K.LIEIISP	3.9159	0.3632	100	1943.041	1942.033		5611.3	1	6.414	66.7	2
	K.KNSFTP	3.0434	0.3253	99.9	1276.666	1276.665		6787.2	5	7.115	65	4
	K.HQGEHI	4.1192	0.449	100	1707.791	1706.785		5174.2	1	7.906	75	24
	F.KTLNDV	4.6133	0.4284	100	1578.835	1578.835		5224.6	1	7.573	75	7
	K.TLNDVN	3.9643	0.3983	100	1451.748	1450.74		8320.4	1	7.193	72.7	3
	K.ISNLEE	3.1649	0.2923	99.9	1626.811	1626.809		4427	1	6.247	69.2	2
	K.REDYHN	6.2518	0.5576	100	2815.29	2815.297		9102.2	1	9.626	39.8	3
MAL13P1.63		16	5.50%	1192	139116	8.6	U		Annotation|Plasmodium_falciparum_Sanger|(protein coc			
	K.NNENLI	3.4205	0.2372	99.8	1522.804	1522.809		4648.3	5	4.821	58.3	1
	N.NNNNM	4.8197	0.3879	100	2152.896	2151.89		5050.6	1	6.259	52.6	3
	K.NVILNQ	5.041	0.3837	100	1799.956	1798.955		10718.5	1	7.946	80	6
	R.LNTDEI	4.7977	0.365	100	1829.975	1828.966		8478.4	1	5.951	70	6
PFL2365w		8	5.50%	1027	119605	4.8	U		Annotation|Plasmodium_falciparum_Sanger|(protein coc			
	K.KNELFE	4.6603	0.3127	99.9	3224.484	3223.505		10876.2	1	5.817	27.1	3
	R.ANKYDD	3.3729	0.2638	99.9	1389.701	1388.696		7265.3	1	6.213	80	3
	K.LYFNID	5.4536	0.4708	100	2569.351	2568.344		7848.6	1	7.428	43.4	2
PF13_0274		15	5.50%	884	103838	4.9	U		Annotation|Plasmodium_falciparum_Sanger|(protein coc			
	K.NIISST	3.2686	0.2559	99.8	1484.726	1484.723		6961.8	1	6.043	70.8	2

	K.NNNINE	3.9084	0.4083	100	1959.982	1959.978		6292.1	1	6.808	50	1	
	K.NLYSLL	4.0619	0.4343	100	2252.188	2250.181		6201.1	1	8.027	58.3	12	
PF11_0300		3	5.50%	642	76370	8.2	U		Annotation|Plasmodium_falciparum_TIGR|(protein codin				
	N.IYCNIY	3.3072	0.3602	100	2131.016	2131.019		6368.3	2	5.751	50	1	
	K.IKDWNF	3.9125	0.3643	100	2359.234	2359.209		6727	1	6.49	47.2	2	
PF13_0315		1	5.50%	509	57115	5.6	U		Annotation|Plasmodium_falciparum_Sanger|(protein coc				
	K.SSSDKD	4.7277	0.4227	100	3129.452	3128.446		9276.7	1	6.893	27.8	1	
PF14_0717		23	5.50%	328	38569	9.4	U		Annotation|Plasmodium_falciparum_TIGR|(protein codin				
	R.RQPDIA	4.115	0.408	100	2180.117	2180.114		6193	1	7.438	55.9	23	
PFB0305c		2	5.50%	272	30974	4.7	U		Annotation|Plasmodium_falciparum_TIGR|(protein codin				
	R.NNENEL	3.7578	0.2088	99.8	1701.835	1701.83		6354.5	1	6.083	64.3	2	
MAL13P1.180		3	5.40%	297	35497	9.5	U		Annotation|Plasmodium_falciparum_Sanger|(protein coc				
	K.AYENSK	3.8818	0.4232	100	1969.921	1968.913		5216.9	1	6.774	63.3	3	
MAL13P1.50		2	5.40%	205	24413	8.5	U		Annotation|Plasmodium_falciparum_Sanger|(protein coc				
	K.SNLIVNI	3.3975	0.3629	100	1260.734	1260.732		5726.7	2	5.882	75	2	
PF08_0086		22	5.30%	958	117080	9.2	U		Annotation|Plasmodium_falciparum_Sanger|(protein coc				
	K.SVEDNL	3.8564	0.4491	100	1611.704	1611.703		6034.7	1	8.738	76.9	3	
	K.KNEVKD	4.2906	0.3231	100	1607.853	1607.847		4736.2	1	7.105	73.1	1	
	K.DNTNYH	3.2238	0.2369	99.9	1250.58	1250.581		6271.5	2	5.388	77.8	3	
	R.NNKDDI	4.1095	0.3463	100	1650.769	1650.763		4951.1	1	6.228	79.2	15	
PF14_0352		50	5.30%	847	96904	7.2	U		Annotation|Plasmodium_falciparum_TIGR|(protein codin				
	K.RLPLPS	3.1949	0.2876	99.9	1480.839	1480.849		6662.5	2	5.352	58.3	2	
	K.NTSDDV	3.8295	0.4465	100	1889.887	1888.882		7710.6	1	7.51	59.4	21	
	R.VLSGEF	4.2876	0.2619	100	1702.974	1698.958		8477.8	1	5.49	75	27	
PF14_0393		10	5.30%	506	58787	4.7	U		Annotation|Plasmodium_falciparum_TIGR|(protein codin				
	R.FYYPHE	4.2969	0.2925	100	2200.975	2199.974		6959.9	1	6.403	62.5	9	
	K.NQHGIT	2.921	0.3961	99.8	1235.56	1235.559		3139.9	1	6.763	66.7	1	
PFA0670c		3	5.30%	243	28234	8.9	U		Annotation|Plasmodium_falciparum_Sanger|(protein coc				
	K.SEEQKC	4.0114	0.3104	100	1609.763	1609.761		7948.6	1	5.922	79.2	3	
MAL13P1.159		24	5.30%	206	23317	6	U		Annotation|Plasmodium_falciparum_Sanger|(protein coc				
	M.PVSHHE	3.5958	0.3869	100	1270.533	1267.531		6669.7	1	7.667	80	24	
PF14_0174		28	5.20%	466	52942	8.9	U		Annotation|Plasmodium_falciparum_TIGR|(protein codin				
	V.ALAIAQ	4.6061	0.5494	100	2440.369	2440.367		5649.6	1	8.754	50	7	
	L.AIAQMT	5.4342	0.5383	100	2257.248	2256.246		8144.6	1	9.845	52.4	11	
	A.IAQMTS	5.4341	0.5558	100	2185.211	2185.209		7561.3	1	9.781	52.5	8	
	I.AQMTST	3.8879	0.3257	100	2059.091	2058.091		6131.4	1	6.908	47.4	2	

PFF1375c		89	5.20%	387	44491	9.2	U		Annotation|Plasmodium_falciparum_Sanger|(protein coc			
	K.SGGNSL	5.8318	0.4424	100	2271.1	2270.092		7264.2	1	7.409	51.3	43
	K.SGGNSL	5.3266	0.4558	100	2278.116	2277.109		9651.4	1	8.373	57.9	44
	S.GGNSLL	4.3139	0.4644	100	2190.052	2190.077		7992.7	1	7.502	58.3	2
PF11_0117		2	5.20%	349	40347	8.8	U		Annotation|Plasmodium_falciparum_TIGR|(protein codin			
	R.VPLPSE	4.439	0.4281	100	2132.115	2132.114		6125.9	1	8.245	58.8	2
PF10_0028		40	5.20%	248	30051	10.1	U		Annotation|Plasmodium_falciparum_TIGR|(protein codin			
	K.RNEPHE	3.7022	0.4759	100	1553.732	1553.735		7138.8	1	8.216	83.3	36
	R.NEPHEF	3.0446	0.3745	99.8	1397.636	1397.634		5015	1	7.169	68.2	1
	R.NEPHEF	2.7148	0.3175	99.8	1397.637	1397.634		5630.8	3	5.767	54.5	3
PFI1075w		2	5.20%	248	28972	9.5	U		Annotation|Plasmodium_falciparum_Sanger|(protein coc			
	K.IHFNIFD	3.7327	0.4455	100	1668.799	1668.81		7999.2	1	6.984	70.8	2
PFL2465c		3	5.20%	210	24691	6.8	U		Annotation|Plasmodium_falciparum_Sanger|(protein coc			
	R.KIEDIHN	4.3239	0.3325	100	1344.783	1344.779		10221	1	6.897	90	3
PF14_0064		2	5.20%	194	21774	5.5	U		Annotation|Plasmodium_falciparum_TIGR|(protein codin			
	K.NIADSVI	2.6448	0.3187	99.9	1097.598	1097.596		4245.1	1	6.053	77.8	2
MAL8P1.53		8	5.10%	514	60543	9	U		Annotation|Plasmodium_falciparum_Sanger|(protein coc			
	K.INIYDNII	3.8111	0.281	100	1434.787	1434.784		6954.5	1	6.916	86.4	6
	K.STFINW	3.7093	0.2458	99.9	1781.946	1781.941		7703.8	1	5.647	57.7	2
PF11_0258		2	5.10%	294	34486	8.7	U		Annotation|Plasmodium_falciparum_TIGR|(protein codin			
	K.GIEMTE	3.2552	0.2791	99.8	1759.914	1759.905		7563.4	1	5.838	53.6	2
PFB0500c		1	5.10%	235	26671	8.6	U		Annotation|Plasmodium_falciparum_TIGR|(protein codin			
	K.NIFYML	3.2595	0.2346	99.8	1533.756	1533.766		5787.4	1	6.8	77.3	1
PFD0655c		41	5.00%	853	98471	5.3	U		Annotation|Plasmodium_falciparum_Sanger|(protein coc			
	K.IKNENI	4.6713	0.3696	100	1501.771	1499.765		8624.2	1	7.174	86.4	18
	R.KLNEIN	3.3094	0.2486	99.9	1445.726	1445.724		4894.9	1	5.681	77.3	2
	K.LLEDHK	5.0821	0.4925	100	2234.15	2233.139		9232	1	8.934	66.7	21
PFI0530c		3	5.00%	525	62346	9.1	U		Annotation|Plasmodium_falciparum_Sanger|(protein coc			
	K.FFMET	3.6958	0.3929	100	2885.287	2883.28		5303	1	6.334	40	3
PF14_0200		3	5.00%	504	58505	8.1	U		Annotation|Plasmodium_falciparum_TIGR|(protein codin			
	K.HIIGDA	3.9664	0.4664	100	2729.287	2728.283		5701.5	1	7.513	45.8	3
PF10_0360		9	5.00%	464	55783	9.2	U		Annotation|Plasmodium_falciparum_TIGR|(protein codin			
	K.EEFAIFN	4.3803	0.4239	100	2574.52	2573.52		6851.6	1	7.408	40.9	9
PFA0760w		2	5.00%	379	42305	8.8	U		Annotation|Plasmodium_falciparum_Sanger|(protein coc			
	R.DSIINA	3.7079	0.3237	100	2110.071	2110.071		7601.1	1	6.66	55.6	2
PF14_0280		1	5.00%	319	37442	6.7	U		Annotation|Plasmodium_falciparum_TIGR|(protein codin			

	K.SSIYNDI	3.5526	0.3028	99.9	1913.04	1912.044		7179.4	1	6.042	63.3	1
PFI0350c		17	5.00%	258	30661	6.1	U		Annotation|Plasmodium_falciparum_Sanger|(protein coc			
	K.NVFVRF	3.2366	0.4112	100	1568.869	1568.866		5308.3	1	6.226	66.7	17
PFE0845c		2	5.00%	260	28000	10.5	U		Annotation|Plasmodium_falciparum_Sanger|(protein coc			
	K.APLSVG	3.2752	0.5141	100	1292.814	1292.813		8676.4	1	8.305	66.7	2
PF11_0014		4	5.00%	218	25591	9.6	U		Annotation|Plasmodium_falciparum_TIGR|(protein codin			
MAL8P1.213		4	4.80%	231	27398	9.3	U		Annotation|Plasmodium_falciparum_Sanger|(protein coc			
	K.IKETCT	3.0706	0.3115	99.9	1415.7	1415.699		6214.4	1	5.913	85	4
PFA0125c		29	4.90%	1567	181149	5.1	U		Annotation|Plasmodium_falciparum_Sanger|(protein coc			
	K.VGVASE	5.8395	0.4905	100	2009.989	2009.985		5928.5	1	9.254	66.7	2
	R.EDPIAS	5.0231	0.4996	100	2082.948	2082.943		7147.6	1	8.905	72.2	9
	R.IITTEV	3.9998	0.3694	100	1856.015	1855.007		4893.3	1	6.571	68.8	3
	K.DLIDIHL	3.1633	0.3609	100	1121.688	1121.687		4897.1	1	6.393	87.5	12
	R.NKDLLE	3.5071	0.2905	99.9	1607.772	1607.77		6576.8	1	6.686	70.8	3
PF14_0198		12	4.90%	879	103162	7.9	U		Annotation|Plasmodium_falciparum_TIGR|(protein codin			
	K.LKDLYG	3.9688	0.2541	100	1519.831	1519.837		9414.9	1	5.367	70.8	3
	K.DLYGKF	3.1932	0.2952	99.9	1278.662	1278.658		5986.1	5	6.666	70	3
	R.SAYDLQ	2.7578	0.3742	100	1229.574	1229.574		8358	1	6.449	77.8	3
	K.KNWNEY	3.437	0.4139	100	2425.188	2424.195		8815.5	1	6.39	47.4	3
PF10_0368		17	4.90%	709	81526	6.4	U		Annotation|Plasmodium_falciparum_TIGR|(protein codin			
	K.HIDDFS	4.3383	0.3367	100	2686.373	2685.362		8550.2	1	6.792	47.8	2
	K.HIDDFS	5.4584	0.3997	100	2722.458	2720.448		8609.2	1	7.414	40.2	7
	K.SLINNYF	3.3627	0.4315	100	1366.758	1366.767		7543.5	1	6.986	80	8
		27	4.90%	653	72116	5.1	U		owl|P11021| 78 KD GLUCOSE REGULATED PROTEIN			
		27	4.90%	654	72347	5.2	U		owl|P06761| 78 KD GLUCOSE REGULATED PROTEIN			
		27	4.90%	655	72421	5.2	U		owl|P20029| 78 KD GLUCOSE REGULATED PROTEIN			
		27	4.90%	654	72379	5.2	U		owl|P07823| 78 KD GLUCOSE REGULATED PROTEIN			
	S.NPENTV	3.5382	0.357	100	1134.545	1134.543		6839.8	1	6.612	88.9	18
	S.NPENTV	3.0328	0.3783	99.8	1134.547	1134.543		3606.7	1	6.842	72.2	2
	T.VPAYFN	3.5628	0.4036	100	1181.58	1180.575		4808.1	1	7.211	88.9	3
	K.DAGTIA	3.1642	0.262	99.8	1217.636	1217.631		3846	20	5.237	77.3	4
PF10_0149		18	4.90%	593	69424	7.3	U		Annotation|Plasmodium_falciparum_TIGR|(protein codin			
	K.ITNLFV	3.1857	0.2921	99.9	1594.874	1594.867		6756.9	1	5.837	53.8	1
	K.SSKPN	3.503	0.3136	99.9	1751.816	1751.814		4971.9	1	6.162	60.7	15
	K.SSKPN	3.4903	0.3223	99.8	1751.824	1751.814		5838.7	1	6.581	60.7	2
PFE1155c		11	4.90%	536	62057	8.8	U		Annotation|Plasmodium_falciparum_Sanger|(protein coc			

	K.LFENNE	3.7278	0.3303	100	3133.565	3131.555		5380.8	1	6.444	32	11
		15	4.90%	471	51531	5.2	U		no description			
	R.YCMQL/	5.1941	0.4459	100	2739.337	2738.332		7231	1	8.174	56.8	2
	R.YCMQL/	4.4769	0.4905	100	2739.341	2738.332		7410.7	1	7.436	29.5	13
MAL13P1.329		2	4.90%	408	47021	8.5	U		Annotation|Plasmodium_falciparum_Sanger|(protein coc			
	R.LSPILP	3.5409	0.4653	100	2072.269	2071.261		6622.8	1	7.341	52.6	2
PF10_0114		2	4.90%	389	44395	4.5	U		Annotation|Plasmodium_falciparum_TIGR|(protein codin			
	R.LPELLQ	3.7676	0.2861	100	1176.69	1176.689		6552.7	2	6.804	88.9	1
	K.KLESLG	2.7318	0.2748	99.8	1018.594	1018.594		5176.7	1	4.815	87.5	1
PFI0935w		12	4.90%	370	43260	8.7	U		Annotation|Plasmodium_falciparum_Sanger|(protein coc			
	K.IYHPDK	5.0619	0.4172	100	2077.024	2076.023		7847.2	1	8.042	79.4	11
	K.IYHPDK	3.7068	0.3336	99.9	2077.026	2076.023		6369.7	1	6.606	36.8	1
PFC0945w		13	4.90%	328	39076	6.5	U		Annotation|Plasmodium_falciparum_Sanger|(protein coc			
	R.TLNYEC	4.3488	0.4413	100	2044.04	2044.04		6030.6	1	7.438	56.7	13
PF07_0117		34	4.90%	329	37820	5.3	U		Annotation|Plasmodium_falciparum_Sanger|(protein coc			
	K.LIAPPQ	3.9437	0.4884	100	1857.008	1854.997		2975.9	1	8.007	70	21
	A.PPQYVI	4.3265	0.4175	100	1550.774	1550.775		8732.5	1	8.246	79.2	13
PFF0905w		3	4.90%	284	33698	5.4	U		Annotation|Plasmodium_falciparum_Sanger|(protein coc			
	R.LPFYDD	3.4343	0.2611	99.9	1756.773	1756.767		6819.6	1	5.468	65.4	3
PF13_0227		2	4.90%	247	27801	9.1	U		Annotation|Plasmodium_falciparum_Sanger|(protein coc			
	R.RVNALN	2.8785	0.3065	99.8	1385.846	1385.846		5521	1	5.633	72.7	2
PFL1090w		3	4.90%	204	23632	4.5	U		Annotation|Plasmodium_falciparum_Sanger|(protein coc			
	K.RKDIDE	3.6186	0.3414	100	1251.678	1251.677		5499.1	1	5.91	88.9	3
PFL1300c		5	4.80%	495	57333	9.1	U		Annotation|Plasmodium_falciparum_Sanger|(protein coc			
	K.KGHFCE	3.0382	0.3746	100	1176.567	1175.563		6441.1	1	5.608	93.8	3
	R.KQPANS	3.5251	0.3043	99.9	1681.819	1681.815		4054	1	5.977	64.3	2
PF13_0032		7	4.80%	478	56811	9.5	U		Annotation|Plasmodium_falciparum_Sanger|(protein coc			
	K.YIFDSPI	3.0984	0.3209	99.9	1493.788	1493.779		7476.7	1	6.623	86.4	6
	K.NKQEIN	3.5021	0.2137	99.9	1373.768	1373.762		5368.1	27	4.484	65	1
PFL1745c		12	4.80%	459	53238	8.4	U		Annotation|Plasmodium_falciparum_Sanger|(protein coc			
	Q.ANDNS	5.0213	0.3516	100	2542.153	2541.146		8496.7	1	8.323	59.5	12
PFB0106c		2	4.80%	290	33742	9.7	U		Annotation|Plasmodium_falciparum_TIGR|(protein codin			
	K.ALDILEI	3.768	0.3264	100	1617.915	1617.91		9438.6	1	7.112	76.9	2
PF14_0277		16	4.70%	1394	162833	6.5	U		Annotation|Plasmodium_falciparum_TIGR|(protein codin			
	K.HHGVDI	3.2573	0.2424	99.8	1313.753	1313.752		4072.6	1	5.492	77.3	1
	K.INNIHNI	4.023	0.3084	100	1445.775	1445.776		5556.5	1	5.59	77.3	7

	K.ELGEEL	3.985	0.3789	100	1869.971	1869.956		7031.8	1	7.44	70	1	
	K.NNVNQV	3.7073	0.3871	100	2901.581	2900.575		6073.8	1	6.907	45.8	7	
PFL2120w		25	4.70%	1296	154902	7.8	U		Annotation|Plasmodium_falciparum_Sanger|(protein coc				
	K.KNDKVD	6.5806	0.3848	100	3076.377	3075.377		6435.9	1	6.622	41	3	
	K.NDKVDE	4.8279	0.3993	100	2955.332	2954.299		6551.3	1	6.673	43.8	1	
	K.NDKVDE	6.9058	0.4198	100	2957.311	2954.299		6675.2	1	7.994	39.6	3	
	K.VDEEED	4.4272	0.394	100	2598.14	2597.134		7386.3	1	7.404	57.1	3	
	K.LAPVIS	3.2091	0.3581	100	2494.237	2494.248		4665.9	2	6.143	38.1	3	
	K.LAPVIS	3.4284	0.373	100	2609.317	2608.308		5237.9	1	6.459	43.2	2	
	K.LAPVIS	5.1914	0.3038	100	2623.352	2622.343		7321	1	5.959	42	3	
	K.HLSLSE	3.5356	0.4502	100	1405.77	1405.78		6778.9	1	8.073	77.3	7	
PF10_0190		12	4.70%	937	112428	5.2	U		Annotation|Plasmodium_falciparum_TIGR|(protein codin				
	G.DNCVQI	3.5948	0.2883	99.9	1741.717	1741.709		4635.6	1	5.547	65.4	3	
	K.NKIPIN	4.9992	0.518	100	2181.137	2181.139		5550.1	1	8.864	70.6	3	
	K.YDKYEN	4.443	0.1544	99.9	1658.745	1657.739		7201.9	1	5.588	81.8	6	
PFL0580w		28	4.70%	758	85859	8.2	U		Annotation|Plasmodium_falciparum_Sanger|(protein coc				
	R.IEENLP	5.2707	0.4651	100	2590.228	2590.247		5980.6	1	8.276	57.5	27	
	R.ALSERP	3.3016	0.3884	100	1766.995	1765.993		6861.7	1	6.19	57.1	1	
PF14_0587		9	4.70%	677	79339	5.8	U		Annotation|Plasmodium_falciparum_TIGR|(protein codin				
	K.IPGLNV	4.5675	0.4704	100	1852.916	1850.907		6575.9	1	7.805	61.8	4	
	N.VSSPND	3.2333	0.3857	100	1356.624	1356.622		4429.7	1	6.276	66.7	3	
	A.NFISQH	4.0841	0.3797	100	1617.761	1617.762		4926.8	1	7.218	84.6	2	
PF11_0051		47	4.70%	623	72502	5.8	U		Annotation|Plasmodium_falciparum_TIGR|(protein codin				
	K.FTAGLE	5.3603	0.4991	100	1849.046	1847.039		9642	1	8.387	71.9	19	
	R.IVDIVLF	4.2714	0.4516	100	1407.839	1407.836		6627.5	1	7.958	86.4	28	
PFC0250c		7	4.70%	617	72443	8.5	U		Annotation|Plasmodium_falciparum_Sanger|(protein coc				
	K.KRDNHA	4.3644	0.4259	100	2125.071	2125.064		5587.2	1	7.099	61.8	6	
	K.IGQESTI	2.9675	0.285	99.9	1260.721	1260.721		6329	2	5.704	70	1	
PF08_0010		2	4.70%	552	65320	8.7	U		Annotation|Plasmodium_falciparum_Sanger|(protein coc				
	K.TYGINHT	3.0707	0.3473	100	1275.687	1275.688		4826.7	1	6.929	85	1	
	K.NYNINN	4.4946	0.4325	100	1958.882	1958.875		7383.5	1	8.584	78.6	1	
PF14_0239		22	4.70%	343	40249	5.1	U		Annotation|Plasmodium_falciparum_TIGR|(protein codin				
	K.LLTLEC	4.699	0.2553	100	1828.056	1826.046		7579.8	1	5.556	70	22	
PFE1025c		2	4.70%	301	35659	9.4	U		Annotation|Plasmodium_falciparum_Sanger|(protein coc				
	K.AANLLL	3.473	0.3536	100	1514.92	1514.917		5931.5	1	6.177	61.5	2	
PF08_0084		15	4.70%	235	27878	8.3	U		Annotation|Plasmodium_falciparum_Sanger|(protein coc				

	K.KPSFLN	3.9255	0.2307	100	1261.663	1260.659		6630.4	1	5.585	85	15
PF14_0689		1	4.70%	234	27202	6.8	U		Annotation|Plasmodium_falciparum_TIGR|(protein codin			
	K.NIMNDH	3.6562	0.3387	100	1371.71	1371.706		5753.7	1	6.597	80	1
PFL1160c		15	4.70%	192	22175	5.3	U		Annotation|Plasmodium_falciparum_Sanger|(protein coc			
	R.AWENY\	3.3143	0.2189	99.9	1174.575	1171.565		6377.7	1	6.046	87.5	15
PFB0640c		35	4.60%	1460	165488	6.8	U		Annotation|Plasmodium_falciparum_TIGR|(protein codin			
	K.IKQNEE	3.4351	0.2851	99.9	1968.938	1967.931		4749.7	1	5.352	65.6	2
	K.TSLLNV	3.6303	0.3062	100	1478.857	1478.854		7154.9	1	6.443	66.7	5
	N.YQHDN(	2.4128	0.341	99.8	1349.637	1349.635		4462.4	1	5.983	83.3	1
	K.LDCGAF	4.801	0.3551	100	3112.475	3111.452		7792	1	5.773	29.8	5
	K.LDCGAF	4.2996	0.408	100	3125.495	3125.486		6531.6	1	7.958	38.5	22
PF14_0706		27	4.60%	1253	142209	8.3	U		Annotation|Plasmodium_falciparum_TIGR|(protein codin			
	K.GNSMEI	4.3389	0.3691	100	1942.033	1939.023		5135	1	7.33	59.4	9
	K.KYNVVS	3.3227	0.3074	99.9	1486.782	1485.778		8822	1	6.345	86.4	6
	K.YNVVSL	3.0955	0.3634	100	1357.684	1357.683		6306.8	1	6.738	75	3
	R.NIYNDT	4.4441	0.4249	100	1907.946	1907.944		9880.9	1	8.375	67.9	8
	R.PFHAIL	4.997	0.4254	100	1638.873	1638.868		8611.9	1	7.667	84.6	1
PFA0430c		36	4.60%	1152	135621	5.3	U		Annotation|Plasmodium_falciparum_Sanger|(protein coc			
	R.KHDKED	4.2867	0.2909	100	1555.824	1555.823		4337.5	1	6.225	56.8	4
	R.KHDKED	5.0763	0.3453	100	1557.831	1555.823		4851.8	1	6.598	86.4	19
	K.VLSNYE	4.5284	0.3529	100	3190.688	3188.669		9158.6	1	7.107	40.7	6
	K.KKDESF	5.2811	0.3347	100	1658.861	1658.862		7285.8	1	6.336	83.3	6
	K.KKDESF	4.0587	0.233	99.9	1658.861	1658.862		6424.2	1	4.646	54.2	1
PFE0885w		81	4.60%	716	84057	8.1	U		Annotation|Plasmodium_falciparum_Sanger|(protein coc			
	R.YDSHFE	4.9003	0.3485	100	1949.89	1948.887		6316	1	6.309	71.4	63
	R.FLVSAV	5.0808	0.5724	100	1971.976	1970.972		6692.3	1	9.44	67.6	18
PFC0405c		2	4.60%	608	71464	9.9	U		Annotation|Plasmodium_falciparum_Sanger|(protein coc			
	K.KLLLLK	4.1699	0.3618	99.9	3288.026	3281.964		7920.5	14	4.933	22.2	2
PF14_0087		3	4.60%	566	67203	5.2	U		Annotation|Plasmodium_falciparum_TIGR|(protein codin			
	K.IIDDHQS	3.2834	0.3708	100	1167.613	1167.612		5561.8	1	7.221	94.4	2
	K.YGDIYI	4.6326	0.4796	100	1942.02	1942.015		5284.6	1	6.989	63.3	1
PF13_0234		3	4.60%	583	66209	6.4	U		Annotation|Plasmodium_falciparum_Sanger|(protein coc			
	K.TPIFNF	3.6683	0.3351	100	3139.701	3138.7		6710.3	1	6.126	34.6	3
MAL13P1.35		15	4.60%	453	52510	9.2	U		Annotation|Plasmodium_falciparum_Sanger|(protein coc			
	K.ILFVEN	3.8193	0.3566	100	2454.244	2454.256		7713.3	1	5.669	36.2	2
	K.ILFVEN	5.235	0.5472	100	2461.26	2461.273		8033.3	1	8.907	57.5	13

PFF0615c		4	4.60%	347	39434	8.4	U	Annotation|Plasmodium_falciparum_Sanger|(protein coc				
	K.LTNIIM	4.3116	0.4396	100	2001.939	2001.949		6698.7	1	7.37	63.3	4
PFD1105w		1	4.60%	217	24638	4.6	U	Annotation|Plasmodium_falciparum_Sanger|(protein coc				
	K.ININNLK	2.8343	0.2773	99.8	1187.784	1187.778		6487	1	5.342	66.7	1
PF08_0063		17	4.50%	1070	122508	8.5	U	Annotation|Plasmodium_falciparum_Sanger|(protein coc				
	K.YDSAYV	4.6598	0.3781	100	2869.388	2868.398		10167.3	1	6.791	40	12
	K.EFNDEY	4.436	0.4169	100	2622.348	2622.356		9117.2	1	7.624	45.2	5
PFF0690c		10	4.50%	853	96980	7.8	U	Annotation|Plasmodium_falciparum_Sanger|(protein coc				
	K.SKQDDF	2.8093	0.3522	99.9	1516.707	1516.703		5527.2	1	6.642	70.8	3
	K.TNTSYE	3.1208	0.4406	100	1303.629	1303.628		5876.4	1	7.095	70	3
	R.HSGNNS	6.2579	0.4718	100	1596.749	1596.748		4931.2	1	9.237	84.6	4
PF11_0225		5	4.50%	815	95375	7	U	Annotation|Plasmodium_falciparum_TIGR|(protein codin				
	K.NGVDYF	4.2453	0.5421	100	2043.868	2043.865		8817.3	1	9.304	71.9	3
	K.FNIPTN	3.4949	0.3599	100	2287.269	2287.264		6794.5	1	5.933	42.1	2
PF11_0344		9	4.50%	622	72042	5.5	U	Annotation|Plasmodium_falciparum_TIGR|(protein codin				
	K.YDIEEV	4.0671	0.4465	100	1388.703	1388.7		7182.3	1	7.271	90.9	6
	R.HAGNMI	3.8151	0.294	99.9	1817.819	1817.824		6469.3	1	6.75	63.3	3
PFE1355c		9	4.50%	605	70733	6.1	U	Annotation|Plasmodium_falciparum_Sanger|(protein coc				
	K.DILFDS	4.7179	0.5025	100	1958.98	1958.973		10420.3	1	8.601	73.3	7
	K.HVQEHT	3.662	0.3491	100	1354.697	1354.694		6999.1	1	6.687	80	2
PFC0530w		2	4.50%	579	67769	8.7	U	Annotation|Plasmodium_falciparum_Sanger|(protein coc				
	K.INYINS	3.9669	0.3422	100	3036.572	3036.57		4867.6	1	6.792	34	2
PFL1755w		9	4.50%	425	50761	6	U	Annotation|Plasmodium_falciparum_Sanger|(protein coc				
	K.NFLNLY	4.5013	0.4213	100	2348.212	2346.202		5146	1	6.437	52.8	9
PF10_0232		67	4.40%	3328	381256	6.3	U	Annotation|Plasmodium_falciparum_TIGR|(protein codin				
	K.LIGPYL	3.925	0.3212	100	2562.411	2559.405		4716.2	1	5.681	47.6	7
	K.SWLPQ	3.1484	0.3891	100	2060.006	2057.998		7087.7	1	5.562	44.1	6
	R.ANALDL	2.9203	0.3451	100	1060.521	1060.521		3604.5	1	5.995	87.5	3
	K.LFEALHI	3.5728	0.3063	100	1231.684	1231.684		6164.1	1	5.817	88.9	16
	K.KHANVD	4.3751	0.427	100	1700.868	1699.862		6629.8	1	7.816	71.4	3
	K.HANVDV	2.7134	0.3342	99.9	1227.603	1227.597		5053.5	2	6.453	65	3
	K.HANVDV	4.888	0.3884	100	1574.782	1571.767		7149.5	1	8.713	84.6	12
	K.LNNEY	3.7413	0.4792	100	2989.196	2988.193		7915.5	1	8.289	37.5	3
	K.FKDNEY	5.416	0.5398	100	3769.409	3767.403		5964.6	1	9.285	30.8	3
	K.FKDNEY	5.7633	0.5307	100	3776.43	3774.42		8447.7	1	9.678	35	5
	K.DNEYN	4.7128	0.4692	100	3501.252	3499.256		6440.1	1	8.872	35.7	3

	K.NLNINH	5.788	0.5363	100	2199.027	2199.026		6319.7	1	9.962	59.4	3
PF14_0246		36	4.40%	1148	133960	7.8	U	Annotation|Plasmodium_falciparum_TIGR|(protein codin				
	R.VVPLLE	4.7035	0.5067	100	2585.545	2585.543		4877.2	1	9.128	52.3	25
	V.PLLETI	5.3217	0.4489	100	2388.432	2387.406		6411.1	1	9.129	47.5	9
	K.FHPLIE	5.4129	0.3634	100	3320.622	3319.606		5339.7	1	6.264	36.5	2
PFF1350c		11	4.40%	997	113881	7.8	U	Annotation|Plasmodium_falciparum_Sanger|(protein coc				
	K.STHSH	5.0902	0.5441	100	2860.221	2860.216		5698.7	1	8.892	45.7	3
	R.QVIGPI	4.3001	0.4353	100	2167.254	2166.251		7674.9	1	7.686	50	8
PFL0335c		14	4.40%	565	64544	5.8	U	Annotation|Plasmodium_falciparum_Sanger|(protein coc				
	K.KKKEEE	3.829	0.1776	99.9	1521.837	1520.832		7395.7	1	4.318	86.4	1
	K.REHQSI	4.5546	0.2947	100	1680.773	1680.773		5640.4	1	8.736	79.2	8
	K.REHQSI	3.8408	0.4064	100	1687.789	1687.79		8721.7	1	6.679	47.9	1
	R.EHQSIF	3.4735	0.4063	100	1531.692	1531.689		6160	1	7.142	68.2	4
MAL13P1.79		3	4.40%	383	44241	7.9	U	Annotation|Plasmodium_falciparum_Sanger|(protein coc				
	K.ALYTFS	3.0697	0.3466	99.9	2007.022	2007.027		4690.3	2	6.351	46.9	3
PF11_0087		2	4.40%	366	40612	7.3	U	Annotation|Plasmodium_falciparum_TIGR|(protein codin				
	R.YGLHPT	4.2573	0.4397	100	1856.913	1856.908		6214	1	7.803	66.7	2
PF14_0186		6	4.30%	585	69979	9.3	U	Annotation|Plasmodium_falciparum_TIGR|(protein codin				
	K.HVYDKN	3.7326	0.4577	100	1649.809	1649.804		4700.1	1	7.477	75	3
	K.KINFYF	3.7829	0.3256	100	1547.826	1547.834		10058.8	1	5.996	68.2	3
PFL0285w		18	4.30%	322	38414	8.6	U	Annotation|Plasmodium_falciparum_Sanger|(protein coc				
	K.NLLSFY	3.9557	0.2813	100	1649.835	1649.829		7768.9	1	6.697	73.1	18
PF10_0039		2	4.30%	281	32654	7.4	U	Annotation|Plasmodium_falciparum_TIGR|(protein codin				
	R.FKQIED:	3.6158	0.2239	99.9	1478.78	1478.774		8735.9	1	4.909	81.8	2
MAL13P1.167		1	4.30%	184	21125	7	U	Annotation|Plasmodium_falciparum_Sanger|(protein coc				
	R.ILSLHTS	2.5923	0.2732	99.8	905.5548	905.5533		3182.7	1	4.857	92.9	1
PF14_0679		9	4.20%	664	75468	8.4	U	Annotation|Plasmodium_falciparum_TIGR|(protein codin				
	K.VEDATV	4.3648	0.4207	100	2169.017	2168.015		7922.3	1	7.031	52.9	3
	K.WGWGF	2.8623	0.357	100	1193.576	1193.574		5747.9	1	6.179	77.8	6
PF10_0351		4	4.20%	566	65151	3.9	U	Annotation|Plasmodium_falciparum_TIGR|(protein codin				
	K.VPEEVA	5.4541	0.3794	100	2741.371	2740.367		9353.4	1	7.453	41.3	4
PF14_0137		8	4.20%	381	45239	5.8	U	Annotation|Plasmodium_falciparum_TIGR|(protein codin				
	K.DDIFYSI	4.1391	0.3883	100	1995.038	1995.038		5885.3	1	7.013	63.3	8
PF11_0052		2	4.20%	336	40628	8.2	U	Annotation|Plasmodium_falciparum_TIGR|(protein codin				
	F.LILILVII	1.3879	0.6594	99.9	1640.19	1639.154		4194.1	1	9.771	30.8	2
PFB0840w		5	4.20%	330	37923	6.8	U	Annotation|Plasmodium_falciparum_TIGR|(protein codin				

	K.TSAINA	3.8636	0.3739	100	1478.808	1478.808		7068.9	1	8.174	61.5	5	
PF13_0044		24	4.10%	2375	273437	6.6	U		Annotation|Plasmodium_falciparum_Sanger|(protein coc				
	K.NVINHTI	2.5855	0.3276	99.9	1086.65	1086.65		5512.2	1	5.859	81.2	1	
	K.MKNEEF	4.0918	0.2977	100	1634.871	1634.869		7503.1	1	5.447	87.5	6	
	R.GVNQPY	5.3031	0.3813	100	3054.55	3054.544		4667	1	5.834	31	1	
	R.FYSVQF	3.8595	0.3221	100	2561.182	2560.179		6997.8	1	6.556	45.2	12	
	R.AGSHHI	3.5926	0.3097	100	1362.692	1362.688		8253.1	1	6.014	68.2	2	
	K.KENNYN	4.315	0.4435	100	1838.853	1838.846		7550.8	1	7.59	67.9	2	
MAL7P1.12		16	4.10%	2283	263178	8.6	U		Annotation|Plasmodium_falciparum_Sanger|(protein coc				
	N.NNIILIVI	3.2572	0.5604	100	2340.645	2339.639		8406.4	1	8.075	35	2	
	K.LYQASA	3.1177	0.2779	99.9	1443.674	1440.687		4614.9	1	4.799	72.7	3	
	K.THVACA	3.0034	0.3945	100	1551.793	1551.793		6812	1	6.603	83.3	3	
	K.FNLYEIE	4.1468	0.3295	100	1706.002	1705.997		9679.8	1	5.692	65.4	1	
	K.KAVQDA	4.5755	0.4808	100	1608.768	1608.777		8719.8	1	10.022	84.6	6	
	K.DGAADC	4.0278	0.2529	99.9	2181.019	2180.011		9410	1	5.667	52.8	1	
PFD0795w		9	4.10%	1267	147718	6.7	U		Annotation|Plasmodium_falciparum_Sanger|(protein coc				
	K.LSAVNT	4.0477	0.3899	100	2388.192	2388.217		5995	1	7.121	42.5	5	
	K.LISENY	3.0659	0.4066	100	1152.587	1152.588		4729.7	2	6.922	87.5	1	
	K.NNQSDV	4.2612	0.2508	100	2498.234	2497.228		4504	1	6.566	52.4	3	
PF11_0156		5	4.10%	751	87676	8.3	U		Annotation|Plasmodium_falciparum_TIGR|(protein codin				
	K.SKGNAD	4.3582	0.3639	100	1641.834	1641.831		5014.1	1	7.137	71.4	3	
	R.IMHADL	3.77	0.3997	100	1864	1864		5934.5	1	7.039	73.3	2	
PF14_0096		4	4.10%	514	59275	7.5	U		Annotation|Plasmodium_falciparum_TIGR|(protein codin				
	K.EEGRPY	5.9146	0.4369	100	2625.246	2625.256		9654.8	1	8.03	42.5	3	
	K.EEGRPY	4.106	0.3771	100	2626.239	2625.256		3944.5	1	6.836	42.5	1	
PFL0615w		1	4.10%	340	40106	9	U		Annotation|Plasmodium_falciparum_Sanger|(protein coc				
	K.VINNIH	4.0846	0.3331	100	1713.888	1713.886		4603	1	6.717	76.9	1	
PFC1050w		1	4.10%	315	37461	7.5	U		Annotation|Plasmodium_falciparum_Sanger|(protein coc				
	R.SGEVAF	3.2521	0.3538	100	1445.759	1445.758		5005.1	1	5.89	75	1	
PF11_0482		21	4.00%	1361	158897	7.6	U		Annotation|Plasmodium_falciparum_TIGR|(protein codin				
	K.NIIEIGT	5.4389	0.6005	100	2356.437	2356.43		7861.3	1	10.303	54.5	9	
	K.HVDALE	4.701	0.4028	100	1757.859	1756.854		9556.1	1	7.875	73.3	3	
	K.TYTNLL	4.3267	0.292	100	1999.996	1998.993		7441.7	1	6.145	56.7	9	
PF14_0346		6	4.00%	853	97694	5.6	U		Annotation|Plasmodium_falciparum_TIGR|(protein codin				
	R.AYTLVG	3.6185	0.3654	100	2061.093	2060.089		5850.5	1	5.575	47.2	2	
	K.DIKDHP	4.2809	0.4491	100	1916.905	1916.905		6518.3	1	6.803	71.4	1	

	K.DHPFFS	3.2032	0.2772	99.9	1553.684	1553.681		4540.3	1	6.525	77.3	3
PF13_0149		1	4.00%	582	66512	5.6	U		Annotation|Plasmodium_falciparum_Sanger|(protein coc			
	P.FSPHKT	3.3425	0.2894	99.9	2654.338	2654.337		5327	1	5.696	38.6	1
PF14_0586		1	4.00%	323	36939	6.8	U		Annotation|Plasmodium_falciparum_TIGR|(protein codin			
	K.RNGTG(	3.3131	0.358	100	1424.756	1424.755		4946.8	1	6.256	79.2	1
PF14_0128		13	4.00%	299	34417	7.5	U		Annotation|Plasmodium_falciparum_TIGR|(protein codin			
	K.EILNVLY	4.054	0.2468	100	1482.878	1482.868		8865.5	1	5.648	77.3	13
PFF0105w		6	4.00%	252	29441	5.8	U		Annotation|Plasmodium_falciparum_Sanger|(protein coc			
	K.FQNTLG	3.1491	0.2993	99.9	1232.609	1232.606		5957.2	1	5.859	94.4	6
PF14_0146		1	4.00%	201	22332	10.2	U		Annotation|Plasmodium_falciparum_TIGR|(protein codin			
	R.VVGLILI	3.0683	0.3637	100	896.6491	896.6484		3241.2	1	7.241	92.9	1
PF11_0168		35	3.90%	2966	344922	7	U		Annotation|Plasmodium_falciparum_TIGR|(protein codin			
	K.HTEPLT	3.728	0.4625	100	1624.795	1624.793		4316.5	1	8.297	61.5	6
	K.NTEENK	6.9148	0.4445	100	2842.349	2841.345		6105.4	1	8.423	39.8	13
	K.NTEENK	5.4898	0.4217	100	2843.354	2841.345		4747.1	1	7.667	43.2	3
	K.NVPHLD	3.4948	0.3573	100	1569.737	1569.733		3521	1	6.314	69.2	3
	K.HINEHT	4.8583	0.377	100	2576.178	2575.186		6642.1	1	6.757	36.9	3
	K.YYQNMI	5.498	0.5017	100	3519.723	3517.714		7629.5	1	7.431	26.2	2
	K.KPSSSII	3.5323	0.4532	100	1534.958	1534.947		4365.7	1	7.713	64.3	1
	K.KPSSSII	5.3116	0.4729	100	1536.957	1534.947		6575.6	1	8.681	85.7	4
PF11_0212		4	3.90%	791	94405	9	U		Annotation|Plasmodium_falciparum_TIGR|(protein codin			
	N.AKDIEN'	3.3142	0.3282	100	1323.696	1323.702		4814.1	1	6.119	80	2
	K.NHIISTP	4.183	0.3876	100	2276.247	2276.248		6112.5	1	6.735	50	2
PFF0785w		2	3.90%	591	69875	5.6	U		Annotation|Plasmodium_falciparum_Sanger|(protein coc			
	K.NKTENII	3.7193	0.2921	100	1458.832	1458.835		7138.8	1	5.86	77.3	1
	K.TFNQIV	3.5447	0.2589	100	1299.668	1299.666		8840.4	1	5.399	80	1
PF14_0641		4	3.90%	488	55757	8.6	U		Annotation|Plasmodium_falciparum_TIGR|(protein codin			
	K.YFDISS	3.5417	0.3543	100	2205.112	2205.108		5795.1	1	6.567	61.1	4
PF11_0336		3	3.90%	386	46656	9.5	U		Annotation|Plasmodium_falciparum_TIGR|(protein codin			
	K.NFGSNF	4.371	0.4147	100	1756.967	1756.965		6828.9	1	6.286	85.7	3
PF14_0447		13	3.90%	383	44699	8.6	U		Annotation|Plasmodium_falciparum_TIGR|(protein codin			
	K.VLNTYD	4.3178	0.4148	100	1720.91	1720.902		5071	1	7.989	75	13
PF10_0126		6	3.90%	380	43520	9.2	U		Annotation|Plasmodium_falciparum_TIGR|(protein codin			
	I.RLFNTF	3.4958	0.3633	100	1842.951	1842.95		5603.1	1	6.029	67.9	6
PFC0595c		2	3.90%	308	35765	5.3	U		Annotation|Plasmodium_falciparum_Sanger|(protein coc			
	R.GAGWLI	3.2319	0.2949	99.9	1246.659	1246.658		5063	6	5.727	63.6	2

PF10_0143		70	3.80%	2578	300253	8.6	U		Annotation|Plasmodium_falciparum_TIGR|(protein codin			
	K.FGFGNV	5.8213	0.5168	100	2309.154	2309.157		8127.2	1	9.478	62.5	19
	K.FGFGNV	5.8277	0.3295	100	2317.186	2316.174		7552.1	1	6.503	48.8	31
	R.LLIKPD	5.1771	0.3887	100	2094.136	2093.128		6484.3	1	7.948	52.9	5
	R.LLIKPD	3.6694	0.362	100	2095.14	2093.128		7975.3	3	6.581	41.2	2
	R.TQIIGY	3.4263	0.3629	100	1260.732	1260.729		6947	1	6.841	83.3	8
	K.NDNKDI	6.3129	0.3078	100	3846.615	3845.606		7336.9	1	5.996	27.4	2
	K.INENEC	5.255	0.5091	100	1943.884	1943.88		8333.1	1	9.292	73.3	3
PF07_0017		4	3.80%	1010	118838	6.7	U		Annotation|Plasmodium_falciparum_Sanger|(protein coc			
	R.YSEFEE	3.2027	0.3173	99.9	2822.401	2820.392		6478.7	1	5.23	34.8	1
	K.KHEYDV	4.6438	0.4398	100	1762.83	1762.829		5733.9	1	8.078	88.5	3
PF14_0389		3	3.80%	556	65861	8.9	U		Annotation|Plasmodium_falciparum_TIGR|(protein codin			
	K.RPVVIN	3.5778	0.2731	99.8	2293.252	2292.25		4889.1	1	5.603	37.5	3
PF10_0123		11	3.80%	555	63932	6.4	U		Annotation|Plasmodium_falciparum_TIGR|(protein codin			
	K.GVILSG	4.8502	0.5212	100	1969.041	1969.039		7784.7	1	8.794	60.5	8
	K.GVILSG	4.4409	0.381	100	2104.167	2104.152		7663.2	1	6.84	47.5	3
PFC0190c		4	3.80%	533	62303	8	U		Annotation|Plasmodium_falciparum_Sanger|(protein coc			
	R.TEEETV	3.7445	0.3282	100	2366.158	2364.15		4509.6	1	5.715	50	4
PF11_0251		12	3.80%	421	49615	6.5	U		Annotation|Plasmodium_falciparum_TIGR|(protein codin			
	R.LLDDLL	4.1078	0.393	100	1941.913	1940.906		8544.3	1	7.184	63.3	12
PFL1870c		3	3.80%	393	46013	9	U		Annotation|Plasmodium_falciparum_Sanger|(protein coc			
	K.RHDNYN	3.4433	0.4975	100	1695.732	1695.732		5462.5	1	7.944	60.7	3
PF11_0114		24	3.80%	392	44324	5.9	U		Annotation|Plasmodium_falciparum_TIGR|(protein codin			
	R.FYVAEA	4.5277	0.4116	100	1865.921	1864.917		5929.7	1	8.168	75	24
PFE0410w		2	3.80%	342	39040	9.4	U		Annotation|Plasmodium_falciparum_Sanger|(protein coc			
	R.VNQITH	3.4112	0.2588	99.9	1408.793	1408.791		4756.8	1	5.272	79.2	2
PF14_0228		23	3.70%	1558	183575	6.9	U		Annotation|Plasmodium_falciparum_TIGR|(protein codin			
	K.VHNNSA	3.9179	0.4196	100	2122.953	2121.949		6801	1	7.311	50	1
	K.VHNNSA	6.6829	0.4832	100	2122.958	2121.949		6397.8	1	8.078	67.6	12
	R.NQFFNL	4.5106	0.382	100	1990.964	1990.963		7755.8	1	7.292	60	6
	K.DDHHVI	3.9963	0.3097	100	1413.678	1413.684		5681.4	1	6.575	77.3	3
	K.KKDETN	3.3438	0.2518	99.9	1388.743	1388.75		5761.8	1	5.549	75	1
PF08_0132		16	3.70%	1397	160425	6.7	U		Annotation|Plasmodium_falciparum_Sanger|(protein coc			
	R.SVHSVF	3.7697	0.3663	100	2072.025	2072.02		5136.9	1	7.592	62.5	3
	R.SVHSVF	3.8826	0.4314	100	2073.023	2072.02		7341.6	1	6.506	42.2	6
	R.IVSNSS	3.1782	0.3562	100	1571.759	1571.756		5271.4	1	6.56	64.3	1

	K.TVNKN	4.5999	0.4407	100	2203.997	2203.983		4724.4	1	7.876	47.2	3
	K.NDTQDN	3.5987	0.4153	100	1762.741	1761.729		4460.6	1	7.625	64.3	3
PF08_0035		33	3.70%	1176	138241	5.4	U		Annotation|Plasmodium_falciparum_Sanger|(protein coc			
	K.MQHYEI	4.3376	0.3905	100	1882.824	1882.822		5550.7	1	7.733	82.1	4
	R.NYNTMV	4.5463	0.3685	100	1837.799	1837.796		5307.7	1	6.622	67.9	4
	K.NNLFHF	4.2721	0.3535	100	1710.871	1709.865		6776.1	1	6.658	84.6	25
PFD0090c		30	3.70%	428	49743	9.5	U		Annotation|Plasmodium_falciparum_Sanger|(protein coc			
	K.VHENDY	4.4637	0.5084	100	2031.97	2031.97		5554.3	1	8.22	73.3	30
PF13_0345		3	3.70%	406	46903	8.6	U		Annotation|Plasmodium_falciparum_Sanger|(protein coc			
	R.TNCSLF	3.7647	0.3042	100	1897.96	1897.96		7018.9	5	5.89	50	3
PF10_0098		3	3.70%	298	35603	6.9	U		Annotation|Plasmodium_falciparum_TIGR|(protein codin			
	R.NNNHNY	3.3535	0.3394	100	1428.637	1428.63		4019.9	1	6.535	75	3
PFF1020c		15	3.60%	1356	159575	7.1	U		Annotation|Plasmodium_falciparum_Sanger|(protein coc			
	R.NVPIYI	3.2754	0.3722	100	1581.883	1580.88		4435	1	6.303	75	3
	K.SVIHNI	3.9935	0.352	100	1805.008	1805.008		7095.8	1	6.946	56.7	9
	K.REHEAN	2.9549	0.3901	100	2151.984	2150.981		4872.7	1	6.233	44.1	3
PFI0290c		19	3.60%	1010	118245	5.6	U		Annotation|Plasmodium_falciparum_Sanger|(protein coc			
	R.NINHPQ	3.5136	0.2935	100	1336.7	1336.684		6474	1	5.217	80	3
	K.TLIDLNI	5.808	0.478	100	2991.717	2991.711		6769.9	1	7.189	33.3	12
	K.EIGYDKY	3.6291	0.2667	99.9	1568.824	1568.821		8860.7	4	5.873	66.7	4
PF10_0244		25	3.60%	923	109684	6.7	U		Annotation|Plasmodium_falciparum_TIGR|(protein codin			
	R.IINIPL	4.5588	0.3594	100	2138.222	2136.21		5836.9	1	6.131	50	19
	K.KKEHLG	4.4336	0.3216	100	1727.814	1727.809		6509.1	1	6.135	69.2	6
PF10_0218		9	3.60%	563	65041	9	U		Annotation|Plasmodium_falciparum_TIGR|(protein codin			
	K.NPYPNV	4.1505	0.3861	100	2252.07	2251.068		5589.4	1	6.387	44.7	9
PF14_0536		4	3.60%	415	50459	9.1	U		Annotation|Plasmodium_falciparum_TIGR|(protein codin			
	K.NNFFSH	4.3318	0.3527	100	1837.99	1837.986		5363.4	1	6.742	67.9	4
PF14_0151		1	3.60%	337	37504	9.4	U		Annotation|Plasmodium_falciparum_TIGR|(protein codin			
	K.IKEVAN	3.0147	0.299	99.9	1426.76	1426.752		5561.9	45	5.04	54.5	1
PF14_0614		5	3.50%	1502	177743	5.7	U		Annotation|Plasmodium_falciparum_TIGR|(protein codin			
	K.SLNKPV	3.1304	0.3114	99.9	1841.956	1841.924		4599.3	1	6.95	56.2	3
	K.DSSFNV	3.3326	0.2755	99.9	1902.004	1902.023		8231.5	1	5.808	53.1	1
	K.NLIELI	3.5766	0.3637	100	2277.223	2277.221		5820.8	1	6.607	47.2	1
PF08_0026		3	3.50%	630	75292	5.3	U		Annotation|Plasmodium_falciparum_Sanger|(protein coc			
	K.LHHNV	4.8765	0.4405	100	2652.159	2652.154		8412.5	1	7.535	36.9	3
PFI1525w		3	3.50%	288	34573	6.3	U		Annotation|Plasmodium_falciparum_Sanger|(protein coc			

	K.RKEQLE	3.3289	0.2667	99.9	1379.687	1379.692	6821.5	1	5.874	88.9	3
PFF0895w		6	3.50%	313	34041	7.4	U	Annotation|Plasmodium_falciparum_Sanger|(protein coc			
	K.GAHPVE	3.1752	0.3059	99.9	1195.642	1195.648	3976.4	1	6.171	75	6
PF14_0637		12	3.40%	1455	174305	4.9	U	Annotation|Plasmodium_falciparum_TIGR|(protein codin			
	K.DSLLNE	3.6669	0.2841	99.9	1911.95	1910.946	5522.3	1	5.065	56.7	3
	K.ITNKEC	4.8038	0.4011	100	2112.049	2111.044	6062.4	1	7.241	59.4	2
	K.SLLDEL	4.4835	0.1778	99.9	1733.81	1732.803	11361.4	2	4.632	73.1	1
	K.SLLDEL	4.2642	0.3116	100	1990.948	1989.941	9320	1	5.605	70	6
PF11_0189		35	3.40%	1488	173625	5.4	U	Annotation|Plasmodium_falciparum_TIGR|(protein codin			
	K.LNLLKD	3.8955	0.2014	99.9	1459.847	1459.852	8399.6	1	5.206	81.8	4
	K.NLNLNF	3.7189	0.2589	99.8	1937.998	1937.997	6519.1	1	5.785	60	6
	K.NDIINF	4.2617	0.4445	100	2684.336	2684.333	5427	1	7.46	47.6	25
PF14_0094		9	3.40%	768	92748	7.4	U	Annotation|Plasmodium_falciparum_TIGR|(protein codin			
	R.NSFLDT	4.5399	0.4314	100	1862.903	1862.904	7481.5	1	7.901	71.4	7
	K.NASVIVI	2.9922	0.3619	100	1267.706	1267.705	6756.7	1	6.228	85	2
PFE0375w		7	3.40%	652	73424	5.3	U	Annotation|Plasmodium_falciparum_Sanger|(protein coc			
	R.FYAVST	4.0096	0.3053	100	2398.192	2398.208	9362.7	1	6.173	45.2	7
PFE0255w		29	3.40%	565	65868	5.3	U	Annotation|Plasmodium_falciparum_Sanger|(protein coc			
	K.IYNIGG	5.1321	0.446	100	2193.165	2193.174	7607.2	1	8.587	58.3	29
PF13_0190		63	3.40%	524	62674	8.2	U	Annotation|Plasmodium_falciparum_Sanger|(protein coc			
	K.LKIPDNL	3.577	0.3855	100	2217.218	2217.211	6468.3	1	6.433	50	4
	K.IPDNLFN	5.282	0.4617	100	1977.036	1976.032	7888.7	1	8.559	76.7	51
	I.PDNLFNI	5.6435	0.537	100	1855.933	1855.93	8772.9	1	9.755	85.7	8
PFD1100c		3	3.40%	476	58351	9.8	U	Annotation|Plasmodium_falciparum_Sanger|(protein coc			
	K.NHDTHH	4.5872	0.5648	100	1947.795	1947.796	3746.4	1	9.638	70	3
PF14_0125		3	3.40%	496	57242	6.1	U	Annotation|Plasmodium_falciparum_TIGR|(protein codin			
	K.NDQNQ	3.9516	0.3281	100	2070.934	2069.928	4678.1	2	6.704	50	3
PF11_0436		12	3.40%	470	56343	9.2	U	Annotation|Plasmodium_falciparum_TIGR|(protein codin			
	R.GIGGIFF	4.2001	0.3628	100	1776.046	1776.043	6521.6	1	6.756	53.3	12
PFF0825c		9	3.40%	377	43624	8.1	U	Annotation|Plasmodium_falciparum_Sanger|(protein coc			
	K.SPEFML	3.8542	0.4495	100	1607.79	1607.789	9349.6	1	8.032	79.2	9
PFD0265w		49	3.30%	3136	366398	8.7	U	Annotation|Plasmodium_falciparum_Sanger|(protein coc			
	R.LGNQLF	3.4617	0.2992	99.9	2602.212	2601.23	8146.8	1	5.788	45	3
	K.DGPYVS	4.7751	0.4942	100	2707.3	2705.296	6763.6	1	8.621	45.7	26
	R.NLFPNW	3.2029	0.3569	100	2499.306	2498.308	5271.9	4	6.131	35	3
	R.SLTSLD	3.3736	0.4588	100	2262.077	2262.072	5684	1	7.206	47.2	8

	R.TDMIQA	4.7945	0.4433	100	2189.181	2186.172		6571.2	1	8.565	55.3	9
PFA0410w		12	3.30%	2221	255042	5.7	U		Annotation|Plasmodium_falciparum_Sanger|(protein coc			
	K.IPMEEL	5.851	0.378	100	2277.139	2276.136		7518.3	1	7.183	63.9	5
	K.VSIYEL	3.431	0.2573	99.9	1580.876	1580.869		7475.5	1	6.238	65.4	2
	K.SGHNII	4.9139	0.496	100	2138.076	2138.073		5701.3	1	9.313	69.4	3
	K.NNNNNN	4.307	0.1653	99.8	2431.082	2431.074		4232.4	1	5.652	55	2
PF14_0461		13	3.30%	1641	191026	5.2	U		Annotation|Plasmodium_falciparum_TIGR|(protein codin			
	R.NNLNYI	3.8467	0.2224	99.9	1736.813	1735.807		7375.1	4	5.443	61.5	3
	K.SNNNQI	3.879	0.2515	100	1450.773	1450.77		5386.9	2	5.028	68.2	6
	R.NFNHNI	4.4731	0.434	100	2841.332	2840.324		8920.1	1	6.863	28.1	2
	R.NFNHNI	4.1089	0.4277	100	3212.493	3212.489		7516.6	1	6.704	32.4	2
PF10_0165		26	3.30%	1094	126884	8.6	U		Annotation|Plasmodium_falciparum_TIGR|(protein codin			
	R.IDPDFL	5.4361	0.4712	100	2654.421	2654.424		7352.1	1	8.705	54.8	18
	K.GILPLIV	3.9288	0.3083	100	1585.974	1585.983		9849.7	1	7.274	76.9	7
	L.PLIVEEL	4.168	0.4181	100	1295.777	1295.776		7532.7	1	7.359	90	1
PF14_0177		4	3.30%	971	111496	5.7	U		Annotation|Plasmodium_falciparum_TIGR|(protein codin			
	K.TLEDQL	4.1356	0.4318	100	2575.175	2574.17		9414.3	2	7.022	33.3	3
	K.VFNEHY	3.2508	0.2038	99.8	1219.65	1219.648		5869.8	1	5.743	88.9	1
PF13_0208		8	3.30%	903	106776	7.1	U		Annotation|Plasmodium_falciparum_Sanger|(protein coc			
	K.HISSEN	3.0815	0.3424	99.9	1584.729	1584.725		6774.6	2	5.987	53.8	2
	K.SILIGHS	5.1808	0.4857	100	1775.011	1774.005		9002.4	1	9.268	70	6
MAL13P1.37		3	3.30%	886	105384	6.5	U		Annotation|Plasmodium_falciparum_Sanger|(protein coc			
	K.KLNGLV	2.9903	0.3119	99.9	1145.634	1145.635		6848.8	1	6.735	77.8	2
	N.NNDGHI	4.0248	0.4146	100	2205.968	2206.01		3941.1	1	7.327	61.1	1
PF14_0025		37	3.30%	666	78381	6.3	U		Annotation|Plasmodium_falciparum_TIGR|(protein codin			
	K.MYQNVI	5.136	0.5182	100	2648.369	2648.379		8708.6	1	9.209	54.8	33
	Q.NVLDII	4.0567	0.3295	100	2227.229	2226.217		9681.6	1	6.984	52.8	4
PF14_0640		2	3.30%	579	69046	5.2	U		Annotation|Plasmodium_falciparum_TIGR|(protein codin			
	R.FYENND	3.9249	0.312	100	2287.137	2286.129		6248.4	1	5.25	47.2	2
PF14_0350		3	3.30%	548	64655	8.2	U		Annotation|Plasmodium_falciparum_TIGR|(protein codin			
	R.LLTQLE	4.2282	0.293	100	2205.104	2204.099		4366.6	1	5.214	50	3
PF13_0140		4	3.30%	522	60064	6.7	U		Annotation|Plasmodium_falciparum_Sanger|(protein coc			
	R.NLSVFH	3.6468	0.3145	100	1935.037	1934.018		6371	1	6.128	50	4
PFF0815w		10	3.30%	521	59507	9	U		Annotation|Plasmodium_falciparum_Sanger|(protein coc			
	K.VWNPDI	3.481	0.3639	100	2146.105	2146.101		6521.7	1	6.848	59.4	10
MAL13P1.83		24	3.20%	1232	142997	6	U		Annotation|Plasmodium_falciparum_Sanger|(protein coc			

	K.EANEVN	4.9289	0.5069	100	2666.341	2666.357		7801.4	1	8.673	50	18
	K.DILQLL	3.3391	0.3928	100	2196.192	2196.203		5741.9	1	6.345	52.9	6
PFE0675c		5	3.20%	1113	129199	9.1	U	Annotation|Plasmodium_falciparum_Sanger|(protein coc				
	K.ILQVDSI	4.1909	0.432	100	1966.108	1963.102		7530.8	1	6.582	62.5	2
	K.LTVYIP	3.287	0.3014	99.8	2303.213	2301.21		3955.6	1	5.008	50	3
PF13_0243		7	3.20%	994	118920	5.9	U	Annotation|Plasmodium_falciparum_Sanger|(protein coc				
	K.SNYIHE	3.5471	0.4528	100	1575.711	1575.713		6764.4	1	7.58	72.7	3
	K.YSAYHD	5.1551	0.4719	100	2369.192	2369.168		5982	1	7.939	60.5	4
PFA0145c		65	3.20%	626	73450	8.2	U	Annotation|Plasmodium_falciparum_Sanger|(protein coc				
	K.YADSIM	5.2799	0.4164	100	2489.192	2487.179		9505.4	1	8.014	57.9	65
PF13_0031		1	3.20%	475	57925	9.4	U	Annotation|Plasmodium_falciparum_Sanger|(protein coc				
	K.NQPSAI	3.0054	0.3582	99.9	1652.838	1652.837		6158.8	1	6.4	50	1
PFB0210c		2	3.20%	504	56417	8.6	U	Annotation|Plasmodium_falciparum_TIGR|(protein codin				
	K.KIYETD	4.0297	0.3046	100	1861.959	1861.955		6682.1	1	6.153	60	2
PF10_0067		1	3.20%	349	41780	7.4	U	Annotation|Plasmodium_falciparum_TIGR|(protein codin				
	K.KDNDKD	3.7102	0.1817	99.9	1331.682	1331.681		4638.2	1	4.761	90	1
PFF0485c		4	3.20%	284	33147	8.8	U	Annotation|Plasmodium_falciparum_Sanger|(protein coc				
	K.ICNVWK	3.2001	0.2115	99.9	1187.601	1187.6		6171.8	1	4.981	87.5	4
PFL0130c		30	3.10%	2158	251675	7.7	U	Annotation|Plasmodium_falciparum_Sanger|(protein coc				
	K.NVTFLC	4.1449	0.3704	100	2084.942	2083.936		4985.4	1	7.276	50	3
	L.KEVEQ	6.2029	0.517	100	3596.427	3594.412		6709.9	1	8.615	30.8	3
	K.EVEQE	7.455	0.5812	100	3467.325	3466.317		5284.3	1	9.978	34.5	6
	K.EVEQE	5.7081	0.6266	100	3467.352	3466.317		6508.5	1	10.657	41.4	6
	K.KNTNYD	4.7871	0.3597	100	2118.011	2117.001		4465.8	1	7.448	58.8	3
	K.NTNYDN	4.8166	0.4379	100	1990.916	1988.906		4494.1	1	7.3	59.4	9
MAL8P1.203		22	3.10%	1471	173404	6.5	U	Annotation|Plasmodium_falciparum_Sanger|(protein coc				
	K.KNVDKN	4.6831	0.3311	100	2278.171	2278.187		7798.2	1	5.626	39.5	1
	K.NFVNDK	3.9997	0.3952	100	1719.797	1718.788		8544.2	1	6.6	80.8	3
	K.DLSAHP	3.3476	0.3349	100	1373.673	1373.664		4942.1	1	6.558	75	18
MAL13P1.405		4	3.10%	1397	162829	7.3	U	Annotation|Plasmodium_falciparum_Sanger|(protein coc				
	K.STVNSA	3.0874	0.3618	99.9	2246.031	2246.029		6788.3	1	6.257	37.5	3
	R.DAHIHA	5.9165	0.5173	100	2694.177	2693.172		6655.7	1	8.322	46.6	1
PFL1635w		24	3.10%	1016	122082	7.9	U	Annotation|Plasmodium_falciparum_Sanger|(protein coc				
	K.FKDNIS	3.2058	0.3051	100	1363.708	1363.708		5560.1	1	5.095	90	2
	K.NLINIID	5.0335	0.5524	100	2397.272	2396.261		9796.1	1	10.119	65.8	22
PF10_0310		7	3.10%	904	106689	4.7	U	Annotation|Plasmodium_falciparum_TIGR|(protein codin				

	K.NIDHSI	3.4328	0.2569	99.9	1675.833	1674.827	6950.5	1	5.357	73.1	1
	K.SSIQHG	4.0483	0.2738	100	1560.808	1560.81	7211.2	1	7.248	76.9	6
PFE0370c		12	3.10%	688	77647	6 U		Annotation|Plasmodium_falciparum_Sanger|(protein coc			
	K.YCQLAA	5.0873	0.5083	100	2394.136	2391.127	5048.8	1	8.72	52.5	12
PFB0240w		4	3.10%	457	54351	8.9 U		Annotation|Plasmodium_falciparum_TIGR|(protein codin			
	K.NDPFPS	2.9519	0.3233	99.8	1565.744	1565.746	4594.2	1	5.321	80.8	4
PF08_0034		5	3.00%	1485	173511	6.8 U		Annotation|Plasmodium_falciparum_Sanger|(protein coc			
	K.IYNVVHS	2.8657	0.3551	100	1185.721	1185.718	6787	1	6.17	77.8	1
	R.LANPNL	4.2551	0.3935	100	2142.098	2142.095	6779.7	1	6.242	52.9	1
	K.NTVIGG	3.4603	0.3395	100	1922.977	1921.971	6576.9	1	6.414	50	3
PF14_0159		12	3.00%	938	110711	7.5 U		Annotation|Plasmodium_falciparum_TIGR|(protein codin			
	K.NKLDEIS	4.9624	0.3868	100	1970.105	1970.092	8280.1	1	7.793	78.1	10
	K.HIQAIS	3.0622	0.3814	100	1201.67	1201.669	6302.2	1	7.617	85	2
PF13_0176		10	3.00%	597	69722	9.2 U		Annotation|Plasmodium_falciparum_Sanger|(protein coc			
	K.NFILPH	3.9397	0.3221	100	2026.067	2026.076	5794.9	1	6.295	64.7	10
PFF0995c		4	3.00%	525	61381	5.1 U		Annotation|Plasmodium_falciparum_Sanger|(protein coc			
	K.KKEEDL	4.1602	0.3503	100	1968.096	1967.089	10175.8	1	6.727	70	4
PFB0550w		4	3.00%	427	48064	7 U		Annotation|Plasmodium_falciparum_TIGR|(protein codin			
	K.ISLTDW	3.0836	0.3275	99.9	1550.887	1550.877	7764.1	1	6.467	62.5	4
PF14_0242		1	3.00%	401	47446	6.5 U		Annotation|Plasmodium_falciparum_TIGR|(protein codin			
	N.KEKEMK	3.641	0.2118	99.9	1583.737	1583.763	5287.8	2	4.445	68.2	1
PF07_0057		2	3.00%	403	47352	8 U		Annotation|Plasmodium_falciparum_Sanger|(protein coc			
	K.GYDTIY	3.7022	0.3184	100	1494.729	1494.734	6269.3	1	6.386	72.7	2
PF14_0714		1	3.00%	394	46190	8.7 U		Annotation|Plasmodium_falciparum_TIGR|(protein codin			
	K.NVDEEE	2.6546	0.3253	99.8	1387.7	1387.696	7202.5	1	5.279	63.6	1
PF14_0036		2	3.00%	302	35824	6.1 U		Annotation|Plasmodium_falciparum_TIGR|(protein codin			
	R.GNHGW	2.5233	0.3046	99.8	1147.495	1147.492	5057.4	1	5.371	81.2	2
MAL7P1.229		9	2.90%	1370	162782	8.7 U		Annotation|Plasmodium_falciparum_Sanger|(protein coc			
	K.TTYVHL	3.4058	0.3271	100	2108.152	2107.119	4967.2	1	4.99	55.9	3
	K.HLPNNL	3.2015	0.3252	100	1446.806	1446.807	3376.8	2	5.955	63.6	3
	K.YHNIYN	3.5188	0.3115	100	1306.673	1306.673	5311.2	1	5.617	83.3	3
PFB0715w		44	2.90%	1364	154728	7.7 U		Annotation|Plasmodium_falciparum_TIGR|(protein codin			
	K.RGPTVG	3.0782	0.2485	99.8	900.5016	900.5015	5054.3	1	4.923	93.8	3
	R.IKEDDR	3.5415	0.286	100	1494.77	1494.778	5975.2	1	6.798	81.8	1
	K.WVGCFI	4.6818	0.4486	100	2398.206	2397.21	7594.3	1	8.019	58.3	40
PF10_0278		1	2.90%	483	57057	5.6 U		Annotation|Plasmodium_falciparum_TIGR|(protein codin			

	Q.GSYFEC	3.2156	0.2607	99.8	1734.815	1734.809		9485.3	2	4.581	61.5	1
PFL0865w		2	2.90%	483	56090	6.3	U		Annotation|Plasmodium_falciparum_Sanger|(protein coc			
	K.ENYNIH	3.285	0.3587	100	1686.816	1686.82		4477.1	1	5.306	61.5	2
PF11_0287		4	2.90%	280	33119	8.5	U		Annotation|Plasmodium_falciparum_TIGR|(protein codin			
	K.GYCYIH	2.7954	0.296	99.9	1032.481	1032.48		5422.3	1	7.088	85.7	4
PF10_0031		14	2.80%	944	111602	5.6	U		Annotation|Plasmodium_falciparum_TIGR|(protein codin			
	K.KNICEQ	4.3504	0.2477	100	1549.775	1549.755		5638.2	1	5.444	61.4	3
	K.KNICEQ	4.2936	0.2772	100	1563.791	1563.789		6987.9	1	6.747	81.8	1
	K.NICEQIE	4.1373	0.2658	100	1421.661	1421.66		6529.5	1	5.931	85	4
	R.KKNELY	4.6874	0.5566	100	1864.921	1864.934		8698.2	1	9.908	80.8	6
PFA0570w		2	2.80%	637	75052	9.3	U		Annotation|Plasmodium_falciparum_Sanger|(protein coc			
	K.NENYLC	2.8633	0.3657	99.9	2313.133	2313.13		4853.5	1	5.563	52.9	2
PF13_0253		23	2.80%	573	66040	5.8	U		Annotation|Plasmodium_falciparum_Sanger|(protein coc			
	K.VFINEL	4.0992	0.341	100	1831.93	1831.93		7504.5	1	6.62	41.7	4
	K.VFINEL	5.6273	0.4839	100	1846.971	1845.965		6076.8	1	9.276	73.3	19
PFF0160c		1	2.80%	569	65559	9	U		Annotation|Plasmodium_falciparum_Sanger|(protein coc			
	K.HLDFIN	3.6979	0.3545	100	1756.89	1754.898		7049.6	1	6.842	63.3	1
PF14_0602		9	2.80%	539	64036	6.6	U		Annotation|Plasmodium_falciparum_TIGR|(protein codin			
	K.YYVSNV	3.6412	0.3608	100	1711.912	1710.886		6047.9	1	6.445	71.4	9
PF11_0101		6	2.80%	463	55433	6.2	U		Annotation|Plasmodium_falciparum_TIGR|(protein codin			
	K.HVDHPL	4.0844	0.3895	100	1577.783	1577.779		7542.9	1	7.789	83.3	6
PFB0295w		3	2.80%	471	55176	7	U		Annotation|Plasmodium_falciparum_TIGR|(protein codin			
	K.THGQPA	3.278	0.3922	100	1328.66	1328.66		5748	1	6.811	79.2	3
PFF0220w		63	2.70%	2874	334410	8.4	U		Annotation|Plasmodium_falciparum_Sanger|(protein coc			
	R.RLNDEH	3.7149	0.3398	100	1530.696	1530.694		4980.7	1	6.529	77.3	6
	K.SNTPSH	3.5692	0.212	99.8	1441.654	1441.646		5336.3	1	6.403	70.8	13
	K.NSNNND	3.9221	0.327	100	2335.083	2334.081		4368.2	1	5.793	45.2	3
	R.FNDMFF	4.7838	0.3817	100	2325.147	2322.134		5766.7	1	8.577	52.6	39
	K.LPLDDS	2.8091	0.2969	99.8	1387.792	1387.784		8816.8	1	5.541	77.3	2
PFL1705w		21	2.70%	1662	194673	7.7	U		Annotation|Plasmodium_falciparum_Sanger|(protein coc			
	R.KNNNYE	6.1438	0.4167	100	3284.431	3284.428		8491.8	1	7.49	30.8	1
	R.KNNNYE	7.7323	0.4991	100	3285.434	3284.428		9785.2	1	8.532	40.4	6
	R.KNNNYE	4.155	0.5039	100	3286.445	3284.428		8253	1	8.152	38.5	4
	K.NNNYEK	5.7328	0.3888	100	3157.338	3156.333		5717.5	1	6.72	34	5
	K.NNNYEK	4.6992	0.4393	100	3157.342	3156.333		7793.6	1	8.468	40	3
	K.KKLENN	3.7212	0.3651	100	2130.111	2130.104		4457.2	1	6.173	58.8	2

PF10_0124		3	2.70%	1438	171012	5.2	U	Annotation|Plasmodium_falciparum_TIGR|(protein codin			
	K.NKNEHE	2.9307	0.3065	99.9	1341.653	1341.651	4122.2	1	4.96	70	2
	R.NDLIEL	4.7084	0.3717	100	3440.605	3439.589	8172.5	1	6.175	30.6	1
PFE0870w		7	2.70%	1141	132682	5	U	Annotation|Plasmodium_falciparum_Sanger|(protein coc			
	K.NSLAHN	4.1697	0.4705	100	2055.959	2055.952	6982.2	1	7.764	62.5	3
	K.NSLAHN	3.1313	0.4377	100	2055.959	2055.952	5121.8	1	6.455	34.4	3
	K.RIDVIPT	4.5231	0.4118	100	1703.994	1703.996	7541.6	1	8.014	73.1	1
PFE0090w		4	2.70%	1076	125356	5	U	Annotation|Plasmodium_falciparum_Sanger|(protein coc			
	K.TNTSTE	4.6154	0.4	100	1994.952	1994.953	4782.1	1	7.631	59.4	3
	R.FIGHSG	3.3059	0.348	100	1402.71	1402.709	7283.3	1	5.698	86.4	1
PFC0390w		8	2.70%	955	112248	5.4	U	Annotation|Plasmodium_falciparum_Sanger|(protein coc			
	R.LLCKDD	4.2109	0.299	100	1799.014	1799.01	11031.9	1	5.967	75	2
	K.YLPIHDI	3.4744	0.3697	100	1372.728	1372.727	4060.5	1	6.008	75	6
MAL13P1.22		5	2.70%	912	104506	7.6	U	Annotation|Plasmodium_falciparum_Sanger|(protein coc			
	K.INKPNIF	4.2279	0.366	100	1626.955	1626.952	4148.4	1	5.82	83.3	3
	R.AQIHYIE	3.4684	0.2869	99.9	1457.781	1457.775	6969.1	1	6.097	72.7	2
PF08_0069		4	2.70%	877	99964	5	U	Annotation|Plasmodium_falciparum_Sanger|(protein coc			
	K.LVNNIIE	3.1628	0.1959	99.9	956.5977	956.5967	3202.9	2	4.499	85.7	1
	R.VAANVC	3.912	0.3076	100	1833.952	1832.948	6358.6	1	6.214	63.3	3
PF13_0205		7	2.70%	632	73100	8	U	Annotation|Plasmodium_falciparum_Sanger|(protein coc			
	K.NTEYAG	4.6907	0.5129	100	1976.024	1976.024	7861.3	1	8.514	62.5	7
PFF1500c		1	2.70%	601	69359	8.6	U	Annotation|Plasmodium_falciparum_Sanger|(protein coc			
	K.TLAFLVI	3.3662	0.3028	99.9	1840.13	1840.125	3683.1	1	5.182	60	1
PFB0795w		2	2.70%	551	61770	8.5	U	Annotation|Plasmodium_falciparum_TIGR|(protein codin			
	K.IQSNCH	3.4082	0.3368	99.9	1799.865	1799.86	5122.4	1	6.541	64.3	2
PFE1510c		3	2.70%	524	59789	9.9	U	Annotation|Plasmodium_falciparum_Sanger|(protein coc			
	R.NQPELF	4.0124	0.4688	100	1808.887	1808.882	4480.6	1	7.939	73.1	3
PFE0820c		3	2.70%	335	39798	5.6	U	Annotation|Plasmodium_falciparum_Sanger|(protein coc			
	K.FGNLPP	3.0428	0.3323	100	982.5747	982.5726	3495.6	1	5.211	81.2	3
PFF1095w		6	2.60%	1447	170240	6.5	U	Annotation|Plasmodium_falciparum_Sanger|(protein coc			
	K.NMVCDI	3.7299	0.3891	100	3002.419	3001.416	5774.6	1	6.244	40	4
	K.QINHIS	2.8626	0.3172	99.9	1409.69	1409.689	3149.4	5	5.12	68.2	2
PF11_0275		5	2.60%	1368	163535	6.6	U	Annotation|Plasmodium_falciparum_TIGR|(protein codin			
	K.HHDED	3.9684	0.3115	100	1708.721	1708.716	5396.5	1	5.972	84.6	3
	K.LFYSTT	3.5112	0.3991	100	2541.421	2541.419	5616.1	1	6.072	33.3	2
PFE0835w		6	2.60%	1332	156332	6.8	U	Annotation|Plasmodium_falciparum_Sanger|(protein coc			

	K.NLACNN	3.3187	0.3525	100	2738.283	2737.279		6187.5	3	5.788	29.5	3		
	N.KIINNNN	3.2961	0.2262	99.8	1322.721	1322.718		6319.9	2	5.4	70	2		
	K.IINNNND	4.1742	0.1502	99.9	1343.774	1343.77		5982	3	4.296	75	1		
PF14_0529		1	2.60%	1081	124243	6.9	U		Annotation|Plasmodium_falciparum_TIGR|(protein codin					
	K.EGGNNS	3.2981	0.3351	99.9	3098.505	3097.496		5435.7	1	5.641	33.3	1		
PF10_0341		1	2.60%	993	116719	8.4	U		Annotation|Plasmodium_falciparum_TIGR|(protein codin					
	K.ENHNIS	4.2248	0.3951	100	3033.629	3033.621		6196.9	1	5.983	33	1		
PFC1000w		7	2.60%	899	108575	7.2	U		Annotation|Plasmodium_falciparum_Sanger|(protein coc					
	K.YLNYYF	3.5809	0.375	100	2831.529	2831.529		6623.3	1	6.307	47.7	7		
PFF1055c		1	2.60%	776	92452	8.2	U		Annotation|Plasmodium_falciparum_Sanger|(protein coc					
	R.ASLVYE	3.6547	0.2608	99.9	2319.136	2318.163		4520.2	1	5.35	44.7	1		
PFE0215w		1	2.60%	755	87598	9.2	U		Annotation|Plasmodium_falciparum_Sanger|(protein coc					
	R.ASSFFD	3.4452	0.2487	99.8	2354.193	2354.178		8001.6	2	4.968	39.5	1		
PF14_0482		3	2.60%	620	72758	9.8	U		Annotation|Plasmodium_falciparum_TIGR|(protein codin					
	K.KKENVN	5.2232	0.4845	100	1817.016	1816.012		6418.2	1	8.668	80	3		
PF13_0149		6	2.60%	582	66512	5.6	U		Annotation|Plasmodium_falciparum_Sanger|(protein coc					
	K.GHTNE	4.6459	0.5226	100	1701.763	1700.756		4863.8	1	8.218	78.6	6		
PFD0880w		12	2.60%	429	51030	7.1	U		Annotation|Plasmodium_falciparum_Sanger|(protein coc					
	K.KYEESY	4.74	0.3519	100	1453.725	1453.733		6833.3	1	6.798	90	12		
PFL2390c		13	2.50%	2932	348677	8.5	U		Annotation|Plasmodium_falciparum_Sanger|(protein coc					
	L.YKEDLS	5.2747	0.3328	100	5279.466	5275.426		7887.1	1	5.061	15.2	2		
	K.SSTQIL	3.5423	0.4496	100	3309.752	3309.727		5467.6	1	6.789	29.6	11		
MAL13P1.148		8	2.50%	2160	250288	8.6	U		Annotation|Plasmodium_falciparum_Sanger|(protein coc					
	R.FDIDEIY	4.1065	0.4459	100	2441.372	2441.338		5968.9	1	7.883	47.5	1		
	K.NTYVSH	3.3583	0.4393	100	1642.821	1642.823		6721.7	1	7.345	70.8	6		
	K.ESINPA	3.762	0.3678	100	2089.185	2089.184		5908.7	1	6.384	50	1		
PF13_0277		6	2.50%	2068	248259	8.3	U		Annotation|Plasmodium_falciparum_Sanger|(protein coc					
	K.SCQEE	7.9751	0.5136	100	4500.649	4497.647		9677.4	1	10.639	32.6	3		
	K.GIANVD	4.3292	0.3957	100	1575.811	1575.811		6394.4	1	7.743	76.9	3		
PF14_0607		2	2.50%	1068	126834	8.6	U		Annotation|Plasmodium_falciparum_TIGR|(protein codin					
	K.NNMIQ	3.1316	0.3099	99.9	3284.432	3284.428		4191.5	1	5.49	28.8	2		
PF14_0727		1	2.50%	790	92650	5.4	U		Annotation|Plasmodium_falciparum_TIGR|(protein codin					
	F.YDDYLA	3.5793	0.2681	99.9	2311.127	2308.103		9646.1	1	5.227	44.7	1		
MAL8P1.146		3	2.50%	728	86743	7.5	U		Annotation|Plasmodium_falciparum_Sanger|(protein coc					
	K.GKNENF	3.725	0.3808	100	2108.114	2108.122		8394.2	1	6.529	55.9	1		
	K.NENFQN	3.5617	0.2434	99.8	1908.958	1908.971		8028	3	5.295	53.3	2		

PF13_0192		6	2.50%	633	75558	5.3	U		Annotation|Plasmodium_falciparum_Sanger|(protein coc				
	R.QLFDEA	4.8711	0.4237	100	1915.969	1914.978		8947.9	1	7.471	73.3	6	
PFC0265c		3	2.50%	628	74294	4.9	U		Annotation|Plasmodium_falciparum_Sanger|(protein coc				
	K.SLDNSY	4.3272	0.4673	100	1860.846	1859.841		6444.3	1	7.737	63.3	3	
PF08_0129		2	2.50%	604	70076	5	U		Annotation|Plasmodium_falciparum_Sanger|(protein coc				
	K.EVIQILN	4.2884	0.3438	100	1875.003	1874.998		7533.9	1	5.838	64.3	2	
PF11_0268		3	2.50%	596	68047	5.8	U		Annotation|Plasmodium_falciparum_TIGR|(protein codin				
	R.WKEITN	3.4665	0.3423	100	1720.856	1720.844		5602.1	1	6.818	64.3	3	
PF11_0506		5	2.50%	570	66300	4.9	U		Annotation|Plasmodium_falciparum_TIGR|(protein codin				
	K.IKPTYE	3.6586	0.2069	99.8	1705.836	1702.825		4616.9	1	4.694	73.1	5	
PF14_0215		6	2.50%	518	60706	8.1	U		Annotation|Plasmodium_falciparum_TIGR|(protein codin				
	K.LSCSHI	3.8108	0.2813	100	1622.789	1622.79		6967.1	1	5.701	79.2	6	
PF14_0059		15	2.40%	2820	338381	5.3	U		Annotation|Plasmodium_falciparum_TIGR|(protein codin				
	K.IKGDDD	5.9907	0.3132	100	5065.972	5063.957		9622.8	1	8.904	20.8	5	
	K.IKGDDD	5.5121	0.4524	100	5437.143	5435.137		8289	1	7.414	18.3	2	
	R.KTEDIH	3.4845	0.2448	99.9	1243.648	1243.647		4153	1	5.677	83.3	5	
	K.SYVSNK	3.338	0.3167	99.9	1554.78	1554.776		5357.2	1	5.968	66.7	3	
PF14_0644		39	2.40%	2104	237000	8.8	U		Annotation|Plasmodium_falciparum_TIGR|(protein codin				
	K.FNIASID	5.0293	0.3233	100	1936.979	1935.974		9277.7	1	7.663	75	9	
	K.VVHNVV	8.1001	0.5445	100	3757.952	3756.942		10453	1	10.454	34.8	30	
MAL7P1.129		2	2.40%	1003	124126	8.6	U		Annotation|Plasmodium_falciparum_Sanger|(protein coc				
	K.KLLNIE	4.5565	0.3728	100	1620.883	1620.882		6296.4	1	6.683	75	1	
	K.KLHSND	2.6261	0.3171	99.8	1283.673	1283.671		5994.8	1	6.27	70	1	
PF07_0026		16	2.40%	961	110779	8.2	U		Annotation|Plasmodium_falciparum_Sanger|(protein coc				
	N.NHNNIN	3.5895	0.3442	100	1382.637	1382.635		3508.7	1	5.828	80	7	
	R.TDVNVH	3.818	0.438	100	1453.672	1450.661		5528.3	1	7.237	86.4	9	
PF10_0348		1	2.40%	697	80248	5.1	U		Annotation|Plasmodium_falciparum_TIGR|(protein codin				
	K.LTSKDE	4.684	0.3578	100	2055.057	2055.054		7668	1	8.047	65.6	1	
PF14_0616		2	2.40%	706	79815	8.9	U		Annotation|Plasmodium_falciparum_TIGR|(protein codin				
	K.GCDEVK	3.361	0.3224	99.9	2080.976	2081.011		8039.9	1	6.107	62.5	2	
PF10_0208		2	2.40%	627	73607	9	U		Annotation|Plasmodium_falciparum_TIGR|(protein codin				
	K.GFYYTG	3.7557	0.4332	100	1771.882	1771.881		3582.9	1	7.302	71.4	2	
MAL13P1.344		1	2.40%	619	70270	8.9	U		Annotation|Plasmodium_falciparum_Sanger|(protein coc				
	K.LSPIIKP	3.2898	0.3247	99.9	1696.059	1696.067		7021.1	3	5.612	50	1	
PF11_0397		1	2.40%	500	59200	5.2	U		Annotation|Plasmodium_falciparum_TIGR|(protein codin				
	K.YKDKDF	3.7618	0.234	99.9	1471.79	1471.787		6834.9	2	5.779	72.7	1	

MAL8P1.84		1	2.40%	491	58164	6.4	U		Annotation|Plasmodium_falciparum_Sanger|(protein coc			
	K.NIFDLY	3.4413	0.208	99.8	1560.842	1560.847		7556	2	4.404	68.2	1
PFC0370w		3	2.40%	492	57959	7.9	U		Annotation|Plasmodium_falciparum_Sanger|(protein coc			
	R.KPALYK	3.6539	0.415	100	1461.798	1461.807		6305.5	1	7.618	77.3	3
PFL1040w		3	2.40%	458	54019	6.3	U		Annotation|Plasmodium_falciparum_Sanger|(protein coc			
	K.CVVHG\	3.3158	0.2718	99.9	1240.619	1239.616		6645.4	1	5.262	90	3
MAL13P1.261		11	2.40%	423	50374	6.5	U		Annotation|Plasmodium_falciparum_Sanger|(protein coc			
	K.ICDDKIC	3.2393	0.2148	99.8	1295.58	1295.58		6766	1	5.376	77.8	11
PFI1590c		7	2.30%	1342	159114	6.9	U		Annotation|Plasmodium_falciparum_Sanger|(protein coc			
	K.ILLCHN	3.2562	0.3048	99.9	1835.934	1835.934		5767.2	1	6.45	53.6	1
	K.IRNEPF	4.7145	0.3889	100	1886.077	1885.073		6612	1	7.44	66.7	6
PF10_0046		6	2.30%	1130	131697	5.3	U		Annotation|Plasmodium_falciparum_TIGR|(protein codin			
	R.EEYKEN	2.5109	0.3497	99.8	1447.648	1447.645		3743.7	1	5.668	65	3
	R.KEDMC	3.8324	0.4388	100	1711.717	1711.713		5092.2	1	7.468	78.6	3
PF14_0455		26	2.30%	1024	119012	9.1	U		Annotation|Plasmodium_falciparum_TIGR|(protein codin			
	K.FCNALS	2.7807	0.2794	99.8	1281.589	1281.58		5741.9	1	4.892	77.8	7
	K.TSNDHN	4.1308	0.3885	100	1513.724	1513.722		5070.1	1	7.111	70.8	11
	K.TSNDHN	3.9452	0.3253	100	1641.82	1641.817		6392.1	1	7.76	65.4	8
PF14_0224		6	2.30%	959	112585	7	U		Annotation|Plasmodium_falciparum_TIGR|(protein codin			
	K.LSTEGL	3.5762	0.3699	100	2568.314	2566.294		5317.6	1	5.867	40.5	6
MAL7P1.208		2	2.30%	786	95646	4.3	U		Annotation|Plasmodium_falciparum_Sanger|(protein coc			
	K.NNNELL	3.4624	0.2899	99.9	2029.969	2028.936		5793.5	2	5.425	50	2
PF08_0082		3	2.30%	662	78564	5.1	U		Annotation|Plasmodium_falciparum_Sanger|(protein coc			
	K.NYNKEN	5.198	0.2615	100	1952.874	1951.867		5173.3	1	6.266	71.4	3
PF11_0090		1	2.30%	643	77180	9	U		Annotation|Plasmodium_falciparum_TIGR|(protein codin			
	R.IHIQPQ	3.219	0.4058	100	1939.005	1939.005		5982.1	1	6.034	60.7	1
PF07_0085		1	2.30%	642	72722	8.7	U		Annotation|Plasmodium_falciparum_Sanger|(protein coc			
	K.EFNFIP	3.1935	0.3222	99.9	1848.95	1848.951		4556.2	1	5.69	53.6	1
PF11_0439		2	2.30%	615	71890	4.9	U		Annotation|Plasmodium_falciparum_TIGR|(protein codin			
	K.NPFLNL	3.8227	0.3567	100	1680.85	1679.858		4525.3	1	5.872	76.9	2
PFC0405c		1	2.30%	608	71464	9.9	U		Annotation|Plasmodium_falciparum_Sanger|(protein coc			
	A.KTENVL	3.8893	0.2191	99.9	1558.792	1555.797		6942	1	4.65	73.1	1
PFD0485w		1	2.30%	575	68533	9	U		Annotation|Plasmodium_falciparum_Sanger|(protein coc			
	V.NEYVFN	3.1394	0.2737	99.8	1638.858	1638.886		10653.3	1	5.009	70.8	1
PF14_0409		6	2.30%	574	67605	8	U		Annotation|Plasmodium_falciparum_TIGR|(protein codin			
	K.NKYDEN	4.3444	0.3208	100	1666.766	1665.762		4080.9	1	5.913	70.8	6

PFL0175c		1	2.30%	556	64888	5.8	U		Annotation|Plasmodium_falciparum_Sanger|(protein coc				
	R.INHEDK	3.3819	0.2751	99.9	1561.794	1561.794		4284.9	23	4.707	58.3	1
PF11_0392		29	2.20%	2849	335918	6	U		Annotation|Plasmodium_falciparum_TIGR|(protein codin				
	K.NLKDDY	4.046	0.4758	100	2019.041	2019.055		7589.8	1	7.603	59.4	12
	K.MIDVNS	4.278	0.5049	100	2091.059	2091.045		9694	1	8.584	59.4	8
	K.NENNQI	4.0532	0.3691	100	1786.799	1786.796		7316.9	1	6.831	80.8	3
	K.VKVPDD	3.0723	0.3427	99.9	1608.767	1608.762		5403.8	1	6.072	61.5	6
PFD0875c		6	2.20%	1215	144834	5	U		Annotation|Plasmodium_falciparum_Sanger|(protein coc				
	K.TGKDHL	4.0949	0.386	100	1520.75	1520.746		4983	1	6.976	79.2	3
	K.NESNNN	3.5855	0.3911	100	1582.75	1582.746		5230.1	1	6.807	65.4	3
PF08_0045		13	2.20%	1038	121150	7.8	U		Annotation|Plasmodium_falciparum_Sanger|(protein coc				
	K.NIDFGT	3.8864	0.4095	100	2502.274	2502.274		6112.6	1	8.636	40.9	13
PF10_0307		17	2.20%	919	109277	8.6	U		Annotation|Plasmodium_falciparum_TIGR|(protein codin				
	K.GIGLES	5.3485	0.5396	100	2224.167	2224.158		9983.5	1	10.143	60.5	17
PF14_0364		3	2.20%	876	101184	5.5	U		Annotation|Plasmodium_falciparum_TIGR|(protein codin				
	K.NKVDEH	4.2833	0.3653	100	2276.124	2276.121		5235	1	6.535	40.3	3
PFL1890c		15	2.20%	852	99566	7.7	U		Annotation|Plasmodium_falciparum_Sanger|(protein coc				
	R.SLSQLG	4.7761	0.5386	100	2095.003	2095.003		5751	1	8.607	52.8	15
PF14_0724		22	2.20%	729	85803	7.2	U		Annotation|Plasmodium_falciparum_TIGR|(protein codin				
	K.SEDVFQ	4.8961	0.3082	100	1847.986	1846.98		8531	1	6.729	76.7	22
MAL7P1.209		12	2.20%	667	78628	9.4	U		Annotation|Plasmodium_falciparum_Sanger|(protein coc				
	R.NALATF	4.2199	0.4819	100	1639.815	1638.813		7814.4	1	8.426	60.7	12
PFC0465c		2	2.20%	600	71663	10	U		Annotation|Plasmodium_falciparum_Sanger|(protein coc				
	K.KEEHIA	4.4248	0.3575	100	1540.764	1540.761		7573.9	1	7.896	83.3	2
PF14_0401		2	2.20%	402	46441	7.3	U		Annotation|Plasmodium_falciparum_TIGR|(protein codin				
	K.HLNDLL	2.9612	0.3055	100	1141.571	1141.568		4924.8	1	6.022	87.5	2
PFD1110w		3	2.20%	372	42618	4.7	U		Annotation|Plasmodium_falciparum_Sanger|(protein coc				
	K.WSPELH	2.7208	0.2692	99.9	1011.503	1011.501		3789.3	1	4.783	100	3
PFL1410c		21	2.10%	2108	248301	8.7	U		Annotation|Plasmodium_falciparum_Sanger|(protein coc				
	K.SLISGVS	4.0893	0.374	100	2582.517	2582.512		5408.6	1	6.751	41.3	10
	R.NISYLY	3.8128	0.3891	100	2538.264	2538.266		5189.2	1	7.576	47.5	11
PF13_0199		2	2.10%	1609	193637	5.4	U		Annotation|Plasmodium_falciparum_Sanger|(protein coc				
	K.NLNHET	3.779	0.372	100	1437.72	1437.72		5681	1	6.692	86.4	1
	K.NNHLHN	5.7476	0.5109	100	2497.237	2497.237		7804	1	8.736	40	1
PFL1240c		10	2.10%	1501	178073	5.6	U		Annotation|Plasmodium_falciparum_Sanger|(protein coc				
	K.LLDEHK	4.6784	0.3559	100	2072.993	2072.989		7853.7	1	6.138	67.6	3

	K.LNNLVN	4.1893	0.2861	100	1637.884	1636.877		5667.5	1	5.187	69.2	7
PFE0270c		3	2.10%	1350	156439	6.4	U		Annotation|Plasmodium_falciparum_Sanger|(protein coc			
	R.TILAQL	3.3919	0.3314	99.9	1810.014	1806.968		4196	2	5.859	56.7	2
	K.LKEFVT	3.0846	0.2633	99.8	1456.83	1456.82		6380.6	1	4.734	72.7	1
PF08_0130		1	2.10%	1121	129051	8	U		Annotation|Plasmodium_falciparum_Sanger|(protein coc			
	E.DVSRDD	3.4716	0.3391	100	2619.148	2619.1		8019.8	1	5.636	38.6	1
PF11_0037		6	2.10%	624	73889	6	U		Annotation|Plasmodium_falciparum_TIGR|(protein codin			
	K.RKPSEI	3.3991	0.2741	99.9	1528.82	1528.827		5591.6	1	5.982	62.5	5
	K.RKPSEI	3.5624	0.3987	100	1528.827	1528.827		6813.8	1	7.076	50	1
PFC0160w		3	2.10%	616	73863	8.2	U		Annotation|Plasmodium_falciparum_Sanger|(protein coc			
	R.NICVTCI	3.2632	0.307	99.9	1605.887	1605.887		5378.3	1	5.851	58.3	3
PFI1680w		7	2.10%	531	62570	7	U		Annotation|Plasmodium_falciparum_Sanger|(protein coc			
	K.NNIHIND	4.1356	0.4236	100	1271.713	1271.712		6229	1	6.953	85	7
PFC0185w		3	2.10%	525	61765	7.1	U		Annotation|Plasmodium_falciparum_Sanger|(protein coc			
	R.NFCNVN	3.1429	0.2653	99.9	1424.626	1424.623		6822.5	1	5.898	75	3
PF11_0227		1	2.10%	525	61682	6.8	U		Annotation|Plasmodium_falciparum_TIGR|(protein codin			
	K.LNTPGH	3.0859	0.3151	99.9	1170.671	1170.671		4164.6	1	6.23	75	1
PFB0815w		10	2.10%	524	60800	7.6	U		Annotation|Plasmodium_falciparum_TIGR|(protein codin			
	K.NVEEEV	3.723	0.3539	100	1308.679	1308.676		5840	1	6.632	85	10
PF10_0306		3	2.10%	422	48734	9	U		Annotation|Plasmodium_falciparum_TIGR|(protein codin			
	R.HGMGTI	3.1162	0.4071	100	1040.461	1038.45		4256.7	1	6.772	87.5	3
PFF0645c		35	2.00%	1347	163178	8.8	U		Annotation|Plasmodium_falciparum_Sanger|(protein coc			
	K.NDLYNT	4.8418	0.4078	100	1840.884	1838.875		9198.9	1	7.692	80.8	29
	K.KMQEHI	3.5376	0.3066	100	1658.755	1658.763		6024.6	1	5.07	70.8	6
PF14_0056		1	2.00%	1105	130405	9	U		Annotation|Plasmodium_falciparum_TIGR|(protein codin			
	C.GNIITI	3.0637	0.3117	99.8	2480.356	2479.313		3594.1	1	5.236	35.7	1
PF11_0374		8	2.00%	1098	128545	8.6	U		Annotation|Plasmodium_falciparum_TIGR|(protein codin			
	K.KLNTPL	3.2153	0.2383	99.8	1230.678	1227.67		5198.8	1	5.527	85	6
	K.NRKEEL	3.1739	0.2318	99.8	1399.809	1399.802		4822.7	1	5.198	70	2
PFI0925w		15	2.00%	1063	124462	5.5	U		Annotation|Plasmodium_falciparum_Sanger|(protein coc			
	K.VHNSNN	3.2089	0.2071	99.8	1183.608	1183.605		5165.7	5	5.511	72.2	8
	R.NFILNH	3.8935	0.2741	100	1352.698	1352.708		7469.6	1	5.478	80	7
PF13_0153		11	2.00%	1004	115889	5.9	U		Annotation|Plasmodium_falciparum_Sanger|(protein coc			
	K.DKENTT	5.2569	0.3748	100	2314.18	2313.174		6024.8	1	6.831	40.8	6
	K.DKENTT	5.0227	0.4265	100	2314.182	2313.174		5407.1	1	7.748	55.3	5
PFE0520c		2	2.00%	839	98109	9.3	U		Annotation|Plasmodium_falciparum_Sanger|(protein coc			

	M.DPRITV	3.6973	0.2825	99.9	2065.091	2063.1		7594	4	5.038	50	2
PF08_0094		4	2.00%	804	97163	6	U		Annotation|Plasmodium_falciparum_Sanger|(protein coc			
	R.TYILFS	3.2008	0.3289	99.9	1870.013	1870.012		7768.8	1	5.862	46.7	4
MAL8P1.58		6	2.00%	661	78063	7.8	U		Annotation|Plasmodium_falciparum_Sanger|(protein coc			
	K.KYEQN\	4.0003	0.3133	100	1533.768	1533.766		6207.9	1	6.566	83.3	6
PF10_0071		6	2.00%	653	75108	5.6	U		Annotation|Plasmodium_falciparum_TIGR|(protein codin			
	K.TLLTIVT	3.2205	0.4222	100	1429.868	1429.867		8749.4	1	6.107	62.5	6
PFL2460w		14	2.00%	602	69015	6	U		Annotation|Plasmodium_falciparum_Sanger|(protein coc			
	K.HISSIH	4.9824	0.4542	100	1468.737	1468.737		5286.2	1	7.729	90.9	13
	K.HISSIH	3.5377	0.2907	99.9	1468.738	1468.737		7704.7	1	4.911	56.8	1
PF14_0694		1	2.00%	553	66518	5.7	U		Annotation|Plasmodium_falciparum_TIGR|(protein codin			
	K.IDHLYN	2.9229	0.2673	99.8	1473.735	1472.729		7572.8	1	4.751	80	1
MAL8P1.109		6	2.00%	545	63139	5.2	U		Annotation|Plasmodium_falciparum_Sanger|(protein coc			
	R.ASHFQE	3.5735	0.2996	100	1365.643	1365.644		8123	1	6.767	75	6
MAL13P1.132		4	2.00%	490	59500	4.8	U		Annotation|Plasmodium_falciparum_Sanger|(protein coc			
	K.LVEHIQ	3.4669	0.2512	99.9	1211.647	1210.643		8003	1	5.987	83.3	4
PFI1720w		1	2.00%	512	58344	4.1	U		Annotation|Plasmodium_falciparum_Sanger|(protein coc			
	R.LHSSIE	3.0942	0.3211	100	1148.622	1147.619		3912.9	1	5.706	88.9	1
PFI0165c		9	1.90%	2536	300924	6.9	U		Annotation|Plasmodium_falciparum_Sanger|(protein coc			
	K.LFQNYN	2.8889	0.2778	99.9	1180.65	1180.648		5750.6	2	5.773	87.5	3
	R.NAFIIHC	2.8708	0.2919	99.9	1016.555	1016.554		5939	1	5.193	92.9	2
	K.KLEDIN	4.0352	0.3425	100	2168.164	2168.14		4329.1	3	6.248	46.9	1
	K.LYIFEQ	3.5356	0.3269	100	1643.879	1640.858		2945.8	1	5.525	58.3	3
PF10_0183		8	1.90%	2405	280754	8.6	U		Annotation|Plasmodium_falciparum_TIGR|(protein codin			
	K.KKNDN	5.1955	0.4116	100	2046.975	2046.985		4307.1	1	7.368	67.6	6
	R.VWSEM	2.6544	0.3524	99.8	1840.884	1840.877		8334.8	1	5.712	64.3	1
	K.PQPVLQ	3.325	0.2248	99.8	1481.776	1480.769		5851.8	2	5.546	72.7	1
PF07_0101		12	1.90%	2190	260765	5.7	U		Annotation|Plasmodium_falciparum_Sanger|(protein coc			
	K.LSIDLL	4.4208	0.2475	100	1631.876	1629.859		7862.4	1	6.104	76.9	4
	K.KADKGE	3.7789	0.2906	100	1344.728	1344.724		6997.5	1	6.377	86.4	6
	Y.RNKPSS	3.8122	0.3776	100	1857.954	1857.942		4659.6	1	6.866	56.7	2
PF08_0060		4	1.90%	2235	254753	5.2	U		Annotation|Plasmodium_falciparum_Sanger|(protein coc			
	R.INIKDE	4.2863	0.3474	100	3529.76	3528.75		8196.8	1	5.796	27.6	1
	K.SVVDGN	3.4418	0.3374	100	1468.741	1468.74		7798.1	1	6.239	70.8	3
PF11_0218		9	1.90%	1464	175530	5	U		Annotation|Plasmodium_falciparum_TIGR|(protein codin			
	K.EKDFVE	3.6903	0.2172	99.9	1619.89	1618.884		8603	1	5.183	70.8	7

	K.NKYEEE	5.0164	0.3182	100	1880.968	1880.961		5712.4		1	6.49	78.6	2	
PFF0830w		2	1.90%	1236	145364	8.6	U		Annotation|Plasmodium_falciparum_Sanger|(protein coc					
	K.ALTQVH	4.5442	0.4081	100	1759.942	1757.933		6763.9		1	7.601	63.3	1	
	K.HLNNYL	2.702	0.2778	99.9	1015.533	1015.533		5449.2		1	5.745	92.9	1	
PF14_0417		3	1.90%	927	106999	5.5	U		Annotation|Plasmodium_falciparum_TIGR|(protein codin					
	K.VMDIIV	3.7847	0.3669	100	2142.172	2141.132		9478.8		1	7.392	52.9	3	
MAL13P1.321		1	1.90%	827	97401	6.9	U		Annotation|Plasmodium_falciparum_Sanger|(protein coc					
	Y.YLKEIS	3.4331	0.2889	99.9	1900.066	1897.018		5550.6		1	5.622	56.7	1	
PFB0750w		1	1.90%	722	86256	7	U		Annotation|Plasmodium_falciparum_TIGR|(protein codin					
	R.HQSVFN	3.3103	0.2789	99.9	1733.902	1733.905		6618.3		4	6.06	50	1	
PF13_0290		2	1.90%	637	76565	5.8	U		Annotation|Plasmodium_falciparum_Sanger|(protein coc					
	K.NLNDLH	3.9066	0.2696	100	1409.754	1409.75		5574.6		2	5.656	68.2	2	
PF10_0130		2	1.90%	628	75469	9.3	U		Annotation|Plasmodium_falciparum_TIGR|(protein codin					
	K.NNNIHIN	4.2879	0.3629	100	1462.807	1462.807		6445.9		1	6.741	86.4	2	
PFL1485w		3	1.90%	617	75440	8.7	U		Annotation|Plasmodium_falciparum_Sanger|(protein coc					
	K.NYDDEE	4.0299	0.43	100	1583.605	1581.598		5061.5		1	7.417	86.4	3	
PFD0095c		6	1.90%	575	67788	4.6	U		Annotation|Plasmodium_falciparum_Sanger|(protein coc					
	K.LNGTHY	3.0123	0.2873	99.9	1288.666	1288.665		8061.1		1	5.326	80	4	
	N.GTHYID	2.597	0.3229	99.9	1061.537	1061.538		4626.4		3	6.923	81.2	2	
PF13_0252		1	1.90%	422	47631	8.1	U		Annotation|Plasmodium_falciparum_Sanger|(protein coc					
	K.AYADIE	3.0795	0.2714	100	931.4651	931.4598		3399		2	5.288	92.9	1	
PF11_0507		34	1.80%	5507	621179	3.8	U		Annotation|Plasmodium_falciparum_TIGR|(protein codin					
	-.MKELES	4.2815	0.1389	99.9	1366.657	1366.657		7259.7		1	5.165	90	6	
	K.EQESVL	4.3783	0.4013	100	2483.228	2482.22		7929.1		1	6.77	45.2	3	
	K.INEEVE	5.5695	0.268	100	2512.291	2511.287		6224.3		1	5.723	57.5	20	
	K.ELPQEE	5.4475	0.5117	100	4109.001	4106.986		6875.4		1	7.799	25	1	
	K.SVSEEI	2.9278	0.2654	99.8	1252.637	1252.639		7010.2		1	5.152	75	4	
MAL13P1.26		11	1.80%	3525	418583	7.1	U		Annotation|Plasmodium_falciparum_Sanger|(protein coc					
	K.ILSDYI	4.0696	0.3496	100	2546.283	2544.281		4769		1	5.854	47.5	7	
	K.FDDLDV	3.514	0.2865	99.8	2766.352	2763.315		5485.4		1	5.69	40.9	3	
	K.VLGINV	4.2309	0.4756	100	2201.228	2201.227		5083.2		1	7.83	50	1	
PFI0240c		105	1.80%	2563	298807	5.8	U		Annotation|Plasmodium_falciparum_Sanger|(protein coc					
	K.NYESNH	4.4106	0.4515	100	1804.768	1804.764		3622.6		1	7.633	69.2	3	
	K.HGQDV	5.1906	0.4656	100	1755.824	1755.815		6983.1		1	9.086	67.9	54	
	K.HGQDV	5.4237	0.2972	100	2055.965	2055.958		5934.3		1	8.97	64.7	30	
	K.HGQDV	4.5693	0.3612	100	2056.965	2055.958		8076.5		1	6.439	44.1	18	

PFL0290w		3	1.80%	2284	267019	6.7	U		Annotation|Plasmodium_falciparum_Sanger|(protein coc			
	K.NVEHTH	3.0928	0.3285	99.9	1716.816	1715.822		4220.5	2	5.649	53.8	1
	Y.FNEKLK	3.8343	0.3594	99.9	3401.454	3400.51		5776.8	1	5.091	25	2
MAL13P1.295		15	1.80%	2025	235138	6.2	U		Annotation|Plasmodium_falciparum_Sanger|(protein coc			
	K.WKENQI	5.643	0.4764	100	2400.144	2399.134		5140.1	1	8.728	67.5	3
	K.DVWFPC	3.7505	0.3479	100	1878.964	1877.959		4321.6	1	6.657	60	12
PFL2335w		14	1.80%	1989	231317	6.3	U		Annotation|Plasmodium_falciparum_Sanger|(protein coc			
	R.VVNFHE	3.282	0.3172	99.8	1671.913	1671.911		5590.4	1	6.443	55.8	3
	R.VVNFHE	3.8234	0.3651	100	1671.914	1671.911		8496	1	6.925	69.2	9
	K.HDINSTI	3.0538	0.308	99.9	1247.691	1247.69		7521.8	2	5.606	75	1
	K.LNYNNG	2.7625	0.2881	99.8	1155.583	1155.58		6401.9	1	6.459	77.8	1
PF07_0016		18	1.80%	1859	218375	6.8	U		Annotation|Plasmodium_falciparum_Sanger|(protein coc			
	K.KNNNKE	4.7152	0.3209	100	1559.757	1559.757		4305.6	1	7.048	87.5	12
	K.NNNKEN	3.0179	0.2896	99.9	1431.662	1431.662		4265.5	4	5.981	72.7	3
	K.NEDIEE	5.8585	0.49	100	2360.169	2359.161		6574.8	1	8.086	65.8	3
PF14_0647		11	1.80%	1656	196055	6.9	U		Annotation|Plasmodium_falciparum_TIGR|(protein codin			
	R.NNTEHN	4.6387	0.4072	100	1462.736	1462.734		5198.8	1	8.243	72.7	7
	R.NNINNI	4.375	0.3513	100	2177.992	2177.013		6066.5	1	5.842	58.8	4
PF07_0012		3	1.80%	1540	182541	6.4	U		Annotation|Plasmodium_falciparum_Sanger|(protein coc			
	R.QAGGQ	4.8613	0.5735	100	2400.102	2399.094		10200.7	1	10.284	42.3	3
PFL0465c		7	1.80%	1461	170852	8.7	U		Annotation|Plasmodium_falciparum_Sanger|(protein coc			
	K.KNDNIN	4.1003	0.2751	100	1452.777	1452.775		5992.9	1	6.249	81.8	4
	R.HVNSVH	3.3851	0.3186	99.9	1840.836	1840.84		4822	1	5.937	60.7	3
PF13_0120		4	1.80%	1442	170672	8.8	U		Annotation|Plasmodium_falciparum_Sanger|(protein coc			
	K.LKEQEV	4.6389	0.3112	100	1873.967	1873.973		7100.7	1	6.272	64.3	3
	K.NGYIIH	3.1032	0.2425	99.8	1286.67	1285.665		7664.1	1	4.67	80	1
PF14_0120		7	1.80%	956	112774	6.4	U		Annotation|Plasmodium_falciparum_TIGR|(protein codin			
	K.IPEDITN	3.8649	0.3855	100	2009.051	2009.06		7976.4	1	8.226	59.4	6
	I.PEDITNY	4.3731	0.4881	100	1910.011	1910.01		7421.6	1	8.085	70	1
PF14_0512		3	1.80%	961	111445	5.1	U		Annotation|Plasmodium_falciparum_TIGR|(protein codin			
	K.TNNINN	3.8402	0.3328	100	1985.967	1985.964		5103.8	1	5.942	56.2	3
PF07_0030		3	1.80%	912	108907	9.6	U		Annotation|Plasmodium_falciparum_Sanger|(protein coc			
	K.LKEDEE	5.9252	0.3004	100	1974.966	1974.962		5735.5	1	6.91	73.3	3
PF11_0375		3	1.80%	886	107110	8.4	U		Annotation|Plasmodium_falciparum_TIGR|(protein codin			
	K.EQLVQE	3.7567	0.4011	100	2051	2049.996		5259.8	1	7.332	60	3
PFB0895c		21	1.80%	904	104185	9	U		Annotation|Plasmodium_falciparum_TIGR|(protein codin			

	K.WNVFS(	5.252	0.4896	100	1851.954	1850.951		7948.1	1	8.184	76.7	21	
PF14_0630		30	1.80%	889	100775	7.3	U		Annotation|Plasmodium_falciparum_TIGR|(protein codin				
	K.RPLVVS	5.3275	0.4179	100	1850.034	1849.03		5739.4	1	6.46	76.7	30	
PF11_0321		2	1.80%	773	94428	8	U		Annotation|Plasmodium_falciparum_TIGR|(protein codin				
	R.FGPVIE	3.0883	0.3334	99.9	1665.827	1665.828		6171.8	1	5.9	61.5	2	
PFE0340c		1	1.80%	759	86654	9.1	U		Annotation|Plasmodium_falciparum_Sanger|(protein coc				
	K.FSGVHS	3.4251	0.3025	99.9	1752.78	1751.778		6462.5	1	6.164	65.4	1	
MAL7P1.151		10	1.80%	706	83784	8.5	U		Annotation|Plasmodium_falciparum_Sanger|(protein coc				
	N.EHEKTQ	3.803	0.237	99.9	1654.977	1653.976		7970.8	1	5.219	66.7	10	
PFD0462w		1	1.80%	672	75916	9.2	U		Annotation|Plasmodium_falciparum_Sanger|(protein coc				
	K.DNTNIS	3.3025	0.3765	100	1453.744	1453.737		5705.7	1	6.234	68.2	1	
PFF0675c		30	1.70%	2153	255083	4.9	U		Annotation|Plasmodium_falciparum_Sanger|(protein coc				
	K.VEDNII	4.2525	0.3404	100	2107.053	2107.045		9636.5	1	6.473	52.9	2	
	K.EDLSNS	4.0646	0.4151	100	2215.054	2214.051		6164.9	1	7.299	61.8	28	
PF11_0464		19	1.70%	2075	246146	6.2	U		Annotation|Plasmodium_falciparum_TIGR|(protein codin				
	R.LSLQNV	5.4006	0.3117	100	2927.496	2927.473		10129.4	1	5.912	32.3	9	
	R.LSLQNV	3.6182	0.4509	100	2928.493	2927.473		6442.7	1	7.585	43.8	7	
	R.MYDQTI	2.853	0.298	99.9	1392.624	1392.622		5894.9	1	5.821	70	3	
PF10_0205		3	1.70%	1934	231384	6.6	U		Annotation|Plasmodium_falciparum_TIGR|(protein codin				
	K.SGMHNI	5.1282	0.1936	100	2205.968	2205.956		3941.1	1	8.782	66.7	2	
	R.FNNNNN	3.8927	0.3728	100	1611.803	1611.793		4762.8	1	6.082	66.7	1	
PF14_0337		2	1.70%	1788	210711	8.6	U		Annotation|Plasmodium_falciparum_TIGR|(protein codin				
	R.NNINNI	4.277	0.2344	100	2239.04	2239.036		3961.2	1	6.446	50	1	
	K.EINNLQ	3.2623	0.244	99.8	1496.733	1496.732		3611.2	80	5.644	54.5	1	
PF14_0487		4	1.70%	1504	176230	5.6	U		Annotation|Plasmodium_falciparum_TIGR|(protein codin				
	R.INHTEN	4.4459	0.3997	100	1540.793	1540.791		4068.7	1	7.408	76.9	1	
	R.STHINN	3.2212	0.3542	100	1376.701	1376.7		5530	1	7.83	77.3	3	
PF13_0354		12	1.70%	1408	165135	8.9	U		Annotation|Plasmodium_falciparum_Sanger|(protein coc				
	K.HQLVSD	2.5746	0.3382	99.8	1302.644	1302.644		7548.4	1	5.405	80	3	
	K.NVHNIN	4.3948	0.391	100	1544.802	1541.794		6491.5	2	7.372	70.8	8	
	V.HNINPT	3.4259	0.3479	100	1328.682	1328.683		5614.6	1	6.461	75	1	
PF14_0048		2	1.70%	1095	131984	5.8	U		Annotation|Plasmodium_falciparum_TIGR|(protein codin				
	R.KKNDDI	5.1413	0.2661	100	2290.165	2289.158		6043.9	4	5.705	33.3	2	
PF13_0291		36	1.70%	929	105674	5.3	U		Annotation|Plasmodium_falciparum_Sanger|(protein coc				
	R.IHPNYA	5.3098	0.4187	100	1710.93	1708.921		4354.5	1	7.016	76.7	27	
	R.IHPNYA	6.3835	0.3675	100	1710.93	1708.921		8192.6	1	7.679	58.3	9	

PF14_0100		3	1.70%	858	98517	6.5	U		Annotation|Plasmodium_falciparum_TIGR|(protein codin			
	G.KTVQVV	4.301	0.4658	100	1669.974	1669.971		6120.3	1	6.562	78.6	3
PF13_0238		1	1.70%	726	83665	5.9	U		Annotation|Plasmodium_falciparum_Sanger|(protein coc			
	K.AWVEV/	3.0409	0.2983	99.9	1352.734	1352.733		6456.7	1	5.216	81.8	1
PFE1445c		6	1.70%	719	82905	5.4	U		Annotation|Plasmodium_falciparum_Sanger|(protein coc			
	K.TLGGNA	3.4921	0.4175	100	1201.623	1199.617		5140.7	1	6.56	77.3	6
PF14_0580		3	1.70%	635	75928	7.8	U		Annotation|Plasmodium_falciparum_TIGR|(protein codin			
	K.YKDDAS	2.7615	0.3189	99.9	1212.588	1212.586		5638.4	1	6.036	90	3
PF11_0254		2	1.70%	525	62334	5.1	U		Annotation|Plasmodium_falciparum_TIGR|(protein codin			
	K.LAFQLLI	2.7594	0.3193	99.9	1032.647	1032.646		4819.5	1	5.45	87.5	2
MAL13P1.333		8	1.60%	3482	417183	5.6	U		Annotation|Plasmodium_falciparum_Sanger|(protein coc			
	R.TESHIL	4.8023	0.2962	100	2284.091	2283.085		5627.8	1	7.721	55.6	3
	K.DFLNNM	4.0367	0.4656	100	2388.194	2388.191		8896.7	1	7.866	55.3	2
	K.NKNDNI	5.4382	0.3671	100	2048.998	2048.994		4105.9	1	7.35	68.8	1
	K.NDNINN	3.5926	0.266	99.9	1793.837	1792.821		3857.5	6	5.218	50	2
PFD1060w		52	1.60%	2874	337908	5.8	U		Annotation|Plasmodium_falciparum_Sanger|(protein coc			
	R.FLLGDN	5.2228	0.4383	100	2597.338	2596.333		6726.1	1	7.905	52.3	42
	K.YENFHI	2.9397	0.3462	100	1056.532	1056.534		6918.4	1	6.229	92.9	4
	K.KTEEGC	5.4131	0.5165	100	1945.934	1945.929		7432.4	1	9.407	86.7	6
MAL8P1.82		15	1.60%	1833	214691	6.6	U		Annotation|Plasmodium_falciparum_Sanger|(protein coc			
	R.KGENQY	3.9418	0.3219	100	1670.749	1670.744		5693.5	1	7.499	73.1	12
	K.SGLNNF	4.3633	0.3926	100	1742.86	1742.857		4028.7	1	6.814	60.7	3
PFL1680w		1	1.60%	1329	151811	5.9	U		Annotation|Plasmodium_falciparum_Sanger|(protein coc			
	R.ILQHGL	4.5504	0.4106	100	2315.281	2315.28		7259.6	1	7.963	57.5	1
PFL0440c		2	1.60%	1180	138954	6.6	U		Annotation|Plasmodium_falciparum_Sanger|(protein coc			
	L.NNIHIN	3.6783	0.286	99.9	2338.077	2338.114		3738.2	9	4.764	47.2	2
PFD0460c		1	1.60%	1010	120659	7.1	U		Annotation|Plasmodium_falciparum_Sanger|(protein coc			
	K.NDYSAH	4.475	0.4695	100	1977.983	1977.983		6263.3	1	7.666	60	1
PF11_0107		9	1.60%	891	105638	5.4	U		Annotation|Plasmodium_falciparum_TIGR|(protein codin			
	R.HANAN/	3.9054	0.4609	100	1529.745	1529.746		5639.4	1	8.682	65.4	9
PF14_0412		6	1.60%	819	95825	6.4	U		Annotation|Plasmodium_falciparum_TIGR|(protein codin			
	K.LEPTWS	3.4876	0.235	99.8	1561.828	1561.827		4346.1	1	5.428	75	6
PFF1030w		1	1.60%	793	94284	8.9	U		Annotation|Plasmodium_falciparum_Sanger|(protein coc			
	K.IFMSTN	3.2671	0.3065	99.9	1499.772	1497.76		2536	37	5.448	62.5	1
PF11_0348		2	1.60%	794	93831	8.8	U		Annotation|Plasmodium_falciparum_TIGR|(protein codin			
	K.NVDQCI	2.9712	0.3883	100	1563.761	1562.757		7725.6	1	6.351	62.5	2

PF14_0569		5	1.60%	801	93594	8.7	U		Annotation|Plasmodium_falciparum_TIGR|(protein codin				
	K.TSNINY	4.6358	0.39	100	1561.776	1561.784		4741.6		1	7.673	75	5
PFL1310c		5	1.60%	742	87020	8.1	U		Annotation|Plasmodium_falciparum_Sanger|(protein coc				
	K.HKEIIQ	3.9537	0.1861	99.9	1465.754	1465.751		5150.9		1	5.374	86.4	5
PF11_0146		6	1.60%	702	85752	9.2	U		Annotation|Plasmodium_falciparum_TIGR|(protein codin				
	K.NTDNLV	3.2269	0.3505	100	1295.626	1293.619		2975.7		1	5.427	70	6
PF11_0433		3	1.50%	2738	324826	5.9	U		Annotation|Plasmodium_falciparum_TIGR|(protein codin				
	K.NNNNNI	5.5358	0.4526	100	3496.629	3495.621		5920.3		1	7.619	26.7	1
	K.NVQEQI	3.4142	0.2313	99.9	1272.635	1272.634		7807.8		1	5.025	83.3	2
PFI1275w		1	1.50%	1277	153344	8.9	U		Annotation|Plasmodium_falciparum_Sanger|(protein coc				
	I.KIDGKK	3.2762	0.3088	99.9	2200.186	2199.211		4487.8		11	4.908	33.3	1
PF14_0196		1	1.50%	1245	148025	7	U		Annotation|Plasmodium_falciparum_TIGR|(protein codin				
	K.LIHFYE	4.4515	0.4766	100	2319.283	2319.283		6822.7		1	7.917	66.7	1
PF13_0265		3	1.50%	1144	133580	7.5	U		Annotation|Plasmodium_falciparum_Sanger|(protein coc				
	K.KLSEGT	4.3436	0.3865	100	1970.117	1970.1		7700.2		1	5.727	53.1	3
PF11_0463		5	1.50%	1068	124369	6.9	U		Annotation|Plasmodium_falciparum_TIGR|(protein codin				
	K.HIWDSN	5.3502	0.537	100	1936.912	1936.909		10635.6		1	9.739	76.7	5
PF11_0112		24	1.50%	1050	123467	6.4	U		Annotation|Plasmodium_falciparum_TIGR|(protein codin				
	R.SPNEDI	4.9183	0.5627	100	1850.8	1850.806		4684.5		1	10.066	63.3	21
	R.SPNEDI	3.8582	0.3423	100	1851.824	1850.806		8099.4		1	5.558	40	3
PF10_0171		4	1.50%	971	115082	5.4	U		Annotation|Plasmodium_falciparum_TIGR|(protein codin				
	K.EHPTVN	3.3245	0.3179	99.9	1738.865	1737.856		6795.8		1	6.33	64.3	4
PFC0240c		1	1.50%	956	114503	5.6	U		Annotation|Plasmodium_falciparum_Sanger|(protein coc				
	R.NSNIND	4.151	0.4417	100	1673.786	1673.784		6045.4		1	7.485	69.2	1
PFL0815w		2	1.50%	939	111065	8.6	U		Annotation|Plasmodium_falciparum_Sanger|(protein coc				
	K.VLPYVS	3.0375	0.3256	99.9	1729.901	1729.888		5703.2		1	6.52	69.2	2
PF10_0327		3	1.50%	915	108410	6.6	U		Annotation|Plasmodium_falciparum_TIGR|(protein codin				
	R.HLRPGE	3.3294	0.3748	100	1545.806	1545.803		3692.5		1	6.075	69.2	3
PFI0175w		1	1.50%	742	88671	5.5	U		Annotation|Plasmodium_falciparum_Sanger|(protein coc				
	K.KLENQII	3.8619	0.2764	100	1378.773	1378.777		7422.9		1	5.908	80	1
PFC0500w		2	1.50%	609	73524	6.2	U		Annotation|Plasmodium_falciparum_Sanger|(protein coc				
	K.HSIYNL	3.0671	0.2594	99.9	1102.566	1102.565		2983.9		2	5.998	75	2
MAL13P1.176		22	1.40%	3179	374192	5.3	U		Annotation|Plasmodium_falciparum_Sanger|(protein coc				
PF13_0198		22	1.50%	3130	370437	5.6	U		Annotation|Plasmodium_falciparum_Sanger|(protein coc				
	K.NEEILNI	4.5275	0.4526	100	2096.084	2096.082		6467.1		1	7.723	68.8	17
	K.NLSHDI	4.2529	0.3274	100	1639.752	1639.747		5159.1		1	7.207	80.8	3

	K.ILIDDIC	3.4609	0.4566	100	1774.896	1774.89		9265.6	1	7.701	75	2
PFC0325c		6	1.40%	1826	216257	6.6	U		Annotation|Plasmodium_falciparum_Sanger|(protein coc			
	K.KNELNE	3.7085	0.4445	100	1564.732	1564.728		7527	1	7.151	77.3	3
	R.DQYHFY	4.2267	0.4837	100	1838.764	1838.762		5989.3	1	8.944	73.1	3
PFC0105w		1	1.40%	1338	157372	6	U		Annotation|Plasmodium_falciparum_Sanger|(protein coc			
	K.NKDNHN	3.5745	0.2924	99.9	2345.025	2345.019		4151.1	2	6.041	41.7	1
PFI1305w		4	1.40%	1327	156850	9.2	U		Annotation|Plasmodium_falciparum_Sanger|(protein coc			
	E.LFMLLTI	3.7232	0.2481	99.9	2160.185	2160.185		7583.8	1	4.573	50	4
PF14_0692		2	1.40%	1281	156158	9.9	U		Annotation|Plasmodium_falciparum_TIGR|(protein codin			
	E.IKYKSE	3.7416	0.2478	99.9	2103.154	2103.12		7969.8	4	5.039	44.1	2
PFI0170w		35	1.40%	1384	155151	6	U		Annotation|Plasmodium_falciparum_Sanger|(protein coc			
	K.ELDQEM	6.0967	0.2782	100	2223.137	2222.127		10595.4	1	8.49	80.6	35
PF07_0047		3	1.40%	1229	142079	9	U		Annotation|Plasmodium_falciparum_Sanger|(protein coc			
	K.TPCIIFI	4.0456	0.2599	99.9	2005.036	2005.043		7321.2	1	5.59	59.4	3
PF11_0350		3	1.40%	1060	125978	4.9	U		Annotation|Plasmodium_falciparum_TIGR|(protein codin			
	K.NVQEKE	4.2443	0.2183	99.9	1819.918	1818.909		7124.1	1	5.688	78.6	3
PF14_0494		1	1.40%	1029	120667	8.1	U		Annotation|Plasmodium_falciparum_TIGR|(protein codin			
	R.HFIGCP	3.4939	0.2761	99.9	1697.893	1695.919		8199.7	1	5.406	76.9	1
PF11_0424		3	1.40%	1012	120381	9.1	U		Annotation|Plasmodium_falciparum_TIGR|(protein codin			
	K.LNHNDN	3.2556	0.2914	99.9	1707.775	1707.762		3872.5	1	5.208	53.8	3
PF13_0230		2	1.40%	972	119242	9.5	U		Annotation|Plasmodium_falciparum_Sanger|(protein coc			
	I.GSDYYI	3.4044	0.2582	99.9	1707.89	1707.878		8605.3	2	5.573	57.7	2
MAL7P1.83		3	1.40%	936	111788	7.4	U		Annotation|Plasmodium_falciparum_Sanger|(protein coc			
	K.HGDTDF	4.1083	0.3424	100	1531.737	1531.739		5253.3	1	7.253	75	3
PF11_0311		4	1.40%	940	111658	7.8	U		Annotation|Plasmodium_falciparum_TIGR|(protein codin			
	R.CFVRPS	2.7566	0.365	99.9	1548.794	1548.796		5244.9	1	6.454	62.5	4
PFF1485w		1	1.40%	946	111412	5.8	U		Annotation|Plasmodium_falciparum_Sanger|(protein coc			
	K.ALNDHV	3.1137	0.2927	99.9	1575.855	1575.854		6789.2	1	5.876	79.2	1
PF13_0091		3	1.40%	906	106123	8.2	U		Annotation|Plasmodium_falciparum_Sanger|(protein coc			
	K.YFRDDE	3.287	0.3373	100	1709.776	1708.772		5104.3	1	5.671	79.2	3
PF11_0329		3	1.40%	882	101123	6.5	U		Annotation|Plasmodium_falciparum_TIGR|(protein codin			
	R.NNDIIN	4.565	0.2391	100	1414.745	1414.745		7121.8	1	5.993	86.4	3
PFE0750c		6	1.40%	848	98383	5.7	U		Annotation|Plasmodium_falciparum_Sanger|(protein coc			
	R.GCCAY(	3.6565	0.5318	100	1502.602	1502.598		7279.8	1	8.712	72.7	6
PFF0560c		1	1.40%	830	97790	5.1	U		Annotation|Plasmodium_falciparum_Sanger|(protein coc			
	S.EIHETE	3.4692	0.2704	99.9	1514.774	1514.771		5945.5	1	5.341	68.2	1

PF13_0109		1	1.40%	804	94817	8.3	U		Annotation|Plasmodium_falciparum_Sanger|(protein coc			
	K.NNIHCN	2.7031	0.3789	100	1443.647	1442.642		5159.5	1	6.272	70	1
PF10_0326		9	1.40%	709	83230	6.7	U		Annotation|Plasmodium_falciparum_TIGR|(protein codin			
	K.KGVLDY	3.2267	0.3047	100	1191.674	1191.674		7009.2	1	5.904	88.9	9
MAL7P1.17		20	1.30%	3559	425267	8.8	U		Annotation|Plasmodium_falciparum_Sanger|(protein coc			
	R.YNTNHT	3.1354	0.3656	100	1409.66	1409.656		4018.6	1	6.826	80	4
	K.NINQHI	5.5601	0.5005	100	2669.302	2669.289		4432.1	1	9.011	57.1	5
	K.NINQHI	4.2894	0.3206	100	2669.304	2669.289		4845.5	2	5.541	29.8	2
	K.TKNEHE	5.107	0.4159	100	1783.821	1782.816		5115.6	1	7.087	76.9	9
PFF0920c		3	1.30%	1869	222645	5.6	U		Annotation|Plasmodium_falciparum_Sanger|(protein coc			
	K.ELKDQE	3.2755	0.2998	99.9	1739.862	1739.856		4050.5	1	6.017	57.7	2
	K.KKDILD	3.5245	0.3125	100	1255.713	1255.712		7180.7	1	5.805	83.3	1
PFF0655c		3	1.30%	1363	159774	6.4	U		Annotation|Plasmodium_falciparum_Sanger|(protein coc			
	R.NFFNEH	3.6616	0.3856	100	2273.199	2273.183		7798.9	1	6.515	64.7	3
PF13_0239		2	1.30%	1244	150357	6.4	U		Annotation|Plasmodium_falciparum_Sanger|(protein coc			
	K.NIEFFIP	3.3604	0.2807	99.8	2009.113	2008.11		6680.7	1	4.977	56.7	2
PFD0535w		3	1.30%	1298	150269	6.6	U		Annotation|Plasmodium_falciparum_Sanger|(protein coc			
	Q.WEKENI	3.3846	0.2868	99.9	2258.961	2257.957		9714.5	1	5.753	59.4	3
PFI1560c		1	1.30%	1247	144529	9.4	U		Annotation|Plasmodium_falciparum_Sanger|(protein coc			
	K.NIGNIN	4.7571	0.3759	100	1854.927	1854.929		6287.4	1	7.49	76.7	1
PF14_0184		3	1.30%	1166	136940	8.7	U		Annotation|Plasmodium_falciparum_TIGR|(protein codin			
	K.VFNIVH	4.012	0.3867	100	1791.934	1791.933		9655	1	6.916	78.6	3
PFL0635c		2	1.30%	1088	125450	9.1	U		Annotation|Plasmodium_falciparum_Sanger|(protein coc			
	K.HGSNNS	3.8658	0.4228	100	1591.732	1591.733		4741.8	1	6.811	69.2	2
PFL2410w		4	1.30%	1039	121331	9.2	U		Annotation|Plasmodium_falciparum_Sanger|(protein coc			
	K.SVHDCI	3.5788	0.485	100	1738.851	1738.852		7363.1	1	7.835	69.2	4
PF14_0380		3	1.30%	1014	118503	5.1	U		Annotation|Plasmodium_falciparum_TIGR|(protein codin			
	K.NNQLAL	3.9399	0.3041	100	1480.753	1480.751		5759.9	1	7.05	79.2	3
PF13_0299		1	1.30%	964	113756	5.8	U		Annotation|Plasmodium_falciparum_Sanger|(protein coc			
	K.SAELSH	3.2643	0.3224	100	1476.754	1476.752		5041.4	5	5.316	54.2	1
PF14_0511		3	1.30%	910	106987	6.9	U		Annotation|Plasmodium_falciparum_TIGR|(protein codin			
	K.NHSYHD	3.6066	0.3454	100	1484.665	1484.663		5382.6	1	6.911	72.7	3
PF10_0279		2	1.30%	838	98543	5.5	U		Annotation|Plasmodium_falciparum_TIGR|(protein codin			
	R.DNYHPN	3.3142	0.2197	99.8	1355.709	1355.707		6321.6	1	5.117	80	2
PFI1510w		6	1.30%	825	97929	8.7	U		Annotation|Plasmodium_falciparum_Sanger|(protein coc			
	R.KASHDC	3.4678	0.301	100	1300.702	1300.701		7643.2	1	6.307	85	6

PFF1110c		3	1.30%	786	91145	8.9	U	Annotation|Plasmodium_falciparum_Sanger|(protein coc			
	K.KGGEDI	2.7956	0.2861	99.8	1166.546	1166.544	5539.5	2	5.067	72.2	3
PFF0670w		9	1.20%	4095	483830	6.2	U	Annotation|Plasmodium_falciparum_Sanger|(protein coc			
	H.KEHVIN	2.6664	0.2566	99.9	903.533	902.5285	4241.6	3	4.921	91.7	2
	K.IVDNVD	3.7887	0.3134	100	1384.719	1384.719	7016.2	1	6.952	77.3	3
	K.KNNNIN	4.361	0.4248	100	2187.025	2187.022	6015.8	1	6.959	65.6	1
	K.HNEHNE	3.565	0.4307	100	1604.713	1604.709	5098.5	1	7.034	72.7	3
MAL13P1.266		1	1.20%	1606	191762	8.5	U	Annotation|Plasmodium_falciparum_Sanger|(protein coc			
	R.GGGGG	3.4215	0.3808	100	1812.812	1811.84	5504.4	26	6.389	33.3	1
PF10_0057		1	1.20%	1554	180787	7.5	U	Annotation|Plasmodium_falciparum_TIGR|(protein codin			
	R.NYSAPN	3.2047	0.3713	100	2080.1	2080.101	5710.9	1	5.492	41.7	1
PFL0950c		2	1.20%	1555	179133	7.1	U	Annotation|Plasmodium_falciparum_Sanger|(protein coc			
	R.TSSLIEE	4.1108	0.3907	100	2074.044	2072.044	6208.9	1	6.627	61.8	2
PF11_0456		2	1.20%	1464	175410	6.8	U	Annotation|Plasmodium_falciparum_TIGR|(protein codin			
	L.KMNKYT	4.0578	0.2598	99.9	2192.983	2191.964	4704.1	1	5.147	52.9	2
MAL13P1.52		3	1.20%	1385	164521	6.1	U	Annotation|Plasmodium_falciparum_Sanger|(protein coc			
	K.APHEQL	3.3341	0.3791	100	1965.918	1964.914	5052.7	1	6.079	53.1	3
PF07_0056		3	1.20%	1361	159528	5.8	U	Annotation|Plasmodium_falciparum_Sanger|(protein coc			
	K.NVTDNV	4.3858	0.3512	100	1986.888	1985.881	4805.5	1	6.877	62.5	3
PF08_0020		15	1.20%	1326	154633	6.1	U	Annotation|Plasmodium_falciparum_Sanger|(protein coc			
	K.FFNPIID	5.5271	0.2941	100	1903.057	1903.044	7359.1	1	7.124	76.7	15
PF14_0217		3	1.20%	1190	140517	5.8	U	Annotation|Plasmodium_falciparum_TIGR|(protein codin			
	K.HDISNN	5.4452	0.4275	100	1640.842	1640.841	6221.1	1	8.73	80.8	3
PFL1215c		2	1.20%	1187	138143	6.3	U	Annotation|Plasmodium_falciparum_Sanger|(protein coc			
	K.KKPSTY	3.9697	0.3935	100	1681.884	1681.883	8002.4	1	7.463	73.1	2
MAL8P1.105		2	1.20%	1133	133758	9.1	U	Annotation|Plasmodium_falciparum_Sanger|(protein coc			
	K.YVNPPF	4.152	0.2511	100	1711.936	1711.936	7747.9	1	5.869	73.1	2
PF13_0018		3	1.20%	1022	118622	6.1	U	Annotation|Plasmodium_falciparum_Sanger|(protein coc			
	K.YGYNHI	3.2818	0.3109	99.9	1496.704	1496.702	6108.9	3	5.316	68.2	3
PFI0135c		3	1.20%	932	105544	5.3	U	Annotation|Plasmodium_falciparum_Sanger|(protein coc			
	K.EHKEED	2.7818	0.2984	99.8	1430.694	1430.692	5297	1	5.471	75	3
PFL2220w		1	1.20%	888	104015	8.1	U	Annotation|Plasmodium_falciparum_Sanger|(protein coc			
	K.KKDEQI	4.4969	0.1424	99.9	1386.817	1386.814	7489.4	7	4.894	80	1
PFL1480w		1	1.20%	822	90749	6.6	U	Annotation|Plasmodium_falciparum_Sanger|(protein coc			
	K.IYQMND	3.1025	0.2226	99.8	1273.638	1273.636	7961.7	1	5.313	83.3	1
PF10_0256		2	1.20%	757	89209	8.2	U	Annotation|Plasmodium_falciparum_TIGR|(protein codin			

	K.KIENFN	2.8922	0.2803	99.9	1078.588	1078.59	5730.5	1	4.918	81.2	2
PF14_0315		6	1.10%	5561	658544	8.7	U	Annotation|Plasmodium_falciparum_TIGR|(protein codin			
	R.KVSNAC	3.5252	0.4281	100	1448.717	1448.717	6483.9	1	6.759	75	3
	K.LCHAD	5.228	0.4379	100	5441.3	5439.294	11113.7	1	6.008	15.3	3
PFL1620w		11	1.10%	5439	646349	6.4	U	Annotation|Plasmodium_falciparum_Sanger|(protein coc			
	R.VREDLE	4.0042	0.3412	100	1728.927	1727.923	6425.8	1	6.24	73.1	1
	K.RDYNHI	3.9899	0.4166	100	1948.057	1946.051	4589.6	1	6.617	56.7	3
	K.ETTYNH	3.4631	0.3876	100	1383.681	1383.677	3554.1	1	5.838	80	1
	K.YDCNNY	3.9006	0.37	100	2419.067	2418.061	6507	1	6.595	55.6	6
PFB0555c		13	1.10%	4091	494215	8.3	U	Annotation|Plasmodium_falciparum_TIGR|(protein codin			
	K.NFIDNTI	4.4103	0.4221	100	2155.168	2155.167	6538	1	6.904	58.8	9
	K.NFTSIY	3.0319	0.361	100	1918.991	1917.987	5040.9	1	5.985	53.6	1
	K.NHENVN	3.6272	0.4278	100	1617.75	1617.752	4286.3	1	6.9	61.5	3
PF10_0177		9	1.10%	3165	370536	5.8	U	Annotation|Plasmodium_falciparum_TIGR|(protein codin			
	K.NNFMEV	3.1637	0.2473	99.8	1438.608	1437.604	7180.9	1	5.144	75	3
	K.KTENLN	3.4832	0.306	100	1483.742	1483.739	5833.7	1	5.718	68.2	4
	R.KPILIP	3.0379	0.2872	99.9	1292.775	1292.769	5762.1	1	5.379	75	2
PFB0190c		25	1.10%	2295	271488	8.3	U	Annotation|Plasmodium_falciparum_TIGR|(protein codin			
	K.NNVSPF	4.3701	0.3991	100	2889.504	2888.494	6669.8	1	7.801	42	25
MAL13P1.202		3	1.10%	1936	231556	7.2	U	Annotation|Plasmodium_falciparum_Sanger|(protein coc			
	K.HQETNI	3.5938	0.3247	100	1254.654	1254.652	6603.7	1	5.685	77.8	1
	K.NNNIVH	3.1084	0.274	99.9	1292.708	1292.708	6471	1	5.713	80	2
PFA0590w		10	1.10%	1822	214439	8.7	U	Annotation|Plasmodium_falciparum_Sanger|(protein coc			
	I.PIFNLN	5.0617	0.4673	100	2286.218	2285.212	5397.2	1	8.058	52.6	10
PFB0665w		2	1.10%	1714	204288	9.4	U	Annotation|Plasmodium_falciparum_TIGR|(protein codin			
	M.NYEYLK	2.9669	0.3234	99.8	2387.052	2387.064	6648	1	5.264	44.4	2
PFE1465w		2	1.10%	1631	191619	7.2	U	Annotation|Plasmodium_falciparum_Sanger|(protein coc			
	K.VTDEND	4.2763	0.397	100	2071.884	2071.88	5701.9	1	7.279	50	2
PFL1125w		2	1.10%	1618	190445	8.6	U	Annotation|Plasmodium_falciparum_Sanger|(protein coc			
	K.SNHHHN	5.1642	0.482	100	2066.903	2065.897	6125.8	1	8.67	71.9	2
PF07_0097		4	1.10%	1422	167924	7	U	Annotation|Plasmodium_falciparum_Sanger|(protein coc			
	R.DRKPLG	4.3313	0.2547	100	1859.894	1858.89	6145.5	1	5.566	73.3	4
PFF0945c		3	1.10%	1392	163906	7	U	Annotation|Plasmodium_falciparum_Sanger|(protein coc			
	K.YCNFQY	2.8543	0.3598	99.9	1996.926	1996.927	5305.8	1	5.578	64.3	3
PF07_0035		8	1.10%	1248	146629	9	U	Annotation|Plasmodium_falciparum_Sanger|(protein coc			
	K.NHNEYT	3.5876	0.2554	99.8	1784.839	1784.84	4466.6	1	5.866	61.5	8

MAL7P1.126		1	1.10%	1222	144627	6.6	U		Annotation|Plasmodium_falciparum_Sanger|(protein coc				
	K.LKDYINI	3.7867	0.3088	100	1705.012	1705.009		8084.9	1	6.411	76.9	1	
PF10_0041		2	1.10%	1235	142944	5.1	U		Annotation|Plasmodium_falciparum_TIGR|(protein codin				
	K.SVILRPL	2.6335	0.3639	99.9	1569.904	1569.912		7829.9	1	6.617	57.7	2	
PFL2280w		1	1.10%	909	105537	6.2	U		Annotation|Plasmodium_falciparum_Sanger|(protein coc				
	K.KKEDVL	3.6081	0.2397	100	1235.72	1235.719		5841	1	4.759	88.9	1	
MAL7P1.147		32	1.00%	3183	373171	7.9	U		Annotation|Plasmodium_falciparum_Sanger|(protein coc				
	K.NLRPVN	3.8934	0.3378	100	2426.272	2425.248		5408.2	1	5.582	44.7	6	
	K.IDHFNF	3.7871	0.3523	100	1649.79	1649.79		5656.9	1	5.924	75	26	
PFF0325c		2	1.00%	2402	279071	5.2	U		Annotation|Plasmodium_falciparum_Sanger|(protein coc				
	K.NMDNIL	3.2377	0.3025	99.9	1548.732	1548.73		5451.7	2	5.661	62.5	1	
	K.YINITN	3.328	0.2994	99.9	1553.805	1553.794		5733	2	5.83	72.7	1	
PF14_0343		3	1.00%	2269	270477	8.2	U		Annotation|Plasmodium_falciparum_TIGR|(protein codin				
	K.NNNYEE	3.3163	0.3003	99.9	1507.724	1507.722		7646.8	1	5.951	63.6	1	
	R.EHDIHN	3.194	0.3096	99.9	1397.648	1397.645		5395.9	1	7.183	75	2	
PF11_0342		8	1.00%	2072	245039	5.7	U		Annotation|Plasmodium_falciparum_TIGR|(protein codin				
	R.KLSHDN	2.79	0.2546	99.8	1069.566	1069.564		6328.9	1	5.621	81.2	5	
	K.EQYRPI	3.7822	0.2726	100	1561.761	1561.761		6505.7	8	5.233	68.2	3	
MAL13P1.123		4	1.00%	1937	225986	8.2	U		Annotation|Plasmodium_falciparum_Sanger|(protein coc				
	L.QICKNK	3.8435	0.2772	99.9	2336.226	2333.204		6003.3	5	4.84	36.1	4	
PF11_0291		6	1.00%	1789	212864	6.2	U		Annotation|Plasmodium_falciparum_TIGR|(protein codin				
	R.DTCIHP	4.2317	0.4372	100	2174.205	2174.201		6084.1	1	7.625	58.8	6	
MAL8P1.157		3	1.00%	1738	205288	6.5	U		Annotation|Plasmodium_falciparum_Sanger|(protein coc				
	K.ISHHNN	6.264	0.5454	100	2003.976	2003.972		7569.4	1	10.06	78.1	3	
PFD1150c		1	1.00%	1697	203378	7.4	U		Annotation|Plasmodium_falciparum_Sanger|(protein coc				
	K.NIQQQL	4.1554	0.3717	100	2037.091	2037.092		5344.3	1	6.682	46.9	1	
PF14_0558		3	1.00%	1469	174108	6	U		Annotation|Plasmodium_falciparum_TIGR|(protein codin				
	R.NHDEKD	4.4242	0.4285	100	1675.769	1675.768		4061.3	1	8.496	88.5	3	
PF10_0322		6	1.00%	1434	168169	6.4	U		Annotation|Plasmodium_falciparum_TIGR|(protein codin				
	K.INTHTIN	4.5479	0.4234	100	1570.861	1570.86		6257.6	1	7.957	76.9	6	
PF10_0246		3	1.00%	1333	159219	8.2	U		Annotation|Plasmodium_falciparum_TIGR|(protein codin				
	K.NFILQS	4.093	0.3292	100	1587.873	1587.879		8682.6	1	6.865	83.3	3	
PFI0330c		16	1.00%	1261	153280	9.6	U		Annotation|Plasmodium_falciparum_Sanger|(protein coc				
	K.YNSTDN	3.7243	0.2688	99.9	1672.819	1672.79		6699.2	1	5.909	66.7	16	
PFF1445c		3	1.00%	1129	136343	8.2	U		Annotation|Plasmodium_falciparum_Sanger|(protein coc				
	K.FLPLLG	3.6724	0.3573	100	1302.762	1302.761		3977.5	1	6.047	90	3	

PFC0610c		2	1.00%	1181	135638	6.1	U		Annotation|Plasmodium_falciparum_Sanger|(protein coc				
	K.KTEAQE	2.8947	0.31	99.9	1300.715	1300.711		4789	20	5.573	63.6	2
PF13_0286		3	1.00%	1019	120251	9	U		Annotation|Plasmodium_falciparum_Sanger|(protein coc				
	K.HGDHQY	3.2809	0.3089	100	1241.568	1241.566		3911.4	1	6.253	77.8	3
PF07_0021		3	1.00%	989	118344	8	U		Annotation|Plasmodium_falciparum_Sanger|(protein coc				
	K.KNDYFL	3.4123	0.2284	99.9	1282.68	1282.68		7572.3	1	4.541	88.9	3
PFL2125c		2	1.00%	945	111870	5.2	U		Annotation|Plasmodium_falciparum_Sanger|(protein coc				
	M.FRENIN	2.9443	0.3662	100	1139.57	1139.585		5251.4	1	5.734	93.8	2
PF14_0538		2	0.90%	2391	283201	7.2	U		Annotation|Plasmodium_falciparum_TIGR|(protein codin				
	K.ELIHHE	4.0956	0.3331	100	1493.732	1492.726		4989.4	1	5.993	68.2	1
	K.YNNMHY	2.786	0.3156	99.9	1122.586	1122.584		4747.2	1	5.983	81.2	1
PF14_0690		2	0.90%	2251	268975	9	U		Annotation|Plasmodium_falciparum_TIGR|(protein codin				
	K.IDPIILP	3.633	0.2767	99.9	2512.372	2509.335		4650.5	1	5.667	57.9	2
PFD0735c		5	0.90%	2212	261820	8.5	U		Annotation|Plasmodium_falciparum_Sanger|(protein coc				
	K.KENVDL	3.1113	0.2415	99.8	1255.629	1255.628		4825.8	1	5.68	75	2
	K.LNHHDY	2.7525	0.3939	100	1349.601	1349.599		4358.5	1	6.824	77.8	3
MAL13P1.262		1	0.90%	2006	240018	6	U		Annotation|Plasmodium_falciparum_Sanger|(protein coc				
	Y.FCVNKI	4.0077	0.2227	99.8	2334.191	2332.201		9150.9	6	4.597	47.2	1
PFD0385w		34	0.90%	1960	230123	6.9	U		Annotation|Plasmodium_falciparum_Sanger|(protein coc				
	K.DNFDEN	4.9781	0.4427	100	2260.115	2257.104		6024.5	1	7.604	67.6	34
PF07_0010		3	0.90%	1843	220455	8	U		Annotation|Plasmodium_falciparum_Sanger|(protein coc				
	K.NEFHDF	4.0782	0.3282	100	2016.007	2015.003		6707.3	1	6.374	59.4	3
PFL1865w		2	0.90%	1812	212760	5.1	U		Annotation|Plasmodium_falciparum_Sanger|(protein coc				
	K.FLIIIKT	4.9146	0.2525	100	1943.179	1943.17		8677.6	1	5.656	55	2
PF11_0185		6	0.90%	1509	180154	9	U		Annotation|Plasmodium_falciparum_TIGR|(protein codin				
	K.RKDEKE	3.9437	0.2267	99.9	1761.879	1760.868		6197.7	1	6.103	83.3	6
PFE0430w		5	0.90%	1490	175807	8.8	U		Annotation|Plasmodium_falciparum_Sanger|(protein coc				
	K.VKGNNI	3.9939	0.254	99.9	1578.838	1578.835		7228.4	1	7.31	73.1	5
MAL13P1.260		1	0.90%	1318	151517	8.8	U		Annotation|Plasmodium_falciparum_Sanger|(protein coc				
	K.IIDVPVP	2.9937	0.3333	99.9	1317.863	1317.87		5055.5	1	5.761	63.6	1
PFF0535c		1	0.90%	1280	149366	6.8	U		Annotation|Plasmodium_falciparum_Sanger|(protein coc				
	K.NYVTHI	3.4504	0.3915	100	1438.737	1438.74		6040.9	1	6.817	81.8	1
PFF0165c		1	0.90%	1103	132470	5.7	U		Annotation|Plasmodium_falciparum_Sanger|(protein coc				
	R.NVEEEL	2.8286	0.3361	99.9	1225.621	1225.618		7985.3	1	5.623	77.8	1
PFD0540c		6	0.90%	977	117528	6.6	U		Annotation|Plasmodium_falciparum_Sanger|(protein coc				
	K.NDNLHV	3.1245	0.2511	99.9	1064.533	1063.528		4108.7	1	5.987	87.5	6

MAL13P1.86		2	0.90%	896	104782	6.6	U		Annotation|Plasmodium_falciparum_Sanger|(protein coc				
	K.YNHIIIG	2.9148	0.2532	99.9	957.5527	957.5522		4054.7	1	4.99	92.9	2
PFB0405w		1	0.80%	3135	363219	5.5	U		Annotation|Plasmodium_falciparum_TIGR|(protein codin				
	S.VKLVNG	4.3844	0.2036	99.9	2923.552	2919.508		9632.5	1	4.295	33.3	1
PF07_0024		5	0.80%	2814	330702	7.4	U		Annotation|Plasmodium_falciparum_Sanger|(protein coc				
	K.KHLNIP	3.9756	0.3631	100	1533.815	1533.814		6360.5	1	6.792	70.8	3
	K.HNYINE	2.9931	0.2556	99.8	1259.65	1258.643		7250.9	1	5.904	83.3	2
PF13_0273		2	0.80%	2533	304306	6.7	U		Annotation|Plasmodium_falciparum_Sanger|(protein coc				
	K.TNVDDI	3.5078	0.3787	100	2338.273	2338.271		5684	1	5.904	44.7	2
PF13_0072		1	0.80%	2361	277697	7.8	U		Annotation|Plasmodium_falciparum_Sanger|(protein coc				
	D.KDDDE	4.0084	0.2136	99.9	2453.958	2454.003		9621.3	3	4.99	47.4	1
PFC0165w		1	0.80%	2226	267976	7.5	U		Annotation|Plasmodium_falciparum_Sanger|(protein coc				
	R.YLNYNE	3.4097	0.2713	99.9	2066.903	2066.939		6125.8	2	4.858	46.9	1
PFI0830c		3	0.80%	1826	215349	6	U		Annotation|Plasmodium_falciparum_Sanger|(protein coc				
	K.EDTHLD	3.9514	0.3526	100	1827.831	1827.826		5382.2	1	7.074	75	3
MAL8P1.104		7	0.80%	1774	200213	7.4	U		Annotation|Plasmodium_falciparum_Sanger|(protein coc				
	K.GINHTN	4.1899	0.3208	100	1664.829	1664.836		6910.3	1	6.144	64.3	7
PF11_0327		3	0.80%	1576	184693	6.2	U		Annotation|Plasmodium_falciparum_TIGR|(protein codin				
	K.VHNTNN	4.3171	0.4202	100	1430.715	1428.706		5439.6	1	7.77	86.4	3
MAL13P1.226		1	0.80%	1498	177556	5.8	U		Annotation|Plasmodium_falciparum_Sanger|(protein coc				
	K.KGGEDI	3.381	0.3539	100	1329.699	1329.699		5282.9	1	6.461	77.3	1
MAL8P1.123		3	0.80%	1421	171290	8.2	U		Annotation|Plasmodium_falciparum_Sanger|(protein coc				
	K.KKNDEQ	3.6629	0.3687	100	1498.75	1498.744		5797.1	6	6.058	59.1	3
PF10_0032		3	0.80%	1425	170850	8.8	U		Annotation|Plasmodium_falciparum_TIGR|(protein codin				
	N.ISYLHNI	3.3596	0.2038	99.8	1335.767	1335.775		8433	2	4.387	70	3
PFL0445w		1	0.80%	1442	166863	6.5	U		Annotation|Plasmodium_falciparum_Sanger|(protein coc				
	K.SHNSTF	2.7971	0.2844	99.8	1143.625	1143.624		5915	1	6.012	75	1
PFB0125c		2	0.80%	1353	160247	4.5	U		Annotation|Plasmodium_falciparum_TIGR|(protein codin				
	K.IKDKYDI	3.3404	0.286	99.9	1427.708	1427.706		5904.1	1	5.936	90	2
PF13_0067		1	0.80%	1333	159297	8.1	U		Annotation|Plasmodium_falciparum_Sanger|(protein coc				
	K.VHINNLY	3.3263	0.2645	99.9	1272.715	1272.714		5717.7	1	6.134	85	1
PFI0455w		4	0.80%	1311	154959	7	U		Annotation|Plasmodium_falciparum_Sanger|(protein coc				
	K.IYVSSN	3.6167	0.1988	99.9	1252.688	1251.681		6178.1	1	5.733	80	4
PF11_0477		1	0.80%	1301	150982	4.8	U		Annotation|Plasmodium_falciparum_TIGR|(protein codin				
	R.ISNKNE	3.4239	0.2076	99.8	1331.703	1331.699		5582.2	1	5.255	80	1
PFF0295c		3	0.80%	1063	123332	5.9	U		Annotation|Plasmodium_falciparum_Sanger|(protein coc				

	R.NHVLLD	2.5539	0.3515	99.9	1090.565	1090.565	4928.5		1	6.192	81.2	3
MAL13P1.249		1	0.70%	3381	405413	7.9	U	Annotation|Plasmodium_falciparum_Sanger|(protein coc				
	K.NEFINE	3.3681	0.3012	99.9	2757.354	2756.355	5862.1		1	5.382	38.1	1
PFE0440w		4	0.70%	3134	370183	5.4	U	Annotation|Plasmodium_falciparum_Sanger|(protein coc				
	K.KNEIYI	3.7837	0.388	100	1582.825	1582.834	7169.7		1	5.774	75	2
	I.MNISSNL	3.3743	0.1775	99.8	1021.517	1021.499	7387.7		21	4.076	81.2	2
PFL1900w		11	0.70%	2577	299381	6.3	U	Annotation|Plasmodium_falciparum_Sanger|(protein coc				
	K.VCTLIN	4.7926	0.2843	100	2065.011	2065.007	5921		1	7.279	68.8	11
PF13_0072		1	0.70%	2361	277697	7.8	U	Annotation|Plasmodium_falciparum_Sanger|(protein coc				
	K.ISSFHS	3.6174	0.3754	100	1989.934	1989.917	5156.8		2	7.153	46.7	1
PF11_0242		15	0.70%	2265	265135	6	U	Annotation|Plasmodium_falciparum_TIGR|(protein codin				
	K.ETFLQF	3.7819	0.3479	100	1937.999	1936.996	6679.4		1	6.186	66.7	15
PFE0765w		4	0.70%	2133	255919	9.2	U	Annotation|Plasmodium_falciparum_Sanger|(protein coc				
	R.NHEHYF	3.0909	0.3669	100	1681.78	1681.78	4148.3		1	5.713	61.5	4
MAL13P1.1		1	0.70%	2162	247399	5.3	U	Annotation|Plasmodium_falciparum_Sanger|(protein coc				
PF07_0051		1	0.70%	2215	251675	5.5	U	Annotation|Plasmodium_falciparum_Sanger|(protein coc				
	P.YEDLLL	4.8718	0.1531	99.9	1864.89	1864.87	11826.7		2	5.382	67.9	1
PFF0490w		1	0.70%	2017	244671	6.5	U	Annotation|Plasmodium_falciparum_Sanger|(protein coc				
	K.KINSEHI	3.1822	0.2803	99.8	1850.021	1850.029	7753.7		1	4.85	53.6	1
PFL1315w		1	0.70%	1979	233834	9.4	U	Annotation|Plasmodium_falciparum_Sanger|(protein coc				
	I.IIIGIIILA	1.3995	0.7306	100	1402.112	1398.075	5060.3		1	3.399	41.7	1
PFL1800w		4	0.70%	1915	230847	9	U	Annotation|Plasmodium_falciparum_Sanger|(protein coc				
	R.NYQHID	4.3078	0.4159	100	1791.85	1788.813	3189.3		1	7.877	73.1	4
MAL13P1.123		2	0.70%	1937	225986	8.2	U	Annotation|Plasmodium_falciparum_Sanger|(protein coc				
	K.YINHINT	4.1844	0.1488	99.8	1634.856	1634.854	8714.7		1	5.341	83.3	2
PF13_0162		1	0.70%	1844	218437	8.1	U	Annotation|Plasmodium_falciparum_Sanger|(protein coc				
	K.KKTLEN	4.7413	0.1027	99.9	1464.804	1462.791	10238.5		19	3.594	68.2	1
PFB0375w		2	0.70%	1802	216824	9	U	Annotation|Plasmodium_falciparum_TIGR|(protein codin				
	L.KERKIN	3.4438	0.257	99.8	1661.975	1660.95	6122.4		2	5.164	50	2
PF11_0206		6	0.70%	1749	208588	6	U	Annotation|Plasmodium_falciparum_TIGR|(protein codin				
	K.GKVENY	3.7661	0.3003	100	1502.717	1501.711	7969.6		1	6.642	77.3	6
PFI0225w		4	0.70%	1770	207567	6.2	U	Annotation|Plasmodium_falciparum_Sanger|(protein coc				
	K.NLFSLF	3.3776	0.2689	99.9	1618.845	1618.834	7669.5		4	6.452	54.2	4
PF13_0053		1	0.70%	1672	198334	7.7	U	Annotation|Plasmodium_falciparum_Sanger|(protein coc				
	K.HNEHNE	3.3277	0.2341	99.9	1363.636	1363.633	5148.4		1	6.557	80	1
MAL7P1.204		6	0.70%	1706	196569	8.1	U	Annotation|Plasmodium_falciparum_Sanger|(protein coc				

	K.SNVLVP	3.3712	0.2754	99.9	1356.719	1356.728		3833.8	2	5.815	68.2	6
PF11_0252		9	0.70%	1653	195835	7.7	U	Annotation|Plasmodium_falciparum_TIGR|(protein codin				
	R.RCPFPY	3.555	0.2353	99.9	1486.683	1485.68		7623.7	1	5.422	86.4	9
MAL13P1.124		1	0.70%	1510	177676	8.2	U	Annotation|Plasmodium_falciparum_Sanger|(protein coc				
	K.NKGDIN	3.172	0.2121	99.8	1152.614	1152.613		4356.3	1	5.202	77.8	1
PFD0250c		1	0.70%	1362	157258	6.3	U	Annotation|Plasmodium_falciparum_Sanger|(protein coc				
	K.STIHNF	2.6548	0.3035	99.8	1124.581	1124.581		6624.4	14	5.358	66.7	1
PF08_0126		2	0.70%	1239	143045	5.7	U	Annotation|Plasmodium_falciparum_Sanger|(protein coc				
	K.VAHVDS	2.6635	0.3653	99.9	982.5332	982.5322		4068.6	1	6.363	87.5	2
PFI0490c		1	0.70%	1195	139026	6.6	U	Annotation|Plasmodium_falciparum_Sanger|(protein coc				
	K.ILFNLNN	2.8505	0.2954	99.9	1010.587	1010.586		3910.3	1	5.339	78.6	1
PF07_0086		3	0.60%	3429	416634	8.5	U	Annotation|Plasmodium_falciparum_Sanger|(protein coc				
	K.NQENYH	3.5259	0.3573	100	2730.148	2729.147		4319.9	1	5.783	42.5	3
MAL13P1.176		7	0.60%	3179	374192	5.3	U	Annotation|Plasmodium_falciparum_Sanger|(protein coc				
PF13_0198		7	0.60%	3130	370438	5.6	U	Annotation|Plasmodium_falciparum_Sanger|(protein coc				
	I.NSKSKFI	4.5957	0.2073	99.8	2192.129	2192.124		10616.5	1	5.37	41.2	7
MAL13P1.351		12	0.60%	2825	341429	7.2	U	Annotation|Plasmodium_falciparum_Sanger|(protein coc				
	R.VTYDTL	4.7555	0.4048	100	2095.085	2095.083		7211.4	1	7.417	67.6	12
MAL7P1.167		3	0.60%	2773	329465	9.2	U	Annotation|Plasmodium_falciparum_Sanger|(protein coc				
	K.KGNSN	3.0308	0.3124	99.8	1764.788	1764.782		4483.3	1	5.742	56.7	3
PFC0805w		2	0.60%	2457	278676	5.6	U	Annotation|Plasmodium_falciparum_Sanger|(protein coc				
	K.FKNENN	4.9375	0.4034	100	1731.89	1730.886		6871.5	1	6.864	84.6	2
PFL1980c		15	0.60%	2309	276530	8.2	U	Annotation|Plasmodium_falciparum_Sanger|(protein coc				
	K.NVVSNL	3.4936	0.4054	100	1528.753	1528.751		7607.8	1	7.171	70.8	3
	K.NVVSNL	4.5609	0.3822	100	1656.847	1656.846		5468.1	1	7.711	69.2	12
PFB0095c		18	0.60%	2441	273646	8.9	U	Annotation|Plasmodium_falciparum_TIGR|(protein codin				
	K.AHENLE	4.7497	0.4115	100	1775.81	1775.809		5955.5	1	9.637	78.6	18
PFL1010c		6	0.60%	2329	271069	6.1	U	Annotation|Plasmodium_falciparum_Sanger|(protein coc				
	K.KKEEDI	4.7407	0.3603	100	1657.849	1657.852		7093.8	1	7.162	79.2	6
MAL7P1.55		1	0.60%	2239	256544	5.8	U	Annotation|Plasmodium_falciparum_Sanger|(protein coc				
	R.GPQIGS	3.6934	0.4431	100	1340.67	1340.667		7373.5	1	7.002	70.8	1
PFB0145c		2	0.60%	1979	237745	6	U	Annotation|Plasmodium_falciparum_TIGR|(protein codin				
	K.INTLNEC	3.3838	0.2496	99.9	1346.68	1346.678		4631.6	1	5.236	80	2
PF07_0115		3	0.60%	1918	225075	8.5	U	Annotation|Plasmodium_falciparum_Sanger|(protein coc				
	R.HNDVHH	5.1062	0.342	100	1559.701	1558.694		7273.2	1	8.529	90.9	3
PFI0295c		1	0.60%	1807	215074	8.8	U	Annotation|Plasmodium_falciparum_Sanger|(protein coc				

<table>
<tr><th></th><th></th><th></th><th></th><th></th><th></th><th></th><th></th><th></th><th></th><th></th><th></th><th></th></tr>
<tr><td></td><td>L.KNVDIEI</td><td>3.8603</td><td>0.1402</td><td>99.8</td><td>1365.736</td><td>1363.747</td><td></td><td>7639.1</td><td>12</td><td>3.556</td><td>80</td><td>1</td></tr>
<tr><td>PF14_0152</td><td></td><td>3</td><td>0.60%</td><td>1474</td><td>178501</td><td>8.8</td><td>U</td><td colspan="5">Annotation|Plasmodium_falciparum_TIGR|(protein codin</td></tr>
<tr><td></td><td>K.VEDNKL</td><td>3.2286</td><td>0.2144</td><td>99.9</td><td>1131.565</td><td>1131.565</td><td></td><td>6313.2</td><td>6</td><td>5.246</td><td>87.5</td><td>3</td></tr>
<tr><td>PFE0570w</td><td></td><td>8</td><td>0.50%</td><td>10061</td><td>1187631</td><td>7.5</td><td>U</td><td colspan="5">Annotation|Plasmodium_falciparum_Sanger|(protein coc</td></tr>
<tr><td></td><td>K.SRDIEN</td><td>4.4388</td><td>0.3024</td><td>100</td><td>1582.778</td><td>1582.776</td><td></td><td>4947.8</td><td>1</td><td>5.96</td><td>79.2</td><td>3</td></tr>
<tr><td></td><td>K.LYAHFQ</td><td>3.6664</td><td>0.2779</td><td>99.9</td><td>2124.097</td><td>2123.097</td><td></td><td>6959.9</td><td>1</td><td>5.218</td><td>62.5</td><td>3</td></tr>
<tr><td></td><td>R.NLNILH(</td><td>3.6067</td><td>0.3284</td><td>100</td><td>1936.016</td><td>1936.019</td><td></td><td>5229.9</td><td>1</td><td>6.783</td><td>66.7</td><td>2</td></tr>
<tr><td>PFB0560w</td><td></td><td>11</td><td>0.50%</td><td>3990</td><td>477857</td><td>8.3</td><td>U</td><td colspan="5">Annotation|Plasmodium_falciparum_TIGR|(protein codin</td></tr>
<tr><td></td><td>R.FSELFD</td><td>5.0273</td><td>0.4719</td><td>100</td><td>2384.151</td><td>2382.147</td><td></td><td>9437.8</td><td>1</td><td>8.854</td><td>57.9</td><td>11</td></tr>
<tr><td>PFD0320c</td><td></td><td>4</td><td>0.50%</td><td>3358</td><td>401567</td><td>8.5</td><td>U</td><td colspan="5">Annotation|Plasmodium_falciparum_Sanger|(protein coc</td></tr>
<tr><td></td><td>S.FTSKVN</td><td>4.4151</td><td>0.2357</td><td>99.9</td><td>2138.212</td><td>2136.221</td><td></td><td>9535.5</td><td>1</td><td>6.028</td><td>55.9</td><td>4</td></tr>
<tr><td>PFE0655w</td><td></td><td>12</td><td>0.50%</td><td>2763</td><td>332134</td><td>9.1</td><td>U</td><td colspan="5">Annotation|Plasmodium_falciparum_Sanger|(protein coc</td></tr>
<tr><td></td><td>R.FLPAHT</td><td>3.556</td><td>0.3364</td><td>100</td><td>1724.879</td><td>1723.877</td><td></td><td>4715.9</td><td>1</td><td>5.487</td><td>71.4</td><td>12</td></tr>
<tr><td>PF11_0418</td><td></td><td>10</td><td>0.50%</td><td>2275</td><td>267963</td><td>5</td><td>U</td><td colspan="5">Annotation|Plasmodium_falciparum_TIGR|(protein codin</td></tr>
<tr><td></td><td>E.LKEEEL</td><td>4.5399</td><td>0.1097</td><td>99.9</td><td>1516.815</td><td>1516.811</td><td></td><td>7520.6</td><td>7</td><td>3.985</td><td>77.3</td><td>10</td></tr>
<tr><td>PFB0540w</td><td></td><td>3</td><td>0.50%</td><td>1844</td><td>214679</td><td>7.3</td><td>U</td><td colspan="5">Annotation|Plasmodium_falciparum_TIGR|(protein codin</td></tr>
<tr><td></td><td>K.SPNNIT</td><td>3.2363</td><td>0.3686</td><td>100</td><td>1129.644</td><td>1129.644</td><td></td><td>8666.3</td><td>1</td><td>5.979</td><td>83.3</td><td>3</td></tr>
<tr><td>PFL1530w</td><td></td><td>5</td><td>0.50%</td><td>1824</td><td>212658</td><td>6.5</td><td>U</td><td colspan="5">Annotation|Plasmodium_falciparum_Sanger|(protein coc</td></tr>
<tr><td></td><td>K.HSYNNE</td><td>3.2484</td><td>0.2057</td><td>99.8</td><td>1280.604</td><td>1280.602</td><td></td><td>3896.8</td><td>1</td><td>7.366</td><td>88.9</td><td>5</td></tr>
<tr><td>MAL8P1.23</td><td></td><td>13</td><td>0.40%</td><td>8591</td><td>1005178</td><td>5.3</td><td>U</td><td colspan="5">Annotation|Plasmodium_falciparum_Sanger|(protein coc</td></tr>
<tr><td></td><td>R.KREEYF</td><td>3.365</td><td>0.2229</td><td>99.8</td><td>1517.751</td><td>1517.75</td><td></td><td>8053.4</td><td>1</td><td>5.101</td><td>80</td><td>4</td></tr>
<tr><td></td><td>K.VKEQHI</td><td>3.4097</td><td>0.2603</td><td>99.9</td><td>1289.679</td><td>1289.672</td><td></td><td>6961.4</td><td>1</td><td>6.149</td><td>83.3</td><td>3</td></tr>
<tr><td></td><td>K.LPQTHN</td><td>2.7468</td><td>0.3337</td><td>99.9</td><td>1224.618</td><td>1224.616</td><td></td><td>4945.4</td><td>1</td><td>5.588</td><td>72.2</td><td>6</td></tr>
<tr><td>PFD0872w</td><td></td><td>1</td><td>0.40%</td><td>4830</td><td>576662</td><td>8.5</td><td>U</td><td colspan="5">Annotation|Plasmodium_falciparum_Sanger|(protein coc</td></tr>
<tr><td></td><td>K.YYEDKV</td><td>4.0657</td><td>0.1828</td><td>99.8</td><td>2108.055</td><td>2106.066</td><td></td><td>8759.1</td><td>1</td><td>4.556</td><td>53.1</td><td>1</td></tr>
<tr><td>PFI1500w</td><td></td><td>1</td><td>0.40%</td><td>4530</td><td>542004</td><td>8.8</td><td>U</td><td colspan="5">Annotation|Plasmodium_falciparum_Sanger|(protein coc</td></tr>
<tr><td></td><td>S.LNFVCV</td><td>3.9551</td><td>0.2516</td><td>99.9</td><td>2114.192</td><td>2111.224</td><td></td><td>8178.2</td><td>1</td><td>5.358</td><td>58.8</td><td>1</td></tr>
<tr><td>PF10_0212</td><td></td><td>7</td><td>0.40%</td><td>4450</td><td>527597</td><td>6.3</td><td>U</td><td colspan="5">Annotation|Plasmodium_falciparum_TIGR|(protein codin</td></tr>
<tr><td></td><td>K.FINVVNY</td><td>3.4272</td><td>0.2114</td><td>99.9</td><td>1123.671</td><td>1123.67</td><td></td><td>3632.6</td><td>1</td><td>4.842</td><td>87.5</td><td>4</td></tr>
<tr><td></td><td>K.GKDLHE</td><td>3.0538</td><td>0.2759</td><td>99.9</td><td>1087.555</td><td>1087.554</td><td></td><td>5975.3</td><td>1</td><td>5.79</td><td>87.5</td><td>3</td></tr>
<tr><td>PF10_0079</td><td></td><td>20</td><td>0.40%</td><td>3853</td><td>453008</td><td>8.5</td><td>U</td><td colspan="5">Annotation|Plasmodium_falciparum_TIGR|(protein codin</td></tr>
<tr><td></td><td>R.LLINHS</td><td>4.1558</td><td>0.4126</td><td>100</td><td>1860.018</td><td>1859.014</td><td></td><td>6552.4</td><td>1</td><td>6.402</td><td>63.3</td><td>20</td></tr>
<tr><td>PF14_0407</td><td></td><td>6</td><td>0.40%</td><td>3384</td><td>404585</td><td>6.5</td><td>U</td><td colspan="5">Annotation|Plasmodium_falciparum_TIGR|(protein codin</td></tr>
<tr><td></td><td>K.SGFHTT</td><td>3.3618</td><td>0.3387</td><td>100</td><td>1390.637</td><td>1390.635</td><td></td><td>5256.9</td><td>1</td><td>5.99</td><td>70.8</td><td>6</td></tr>
<tr><td>PFC0760c</td><td></td><td>7</td><td>0.40%</td><td>3394</td><td>402951</td><td>4.8</td><td>U</td><td colspan="5">Annotation|Plasmodium_falciparum_Sanger|(protein coc</td></tr>
<tr><td></td><td>K.NNSLFS</td><td>4.0509</td><td>0.2819</td><td>100</td><td>1776.948</td><td>1776.947</td><td></td><td>7829.5</td><td>1</td><td>6.426</td><td>64.3</td><td>7</td></tr>
</table>

PFD0320c		3	0.40%	3358	401567	8.5	U		Annotation|Plasmodium_falciparum_Sanger|(protein coc				
	K.KENMTE	4.5488	0.1881	99.9	1740.783	1740.778		7862.8	1	7.228	73.1	3	
PFI0975c		3	0.40%	3381	396154	6.9	U		Annotation|Plasmodium_falciparum_Sanger|(protein coc				
	K.EITNNH	3.4216	0.3705	100	1750.853	1749.848		3518.4	1	5.803	64.3	3	
PFL1600c		4	0.40%	3193	377926	7.8	U		Annotation|Plasmodium_falciparum_Sanger|(protein coc				
	I.SVNNYS	3.3768	0.2716	99.9	1348.638	1346.623		9618.4	1	5.279	68.2	4	
PFF1370w		3	0.40%	3072	364333	9	U		Annotation|Plasmodium_falciparum_Sanger|(protein coc				
	R.HDNMNI	4.1633	0.3419	100	1454.727	1454.725		6184.8	1	7.372	77.3	3	
PF07_0042		2	0.40%	2910	350702	5.8	U		Annotation|Plasmodium_falciparum_Sanger|(protein coc				
	K.IKDTQQ	3.7067	0.2071	99.9	1430.787	1430.786		8303.5	4	5.299	72.7	2	
PFF0745c		2	0.40%	2747	325600	9.3	U		Annotation|Plasmodium_falciparum_Sanger|(protein coc				
	K.NVNHIQ	4.0186	0.4064	100	1467.732	1467.731		5784.4	1	7.311	77.3	2	
PF14_0282		1	0.40%	2657	306807	7	U		Annotation|Plasmodium_falciparum_TIGR|(protein codin				
	K.HGDNLI	3.1958	0.2151	99.8	1125.576	1124.57		6829.4	1	5.551	88.9	1	
PFA0215w		1	0.40%	2359	281507	7.1	U		Annotation|Plasmodium_falciparum_Sanger|(protein coc				
	K.MIQSHV	3.1705	0.2487	99.9	1197.661	1197.66		7597.9	2	5.086	77.8	1	
PFD0585c		3	0.40%	1928	229692	7.1	U		Annotation|Plasmodium_falciparum_Sanger|(protein coc				
	K.CNVHDH	3.0858	0.3453	100	1056.469	1056.469		3864.5	1	6.032	78.6	3	
MAL13P1.39		2	0.30%	6088	730383	9	U		Annotation|Plasmodium_falciparum_Sanger|(protein coc				
	E.NNKQVN	3.5746	0.2323	99.8	2429.184	2429.213		5900	1	4.923	44.7	2	
PFF0445w		1	0.30%	6077	720533	7	U		Annotation|Plasmodium_falciparum_Sanger|(protein coc				
	R.IDQHILN	5.1635	0.4577	100	2324.273	2323.271		6083.7	1	8.067	66.7	1	
MAL7P1.17		1	0.30%	3559	425269	8.8	U		Annotation|Plasmodium_falciparum_Sanger|(protein coc				
	N.EINKHI	3.5893	0.1721	99.8	1324.739	1324.723		6105	1	3.889	80	1	
PF11_0049		3	0.30%	3371	391942	7	U		Annotation|Plasmodium_falciparum_TIGR|(protein codin				
	K.SLDNHS	3.039	0.3145	99.9	1347.651	1346.645		5322.5	1	5.99	75	3	
PFF0575c		1	0.30%	3248	374892	5.8	U		Annotation|Plasmodium_falciparum_Sanger|(protein coc				
	K.VISCNE	2.8948	0.3642	100	1353.669	1353.67		5796	1	5.897	75	1	
MAL8P1.139		6	0.20%	5910	705835	8.6	U		Annotation|Plasmodium_falciparum_Sanger|(protein coc				
	R.LNHEQN	3.8322	0.3202	100	1596.808	1595.803		5355.5	1	5.458	79.2	6	
PF10_0374		6	0.10%	9563	1099303	3.6	U		Annotation|Plasmodium_falciparum_TIGR|(protein codin				
	I.EEVVEEL	4.2985	0.2361	99.9	1673.836	1671.858		6069.1	1	5.24	69.2	6	
PF14_0326		3	0.10%	8094	969749	7.9	U		Annotation|Plasmodium_falciparum_TIGR|(protein codin				
	K.SKQNNI	3.6747	0.1891	99.8	1401.745	1401.745		4606.5	1	5.43	81.8	3	
PF14_0084		2	0.10%	7149	847912	8.7	U		Annotation|Plasmodium_falciparum_TIGR|(protein codin				
	K.IVCGHY	2.9432	0.3114	99.9	1155.569	1155.57		5237.2	2	6.022	66.7	2	

ding) 60S Acidic ribosomal protein P2 Location= chr3 404654..404992

ding) phosphoethanolamine N-methyltransferase, putative Location= chr13 join(1696925..1696991, 1697137..1697167, 1697339..1697421, 169752[illegible]

ıg) glyceraldehyde-3-phosphate dehydrogenase Location= chr14 join(2559033..2559116, 2559353..2560282)

ling) uridine phosphorylase, putative Location= chr5 complement(569180..569917)

ling) L-lactate dehydrogenase Location= chr13 1041358..1042308

ıg) hypoxanthine phosphoribosyltransferase Location= chr10 complement(join(476245..476338, 476546..476562, 475326..475910))

ıg) adenosine deaminase, putative Location= chr10 1206300..1207403

ıg) triose-phosphate isomerase Location= chr14 join(1622519..1622630, 1622921..1623555)

ding) macrophage migration inhibitory factor homolog, putative Location= chr12 join(1208901..1208979, 1209260..1209531)

ng) endoplasmic reticulum-resident calcium binding protein Location= chr11 complement(369675..370706)

g) phosphoglycerate mutase, putative Location= chr11 join(761694..761727, 761883..761964, 762181..762817)

ling) hypothetical protein Location= chr13 complement(join(1895157..1895693, 1894314..1894895))

ıg) 60S acidic ribosomal protein p1, putative Location= chr11 complement(join(140603..140802, 140049..140205))

ling) Phosphoglycerate kinase Location= chr9 913220..914470

ding) histamine-releasing factor, putative Location= chr5 complement(467583..468098)

ıg) enolase Location= chr10 join(637137..637179, 637713..639010)

ling) Heat shock protein Location= chr9 join(737975..738050, 738384..740266)

ling) ornithine aminotransferase Location= chr6 362631..363875

ling) disulfide isomerase precursor, putative Location= chr8 complement(join(1209291..1209466, 1207860..1209135))

ıg) calmodulin Location= chr14 complement(join(1369661..1369739, 1368758..1369128))

ding) heat shock 70 kDa protein Location= chr8 861481..863514

ling) guanine nucleotide-binding protein, putative Location= chr8 complement(join(1162651..1162780, 1161236..1162077))

ıg) peptidyl-prolyl cis-trans isomerase Location= chr11 complement(join(590382..590459, 590617..590628, 589706..590203))

ling) 14-3-3 protein homologue, putative Location= chr8 join(831139..831223, 831519..831604, 831747..832346)

ıg) ribosomal phosphoprotein P0 Location= chr11 1165219..1166169

ıg) HAP protein Location= chr14 297468..298823

ling) u6 snRNA-associated Sm-like protein, putative Location= chr12 join(427266..427380, 427480..427551, 427715..427785, 427904..427972)

ling) early transcribed membrane protein Location= chr5 1301219..1301764

ling) ubiquitin-like protein, putative Location= chr5 complement(join(252420..252606, 252047..252162))

ling) elongation factor 1 alpha Location= chr13 complement(2262150..2263481)
ling) elongation factor 1 alpha Location= chr13 2265234..2266565

[illegible]ling) PFCYP19, cyclophilin, peptidyl-prolyl cis-trans isomerase Location= chr3 complement(925315..925830)

ıg) RNA helicase-1, putative Location= chr14 join(2825306..2825632, 2825755..2826473, 2826567..2826699, 2826837..2826854)

ıg) centrin, putative Location= chr14 complement(join(1917510..1917664, 1917830..1917953, 1917175..1917402))

ıg) elongation factor 2 Location= chr14 2090839..2093337

ling) 1-cys peroxidoxin Location= chr8 166536..167198

ling) actin depolymerizing factor, putative Location= chr5 join(140710..140917, 141311..141471)

ling) pyruvate kinase, putative Location= chr6 join(1078996..1079179, 1079471..1080188, 1080688..1081321)

ling) beta3 proteasome subunit, putative Location= chr1 complement(join(329762..329856, 329987..330457, 329584..329632))

ling) heat shock protein 86 Location= chr7 join(436972..438402, 439188..439994)

ling) glutaredoxin, putative Location= chr3 complement(join(292056..292117, 292240..292306, 292444..292507, 292690..292769, 291782..291844)

ling) conserved protein Location= chr9 join(1286998..1287081, 1287421..1287534, 1287670..1287928, 1288304..1288362)

g) peptidase, putative Location= chr14 2234118..2236412

ıg) hypothetical protein, conserved Location= chr14 join(1533272..1533337, 1533682..1534890)

ding) pyridoxine biosynthetic enzyme pdx1 homologue, putative Location= chr6 complement(871294..872199)

ling) nascent polypeptide associated complex alpha chain, putative Location= chr6 join(891995..892304, 892431..892538, 892639..892775)

ling) hypothetical protein Location= chr13 complement(468434..468865)

ıg) circumsporozoite-related antigen Location= chr11 complement(join(815567..815886, 816201..816321, 815385..815432))

ding) 4-methyl-5(B-hydroxyethyl)-thiazol monophosphate biosynthesis enzyme Location= chr6 complement(join(1102581..1102638, 1102821..11028

ng) serine/threonine protein phosphatase, putative Location= chr14 join(575495..575759, 575865..576188, 576397..576548, 576744..576878, 5770

ıg) 40S ribosomal protein, putative Location= chr10 join(1122960..1123022, 1123307..1124035)

ıg) deoxyuridine 5'-triphosphate nucleotidohydrolase, putative Location= chr11 complement(1060260..1060781)

ıg) thioredoxin Location= chr14 complement(join(2344127..2344154, 2343576..2343862))

ıg) ribosomal protein S8e, putative Location= chr14 join(323317..323651, 323851..324172)

ıg) 2-Cys peroxiredoxin Location= chr14 1575927..1576514

ling) rhoptry protein, putative Location= chr9 complement(join(271553..272323, 272533..272589, 272780..272842, 273044..274004, 274464..27457

ling) 40S ribosomal protein S12, putative Location= chr3 complement(join(309936..310016, 309367..309711))

ding) elongation factor 1 (EF-1), putative Location= chr3 join(814583..814710, 814866..815016, 815174..815365)

ng) high mobility group-like protein NHP2, putative Location= chr11 join(945469..945643, 945812..945915, 945989..946147)

ng) rhoptry-associated protein 1 Location= chr14 420442..422790

ıg) hypothetical protein Location= chr11 join(251058..251264, 251400..251509, 251656..251771, 251897..251939, 252097..252248, 252381..2524[illegible]

ling) s-adenosylmethionine synthetase, putative Location= chr9 903352..904560

ling) eukaryotic initiation factor 5a, putative Location= chr12 complement(205488..205973)

ling) histone h2a Location= chr6 complement(748413..748811)

ling) actin Location= chr12 1920770..1921900

ling) hypothetical protein Location= chr9 join(1175193..1176381, 1176487..1176903, 1177030..1177092, 1177203..1177271, 1177442..1177948, 1[illegible]

ling) m1-family aminopeptidase Location= chr13 501992..505249

ling) Nucleosome assembly protein Location= chr9 complement(join(782244..782296, 782613..782665, 781358..782061))

ling) rabGDI protein Location= chr12 complement(join(1806721..1806813, 1807000..1807060, 1807326..1807343, 1805367..1806574))

ling) endoplasmin homolog precursor, putative Location= chr12 complement(883558..886023)

ding) Serine hydroxymethyltransferase Location= chr12 join(1484293..1484295, 1484414..1484515, 1484675..1485898)

ding) EF-1B Location= chr9 575670..576500

ng) histone H2B Location= chr11 226066..226419

ding) centrin, putative Location= chr1 join(293860..293983, 294178..294245, 294468..294554, 294651..294731, 294928..294987, 295100..295186)

ng) histone H4, putative Location= chr11 complement(221819..222130)

ding) hypothetical protein, conserved Location= chr13 complement(join(2036958..2037069, 2037213..2037286, 2037421..2037484, 2037610..2037[illegible]

ng) hypothetical protein Location= chr10 join(1495972..1496074, 1496240..1496360, 1496564..1496657)

ding) myosin A tail domain interacting protein MTIP, putative Location= chr12 1931956..1932570

ding) 10 kd chaperonin, putative Location= chr12 complement(join(629311..629409, 629002..629178))

ling) nucleosome assembly protein 1, putative Location= chr12 complement(join(188260..188353, 188816..188839, 187143..188104))

ling) 40S ribosomal subunit protein S14, putative Location= chr5 complement(join(676021..676198, 676441..676612, 675807..675912))

ling) merozoite surface protein 1, precursor Location= chr9 1201802..1206964

ıg) hypothetical protein Location= chr10 complement(join(262465..262627, 262947..262997, 262150..262259))

ıg) GTP-binding nuclear protein ran/tc4 Location= chr11 complement(join(671397..671514, 670710..671236))

ıg) hypothetical protein Location= chr10 join(277621..277638, 278072..278209, 278353..278431, 278663..279168)

ling) ribosomal protein S19s, putative Location= chr4 join(1031088..1031090, 1031330..1031839)

ig) plasmepsin 2 Location= chr14 293471..294832

ig) hypothetical protein Location= chr14 join(2206963..2206967, 2207434..2208256)

ling) hypothetical protein Location= chr6 complement(join(765376..765378, 764664..765260))

ig) tubulin beta chain, putative Location= chr10 complement(join(361082..362034, 362385..362480, 360625..360913))

ling) translation initiation factor 6, putative Location= chr13 1348977..1349720

ling) karyopherin beta Location= chr5 998748..1002119

ng) ADP-ribosylation factor Location= chr10 complement(join(845293..845443, 845592..845679, 845810..845880, 846044..846191, 845085..845172

ding) hypothetical protein Location= chr9 join(1040703..1040795, 1040946..1041506)

ıg) hypothetical protein, conserved Location= chr14 1092878..1093723

ling) Mature parasite-infected erythrocyte surface antigen (MESA) or PfEMP2 Location= chr5 complement(join(47612..47761, 43327..47481))

ling) Cg4 protein Location= chr7 451091..453712

ling) Fe-superoxide dismutase Location= chr8 709349..709945

ling) myo-inositol 1-phosphate synthase, putative Location= chr5 complement(join(521653..522691, 520718..521493))

ıg) 26S protease subunit regulatory subunit 6a, putative Location= chr11 1168877..1170196

ling) chromatin assembly factor 1 protein WD40 domain, putative Location= chr1 complement(414914..416254)

ling) 40S ribosomal protein S5, putative Location= chr7 complement(join(987465..987542, 986837..987346))

ling) histone H2A variant, putative Location= chr3 join(871883..871976, 872372..872623, 872835..872965)

ng) ribosomal protein S2, putative Location= chr14 join(1929874..1929880, 1930230..1930308, 1930450..1931035, 1931176..1931322)

ıg) merozoite surface protein 2 precursor Location= pfal_chr2 complement(273689..274507)

ding) BIS(5'-nucleosyl)-tetraphosphatase (diadenosine tetraphosphatase), putative Location= chr5 complement(join(843867..843976, 844105..8442[illegible]

ding) hypothetical protein Location= chr8 join(657400..657439, 657814..658751)

ding) ran binding protein 1 Location= chr4 join(877717..877785, 878080..878853)

ıg) Sec61-gamma subunit of protein translocation complex, putative Location= pfal_chr2 409983..410228

ding) rhoptry-associated protein 2, RAP2 Location= chr5 complement(84041..85237)

ıg) casein kinase II, alpha subunit, putative Location= chr11 365093..366100

ling) hypothetical protein, conserved in other Plasmodium species Location= chr8 complement(join(886794..887233, 886295..886688))

ıg) hypothetical protein Location= chr11 1253071..1253892

ling) Rab18 GTPase, putative Location= chr8 join(382685..382748, 382984..383049, 383280..383418, 383632..383968)

ling) hypothetical protein Location= chr8 join(424318..424328, 424884..425658)

ıg) small nuclear ribonucleoprotein F, putative Location= chr11 complement(join(1054061..1054151, 1054261..1054275, 1053812..1053918))

ıg) casein kinase II beta chain, putative Location= chr11 160116..160853

ling) glutamate--ammonia ligase (glutamine synthetase), putative Location= chr9 917189..918475

ling) tubulin-specific chaperone a, putative Location= chr1 complement(join(376112..376240, 375592..375963))

ling) hypothetical protein Location= chr7 394422..396779

ling) small GTP-binding protein sar1 Location= chr4 join(736944..736960, 737135..737196, 737706..738205)

g) cytochrome c, putative Location= chr14 complement(join(145547..145649, 145134..145378))

ling) elongation factor 1-gamma, putative Location= chr13 join(1549043..1549120, 1549365..1550591)

ling) histone h2b, putative Location= chr7 complement(698480..698851)

ling) Cytoadherence linked asexual protein, CLAG Location= chr3 join(132097..132952, 133049..133624, 133741..133794, 133866..133928, 13407

ling) hypothetical protein Location= chr7 join(772659..774091, 774211..774346)

ling) chaperonin, putative Location= chr13 join(1355483..1355566, 1355705..1355765, 1355952..1356070, 1356163..1356230, 1356674..1356740,

ling) Translation initiation factor SUI1, putative Location= chr12 join(1825946..1826045, 1826400..1826500, 1826725..1826871)

ling) 60S ribosomal subunit protein L6e, putative Location= chr13 complement(1545265..1545930)

ling) hypothetical protein Location= chr9 join(1458380..1458484, 1458804..1459850)

ling) hypothetical protein Location= chr7 join(993657..993807, 993943..994005, 994167..994273)

ling) histone H3, putative Location= chr6 444742..445152

ling) protein kinase c inhibitor-like protein, putative Location= chr8 join(800563..800629, 800765..800817, 800930..801064, 801194..801331)

ling) cell division cycle protein 48 homologue, putative Location= chr6 complement(join(817218..817804, 817944..818244, 815619..817151))

ıg) hypothetical protein, conserved Location= chr10 1339760..1340626

ling) 40S ribosomal protein S15, putative Location= chr13 join(740905..740913, 741221..741655)

ıg) spermidine synthase Location= chr11 join(1121928..1122143, 1122323..1122793, 1122923..1123043, 1123218..1123375)

ling) 60S ribosomal protein L27a, putative Location= chr6 join(768789..768953, 769172..769453)

ling) glutaredoxin-like protein, putative Location= chr6 complement(290422..291081)

g) deoxyribose-phosphate aldolase, putative Location= chr10 871093..871884

ling) hypothetical protein, conserved Location= chr9 complement(join(752155..752412, 751348..752052))

ling) vacuolar ATP synthetase, putative Location= chr5 complement(join(791914..792034, 792209..792288, 792418..792576, 792865..792934, 791

g) ribosomal protein L30e, putative Location= chr10 join(786502..786625, 786805..787007)

ling) GTPase, putative Location= chr5 join(544538..544551, 544789..544861, 545124..545219, 545310..545373, 545505..545580, 545712..545991

ling) Bet3 transport protein, putative Location= chr4 complement(join(838203..838286, 838492..838674, 838904..839003, 839100..839197, 839349

ling) hypothetical protein Location= chr7 join(1134393..1134443, 1134778..1134821, 1135049..1135084, 1135235..1135433)

g) heat shock protein hsp70 homologue Location= chr11 1320112..1322103

ling) T-complex protein eta subunit, putative Location= chr3 complement(join(360894..360908, 359017..360621))

ıg) exported protein 2 Location= chr14 complement(join(2905919..2906093, 2906367..2906503, 2905101..2905652))

ıg) plasmepsin, putative Location= chr14 283086..284435

ding) 40S ribosomal protein S17, putative Location= chr12 join(1803640..1803642, 1804060..1804470)

ding) 60S ribosomal subunit protein L31, putative Location= chr5 complement(join(151823..151977, 152375..152384, 151422..151619))

ling) hypothetical protein, conserved Location= chr13 join(2469863..2469878, 2470158..2477959)

[illegible]ling) RNA lariat debranching enzyme, putative Location= chr13 complement(1618196..1619923)

ıg) ribosomal protein L3, putative Location= chr10 complement(1153269..1154429)

ding) membrane associated histidine-rich protein, MAHRP-1 Location= chr13 join(2784306..2784698, 2784888..2785247)

ding) hexokinase Location= chr6 981067..982548

ding) hsp70 interacting protein, putative Location= chr5 join(1143612..1143630, 1143996..1145353)

ng) translocation protein sec62, putative Location= chr14 complement(1541570..1542577)

ding) small heat shock protein, putative Location= chr13 complement(236002..236637)

ling) RNA-binding protein, putative Location= chr8 complement(join(1024168..1024611, 1024859..1024918, 1023701..1024057))

ling) thioredoxin-related protein, putative Location= chr13 complement(join(2090533..2090668, 2089696..2090186))

ling) F49C12.11-like protein Location= chr3 complement(join(965577..965674, 965857..965874, 965347..965395))

ling) hypothetical protein Location= chr5 join(64065..64133, 64332..65489)

ling) skeleton binding protein Location= chr5 join(68930..69652, 69823..70113)

ıg) Proteosome subunit alpha type 1, putative Location= chr14 join(3067497..3067545, 3067658..3067740, 3067995..3068627)

ling) nucleoside diphosphate kinase b; putative Location= chr13 2657908..2658357

ıg) cysteine protease, putative Location= pfal_chr2 complement(join(305700..305840, 306016..306846, 306994..307027, 303593..305580))

ıg) adenylate kinase, putative Location= chr10 complement(join(370103..370225, 369199..369804))

ling) 6-phosphofructokinase, putative Location= chr9 complement(650576..654832)

ling) arsenical pump-driving ATPase, putative Location= chr4 complement(675561..676700)

ng) hypothetical protein, conserved Location= chr14 join(1828603..1828735, 1829056..1829669, 1829855..1829905)

ling) 60S ribosomal subunit porotein L18, putative Location= chr13 join(1636877..1636882, 1636935..1637190, 1637560..1637864)

ng) ribosomal protein S18, putative Location= chr11 complement(join(1024292..1024495, 1023789..1024055))

ling) 40S ribosomal protein S23, putative Location= chr3 join(307326..307335, 307539..307717, 308231..308479)

ıg) glucose-6-phosphate isomerase Location= chr14 1458074..1459813

ling) hypothetical protein Location= chr5 join(55841..56038, 56288..56872)

ling) hypothetical protein, conserved Location= chr3 join(366600..366710, 366945..367167, 367376..367464)

ıg) ribosomal protein L1, putative Location= chr14 join(1678605..1678609, 1678781..1679429)

ıg) hypothetical protein Location= chr14 join(3139075..3139176, 3139301..3140917)

ıg) NADP-specific glutamate dehydrogenase Location= chr14 667411..668823

ıg) ribonucleotide reductase small subunit Location= chr14 complement(193216..194265)

ıg) eukaryotic translation initiation factor 2 gamma subunit, putative Location= chr14 427191..428588

ıg) protein disulfide isomerase related protein Location= chr11 complement(1323041..1324312)

ıg) hypothetical protein Location= chr14 170000..170893

ling) eukaryotic translation initiation factor, putative Location= chr8 complement(join(731192..731303, 731475..731542, 731708..731764, 731900..7

ling) hypothetical protein Location= chr13 join(1845859..1846100, 1846305..1846597, 1846748..1846848)

ling) Skp1 family protein, putative Location= chr13 complement(join(2675926..2676066, 2676166..2676261, 2676416..2676558, 2676657..2676759

ling) acid phosphatase, putative Location= chr9 complement(742484..743674)

ding) hypothetical protein Location= chr13 complement(2465460..2465873)

ng) hypothetical protein, conserved Location= chr14 complement(2346114..2348033)

ng) ethanolamine kinase, putative Location= chr11 complement(970104..971375)

ng) hypothetical protein, conserved Location= chr11 complement(join(522168..522302, 522445..522540, 522679..522823, 521121..521911))

ling) proliferating cell nuclear antigen Location= chr13 complement(2478805..2479629)

ling) 60S ribosomal protein L23, putative Location= chr13 join(1324210..1324335, 1324707..1325000)

ling) cyclophilin, putative Location= chr13 complement(join(857696..857842, 858010..858145, 857482..857570))

ling) hypothetical protein Location= chr7 971812..972546

ling) Chromatin assembly protein (ASF1), putative Location= chr12 join(998344..998601, 998703..999266)

ig) T-complex protein 1, putative Location= pfal_chr2 566320..567894

ig) Ribosomal protein family L5, putative Location= chr14 complement(join(974234..975015, 975413..975485, 973795..973824))

ling) 60S ribosomal protein L12, putative Location= chr5 complement(join(716637..716673, 715763..716223))

ıg) small nuclear ribonuclear protein, putative Location= chr14 join(1775953..1776013, 1776217..1776296, 1776441..1776605)

ling) hypothetical protein Location= chr3 join(563358..563406, 563489..563518, 563601..563629, 563753..563795, 563991..564057, 564257..5643

ling) hypothetical protein, conserved Location= chr8 join(577418..577492, 577548..578810, 579019..579137, 579301..579490)

ling) ubiquitin-conjugating enzyme, putative Location= chr5 complement(join(1130274..1130368, 1130483..1130569, 1130695..1130749, 1129954..

ling) Rab1 protein Location= chr5 complement(585355..585978)

ling) sexual stage-specific protein precursor Location= chr4 335693..336166

ling) plasmodium falciparum gamete antigen 27/25 Location= chr13 complement(121840..122493)

ling) Merozoite Surface Protein 7 precursor, MSP7 Location= chr13 complement(1419284..1420339)

ig) hypothetical protein Location= chr14 1479720..1482701

ıg) hypothetical protein, conserved Location= chr14 1410082..1410840

ıg) hypothetical protein Location= chr14 complement(join(62801..63003, 63194..63224, 61365..62612))

ıg) QF122 antigen Location= chr10 455397..458816

ling) adenosylhomocysteinase(S-adenosyl-L-homocyste ine hydrolase) Location= chr5 857035..858474

ig) leucine aminopeptidase, putative Location= chr14 complement(1893143..1894960)

ıg) 26S proteasome regulatory subunit 4, putative Location= chr10 complement(347206..348573)

ıg) hypothetical protein Location= chr11 complement(join(1056687..1056791, 1056928..1057046, 1057203..1057280, 1057417..1057488, 1058001.

ling) histone h3 Location= chr6 754392..754802

ling) transketolase, putative Location= chr6 453666..455684

ng) acyl carrier protein, putative Location= pfal_chr2 join(352836..352881, 353086..353306, 353482..353628)

ling) ubiquitin conjugating enzyme E2, putative Location= chr9 complement(join(857809..857945, 858043..858087, 858202..858294, 858502..8586

ling) hypothetical protein Location= chr8 complement(join(663058..663093, 661275..662972))

ling) alpha tubulin Location= chr9 join(175540..175601, 175946..176066, 176262..177440)

ling) 6-pyruvoyl tetrahydropterin synthase, putative Location= chr6 1121691..1122212

ling) 26s proteasome aaa-ATPase subunit Rpt3, putative Location= chr4 complement(join(625244..625333, 623897..624985))

ling) hypothetical protein Location= chr12 complement(join(2119021..2120265, 2120472..2120719, 2120862..2121263, 2121416..2121541, 212167

ıg) hypothetical protein Location= chr14 complement(2433297..2434319)

ding) 60S ribosomal protein L13, putative Location= chr8 join(682078..682317, 682523..682930)

ling) vacuolar ATP synthase subunit b Location= chr4 complement(join(331590..331698, 329739..331114))

ıg) rab6 Location= chr11 complement(join(1778345..1778374, 1777522..1778022))

ıg) merozoite capping protein 1 Location= chr10 complement(join(1141900..1142144, 1142582..1142600, 1140587..1141504))

ıg) hypothetical protein Location= chr10 complement(689887..690819)

ling) 40S ribosomal protein S10, putative Location= chr7 join(916961..917045, 917392..917553, 917699..917865)

ıg) Aquaglyceroporin Location= chr11 1271202..1271978

ling) ubiquitin-conjugating enzyme E2, putative Location= chr3 complement(join(275963..276232, 276495..276503, 275631..275780))

ling) hypothetical protein Location= chr7 1001741..1003990

ding) u6 snRNA-associated sm-like protein, putative Location= chr13 complement(1043056..1043289)

ding) hypothetical protein Location= chr8 complement(join(587984..588073, 584103..587645))

ng) hypothetical protein Location= chr11 1125950..1127308

ding) conserved GTP-binding protein, putative Location= chr7 join(1010162..1010211, 1010410..1010456, 1010637..1011721)

ling) cAMP-dependent protein kinase regulatory subunit, putative Location= chr12 complement(join(929026..929330, 929963..929982, 927816..928

ling) hypothetical protein Location= chr13 complement(join(2121406..2121603, 2120605..2121264))

ling) 60S ribosomal protein L7, putative Location= chr3 complement(join(312537..312662, 312905..312910, 311650..312291))

ig) dipeptidyl aminopeptidase 1 Location= chr11 complement(630778..632880)

ling) adenylosuccinate synthetase Location= chr13 join(2170679..2171759, 2171883..2172130)

ling) phosphoribosylpyrophosphate synthetase Location= chr13 complement(1044753..1046066)

ling) proteasome subunit alpha type 5, putative Location= chr7 join(1218088..1218101, 1218344..1218425, 1218535..1219209)

ling) proteasome component C8, putative Location= chr3 complement(join(679914..680358, 679468..679781))

ling) integral membrane protein, conserved in P. falciparum Location= chr4 complement(1075873..1076283)

ıg) hypothetical protein, conserved Location= chr14 1372846..1374540

ıg) actin II Location= chr14 join(507360..507807, 508186..508868)

ıg) ribosomal protein L7a, putative Location= chr14 join(977850..977856, 978148..978452, 978572..979108)

ling) rhoptry-associated protein 3, RAP3 Location= chr5 complement(81134..82336)

ling) proteasome beta-subunit Location= chr8 complement(join(235462..235546, 234587..235299))

ling) prefoldin subunit 3, putative Location= chr7 join(879823..879888, 880059..880129, 880505..880616, 880709..880813, 880894..880983, 88112

ıg) liver stage antigen 3 Location= pfal_chr2 join(796750..796947, 797106..801584)

ling) hypothetical protein, conserved Location= chr12 complement(join(1873354..1873406, 1873520..1873653, 1873789..1873961, 1874076..18741

ling) hypothetical protein Location= chr4 join(485925..485976, 486185..487049, 487302..487365)

ling) 40S ribosomal protein S7 homologue, putative Location= chr13 complement(join(151419..151837, 150985..151150))

ling) orotate phosphoribosyltransferase, putative Location= chr5 complement(546732..547577)

ıg) aspartate aminotransferase, putative Location= pfal_chr2 complement(198353..199570)

ıg) ribosomal protein S4, putative Location= chr11 complement(join(236471..236536, 235598..236380))

ling) T-complex protein beta subunit, putative Location= chr3 complement(302782..304380)

ling) hypothetical protein Location= chr4 complement(273709..274845)

ng) centrin, putative Location= chr10 complement(join(1150930..1150973, 1151059..1151461, 1151669..1151851, 1150661..1150780))

ling) ras family GTP-ase, putative Location= chr9 complement(join(133929..134077, 134203..134277, 134402..134548, 134705..134803, 133761..1

ling) hypothetical protein Location= chr1 join(272693..272754, 272904..273001, 273096..273181, 273417..273641)

ling) 40S ribosomal protein S13 Location= chr13 2327407..2327862

ling) hypothetical protein Location= chr4 complement(join(111958..112053, 110192..111778))

ling) hypothetical protein Location= chr13 join(1294813..1294882, 1295444..1297164)

ling) hypothetical protein, conserved Location= chr6 1075019..1076137

ng) hypothetical protein Location= pfal_chr2 359142..359849

ng) S-antigen Location= chr10 1394836..1396593

ng) hypothetical protein Location= chr14 complement(join(430056..430861, 429690..429888))

ng) hypothetical protein Location= pfal_chr2 join(557048..557338, 557523..557696)

ng) hypothetical protein Location= chr11 661999..662385

ling) Thioredoxin reductase Location= chr9 complement(961923..963548)

ling) small GTPase Rab11 Location= chr13 join(847291..847330, 847504..847552, 847691..848115, 848289..848371, 848531..848584)

ıg) hypothetical protein Location= chr14 complement(join(367018..367027, 366484..366725))

ıg) glutathione S-transferase Location= chr14 join(802716..802753, 802883..803480)

ling) hypothetical protein Location= chr1 350323..350862

ling) hypothetical protein Location= chr3 join(289618..289655, 289765..289882, 289997..290143, 290272..290425, 290631..291223)

ling) hypothetical protein Location= chr12 complement(join(249572..249904, 250119..250277, 250381..250448, 250576..250700, 250877..250991,

ling) proteasome subunit, putative Location= chr13 complement(join(2148176..2148706, 2147735..2147944))

ling) putative hsp protein Location= chr13 1769159..1771954

ling) t-complex protein 1, gamma subunit, putative Location= chr12 join(1211777..1211863, 1212011..1213552)

ling) polyadenylate-binding protein, putative Location= chr12 988619..991246

g) asparagine -- tRNA ligase, putative Location= pfal_chr2 475242..477074

ling) hypothetical protein Location= chr3 857483..858772

ng) hypothetical protein Location= chr11 join(196768..197756, 198071..198356)

ng) hypothetical protein Location= chr10 join(422960..423081, 423230..423281, 423417..423477, 423636..423757, 423875..424020, 424103..4242[illegible]

ng) hypothetical protein Location= chr14 complement(join(553829..553861, 553973..554103, 554269..554350, 554486..554590, 554738..554786, 5[illegible]

ling) u6 snRNA-associated sm-like protein Lsm2, putative Location= chr5 join(838425..838427, 838549..838713, 838937..839080)

ng) hypothetical protein Location= chr14 228410..229837

ıg) glutathione reductase Location= chr14 complement(join(823936..824014, 824130..824197, 822418..823773))

ding) hypothetical protein Location= chr8 complement(120155..123190)

ding) small nuclear ribonucleoprotein (snRNP), putative Location= chr9 complement(1366558..1367139)

ıg) ribosomal protein L21e, putative Location= chr14 complement(1016075..1016560)

ding) BolA-like protein, conserved Location= chr5 complement(658421..658675)

ıg) hsp60 Location= chr10 complement(join(628898..629039, 627043..628643))

ding) proteasome subunit, putative Location= chr13 join(2150495..2150752, 2150914..2151240, 2151321..2151410, 2151500..2151550)

ng) cysteine protease, putative Location= pfal_chr2 complement(join(301166..301300, 301403..301793, 298897..301052))

ng) nuclear transport factor 2, putative Location= chr14 complement(join(491688..491950, 492142..492221, 491478..491509))

ding) proteasome subunit alpha, putative Location= chr8 join(388339..388414, 388594..389016, 389129..389484)

ng) 6-phosphogluconate dehydrogenase, decarboxylating, putative Location= chr14 2241369..2242775

ng) plasmepsin 1 precursor Location= chr14 288297..289655

ding) bifunctional dihydrofolate reductase-thymidylate synthase Location= chr4 755069..756895

ding) splicing factor, putative Location= chr5 complement(join(725308..725749, 724679..725049))

ding) 40S ribosomal protein S11, putative Location= chr3 join(721686..721709, 722158..722528, 722711..722801)

ng) hypothetical protein Location= chr10 1181003..1182499

ng) helicase, putative Location= pfal_chr2 complement(join(406082..406355, 404281..405380))

ling) hypothetical protein Location= chr5 1071243..1072145

ling) hypothetical protein, conserved Location= chr6 758606..760993

ng) hypothetical protein Location= chr10 complement(1591577..1592143)
ng) hypothetical protein Location= pfal_chr2 complement(join(879181..879249, 878466..879086))
ling) hypothetical protein, conserved in P. falciparum Location= chr6 complement(join(1319048..1319119, 1318330..1318956))
ng) hypothetical protein Location= chr11 join(85629..85697, 85792..86418)

ng) hypothetical protein Location= chr14 complement(join(685661..685734, 685960..686083, 686228..686367, 685436..685541))

ling) conserved protein, putative Location= chr9 803019..803747

ıg) hypothetical protein Location= chr14 2473694..2474140

ıg) methionine -- tRNA ligase, putative Location= chr10 1383447..1386116

ding) hypothetical protein Location= chr12 complement(join(78993..79067, 76846..78732))

ding) importin alpha, putative Location= chr8 complement(622925..624562)

ıg) small nuclear ribonucleoprotein D1, putative Location= chr11 complement(1001181..1001561)

ding) ubiquitin-activating enzyme e1, putative Location= chr12 join(1045985..1046008, 1046269..1049395, 1049550..1049821)

ng) Protein phosphatase 2C Location= chr11 join(1518305..1518346, 1518461..1521235)

ding) Merozoite Surface Protein 9, MSP-9 Location= chr12 complement(1160719..1162950)

ding) aminopeptidase, putative Location= chr9 complement(1289305..1291017)

ling) eukaryotic translation initiation factor 3 37.28 kDa subunit, putative Location= chr7 complement(join(788521..788566, 788785..788874, 78744[illegible]

ling) hypothetical protein, conserved Location= chr9 complement(join(552433..553158, 551417..552031))

ng) merozoite surface protein 6 Location= chr10 1407273..1408388

ling) hypothetical protein Location= chr6 complement(288373..289272)

ling) branched-chain alpha keto-acid dehydrogenase, putative Location= chr13 530178..531467

ling) hypothetical protein, conserved Location= chr6 complement(join(446258..446358, 445756..446026))

ng) proteasome 26S regulatory subunit, putative Location= pfal_chr2 join(235237..235257, 235535..238219, 238482..238605, 238737..238816)

ling) vacuolar ATP synthase, catalytic subunit a Location= chr13 508102..509937

ling) Inosine-5'-monophosphate dehydrogenase Location= chr9 complement(852515..854047)

ıg) hypothetical protein Location= chr14 complement(53412..53735)

ıg) ubiquinol-cytochrome c reductase hinge protein, putative Location= chr14 complement(1049992..1050258)

ıg) pre-mRNA splicing factor, putative Location= chr10 complement(join(941389..941475, 941910..941992, 942131..942232, 939936..941280))

ling) 60S ribosomal subunit protein L18, putative Location= chr13 complement(join(1634526..1634561, 1633891..1634409))

ling) ribosomal protein L6 homologue, putative Location= chr13 complement(join(966436..966481, 965687..966213))

ng) hypothetical protein Location= chr11 complement(699868..700788)

ling) 40S ribosomal protein S3A, putative Location= chr3 complement(join(963012..963174, 962225..962850))

ling) high mobility group protein Location= chr12 complement(162426..162719)

ling) hypothetical protein Location= chr8 complement(join(402393..402482, 400713..402068))

ling) ADP-ribosylation factor GTPase-activating protein Location= chr12 complement(1868521..1869519)

ling) organelle processing peptidase, putative Location= chr9 complement(1331339..1332793)

ling) ubiquitin-conjugating enzyme, putative Location= chr13 join(2228216..2228263, 2228414..2228587, 2228735..2228885, 2228983..2229052, 2[illegible]

ling) tat-binding protein homolog Location= chr12 complement(2002709..2004016)

ling) transporter, putative Location= chr3 complement(join(666159..666265, 666485..667089, 667355..667547, 665976..666000))

ling) hypothetical protein Location= chr1 364471..365145

ling) high mobility group protein, putative Location= chr8 complement(817929..818228)

ig) dihydroorotase, putative Location= chr14 2972915..2973991

ling) 60S ribosomal protein L26, putative Location= chr3 join(530916..530931, 531342..531454, 531664..531915)

ling) 26S proteasome regulatory subunit, putative Location= chr13 complement(297380..298561)

ling) transmembrane protein, putative Location= chr5 join(1123605..1123785, 1123896..1123973, 1124079..1124320, 1124447..1124590)

ling) hypothetical protein Location= chr7 join(199310..199315, 199757..199855, 199961..200050, 200239..200333, 200424..200517, 200632..2006[illegible]

ıg) vesicle-associated membrane protein, putative Location= chr14 join(1618762..1619260, 1619511..1619737)

ling) conserved protein, putative Location= chr3 369307..370905

ıg) falcipain-3 Location= chr11 complement(584327..585805)

ling) hypothetical protein Location= chr5 complement(join(254634..254702, 254801..254885, 255010..255059, 255271..255384, 255485..255517, 2

ling) hypothetical protein, conserved Location= chr12 join(968971..969672, 969945..970064)

ling) hypothetical protein Location= chr6 join(717047..717151, 717317..717719, 717943..718695, 718835..718914)

ding) proteasome subunit beta type 1 Location= chr5 complement(join(760334..760463, 760577..760684, 760884..760996, 759829..760260))

ding) hypothetical protein Location= chr7 join(1359725..1359904, 1360050..1360190, 1360319..1360474)

ng) knob associated histidine-rich protein Location= pfal_chr2 complement(join(105686..105796, 103385..105238))

ding) glycogen synthase kinase, putative Location= chr3 complement(join(521222..521268, 521392..521481, 521597..521685, 521797..521886, 522

ng) helicase, truncated, putative Location= chr14 join(1885386..1886408, 1886865..1886900)

ding) pyrroline carboxylate reductase Location= chr13 join(2291970..2291988, 2292156..2292295, 2292384..2292458, 2292609..2292674, 2292847

ng) 20S proteasome beta 4 subunit, putative Location= chr14 join(2897854..2898237, 2898335..2898595)

ding) hypothetical protein, conserved Location= chr8 complement(518542..518814)

ıg) transcription factor, putative Location= chr10 join(1223373..1223388, 1223525..1223579, 1223718..1223817, 1223935..1224165)

ling) lysine--tRNA ligase Location= chr13 complement(join(2004079..2004281, 2002293..2003841))

ling) RNA binding function, putative Location= chr9 join(1168500..1168544, 1168756..1169032, 1169187..1169311, 1169442..1169567)

ling) phosphoesterase, putative Location= chr12 complement(270297..271211)

ling) hypothetical protein Location= chr9 630648..632198

ling) UMP-CMP kinase, putative Location= chr1 complement(join(441102..441545, 441707..441796, 441916..442003, 442250..442570, 440804..44

ling) cAMP-dependent protein kinase catalytic subunit Location= chr9 join(1361924..1362050, 1362235..1362392, 1362633..1362817, 1362955..13

ding) PfGLP-1, 1-cys-glutaredoxin-like protein-1 Location= chr3 complement(join(216105..216203, 216334..216546, 215791..215994))

ng) hypothetical protein Location= chr14 join(3202516..3202548, 3203161..3203952)

ding) chromodomain protein Location= chr12 complement(831245..832045)

ng) proliferation-associated protein 2g4, putative Location= chr14 complement(join(1106345..1106456, 1105142..1106163))

ng) ubiquinol cytochrome c oxidoreductase, putative Location= chr14 complement(1600876..1601943)

ding) isoleucine--tRNA ligase, putative Location= chr13 complement(1350240..1354058)

ng) methyltransferase, putative Location= chr11 complement(1063668..1064549)

ıg) ribosomal protein L10, putative Location= chr14 570802..571461

ling) nuclear movement protein, putative Location= chr13 1486652..1487812

ling) Casein kinase II regulatory subunit, putative Location= chr13 complement(join(1667711..1668184, 1666863..1667546))

ling) 70 kDa peptidylprolyl isomerase, putative Location= chr12 complement(1955110..1956024)

ling) cytoadherence linked asexual protein (CLAG) Location= chr9 join(1413829..1414603, 1414954..1415556, 1415665..1415718, 1415863..1415[illegible]

ling) hypothetical protein, conserved Location= chr8 join(1253421..1253576, 1253730..1253831, 1253935..1254027, 1254124..1254214, 1254390..

ıg) Ribosomal protein, 40S subunit, putative Location= chr11 join(1755435..1755548, 1755798..1755932)

ling) TCP-1/cpn60 chaperonin family, putative Location= chr13 join(2288507..2288547, 2288733..2288835, 2289025..2290467)

ling) hypothetical protein, conserved Location= chr8 688461..689207

ling) RNA binding protein, putative Location= chr6 217237..219504

ling) hypothetical protein, conserved Location= chr12 complement(join(1011278..1011333, 1010823..1011087))

ling) cop-coated vesicle membrane protein p24 precursor, putative Location= chr13 complement(join(623210..623380, 623557..623674, 623772..62

ling) hypothetical protein, conserved Location= chr3 complement(283581..283994)

ng) dynamin-like protein Location= chr11 complement(join(1800732..1800778, 1800926..1801062, 1801245..1803547, 1800541..1800567))

ng) hypothetical protein Location= chr10 complement(join(473178..473385, 472545..472915))

ng) hypothetical protein Location= pfal_chr2 complement(join(162697..162786, 163223..163253, 162193..162374))

ling) T-complex protein 1 epsilon subunit, putative Location= chr3 842383..843990

ling) proteasome regulatory subunit, putative Location= chr13 complement(join(2706912..2707674, 2707909..2708052, 2706740..2706768))

ling) calcyclin binding protein, putative Location= chr12 complement(join(1583912..1584583, 1583611..1583709))

ng) hypothetical protein Location= chr14 complement(365509..365907)

ling) hypothetical protein Location= chr8 join(966895..966963, 967425..967730)

ling) dynein light chain 1, putative Location= chr12 586802..587083

ling) replication factor A-related protein, putative Location= chr9 join(222269..222282, 222714..224154)

ling) S1/P1nuclease, putative Location= chr9 complement(370444..371547)

ıg) hypothetical protein Location= chr11 complement(596288..597202)

ıg) early transcribed membrane protein 11.2 Location= chr11 complement(131704..131988)

ling) hypothetical protein Location= chr9 complement(join(1422608..1422796, 1420472..1422424))

ıg) heat shock protein DnaJ homologue Pfj2 Location= chr11 complement(373238..374860)

ling) RESA-like protein, truncated Location= chr4 complement(join(1117927..1118115, 1116974..1117714))

ıg) hypothetical protein Location= chr10 join(1505834..1505870, 1506288..1506427, 1506546..1506613, 1506778..1506895)

ling) hypothetical protein Location= chr13 join(2126864..2127145, 2127267..2127398)

ling) hypothetical protein Location= chr5 complement(1224591..1224884)

ıg) hypothetical protein Location= pfal_chr2 join(610087..610150, 610872..612646, 612764..612889, 613019..613126, 613250..613319, 613453..61

ıg) ATP synthase (C/AC39) subunit, putative Location= chr14 complement(2625387..2626535)

ling) single-strand binding protein, putative Location= chr5 complement(363588..364442)

ling) hypothetical protein Location= chr7 complement(join(677089..677216, 677331..677376, 677691..677728, 677866..677941, 678006..678083, 6

ling) Bi-functional aminoacyl-tRNA synthetase, putative Location= chr12 complement(590187..592427)

ling) hypothetical protein Location= chr12 join(113787..113893, 114020..114190, 114311..114849, 115173..115247, 115347..115443, 115572..115

ling) ubiquitin-conjugating enzyme e2, putative Location= chr12 join(194141..194164, 194626..194729, 194897..194966, 195096..195243, 195362..

ling) vesicle transport protein, putative Location= chr13 join(2446794..2449315, 2449385..2449470, 2449581..2449701, 2449842..2449915, 24500

ıg) hypothetical protein, conserved Location= chr14 2981537..2982901

ıg) ribosomal S27a, putative Location= chr14 complement(join(93183..93268, 93435..93537, 92264..92524))

ıg) hypothetical protein Location= chr14 complement(join(1876008..1876360, 1876655..1876817, 1875364..1875903))

ling) hypothetical protein Location= chr5 complement(328666..329715)

ling) small nuclear ribonucleoprotein (snRNP), putative Location= chr9 join(445779..445911, 445971..446041, 446147..446320)

ling) hypothetical protein Location= chr8 1044885..1046852

ling) cutA, putative Location= chr12 complement(join(2017611..2017643, 2017803..2017901, 2018005..2018097, 2018243..2018455, 2017350..201

ling) DNAJ domain protein, putative Location= chr6 complement(1199854..1200996)

ling) hypothetical protein Location= chr12 complement(join(1793737..1793785, 1793894..1793987, 1794116..1794177, 1794282..1794307, 179450

ling) small nuclear ribonucleoprotein, putative Location= chr13 complement(join(2005986..2006110, 2006209..2006298, 2006565..2006612, 20058

ling) hypothetical protein Location= chr4 1006053..1007210

ling) hypothetical protein Location= chr1 complement(join(226002..226017, 225348..225820))

ling) long-chain fatty acid CoA ligase, putative Location= chr5 1041741..1043762

ling) replication factor c subunit 4 Location= chr12 join(1778141..1778305, 1778553..1779398)

g) ribosomal protein L27, putative Location= chr14 complement(join(2475849..2476088, 2476399..2476409, 2475603..2475717))

g) casein kinase 1 Location= chr11 complement(join(1433478..1433577, 1433734..1434060, 1434196..1434268, 1434385..1434533, 1434681..143

ling) hypothetical protein Location= chr13 829735..833511

ling) hypothetical protein, conserved Location= chr8 complement(747136..748611)

ig) farnesyl pyrophosphate synthase, putative Location= chr11 complement(join(1104530..1104690, 1104803..1104901, 1105018..1105103, 11052[illegible]

ling) ribosomal protein l15, putative Location= chr4 complement(join(705566..705610, 705660..705751, 704901..705426))

ig) cyclophilin, putative Location= chr11 join(618311..618388, 618513..618565, 618687..618795, 618995..619116, 619478..619518, 619677..6197[illegible]

ling) hypothetical protein Location= chr12 complement(join(726030..726105, 726212..726363, 726520..728805, 729182..729830, 725834..725856))

ling) tyrosyl-tRNA synthetase, putative Location= chr8 join(403717..403936, 404198..404905, 405070..405263)

ıg) ribosomal protein S3, putative Location= chr14 join(2684077..2684088, 2684609..2684730, 2684939..2685470)

ıg) N-acetyltransferase, putative Location= chr10 complement(150417..150875)

ıg) hypothetical protein Location= chr10 join(701745..701891, 702070..702198, 702293..702421)

ıg) threonine -- tRNA ligase, putative Location= chr11 complement(1014333..1017374)

ling) NAD synthase, putative Location= chr9 1089901..1092420

ling) protein disulfide isomerase, putative Location= chr9 join(795529..796455, 796642..797155, 797354..797460)

ıg) hypothetical protein Location= chr10 join(1348431..1349136, 1349324..1349428, 1349623..1350278)

ıg) 26s proteasome subunit p55, putative Location= chr10 join(715877..715999, 716156..717436)

ıg) hypothetical protein Location= chr14 complement(1943879..1944907)

ding) hypothetical protein, conserved in P. falciparum Location= chr7 complement(join(81707..81798, 80831..81500))

ding) hypothetical protein Location= chr3 444174..448058

ding) chaperonin cpn60 Location= chr12 complement(join(1330341..1331361, 1328301..1329388))

ıg) ubiquitin-conjugating enzyme, putative Location= chr10 complement(join(1354817..1354945, 1355047..1355214, 1355333..1355412, 1355520..[illegible]

ıg) ribosomal protein S20e, putative Location= chr10 complement(join(160864..160963, 160317..160573))

ding) coatomer alpha subunit, putative Location= chr6 join(282045..286307, 286431..286599, 286734..286879, 286978..287013)

ıg) DEAD-box RNA helicase, putative Location= chr14 2420700..2422925

ling) chaperone, putative Location= chr6 join(358387..359896, 360078..360199)

ling) hypothetical protein Location= chr7 554716..555075

ıg) falcipain-2 precursor, putative Location= chr11 complement(581249..582697)
ıg) falcipain 2 precursor Location= chr11 complement(592129..593583)

ling) 3-methyl-2-oxobutanoate dehydrogenase (lipoamide), putative Location= chr5 177410..178555

ling) hypothetical protein Location= chr13 1892314..1892733

ling) hypothetical protein Location= chr13 join(2671924..2671960, 2672131..2672277, 2672427..2674300)

ling) 26S proteasome regulatory subunit, putative Location= chr9 join(567274..567793, 568135..568631)

ling) seryl-tRNA synthetase, putative Location= chr7 complement(820172..821791)

ling) proteasome precursor, putative Location= chr9 complement(1273072..1273920)

ling) 60S Ribosomal protein L44, putative Location= chr3 join(214615..214827, 215062..215163)

ıg) hypothetical protein Location= chr11 join(756191..758885, 759027..759183, 759476..759518)

ling) 26S proteasome regulatory subunit 7, putative Location= chr13 complement(join(491743..491811, 490129..491322))

ling) hypothetical protein Location= chr12 join(1572359..1572404, 1572959..1573545)

ling) hypothetical protein Location= chr12 1784367..1786397

g) hypothetical protein Location= chr11 703174..704958

ling) hypothetical protein Location= chr9 630648..632198

ling) eukaryotic initiation factor, putative Location= chr4 1044910..1046082

ling) regulator of chromosome condensation protein, putative Location= chr7 560625..562691

g) hypothetical protein Location= chr14 join(1578136..1578471, 1578747..1579118)

ling) glutathione peroxidase Location= chr12 complement(join(535225..535395, 535613..535909, 534906..535055))

ling) acyl carrier protein, mitochondrial precursor, putative Location= chr12 join(388540..388905, 389035..389142)

ng) hypothetical protein Location= chr10 join(414731..415002, 415102..415163, 415305..415327)

ling) hypothetical protein Location= chr1 complement(join(182146..182172, 182185..182215, 182343..182372, 182637..182656, 183034..183062, 1

ling) steroid dehydrogenase kik-i, putative Location= chr4 join(1003087..1003209, 1003402..1003734, 1003850..1003940, 1004091..1004221, 1004

ng) fibrillarin, putative Location= chr14 join(258746..258752, 259039..259988)

ling) hypothetical protein, conserved Location= chr13 complement(join(2403463..2403536, 2403619..2403684, 2403795..2403859, 2403979..24040

ling) translation initiation factor E4, putative Location= chr3 complement(609414..610097)

ng) ubiquitin carboxyl-terminal hydrolase, putative Location= chr14 join(2461257..2461388, 2461603..2461656, 2461791..2461931, 2462403..24624

ling) ribosomal protein, putative Location= chr8 join(974659..974838, 974939..975070, 975233..975340)

ling) prefoldin subunit, putative Location= chr9 join(210075..210285, 210429..210607)

ling) myosin a Location= chr13 join(1673730..1673825, 1673983..1674120, 1674348..1676570)

ling) subunit of proteaseome activator complex, putative Location= chr9 complement(364062..364901)

ling) hypothetical protein Location= chr8 complement(join(1019628..1019795, 1019895..1020004, 1020142..1020340, 1019161..1019493))

ling) transportin Location= chr6 1108198..1111641

ling) dihydropteroate synthetase Location= chr8 join(549321..549456, 549633..551501, 551622..551737)

ling) hypothetical protein Location= chr9 complement(187340..188620)

ling) Rab2 GTPase, putative Location= chr12 join(1276246..1276294, 1276426..1276564, 1276739..1276822, 1276962..1277163, 1277276..12773

ıg) small nuclear ribonucleoprotein, putative Location= pfal_chr2 join(752965..753099, 753268..753438)

ıg) eukaryotic translation initiation factor 3 subunit 7, putative Location= chr10 316649..318280

ıg) glyoxalase I, putative Location= chr11 529492..530562

ling) lysophospholipase-like protein, putative Location= chr7 complement(490469..491575)

ling) hypothetical protein Location= chr12 join(618757..618933, 619036..619149, 619352..619555, 619705..619863, 620021..620104)

ling) multidrug resistance protein Location= chr5 957885..962144

ıg) hypothetical protein Location= chr14 complement(1673329..1674012)

ling) antioxidant protein, putative Location= chr7 join(1303047..1303064, 1303133..1303642)

ling) hypothetical protein, conserved Location= chr7 348783..352472

ling) long-chain-fatty-acid--CoA ligase, putative Location= chr12 1610351..1612729

ling) FAD-dependent glycerol-3-phosphate dehydrogenase, putative Location= chr3 295191..297152

ling) thioredoxin, putative Location= chr13 complement(join(1806376..1806409, 1806502..1806551, 1806668..1806859, 1806074..1806271))

ıg) hypothetical protein Location= chr11 join(1608003..1608326, 1608427..1608606)

ling) GTP cyclohydrolase I Location= chr12 974363..975532

ling) thioredoxin-like redox-active protein, putative Location= chr3 186856..187395

ling) hypothetical protein Location= chr7 complement(join(169033..169141, 169246..169317, 168667..168803))

ling) calcium-transporting ATPase Location= chr1 complement(join(265913..266211, 266355..269412, 265447..265776))

ıg) valine - tRNA ligase, putative Location= chr14 2514608..2517880

ig) hypothetical protein Location= chr11 complement(1194522..1195034)

ding) V-type ATPase, putative Location= chr13 complement(join(2165808..2165911, 2166060..2166240, 2166377..2166477, 2166646..2166749, 21

ding) conserved protein Location= chr3 complement(join(149038..149130, 149229..152822, 148844..148921))

ig) proliferation-associated protein 2g4, putative Location= chr14 complement(join(1106345..1106456, 1105142..1106163))

ig) ADP/ATP transporter on adenylate translocase Location= chr10 complement(1477939..1478844)

ding) Fe-superoxide dismutase Location= chr6 complement(join(953107..953166, 953272..953391, 953481..953624, 953919..953960, 954223..954

ding) Ubiquitin Carboxyl-terminal Hydrolase-like zinc finger protein Location= chr13 708548..710470

ding) arginyl-tRNA synthetase, putative Location= chr12 complement(join(732314..732440, 732572..732674, 732833..732922, 733079..733198, 733

ıg) translation elongation factor EF-1, subunit alpha, putative Location= chr11 join(925727..926492, 926622..927523)

ling) lipoamide dehydrogenase Location= chr12 1333390..1334886

ling) ribosomal protein L32, putative Location= chr9 join(185819..185905, 186073..186381)

ıg) heat shock protein 90, putative Location= chr11 complement(688765..691557)

ling) SNAP protein (soluble N-ethylmaleimide-sensitive factor Attachment Protein), putative Location= chr5 complement(join(377372..377450, 3775

ıg) hypothetical protein Location= chr10 complement(207001..209643)

ıg) hypothetical protein Location= chr11 complement(1374590..1376659)

ıg) hypothetical protein Location= chr14 820063..821592

ıg) N-myristoyltransferase Location= chr14 complement(join(517765..517812, 517939..518061, 518179..518193, 516574..517620))

ıg) hypothetical protein Location= chr11 complement(join(684238..684615, 683859..684083))

ıg) hypothetical protein Location= chr11 complement(1985867..1986433)

ıg) ADP-ribosylation factor-like protein Location= chr10 1378756..1379292

ding) ubiquitin-conjugating enzyme E2, putative Location= chr6 complement(join(261384..261492, 261610..261670, 261865..261941, 261027..2612

ding) 40S ribosomal protein S16, putative Location= chr8 complement(join(679805..679846, 679110..679502))

ıg) hypothetical protein, conserved Location= chr14 join(269881..269912, 270092..270251, 270435..270521, 270634..270724, 270893..270927)

ding) vacuolar proton-translocating ATPase subunit A, putative Location= chr8 complement(364381..367542)

ıg) methionine aminopeptidase, type II, putative Location= chr14 1404255..1406141

ling) Histone deacetylase Location= chr9 complement(1028651..1030000)

ling) dihydrolipoamide acyltransferase, putative Location= chr3 complement(187921..189267)

ling) 26S proteasome regulatory subunit S14, putative Location= chr3 518644..519558

ling) ribose 5-phosphate epimerase, putative Location= chr5 complement(join(607394..607645, 607756..607923, 608026..608187, 608426..608449

ling) RNA-binding protein, putative Location= chr9 complement(join(967022..967119, 967250..967337, 967466..967572, 967670..967774, 967934..

ling) serine/threonine protein phosphatase pfPp5 Location= chr13 2195927..2197903

ıg) hypothetical protein Location= chr10 complement(join(1098804..1099880, 1098453..1098638))

ling) G10 protein, putative Location= chr5 complement(join(944398..944570, 944710..944926, 944179..944232))

ling) inorganic pyrophosphatase, putative Location= chr3 join(656694..657356, 657596..657658, 657901..658317)

ıg) 26S proteasome regulatory complex subunit, putative Location= chr11 join(1128772..1129134, 1129506..1129838, 1130112..1130558)

ling) glycerol-3-phosphate dehydrogenase, putative Location= chr12 join(648882..648926, 649098..649556, 649653..649784, 649962..650264, 650

ıg) uracil-DNA glycosylase, putative Location= chr14 complement(606379..607347)

ıg) hypothetical protein Location= chr14 1167086..1167745

ling) hypothetical protein Location= chr12 complement(join(108164..108202, 107338..107922))

ıg) ribonucleotide reductase small subunit, putative Location= chr10 join(633681..633767, 633890..633922, 634048..634151, 634411..635068, 6351

ling) hypothetical protein Location= chr13 join(982812..983125, 983251..983312, 983467..984002)

ling) heat shock protein DNAJ homologue Pfj4 Location= chr12 join(505338..505443, 505679..505780, 505952..506215, 506394..506656)

ling) conserved protein, putative Location= chr3 join(671131..671434, 671948..672309)

ling) snare protein homologue, putative Location= chr13 1035101..1035730

ling) dim1 protein homolog, putative Location= chr12 1299598..1300017

ling) clathrin heavy chain, putative Location= chr12 757642..763635

ling) hypothetical protein Location= chr13 join(916561..917907, 918099..918256, 918432..918507, 918679..918795)

ıg) hypothetical protein Location= chr14 complement(2410890..2411951)

ling) hypothetical protein, conserved Location= chr13 join(1369943..1369950, 1370223..1370997)

ling) hypothetical protein, conserved Location= chr9 complement(join(219293..219460, 218673..219074))

ıg) 26S proteasome subunit, putative Location= chr14 join(2709354..2712644, 2712729..2712856, 2713029..2713128)

ıg) hypothetical protein Location= chr14 2259401..2261233

ling) hypothetical protein, conserved Location= chr4 complement(join(784371..784424, 784531..784601, 784757..784805, 784925..784985, 78509[illegible]

ling) hypothetical protein Location= chr12 join(1941351..1941814, 1941916..1942047, 1942191..1942308, 1942406..1942477)

ling) iswi protein homologue Location= chr6 999525..1007684

ding) minichromosome maintenance protein 3, putative Location= chr5 complement(join(1125397..1128141, 1125094..1125237))

ding) polyadenylate binding protein, putative Location= chr13 complement(join(2437271..2437313, 2437462..2437646, 2437773..2437883, 2438035

ng) hypothetical protein Location= chr10 join(138766..138890, 139134..139214, 139406..139436, 139579..139668)

ng) hypothetical protein Location= chr14 join(859957..860069, 860230..863026)

ding) hypothetical protein Location= chr13 2564891..2567281

ding) transport protein Location= chr8 join(998127..998426, 998622..1000601)

ding) hypothetical protein Location= chr4 complement(join(324682..325101, 325215..325302, 325498..325650, 323012..324546))

ıg) merozoite surface protein 3 Location= chr10 1404192..1405256

ıg) heat shock 40 kDa protein, putative Location= pfal_chr2 join(537789..537797, 538286..538691, 538849..539420)

ıg) hypothetical protein Location= chr10 join(470978..471547, 471699..471932)

ling) hypothetical protein Location= chr13 join(99651..99719, 99956..100624)

ling) glutaminyl-tRNA synthetase, putative Location= chr13 complement(1319287..1322043)

ling) hypothetical protein, conserved Location= chr1 complement(421447..422007)

ıg) hypothetical protein Location= chr14 complement(join(1921141..1921588, 1920913..1920983))

ling) 60S ribosomal protein L11a, putative Location= chr7 complement(join(914796..914870, 914120..914566))

ıg) hypothetical protein, conserved Location= chr11 join(1067195..1067349, 1067516..1067618, 1067738..1067811, 1067916..1068002, 1068191..

ling) Pfsec61 Location= chr13 1839850..1841232

ling) hypothetical protein Location= chr9 complement(join(539797..539863, 538212..539386))

ling) proliferating cell nuclear antigen, putative Location= chr12 complement(join(1078588..1078766, 1077858..1078473))

ling) proteasome regulatory protein, putative Location= chr3 complement(734876..735553)

ling) asparagine synthetase, putative Location= chr3 join(399607..399686, 400362..402114)

ling) hypothetical protein Location= chr5 complement(653964..655334)

ling) ribosomal protein L17, putative Location= chr13 join(2048439..2048478, 2048664..2048932, 2049413..2049715)

ling) eukaryotic translation initiation factor 3 subunit 10, putative Location= chr12 complement(558040..562173)

ling) hypothetical protein Location= chr4 complement(join(411258..411530, 411661..411731, 411894..411933, 412240..412261, 410521..411149))

ling) cof-like hydrolase, had-superfamily, subfamily iib Location= chr12 1065786..1066673

ling) p-type ATPase, putative Location= chr12 complement(join(530399..532792, 528999..530231))

ıg) Leu/Phe-tRNA protein transferase, putative Location= pfal_chr2 533253..534350

ling) hydroxyacyl glutathione hydrolase, putative Location= chr4 join(338602..338634, 338805..338869, 339046..339091, 339174..339249, 339377.

ling) synaptobrevin-like protein, putative Location= chr13 complement(join(161107..161191, 161351..161458, 161559..161673, 161832..161862, 16

ling) falcilysin Location= chr13 2431676..2435257

ling) hypothetical protein Location= chr5 complement(247801..250575)

ling) DNA replication licensing factor mcm7 homologue, putative Location= chr7 complement(320356..322821)

ling) signal recognition particle receptor alpha subunit, putative Location= chr13 complement(2659757..2661487)

ling) protein phosphatase, putative Location= chr8 complement(join(535806..535907, 536034..536184, 536290..536422, 536617..536724, 536894..

ling) ring-infected erythrocyte surface antigen precursor Location= chr1 join(99052..99246, 99453..102515)

ling) glutamate--tRNA ligase Location= chr13 complement(1968221..1970812)

ng) V-type H(+)-translocating pyrophosphatase, putative Location= chr14 complement(2329869..2332022)

ng) cyclophilin, putative Location= chr14 join(933004..933223, 933365..934167, 934262..934327, 934492..934546, 934667..934740, 934875..9349[illegible]

ling) Ser/Arg-rich splicing factor, putative Location= chr5 complement(135004..135930)

ıg) protein-L-isoaspartate O-methyltransferase beta-aspartate methyltransferase, putative Location= chr14 complement(join(1291004..1291157, 129

ling) P. falciparum homologue of yeast snf7 Location= chr12 complement(join(1821021..1821073, 1821174..1821209, 1821295..1821352, 1821498

ıg) sortilin, putative Location= chr14 join(2123397..2125701, 2125865..2125969, 2126114..2126220, 2126365..2126535)

ıg) hypothetical protein Location= chr14 complement(join(1296410..1297153, 1296115..1296180))

ling) hypothetical protein, conserved in P. falciparum Location= chr7 join(661033..661101, 661194..661820)

ling) 40S ribosomal subunit protein S9, putative Location= chr5 join(828950..829012, 829229..829296, 829442..829880)

ıg) ribosomal protein S25, putative Location= chr14 complement(join(876025..876189, 875632..875874))

ıg) hypothetical protein Location= chr10 join(542977..544049, 544153..544555)

ling) hypothetical protein, expressed Location= chr7 complement(join(800898..802041, 800569..800741))

ıg) hypothetical protein Location= chr10 1335220..1336287

ıg) hypothetical protein, conserved Location= chr14 join(2255135..2255180, 2255294..2255326, 2255495..2255608, 2255758..2255843, 2256076..2

ıg) PfSNF2L Location= chr11 complement(join(182589..182708, 182841..183080, 183179..183262, 183430..187224, 182384..182425))

ıg) heat shock protein 101, putative Location= chr11 complement(join(638841..638929, 639079..639202, 639412..639510, 636093..638501))

ıg) acyl-CoA synthetase, PfACS9 Location= pfal_chr2 complement(616772..619429)

ıg) hypothetical protein Location= chr14 complement(523890..525305)

ding) hypothetical protein Location= chr5 553988..554785

ıg) hypothetical protein Location= chr14 complement(join(2794164..2794259, 2786244..2793824))

ıg) hypothetical protein Location= chr11 complement(join(332578..335601, 329867..332329))

ling) hypothetical protein Location= chr8 complement(join(807163..807264, 807384..807446, 807544..807615, 807732..807806, 807966..808073, 8

ling) hypothetical protein, conserved Location= chr6 complement(1196042..1198039)

ling) DNAJ-like Sec63 homologue Location= chr13 complement(774991..776946)

ling) hypothetical protein Location= chr5 join(1308273..1308473, 1308607..1309935)

ıg) hypothetical protein Location= chr11 complement(join(1603296..1603305, 1601254..1602593))

ling) TatD-like deoxyribonuclease, putative Location= chr1 complement(join(452744..453590, 453740..453840, 453982..454268, 452384..452453))

ling) PfpUB Plasmodium falciparum polyubiquitin Location= chr12 join(526103..526130, 526657..527774)
ling) ubiquitin/ribosomal fusion protein uba52 homologue, putative Location= chr13 complement(join(2629389..2629595, 2628869..2629048))

ıg) hypothetical protein, conserved Location= chr11 join(1593963..1594172, 1594311..1594511, 1594628..1594725, 1594861..1594948)

ıg) t-complex protein 1, alpha subunit, putative Location= chr11 complement(join(1250173..1250230, 1248308..1249884))

ling) cytochrome c oxidase subunit, putative Location= chr9 join(1128861..1129235, 1129484..1129930)

ıg) merozoite surface protein 4 Location= pfal_chr2 complement(join(277973..278456, 277494..277828))

ıg) hypothetical protein Location= chr14 complement(2524856..2528929)

ling) homologue of human HSPC025 Location= chr6 complement(509155..511125)

ling) hypothetical protein Location= chr1 complement(222492..224198)

ıg) hypothetical protein Location= chr11 complement(join(1095311..1095689, 1095813..1095929, 1094907..1095157))

ıg) protein tyrosine phosphatase, putative Location= chr11 complement(511104..511760)

ling) peptidyl-prolyl cis-trans isomerase precursor Location= chr8 279497..280150

ling) high mobility group protein 4, putative Location= chr13 complement(join(2347865..2347903, 2348014..2348080, 2348232..2348266, 2348469.

ling) 40S ribosomal protein S15A, putative Location= chr3 join(674515..674747, 674988..675147)

ling) ubiquitin-like protein, putative Location= chr9 join(895405..899649, 899822..899896, 899986..900294)

ling) hypothetical protein Location= chr4 543671..546007

g) hypothetical protein Location= chr14 complement(3055650..3057755)

ling) hypothetical protein Location= chr1 173099..174826

ling) hypothetical protein Location= chr13 complement(join(1094306..1094388, 1092595..1094095))

ling) eukaryotic translation initiation factor 3, subunit 6, putative Location= chr5 complement(join(1164968..1165066, 1163402..1164856))

ling) hypothetical protein Location= chr12 join(1085377..1085457, 1085848..1086573, 1086675..1086785, 1086899..1087054, 1087183..1087254)

ding) hypothetical protein Location= chr13 join(1822749..1823354, 1823481..1823987)

ding) DNA-directed RNA polymerase 2 subunit, putative Location= chr1 complement(join(395823..395989, 395029..395611))

ding) proteasome subunit alpha type 2, putative Location= chr6 complement(join(352663..352771, 351776..352374))

ding) ER lumen protein retaining receptor Location= chr13 complement(join(2143037..2143215, 2143341..2143480, 2142514..2142860))

ding) hypothetical protein Location= chr13 733446..734063

ng) glutamate-rich protein Location= chr10 1399192..1402893

ding) eukaryotic translation initiation factor 3 subunit 8, putative Location= chr12 complement(join(276186..278890, 279253..279333, 275741..27590

ding) splicing factor, putative Location= chr13 join(902584..904791, 904981..905367)

ding) RNA-binding protein, putative Location= chr9 complement(696853..698019)

ding) developmental protein, putative Location= chr9 join(305067..305129, 305255..305346, 305481..305595, 305730..305843, 305957..306025, 30

ng) protein phosphatase 2b regulatory subunit, putative Location= chr14 complement(join(2119024..2119104, 2119225..2119370, 2119569..211965

ling) beta adaptin protein, putative Location= chr5 complement(join(1158038..1158154, 1158474..1158608, 1158744..1158814, 1159004..1159065,

ıg) 26S proteasome subunit, putative Location= chr10 1244213..1245394

ling) 60S ribosomal subunit protein L4/L1, putative Location= chr5 complement(294668..295903)

ling) hypothetical protein Location= chr4 join(1080786..1081411, 1081511..1081973)

ling) prohibitin, putative Location= chr8 1255629..1256447

ıg) dolichyl-phosphate b-D-mannosyltransferase, putative Location= chr11 complement(1668299..1669078)

ıg) multiprotein bridging factor type 1, putative Location= chr11 1097115..1097525

ıg) histidine -- tRNA ligase, putative Location= chr14 complement(1851599..1854997)

ling) hypothetical protein Location= chr12 complement(join(1129860..1130851, 1131127..1131676, 1128929..1129702))

ling) hypothetical protein Location= chr3 663680..665065

ıg) ubiquitin C-terminal hydrolase, family 1, putative Location= chr11 652578..653975

ıg) hypothetical protein, conserved Location= chr11 join(1729790..1729913, 1730055..1730896)

ling) 40S ribosomal subunit protein S6, putative Location= chr13 complement(1653242..1654162)

ling) cytochrome c1 heme lyase, putative Location= chr12 join(184911..185061, 185229..185478, 185612..185765)

ling) hypothetical protein, conserved Location= chr13 complement(2322727..2323188)

ling) hypothetical protein, conserved Location= chr3 join(166194..166222, 166465..169693, 169807..169907, 170093..170171, 170899..170976)

ling) ATP-dept. acyl-coa synthetase Location= chr7 1423539..1425974

ıg) hypothetical protein Location= chr11 complement(619892..621349)

ling) hypothetical protein Location= chr8 complement(join(1258842..1260112, 1258562..1258607))

ling) hypothetical protein Location= chr13 join(1923780..1923843, 1924041..1924591)

ling) hypothetical protein Location= chr12 complement(join(595441..598637, 595160..595244))

ling) STEVOR Location= chr6 complement(join(739194..739262, 738256..739095))

ling) hypothetical protein Location= chr4 complement(join(154733..154840, 153950..154606))

ıg) hypothetical protein, conserved Location= chr14 complement(1433681..1434253)

ling) erythrocyte binding antigen Location= chr7 join(1413433..1417696, 1417822..1417946)

ıg) hypothetical protein Location= chr14 complement(1536427..1538412)

ıg) hypothetical protein Location= pfal_chr2 complement(735437..736996)

ling) isocitrate dehydrogenase (NADP), mitochondrial precursor Location= chr13 complement(1824621..1826027)

ling) PfmpC Location= chr12 complement(116475..117449)

ling) D-ribulose-5-phosphate 3-epimerase, putative Location= chr12 796493..797176

ling) ATP-dependent heat shock protein, putative Location= chr9 complement(351948..354716)

ling) phenylalanyl-tRNA synthetase beta chain, putative Location= chr1 381851..383578

ling) hypothetical protein Location= chr13 2191782..2193311

ling) hypothetical protein Location= chr8 complement(280827..282302)

ling) hypothetical protein Location= chr8 complement(join(652818..653058, 653182..653343, 653481..653925, 652538..652631))

ig) U2 snRNP auxiliary factor, small subunit, putative Location= chr11 complement(724854..725738)

ling) long chain polyunsaturated fatty acid elongation enzyme, putative Location= chr6 join(247637..247951, 248074..248201, 248333..248497, 248

ig) methionine aminopeptidase, putative Location= chr10 618439..619992

ling) ATP synthase beta chain, mitochondrial precursor, putative Location= chr12 1487071..1488678

ling) hypothetical protein Location= chr13 complement(join(1197008..1197141, 1197281..1197710, 1196430..1196864))

ling) RIFIN Location= chr1 complement(join(40377..40430, 39205..40146))

ling) hypothetical protein, conserved Location= chr3 complement(join(94405..94587, 93248..94018))

ig) asparagine-rich antigen Location= chr11 join(414797..415106, 415281..415394, 415529..419556)

ling) hypothetical protein Location= chr12 1204980..1207952

ling) translation initiation factor IF-2, putative Location= chr6 293860..296793

ding) tryptophan/threonine-rich antigen Location= chr8 complement(join(1299223..1299399, 1297205..1299055))

ding) aspartyl (acid) protease, putative Location= chr13 975604..977376

ding) chloroquine resistance transporter, putative Location= chr7 join(458602..458692, 458870..459138, 459318..459490, 459663..459795, 459949.

ding) hypthetical protein Location= chr1 complement(204860..205627)

ding) RNA helicase, putative Location= chr8 543092..545917

ng) replication factor C3 Location= chr14 join(2570369..2570413, 2570665..2571654)

ng) basic transcription factor 3b, putative Location= chr14 complement(1018687..1019202)

ding) hypothetical protein Location= chr4 complement(join(1088526..1088589, 1086802..1088318))

ng) choline kinase, putative Location= chr14 71368..72690

g) Ribosomal protein, putative Location= chr11 complement(join(1708525..1708676, 1708063..1708333))

g) 10b antigen, putative Location= chr10 complement(905471..912343)

g) DNA topoisomerase II, putative Location= chr14 complement(join(1332475..1332546, 1332671..1332789, 1333058..1337243, 1332305..133234

ling) DNA replication licensing factor mcm4-related Location= chr13 712146..715163

ling) er lumen protein retaining receptor 1, putative Location= chr13 complement(join(1280244..1280303, 1280427..1280480, 1280603..1280674, 1

ling) hypothetical protein, conserved Location= chr8 join(1069635..1070099, 1070255..1070482, 1070644..1070689, 1070796..1070857)

g) hypothetical protein Location= chr11 complement(301283..311287)

ıg) hypothetical protein Location= chr14 2134234..2140803

ıg) hypothetical protein Location= chr10 complement(join(1452801..1452995, 1453165..1458005, 1452598..1452607))

ding) Plasmodium falciparum asparagine-rich protein Location= chr13 549465..553043

ding) hypothetical protein Location= chr12 2012629..2015712

ding) hypothetical protein, conserved Location= chr13 join(2106685..2108813, 2108963..2109083, 2109285..2109689)

ıg) hypothetical protein Location= chr11 join(1118247..1119092, 1119314..1119971, 1120163..1120504, 1120601..1120683)

ling) RNA binding protein, putative Location= chr13 complement(join(2356439..2357531, 2355584..2356020))

ıg) hypothetical protein Location= chr14 3072196..3073182

ıg) merozoite surface protein 5 Location= pfal_chr2 complement(join(275981..276494, 275540..275844))

ling) hypothetical protein Location= chr13 join(1474471..1475081, 1475171..1475251, 1475412..1475459, 1475596..1475710, 1475820..1475858)

ling) hypothetical protein Location= chr13 complement(463587..464204)

ling) hypothetical protein, conserved Location= chr8 complement(627189..630065)

ıg) ribonucleoside-diphosphate reductase, large subunit Location= chr14 complement(join(1507798..1510032, 1507302..1507610))

ıg) structure specific recognition protein, putative Location= chr14 1690936..1692456

ling) hypothetical protein Location= chr1 complement(join(539679..539747, 538831..539493))

ling) hypothetical protein, conserved Location= chr13 join(1272411..1272527, 1272649..1272735, 1272866..1272898, 1273072..1273163, 1273318

ıg) hypothetical protein, conserved Location= chr14 complement(726978..728378)

ling) ethanolaminephosphotransferase, putative Location= chr6 complement(join(1170759..1170812, 1170916..1171059, 1171203..1171295, 1171[illegible]

g) replication factor C subunit 5, putative Location= chr11 complement(437171..438220)

g) hypothetical protein, conserved Location= chr10 join(121116..121170, 121427..121544, 121657..121745, 122255..122332, 122501..122588, 12[illegible]

ling) hypothetical protein Location= chr9 888549..889295

ling) thymidylate kinase, putative Location= chr12 complement(join(2095219..2095328, 2095452..2095560, 2095695..2095785, 2095949..2096057,

g) vacuolar protein sorting 29, putative Location= chr14 244908..245492

ling) hypothetical protein Location= chr8 join(949825..950989, 951153..951255, 951349..951419, 951560..951684, 951894..951974)

g) co-chaperone GrpE, putative Location= chr11 complement(974653..975537)

g) rab5 protein, putative Location= pfal_chr2 complement(455579..456286)

ling) ubiquitin carboxyl-terminal hydrolase a, putative Location= chr4 complement(join(630393..630480, 630663..632944, 633112..633209, 630151.

ling) DNA primase, large subunit, putative Location= chr9 complement(496141..497718)

g) hypothetical protein Location= chr14 complement(join(854322..854556, 854675..855909, 854069..854113))

g) hypothetical protein Location= chr10 complement(join(1449088..1449145, 1449548..1450487, 1450651..1450835, 1451559..1451645, 1448617.

ling) RIFIN Location= chr1 join(605882..605935, 606166..607251)

g) phosphotyrosyl phosphatase activator, putative Location= chr14 join(1184852..1185417, 1186123..1186273, 1186428..1186502, 1186654..1186

ding) hypothetical protein Location= chr9 complement(350179..350955)

ding) 60S ribosomal subunit protein L8, putative Location= chr5 complement(join(713752..713770, 712760..713523))

ng) hypothetical protein Location= chr11 join(59398..59427, 59702..60328)
ding) hypothetical protein, conserved in P. falciparum Location= chr8 complement(join(1371173..1371241, 1370435..1371061))

ding) Ebl-1 like protein, putative Location= chr1 complement(join(111196..111284, 111401..111479, 111572..116033, 110984..111057))

ng) glycine -- tRNA ligase, putative Location= chr14 846533..849172

ng) dynamin protein, putative Location= chr10 1487958..1490087

PRECURSOR (GRP 78) (IMMUNOGLOBULIN...
PRECURSOR (GRP 78) (IMMUNOGLOBULIN...
PRECURSOR (GRP 78) (IMMUNOGLOBULIN...
PRECURSOR (GRP 78) (IMMUNOGLOBULIN...

ng) cysteine -- tRNA ligase, putative Location= chr10 complement(join(616679..616795, 614871..616535))

ding) mitochondrial processing peptidase alpha subunit, putative Location= chr5 complement(join(964661..965039, 963222..964453))

ling) hypothetical protein, conserved Location= chr13 complement(join(2621216..2621272, 2621422..2622433, 2620888..2621045))

ıg) DNA repair protein RAD23, putative Location= chr10 449153..450322

ling) DNAJ-like molecular chaperone protein, putative Location= chr9 join(786146..786236, 786792..786946, 787047..787164, 787296..787414, 78[illegible]

ling) protein kinase, putative Location= chr3 join(891430..891734, 891824..892362, 892458..892506, 892605..892631, 892715..892781)

ling) eukaryotic translation initiation factor 2 alpha subunit, putative Location= chr7 complement(join(1247517..1247667, 1246557..1247395))

ling) hypothetical protein Location= chr6 join(778689..779511, 779602..779633)

ling) vacuolar ATP synthase subunit D, putative Location= chr13 complement(join(1649331..1649401, 1649563..1649642, 1649763..1649828, 164[illegible]

ling) hypothetical protein Location= chr12 918713..919327

ling) hypothetical protein Location= chr12 complement(1088526..1090013)

ling) hypothetical protein Location= chr13 complement(289766..291202)

ling) clustered-asparagine-rich protein Location= chr12 complement(join(1508507..1508548, 1506919..1508256))

ıg) hypothetical protein Location= pfal_chr2 complement(join(113452..113682, 112547..113188))

ıg) coatamer protein, beta subunit, putative Location= chr14 join(1170771..1174501, 1174695..1175082, 1175170..1175235)

ling) hypothetical protein Location= chr12 join(1847665..1851043, 1851150..1851260, 1851441..1851626, 1851781..1851950, 1852100..1852144)

ıg) hypothetical protein Location= chr10 complement(join(801653..803501, 800277..801241))

ling) DNA replication licensing factor mcm5, putative Location= chr12 join(521681..523632, 523802..523946, 524184..524293, 524395..524464)

ıg) hypothetical protein Location= chr14 complement(join(2499953..2500079, 2500230..2500319, 2497982..2499798))

ıg) phenylalanine -- tRNA ligase, putative Location= chr11 complement(join(175801..175920, 176081..177779, 175609..175661))

ling) AP endonuclease (DNA-(apurinic or apyrimidinic site) lyase), putative Location= chr3 complement(272539..274392)

ling) hypothetical protein Location= chr8 complement(1222097..1223755)

ıg) hypothetical protein Location= chr14 complement(join(1013014..1013692, 1013925..1014207, 1012638..1012707))

ling) hypothetical protein Location= chr5 complement(839652..840557)

ling) hypothetical protein Location= chr8 635110..635817

ıg) hypothetical protein Location= chr14 complement(join(2942469..2942550, 2942682..2943196, 2942270..2942377))

ling) hypothetical protein, conserved Location= chr12 complement(join(977041..977223, 977363..977528, 977758..977880, 976811..976917))

ıg) hypothetical protein, conserved Location= pfal_chr2 complement(join(569171..569259, 569376..573315, 568695..569048))

ıg) hypothetical protein Location= chr14 complement(2998529..3002290)

ling) hypothetical protein Location= chr1 complement(join(353612..353693, 353836..353968, 354164..357364, 353467..353509))

ling) eukaryotic translation initiation factor 3 subunit, putative Location= chr5 743177..745327

ling) hypothetical protein Location= chr3 complement(405776..407602)

ıg) hypothetical protein Location= chr14 complement(join(353497..355141, 353263..353318))

ling) phosphoenolpyruvate carboxykinase Location= chr13 1679724..1681475

ling) u1 small nuclear ribonucleoprotein a, putative Location= chr13 join(323675..324965, 325177..325234, 325426..325438)

ding) Plasmodium falciparum membrane protein pf12 precursor Location= chr6 complement(525982..527025)

ding) hypothetical protein Location= chr4 1060105..1060758

ding) hypothetical protein Location= chr8 complement(763246..766458)

ding) hypothetical protein Location= chr6 complement(join(597800..597872, 598023..598114, 598281..600512, 597479..597643))

ng) PfGCN20 Location= chr11 820072..822519

ng) apical membrane antigen 1 precursor Location= chr11 1293854..1295722

ding) ubiquitin carboxyl-terminal hydrolase, putative Location= chr5 complement(join(1134749..1134849, 1132932..1134648))

ding) hypothetical protein Location= chr3 525713..527452

ding) hypothetical protein Location= chr12 1520315..1521592

ng) hypothetical protein Location= chr10 complement(997473..1007459)

ıg) phosphoenolpyruvate carboxylase, putative Location= chr14 complement(1036079..1039525)

ling) acetyl-coenzyme a synthetase Location= chr6 complement(1114545..1117538)

ling) eukaryotic translation initiation factor 5, putative Location= chr12 complement(301126..302823)

ling) hypothetical protein Location= chr13 complement(join(648443..649553, 648188..648228))

ıg) Rad51 homolog, putative Location= chr11 complement(join(319190..319217, 317819..318891))

ıg) hypothetical protein Location= chr14 complement(797708..799465)

ling) glyoxalase II family protein, putative Location= chr12 253152..254120

ıg) hypothetical protein Location= chr10 164946..165791

ling) signal peptidase, putative Location= chr13 join(1309375..1309525, 1309801..1310204)

ıg) sulfate transporter, putative Location= chr14 join(2912323..2913078, 2913276..2913510, 2913848..2914305, 2914400..2914522, 2914677..291[illegible]

ıg) hypothetical protein Location= chr10 1423980..1425680

ıg) hypothetical protein Location= chr14 join(551862..552654, 552758..553017, 553143..553235)

ıg) hypothetical protein Location= chr11 complement(join(179480..180232, 180352..180451, 180807..180913, 179152..179202))

ıg) replication factor C, subunit 2 Location= pfal_chr2 738859..739851

ling) carbamoyl phosphate synthetase, putative Location= chr13 complement(join(368671..368698, 361369..368468))

ling) erythrocyte membrane-associated antigen Location= chr7 join(190980..191624, 191678..197884)

ling) hypothetical protein Location= chr4 join(725185..725472, 725586..728987, 729295..729351, 729601..729657)

ng) hypothetical protein Location= chr11 join(555513..556201, 556327..556555, 556647..556943, 557061..557371, 557486..557592, 557699..55792

ng) hypothetical protein Location= chr14 383188..384732

ling) hypothetical protein Location= chr12 549882..550904

ling) hypothetical protein Location= chr3 join(982935..982970, 983117..983285, 983427..983736, 983848..984280)

ng) hypothetical protein Location= chr11 1883772..1887857

ng) cGMP-dependent protein kinase 1, beta isozyme, putative Location= chr14 complement(join(1492718..1493148, 1493318..1493492, 1493697..1

ling) chromatin assembly factor 1 subunit, putative Location= chr13 complement(1239687..1241435)

ng) hypothetical protein Location= chr14 complement(join(2493791..2494409, 2494533..2494697, 2493426..2493613))

ng) ubiquitin conjugating enzyme, putative Location= chr14 join(521209..521256, 521485..521534, 521636..521705, 521807..521860, 522032..5220

ling) MYND finger domain protein Location= chr6 join(97887..98425, 98621..98738, 98847..98948)

ng) ribonucleoprotein, putative Location= chr14 complement(join(600509..600679, 599762..600196))

ng) hypothetical protein Location= chr11 complement(join(606842..606923, 607922..613393, 603294..606640))

ng) hypothetical protein Location= chr11 join(772538..774838, 774967..775041)

ling) hypothetical protein, conserved Location= chr6 join(674114..675616, 675719..675748, 675864..675986, 676121..676173, 676306..676372)

ng) 1-deoxy-D-xylulose 5-phosphate reductoisomerase Location= chr14 2750894..2752360

ng) hypothetical protein, conserved Location= chr11 complement(join(1267453..1267582, 1266248..1267278))

ng) hypothetical protein Location= chr14 complement(join(1924725..1925595, 1926517..1926593, 1924368..1924571))

ng) hypothetical protein, conserved Location= chr10 complement(join(497443..497572, 496330..497342))

ling) serine/threonine protein phosphatase, putative Location= chr3 complement(576215..577141)

ıg) transcriptional activator ADA2, putative Location= chr10 complement(581353..589089)

ding) hypothetical protein Location= chr7 260099..263131

ıg) hypothetical protein Location= chr14 complement(1669923..1671593)

ıg) GMP synthetase Location= chr10 complement(482526..484193)

ding) EH (Eps15 homology) protein Location= chr3 complement(204018..205619)

ıg) endoplasmic reticulum oxidoreductin, putative Location= chr11 join(948590..949170, 949412..949755, 949920..950146, 950358..950461, 95057

ding) hypothetical protein Location= chr12 complement(1601503..1602684)

ıg) actin, putative Location= chr11 join(428362..428398, 428581..429515, 429696..429902)

ding) triose or hexose phosphate / phosphate translocator, putative Location= chr5 342205..343233

ıg) hypothetical protein Location= chr14 complement(959019..963695)

ding) glutamate dehydrogenase, putative Location= chr8 147210..151403

ding) hypothetical protein Location= chr8 complement(1008849..1012379)

ding) hypothetical protein Location= chr4 complement(join(122360..122509, 121052..122188))

ding) aminomethyltransferase, mitochondrial precursor Location= chr13 join(2615363..2615437, 2615757..2616902)

ıg) hypothetical protein Location= chr10 403678..404574

ding) hypothetical protein, conserved Location= chr6 complement(join(865848..869906, 865744..865755))

ding) beta subunit of coatomer complex, putative Location= chr9 complement(293582..296614)

ıg) hypothetical protein Location= chr10 complement(join(1046027..1048017, 1048198..1048593, 1049118..1049241, 1049369..1049462, 1049613.

ıg) citrate synthase, mitochondrial precursor, putative Location= chr10 complement(944396..946087)

ıg) hypothetical protein Location= chr14 complement(2306891..2308138)

ıg) hypothetical protein Location= chr14 615166..616179

ıg) hypothetical protein Location= chr14 2619614..2624122

ding) hypothetical protein Location= chr8 1099556..1101448

ding) hypothetical protein Location= chr9 join(1263337..1263354, 1263499..1263577, 1263792..1264561)

ling) malate dehydrogenase, putative Location= chr6 774526..775467

ng) rhoptry protein, putative Location= chr14 join(2736844..2737039, 2737207..2741255, 2742011..2742133)

ng) hypothetical protein Location= chr11 694654..699120

ng) hypothetical protein Location= chr14 374800..377106

ling) cell differentiation protein rcd1, putative Location= chr5 313199..315157

ling) actin-related protein, putative Location= chr5 230695..232392

ling) hypothetical protein Location= chr13 1398469..1400043

ling) hypothetical protein Location= chr4 complement(1056016..1057446)

ng) deoxyhypusine synthase Location= chr14 complement(509686..511176)

ng) hypothetical protein Location= chr11 complement(join(1703628..1703639, 1702086..1703486))

ling) mitochondrial import receptor subunit tom40 Location= chr6 complement(704032..705165)

ling) pre-mRNA splicing factor, putative Location= chr4 join(297721..297771, 298087..307446)

ling) hypothetical protein Location= chr1 338321..344986

ıg) hypothetical protein Location= chr14 complement(1972441..1977366)

ıg) DNA polymerase delta catalytic subunit Location= chr10 685577..688861

ıg) DNA replication licensing factor MCM2 Location= chr14 join(753388..753393, 753530..756439)

ling) hypothetical protein Location= chr13 join(1503211..1505055, 1505352..1506218)

ling) hypothetical protein Location= chr13 complement(join(354870..355021, 352043..354551))

ıg) proteosome subunit, putative Location= chr14 complement(85883..87883)

ıg) hypothetical protein Location= chr14 2748328..2750067

ıg) hypothetical protein Location= chr14 join(1502591..1502648, 1502839..1504427)

ling) dihydrofolate synthase/folylpolyglutamate synthase Location= chr13 complement(join(1036914..1037036, 1037182..1037380, 1037530..10387

ling) malate:quinone oxidoreductase, putative Location= chr6 695926..697491

ling) hypothetical protein Location= chr13 join(663943..667329, 667513..667602, 667769..667930, 668072..668131)

ling) deoxyribodipyrimidine photolyase (photoreactivating enzyme, DNA photolyase), putative Location= chr5 complement(join(575868..578503, 57[illegible]

ling) hypothetical protein Location= chr13 join(1828180..1830103, 1830269..1831329)

ling) aspartyl-tRNA synthetase Location= chr1 complement(129194..131074)

ling) hypothetical protein Location= chr13 complement(join(281315..281903, 280192..281030))

ng) monosaccharide transporter, putative Location= pfal_chr2 complement(205889..207403)

ng) hypothetical protein Location= chr10 join(273599..273604, 274383..275426)

ling) hypothetical protein, conserved Location= chr6 complement(join(421417..421938, 422193..422320, 422527..422617, 421152..421265))

ling) hypothetical protein, conserved Location= chr12 complement(join(148466..148594, 148706..148804, 148983..149111, 149246..149368, 1495[illegible]

ling) hypothetical protein Location= chr8 complement(join(315305..315378, 315560..319852, 315166..315214))

ling) erythrocyte membrane protein pfemp3, putative Location= chr13 complement(1774308..1778498)

ling) hypothetical protein Location= chr12 join(1407661..1410576, 1410738..1410872)

ng) hypothetical protein Location= chr10 1275861..1278575

ling) subtilisin-like protease precursor, putative Location= chr5 complement(307490..309556)

ig) hypothetical protein, conserved Location= pfal_chr2 223478..224851

ling) histone acetyltransferase Gcn5, putative Location= chr8 join(1029824..1033135, 1033292..1033766, 1033812..1034320, 1034425..1034586)

ig) hypothetical protein, conserved Location= chr14 complement(join(645658..648453, 645499..645519))

ling) apurinic/apyrimidinic endonuclease Apn1 Location= chr13 complement(1342210..1344003)

ling) Merozoite surface protein 10, MSP10 Location= chr6 complement(851375..852952)

ig) peptide chain release factor subunit 1, putative Location= pfal_chr2 join(492616..492689, 492831..492985, 493252..494306)

ig) arginine n-methyltransferase, putative Location= chr14 complement(1021594..1022799)

ling) transcription elongation factor s-ii, putative Location= chr7 720334..721545

ig) hypothetical protein, conserved Location= chr14 join(3060799..3061233, 3061395..3061529, 3061679..3061829, 3062008..3062396, 3062495..[illegible]

ig) acid phosphatase, putative Location= chr14 complement(join(142266..143138, 141410..141445))

ling) Cytoadherence linked asexual protein Location= chr7 complement(join(111997..112763, 112912..113070, 113208..113384, 113519..113650, [illegible]

ig) DNA-directed RNA polymerase II second largest subunit, putative Location= pfal_chr2 join(645929..645980, 646115..650079, 650684..650761)

ig) hypothetical protein, conserved Location= chr10 1169938..1171389

ling) hypothetical protein Location= chr12 join(700524..700576, 700715..700806, 700898..702204)

ng) hypothetical protein Location= chr11 join(1071907..1071933, 1072032..1072255, 1072374..1072476, 1072592..1072681, 1072792..1072825, 10

ng) hypothetical protein Location= chr10 129277..132111

ling) hypothetical protein Location= chr1 join(447177..447926, 448043..448195, 448490..448573, 448768..448898, 448992..449013, 449178..4493

ling) ethanolamine-phosphate cytidylyltransferase, putative Location= chr13 join(1905291..1906880, 1906989..1907062, 1907159..1907216)

ling) dihydroorotate dehydrogenase, mitochondrial precursor Location= chr6 complement(130326..132035)

ng) DNA polymerase alpha subunit, putative Location= chr14 2572923..2574542

ng) hypothetical protein Location= chr11 379247..380638

ng) adenylosuccinate lyase, putative Location= pfal_chr2 271594..273009

ling) hypothetical protein, expressed Location= chr6 join(189009..197382, 197544..197588, 197742..197815, 197944..198075)

ling) hypothetical protein Location= chr12 1470699..1475687

ıg) hypothetical protein Location= chr10 join(487251..491271, 491404..491605, 491734..491827)

ling) transcriptional regulator, putative Location= chr5 join(731405..734611, 734830..734968, 735081..735160)

ling) hypothetical protein Location= chr5 91361..94591

ling) hypothetical protein, conserved Location= chr3 394323..397190

ling) DNA ligase 1 Location= chr13 complement(join(224726..224922, 222018..224559))

ling) importin beta, putative Location= chr8 718234..720867

ling) tryptophan--tRNA ligase, putative Location= chr13 complement(join(1490345..1490458, 1488257..1490041))

ling) DEAD/DEAH box ATP-dependent RNA helicase, putative Location= chr6 complement(join(1291314..1292975, 1291038..1291181))

ıg) ATP synthase F1, alpha subunit, putative Location= pfal_chr2 707737..709392

ling) phosphoenolpyruvate/phosphate translocator precursor, putative Location= chr5 complement(1229070..1230644)

ling) hypothetical protein, conserved Location= chr5 complement(681428..682435)

ling) leucyl-tRNA synthetase, cytoplasmic, putative Location= chr6 919683..924026

ıg) hypothetical protein Location= chr11 1033060..1037166

ling) ubiquitin carboxyl-terminal hydrolase 2, putative Location= chr5 join(694490..694614, 694771..695176, 695309..698776)

ıg) gamma-adaptin, putative Location= chr14 join(2267137..2269927, 2270093..2270154, 2270281..2270457, 2270572..2270787)

ıg) hypothetical protein, conserved Location= chr10 complement(1386646..1389627)

ling) hypothetical protein Location= chr3 join(939216..941825, 941893..941969, 942058..942070)

ling) hypothetical protein, conserved Location= chr6 complement(893448..895778)

ling) ATP-dependent helicase, putative Location= chr5 172150..174417

ıg) hypothetical protein, conserved Location= chr14 2078425..2080287

ling) chromatin assembly factor 1 subunit, putative Location= chr13 complement(1239687..1241435)

ling) hypothetical protein Location= chr4 824381..825670

ling) hypothetical protein Location= chr12 complement(join(2030944..2031046, 2031220..2031336, 2031437..2031513, 2031688..2039837, 202998

ling) P. falciparum myosin Location= chr13 complement(join(1227865..1234073, 1234348..1234462, 1234560..1234619, 1227573..1227671))

ling) hypothetical protein Location= chr13 complement(join(2134798..2134916, 2128401..2134488))

ıg) hypothetical protein Location= chr14 complement(join(2585217..2585300, 2585415..2585534, 2585672..2585881, 2585967..2586035, 2586126.

ıg) hypothetical protein Location= chr14 3124272..3126644

ling) hypothetical protein Location= chr8 complement(join(205832..207781, 205361..205597))

ling) hypothetical protein Location= chr13 complement(join(1408108..1408127, 1405929..1407810))

ling) hypothetical protein, conserved Location= chr3 complement(join(285623..287422, 285312..285398))

ling) protein phosphatase, putative Location= chr8 join(193096..193161, 193475..195223)

ig) hypothetical protein Location= chr11 complement(join(1008802..1009188, 1009348..1009433, 1009669..1010852, 1008408..1008541))

ig) hypothetical protein Location= chr11 1950207..1951919

ig) hypothetical protein Location= chr14 join(906565..906699, 906885..907148, 907249..908142, 908574..908837)

ig) hypothetical protein Location= chr14 217845..226307

ig) hypothetical protein Location= chr14 complement(join(2759601..2764086, 2757601..2759429))

ling) hypothetical protein Location= chr7 complement(join(1070582..1071827, 1068673..1070438))

ling) hypothetical protein Location= chr7 413081..415966

ig) hypothetical protein Location= chr10 1413197..1415290

ig) hypothetical protein, conserved Location= chr14 complement(2630493..2632613)

ig) hypothetical protein, conserved Location= chr10 join(863905..863997, 864124..864507, 864683..864792, 865010..865888, 866102..866155, 86[illegible]

ling) RNAse L inhibitor protein, putative Location= chr13 complement(join(2709937..2711770, 2709797..2709822))

ig) hypothetical protein, conserved Location= chr11 complement(join(1524275..1524343, 1522596..1524029))

ling) hypothetical protein Location= chr8 join(728783..729344, 729548..729867, 729994..730369, 730519..730657, 730771..730849)

ling) hypothetical protein Location= chr3 join(372481..373764, 373918..374112)

ling) hypothetical protein conserved Location= chr12 863803..865179

ling) hypothetical protein Location= chr13 complement(join(2063864..2065098, 2063645..2063681))

ling) hypothetical protein Location= chr9 complement(join(1303663..1303964, 1304170..1307779, 1303380..1303496))

ıg) hypothetical protein Location= chr10 complement(199453..202845)

ıg) multidrug resistance protein 2 Location= chr14 complement(1954544..1957618)

ıg) PP1-like protein serine/threonine phosphatase Location= chr14 join(936113..936350, 936445..936583, 936734..936860, 936998..937093, 9372(

ling) hypothetical protein Location= chr7 394422..396779

ling) hypothetical protein Location= chr8 complement(648437..650425)

ıg) hypothetical protein Location= chr11 326881..328812

ling) ferrodoxin reductase-like protein Location= chr7 join(938314..939868, 939993..940366)

ıg) hypothetical protein Location= chr11 complement(1710322..1712169)

ling) hypothetical protein Location= chr3 complement(405776..407602)

ling) hypothetical protein Location= chr4 466607..468334

ıg) hypothetical protein Location= chr14 complement(1769569..1771293)

ling) hypothetical protein, conserved Location= chr12 complement(182295..183965)

ıg) hypothetical protein Location= chr11 1487591..1496140

ling) hypothetical protein Location= chr4 complement(817976..821623)

ling) 2-oxoglutarate dehydrogenase e1 component Location= chr8 join(933417..933574, 933764..936722)

ıg) hypothetical protein Location= chr10 1260308..1263067

ıg) cleavage and polyadenylation specifity factor protein, putative Location= chr14 complement(1557470..1560100)

ling) hypothetical protein, conserved Location= chr12 complement(join(1621230..1623693, 1620876..1620970))

ıg) hypothetical protein Location= chr14 complement(3112444..3114633)

ling) hypothetical protein Location= chr7 399278..401281

ling) hypothetical protein Location= chr3 complement(join(467808..469002, 469165..469389, 469588..469837, 467536..467668))

ıg) methionine -- tRNA ligase, putative Location= chr14 complement(join(1713880..1714008, 1712569..1713648))

ling) hypothetical protein Location= chr4 join(1063436..1064120, 1064557..1064990)

ling) hypothetical protein Location= chr12 complement(1192877..1199203)

ling) hypothetical protein Location= chr13 complement(join(1456671..1457100, 1457297..1461524, 1456308..1456479))

ling) hypothetical protein Location= chr12 complement(1039400..1043905)

ling) DNA repair protein, putative Location= chr5 complement(join(240644..243880, 239456..240271))

ling) wd repeat protein, putative Location= chr8 complement(168189..171554)

ıg) hypothetical protein Location= chr11 complement(join(121346..121507, 119413..121125))

ling) binding protein, putative Location= chr3 join(175096..176403, 176495..176568, 176711..176751, 176892..177007, 177112..177239, 177374..1

ling) hypothetical protein Location= chr9 join(1357400..1357495, 1357607..1357657, 1357803..1359251)

ling) hypothetical protein, conserved Location= chr3 201218..202795

ıg) hypothetical protein Location= chr11 complement(835888..837465)

ıg) protein kinase, putative Location= pfal_chr2 join(720436..721653, 721894..721993, 722143..722261, 722394..722471, 722601..722660)

ıg) hypothetical protein Location= chr10 join(1257382..1258472, 1259132..1259309)

ling) integral membrane protein Location= chr6 complement(join(542691..543916, 544092..544260, 544599..544793, 540072..542525))

ıg) hypothetical protein Location= chr14 205559..208876

ıg) hypothetical protein Location= chr11 join(1422741..1422813, 1422975..1426198)

ling) gamma-glutamylcysteine synthetase Location= chr9 777548..780739

ling) hypothetical protein Location= chr13 complement(1204387..1207401)

ling) topoisomerase I Location= chr5 complement(445981..448500)

ling) cullin-like protein, putative Location= chr8 555558..557972

ling) phosphatidylglycerophosphate synthase Location= chr8 complement(913360..915345)

ng) rhoGAP protein Location= chr10 complement(join(289170..290310, 290443..290556, 290738..290826, 288383..289000))

ling) coronin Location= chr12 join(2092072..2092105, 2092248..2092339, 2092534..2094216)

ng) protein disulfide isomerase, putative Location= chr14 complement(join(2963118..2963224, 2963352..2963679, 2963881..2965023, 2962923..29

ling) hypothetical protein, conserved Location= chr8 complement(join(526420..526594, 526767..528078, 528363..528384, 526179..526307))

ling) microfibril-associated protein homologue, putative Location= chr13 complement(join(1001547..1001796, 1001958..1002083, 1000301..100139

ling) hypothetical protein Location= chr9 join(1405181..1405852, 1406145..1407011)

ling) DEAD/DEAH box helicase, putative Location= chr9 complement(join(152389..159540, 151699..152157))

ng) hypothetical protein Location= chr10 join(757794..764822, 764969..765157)

ling) hypothetical protein Location= chr7 join(1048610..1054798, 1055003..1055166, 1055295..1055365, 1055463..1055611)

ling) asparagine-rich antigen Location= chr8 complement(784038..790745)

ng) hypothetical protein Location= chr11 complement(join(795630..798818, 794253..795458))

ling) alpha adaptin-like protein, putative Location= chr6 join(710056..712664, 712877..713419, 713556..713724, 713845..714045, 714150..714191,

ng) heat shock protein, putative Location= chr14 1797231..1800014

ling) hypothetical protein Location= chr13 2560960..2563443

ng) vacuolar protein-sorting protein VPS45, putative Location= pfal_chr2 675430..677598

ling) hypothetical protein Location= chr13 2180370..2182283

ng) hypothetical protein Location= chr10 complement(515720..517606)

ling) hypothetical protein Location= chr12 join(1263650..1263838, 1263918..1265582)

ling) hypothetical protein Location= chr4 complement(join(131837..131920, 130044..131687))

ling) nucleoside transporter 1 Location= chr13 complement(1886184..1887452)

ng) antigen 332, putative Location= chr11 1952200..1968723

ling) hypothetical protein Location= chr13 complement(join(256975..267384, 256632..256799))

ling) E1-E2_ATPase/hydrolase, putative Location= chr9 complement(join(225488..232829, 225017..225366))

ling) hypothetical protein Location= chr12 join(256364..261994, 262188..262347, 262507..263412, 263571..263728)

ling) hypothetical protein Location= chr13 2368215..2374289

ling) hypothetical protein Location= chr12 1994761..2000730

ling) hypothetical protein Location= chr7 253050..258629

ig) hypothetical protein Location= chr14 join(2771037..2771186, 2771280..2771377, 2771522..2776244)

ling) hypothetical protein Location= chr7 complement(177878..182500)

ling) Zinc finger transcription factor (krox1) Location= chr12 complement(428489..432874)

ling) hypothetical protein Location= chr13 849863..854191

ig) hypothetical protein Location= chr14 484438..487308

ig) hypothetical protein Location= chr14 2214846..2217731

ling) heat shock protein 86 family protein Location= chr7 complement(440963..443701)

ig) hypothetical protein Location= chr11 complement(1427008..1429668)

ig) replication factor C subunit 1, putative Location= pfal_chr2 complement(779416..782130)

ıg) protein serine/threonine phosphatase Location= chr14 join(2690440..2693024, 2693178..2693262)

ıg) hypothetical protein Location= chr11 complement(1195986..1198307)

ling) hypothetical protein, conserved Location= chr5 complement(288776..291055)

ling) modification methylase-like protein, putative Location= chr7 complement(1215078..1217198)

ling) DNAJ protein Location= chr4 452897..454915

ling) myosin-like protein, putative Location= chr6 complement(579430..585891)

ıg) hypothetical protein Location= chr11 complement(join(1795609..1795637, 1789320..1795518))

ıg) hypothetical protein Location= chr10 complement(join(850167..850469, 851020..856283, 849790..850027))

ıg) hypothetical protein Location= chr14 complement(1437454..1442820)

ıg) hypothetical protein Location= chr14 complement(join(2099547..2099673, 2094277..2098664))

ling) alanine--tRNA ligase, putative Location= chr13 2691989..2696215

ıg) hypothetical protein Location= chr14 complement(176418..179705)

ling) replication licensing factor, putative Location= chr13 2183768..2186557

ıg) cytidine triphosphate synthetase Location= chr14 400418..402994

ling) conserved protein, putative Location= chr13 complement(1724848..1727028)

ling) hypothetical protein Location= chr5 complement(1186780..1188939)

ıg) hypothetical protein Location= chr14 complement(2477764..2479671)

ıg) hypothetical protein Location= chr11 join(962155..963540, 963648..963742, 963861..963957)

ling) hypothetical protein Location= chr13 complement(join(2645772..2645809, 2635290..2645700))

ling) u5 small nuclear ribonucleoprotein-specific protein, putative Location= chr4 1033769..1042393

ling) hypothetical protein Location= chr8 complement(736801..742302)

ling) splicing factor 3b, subunit 3, 130kD, putative Location= chr12 join(1452593..1456102, 1456232..1456324, 1456420..1456566, 1456687..14567

ling) hypothetical protein Location= chr12 complement(405603..409145)

ling) hypothetical protein Location= chr4 complement(join(448044..448127, 448266..451139, 447865..447939))

ıg) hypothetical protein Location= chr11 join(400686..403118, 403432..403628, 403859..403904)

ıg) hypothetical protein Location= chr14 complement(join(1779426..1779549, 1776901..1779236))

ling) hypothetical protein, conserved Location= chr6 873807..876188

ıg) hypothetical protein Location= chr11 join(1309211..1310738, 1310935..1311791)

ig) hypothetical protein Location= chr14 join(2441281..2442967, 2443114..2443832)

ling) ATP-dependent RNA helicase, putative Location= chr12 complement(1093533..1095761)

ig) hypothetical protein Location= chr11 complement(531176..533284)

ig) hypothetical protein Location= chr11 complement(1681865..1690081)

ling) protein kinase, putative Location= chr9 join(1043532..1043639, 1043785..1043888, 1043995..1044062, 1044231..1047784)

ig) hypothetical protein Location= chr14 complement(838922..842659)

ling) hypothetical protein Location= chr13 complement(2011698..2015132)

ig) coat protein, gamma subunit, putative Location= chr11 complement(1783354..1786560)

ig) vacuolar sorting protein 35, putative Location= chr11 join(422057..422188, 422398..425418)

ig) hypothetical protein Location= chr10 705536..708451

ling) hypothetical protein, conserved Location= chr3 complement(join(252338..252481, 252603..255091, 251951..252188))

ling) DNA-binding chaperone, putative Location= chr12 670440..673259

ig) Myb2 protein Location= chr10 complement(1344148..1346895)

ling) hypothetical protein Location= chr9 join(168491..168736, 168924..169031, 169163..169300, 169703..170146, 170269..170362, 170450..1705

ling) hypothetical protein Location= chr3 join(503330..503355, 503560..503778, 503937..505521)

ling) Plasmodium falciparum reticulocyte binding protein 2 homolog b Location= chr13 complement(1429072..1438611)
ling) reticulocyte binding protein 2 homolog a Location= chr13 join(1440977..1441034, 1441249..1450583)

ling) hypothetical protein Location= chr3 complement(join(325667..325791, 325927..326025, 326187..326239, 326354..331416, 331634..331701, 3

ling) serine/threonine protein kinase, putative Location= chr3 110750..114766

ling) hypothetical protein Location= chr9 join(1084686..1085373, 1085490..1088785)

ıg) hypothetical protein Location= chr14 join(2957411..2957484, 2957646..2961132, 2961271..2961555)

ling) hypothetical protein Location= chr9 162768..166922

ling) cell division cycle ATPase, putative Location= chr7 complement(542661..546350)

ıg) hypothetical protein Location= chr11 1313989..1317171

ıg) hypothetical protein, conserved Location= chr14 complement(2128104..2131193)

ıg) hypothetical protein Location= chr11 complement(join(1659732..1662356, 1659067..1659480))

ling) hypothetical protein Location= chr13 complement(1660843..1663761)

ling) hypothetical protein Location= chr7 join(797388..800150, 800232..800279)

ıg) N-acetyl glucosamine phosphate mutase, putative Location= chr11 complement(1159364..1162186)

ling) hypothetical protein Location= chr6 join(1279823..1279930, 1280394..1283075, 1283293..1283343)

ling) hypothetical protein Location= chr13 686153..688873

ıg) hypothetical protein Location= chr11 1239898..1242546

ling) hypothetical protein, conserved Location= chr5 complement(620808..623354)

ling) hypothetical protein, conserved Location= chr6 complement(471653..474145)

ling) N2,N2-dimethylguanosine tRNA methyltransferase, putative Location= chr13 complement(796140..798554)

ıg) hypothetical protein Location= chr10 1341644..1343773

ling) hypothetical protein Location= chr7 235413..246092

ling) hypothetical protein Location= chr6 complement(join(788714..788784, 788928..789030, 789179..789561, 789688..789799, 789939..790000, 7

ling) adapter-related protein, putative Location= chr6 complement(548275..552366)

ling) hypothetical protein Location= chr13 complement(1787370..1791104)

ling) hypothetical protein Location= chr4 492729..496625

ling) hypothetical protein Location= chr9 complement(join(1279418..1279474, 1279586..1282846, 1283133..1283511, 1279209..1279255))

ıg) hypothetical protein Location= chr14 complement(786957..790457)

ling) bromodomain protein, putative Location= chr12 complement(568562..571828)

ling) hypothetical protein Location= chr12 join(2059092..2061321, 2061725..2061924, 2062022..2062169, 2062348..2062459, 2062688..2062856, [illegible]

ıg) hypothetical protein Location= chr14 complement(1633154..1636198)

ling) hypothetical protein Location= chr13 complement(2220479..2223373)

ıg) glucose-6-phosphate dehydrogenase-6-phosphogluconolactonase Location= chr14 complement(2209728..2212460)

ıg) hypothetical protein Location= chr10 complement(join(1174759..1174837, 1174965..1174995, 1172127..1174533))

ling) hypothetical protein Location= chr9 1254805..1257282

ling) hypothetical protein Location= chr6 complement(join(940355..940410, 940512..940584, 940710..940784, 940893..940954, 941156..941836, 9

ling) hypothetical protein Location= chr6 join(566134..567816, 567859..574990, 575311..577827, 578033..578988)

ling) hypothetical protein Location= chr13 join(2083724..2088498, 2088895..2088940)

ıg) regulator of nonsense transcripts, putative Location= chr10 complement(join(241592..242245, 237507..241517))

ling) p-type Atpase2 Location= chr12 complement(join(785804..785878, 786016..790465, 790617..790666, 785564..785656))

ıg) hypothetical protein Location= chr11 1762253..1766647

ling) hypothetical protein Location= chr13 complement(join(471032..471150, 471278..475204, 470797..470908))

ling) hypothetical protein Location= chr7 706212..710297

ling) hypothetical protein Location= chr8 1153374..1157354

ıg) hypothetical protein Location= chr14 913764..917336

ling) hypothetical protein Location= chr12 complement(1024446..1028009)

ling) hypothetical protein Location= chr8 join(561865..562138, 562270..565397)

ling) hypothetical protein Location= chr13 complement(165215..168283)

ling) papain family cysteine protease, putative Location= chr9 complement(join(123794..123934, 124142..124738, 124972..125005, 121620..12364

ling) hypothetical protein Location= chr12 join(1925628..1925849, 1926024..1926354, 1926491..1926584, 1926693..1926771, 1926901..1927483,

ling) hypothetical protein Location= chr12 1259609..1262077

ıg) hypothetical protein Location= chr10 complement(1095002..1097275)

ıg) hypothetical protein Location= chr14 complement(join(1327644..1329519, 1312246..1327055))

ding) asparagine/aspartate rich protein, putative Location= chr12 join(1385694..1401818, 1401920..1402076, 1402168..1402205)

ıg) hypothetical protein Location= pfal_chr2 complement(join(495882..501641, 501855..507854, 495166..495681))

ıg) erythrocyte membrane-associated antigen Location= chr10 complement(join(721918..728331, 729860..732855, 721733..721820))

ıg) hypothetical protein Location= pfal_chr2 complement(182228..189115)

ding) hypothetical protein Location= chr13 complement(1590705..1596515)

ding) ABC transporter, putative Location= chr1 465876..471344

ıg) Ser/Thr protein kinase, putative Location= pfal_chr2 594196..599340

ding) hypothetical protein Location= chr5 join(1199158..1201849, 1202014..1202168, 1202207..1202600, 1202816..1202868, 1202989..1203528, 1[illegible]

ding) phospholipid-transporting ATPase, putative Location= chr12 join(941116..944793, 944940..945242, 945440..945793, 945917..946224, 94638[illegible]

ding) hypothetical protein Location= chr7 complement(1034298..1038566)

ding) bi-functional enzyme: long-chain fatty-acid Co-A ligase and oxalyl Co-A decarboxylase, putative Location= chr6 complement(820066..824244)

ding) cg1 protein Location= chr7 463595..467341

ling) hypothetical protein Location= chr7 complement(join(1056247..1056380, 1056491..1056588, 1056742..1060124, 1056079..1056132))

ıg) U5 small nuclear ribonuclear protein, putative Location= chr10 complement(172724..176431)

ling) cyclin g-associated kinase, putative Location= chr12 1959598..1962327

ling) ubiquitin carboxyl-terminal hydrolase, putative Location= chr7 complement(join(1182152..1182320, 1182499..1182567, 1182698..1191955, 11

ling) hypothetical protein, expressed Location= chr6 complement(270550..277758)

ıg) hypothetical protein Location= chr14 complement(1467572..1474381)

ıg) hypothetical protein Location= chr11 complement(join(1281730..1287631, 1281282..1281598))

ling) hypothetical protein Location= chr13 join(931515..936514, 936720..937000, 937137..937233, 937389..937640, 937727..937860, 937956..938

ıg) hypothetical protein Location= chr11 join(1087312..1091827, 1091931..1092088, 1092247..1092504, 1092666..1093103)

ling) hypothetical protein Location= chr8 join(113422..114634, 114748..118751)

ling) hypothetical protein Location= chr4 complement(1090419..1095512)

ıg) hypothetical protein Location= chr14 join(2400451..2404653, 2404786..2404850, 2405011..2405129, 2405325..2405347)

ıg) S-adenosylmethionine decarboxylase-ornithine decarboxylase Location= chr10 complement(1324130..1328434)

ıg) hypothetical protein Location= chr10 complement(join(1057928..1060656, 1055742..1057014))

ling) hypothetical protein Location= chr9 complement(join(332292..334594, 330496..331978))

ling) cullin-like protein, putative Location= chr6 complement(join(1247171..1247222, 1243583..1246920))

ling) hypothetical protein Location= chr3 complement(585679..589224)

ling) methyltransferase, putative Location= chr13 complement(join(2160908..2163749, 2160572..2160789))

ling) hypothetical protein Location= chr7 307664..310633

ling) hypothetical protein Location= chr12 complement(1852832..1855669)

ig) hypothetical protein Location= chr14 2314316..2321491

ig) histone deactylase, putative Location= chr14 join(2945599..2952311, 2952446..2952488)

ling) hypothetical protein Location= chr4 complement(679483..686121)

ling) hypothetical protein Location= chr13 2065648..2071668

ling) hypothetical protein Location= chr4 391851..397733

ling) hypothetical protein Location= chr7 170608..176139

ling) hypothetical protein Location= chr12 join(1594814..1600072, 1600197..1600267, 1600406..1600514)

ig) hypothetical protein Location= chr11 678751..683280

ling) ATP-dependent RNA helicase, putative Location= chr5 358261..362733

ling) hypothetical protein Location= chr13 join(2055947..2055994, 2056169..2056228, 2056407..2060255)

ling) trancription factor, putative Location= chr6 complement(456742..460584)

ling) hypothetical protein Location= chr6 complement(133147..136458)

ling) hypothetical protein Location= chr4 complement(497339..500272)

ling) cholinephosphate cytidylyltransferase Location= chr13 join(691216..691251, 691569..691647, 691752..694327)

ıg) transmission-blocking target antigen s230 precursor Location= pfal_chr2 370438..379845

ling) hypothetical protein Location= chr7 complement(join(326258..334089, 325405..326017))

ling) hypothetical protein, conserved Location= chr13 2097818..2105419

ling) hypothetical protein Location= chr13 complement(join(544661..544725, 536904..543924))

ling) hypothetical protein, conserved Location= chr3 179407..186087

ling) hypothetical protein Location= chr9 complement(706582..712062)

ling) hypothetical protein Location= chr8 568944..574268

ıg) hypothetical protein Location= chr11 complement(1227218..1231948)

ling) hypothetical protein Location= chr13 complement(join(1813155..1813669, 1813892..1814050, 1814166..1817638, 1812682..1813031))

ling) hypothetical protein Location= chr8 join(411622..415452, 415594..415831, 416094..416290)

ıg) hypothetical protein Location= chr10 132996..137273

ling) hypothetical protein Location= chr12 415398..419726

ıg) hypothetical protein Location= pfal_chr2 complement(join(129605..129690, 129768..133563, 129342..129521))

ling) hypothetical protein Location= chr13 complement(join(517259..517528, 513270..517001))

ling) exoribonuclease, putative Location= chr9 424163..428098

ıg) CCAAT-box DNA binding protein subunit B Location= chr11 join(1834398..1837922, 1838086..1838161, 1838271..1838323, 1838519..1838770)

ling) hypothetical protein Location= chr6 complement(250609..253800)

ling) hypothetical protein Location= chr13 complement(join(1981858..1991945, 1981668..1981725))

ling) hypothetical protein Location= chr5 367022..376426

ling) hypothetical protein Location= chr12 1634683..1642416

ling) hypothetical protein Location= chr13 complement(join(544661..544725, 536904..543924))

ıg) asparagine-rich protein Location= chr11 join(909365..909469, 909631..916323)

ling) phosphatidylinositol 3-kinase, putative Location= chr5 628979..635380

ling) erythrocyte membrane protein 1 (PfEMP1) Location= chr13 join(21467..26641, 27577..28890)
ling) erythrocyte membrane protein 1 (PfEMP1) Location= chr7 complement(join(624394..629688, 622106..623458))

ling) hypothetical protein Location= chr6 425800..431853

ling) hypothetical protein Location= chr12 1098119..1104058

ling) hypothetical protein Location= chr12 1550312..1556059

ling) hypothetical protein Location= chr13 join(931515..936514, 936720..937000, 937137..937233, 937389..937640, 937727..937860, 937956..938

ling) hypothetical protein Location= chr13 join(1252257..1256286, 1256416..1256485, 1256737..1258171)

ıg) hypothetical protein Location= pfal_chr2 337558..342966

ıg) hypothetical protein Location= chr11 749151..754400

ling) hypothetical protein Location= chr9 join(212513..217732, 217889..217981)

ling) hypothetical protein Location= chr13 440467..445485

ling) hypothetical protein Location= chr7 complement(365283..370403)

ıg) hypothetical protein Location= chr11 complement(951381..956342)

ling) hypothetical protein Location= chr13 join(938868..938892, 938971..939284, 939578..943771)

ling) hypothetical protein Location= chr4 complement(join(278956..279037, 279149..279218, 279372..279752, 279858..283289, 278720..278843))

ling) DNA repair protein rad54, putative Location= chr8 213372..217091

ling) hypothetical protein Location= chr9 complement(join(457132..457195, 457286..457409, 457527..457605, 457765..460742, 460779..461008, 4

ling) hypothetical protein Location= chr7 join(959521..962682, 962922..970049)

ling) Plasmodium falciparum reticulocyte binding protein 2 homolog b Location= chr13 complement(1429072..1438611)
ling) reticulocyte binding protein 2 homolog a Location= chr13 join(1440977..1441034, 1441249..1450583)

ling) hypothetical protein Location= chr13 join(2756090..2756185, 2756302..2759274, 2759404..2764812)

ling) hypothetical protein Location= chr7 complement(1361272..1369593)

ling) DNA-directed RNA polymerase II, putative Location= chr3 745136..752509

ling) hypothetical protein Location= chr12 complement(join(1755853..1755955, 1756170..1756224, 1748860..1755631))

ıg) erythrocyte membrane protein 3 Location= pfal_chr2 complement(join(98728..98838, 91318..98532))

ling) hypothetical protein conserved Location= chr12 complement(836905..843894)

ling) erythrocyte membrane protein 1 (PfEMP1) Location= chr7 complement(join(639021..644303, 636766..638202))

ıg) hypothetical protein Location= pfal_chr2 complement(141625..147564)

ling) integral membrane protein, putative Location= chr7 1231322..1237078

ling) hypothetical protein, conserved Location= chr9 complement(join(301256..301285, 301340..304176, 298662..301218))

ıg) hypothetical protein Location= chr14 618896..623320

ding) hypothetical protein Location= chr5 486600..516785

ıg) hypothetical protein Location= pfal_chr2 join(509525..516077, 516327..521746)

ding) hypothetical protein Location= chr4 complement(join(341081..350972, 340759..340943))

ding) hypothetical protein Location= chr5 join(559511..560683, 560972..567969, 568173..568251, 568341..568382)

ıg) hypothetical protein Location= chr11 complement(1623444..1630271)

ıg) hypothetical protein Location= pfal_chr2 483436..488970

ding) asparagine-rich protein, putative Location= chr12 1314686..1320160

ding) ubiquitin-protein ligase 1, putative Location= chr8 complement(1114441..1140216)

ding) hypothetical protein Location= chr4 join(800808..802122, 802320..804826, 805044..805156, 805322..805407, 805703..805899, 806054..8082

ding) hypothetical protein Location= chr9 join(1224981..1225077, 1225841..1239336)

ıg) hypothetical protein Location= chr10 complement(join(894667..894858, 897436..897528, 897634..903606, 887414..894508))

ıg) hypothetical protein Location= chr10 331246..342807

ıg) hypothetical protein Location= chr14 1753453..1763607

ding) hypothetical protein Location= chr3 complement(696215..706399)

ling) hypothetical protein Location= chr4 complement(join(341081..350972, 340759..340943))

ling) hypothetical protein Location= chr9 complement(809649..819794)

ling) hypothetical protein Location= chr12 complement(join(1363855..1364334, 1364469..1370625, 1360620..1363564))

ling) P. falciparum PK4 protein kinase Location= chr6 1159863..1169081

ling) hypothetical protein Location= chr7 518485..527217

ling) ribonuclease, putative Location= chr6 complement(join(631167..631249, 631319..631408, 631532..631588, 631695..631759, 631894..631945

ıg) hypothetical protein, conserved Location= chr14 complement(1191888..1199861)

ling) hypothetical protein Location= chr1 190507..197586

ling) hypothetical protein Location= chr4 complement(526729..532515)

ling) hypothetical protein Location= chr13 complement(join(380045..381036, 381179..383363, 383499..392275, 373690..380002))

ling) hypothetical protein Location= chr6 370257..388490

ling) hypothetical protein Location= chr7 235413..246092

ıg) hypothetical protein, conserved Location= chr11 163986..174101

ling) hypothetical protein Location= chr6 complement(join(491066..491159, 491278..491424, 491573..491645, 491805..501135, 490853..490954))

ling) hypothetical protein Location= chr8 join(251203..262026, 262398..268658, 268909..269556)

ıg) Pf 11-1 protein Location= chr10 join(1519020..1519231, 1519345..1547824)

ıg) hypothetical protein Location= chr14 complement(join(1392036..1402608, 1378291..1392002))

ıg) hypothetical protein Location= chr14 complement(join(325114..328827, 328867..335721, 335782..346468, 324754..324947))

5..1698144)

[illegible])

353, 1101735..1102213))

16..577054)

'3, 274724..274787, 270738..271405))

[illegible]1, 252622..252680, 252830..252872)

178064..1178384, 1178654..1179241, 1179424..1179534, 1179668..1180497)

591, 2037799..2037886, 2036736..2036786))

2))

)8, 844364..844503, 844567..844574, 843583..843682))

3..134183, 134313..134498, 134579..134737, 134885..135651, 135858..137339)

1357015..1357392)

717..791784))

)

..839408, 837969..838007))

31965, 732086..732259, 732484..732576, 732758..732885, 733064..733123, 733296..733308, 731038..731067))

, 2675749..2675784))

30, 564402..564501, 564613..564695, 564811..564947, 565058..565194, 565302..565527)

1130175))

.1058052, 1056196..1056513))

)9, 858777..858819, 857577..857642))

'8..2121812, 2121948..2122115, 2122349..2122472, 2114613..2118812))

[illegible]816))

28..881262)

152, 1874278..1874347, 1874493..1874734, 1874921..1875045, 1875166..1875275, 1873182..1873205))

.33830))

251159..251224, 251390..251396, 249333..249461))

)8, 424302..424364)

55326..555382, 555546..555638, 555761..555772, 553639..553697))

3..788295))

229171..2229225, 2229350..2229394, 2229516..2229581)

97, 200848..200984, 201101..201173, 201245..201331)

54263..254361))

2011..522760, 523096..523114, 520829..521066))

'..2293279, 2293434..2293489)

0976))

63114, 1363439..1363837)

[illegible]25, 1416082..1416213, 1416362..1416547, 1416911..1417069, 1417481..1418247, 1418460..1419743)

1254460, 1254601..1254696, 1254826..1254867)

23917, 622893..623078))

.3493, 613649..613688, 613831..613872, 613980..614032, 614114..614191, 614326..614388, 614534..614575, 614697..614755, 614868..614970,

578171..678200, 678347..678401, 678543..678574, 676843..676974))

675, 115811..115923)

.195459)

26..2450093)

l7391))

[illegible]3..1794558, 1794668..1794822, 1793519..1793637))

[illegible]08..2005826))

[illegible]34750, 1434910..1434950, 1435092..1435167, 1433138..1433273))

)8..1105479, 1105623..1105694, 1105767..1105854, 1106004..1106102, 1106221..1106338, 1104152..1104266))

'7)

)

l355569, 1354452..1354600))

83080..184457, 181871..181927))

4317..1004625)

078, 2404211..2404473, 2404570..2404718, 2403171..2403227))

83, 2462594..2462647, 2462774..2462833, 2462936..2463040)

44, 1277480..1277531, 1277677..1277723)

65647..2165702))

334, 954491..954663, 954783..954849, 952907..952965))

323..734480, 734823..734952, 732167..732211))

63..377740, 377844..377966, 378305..378452, 376855..377223))

47))

, 607103..607207))

968059, 966785..966869))

381..650545)

l93..635285)

5..785173, 785318..785388, 785508..785628, 785739..785851, 785932..786000, 786144..786225, 786354..786405, 784181..784249))

[illegible]..2438196, 2438316..2438393, 2438553..2438774, 2438872..2439030, 2436771..2437037))

1068281, 1068414..1068479, 1068588..1068674, 1068782..1068875, 1068959..1069040, 1069166..1069223, 1069340..1069408, 1069553..10696[illegible]

.339490, 339654..339918, 340114..340333)

[illegible]0690..160977))

536957, 537113..537260, 537387..537513, 537704..537740, 535590..535631))

)4, 934992..935066)

91303..1291449, 1290410..1290831))

..1821606, 1821851..1822077, 1822301..1822387, 1822517..1822582, 1820868..1820912))

2256137, 2256218..2256281, 2256438..2256501, 2256613..2256677)

08121..808165, 808273..808329, 808469..808501, 808632..808661, 808759..808791, 808930..808968, 809108..809182, 809281..809499, 80967

.2348528, 2348628..2348696, 2348822..2348921, 2349146..2349153, 2347583..2347687))

9))

6199..306282, 306573..306623)

3, 2119860..2120028, 2120578..2120588, 2118883..2118930))

, 1159195..1159276, 1159393..1161547, 1157834..1157893))

[illegible]728..248833, 248996..249163)

.460020, 460144..460219, 460316..460398, 460526..460576, 460714..460770, 460919..461011, 461205..461249, 461397..461451, 461621..4616[illegible]

6))

280953..1281483, 1279998..1280135))

..1273393, 1273613..1273697, 1273806..1273852, 1273974..1274057)

404..1171460, 1171617..1171689, 1171906..1171973, 1172139..1172225, 1172353..1172421, 1172576..1172614, 1172748..1172810, 1172943..1[illegible]

2701..122994, 123268..123292)

2096190..2096272, 2096441..2096493, 2095047..2095124))

.630244))

.1448741))

718, 1186882..1186984)

7573..788202)

[illegible]937..1650236, 1650360..1650477, 1650640..1650680, 1649144..1649211))

5003, 2915196..2915291)

?2, 558076..558307, 558501..558667)

.493766, 1494129..1494157, 1490597..1492453))

)81, 522189..522559, 522642..522898)

2..950581)

.1049758, 1045606..1045626))

32, 1036744..1036787))

4939..575644))

20..153536, 153602..155476, 148237..148341))

3062569)

113957..114010, 114153..114725, 114870..115698, 110394..111815))

)72975..1073102, 1073197..1073289, 1073388..1073432, 1073580..1073619, 1073753..1073811)

40, 449542..449970, 450252..450433)

9..2030340))

.2586335, 2586455..2586571, 2586695..2586823, 2586980..2587073, 2587198..2587365, 2587489..2587671, 2587832..2587938, 2588062..2589

[illegible]293..866412, 866497..866740)

)5..937330, 937525..937604, 937750..937910, 938006..938086, 938277..938794, 938906..939063, 939354..939658, 939854..940210, 940516..94(

.77454, 177574..177637, 177765..177803)

ꞏ63006))

7))

714303..714361, 714486..714573)

788, 1457077..1457214)

49, 170711..170773, 170916..171021, 171136..171252, 171343..171753, 171885..171947, 172098..172199, 172431..172498, 172584..172718, 17

[illegible]31918..331944, 325462..325507))

'90106..790173, 790289..790379, 790479..790553, 790723..794537, 794729..794834, 794959..795107, 795260..795344, 795501..795675, 79578[illegible]

2062951..2063211)

941951..942006, 942217..942292, 942480..942855, 943068..943111, 943232..943385, 943538..943720, 939658..940182))

6))

1927624..1928327, 1928426..1928605, 1928757..1928818, 1928914..1929112, 1929212..1929424)

203610..1203798, 1204002..1204367, 1204569..1204667, 1204773..1205180)

3..946470, 946611..946736)

81934..1181989))

005)

)

005)

·61257..461309, 456950..457009))

67, 808426..810950, 811150..816685)

, 632046..632123, 632220..636338, 636540..640169, 630965..631034))

615181..615372)

04, 1069702..1069759, 1069897..1069996, 1070119..1070217)

[illegible]..810053, 810119..810559, 810783..811173, 811373..811566, 811691..811813, 811928..812008, 812099..812221, 812366..812446, 812589..812[illegible]

97)

173038, 1173216..1173356, 1173563..1173697, 1170473..1170517))

591, 2589685..2589717, 2584735..2584887))

)619, 940769..940873, 941003..941163, 941460..941583)

2852..172885)

2..795909, 788220..788406))

654, 812852..812891, 812927..813087, 813241..813429, 813578..813604, 813781..813813, 814098..814211, 814347..814424, 814531..814591, 8[illegible]

06923..807035))
